# Synthetic macrolides overcoming MLS_B_K-resistant pathogens

**DOI:** 10.1038/s41421-024-00702-y

**Published:** 2024-07-11

**Authors:** Cong-Xuan Ma, Ye Li, Wen-Tian Liu, Yun Li, Fei Zhao, Xiao-Tian Lian, Jing Ding, Si-Meng Liu, Xie-Peng Liu, Bing-Zhi Fan, Li-Yong Liu, Feng Xue, Jian Li, Jue-Ru Zhang, Zhao Xue, Xiao-Tong Pei, Jin-Zhong Lin, Jian-Hua Liang

**Affiliations:** 1 Key Laboratory of Medicinal Molecule Science and Pharmaceutical Engineering, School of Chemistry and Chemical Engineering, Beijing Institute of Technology, Beijing, China; 2 State Key Laboratory of Genetic Engineering, School of Life Sciences, Zhongshan Hospital, Fudan University, Shanghai, China; 3 Center for mRNA Translational Research, Fudan University, Shanghai, China; 4 Institute of Clinical Pharmacology, Peking University First Hospital, Beijing, China; 5 National Institute for Communicable Disease Control and Prevention, Chinese Center for Disease Control and Prevention, State Key Laboratory of Infectious Disease Prevention and Control, Beijing, China

**Keywords:** Cryoelectron microscopy, Ribosome

## Abstract

Conventional macrolide-lincosamide-streptogramin B-ketolide (MLS_B_K) antibiotics are unable to counter the growing challenge of antibiotic resistance that is conferred by the constitutive methylation of rRNA base A2058 or its G2058 mutation, while the presence of unmodified A2058 is crucial for high selectivity of traditional MLS_B_K in targeting pathogens over human cells. The absence of effective modes of action reinforces the prevailing belief that constitutively antibiotic-resistant *Staphylococcus aureus* remains impervious to existing macrolides including telithromycin. Here, we report the design and synthesis of a novel series of macrolides, featuring the strategic fusion of ketolide and quinolone moieties. Our effort led to the discovery of two potent compounds, MCX-219 and MCX-190, demonstrating enhanced antibacterial efficacy against a broad spectrum of formidable pathogens, including A2058-methylated *Staphylococcus aureus*, *Streptococcus pneumoniae*, *Streptococcus pyogenes*, and notably, the clinical *Mycoplasma pneumoniae* isolates harboring A2058G mutations which are implicated in the recent pneumonia outbreak in China. Mechanistic studies reveal that the modified quinolone moiety of MCX-190 establishes a distinctive secondary binding site within the nascent peptide exit tunnel. Structure-activity relationship analysis underscores the importance of this secondary binding, maintained by a sandwich-like π–π stacking interaction and a water–magnesium bridge, for effective engagement with A2058-methylated ribosomes rather than topoisomerases targeted by quinolone antibiotics. Our findings not only highlight MCX-219 and MCX-190 as promising candidates for next-generation MLS_B_K antibiotics to combat antibiotic resistance, but also pave the way for the future rational design of the class of MLS_B_K antibiotics, offering a strategic framework to overcome the challenges posed by escalating antibiotic resistance.

## Introduction

The bacterial 70S ribosome serves as the universal translator for protein synthesis and is a vital target for antibacterial agents. This is because more than half of antibiotics bind to different sites on the ribosome, thereby selectively disrupting the process of protein synthesis^1,2^. The World Health Organization has identified twelve lethal pathogenic species, including methicillin-resistant *Staphylococcus aureus* (MRSA), penicillin-resistant *Streptococcus pneumoniae* and ampicillin-resistant *Haemophilus influenzae*, as high- to medium-priority resistant bacteria which require immediate attention. Macrolide-lincosamide-streptogramin B (MLS_B_), one of the most successful classes of antibiotics, was widely prescribed to treat infections caused by those pathogens. However, multi-drug-resistant pathogens pose a significant threat to human health and reduce the effectiveness of currently available MLS_B_ antibiotics^3–6^.

Structurally unrelated MLS_B_ antibiotics are positioned at the narrowest site within the nascent peptide exit tunnel (NPET) of the ribosome, close to the catalytic peptidyl transferase center (PTC). Clinically, it is observed that alteration of bacterial ribosomal RNA (rRNA) bases renders bacteria insensitive to the available arsenal of ribosome-targeting antibiotics. It has been clarified that the most prevalent mechanism of cross-resistance to MLS_B_ is mono- or di-methylation of the N6 position of rRNA base A2058 (*Escherichia coli* numbering is used throughout the text unless concurrent *S. aureus* numbering indicated in parentheses), which is catalyzed by erm-mediated methyltransferases^7,8^. In contrast to the low-level efflux resistance encoded by *mef* genes, methylation at A2058 and mutation of A2058 can lead to high-level antibiotic resistance, either induced by erythromycin (inducible phenotype) or occurring in the absence of erythromycin (constitutive phenotype).

**Fig. 1:**
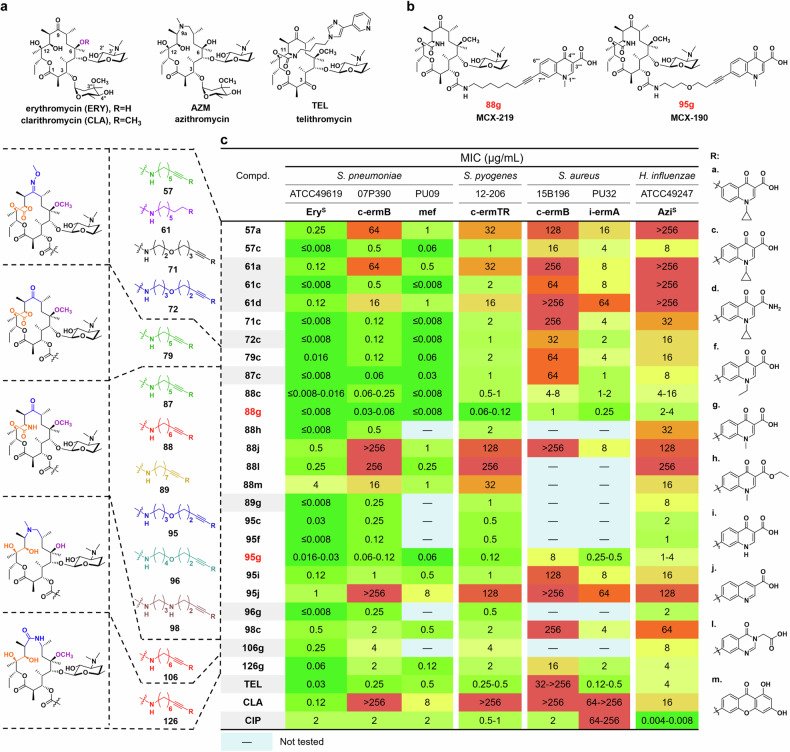
The SARs of new hybrids of macrolides and quinolones. **a** Structures of the first-, second- and third-generation marketed erythromycins. **b** Structures of the representative macrolones MCX-219 and MCX-190. **c** MICs (μg/mL) of the selected synthetic macrolides. Ery^S^ erythromycin sensitive, AZM^S^ azithromycin sensitive, TEL telithromycin, CLA clarithromycin, CIP ciprofloxacin. The strains 07P390, PU09, 12-206, 15B196, and PU32 are erythromycin-resistant clinical isolates encoding various resistant genes.

Resistance to MRSA is often associated with constitutive phenotypes of MLS_B_ rather than inducible phenotypes of MLS_B_^9^, necessitating the development of new MLS_B_ actively against constitutive resistance. Nevertheless, there is a long-standing notion that constitutively resistant *S. aureus* cannot be effectively overcome by currently available macrolides, which was first claimed by scientists at Pfizer in 2007^9–11^. To revitalize MLS_B_, unnatural MLS_B_ scaffolds which are not accessible by semisynthetic method, approaches have been constructed through a convergent and efficient total synthesis method that enables the synthesis of diverse structures^12–14^. The availability of unnatural scaffolds significantly expands the chemical space for drug design of newer MLS_B_. However, the optimized macrolides, regardless of their varying macrolactone ring sizes, still demonstrate inactivity against constitutively A2058-methylated *S. aureus*^12^. Recently, cresomycin, a novel lincosamide with an additional isobutyl substituent, restored activity against A2058-methylated *S. aureus* by extending the isobutyl substituent to the A-site of PTC and disrupting translation initiation^14^. On the other hand, infections caused by MLS_B_-resistant *Mycoplasma pneumoniae*, occurring both endemically and epidemically worldwide, have received less attention^3,15^. *M. pneumoniae* is a unique bacterium as it lacks a cell wall, making it resistant to certain antibiotics that target cell walls. In 2023, the prevalence of azithromycin-resistant *M. pneumoniae* as a first-line treatment challenge has led to an extended outbreak among children in China, primarily due to the lack of safe and efficacious antibiotics for combating the infection. Notably, *M. pneumoniae*, which carries a single chromosomal rRNA operon (in contrast to six *rrn* alleles in the conventional *S. aureus* strains), exhibits resistance to the first-, second-, and third-generation macrolides currently available in the market (such as erythromycin, clarithromycin/azithromycin and telithromycin, respectively, Fig. 1a) by the G2058 mutation in rRNA^15^. Intriguingly, eukaryotic ribosomes have a conserved G2058 at the equivalent position, serving as a determinant contributing to the selective action of MLS_B_ antibiotics on bacterial ribosomes^16^. Thus far, A2058-modified MLS_B_ resistance remains a great challenge for the development of the next generation of MLS_B_.

Macrolides, consisting of a 14–16 membered macrolactone and several sugars, are best represented by erythromycin, which has been safely used for more than 70 years to treat community-acquired or hospital-acquired respiratory tract infections caused by *S. aureus*, *S. pneumoniae*, *Streptococcus pyogenes*, *M. pneumoniae*, *H. influenzae* and *Moraxella catarrhalis*. The prevalence of resistance has been exacerbated by the second-generation cladinose-containing erythromycins, such as clarithromycin and 15-membered azithromycin. To address the issue of cladinose-induced A2058 methylation and efflux resistance, a direct approach comprises eliminating the C-3 cladinose and introducing a validated non-inducible group^17–21^. Telithromycin and its analog solithromycin, the representative ketolides structurally characterized by the 3-ketone and additional alkyl-aryl arms extending from the 11, 12-position of the lactone, demonstrate potency against constitutively methylated strains of *S. pneumoniae* and *S. pyogenes* by providing an additional π–π contact with rRNA base pair A752–U2609^22–24^. Nevertheless, a comparative analysis of the structure of telithromycin in complex with the *S. aureus* ribosome revealed that the aryl group anchored at the end of the side chain of telithromycin is unable to interact with rRNA bases of *S. aureus*, rationalizing the observed ineffectiveness of macrolides against constitutively methylated *S. aureus*^25^. Notably, the development of ketolides is hindered by unfavorable impacts, such as liver damage, named the Ketek (telithromycin) effect, as well as the weak inducer due to a ribosomal frameshifting mechanism^26^. Moreover, a significant proportion of these ketolide-sensitive *S. pyogenes* and *S. pneumoniae* remains rRNA A2058-monomethylated, and the inducer would further increase the proportion of dimethylated A2058 ribosomes, which confers high resistance to ketolides^27^. This highlights the difficulties in developing effective strategies to address MLS_B_K resistance^28^.

Hybridizing two pharmacophores is a promising therapeutic strategy in drug discovery and several hybrids are under clinical evaluation^29,30^. Initially, attaching the fluoroquinolone element to the 4”-position of C-3 cladinose of macrolides was developed by ref. ^31^. Thus, many so-called macrolones (a hybrid of the macrolide and the quinolone, mostly characterized with an extended linker originating from the cladinose) have been extensively investigated^11,28,31–44^. Thus far, only one of the C-6-derived 15-membered macrolones has shown moderate activity against an isolate of constitutively MLS_B_-resistant but quinolone-sensitive *S. aureus*^11^. Nonetheless, it remains unclear whether its antibacterial mechanism involves action on topoisomerase^43^, especially given that the tested strain is sensitive to quinolones, and no further biological analysis on potential targets has been provided. Hence, the approach to improve the efficacy of macrolones in inhibiting A2058-modified *S. aureus* and *M. pneumoniae* cell growth remains unidentified.

In this work, we have synthesized a novel class of synthetic macrolides, representing a unique hybridization of ketolide and quinolone moieties. The lead compounds, MCX-219 and MCX-190, featuring a modified quinolone motif and an effective hybrid approach, demonstrate a revived antibacterial spectrum against a range of microorganisms resistant to MLS_B_K, including *S. aureus*, *S. pneumoniae*, *S. pyogenes*, and *M. pneumoniae*, as well as the Gram-negative *H. influenzae* and *M. catarrhalis*. We provide structural and biochemical evidence that elucidates the mechanisms underpinning the robust and extensive antibacterial activity of these newly developed macrolides, specifically targeting the A2058-modified ribosome in a manner that bears resemblance to targeting topoisomerase. This discovery would be instrumental in formulating the other MLS_B_K antibiotics.

## Result

### Discovery of MCX-219 and MCX-190 guided by structure-activity relationships (SARs)

The known SARs of macrolones exhibit notable distinctions from those of quinolones targeting topoisomerases. For example, a majority of macrolones are consistently constructed by linking through the C-4”-position of the macrolide and the C-6-position of the quinolone nucleus because slightly favorable effects on activity were observed when substituting positions shifted from C-7 to C-6 of quinolones^34,37,39–41^. Moreover, 3-esterification of quinolone in macrolones resulted in an acceptable increase in minimum inhibitory concentrations (MICs)^34,36,38^. In contrast, this work underscores the significance of substituting the linker at the C-7 position of quinolones (57c vs 57a, 61c vs 61a) and maintaining the original chemotype of 3-carboxylic acid of quinolones (88h vs 88g, 61c vs 61d), as shown in Fig. 1c. This disparity can be attributed to the limited knowledge of SARs when a quinolone is attached to the 3-*O* position of 3-*O*-descladinosyl macrolides. Therefore, the SARs discovered in this work differ significantly from those of macrolones but share similarities with the established SARs of quinolones. Additionally, a comparison of the rigid alkyne linker in 57c vs the flexible alkyl linker in 61c demonstrates the favorability of the rigid linker in enhancing activity against fastidious *H. influenzae*.

Further exploration into varying functionality at the 9- or/and 11-positions (57c vs 79c vs 87c), varying linkers’ length (87c vs 88c, 88g vs 89g, 95g vs 96g), and the insertion of heteroatoms such as oxygen or nitrogen atoms into the linkers connecting macrolides to quinolones (71c vs 72c, 88g vs 95g, 89g vs 96g, 95c vs 98c) was performed, as detailed in Fig. 1c. Among the hybrids, compound 88g, characterized by 11,12-cyclic carbamate, 9-keto functionality, and the introduction of a new spacer of octynyl carbamate at C-3, exhibited the highest activity against constitutively MLS_B_K-resistant *S. pneumoniae*, *S. pyogenes*, and *S. aureus*.

The significantly increased MICs of 95j and 88j possessing 3-quinolylcarboxylic acid emphasized the importance of the C-4 ketone of quinolones, as shown in Fig. 1c. Further exploration of quinolone-like scaffolds, such as quinazolin-4(*3H*)-one and 1,3-dihydroxylxanthone proved unsuccessful, indicating the importance of quinolones (88g vs 88j, 88l and 88m). However, varying groups at N-1 of quinolones, such as H atom, or methyl, ethyl, and bulky cyclic propyl groups (88c vs 88g, 95c, 95f, and 95g vs 95i), indicated that the sterically smaller methyl group at N-1 is the best substituent for improving activities (88g and 95g, Fig. 1c), which is different from the established SARs for the antibiotics of quinolones. Remarkably, 88g and 95g (coded MCX-219 and MCX-190, Fig. 1b) exhibited superior activities over telithromycin. We reasoned that the presence of an unsubstituted 1-NH in quinolones can lead to the formation of a tautomeric isomer known as 3-carboxy-4-hydroxyquinoline, which may be unfavorable for chelating function with a cation, such as Mg^2+^, as depicted in the mode of action of quinolones (95i vs 95g)^45^.

A consistent trend was observed regarding the variation in linkers and quinolones on the 15-membered macrolide scaffold, for example, azithromycin, 8a-lactam, and 9a-lactam. Among them, 106g (azithromycin derivative) and 126g (8a-lactam derivative), both possessing the same linker and anchor as 88g (MCX-219), were the most potent, but less potent than the 14-membered MCX-219 and MCX-190. Detailed SARs are presented in Supplementary Fig. S1 and Tables S1–S8.

### MCX-219 and MCX-190 overcome MLS_B_ resistance across pathogenic species

**Fig. 2:**
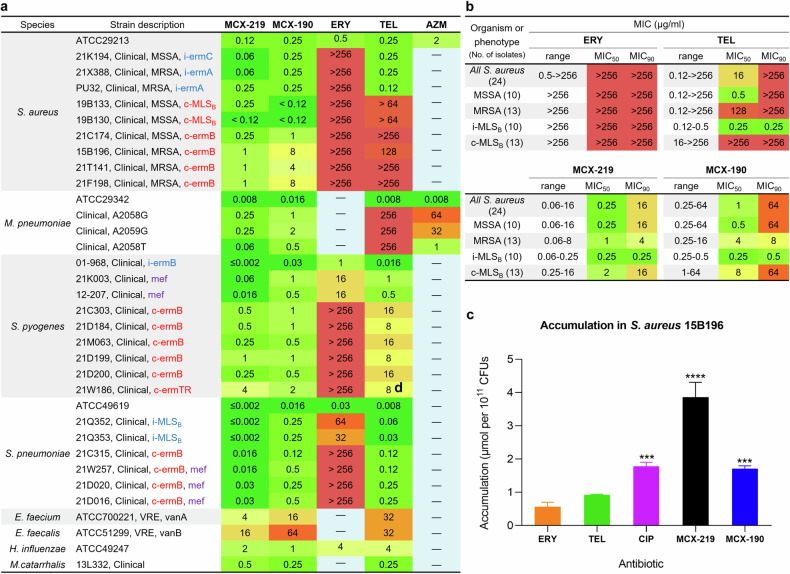
MCX-219 and MCX-190 are active against a variety of resistant clinical isolates and highly accumulative inside cells. **a** MICs of MCX-219 and MCX-190 against *S. aureus*, *M. pneumoniae*, *S. pyogenes*, *S. pneumoniae*, *E. faecium*, *E. faecalis*, *H. influenzae* and *M. catarrhalis*. **b** MIC_50_ and MIC_90_ of erythromycin (ERY), telithromycin (TEL), MCX-219, and MCX-190 against 23 clinical isolates of *S. aureus* from hospitals in China, 2021. **c** Cellular accumulation in *S. aureus* 15B196 for MCX-219, MCX-190, ERY, TEL and ciprofloxacin (CIP). Data are expressed as mean ± SD and analyzed by using one-way ANOVA plus Dunnett. Statistical significance is reported as follows: *P < 0.05, **P < 0.01, ***P < 0.001, ****P < 0.0001 vs ERY.

Strikingly, clinical isolates of *S. aureus*, irrespective of inducible or constitutive phenotypes, are susceptible to MCX-219 and MCX-190 (Fig. 2a). Furthermore, three mutant isolates of *M. pneumoniae* with mutations A2058G, A2059G, or A2058T are fully sensitive to MCX-219 and MCX-190. By contrast, telithromycin is inactive against both A2058-methylated *S. aureus* and A2058-mutated *M. pneumoniae*. Moreover, MCX-219 and MCX-190 demonstrate superior activity over telithromycin against clinical isolates of the other pathogens possessing various resistance types, such as *S. pneumoniae* and *S. pyogenes*, the majority of which might be the A2058-dimethylated as described by Douthwaite (Fig. 2a; Supplementary Table S9)^27^.

Regardless of the categorization of the extensive clinical isolates of *S. aureus* (whether categorized into MRSA and methicillin-susceptible *S. aureus* groups or alternatively into inducible and constitutive phenotype groups), notable reductions in MIC_50_ values were observed for MCX-219 and MCX-190, rather than telithromycin, against the isolates collected in 2021 from various regions of China, known for their high resistance to antibiotics (Fig. 2b). For example, MCX-219 and MCX-190 had an MIC_50_ of 1 μg/mL and 4 μg/mL against MRSA, respectively, while telithromycin had an MIC_50_ of 128 μg/mL. Meanwhile, MCX-219 and MCX-190 had an MIC_50_ of 2 μg/mL and 8 μg/mL against constitutive *S. aureus*, respectively, while telithromycin had an MIC_50_ of > 256 μg/mL, further corroborating the activity of MCX series against constitutively erm-modified isolates.

The high accumulation inside cells is an important issue for designing new antibiotics^46^. Unexpectedly, the enhanced activity of MCX-219 and MCX-190 against constitutively resistant *S. aureus* was partially contributed by their high cellular accumulation. It was found that MCX-219 and MCX-190 accumulated to a greater extent inside *S. aureus* cells with A2058-methylated ribosomes compared to telithromycin and erythromycin (Fig. 2c). MCX-219 accumulated twice as much as ciprofloxacin, although molecules with a considerably large molecular volume tend to exhibit low accumulation^46^. Meanwhile, the higher accumulation of MCX-219 over MCX-190 accounted for higher in vivo inhibition of cell growth of MCX-219 over MCX-190, although MCX-219 and MCX-190 showed equal in vitro inhibition on the target (Refer to the following text).

MCX-219 showed bactericidal activity against 58.3% of *S. aureus* isolates (MBC/MIC ≤ 4), while telithromycin and its analog solithromycin (suspended in clinical phase III) were bacteriostatic against all selected isolates (Supplementary Fig. S2a and Table S10). Further study on time-kill kinetics also suggested that MCX-219 was bactericidal against constitutively erm-modified *S. aureus* 15B196, but bacteriostatic against induciblely erm-modified *S. aureus* PU32 (Supplementary Fig. S2b). In contrast to telithromycin, MCX-219 exhibited a decreased frequency of inducible resistance against susceptible *S. aureus* ATCC29213 and induciblely erm-modified *S. aureus* PU32 at a concentration of 4× MIC. Moreover, at concentrations of 8× MIC or 16× MIC, MCX-219 showed no inducible resistance against constitutively erm-modified *S. aureus* 15B196 that is insensitive to telithromycin (Supplementary Table S11).

### Synthetic macrolides inhibit protein synthesis rather than DNA replication

The SARs of the synthetic hybrids, MCX-219 and MCX-190, have shown the essential role of the quinolone moiety in their antibacterial activity. Quinolone antibiotics target topoisomerase, a key enzyme involved in DNA supercoiling. DNA supercoiling is a crucial process in bacterial cells that involves the twisting and winding of the DNA double helix. Inhibition of DNA supercoiling can interfere with essential cellular functions, such as replication and transcription. While acknowledging the pivotal role of the quinolone moiety in activities, we want to determine whether these hybrids are topoisomerase inhibitors or protein synthesis inhibitors.

An in vivo dual-reporter assay was conducted in engineered *E. coli* containing pDualrep2 reporter, in which the expression of Katushka2S protein (shown in red color) is induced in the presence of translation elongation inhibitors, while the expression of red fluorescent protein (RFP) (shown in green pseudo-color) is activated when exposed to compounds that cause DNA damage^47^. Unlike ciprofloxacin, MCX-219 and MCX-190 displayed a red color inhibition zone, similar to the observation for erythromycin (Supplementary Fig. S3a).

In agreement with the results of the dual-reporter assay, these compounds showed in vitro minimal inhibition of *E. coli* DNA supercoiling, with IC_50_ values greater than 100 μM (Supplementary Fig. S3b). On the other hand, in vitro cell-free transcription/translation assays using materials from *E. coli* confirmed that MCX-219 and MCX-190 strongly inhibited protein synthesis with IC_50_ values of 1.19 μM and 0.67 μM, respectively (Supplementary Fig. S3c).

To discern the mechanism by which macrolones impede bacterial growth, whether by targeting the ribosome or DNA gyrase, we opted to select and characterize spontaneous resistant mutants. SQ110DTC is an engineered *E. coli* strain with a sole chromosomal rRNA operon, designed specifically for researching ribosome-targeting antibiotics^48^. When subjected to 3–9-fold MIC concentrations of MCX-219 and MCX-190, resistant colonies emerged. From these, twelve colonies (four for each concentration) were randomly chosen for sequencing to identify potential mutations in 23S rRNA and DNA gyrase. The resistance was found to stem from the mutations at A2058G or A2059G positions in the rRNA, with no mutations detected in the gyrase (Supplementary Fig. S4).

Collectively, the consistency in in vitro and in vivo assays suggests that the hybrids of macrolides and quinolones, exemplified by MCX-219 and MCX-190, exert their effects through function of protein synthesis inhibitors.

### Structural basis for the SARs of the MCX series

The pronounced efficacy of MCX-190 against constitutively erm-modified *S. aureus* prompted us to investigate the structural basis for the SARs of our compounds, particularly focusing on how MCX-190 interacts with the ribosome at the molecular level.

We first purified 70S and 50S ribosomes from a wild-type (WT) *S. aureus* strain and a clinical isolate, MRSA 15B196. Notably, the 15B196 strain, constitutively erm-modified, demonstrates complete resistance to macrolides such as telithromycin, azithromycin, clarithromycin, and erythromycin.

**Fig. 3:**
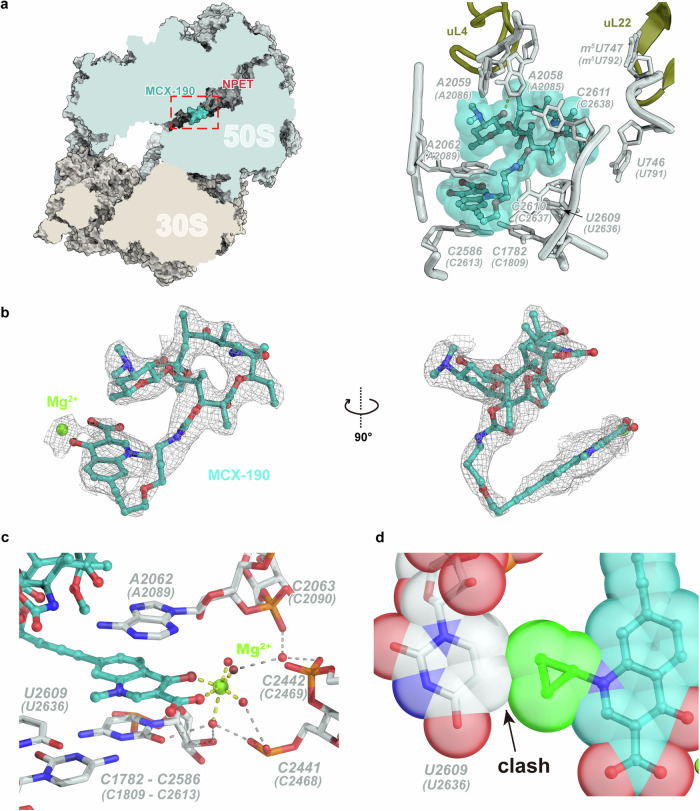
Cryo-EM reconstruction of the complex of MCX-190 and WT ribosomes of *S. aureus.* **a** The overall and detail of MCX-190 bound to the NPET. The distance of the hydrogen bond between the 2’-OH of desosamine and N1 positions of A2058 (A2085) is 2.93 Å. **b** The density map of MCX-190 and cation Mg^2+^ in the WT ribosome complex. **c** Cation Mg^2+^ is chelated by the quinolone element and the other four H_2_O molecules. **d** A cyclopropyl group at the N1 position of quinolone could create steric clashes with U2609 (U2636).

We next determined the atomic structures of MCX-190 when bound to the 70S or 50S ribosomes from both strains using cryo-electron microscopy (Cryo-EM) (Supplementary Figs. S5–S7). Each of the four complexes revealed clear densities for MCX-190, localized within the NPET of the large ribosomal subunit (Fig. 3a, b). The absence of additional density for MCX-190 in the 30S subunit indicates that the NPET is the exclusive binding site for MCX-190. Structurally, the macrolactone ring of MCX-190 aligns similarly to other macrolides like erythromycin, with desosamine engaging with A2058 (*S. aureus* A2085). Remarkably, the quinolone moiety, extending ~8 angstroms away from the macrolactone ring, stacks atop the base pair C1782–C2586 (C1809–C2613) with its flat plane. It has been documented that A2062 (A2089) acts as a gatekeeper within NPET, selectively allowing certain nascent peptides with specific N termini to bypass and release them^49^. The presence of the quinolone moiety causes a conformational shift of the gatekeeper A2062 (A2089), leading it to reposition next to the quinolone, unlike when ribosomes are bound to erythromycin or not bound to drugs (Supplementary Fig. S8). As a result, the quinolone becomes sandwiched between the A2062 (A2089) and the C1782–C2586 (C1809–C2613) base pair (Fig. 3c).

The revelation of the quinolone binding site sheds light on the SARs of the MCX series of antibiotics. MCX-190, featuring a linkage at the C-7 position of the quinolone, retains potent activity, whereas alterations to the C-6 position, seen in compounds 57a and 61a, lead to a dramatic decrease in activity against constitutively erm-modified isolates. In the MCX-190–ribosome structure, the sandwiched quinolone is further stabilized by a water–magnesium bridge connecting the C3/C4 keto oxygen of the quinolone to the rRNA backbone phosphate of C2063, C2441, C2442, and C2586 (C2090, C2468, C2469, and C2613) (Fig. 3c). This stabilized interaction, contingent on the correct orientation of the quinolone, is ensured by the C-7 linkage (57c and 61c**)**. This structural configuration is reminiscent of the one seen in the complex of ciprofloxacin with topoisomerase IV, where a similar water–magnesium bridge is crucial for the drug’s activity^45^. Such a bridge is potentially indispensable for MCX-190 targeting the ribosome. To further support this, 3-esterification (88h) or 3-amidation (61d) of the quinolone, known to significantly reduce the chelating ability of the carboxyl oxygen for metal ions, markedly deteriorates its activity.

Analysis of modifications at the N-1 position of the quinolone reveals that a methyl group confers the highest activity, while larger groups like ethyl and cyclopropyl result in a decrease in activity against constitutive *S. aureus*. Structurally, the quinolone of MCX-190 lies in the same plane as the adjacent base U2609 (U2636), with its methyl group oriented towards U2609 (U2636) at a distance of 4 angstroms. Our model suggests that bulkier chains, such as the ethyl group in compound 95f or a cyclopropyl group in 95c, could create steric clashes with U2609 (U2636), thus impeding activity (Fig. 3d). However, it would be intriguing to investigate the potential π–π interaction by incorporating a phenyl group, like the 2,4-difluorophenyl group, onto the N-1 position.

Finally, the linker connecting the quinolone and macrolactone ring plays a crucial role in determining the efficacy of the MCX series. The lead compounds, MCX-219 and MCX-190, though differing by one atom, share the same linker length. Variations resulting in either shorter (87c vs 88c) or longer linkers (88g vs 89g; 95g vs 96g) lead to decreased antibacterial efficacy. Structurally, in the MCX-190 complex, the linker adopts an L-shape, predominantly extended except at the turn. This conformation suggests a lack of structural tension, ensuring optimal alignment of the molecule. Furthermore, the linker packs against the C2610 (C2637) base, providing additional support for MCX-190 to bind to the ribosome (Fig. 3a). Our results suggest that the linker’s spatial arrangement is critical in maximizing the compound’s therapeutic potential.

### MCX-190 targets WT and A2058-methylated ribosomes of *S. aureus*

**Fig. 4:**
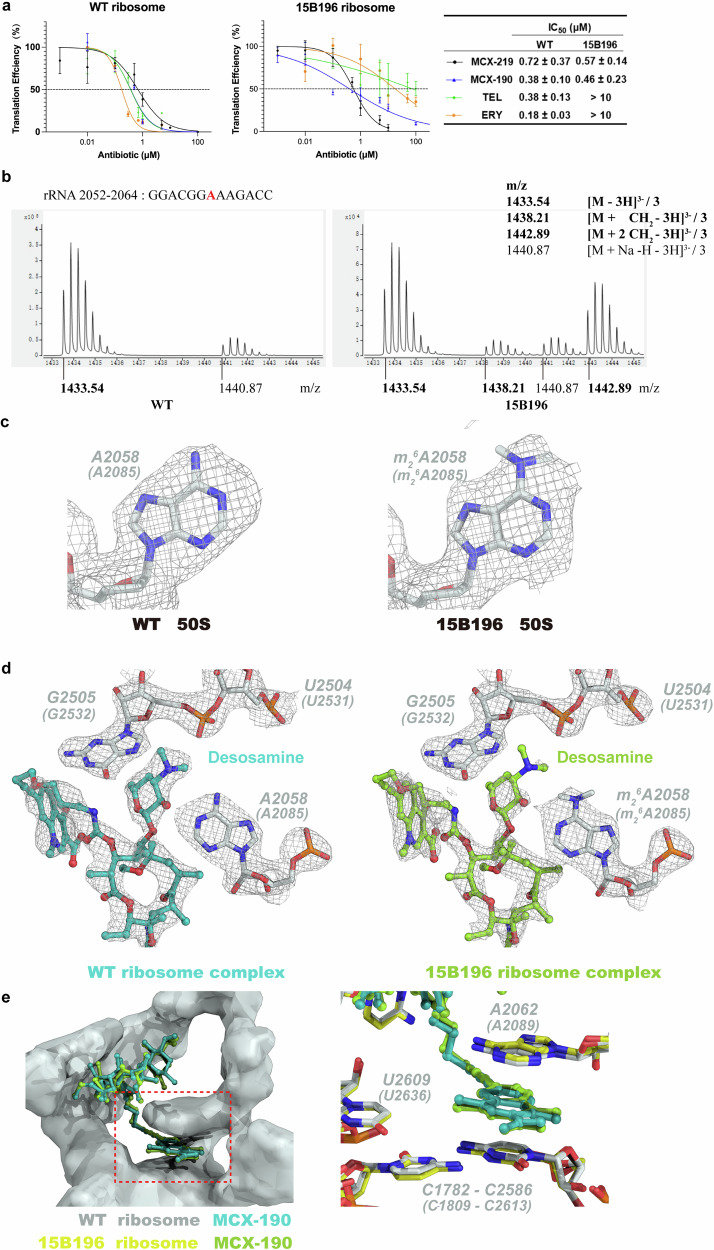
Comparison of MCX-190 and WT/15B196 *S. aureus* ribosome complexes. **a** The results of in vitro translation inhibition assays showed that 15B196 ribosomes were sensitive to MCX-219 and MCX-190. IC_50_ Values are shown as mean ± SD of at least three independent experiments. **b** Q-TOF mass spectrometry analysis of *S. aureus* rRNA 2052–2064. In the triply deprotonated ion of the molecule, the fragment GGACGGAAAGACC runs at 1433.54 m/z (when unmethylated). Monomethylation and dimethylation at A2058 result in mass shifts to 1438.21 m/z and 1442.89 m/z, respectively. The unmethylated fragment with a Na ion is observed at 1440.87 m/z. **c** The density maps of A2058 (A2085) in the 15B196/WT *S. aureus* ribosome complex. Both of two density maps of are contoured at 7.5σ. **d** The density of the desosamine in the complex of 15B196 ribosome complex was significantly decreased, compared to the WT ribosome complex. **e** The π–π interaction of quinolone ring with A2062 (A2089) and C1782–C2586 (C1809–C2613), was not affected by whether A2058 (A2085) was methylated.

To validate the in vitro activity of our compounds, we employed an in vitro reconstituted *E. coli* Protein synthesis Using a Recombinant Elements (PURE) translation system, substituting *E. coli* ribosomes with the purified *S. aureus* ribosomes. As depicted in Fig. 4a, erythromycin and telithromycin, along with MCX-219 and MCX-190, inhibited protein synthesis utilizing the WT *S. aureus* ribosomes. By contrast, the 15B196 ribosomes were sensitive to MCX-219 and MCX-190, mirroring the in vivo activity against erm-modified clinical isolates.

To assess the methylation status of A2058, we employed a modified method based on ref. ^50^ and designed two distinct DNA probes to isolate rRNA fragments spanning 2052–2064 and 2055–2066 from both WT and 15B196 ribosomes. Quadrupole time-of-flight (TOF) mass spectrometry (QTOF-MS, Agilent 6545) was used to analyze the methylation at A2058. This technique allows for highly accurate mass measurements of RNA oligonucleotides, within 1.0 Da, facilitating the detection of mono-methylation (addition of 14 Da) and di-methylation (addition of 28 Da).

In the triply deprotonated ion, the nucleotide A2058 is present in the fragment GGACGGAAAGACC with a mass-to-charge ratio (m/z) of 1433.54 when unmethylated as seen for the WT ribosome. In the 15B196 ribosomes, additional masses corresponding to mono-methylation (1438.21 m/z) and di-methylation (1442.89 m/z) were observed (Fig. 4b). A similar pattern is observed in the fragment CGGAAAGACCCC (Supplementary Fig. S9). By integrating the peak areas (Supplementary Fig. S9), we determined the proportions of mono-methylation in the 15B196 ribosome to be 5.7% and 6.3%, and the proportions of di-methylation to be 40.4% and 37.3%, respectively. This suggests that the main modification is di-methylation.

The MCX-190–50S complexes were resolved at resolutions of 2.8 Å and 2.5 Å for the WT and 15B196 *S. aureus* ribosomes, respectively, which allowed us to verify base modifications (Supplementary Fig. S10). A2058 methylation is crucial for MLS_B_K antibiotic resistance. When comparing the densities for A2058 in both 50S structures, we observed a peak emanating from the N6 of A2058 in the complex with the 15B196 ribosome (Fig. 4c), indicative of methylation. However, due to the low methylation level, the occupancy was expected to be low, which prevented us from clearly visualizing two methyl groups. Since the main modification is dimethylation, we modeled A2058 from the 15B196 ribosome as m_2_^6^A2058. In both complexes, the desosamine of MCX-190 interacted with the unmodified A2058 and the methylated A2058 base via hydrogen bonding. Importantly, the interaction of the latter was weakened as confirmed by the decreased density of the desosamine in the complex of A2058-methylated ribosome (Fig. 4d).

Historically, methylation of A2058 has been known to weaken the interaction with the desosamine in macrolides, leading to high-level resistance^1,7,8^. However, this weakened interaction can be compensated by introducing a secondary binding site. For instance, telithromycin, featuring a side-chain containing an aryl group that extends from the C-11 and C-12 positions, forms a stacking interaction with A752–U2609 (Supplementary Fig. S11)^22–24^. This additional binding confers resistance to telithromycin against A2058-modified ribosomes. A similar rationale may elucidate the mechanism of MCX-190’s activity: the quinolone ring establishes a new binding site, distanced from the mutation hotspot A2058 and thereby not affected by whether A2058 was methylated (Fig. 4e). Intriguingly, MCX-190 exhibits remarkable activity against *M. pneumoniae* clinical isolates harboring the A2058G mutation. Further investigations are warranted to elucidate how MCX-190 interacts with the A2058G-modified ribosome, potentially unveiling novel insights into its mode of action.

The rRNA base A2062, highly flexible, occludes the NPET lumen, and certain nascent peptides may cause it to reposition closer to the NPET wall, influencing peptide selective release^49,51^. In the presence of erythromycin or telithromycin, there is still ample space for the nascent peptide chain to pass through the NPET (Supplementary Fig. S12). By contrast, when MCX-190 is bound, the NPET channel is completely obstructed due to the A2062 base flip and the presence of the quinolone side chain. Thus, the developed macrolone demonstrated a novel mechanism of action, which contributes to its remarkable effectiveness against various pathogenic species, including constitutively resistant *S. aureus* strains, which were previously unresponsive to existing MLS_B_K treatments.

### MCX-190 and MCX-219 do not inhibit human ribosome

**Fig. 5:**
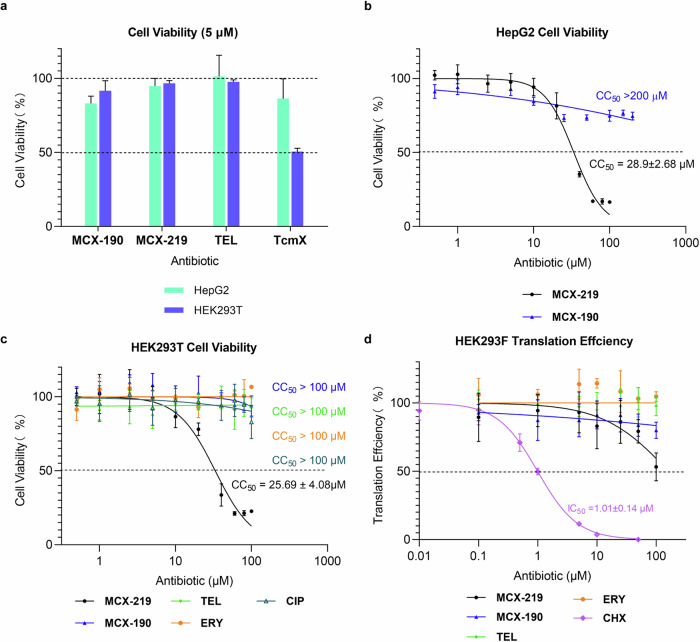
Cytotoxicity and inhibition of eukaryotic translation by MCX-219 and MCX-190. **a** Cytotoxicity of the optimal compounds at a concentration of 5 μM with an incubation for 48 h. **b**, **c** Cell viability of HepG2 (**b**) and HEK293T (**c**) cells in the presence of increasing concentrations of MCX-219 or MCX-190. MCX-219 showed a calculated cytotoxicity concentration at 26 μM that inhibits 50% viability (CC_50_), while TcmX reportedly had a CC_50_ of 2 μM (**c**). **d** In vitro translation of Fluc mRNA in HEK293F whole-cell lysate in increasing concentrations of MCX-219 or MCX-190. CHX cycloheximide. Values are shown as the mean ± SD of at least three independent experiments.

We noted that a tetracyclic antibiotic, tetracenomycin X (TcmX) with a similar scaffold to doxorubicin and tetracycline, interacts U1782–U2586 base pair in the *E. coli* ribosome^47^. Importantly, this report has verified that U1782–U2586, homologous C1782–C2586 in *S. aureus* and *S. pneumoniae*, is conserved across bacterial species. However, TcmX showed high cytotoxicity of ~2 μM and high inhibition on human ribosome at an IC_50_ of 10 μM, limiting its further development in antibacterial application^47^. Therefore, it is necessary to distinguish whether the cytotoxicity is caused by the structure of TcmX or its specific binding to the rRNA base pair C1782–C2586. The results from cytotoxicity assays in human HepG2 and HEK293T cells revealed that MCX-219 and MCX-190 demonstrated significantly reduced cytotoxicity compared to TcmX at a concentration of 5 μM (Fig. 5a). Furthermore, MCX-219 has a CC_50_ of ~26–29 μM, while MCX-190 is essentially non-cytotoxic (Fig. 5b, c). Meanwhile, the inhibition of protein synthesis in human ribosomes treated by MCX-219 and MCX-190 is measured with IC_50_ values of more than 100 μM (Fig. 5d). This comparison clearly indicated that the cytotoxicity of TcmX is basically related to its own structure.

## Conclusion

The MLS_B_K resistance associated with ribosomal alterations in clinical pathogens, particularly in relation to the primary target A2058, remains an unresolved problem. This has imposed constraints on the development of MLS_B_K antibiotics for an extended period. However, we have unveiled a novel and effective mode of action for the newly developed synthetic macrolides, such as their interaction with A2062 through a flipping mechanism and the subsequent sandwich-like A2062-quinolone-C1782/C2586 stacking mode of action within the NPET, the enhanced stabilization through a water–magnesium bridge. It is worth noting that currently available MLS_B_K antibiotics including macrolones have shown an inability to effectively treat constitutively A2058-methylated *S. aureus*, attributed to species-specific effects. However, cryo-EM complex structures in this work have indicated that the methylation status of A2058 does not impact the contact between the quinolone element of MCX-190 and the new binding site, thereby firmly inhibiting protein synthesis of A2058-methylated ribosomes of *S. aureus*. This finding opens the possibility of utilizing quinolones as novel pharmacophores with unprecedented modes of action within ribosomes. Meanwhile, the optimal macrolides exhibited favorable drug-like properties, including low cytotoxicity to human cells, low induction of bacterial resistance, high cellular accumulation in cells, and high blood drug concentration in vivo (Supplementary Table S12).

In conclusion, the comprehensive SARs, combined with biological analysis and structural insights, illuminate the innovative modes of action of these optimized chemotypes. Our research provides a promising basis for the rational design of the next-generation MLS_B_K to tackle prevalent resistance and expand the therapeutic reach to a broader spectrum of bacterial species.

## Materials and methods

### Susceptibility testing

MICs were determined by the microdilution method according to the Clinical and Laboratory Standards Institute guidelines. Briefly, the stock solution of compounds and the reference antibiotics were diluted 2-fold in sterile 96-well plates with cation-adjusted Mueller-Hinton broth, and mixed with CAMHB-containing bacteria to give a final inoculum of 5 × 10^5^ CFU/mL for broth microdilution experiments. The concentration range of the compounds typically employed for each experiment was 256–0.008 µg/mL (the final concentration of DMSO was kept below 1%). Finally, the 96-well plates were incubated at 37˚C for 16–20 h to observe and record the results. The lowest concentration at which no bacterial growth could be observed visually was identified as the MICs of the compounds. The assay for MICs was replicated at least three times.

### Determination of antibacterial mechanism with pDualrep2 reporter

The reporter strain JW5503 Δ*tolC* pDualrep2 has been utilized as described^47,52^. We applied tested antibiotic solutions (spots of ciprofloxacin (1.65 ng), erythromycin (1.28 μg), MCX-190 (1.28 μg), and MCX-219 (6.4 μg)) to an agar plate that already contained a reporter strain lawn. The plate was incubated overnight at 37 ˚C, and then we scanned it with ChemiScope 6100 (Clinx, CN) using the ‘Cy3-blot’ mode for RFP and ‘Cy5-blot’ mode for Katushka2S. Katushka2S expression induction is triggered by translation inhibitors, whereas RFP up-regulation is a consequence of DNA damage and SOS response induction.

### DNA supercoiling assay

The inhibition values of compounds on *E. coli* gyrase were determined using Gyrase Supercoiling Assay Kits purchased from Inspiralis (Norwich, UK). Supercoiling reaction mixtures, consisting of 35 mM Tris-HCl (pH 7.5), 24 mM KCl, 4 mM MgCl_2_, 2 mM DTT, 1.8 mM Spermidine, 1 mM ATP, 6.5% (w/v) glycerol, 0.1 mg/mL BSA, 1 U of DNA gyrase, 0.5 µg of relaxed pBR322 DNA and various concentrations of compounds (ciprofloxacin, MCX-219, MCX-190, 106g, 126g) were incubated at 37 ˚C for 30 min. Ciprofloxacin was employed as a gyrase inhibitor control. The addition of 30 µL Stop Buffer and 30 µL chloroform/iso-amyl alcohol (24/1) stopped each reaction. The DNA products were analyzed by electrophoresis through 1% agarose gels in TAE buffer. After being stained with 0.5 μg/mL ethidium bromide, the gels were photographed and quantified using ChemiScope 6100 (Clinx, China).

### Time-kill kinetics assay

To further determine the bactericidal activity of MCX-219, a time-kill assay was performed according to the method described previously^53^. The *S. aureus* cultured overnight was diluted 10^4^ times with CAMHB broth and incubated for 2 h. Different concentrations of compounds (1× MIC, 2× MIC, 4× MIC, 8× MIC) were added to the bacterial broth and placed in a shaker to continue the incubation for 24 h. The number of colonies was measured at 0 h, 1 h, 3 h, 6 h, 9 h and 24 h. A flask containing bacteria left unexposed to antibiotic was used as blank control, and telithromycin was used as the positive drug (comparator drug).

### Frequency of resistance

Luria-Bertani (LB) agar plates containing the compound at concentrations corresponding to 4× MIC, 8× MIC, and 16× MIC were prepared. Bacterial cultures of *S. aureus* strains ATCC 29213, PU32, and 15B196 were generated through overnight cultivation, and bacterial counts were determined by serial dilution and plating. 200 µL of bacterial inocula (containing ~1 × 10^7^, 1 × 10^8^, and 1 × 10^9^ CFU, respectively) were evenly spread over the media plates. The plates were then incubated at 37 ˚C for 72 h, and the appearance of the colony was regularly monitored throughout this period.

### Accumulation assay

Assays were conducted according to previous reports^46^. In these experiments, the strain *S. aureus* 15B196 was employed. 1 mL of an overnight culture of *S. aureus* 15B196 was diluted in 100 mL of fresh LB broth and grown at 37 ˚C with shaking to an optical density (OD_600_) of 0.55. After incubation, the cells were centrifuged to separate them from the supernatant, which was subsequently removed. Subsequently, the cells were washed once with 30 mL of phosphate-buffered saline before being resuspended. Each experimental tube was then filled with 200 μL of bacterial suspension and incubated with shaking for 5 min. Finally, the antibiotic (MCX-219, MCX-190, erythromycin, telithromycin, or ciprofloxacin) was added to reach a concentration of 50 μM, and the incubation was continued for an additional 10 min. Prior to initiating the experiment, tubes were prepared by adding 300 μL of silicone oil (9:1 AR20/Sigma high temperature, cooled to –78 ˚C). A 200 μL layer of bacterial suspension was carefully added over the silicone oil cushion in each tube, ensuring that the oil layer was completely thawed before subjecting the tubes to centrifugation at 13,000× *g* for 2 min at room temperature. Finally, both the supernatant and oil were thoroughly removed. The resulting pellets of *S. aureus* were resuspended in a 20 µL of buffer containing 2 mg/mL of lysostaphin. The suspension was then incubated at 37 ˚C for 30 min. After the incubation, 30 µL of H_2_O was added before freezing the mixture in liquid nitrogen. Three cycles of freezing/thawing were performed, with each cycle lasting 3 min in liquid nitrogen, followed by incubation in a water bath at 65 ˚C for 3 min. Subsequently, the tubes were centrifuged at 13,000× *g* for 2 min at room temperature, and the precipitate was washed with 100 μL of methanol. The mixture of supernatant was analyzed using Liquid Chromatography-Mass Spectrometry/Mass Spectrometry (LC-MS/MS). The quantification of drug concentration in the samples was quantified using the external standard method.

### In vitro *E. coli* transcription/translation assay

The in vitro transcription/translation assay for *E. coli* utilized materials from the *E. coli* S30 extract system for circular DNA (L1020, Promega, USA). This kit contains all the necessary components for efficient transcription and translation of a user-provided DNA template. To test the inhibition of protein synthesis, the assay uses plasmid pBESTluc, which contains the firefly luciferase gene, as the DNA template. The intensity of luminescence resulting from luciferase expression corresponds to the efficiency of protein synthesis. The luminescence intensity was quantified in the presence or absence of the tested compounds. The compounds were evaluated at concentrations of 5 μM, 1 μM, 0.5 μM, 0.1 μM, and 0.01 μM to determine IC_50_ values. This experiment was repeated twice independently.

### Selection of resistant mutants in SQ110DTC

The *E. coli* strain SQ110DTC, which carries a single *rrnE* allele and Δ*tolC* mutation, was cultured overnight in LB medium supplemented with kanamycin (50 µg/mL). Subsequently, the cells were plated on LB agar plates containing kanamycin (50 µg/mL) along with various concentrations of the compounds, specifically around 3×, 6× or 9× MIC. After incubation at 37 °C for 48 h, ~20 colonies were observed on plates. 23S rRNA gene was then amplified using PCR and sequenced using the primers as previously described^48^. The *GyrA* gene was amplified using the primers GCGATGTCCGGTCATTGTT and ACTTCCGGTCAGGTTGTGC.

### Animal studies

All experimental processes were conducted in strict compliance with the experimental animal management regulations and animal ethics policies of Beijing Institute of Technology. Pathogen-free male SD rats weighing 200× *g* were obtained from Peking University Health Science Center. Animals were acclimatized for at least 3 days before the start of the studies and were exposed to a 12-h light/dark cycle. Animals were kept under controlled conditions with food and water ad libitum.

### In vivo pharmacokinetic study

The SD rats were fasted overnight and dosed i.v. at a dose of 5 mg/kg in a solution of DMSO/PEG 400/water (5%/24%/71%). The whole blood was collected into tubes containing sodium heparin at 0.083 h, 0.25 h, 0.5 h, 1 h, 2 h, 4 h, 8 h and 24 h after dosing. After centrifugation of 6000 rpm for 10 min at 4 ˚C, plasma samples were transferred into 1.5 mL tubes and stored at –20 ˚C. Prior to analysis, the samples were thawed on ice. An amount of 50 μL of each plasma sample was added to a mixture of 50 μL of propranolol (300 ng/mL) and 100 μL of acetonitrile and centrifuged at 12,000 rpm at 4 ˚C for 10 min. The supernatant was diluted and analyzed by LC-MS/MS. The liquid chromatography was conducted on a Thermo Scientific Q Exactive HF-X (ThermoFisher Scientific, USA) instrument, employing an ACQUITY UPLC BEH C18 Column with dimensions of 130 Å, 1.7 µm, 2.1 mm × 100 mm. The liquid phase method employed gradient elution with mobile phases consisting of acetonitrile and an 0.1% aqueous formic acid solution. Mass spectrometry was conducted in positive ion mode. The quantification of drug concentration in plasma samples was performed using the internal standard method, and the obtained pharmacokinetic parameters were calculated using Phoenix WinNonlin software.

### Cell culture

HEK293T (3101HUMGNHu17) and HepG2 (1101HUM-PUMC000035) cell lines were obtained from the Cell Resource Center, Peking Union Medical College. All were acquired in 2023 and were maintained in culture for no more than 30 continuous passages. The cells were cultured in DMEM (11995065, Gibco) supplemented with 10% FBS (164210-50, Procell), 100 units penicillin and 100 mg/mL streptomycin (PB180120, Procell).

### CCK-8 assay for cytotoxicity

HepG2 cells (6 × 10^3^) and HEK293T cells (4 × 10^3^) were seeded into 96-well plates in 90 μL of DMEM medium containing 10% FBS per well. All test compounds were formulated as 10 mM stock solutions in DMSO (final concentration ≤ 0.5%). After overnight incubation, the test compound stock solution was diluted with DMEM medium containing 10% FBS to every target concentration, and 10 μL of each was added to each well. Three parallel experiments were set up for each compound. After incubation for 48 h, 10 μL of cell counting kit-8 (CCK-8) reagent was added to each well in sequence according to the manufacturer’s instructions. After incubating in a 37 ˚C incubator for 1–2 h, the absorbance was measured at a wavelength of 450 nm, and detected by ThermoScientific Multiskan FC (ThermoFisher Scientific, USA). This experiment was independently repeated three times. The data were processed by GraphPad Prism.

### Ribosome purification

Ribosomes were purified from *S. aureus* WT/15B196 as previously described^54^, with minor modifications. The cells were lysed in buffer A (20 mM HEPES-KOH pH 7.5, 100 mM NH_4_Cl, 10.5 mM Mg-acetate, 0.5 mM EDTA-Na pH 8.0, 4 mM DTT, 150 mM sucrose) and the lysate was clarified by centrifugation. Following ultracentrifugation for 20 h (43,000 rpm) at 4 °C in the Type 45Ti rotor (Beckman Coulter, USA), the ribosome pellets were resuspended in the buffer B (10 mM HEPES-KOH pH 7.5, 50 mM KCl, 10 mM NH_4_Cl, 1.25 mM Mg-acetate, 0.25 mM EDTA-Na pH 8.0, 4 mM DTT). Subsequently, the ribosomes were separated by centrifugation through 15%–40% sucrose gradients in the SW32 rotor at 28,000 rpm for 16 h. 50S and 30S ribosomes were separated by ÄKTA pure™ chromatography system (cytiva), and buffer exchanged into 5 mM HEPES-KOH pH 7.5, 60 mM NH_4_Cl, 10 mM MgCl_2_, 4 mM DTT, flash-frozen in liquid nitrogen and stored at –80 °C.

### Sample preparation

MCX-190–WT ribosome complexes were prepared by incubating 0.2 µM 30S, 0.3 μM 50S in 1× ribosome buffer (20 mM Tris-HCl pH 7.5, 100 mM KCl, 10 mM MgCl_2_, 2 mM DTT) at 37 °C for 10 min. Then MCX-190 to a final concentration of 5 µM was added and incubated for another 10 min. Finally, the complexes were transferred on ice for cryo-grid preparation. MCX-190–15B196 ribosome complexes were prepared using the same procedure.

### Cryo-grid preparation, data collection, and processing

To prepare cryo-EM grids, 2.5 µL of the sample was applied onto the R1.2/1.3 300 mesh holey carbon Au grids with graphene oxide (Quantifoil) and left absorption for 8 s in the chamber under 100% humidity at 4˚C. The grids were blotted for 2–4 s in a Vitrobot Mark IV (ThermoFisher Scientific, USA) and plunge-frozen in liquid ethane at liquid nitrogen temperature. The ø 55/20 mm blotting paper was used for plunge freezing (TED PELLA).

All the cryo-EM images were automatically recorded on a Titan Krios (ThermoFisher Scientific) transmission electron microscope by a post-GIF Gatan K3 direct electron detector in the super-resolution mode using EPU software (version 2.8.2.10REL) with a nominal magnification of 81,000× in the ETTEM mode, which yielded a super-resolution pixel size of 0.412 Å on the image plane, and with a defocus ranged from 0.5 to 1.5 μm. Each micrograph stack was dose-fractionated into 40 frames with a total electron dose of ~50 e^–^/ Å^2^. 10,537 micrographs out of 11,326 movies for wide type-ribosome and 11,959 micrographs out of 12,426 movies for 15B196-ribosome were selected for further processing.

For cryo-EM data processing, drift and beam-induced motion correction were applied on the super-resolution movie stacks using MotionCor2^55^ and binned 2-fold to a calibrated pixel size of 0.824 Å/pix. The defocus values were estimated by CTFFIND4^56^ from summed images without dose weighting. As for 15B196-ribosome data, an initial set of 1,762,848 particles was used for 2D classification in RELION^57^. 1,644,372 particles were selected from useful 2D classes for the initial 3D classification, using a 60 Å low-pass filtered ab-initial model reconstructed in cryoSPARC^58^. For the 50S-complex, 68,392 particles were selected and refined to 2.53 Å resolution by NU-refinement^59^. For the 70S-complex, 47,560 particles were used and reconstituted to 2.58 Å resolution. To refine the MCX-190 structure, a masked local refinement followed by a masked local 3D classification focused on the NPET in RELION. As for WT ribosome data, an initial set of 1,207,936 particles was used for 2D classification in RELION^57^. 976,215 particles were selected from useful 2D classes for the initial 3D classification, using a 60 Å low-pass filtered ab-initial model reconstructed in cryoSPARC^58^. For the 50S-complex, 666,754 particles were selected and refined to 2.65 Å resolution by NU-refinement^59^. For the 70S-complex, 27,177 particles were used and reconstituted to 3.60 Å resolution. To refine the MCX-190 structure, a masked local refinement followed by a masked local 3D classification focused on the NPET in RELION.

We used the *S. aureus* 50S ribosome structure (PDB ID: 6s0z) and *S. aureus* 70S ribosome structure (PDB ID: 6s13) as initial models for model building. Firstly, a ten-cycle rigid body refinement was carried out for the map-based model fitting. Then, the models undergo a combined manual refinement using coot and real space refinement using Phenix. Overfitting the model was monitored by refining the model in one of the two half maps from the gold-standard refinement approach and testing the refined model against the other map. Statistics of map reconstruction and model refinement are listed in Supplementary Table S13. The final models were evaluated using MolProbity^60^. Map and model representations in the figures and movie were prepared by PyMOL (https://pymol.org/), UCSF Chimera^61^, or UCSF ChimeraX^62^.

### Methylation level determination at 23S rRNA nucleotide A2058

23S rRNA was extracted from WT/15B196 ribosome with TRIzol Plus RNA Purification Kit (ThermoFisher Scientific).

Two DNA probes (dGdGdGdGdTdCmUmUmUmCmCmGmUmCmCdTdGdTdCdGdC and dCdCdAdCdGdGmGmGmUmCmUmUmUmCmCdGdTdCdCdTdG) are complementary to 23S rRNA fragment G2046–C2066 and C2050–G2070 (*E. coli* numbering), respectively, with the 5′ and 3′ ends consisting of 6 nucleotides of DNA, while the remaining portion is 2′-*O*-methylated RNA. The DNA probe is labeled with biotin at the 3′ end for purification. We employed a modified method based on ref. ^50^, 100 pmoles of 23S rRNA was incubated with 500 pmoles of DNA probe in 180 μL of hybridization buffer (250 mM HEPES, 500 mM KCl at pH 7). After annealing procedure, 180 μL sample was incubated with 36 μL RNase H (180 U, from Apexbio), 24 μL 10× RNase H buffer (500 mM Tris-HCl pH 8.3, 750 mM KCl, 30 mM MgCl_2_, 100 mM DTT) at 37 °C for 30 min.

After RNase H digestion, the target rRNA fragment was purified with streptavidin magnetic beads (Pierce™, ThermoFisher Scientific). rRNA fragment was analyzed by Q-TOF mass spectrometry (6545XT AdvanceBio, Agilent). Data were analyzed by MassHunter Workstation 10.0.

### Prokaryotic cell-free systems and in vitro translation inhibition assays

A homemade *E. coli* in vitro TnT PURE system was used to assess the inhibitory effects of MCX-190, MCX-219, erythromycin, and telithromycin on 70 S ribosome activity^63,64^. 5 μL of PURE reaction mix that translates a gene encoding the Nanoluc luciferase was set up in the presence or absence of tested compounds. Protein synthesis was initiated by incubating reactions at 37 ˚C for 60 min and stopped by placing on ice for 5 min. Luciferase activity was quantified using the Nano-Glo luciferase assay system and read on a GloMax 20/20 luminometer (Promega, USA). For each compound, the in vitro translation inhibition assay was performed in three biological triplicates over a range of concentrations from 0.001 μM to 1000 μM. A compound-free control was used to normalize luminescence measurements. The calculated luminescence percentages were plotted against compound concentrations on a semilog scale using GraphPad Prism.

### Mammalian cell-free systems and in vitro translation assays

For the in vitro translation assay, the whole-cell extract was derived from cultured HEK293F cells^65^. The translation reactions were conducted in a total volume of 10 μL, comprising 4 μL of the HEK293F extract, 1 μL 10× Translation Buffer containing 160 mM HEPESs-KOH (pH 7.6), 100 mM Creatine phosphate, 1 mM Spermidine, 10 mM ATP, 1 mM GTP, 20 mM DTT, 800 ng/μL CKM, 600 mM KOAc, 30 mM Mg(OAc)_2_ and 200 μM Amino acids. The reaction mixture also included 2 U of RiboLock RNase inhibitor (ThermoFisher Scientific), 0.5 mM D-luciferin (Promega), 1 μL of either antibiotic solution or solvent (water or 10% DMSO), as specified, and 100 ng mRNA (added as a 1 μL water solution after pre-incubation of the reaction mixture with antibiotic for 5 min at 30 ˚C). Following the addition of mRNA, the mixtures were transferred to a pre-heated 96-well CELL CULTER PLATE (WHB), sealed with a PCR plate seal, and incubated in the CLARIOstar plate reader (BMG Labtech) at 37 ˚C with continuous luciferase activity measurement, following established procedures^66,67^. Luciferase activity values were determined based on light intensities at 60 min.

### Supplementary information

Supplementary Information

## Data Availability

The atomic coordinates were deposited in the RCSB Protein Data Bank (PDB) under accession codes 8Y36 (WT 50S in complex with MCX-190), 8Y37 (15B196 50S in complex with MCX-190), 8Y38 (15B196 70S in complex with MCX-190), and 8Y39 (WT 70S in complex with MCX-190). The cryo-EM maps have been deposited in the Electron Microscopy Data Bank (EMDB) under accession codes EMD-38873 (WT 50S in complex with MCX-190, from focused refinement), EMD-38874 (15B196 50S in complex with MCX-190, from focused refinement), EMD-38875 (15B196 70S in complex with MCX-190, from focused refinement), and EMD-38876 (WT 70S in complex with MCX-190, from focused refinement).
