# Supplementary material for: Synthetic macrolides overcoming MLSBK-resistant pathogens
Source: Cell Discov. 2024 Jul 11;10:75. doi: 10.1038/s41421-024-00702-y (PMC11239830; doi:10.1038/s41421-024-00702-y)
Supplement: Supplementary file 1 — Supplementary Information [file 41421_2024_702_MOESM1_ESM.pdf]

Supplementary Information for

Synthetic macrolides overcoming MLSBK-resistant pathogens

Cong-Xuan Ma[a,#], Ye Li[b, c,#], Wen-Tian Liu[a], Yun Li[d], Fei Zhao[e], Xiao-Tian Lian[a], Jing Ding[a], Si-Meng Liu[a], Xie-Peng Liu[a], Bing-Zhi Fan[a], Li-Yong Liu[e], Feng Xue[d], Jian Li[b, c], Jue-Ru Zhang[a], Zhao Xue, [b,c] Xiao-Tong Pei [b,c] Jin-Zhong Lin[b, c]*, Jian-Hua Liang[a]*

[a] Key Laboratory of Medicinal Molecule Science and Pharmaceutical Engineering, School of Chemistry and Chemical Engineering, Beijing Institute of Technology, Beijing, 102488, China

[b] State Key Laboratory of Genetic Engineering, School of Life Sciences, Zhongshan Hospital, Fudan University, Shanghai 200438, China,

[c] Center for mRNA Translational Research, Fudan University, Shanghai 200438, China

[d] Institute of Clinical Pharmacology, Peking University First Hospital, Beijing 100034, China

[e] National Institute for Communicable Disease Control and Prevention, Chinese Center for Disease Control and Prevention, State Key Laboratory of Infectious Disease Prevention and Control, Beijing 102206, China

# These authors contributed equally to this work.

* To whom correspondence should be addressed: E-mail: ljhbit@bit.edu.cn (J.-H.L.); linjinzhong@fudan.edu.cn (J.-Z.L.)

Table of Contents

Supplementary Fig. S1. Structure-activity relationships of the novel synthetic macrolides. 3

Supplementary Fig. S2. MBC/MIC and Time-kill kinetics data of **MCX-219** against *S. aureus*. 4

Supplementary Fig. S3. Modes of action of **MCX-219** and **MCX-190**. 5

Supplementary Fig. S4. Identification of cellular targets of compounds by selecting resistant mutants. 6

Supplementary Figs S5-S7. Flow chart of the cryo-EM image processing, data processing procedure and validation. 7

Supplementary Fig. S8. The conformation change of A2062 (A2089 in *S. aureus*) 11

Supplementary Fig. S9. Q-TOF mass spectrometry analysis of *S. aureus* rRNA. 12

Supplementary Fig. S10. Cryo-EM density map of modified rRNA. 13

Supplementary Fig. S11. Comparison of **MCX-190** and telithromycin. 14

Supplementary Fig. S12. The conformation of NPET when ERY/**MCX-190**/TEL is bound. 15

Supplementary Table S1-S8. MICs (μg/mL) of the novel synthetic macrolides. 16

Supplementary Table S9. In vitro antibacterial activity of **MCX-219** and **MCX-190**. 24

Supplementary Table S10. MICs and MBCs (μg/mL) for **MCX-219**, telithromycin and solithromycin. 25

Supplementary Table S11. Frequency of inducible resistance of S. aureus treated by **MCX-219** or telithromycin. 26

Supplementary Table S12. *In vivo* PK parameters of **MCX-219** and **88h** determined in male SD rats. 27

Supplementary Table S13. Refinement and model statistics for 50S-complex and 70S-complex. 28

General Procedures for Chemical Synthesis, Materials, and Instrumentation 30

Synthesis of compounds **4-6** 30

Synthesis of compounds **10-12** 31

Synthesis of compounds **14-15** 32

Synthesis of compound **17** 33

Synthesis of compounds **25-31**, **34** and **37-38** 34

Synthesis of compounds **47-50** 36

Synthesis of compounds **57-64** 37

Synthesis of compounds **70-72** and **74** 45

Synthesis of compounds **79** 48

Synthesis of compounds **87-90** 50

Synthesis of compounds **95-96** and **98** 57

Synthesis of compounds **107-111** 62

synthesis of compounds **116-117** 66

Synthesis of compounds **126-131** 69

Synthesis of compounds **137** and **141-143** 75

Synthesis of compounds **149** 79

Synthesis of compounds **152** 81

Spectra of compounds 83

Supplementary Fig. S1. Structure-activity relationships of the novel synthetic macrolides.

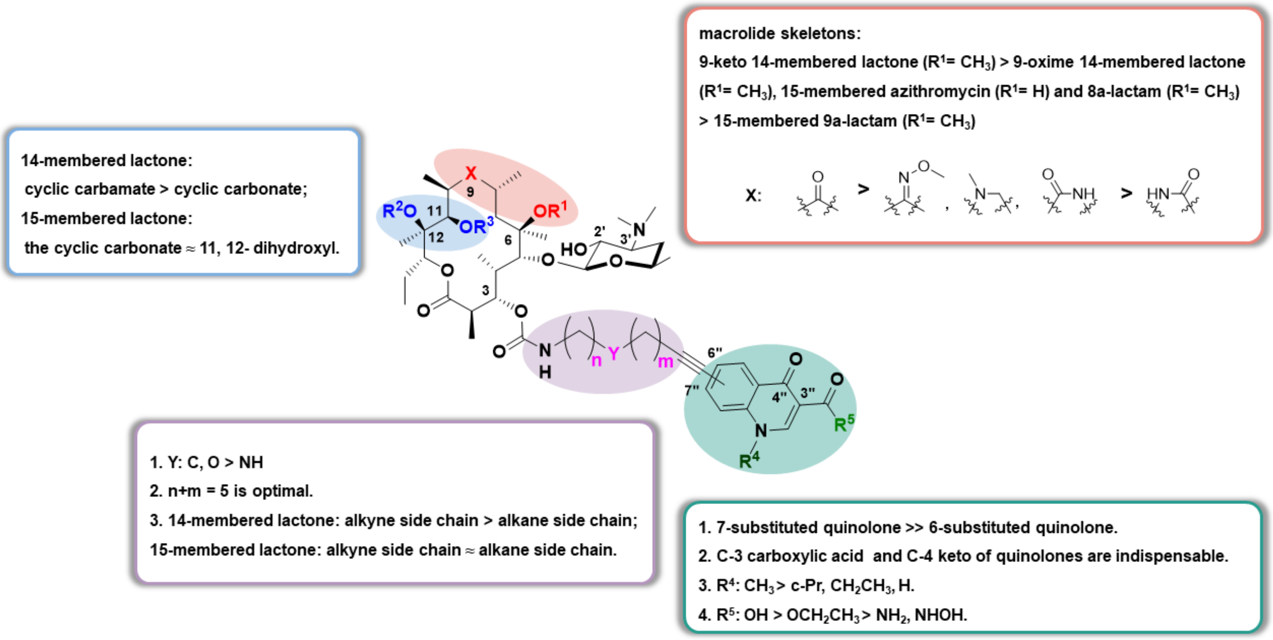

Supplementary Fig. S1 | Structure-activity relationships of the novel synthetic macrolides.

Supplementary Fig. S2. MBC/MIC and Time-kill kinetics data of MCX-219 against *S. aureus*.

a

	Compd.	number of strains with various MBC/MIC ratios: 1	2	4	8	>8	Cidal ratios ≤4
S. aureus (12)	**MCX-219**	0	1	6	3	2	58.3%
	telithromycin	0	0	0	0	12	0.0%
	solithromycin	0	0	0	2	10	0.0%

b

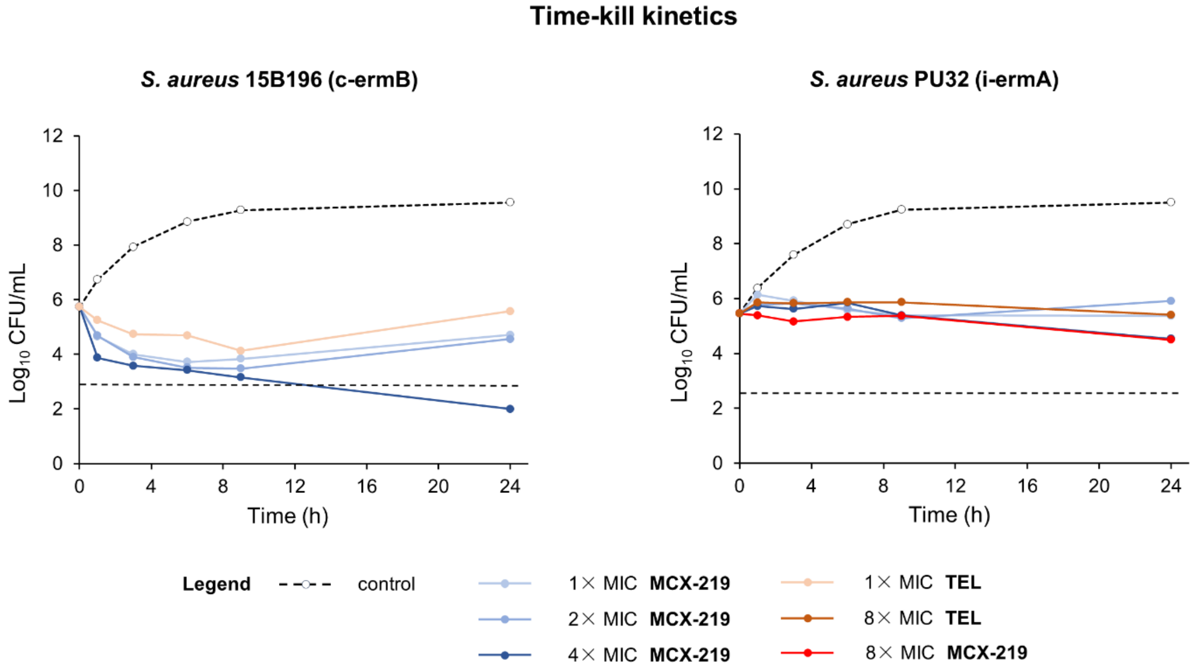

Supplementary Fig. S2 | MBC/MIC and Time-kill kinetics data of MCX-219 against *S. aureus*. a. MBC/MIC ratios for **MCX-219**, telithromycin and solithromycin against selected clinical isolates of *S. aureus*. **b**. Time-kill kinetics of **MCX-219** against erythromycin-resistant *S. aureus* encoded erm genes.

Supplementary Fig. S3. Modes of action of MCX-219 and MCX-190.

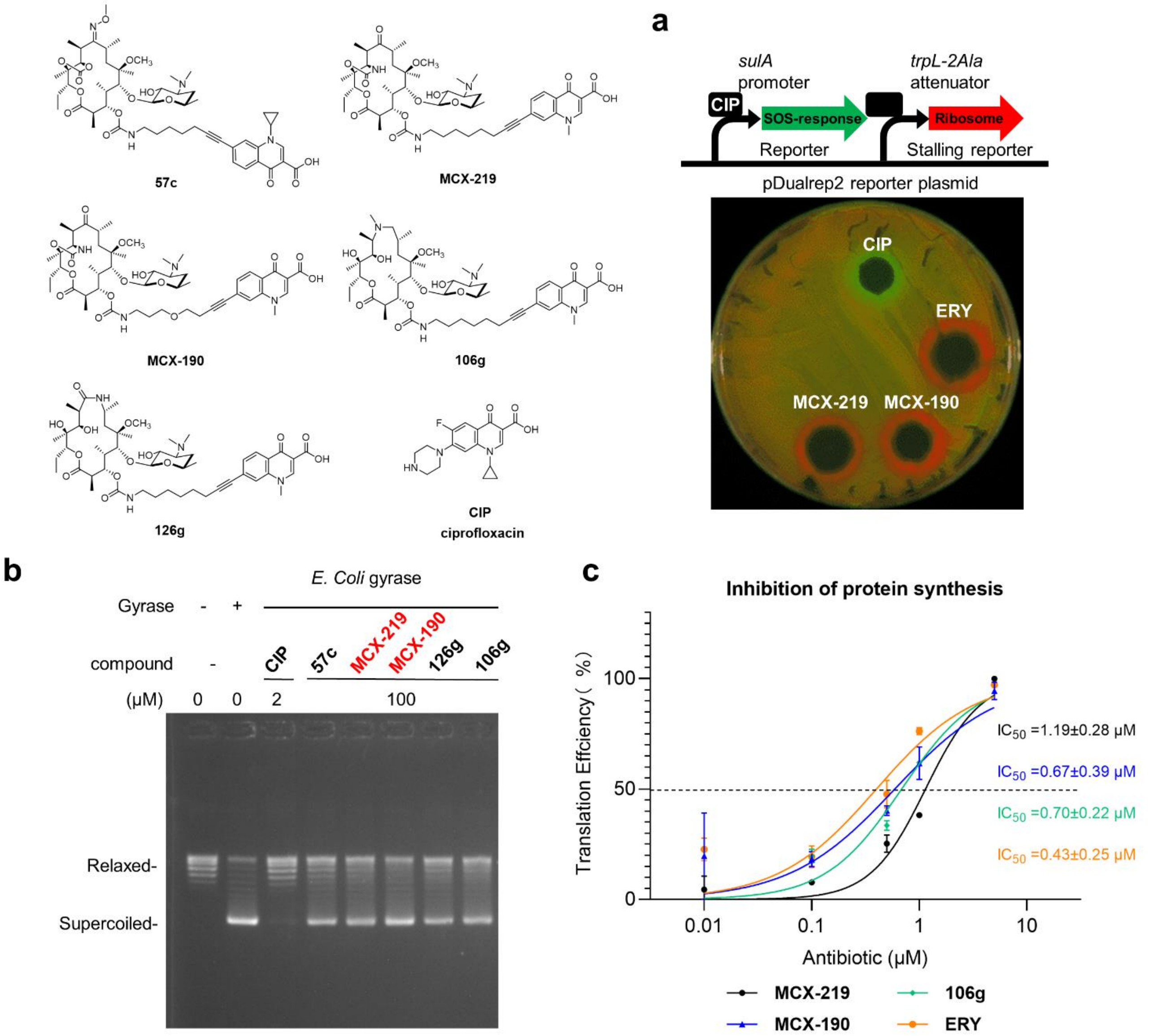

Supplementary Fig. S3 | Modes of action of MCX-219 and MCX-190. a. In vivo induction of a two-color dual-reporter system sensitive to inhibitors of ribosome progression or of DNA replication. Spots of erythromycin (**ERY**, 1.28 μg), ciprofloxacin (**CIP**, 1.65 ng), **MCX-219** (6.4 μg) and **MCX-190** (1.28 μg) were placed on the surface of an agar plate containing *E. coli* Δ*tolC* cells transformed with the pDualrep2 plasmid. Induction of the expression of Katushka2S (red) is triggered by translation inhibitors, whereas RFP expression (green) is induced on DNA damage. **b**, In vitro inhibition of the *E. coli* DNA supercoiling assay. **c**, In vitro inhibition of transcription and translation assay.

Supplementary Fig. S4. Identification of cellular targets of compounds by selecting resistant mutants.

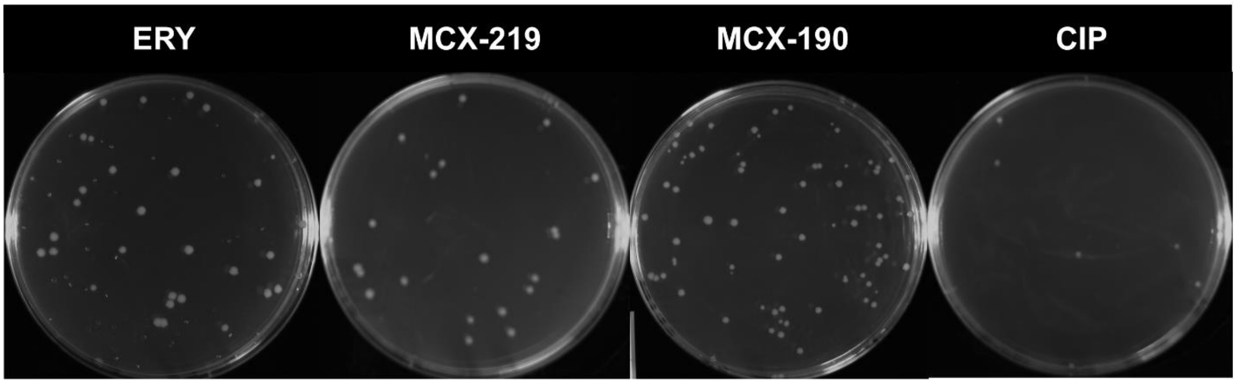

ERY:

Target	Mutation
23S rRNA	A2058G
23S rRNA	A2059G
23S rRNA	A2058C

MCX-219:

Target	Mutation
23S rRNA	A2058G
23S rRNA	A2059G

MCX-190:

Target	Mutation
23S rRNA	A2058G
23S rRNA	A2059G

CIP:

Target	Mutation
GyrA	Asp87Gly
GyrA	Ser83Leu
GyrA	Ala179Val
GyrA	Asp87Tyr

Supplementary Fig. S4 | Identification of cellular targets of compounds by selecting resistant mutants. Selection of the drug-resistant mutants of the antibiotic-hypersusceptible *E. coli* strain SQ110DTC on LB-agar plates with 3-9x MIC of antibiotics. The mutations identified in the sequenced resistant clones are shown underneath the plates. (**ERY**: erythromycin, **CIP**: ciprofloxacin)

Supplementary Figs S5-S7. Flow chart of the cryo-EM image processing, data processing procedure and validation.

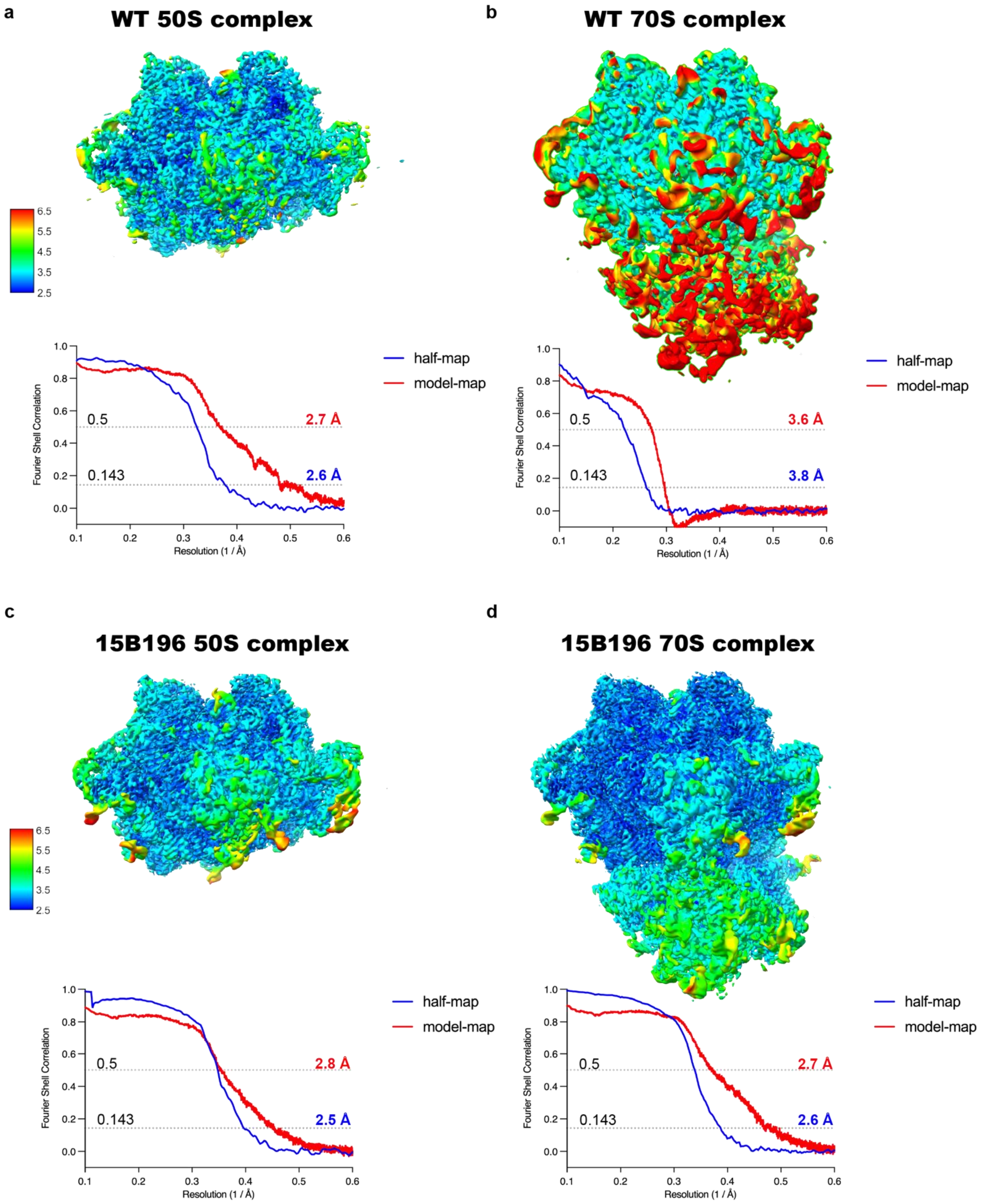

Supplementary Fig. S5 | Local resolution estimation and Fourier Shell Correlation (FSC) validation. a, Local resolution heat maps of WT 50S complex shown in the range of 2.5 – 6.5 Å resolution, calculated with cryoSPARC 4.3.0 implementation of BlocRes.[1] Gold-standard FSC curves of each half-map (blue), using a 'soft mask' excluding solvent and model-map (red), are plotted across resolution.

Map and model validation was performed in PHENIX 1.19.2.[2] **b**, Local resolution heat maps, gold-standard FSC curves of each half-map, and FSC of map-model validation of WT 70S complex. **c**, Local resolution heat maps, gold-standard FSC curves of each half-map, and FSC of map-model validation of 15B196 50S complex. **d**, Local resolution heat maps, gold-standard FSC curves of each half-map, and FSC of map-model validation of 15B196 70S complex.

References:

1. Cardone G. et al. One number does not fit all: mapping local variations in resolution in cryo-EM reconstructions. *J Struct Biol*, **184**, 226-236 (2013).
2. Afonine P V. et al. New tools for the analysis and validation of cryo-EM maps and atomic models. *Acta Cryst D*. **74**, 814-840 (2018)

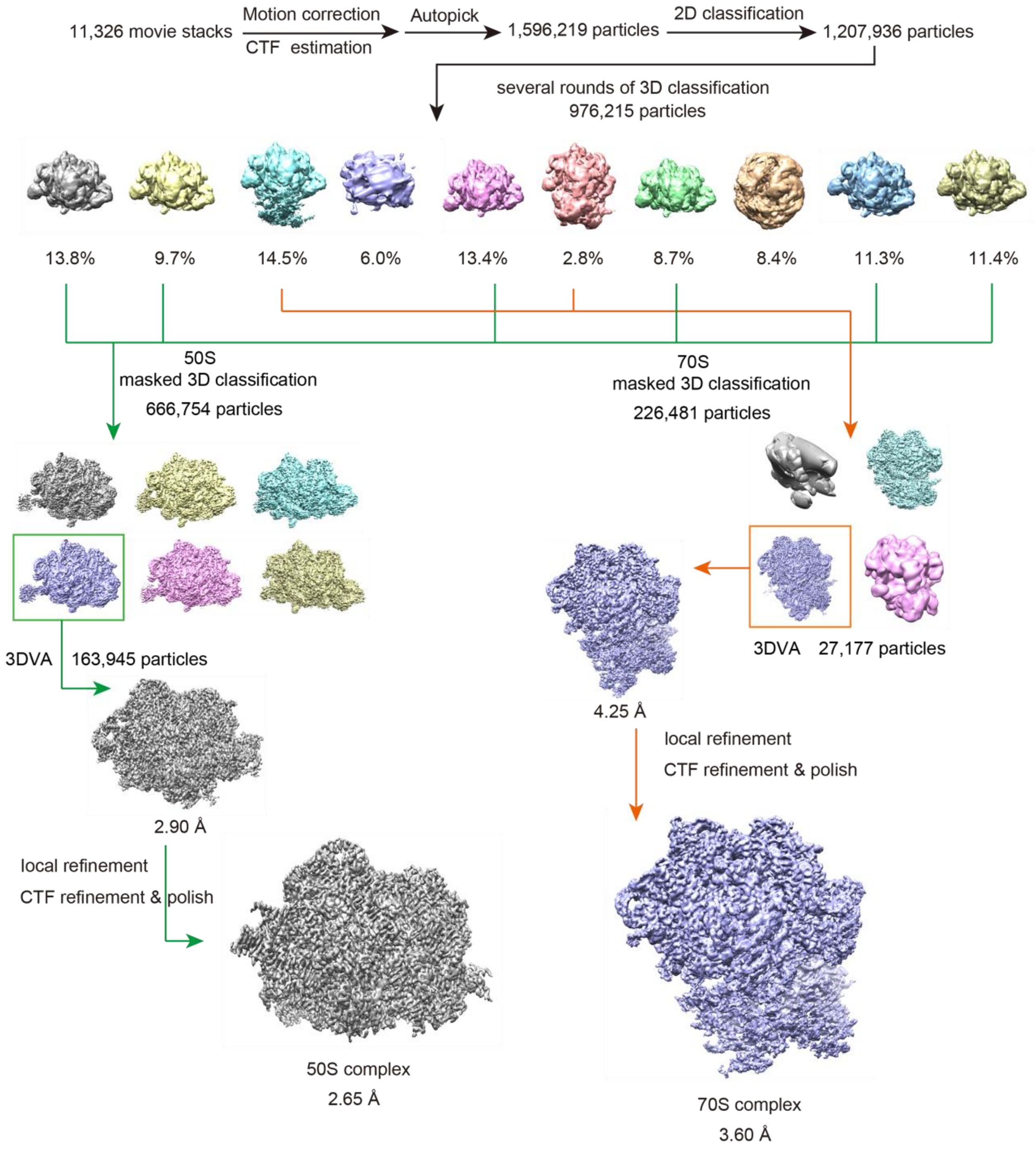

Supplementary Fig. S6 | Flow chart of WT-ribosome data processing procedure.

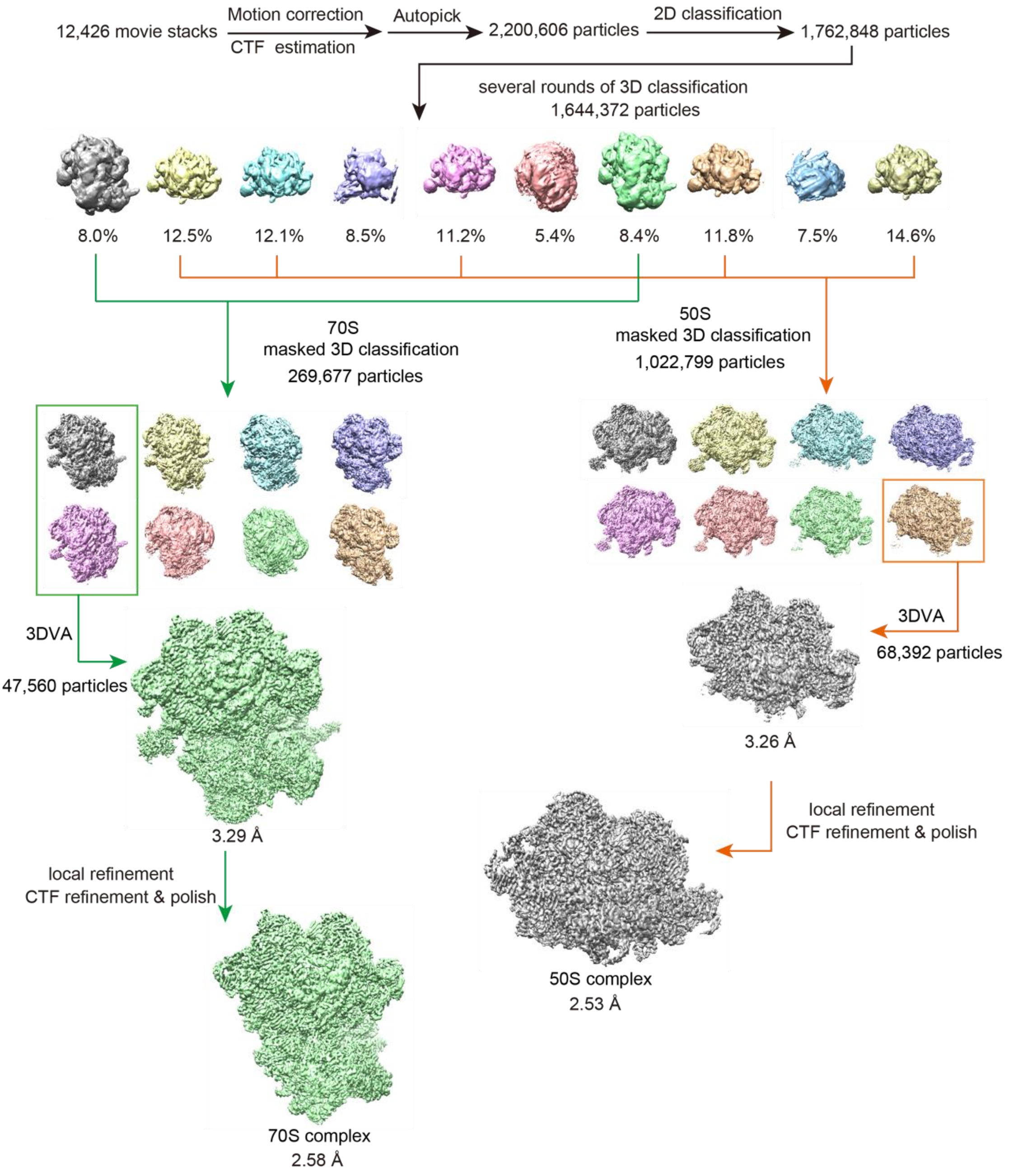

Supplementary Fig. S7 | Flow chart of Methylation-ribosome data processing procedure.

Supplementary Fig. S8. The conformation change of A2062 (A2089 in *S. aureus*)

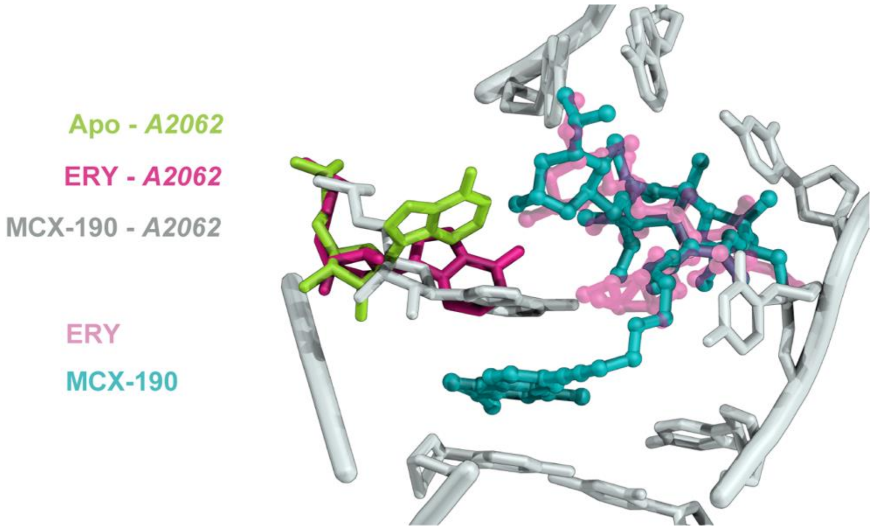

Supplementary Fig. S8 The conformation change of A2062 (A2089 in *S. aureus*) from apo to Ery-bound to MCX-190 bound complex. ERY: 6s0z (*S. aureus*). **Apo**: 6s12 (*S. aureus*).

Supplementary Fig. S9. Q-TOF mass spectrometry analysis of *S. aureus* rRNA.

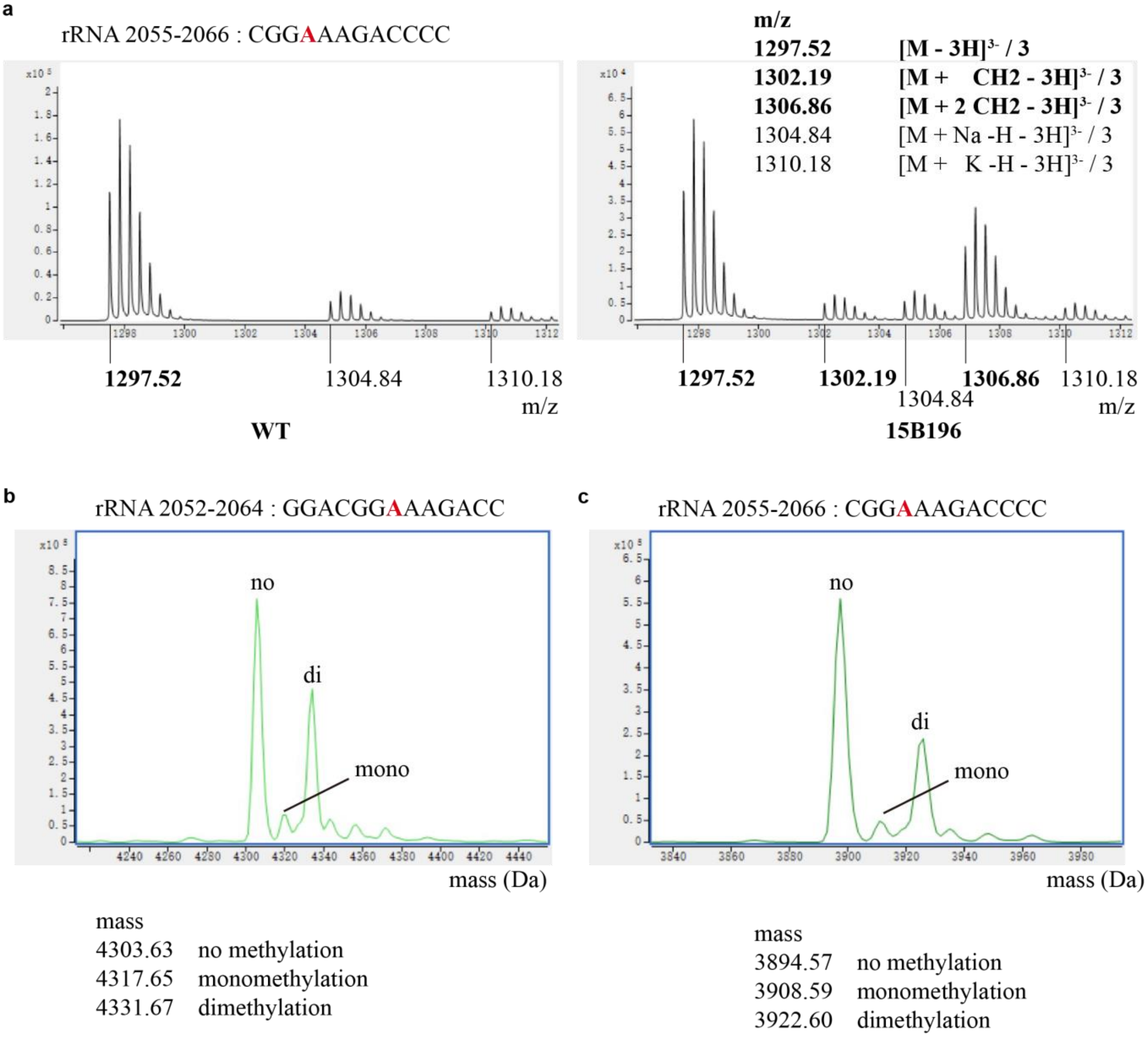

Supplementary Fig. S9 | Q-TOF mass spectrometry analysis of *S. aureus* rRNA. a, The mass spectra of rRNA C2055-C2066. In the triply deprotonated ion of the molecule, the fragment CGGAAAGACCCC runs at 1297.52 m/z (when unmethylated). Monomethylation and dimethylation at A2058 result in mass shifts of to 1302.19 and 1306.86 m/z respectively. The unmethylated fragment with a Na ion and a K ion is observed at 1304.84 m/z and 1310.18 m/z respectively. **b,c**, By integrating the peak areas, in the 15B196 ribosome, the proportions of mono-methylation were determined to be 5.7% and 6.3%, respectively. And the proportions of di-methylation were determined to be 40.4% and 37.3%, respectively.

Supplementary Fig. S10. Cryo-EM density map of modified rRNA.

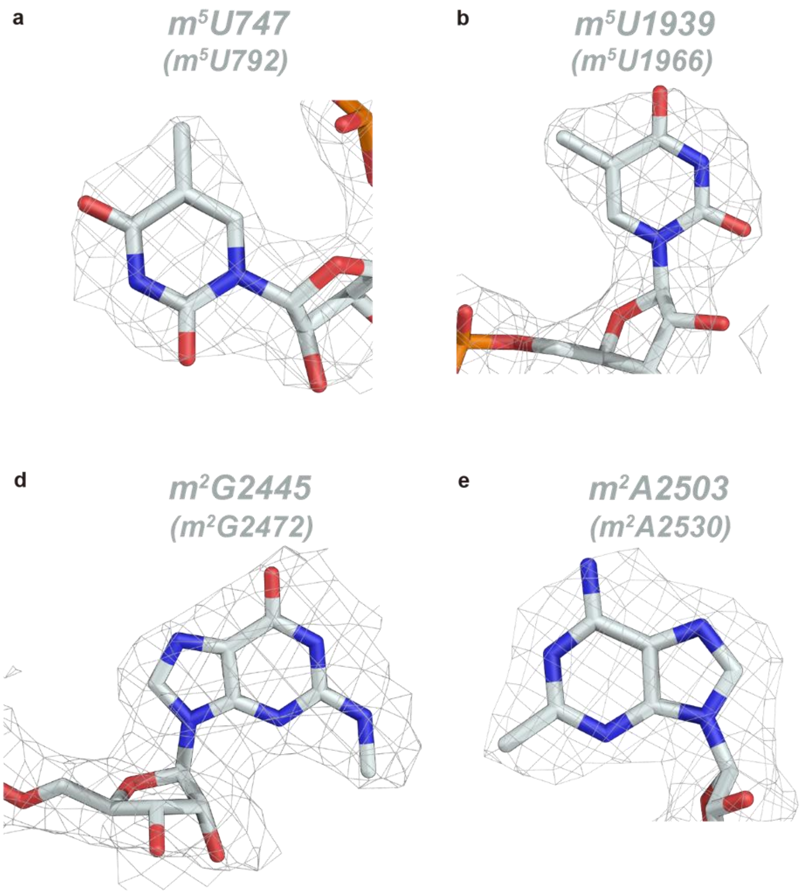

Supplementary Fig. S10 | Cryo-EM density map of modified rRNA (*E. coli* numbering is used, with *S. aureus* numbering indicated in parentheses). a, Methylation at C5 position of U747 (U792). **b**, Methylation at C5 position of U1939 (U1966). **c**, Methylation at 2'-O position of G2251 (G2278). **d**, Methylation at N2 position of G2445 (G2472). **e**, Methylation at C2 position of A2503 (A2530).

Supplementary Fig. S11. Comparison of MCX-190 and telithromycin.

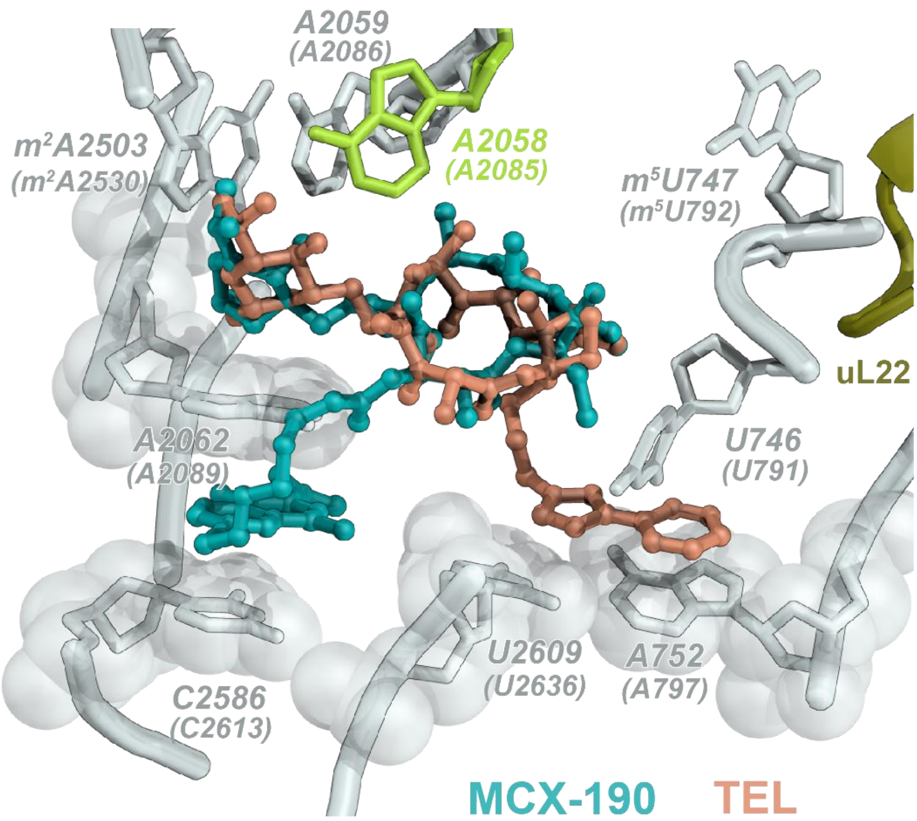

Supplementary Fig. S11 | Comparison of MCX-190 and telithromycin (*E. coli* numbering is used, with *S. aureus* numbering indicated in parentheses). Telithromycin (**TEL**) target the base pair A752-U2609 (A797-U2636), while **MCX-190** targets the new sites A2062 (A2089) and a base pair C1782-C2586 (C1809-C2613). PDB ID: **TEL**: 4v7z (*Thermus thermophilus*).

Supplementary Fig. S12. The conformation of NPET when ERY/MCX-190/TEL is bound.

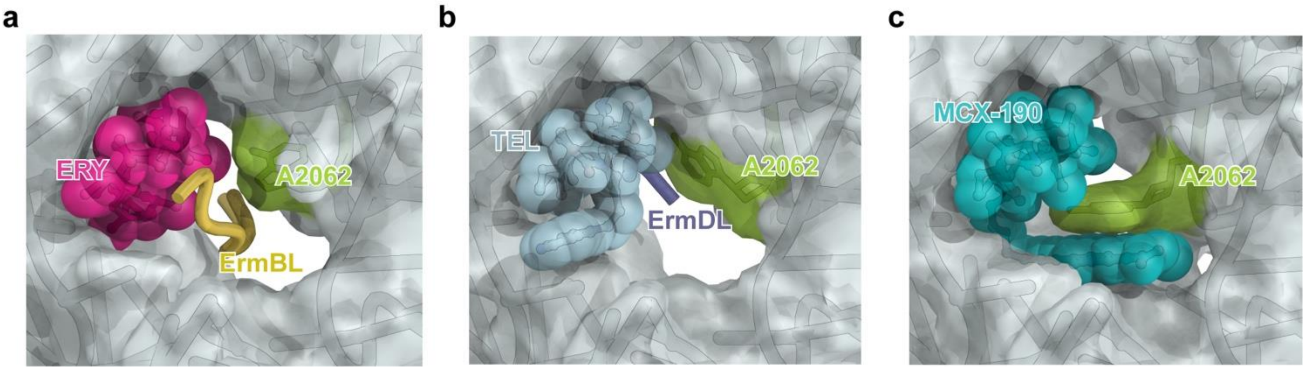

Supplementary Fig. S12 | The conformation of NPET when ERY/MCX-190/TEL is bound. a, ERY, deep pink. A2062 (A2089 in the *S. aureus*), limon. ErmBL nascent peptide, yellow. TEL, light blue. ErmDL nascent peptide, purple. **MCX-190**, cyan. PDB ID: 5JTE(ERY) and 7NSQ(TEL).

Supplementary Table S1-S8. MICs (μg/mL) of the novel synthetic macrolides.

Supplementary Table S1. Effects of 3-*O*-linkers of varying length and flexibility, substitution positions at quinolones and modification of carboxylic acid of quinolones on antibacterial activities.

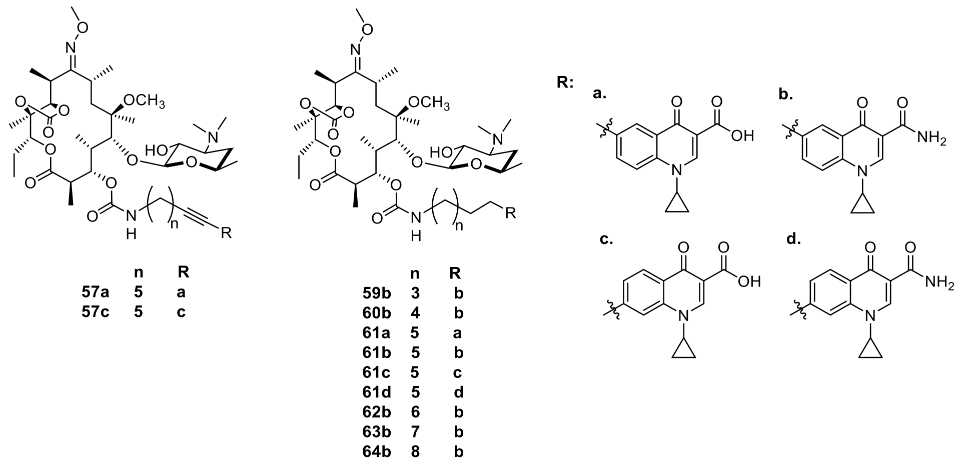

Compd.	MIC (μg/mL)						
	S. pneumoniae			*S. pyogenes*	*S. aureus*		*H. influenzae*
	ATCC49619	07P390	PU09	12-206	15B196	PU32	ATCC49247
	Ery-S	c-ermB	mef	c-ermTR	c-ermB	i-ermA	Azi-S
**57a**	0.25	64	1	32	128	16	>256
**57c**	≤0.008	0.5	0.06	1	16	4	8
**59b**	0.25	64	1	64	>256	128	>256
**60b**	0.25	32	1	32	>256	128	>256
**61a**	0.12	64	0.5	32	256	8	>256
**61b**	1	16	1	16	>256	64	>256
**61c**	≤0.008	0.5	≤0.008	2	64	8	>256
**61d**	0.12	16	1	16	>256	64	>256
**62b**	0.5	32	4	16	>256	128	>256
**63b**	2	16	4	16	>256	64	>256
**64b**	4	16	8	16	>256	64	>256
telithromycin	0.03	0.25	0.5	0.25	>256	2	4
clarithromycin	—	>256	8	>256	>256	64	16
ciprofloxacin	2	2	2	0.5	2	256	0.004

Supplementary Table S2. Effects of 3-*O*-linkers with different length and heteroatoms, as well as 11,12-cyclic rings and modification of C-9 of macrolides on antibacterial activities.

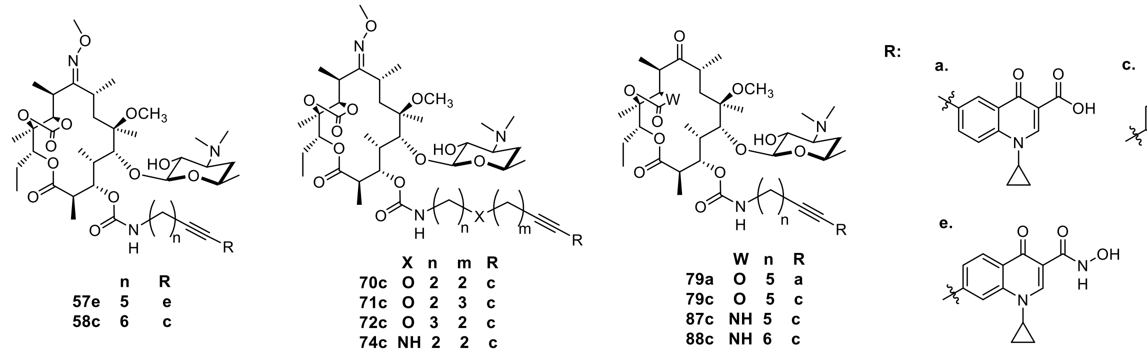

Compd.	MIC (μg/mL)						
	S. pneumoniae			*S. pyogenes*	*S. aureus*		*H. influenzae*
	ATCC49619	07P390	PU09	12-206	15B196	PU32	ATCC49247
	Ery-S	c-ermB	mef	c-ermTR	c-ermB	i-ermA	Azi-S
**57e**	0.016	2	0.5	128	128	16	32
**58c**	0.25	1	0.25	2	64	8	32
**70c**	≤0.008	0.25	0.06	2	128	4	32
**71c**	≤0.008	0.12	≤0.008	2	256	4	32
**72c**	≤0.008	0.12	≤0.008	1	32	2	16
**74c**	0.016	0.5	0.12	2	256	8	16
**79a**	0.25	64	0.5	16	128	4	64
**79c**	0.016	0.12	0.06	2	64	4	16
**87c**	≤0.008	0.06	0.03	1	64	1	8
**88c**	≤0.008	0.06	≤0.008	0.5	8	2	16
telithromycin	0.008	0.06	1	0.12	64	0.5	4
clarithromycin	0.03	512	8	512	512	64	16
ciprofloxacin	1	2	2	0.25	1	32	0.03

Supplementary Table S3. Effects of 3-*O*-linkers of varying heteroatoms and modification at N-1 and C-4 of quinolones on antibacterial activities.

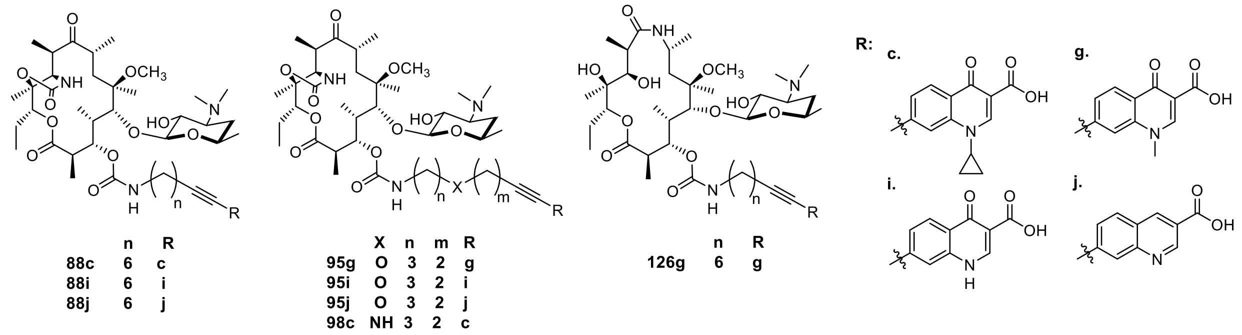

Compd.	MIC (μg/mL)						
	S. pneumoniae			*S. pyogenes*	*S. aureus*		*H. influenzae*
	ATCC49619	07P390	PU09	12-206	15B196	PU32	ATCC49247
	Ery-S	c-ermB	mef	c-ermTR	c-ermB	i-ermA	Azi-S
**88c**	0.016	0.25	≤0.008	1	4	1	4
**88i**	≤0.008	0.25	≤0.008	0.5	8	2	16
**88j**	0.5	>256	1	128	>256	8	128
**95g**	0.03	0.12	0.06	0.12	8	0.5	4
**95i**	0.12	1	0.5	1	128	8	16
**95j**	1	>256	8	128	>256	64	128
**98c**	0.5	2	0.5	2	256	4	64
**126g**	0.06	2	0.12	2	16	2	4
telithromycin	0.03	0.25	0.5	0.25	32	0.12	4
ciprofloxacin	2	2	2	1	2	64	0.008
clarithromycin	0.12	>256	8	>256	>256	>256	16

Supplementary Table S4. Effects of 3-*O*-linkers with various length, flexibility and heteroatoms, and modification at N-1 and C-3 of quinolones on antibacterial activities.

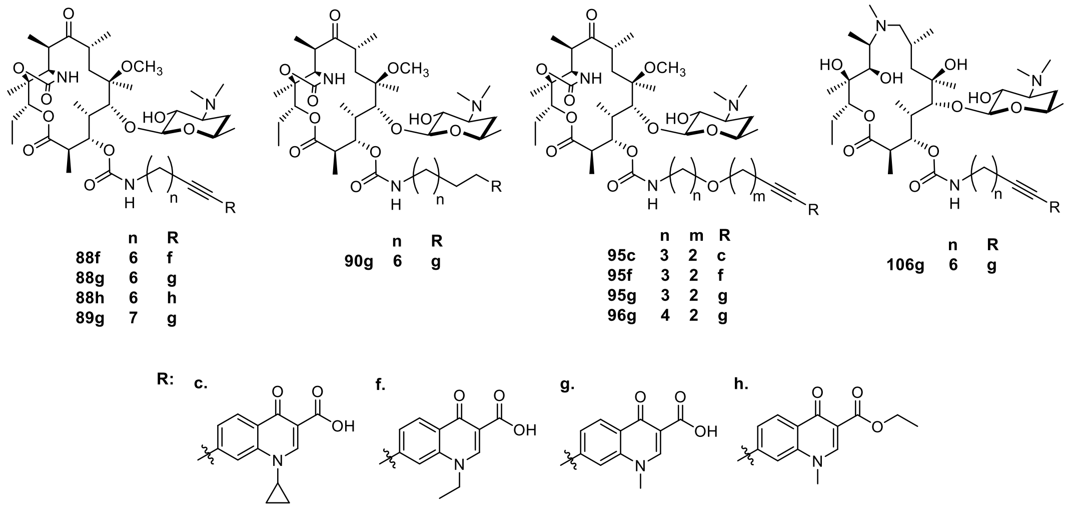

Compd.	MIC (μg/mL)					
	S. pneumoniae			*S. pyogenes*	*H. influenzae*	*M. catarrhalis*
	ATCC49619	07P390	05O173	12-206	ATCC49247	13L332
	Ery-S	c-ermB	c-ermB, mef	c-ermTR	Azi-S	Ery-S
**88f**	≤0.008	0.016	4	0.5	2	1
**88g**	≤0.008	0.03	2	0.06	2	0.5
**88h**	≤0.008	0.5	64	2	32	4
**89g**	≤0.008	0.25	16	1	8	1
**90g**	≤0.008	0.03	8	0.12	4	2
**95c**	0.03	0.25	4	0.5	2	0.5
**95f**	≤0.008	0.12	4	0.5	1	0.5
**95g**	0.016	0.06	2	0.12	1	0.25
**96g**	≤0.008	0.25	8	0.5	2	0.5
**106g**	0.25	4	128	4	8	0.5
telithromycin	0.03	0.25	4	0.5	4	0.25
clarithromycin	0.12	>256	>256	>256	16	0.25

Supplementary Table S5. Effects of quinolones' analogs on antibacterial activities.

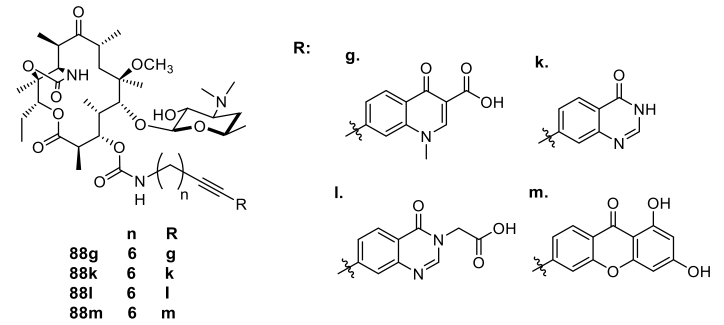

Compd.	MIC (µg/mL)					
	S. pneumoniae			*S. pyogenes*		*H. influenzae*
	ATCC49619	07P390	PU09	12-206	01-968	ATCC49247
	Ery-S	c-ermB	mef	c-ermB	i-ermTR	Azi-S
**88g**	≤0.008	0.06	≤0.008	0.12	≤0.008	4
**88k**	0.06	128	0.5	32	1	64
**88l**	0.25	256	0.25	256	8	256
**88m**	4	16	1	32	16	16
solithromycin	0.016	0.03	0.5	0.03	0.016	2
clarithromycin	0.12	>256	8	>256	4	16
ciprofloxacin	2	1	4	1	0.5	0.008

Supplementary Table S6. Effects of 3-*O*-linkers with various length and substitution positions at quinolones on antibacterial activities of 15-membered azithromycin derivatives.

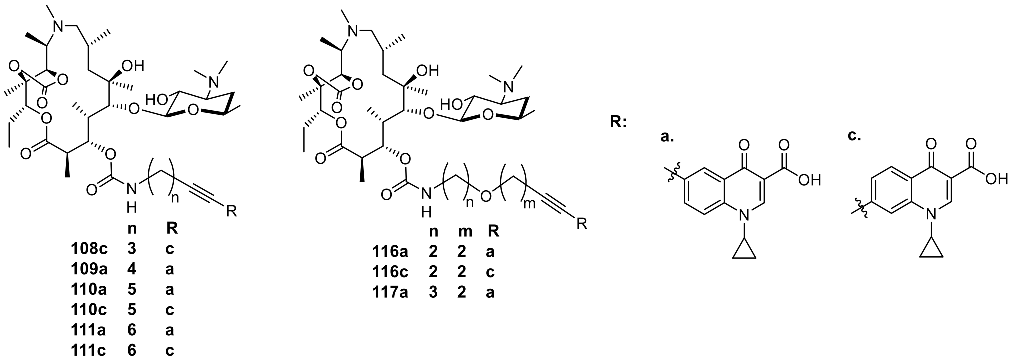

Compd.	MIC (μg/mL)						
	S. pneumoniae			*S. pyogenes*	*S. aureus*		*H. influenzae*
	ATCC49619	07P390	PU09	12-206	15B196	PU32	ATCC49247
	Ery-S	c-ermB	mef	c-ermTR	c-ermB	i-ermA	Azi-S
**108c**	0.25	128	1	64	512	64	8
**109a**	1	256	2	128	256	32	32
**110a**	1	256	2	512	256	16	32
**110c**	0.016	1	0.06	2	128	4	8
**111a**	0.5	256	2	128	128	32	16
**111c**	≤0.008	0.5	≤0.008	2	16	4	8
**116a**	4	512	8	512	512	128	64
**116c**	0.12	4	1	8	512	32	8
**117a**	1	512	2	512	512	32	32
telithromycin	0.008	0.06	1	0.12	64	0.5	4
clarithromycin	0.03	512	8	512	512	64	16
ciprofloxacin	1	2	2	0.25	1	32	0.03

Supplementary Table S7. Effects of 3-*O*-linkers with various length, flexibility and heteroatoms as well as modification at N-1 and C-4 of quinolones on antibacterial activities of 8a- and 9a-lactam derivatives.

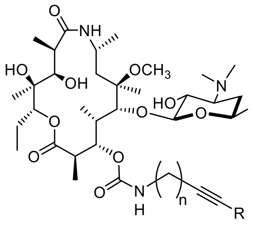

	n	R
**126c**	6	c
**126g**	6	g
**126i**	6	i
**126j**	6	j
**127i**	7	i
**128i**	8	i

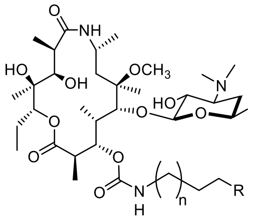

	n	R
**129i**	6	i
**130i**	7	i

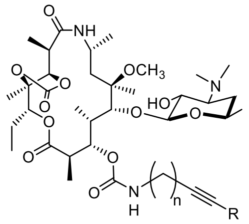

	n	R
**131i**	6	i

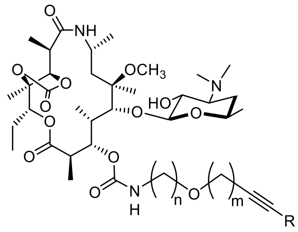

	n	m	R
**137i**	3	2	i

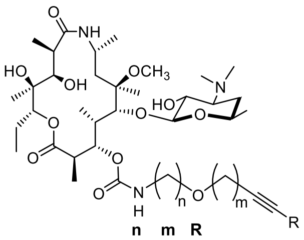

	n	m	R
**141i**	3	2	i
**141j**	3	2	j
**142i**	3	3	i
**143i**	4	2	i

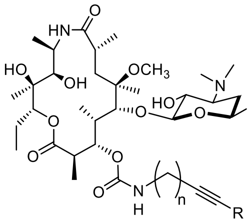

	n	R
**148i**	6	i

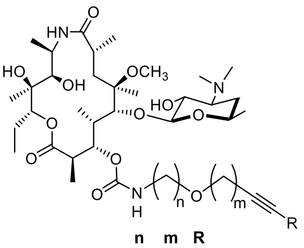

	n	m	R
**152i**	3	2	i

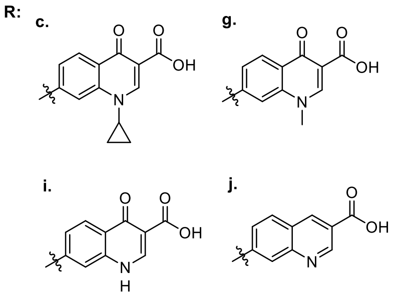

Compd.	MIC (μg/mL)						
	S. pneumoniae			*S. pyogenes*	*S. aureus*		*H. influenzae*
	ATCC49619	07P390	PU09	12-206	15B196	PU32	ATCC49247
	Ery-S	c-ermB	mef	c-ermTR	c-ermB	i-ermA	Azi-S
**126c**	0.12	2	0.25	4	16	2	4
**126g**	0.06	2	0.12	2	16	2	4
**126i**	0.25	2	0.5	4	128	8	16
**126j**	8	>256	16	>256	>256	>256	128
**127i**	0.25	4	1	16	64	4	16
**128i**	1	16	2	64	128	8	64
**129i**	0.12	1	0.25	4	64	4	16
**130i**	0.25	2	0.5	8	32	8	32
**131i**	1	8	4	16	64	32	16
**137i**	2	16	4	16	128	32	16
**141i**	2	32	4	16	>256	64	64
**141j**	32	>256	128	>256	>256	>256	256
**142i**	2	32	4	32	>256	64	32
**143i**	1	16	2	16	>256	16	16
**148i**	0.25	8	1	32	256	16	16
**152i**	2	64	4	128	>256	>256	32
telithromycin	0.03	0.25	0.5	0.25	32	0.12	4
ciprofloxacin	2	2	2	1	2	64	0.008
clarithromycin	0.12	>256	8	>256	>256	>256	16
azithromycin	0.25	>256	8	>256	>256	>256	2

Supplementary Table S8. Effects of various 15-membered macrolactones on antibacterial activities.

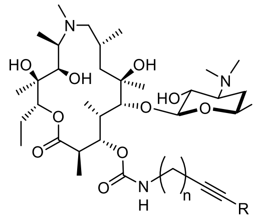

	n	R
**106g**	6	g

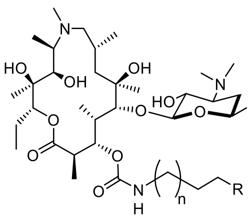

	n	R
**107g**	6	g

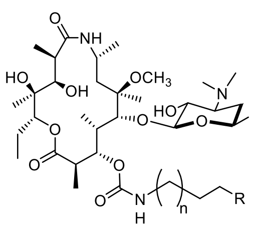

	n	R
**129g**	6	g

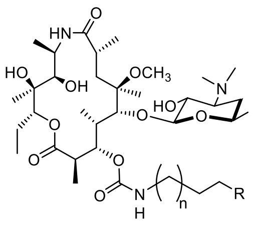

	n	R
**149g**	6	g

R: **g.**

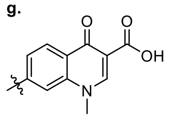

Compd.	MIC (μg/mL)					
	S. pneumoniae			*S. pyogenes*	*H. influenzae*	*M. catarrhalis*
	ATCC49619	07P390	05O173	12-206	ATCC49247	13L332
	Ery-S	c-ermB	c-ermB, mef	c-ermTR	Azi-S	Ery-S
**106g**	0.25	4	128	4	8	0.5
**107g**	0.25	4	128	8	8	1
**129g**	0.06	1	128	4	4	1
**149g**	0.25	8	64	16	8	2
telithromycin	0.03	0.25	4	0.5	4	0.25
clarithromycin	0.12	>256	>256	>256	16	0.25

Supplementary Table S9. In vitro antibacterial activity of MCX-219 and MCX-190.

	Strain description	MCX-219	MCX-190	TEL	CLA	ERY	CIP
S. aureus	**ATCC29213**	0.12	0.25	0.25	0.25	0.5	0.25
	**21B212**, Clinical, MSSA, i-ermC	0.25	0.25	0.25	>256	>256	0.5
	**21C173**, Clinical, MSSA, i-ermC	0.25	0.25	0.25	>256	>256	0.5
	**21D115**, Clinical, MSSA, i-ermC	0.25	0.5	0.12	>256	>256	0.5
	**21K194**, Clinical, MSSA, i-ermC	0.06	0.25	0.25	>256	>256	0.25
	**21P404**, Clinical, MSSA, i-ermC	0.25	0.5	0.5	>256	>256	0.5
	**21C187**, Clinical, MRSA, i-ermA	0.12	0.25	0.12	>256	>256	64
	**21F207**, Clinical, MRSA, i-ermC	0.25	0.25	0.25	>256	>256	32
	**21X388**, Clinical, MRSA, i-ermA	0.06	0.25	0.25	>256	>256	64
	**21Y223**, Clinical, MRSA, i-ermC	0.25	0.5	0.25	>256	>256	0.25
	**PU32**, Clinical, MRSA, i-ermA	0.25	0.25	0.12	16	>256	64
	**21F206**, Clinical, MSSA, c-ermB	8	32	>256	>256	>256	0.5
	**21D112**, Clinical, MSSA, c-ermB	16	64	>256	>256	>256	0.25
	**21C174**, Clinical, MSSA, c-ermB	0.25	1	>256	>256	>256	0.25
	**21P191**, Clinical, MSSA, c-ermC	16	64	>256	>256	>256	0.25
	**21R216**, Clinical, MSSA, c-ermC	16	64	>256	>256	>256	16
	**15B196**, Clinical, MRSA, c-ermB	1	8	128	>256	>256	2
	**21B200**, Clinical, MRSA, c-ermB	4	8	>256	>256	>256	0.25
	**21N354**, Clinical, MRSA, c-ermB	1	8	128	>256	>256	0.25
	**21P407**, Clinical, MRSA, c-ermB	8	16	>256	>256	>256	0.25
	**21T141**, Clinical, MRSA, c-ermB	1	4	>256	>256	>256	0.12
	**21B207**, Clinical, MRSA, c-ermB	2	8	>256	>256	>256	2
	**21F198**, Clinical, MRSA, c-ermB	1	8	>256	>256	>256	0.25
	**21Q253**, Clinical, MRSA, c-ermB	1	4	16	>256	>256	2
S. pneumoniae	**ATCC49619**	≤0.002	0.016	0.008	0.12	0.03	2
	**21Q352**, Clinical, i-MLS$_B$	≤0.002	0.25	0.06	16	64	2
	**21Q353**, Clinical, i-MLS$_B$	≤0.002	0.25	0.03	8	32	1
	**21W257**, Clinical, c-ermB, mef	0.016	0.5	0.12	> 64	> 256	4
	**21C315**, Clinical, c-ermB	0.016	0.12	0.12	> 64	> 256	2
	**21D020**, Clinical, c-ermB, mef	0.03	0.25	0.25	> 64	> 256	2
	**21D016**, Clinical, c-ermB, mef	0.03	0.5	0.25	> 64	> 256	2
	**21M277**, Clinical, c-ermB	0.25	0.5	0.12	> 64	> 256	2
	**21X435**, Clinical	≤0.002	0.016	0.016	0.12	0.03	2
S. pyogenes	**01-968**, Clinical, i-ermB	≤0.002	0.03	0.016	—	1	—
	**21K003**, Clinical, mef	0.06	1	1	2	16	0.5
	**12-207**, Clinical, mef	0.016	0.5	0.5	—	16	—
	**21C303**, Clinical, c-ermB	0.5	1	16	> 64	> 256	0.5
	**21D184**, Clinical, c-ermB	0.5	1	8	> 64	> 256	0.25
	**21M063**, Clinical, c-ermB	0.25	0.5	16	> 64	> 256	1
	**21D199**, Clinical, c-ermB	1	1	8	> 64	> 256	0.5
	**21W186**, Clinical, c-ermTR	4	2	8	> 64	> 256	0.5
	**21D200**, Clinical, c-ermB	0.25	0.5	16	> 64	> 256	0.25

Supplementary Table S10. MICs and MBCs (μg/mL) for MCX-219, telithromycin and solithromycin.

S. aureus **strain**	telithromycin			solithromycin			**MCX-219**		
	MIC	MBC	MBC/MIC	MIC	MBC	MBC/MIC	MIC	MBC	MBC/MIC
ATCC29213	0.12	>1	>8	0.06	>0.5	>8	0.25	1	4
strain 1, MRSA, i-ermA	0.25	>2	>8	0.06	>0.5	>8	0.25	1	4
strain 2, MSSA, i-ermC	0.12	>1	>8	0.06	>0.5	>8	0.25	2	8
strain 3, MSSA, i-ermC	0.12	>1	>8	0.06	>0.5	>8	0.25	2	8
strain 4, MRSA, i-ermA	0.12	>1	>8	0.06	>0.5	>8	0.12	0.5	4
strain 5, MRSA, i-ermC	0.25	>2	>8	0.06	0.5	8	1	2	2
strain 6, MRSA, c-ermB	8	>64	>8	0.25	>2	>8	2	16	8
strain 7, MRSA, c-ermB	8	>64	>8	0.5	>4	>8	2	>16	>8
strain 8, MSSA, c-ermB	>256	——	——	16	128	8	4	16	4
strain 9, MSSA, c-ermB	>256	——	——	32	>256	>8	0.5	2	4
strain 10, MRSA, c-ermB	16	>128	>8	0.25	>2	>8	4	16	4
strain 11, MRSA, c-ermB	128	——	——	0.25	>2	>8	2	>16	>8

Supplementary Table S11. Frequency of inducible resistance of S. aureus treated by MCX-219 or telithromycin.

S. aureus strain	Bacterial count (CFU)	Resistant colonies growing on different conditions					
		**MCX-219**			telithromycin		
		4 × MIC	8 × MIC	16 × MIC	4 × MIC	8 × MIC	16 × MIC
ATCC 29213	1×10^7	none	none	none	none	none	none
	1×10^8	none	none	none	none	none	none
	1×10^9	none	none	none	8	none	3
PU 32 (MRSA, i-ermA)	1×10^7	none	none	none	none	none	none
	1×10^8	none	3	none	1	none	2
	1×10^9	14	13	16	23	8	10
15B196 (MRSA, c-ermB)	1×10^7	2	none	none	NT [a]	NT	NT
	1×10^8	3	none	none	NT	NT	NT

[a] Not tested because of inactivity of telithromycin

Supplementary Table S12. *In vivo* PK parameters of MCX-219 and 88h determined in male SD rats.

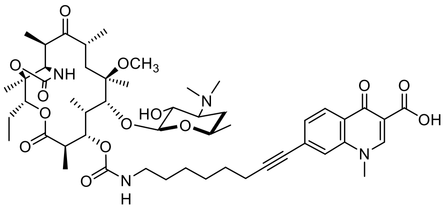

MCX-219

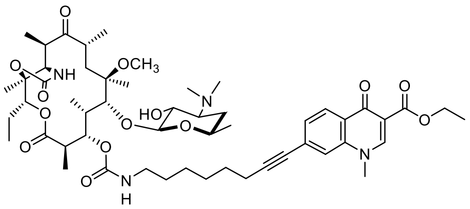

88h

	i.v. 5 mg/kg administration							
	n	AUC_{inf} h*μg/mL	AUC_{0-24} h*μg/mL	CL mL/h/kg	$t_{1/2}$ h	MRT h	T_{max} h	C_{max} μg/mL
**MCX-219**	3	7.09±1.36	7.08±1.36	733±151	3.92±0.117	1.13±0.0139	0.08	15.0±2.81
**88h**	3	1.79±0.09	1.76±0.1	2792±141	2.63±1.30	1.64±0.665	0.08	2.65±0.25

Doses for compounds **MCX-219** and **88h**: 5 mg/kg (Intravenous (i.v.) administration); n: number of rats included to estimate mean ± SD values; AUC_{inf}: concentration-time curve extrapolated to infinity; AUC_{0-24}: total area under the curve; CL: systemic plasma clearance; $t_{1/2}$: half-life; MRT: mean residence time; C_{max}: the highest drug concentration observed in plasma; T_{max}: time at which C_{max} is observed;

Supplementary Table S13. Refinement and model statistics for 50S-complex and 70S-complex.

	50S-**MCX-190** (WT S. aureus)	70S-**MCX-190** (WT S. aureus)	Methy50S-**MCX-190** (15B196 S. aureus)	Methy70S-**MCX-190** (15B196 S. aureus)
**Data collection and processing**				
Magnification	81,000x	81,000x	81,000x	81,000x
Voltage (kV)	300	300	300	300
Electron exposure (e^- /pix/s)	~ 20	~ 20	~ 20	~ 20
Number of frames per movie	40	40	40	40
Energy filter slit width (eV)	20	20	20	20
Automation software	EPU	EPU	EPU	EPU
Defocus range (µm)	-0.5 to -1.5	-0.5 to -1.5	-0.5 to -1.5	-0.5 to -1.5
Pixel size (Å)	0.824	0.824	0.824	0.824
Symmetry imposed	C1	C1	C1	C1
Number of used micrographs (no.)	10,537	10,537	11,959	11,959
Total of extracted particles (no.)	976,215	976,215	1,644,372	1,644,372
Total of refined particles (no.)	163,945	27,177	68,392	47,560
Resolution Masked 0.143 FSC (Å)	2.65	3.60	2.53	2.58
**Refinement**				
Map sharpening B-factor ($Å^2$)	-24.03	-80.89	-37.76	-48.15
Initial model used (PDB ID)	6s0z	6s13	6s0z	6s13
r.m.s. deviations				
Bond lengths (Å)	0.007	0.007	0.008	0.007
Bond angles (°)	0.910	1.072	1.209	1.071

**Validation**				
MolProbity score	2.38	2.57	2.55	2.57
All-atom clashscore	9.50	8.80	12.00	8.80
Rotamers outliers (%)	4.50	7.71	4.10	7.64
Cβ outliers (%)	0	0.30	0.53	0.28
CαBLAM outliers (%)	3.62	3.67	3.98	3.65
B-factors (min/max/mean)				
Protein	0.00/213.91/46.58	7.27/859.09/104.22	0.55/184.86/47.32	0.00/524.60/74.30
Nucletide	2.47/210.20/57.98	55.92/640.72/128.95	6.05/279.00/60.55	0.00/248.87/72.24
Ligand	15.49/51.75/27.51	64.89/84.09/77.21	10.29/32.17/20.69	14.38/37.42/28.68
Overall correlation coefficients				
CC (mask)	0.87	0.76	0.83	0.87
CC (peaks)	0.78	0.65	0.76	0.80
CC (volume)	0.85	0.74	0.82	0.86
Ramachandran plot statistics				
Favored (%)	94.37	93.52	91.79	93.54
Allowed (%)	5.37	6.37	7.98	6.35

General Procedures for Chemical Synthesis, Materials, and Instrumentation

All the reagents were purchased from commercial suppliers (Innochem and Bide Pharmatech Ltd), and used without further purification. All non-aqueous reactions were carried out in dry solvents and under an atmosphere of argon, unless otherwise noted. All of the reactions were monitored by thin-layer chromatography (TLC) using silica gel HSGF254 precoated plates (0.2 mm), and the compounds were visualized under UV light (λ = 254 nm) and/or stained with iodine. Column chromatography was performed with the indicated eluents and silica gel (100-200 mesh) purchased from Qingdao Haiyang. ^{1}H and ^{13}C spectra were taken in $CDCl_3$, DMSO-d_6, CD_3OD on Bruker Ascend 400 MHz and 700 MHz spectrometers with tetramethylsilane (TMS) as an internal standard. High resolution mass spectra (HRMS) were obtained with Agilent Q-TOF 6520 LC/MS. Melting points were determined using a SGW X-4A melting point apparatus. The purities of the target compounds for antibacterial evaluation were examined by TLC under the conditions of different eluents and visualization, and further confirmed by High-performance liquid chromatography (HPLC) and/or NMR spectra. HPLC was performed using a Shimadzu LC-20A HPLC system (Analytical column: Waters XSelect HSS T3 C_{18} column, 4.6 × 250 mm, 5 µm; solvent A: water containing 0.1% (v/v) trifluoroacetic acid, solvent B: acetonitrile).

Synthesis of compounds 4-6

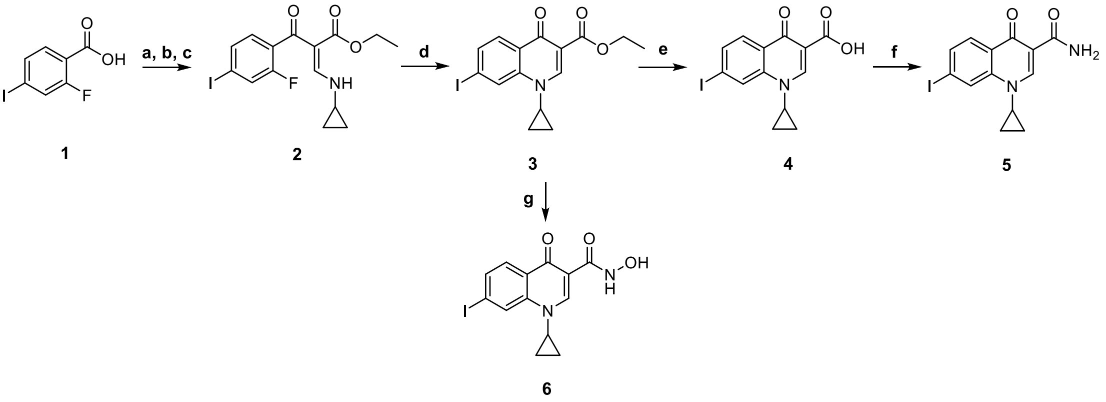

Scheme S1. Synthesis of compounds **4-6**. Reagents and conditions: (a) oxalyl chloride, DCM/DMF, rt, 1-2 h; (b) ethyl 3-(*N,N*-dimethylamino)acrylate, Et_3N, toluene, 80 ˚C, 3-4 h; (c) cyclopropylamine, EtOH/THF (1.5:1), 0 ˚C, 15-30 min; (d) K_2CO_3, DMF, 140 ˚C, 2 h; (e) NaOH, H_2O/THF (1:1), 80 ˚C, 2 h; (f) *N,N'*-carbonydiimiazole (CDI), ammonium hydroxide, DMF, 65˚C for 2 h then rt for 16 h; (g) hydroxylamine hydrochloride, NaOH, H_2O.

*Ethyl 3-(cyclopropylamino)-2-[(2-fluoro-4-iodophenyl)carbonyl]-2-propenoate**(2)***

To a solution of 2-fluoro-4-iodobenzoic acid (10.0 g, 37.6 mmol) in CH_2Cl_2 (50 mL) and DMF (1 mL), oxalyl chloride (3.80 mL, 45.1 mmol) was added dropwise. The mixture was stirred at room temperature for 1 h. The mixture was concentrated in vacuo and redissolved in toluene. Et_3N (7.30 mL, 52.6 mmol) and ethyl 3-(*N,N*-dimethylamino)acrylate (5.38 g, 37.6 mmol) were added to the reaction mixture. After stirring at 80 ˚C for 1 h, the mixture was filtered and the filtrate was concentrated in vacuo. The oil was redissolved in EtOH/THF (15 mL/10 mL). Cyclopropylamine (5.21 mL, 75.2 mmol) was added dropwise at 0 ˚C. The mixture was stirred at room temperature for 10 min. After evaporation of organic solvent, crude product was obtained. The crude product was

added petroleum ether precipitated as a white solid **2** (10.2 g, 25.2 mmol, 67.0% starting from compound **1**) that was filtered off.

Ethyl 1-cyclopropyl-1,4-dihydro-7-iodo-4-oxo-3-quinolinecarboxylate ***(3)***

To a solution of **2** (10.2 g, 25.2 mmol) in anhydrous DMF (15 mL), K_2CO_3 (10.4 g, 75.2 mmol) was added. The reaction mixture was stirred at 140 ˚C for 2 h. The mixture was filtered, and the filter cake was washed with CH_2Cl_2. The filtrate was concentrated in vacuo and precipitated with THF to obtain a white solid **3** (7.00 g, 18.3 mmol, 72.6%) that was filtered off.

General procedure A

Compound **3** or **8-9** was dissolved in a solution of EtOH and 2 N NaOH. The mixture was stirred at 80 ˚C for 2 h. The reaction mixture was neutralized with 2 M HCl, and **4** or **11-12** was precipitated as a white solid that was filtered off.

1-Cyclopropyl-1,4-dihydro -7-iodo-4-oxo-3-quinolinecarboxylic acid ***(4)***

According to the general procedure A, from compound **3** (7.00 g, 18.3 mmol), **4** (3.39 g, 9.54 mmol, 52.1%) was obtained as a white solid. HRMS (ESI) $(M+H)^+$ *m/z* 355.9787, calcd for $C_{13}H_{11}INO_3$ 355.9778. ^{1}H NMR (DMSO-d_6, 400 MHz) δ: 14.86 (s, 1 H, COOH), 8.71 (s, 1 H, 2-quinolyl), 8.61 (d, *J* = 1.4 Hz, 1 H, 8-quinolyl), 8.07 (s, *J* = 8.4 Hz, 1 H, 5-quinolyl), 8.01 (dd, *J* = 1.4 Hz, 8.4 Hz, 1 H, 6-quinolyl), 3.85 (tt, *J* = 4.0 Hz, 7.3 Hz, 1 H, 1 H-cyclopropyl), 1.36-1.22 (m, 2 H, 2 H-cyclopropyl), 1.22-1.15 (m, 2 H, 2 H-cyclopropyl).

1-Cyclopropyl-1,4-dihydro-7-iodo-4-oxo-3-quinolinecarboxamide ***(5)***

A solution of **4** (1.00 g, 2.82 mmol) in anhydrous DMF (8 mL), CDI (0.940 g, 5.63 mmol) was added, and the mixture was stirred at 65 ˚C for 2 h. Ice-cold ammonium hydroxide (20 mL) was slowly added to the mixture at room temperature and allowed to stand overnight. The mixture was filtered and the cake was dried under vacuum to obtain the title compound **5** (0.875 g, 2.47 mmol, 87.6%). HRMS (ESI) $(M+H)^+$ *m/z* 354.9930, calcd for $C_{13}H_{12}IN_2O_2$ 354.9938. ^{1}H NMR ($CDCl_3$, 400 MHz) δ: 9.57 (s, 1 H, NH_2), 8.86 (s, 1 H, 2-quinolyl), 8.38 (d, *J* = 1.5 Hz, 1 H, 8-quinolyl), 8.17 (s, *J* = 8.4 Hz, 1 H, 5-quinolyl), 7.81 (dd, *J* = 1.5 Hz, 8.4 Hz, 1 H, 6-quinolyl), 5.76 (s, 1 H, NH_2), 3.49 (tt, *J* = 3.9 Hz, 7.2 Hz, 1 H, 1 H-cyclopropyl), 1.43-1.33 (m, 2 H, 2 H-cyclopropyl), 1.22-1.13 (m, 2 H, 2 H-cyclopropyl).

1-Cyclopropyl-1,4-dihydro-7-iodo-N-hydroxy-4-oxo-3-quinolinecarboxamide ***(6)***

To a solution of **3** (0.886 g, 2.40 mmol) in water (20 mL), hydroxylamine hydrochloride (8.33 g, 120 mmol) and NaOH (5.57 g, 139 mmol) were added. The mixture was stirred at room temperature for 2 h. 2 M HCl was added to the reaction mixture. The mixture was precipitated as a white solid **6** (0.370 g, 1.00 mmol, 41.7 %) that was filtered off. HRMS (ESI) $(M+H)^+$ *m/z* 370.9885, calcd for $C_{13}H_{12}IN_2O_3$ 370.9887. ^{1}H NMR (DMSO-d_6, 400 MHz) δ: 11.55 (s, 1 H, CONH), 9.25 (s, 1 H, CONHO**H**), 8.63 (s, 1 H, 2-quinolyl), 8.49 (d, *J* = 1.4 Hz, 1 H, 8-quinolyl), 8.03 (s, *J* = 8.4 Hz, 1 H, 5-quinolyl), 7.89 (dd, *J* = 1.4 Hz, 8.4 Hz, 1 H, 6-quinolyl), 3.77 (tt, *J* = 3.9 Hz, 7.2 Hz, 1 H, 1 H-cyclopropyl), 1.36-1.26 (m, 2 H, 2 H-cyclopropyl), 1.16-1.08 (m, 2 H, 2 H-cyclopropyl).

Synthesis of compounds 10-12

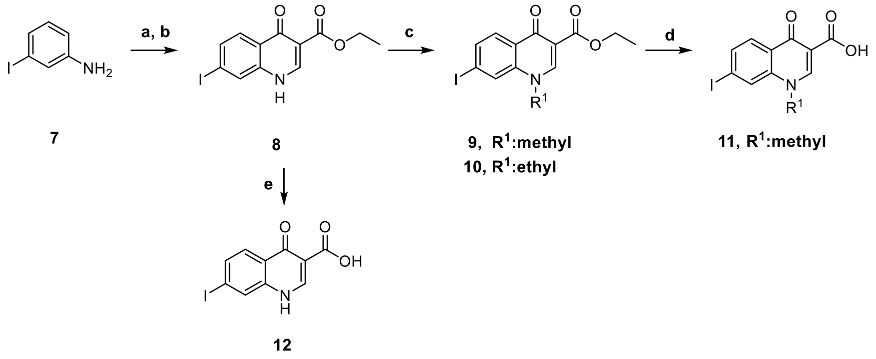

Scheme S2. Synthesis of compounds **10-12**. Reagents and conditions: (a) **7** (3-iodoaniline), diethyl 2-(ethoxymethylene)malonate, 150˚C; (b) diphenyl oxide, 250 ˚C; (c) CH_3I or CH_3CH_2I, K_2CO_3, DMF, 40 ˚C, 2 h; (d) 2 N NaOH, EtOH, 80 ˚C, 2 h; (e) 2 N NaOH, EtOH, 80 ˚C, 2 h.

Ethyl 1,4-dihydro-7-iodo-4-oxo-3-quinolinecarboxylate ***(8)***

3-Iodoaniline (5.00 mL, 41.5 mmol) was added to diethyl 2-(ethoxymethylene)malonate (8.32 mL, 41.5 mmol), and the mixture was stirred at 150˚C for 30 min. After completion of this reaction, diphenyl oxide was added to the reaction mixture. The temperature of mixture was increased to 250˚C with further stirring for 4 h. Petroleum ether was added to the mixture, and **8** (11.2 g, 33.4 mmol, 80.5%) was precipitated as a white solid that was filtered off.

General procedure B

To a solution of **8** and K_2CO_3 in DMF, CH_3I or CH_3CH_2I was added. The mixture was stirred at 40 ˚C for 2 h. After completion of this reaction, ethyl acetate and H_2O were added to the reaction mixture. The organic phase was washed with water (50 mL), saturated $NaHCO_3$ (50 mL), and brine (50 mL). After evaporation of organic solvent, petroleum ether was added to the crude product, and **9** or **10** was precipitated as a white solid that was filtered off.

Ethyl 1,4-dihydro-7-iodo-1-methyl-4-oxo-3-quinolinecarboxylate ***(9)***

According to the general procedure B, from compound **8** (1.00 g, 2.91 mmol) and CH_3I (0.36 mL, 5.83 mmol), **9** (0.830 g, 2.32 mmol, 79.8%) was obtained as a white solid.

Ethyl 1,4-dihydro-1-ethyl -7-iodo-4-oxo-3-quinolinecarboxylate ***(10)***

According to the general procedure B, from compound **8** (1.50 g, 4.37 mmol) and CH_3CH_2I (0.68 mL, 8.74 mmol), **10** (0.560 g, 1.50 mmol, 34.4%) was obtained as a white solid. HRMS (ESI) $(M+H)^+$ *m/z* 372.0090, calcd for $C_{14}H_{15}INO_3$ 372.0091.

1,4-Dihydro-7-iodo-1-methyl-4-oxo-3-quinolinecarboxylic acid ***(11)***

According to the general procedure A, from compound **9** (0.830 g, 2.32 mmol), **11** (0.612 g, 1.86 mmol, 80.0%) was obtained as a white solid. HRMS (ESI) $(M+H)^+$ *m/z* 329.9607, calcd for $C_{11}H_9INO_3$ 329.9622. ^1H NMR (DMSO-d_6, 400 MHz) δ: 15.02 (s, 1 H), 9.01 (s, 1 H), 8.34 (d, J = 1.4 Hz, 1 H), 8.08 (d, J = 8.4 Hz, 1 H), 8.01 (dd, J = 1.4, 8.4 Hz, 1 H), 4.08 (s, 3 H). ^{13}C NMR (DMSO-d_6, 100 MHz) δ: 178.05, 166.31, 151.03, 141.38, 135.68, 127.48, 127.21, 125.00, 108.25, 103.35, 42.25.

1,4-Dihydro-7-iodo-4-oxo-3-quinolinecarboxylic acid ***(12)***

According to the general procedure A, from compound **8** (2.00 g, 5.83 mmol), **12** (1.41 g, 4.47 mmol, 76.7%) was obtained as a white solid. HRMS (ESI) $(M+H)^+$ *m/z* 315.9460, calcd for $C_{10}H_7INO_3$ 315.9465. ^1H NMR (DMSO-d_6, 400 MHz) δ: 15.10 (s, 1 H), 13.29 (s, 1 H), 8.90 (s, 1 H), 8.20 (d, J = 1.6 Hz, 1 H), 8.01 (d, J = 8.6 Hz, 1 H), 7.90 (dd, J = 1.6 Hz, 8.5 Hz, 1 H). ^{13}C NMR (DMSO-d_6, 100 MHz) δ: 183.35, 171.32, 150.99, 145.52, 139.99, 133.29, 131.85, 128.93, 113.27, 107.05.

Synthesis of compounds 14-15

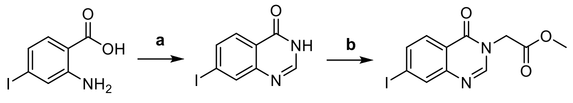

Scheme S3. Synthesis of compounds **14** and **15**. Reagents and conditions: (a) **13** (2-amino-4-iodobenzoic acid), formamidine acetate, 2-methoxyethanol, 130 ˚C, 12 h; (b) methyl bromoacetate, K_2CO_3, acetone, 1 h.

*7-iodoquinazolin-4(3H)-one **(14)***

2-Amino-4-iodobenzoic acid (2.00 g, 9.26 mmol) and formamidine acetate (1.06 g, 10.2 mmol) were dissolved in 2-methoxyethanol (25 mL), and the mixture was stirred at 130 ˚C for 12 h. After completion of this reaction, the mixture was concentrated in vacuo. 5% ammonium hydroxide was added to the reaction mixture. The mixture was precipitated as a white solid **14** (1.40 g, 5.97 mmol, 64.4%) that was filtered off. HRMS (ESI) $(M+H)^+$ *m/z* 224.9643, calcd for $C_8H_6BrN_2O$ 224.9658. ^1H NMR (DMSO-d_6, 400 MHz) δ: 12.40 (s, 1 H), 8.14 (s, 1 H), 8.03 (d, *J* = 8.5 Hz, 1 H), 7.89 (d, *J* = 2.0 Hz, 1 H), 7.69 (dd, *J* = 1.9 Hz, 8.5 Hz, 1 H).

*methyl 2-(7-iodo-4-oxoquinazolin-3(4H)-yl)acetate **(15)***

To a solution of **14** (0.500 g, 2.23 mmol) in acetone, methyl bromoacetate (0.42 mL, 4.46 mmol) and K_2CO_3 (0.370 g, 2.68 mmol) were added. The solution was allowed to stir for 1 h at room temperature before being poured into ice water (20 mL), and 2M HCl (3 mL) was added to the mixture. The mixture was precipitated as a white solid **15** (0.340 g, 1.15 mmol, 51.5%) that was filtered off. HRMS (ESI) $(M+H)^+$ *m/z* 296.9852, calcd for $C_{11}H_{10}BrN_2O_3$ 296.9869. ^1H NMR ($CDCl_3$, 400 MHz) δ: 8.18 (d, *J* = 8.5 Hz, 1 H), 8.00 (s, 1 H), 7.94 (d, *J* = 1.9 Hz, 1 H), 7.66 (dd, *J* = 1.9 Hz, 8.5 Hz, 1 H), 4.73 (s, 2 H), 1.61 (s, 3 H).

Synthesis of compound 17

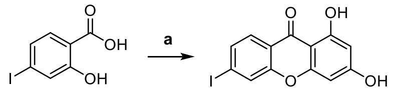

Scheme S4. Synthesis of compound **17**. Reagents and conditions: (a) **16** (2-hydroxy-4-iodobenzoic acid), phloroglucinol, $ZnCl_2$, $POCl_3$, 75˚C, 2 h.

*1,3-dihydroxy-6-iodo-9H-xanthen-9-one **(17)***

To a solution of $ZnCl_2$ (7.85 g, 57.6 mmol) in $POCl_3$ (9 mL, 97.3 mmol), 2-hydroxy-4-iodobenzoic acid (1.00 g, 3.79 mmol) and phloroglucinol (0.525 g, 4.17 mmol) were added at 75 ˚C. The solution was allowed to stir for 2 h at 75 ˚C before being poured into ice water (20 mL), and ethyl acetate (40 mL) was added to the mixture. The organic phase was washed with water and brine, and concentrated in vacuo. The crude product was purified by column chromatography (100-200 mesh silicone, ethyl acetate/ petroleum ether =1/5) to obtain **17** (1.13 g, 3.19 mmol, 84.1%). HRMS (ESI) $(M+H)^+$ *m/z* 354.9455, calcd for $C_{13}H_8IO_4$ 354.9462. ^1H NMR ($CDCl_3$, 400 MHz) δ: 12.69 (s, 1 H), 11.18 (s, 1 H), 8.06 (d, *J* = 1.3 Hz, 1 H), 7.88-7.78 (m, 2 H), 6.39 (d, *J* = 2.1 Hz, 1 H), 6.23 (d, *J* = 2.1 Hz, 1 H).

Synthesis of compounds 25-31, 34 and 37-38

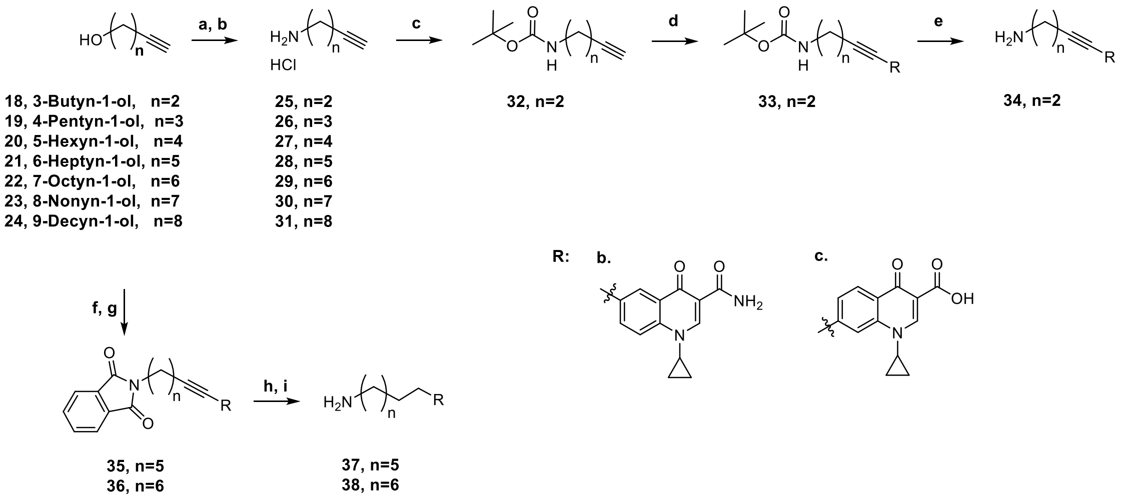

Scheme S5. Synthesis of compounds **25-31**, **34** and **37-38**. Reagents and conditions: (a) phthalimide, diisopropyl azodicarboxylate (DIAD), PPh_3, toluene, 0 ˚C- rt, 2 h; (b) 80% hydrazine hydrate, CH_3CH_2OH, 80 ˚C, 2 h; (c) di-tert-butyl decarbonate, Et_3N, THF, 0.5 h, rt; (d) **4**, CuI, $Pd(PPh_3)_2Cl_2$, Et_3N, CH_3CN, 45 ˚C, 12 h; (e) 12M HCl, CH_3CH_2OH, rt, 1 h; (f) phthalimide, DIAD, PPh_3, toluene, 0 ˚C- rt, 2 h; (g) R-I (R = **b**), CuI, $Pd(PPh_3)_2Cl_2$, Et_3N, CH_3CN, 80 ˚C, 4 h; (h) HCOOH, $HCOONH_4$, 10% Pd/C, H_2, CH_3OH, 60 ˚C, 16 h; (i) 80% hydrazine hydrate, CH_3CH_2OH, 80 ˚C.

General procedure C

Phthalimide (1 eq), PPh_3 (1.1 eq), and **18-24** (1.05 eq) were dissolving in toluene, and DIAD (1.1 eq) was added dropwise at 0 ˚C. The solution was allowed to stir for 2 h at room temperature. The solution was concentrated in vacuo and purified by column chromatography to obtain an oil.

The oil was dissolved in EtOH, and 80% hydrazine hydrate (2 eq) was added to the solution. The mixture was stirred at 80˚C for 2 h. 12 M HCl was added to the solution, and then filtered. After evaporation of the filtrate, ethyl acetate was added to the oil, and **25-31** was precipitated as a white solid that was filtered off.

3-Butyn-1-amine, hydrochloride (1:1) ***(25)***

According to the general procedure C, from **18** (2.64 mL, 36.7 mmol), **25** (2.80 g, 26.5 mmol, 72.3%) was obtained as a white solid.

4-Pentyn-1-amine, hydrochloride (1:1) ***(26)***

According to the general procedure C, from **19** (1.3 mL, 14.3 mmol), **26** (1.27 g, 10.6 mmol, 74.1%) was obtained as a white solid. HRMS (ESI) $(M+H)^+$ *m/z* 84.0806, calcd for C_5H_{10} 84.0808.

5-Hexyn-1-amine, hydrochloride (1:1) ***(27)***

According to the general procedure C, from **20**, **27** was obtained as a white solid.

6-Heptyn-1-amine, hydrochloride (1:1) ***(28)***

According to the general procedure C, from **21** (4.60 mL, 36.7 mmol), **28** (4.52 g, 30.6 mmol, 83.5%) was obtained as a white solid. HRMS (ESI) $(M+H)^+$ *m/z* 112.1125, calcd for $C_7H_{14}N$ 112.1121.

7-Octyn-1-amine, hydrochloride (1:1) ***(29)***

According to the general procedure C, from **22** (2.03 mL, 14.3 mmol), **29** (1.66 g, 10.3 mmol, 72.2%) was obtained as a white solid. HRMS (ESI) $(M+H)^+$ *m/z* 126.1268, calcd for $C_8H_{16}N$ 126.1277.

8-Nonyn-1-amine, hydrochloride (1:1) ***(30)***

According to the general procedure C, from **23** (3.55 mL, 21.4 mmol), **30** (2.85 g, 16.2 mmol, 75.9%) was obtained as a white solid.

9-Decyn-1-amine, hydrochloride (1:1) ***(31)***

According to the general procedure C, from **24** (3.98 mL, 21.4 mmol), **31** (2.64 g, 17.2 mmol, 80.2%) was obtained as a white solid.

tert-Butyl but-3-yn-1-ylcarbamate ***(32)***

To a solution of **25** (0.211g, 2.00 mmol) in THF (20 mL), di-tert-butyl decarbonate (0.436 g, 2.00 mmol) and Et_3N (0.56 mL, 4.00 mmol) were added. The mixture was stirred at room temperature for 30 min, followed by the addition of ethyl acetate. The mixture was washed with water and brine, and concentrated in vacuo to obtain an oil **32**.

1-Cyclopropyl-7-[4-[[(1,1-dimethylethoxy)carbonyl]amino]-1-butyn-1-yl]-1,4-dihydro-4-oxo-3-quinolinecarboxylic acid ***(33)***

TEA (5mL) was added to a stirred suspension of **4** (0.500 g, 1.41 mmol) in CH_3CN (5 mL). The mixture was stirred at room temperature for 20 min. CuI (0.0179 g, 0.0939 mmol), $Pd(PPh_3)_2Cl_2$ (0.0328 g, 0.0468 mmol) and **32** which was obtained in previous step were added. The reaction mixture was flushed with argon, sealed in a pressure tube, and stirred at 45 ˚C for 12 h. After completion of the reaction, CH_2Cl_2 (50 mL) and water (50 mL) were added to the reaction mixture. The organic layer was washed with water and brine and concentrated in vacuo. The crude mixture was purified by column chromatography on silica gel to obtain **45** (0.330 g, 0.832 mmol, 59.1%).

7-(4-Amino-1-butyn-1-yl)-1-cyclopropyl-1,4-dihydro-4-oxo-3-quinolinecarboxylic acid ***(34)***

To a solution of **33** (0.330 g, 0.832 mmol) in EtOH, 6M HCl was added dropwise. After stirring at room temperature for 1 h, ethyl acetate (40 mL) was added to the mixture. The mixture was filtered to obtain **34** (0.220 g, 0.661 mmol, 79.4%). HRMS (ESI) $(M+H)^+$ *m/z* 297.1234, calcd for $C_{17}H_{17}N_2O_3$ 297.1234. ^1H NMR (DMSO-d_6, 400 MHz) δ: 8.73 (s, 1 H, 2-quinolyl), 8.34 (d, *J* = 1.3 Hz, 1 H, 8-quinolyl), 8.30 (d, *J* = 8.3 Hz, 1 H, 5-quinolyl), 7.71 (d, *J* = 1.3 Hz, 8.3 Hz, 1 H, 6-quinolyl), 3.83 (tt, *J* = 4.0 Hz, 7.3 Hz, 1 H, 1 H-cyclopropyl), 3.14-3.03 (m, 2 H, NH_2-C**H**$_2$), 2.93 (t, *J* = 7.0 Hz, 2 H, C**H**$_2$-C≡C-quinolyl), 1.38-1.30 (m, 2 H, 2 H-cyclopropyl), 1.21-1.14 (m, 2 H, 2 H-cyclopropyl).

General procedure D

Phthalimide (1 eq), PPh_3 (1.1 eq) and **21-22** (1.1 eq) were dissolving in toluene, and DIAD (1.1 eq) was added dropwise at 0 ˚C. The solution was allowed to stir for 2 h at room temperature. The solution was concentrated in vacuo to obtain a yellow oil.

To a solution of the yellow oil, 1-cyclopropyl-6-iodo-1,4-dihydro-4-oxo-3-quinolinecarboxamide, CuI, $Pd(PPh_3)_2Cl_2$ in CH_3CN and Et_3N were added. The reaction mixture was flushed with argon, sealed in a pressure tube and stirred at 80 ˚C for 4 h. After completion of the reaction, CH_2Cl_2 (50 mL) and water (50 mL) were added into the reaction mixture. The organic layer was washed with water and brine, concentrated in vacuo, and purified by column chromatography to obtain **35-36**.

*1-Cyclopropyl-6-[6-(1,3-dihydro-1,3-dioxo-2H-isoindol-2-yl)-1-heptyn-1-yl]-1,4-dihydro-4-oxo-3-quinolinecarboxamide****(35)***

According to the general procedure D, from **21** (0.20 mL, 1.57 mmol), **35** (0.325 g, 0.69 mmol, 43.8%) was obtained as a white solid. HRMS (ESI) $(M+H)^+$ *m/z* 468.1913, Calcd for $C_{28}H_{26}N_3O_4$ 468.1918.

*1-Cyclopropyl-6-[7-(1,3-dihydro-1,3-dioxo-2H-isoindol-2-yl)-1-octyn-1-yl]-1,4-dihydro-4-oxo-3-quinolinecarboxamide****(36)***

According to the general procedure G, from **22**, **36** was obtained as a white solid.

General procedure E

Compound **53** (1 eq) was dissolved in CH_3OH (10 mL). HCOOH (8 eq), $HCOONH_4$ (16 eq) and 10% Pd/C (10% mass) were added to the solution. The mixture was stirred at room temperature under H_2 atmosphere for 12 h. The solvent was evaporated out and the residue was purified by column chromatography.

General procedure F

Compound was dissolved in EtOH, and 80% hydrazine hydrate was added to the solution. The mixture was

stirred at 80 ˚C for 2 h. The solvent was concentrated in vacuo, and purified by column chromatography.

*1-Cyclopropyl-6-[6-aminoheptyl]-1,4-dihydro-4-oxo-3-quinolinecarboxamide**(37)***

According to the general procedure E and F, from **35** (0.325, 0.690 mmol), **37** (0.180 g, 0.530 mmol, 76.8%) was obtained as a white solid.

*1-Cyclopropyl-6-[7-aminooctyl]-1,4-dihydro-4-oxo-3-quinolinecarboxamide**(38)***

According to the general procedure E and F, from **36**, **38** was obtained as a white solid.

Synthesis of compounds 47-50

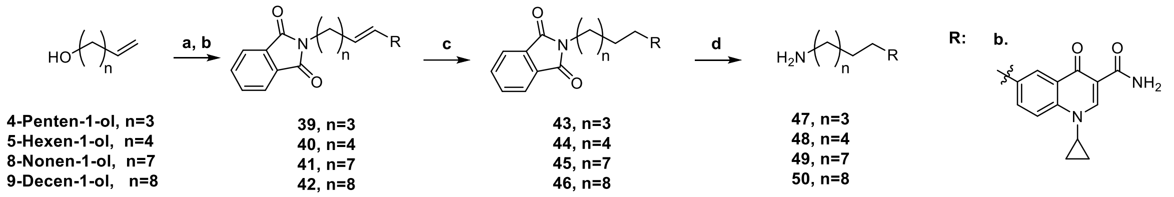

Scheme S6. Synthesis of compounds **47-50**. Reagents and conditions: (a) phthalimide, DIAD, PPh_3, toluene, 0 ˚C-rt, 2 h; (b) RI (R = **b**), $Pd(OAc)_2$, P(*o*-MePh)$_3$, Et_3N, CH_3CN, 60 ˚C for 1 h then 90˚C for 24 h; (c) HCOOH, $HCOONH_4$, 10% Pd/C, H_2, CH_3OH, 60 ˚C, 16 h; (d) 80% hydrazine hydrate, CH_3CH_2OH, 80 ˚C, 2 h.

General procedure G

Phthalimide (1 eq), PPh_3 (1.1 eq) and alkynol (1.1 eq) were dissolved in toluene, and DIAD (1.1 eq) was added dropwise at 0 ˚C. The solution was allowed to stir for 2 h at room temperature. The solution was concentrated in vacuo to a yellow oil.

To a solution of the oil (1 eq), 1-cyclopropyl-6-iodo-1,4-dihydro-4-oxo-3-quinolinecarboxamide (0.8 eq), $Pd(OAc)_2$ (0.25 eq) and P(*o*-MePh)$_3$ (0.5 eq) in CH_3CN, Et_3N (2.5 eq) was added. The reaction mixture was flushed with argon, sealed in a pressure tube and stirred at 60 ˚C for 1 h. After then the temperature of the mixture was increased to 90˚C with further stirring for 24 h. After completion of the reaction, CH_2Cl_2 (50 mL) and water (50 mL) were added into the reaction mixture. The organic layer was washed with water and brine, concentrated in vacuo, and purified by column chromatography to yield **39-42**.

*1-Cyclopropyl-6-[4-(1,3-dihydro-1,3-dioxo-2H-isoindol-2-yl)-1-penten-1-yl]-1,4-dihydro-4-oxo-3-quinolinecarboxamide **(39)***

According to the general procedure G, from 4-penten-1-ol (0.18 mL, 1.70 mmol), **39** (0.420 g, 0.951 mmol, 61.5 %) was obtained as a white solid. (column chromatography conditions: 100-200 mesh silicone, $CH_2Cl_2/EtOH/NH_3.H_2O$ = 10/0.1/0.1). HRMS (ESI) $(M+H)^+$ *m/z* 442.1757, Calcd for $C_{26}H_{24}N_3O_4$ 442.1761.

*1-Cyclopropyl-6-[5-(1,3-dihydro-1,3-dioxo-2H-isoindol-2-yl)-1-hexen-1-yl]-1,4-dihydro-4-oxo-3-quinolinecarboxamide**(40)***

According to the general procedure G, from 5-hexen-1-ol (0.17 mL, 1.47 mmol), **40** (0.410 g, 0.900 mmol, 61.3%) was obtained as a white solid. (column chromatography conditions: 100-200 mesh silicone, $CH_2Cl_2/EtOH/NH_3.H_2O$ = 10/0.1/0.1).

*1-Cyclopropyl-6-[8-(1,3-dihydro-1,3-dioxo-2H-isoindol-2-yl)-1-nonen1-yl]-1,4-dihydro-4-oxo-3-quinolinecarboxamide**(41)***

According to the general procedure G, from 8-nonen-1-ol, **41** was obtained as a white solid.

*1-Cyclopropyl-6-[9-(1,3-dihydro-1,3-dioxo-2H-isoindol-2-yl)-1-decen-1-yl]-1,4-dihydro-4-oxo-3-quinolinecarboxamide**(42)***

According to the general procedure G, from 9-decen-1-ol, **42** was obtained as a white solid.

*1-Cyclopropyl-6-[4-(1,3-dihydro-1,3-dioxo-2H-isoindol-2-yl) pentyl]-1,4-dihydro-4-oxo-3-quinolinecarboxamide**(43)***

According to the general procedure E, from **39** (0.420 g, 0.950 mmol), **43** (0.400 g, 0.900 mmol, 94.7%) was obtained as a white solid. HRMS (ESI) $(M+H)^+$ *m/z* 444.1914, Calcd for $C_{26}H_{26}N_3O_4$ 444.1918.

*1-Cyclopropyl-6-[5-(1,3-dihydro-1,3-dioxo-2H-isoindol-2-yl) hexyl]-1,4-dihydro-4-oxo-3-quinolinecarboxamide**(44)***

According to the general procedure E, from **40** (0.641 g, 1.41 mmol), **44** (0.594 g, 1.30 mmol, 92.2%) was obtained as a white solid. HRMS (ESI) $(M+H)^+$ *m/z* 458.2066, Calcd for $C_{27}H_{28}N_3O_4$ 458.2074.

*1-Cyclopropyl-6-[8-(1,3-dihydro-1,3-dioxo-2H-isoindol-2-yl) nonyl]-1,4-dihydro-4-oxo-3-quinolinecarboxamide**(45)***

According to the general procedure E, from **41**, **45** was obtained as a white solid.

*1-Cyclopropyl-6-[9-(1,3-dihydro-1,3-dioxo-2H-isoindol-2-yl) decyl]-1,4-dihydro-4-oxo-3-quinolinecarboxamide**(46)***

According to the general procedure E, from **42**, **46** was obtained as a white solid.

*1-Cyclopropyl-6-(4-aminopentyl)-1,4-dihydro-4-oxo-3-quinolinecarboxamide**(47)***

According to the general procedure F, from **43** (0.400 g, 0.900 mmol), **47** (0.180 g, 0.570 mmol, 63.3%) was obtained as a white solid.

*1-Cyclopropyl-6-(5-aminohexyl)-1,4-dihydro-4-oxo-3-quinolinecarboxamide**(48)***

According to the general procedure F, from **44** (0.594 g, 1.30 mmol), **48** (0.130 g, 0.400 mmol, 30.8%) was obtained as a white solid.

*1-Cyclopropyl-6-(8-aminononyl)-1,4-dihydro-4-oxo-3-quinolinecarboxamide**(49)***

According to the general procedure F, from **45**, **49** was obtained as a white solid.

*1-Cyclopropyl-6-(9-aminodecyl)-1,4-dihydro-4-oxo-3-quinolinecarboxamide**(50)***

According to the general procedure F, from **46**, **50** was obtained as a white solid.

Synthesis of compounds 57-64

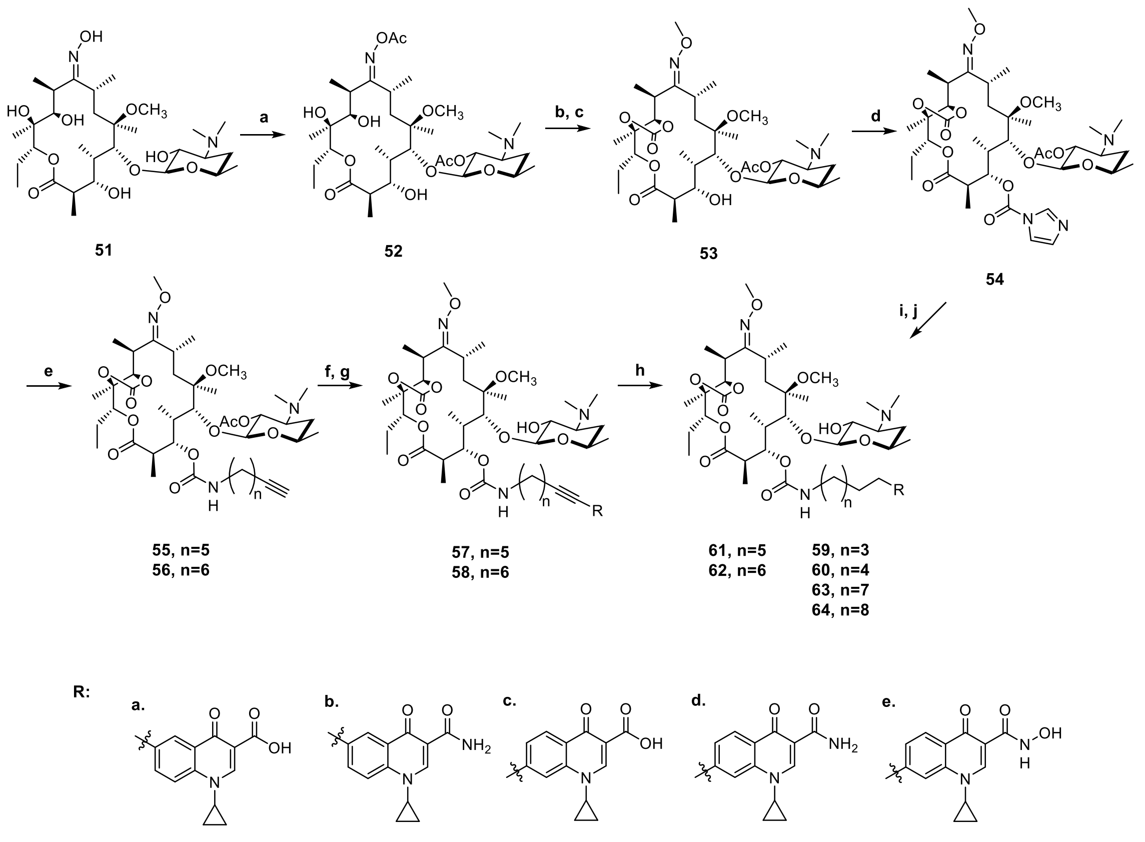

Scheme S7. Synthesis of *erythromycin A 9-O-methyl oxime 11,12-cyclic carbonate* series compounds **57-64**. Reagents and conditions: (a) 1) acetic anhydride, DCM, rt, 1-1.5 h; 2) sat. $NaHCO_3$; (b) $KOBu^t$, CH_3I, DMSO/THF (1:1), rt, 0.5-1 h; (c) pyridine, bis(trichloromethyl)carbonate (BTC), DCM, -10 °C for 4 h then rt for 18 h; (d) CDI,

4-dimethylaminopyridine (DMAP), DCM, rt, 12 h; (e) **28** or **29**, 1,8-Diazabicyclo[5.4.0]undec-7-ene (DBU), DMF, rt, 12 h; (f) R-I (R = **a**, **c**, **e**), CuI, $Pd(PPh_3)_2Cl_2$, Et_3N, CH_3CN, 45 ˚C, 12 h; or R-I (R = **d**), CuI, $Pd(PPh_3)_2Cl_2$, Et_3N, CH_3CN, 80 ˚C, 4 h; (g) CH_3OH, 65 ˚C, 2 h; (h) HCOOH, $HCOONH_4$, 10% Pd/C, H_2, CH_3OH, rt, 16 h; (i) **37-38**, **47-50**, DBU, DMF, rt, 12 h; (j) CH_3OH, 65 ˚C, 2 h.

*2'-O-Acetyl-3-O-descladinosyl-3-hydroxy-6-O-methylerythromycin A 9-O-acetyl oxime **(52)***

To a solution of compound **51** (10.0 g, 16.5 mmol) in anhydrous CH_2Cl_2 (50 mL), acetic anhydride (4.69 mL, 49.6 mmol) was added. The mixture was stirred at room temperature for 1-2 h. The organic layer was washed with saturated $NaHCO_3$ (5 × 50 mL), water and brine, and evaporated in vacuo to yield **52** (11.3 g, 16.5 mmol, 99.6 %).

*2'-O-Acetyl-3-O-descladinosyl-3-hydroxy-6-O-methylerythromycin A 9-O- methyl oxime 11,12- cyclic carbonate **(53)***

The compound **52** (11.3 g, 16.5 mmol) and $KOBu^t$ (2.77 g, 24.7 mmol) were dissolved in DMSO (60 mL) and THF (60 mL). The mixture was stirred at room temperature for 10 min. $KOBu^t$ (1.85 g, 16.5 mmol) and methyl iodide (1.02 mL, 16.5 mmol) were added into the reaction. The mixture was stirred at 25˚C for 20 min followed by the addition of ethyl acetate (50 mL) and water (50 mL). The organic layer was washed with water and brine, and concentrated in vacuo to yield an intermediate (9.96 g, 15.1 mmol, 91.6%).

To a solution of intermediate (9.96 g, 15.1 mmol) of the previous step in CH_2Cl_2, pyridine (14.5 mL, 179 mmol) was added. A solution of BTC (8.95 g, 15.1 mmol) in CH_2Cl_2 was added dropwise at -15 ˚C. After being stirred at -15 ˚C for 4 h, the reaction was warmed to room temperature and stirred for 10 h. This solution was washed with water (50 mL), saturated $NaHCO_3$ (50 mL), and brine. The organic layer was concentrated in vacuo and purified by column chromatography (100-200 mesh silicone, CH_2Cl_2/EtOH/$NH_3.H_2O$=10/0.1/0.1) to yield **53** (2.06 g, 2.99 mmol, 19.8%).

*2'-O-Acetyl-3-O-descladinosyl-3-O-(1H-imidazol-1-ylcarbonyl)-6-O-methylerythromycin A 9-O- methyl oxime 11,12- cyclic carbonate **(54)***

Compound **53** (2.21 g, 3.21 mmol), CDI (1.56 g, 9.64 mmol) and DMAP (0.785 g, 6.43 mmol) were dissolved in anhydrous CH_2Cl_2. The mixture was stirred at 25˚C for 12-18 h followed by the addition of CH_2Cl_2 (50 mL) and water (50 mL). The organic layer was washed with water and brine, and concentrated in vacuo and purified by column chromatography (100-200 mesh silicone, CH_2Cl_2/EtOH/$NH_3.H_2O$=10/0.1/0.1) to yield **54** (1.38 g, 1.78 mmol, 55.2%).

General procedure H

Compound **54** (1 eq) and **25-31** (1.5 eq) were dissolved in anhydrous DMF. DBU (2 eq) was added to the reaction mixture. The reaction mixture was stirred for 16 h at room temperature. After completion of the reaction, ethyl acetate (50 mL) and water (50 mL) were added into the reaction mixture. The organic layer was washed with water and brine, and concentrated in vacuo and purified by column chromatography.

*2'-O-Acetyl-3-O-descladinosyl-3-O-(N-6-heptynylcarbamoyl)-6-O-methylerythromycin A 9-O- methyl oxime 11,12-cyclic carbonate **(55)***

According to the general procedure H, from compound **54** (2.46 g, 3.16 mmol) and **28** (0.560 g, 3.79 mmol), **55** (1.30 g, 1.58 mmol, 50.1%) was obtained as a white solid (column chromatography eluents: DCM/EtOH/$NH_3·H_2O$ = 10:0.1:0.1).

*2'-O-Acetyl-3-O-descladinosyl-3-O-(N-7-octynylcarbamoyl)-6-O-methylerythromycin A 9-O- methyl oxime 11,12-cyclic carbonate **(56)***

According to the general procedure H, from compound **54** (0.500 g, 0.641 mmol) and **29** (0.124 g, 0.769 mmol), **56** (0.421 g, 0.502 mmol, 86.2%) was obtained as a white solid (column chromatography eluents: DCM/EtOH/$NH_3·H_2O$ = 10:0.1:0.1).

General procedure I-1

TEA (5 mL) was added to a stirred suspension of **ArI** (1.2 eq) in CH_3CN (5 mL) and stirred at room temperature for 20 min. And CuI (0.1 eq), $Pd(PPh_3)_2Cl_2$ (0.05 eq) and compound **55-56** (1 eq) were added. The reaction mixture

was flushed with argon, sealed in a pressure tube and stirred at 45 ˚C for 12 h. After completion of the reaction, CH_2Cl_2 (50 mL) and water (50 mL) were added into the reaction mixture. The organic layer was washed with water and brine, and concentrated in vacuo. The product was then dissolved in MeOH (15 mL) to remove the protecting group. The organic solvent was removed in vacuo. The crude mixture was purified by column chromatography on silica gel.

General procedure I-2

To a solution of the **55** (1 eq), **ArI** or **ArBr** (1.1 eq), CuI (0.1 eq) and $Pd(PPh_3)_2Cl_2$ (0.05 eq) in CH_3CN, Et_3N (3 eq) was added. The reaction mixture was flushed with argon, sealed in a pressure tube and stirred at 80 ˚C for 4 h. After completion of the reaction, CH_2Cl_2 (50 mL) and water (50 mL) were added into the reaction mixture. The organic layer was washed with water and brine, concentrated in vacuo. The product was then dissolved in MeOH (15 mL) to remove the protecting group. The organic solvent was removed in vacuo. The crude mixture was purified by column chromatography on silica gel.

3-O-Descladinosyl-3-O-[N-7-(1-cyclopropyl-1,4-dihydro-4-oxo-3-quinolinecarboxylic acid-6-yl)-hept-6-ynylcarbamoyl]-6-O-methylerythromycin A 9-O- methyl oxime 11,12- cyclic carbonate ***(57a)***

Following the general procedure I-1, from compound **55** (0.300 g, 0.364 mmol) and 1-cyclopropyl-1,4-dihydro-4-oxo-6-iodo-3-quinolinecarboxylic acid (0.155 g, 0.437 mmol), **57a** (30.0 mg, 0.0297 mmol，8.17%) was obtained as an off-white solid (column chromatography eluents: DCM/MeOH/NH_3·H_2O = 10:0.8:0.5). HRMS (ESI) $(M+H)^+$ *m/z* 1009.5377, calcd for $C_{53}H_{77}N_4O_{15}$ 1009.5380. ^1H NMR ($CDCl_3$, 400 MHz) δ: 8.85 (s, 1 H, 2″-quinolyl), 8.47 (d, *J* = 1.9 Hz, 1 H, 5″-quinolyl), 8.02 (d, *J* = 8.8 Hz, 1 H, 8″-quinolyl), 7.80 (dd, *J* = 1.9 Hz, 8.8 Hz, 1 H, 7″-quinolyl), 5.16 (dd, *J* = 2.5 Hz, 10.7 Hz, 1 H, H-13), 5.11 (br, 1 H, 3-*O*-CO-N**H**-CH_2), 4.92-4.80 (m, 2 H, H-3, H-11), 4.04 (d, *J* = 7.2 Hz, 1 H, H-1′), 3.82 (s, 3 H, 9-*O*-CH_3), 3.75 (d, *J* = 3.1 Hz, 1 H, H-5), 3.73-3.65 (m, 1 H, H-8), 3.65-3.57 (m, 1 H, 1 H-cyclopropyl), 3.45-3.32 (m, 2 H, H-5′, 3-*O*-CO-NH-C**H**$_2$), 3.22 (dd, *J* = 7.2 Hz, 10.1 Hz, 1 H, H-2′), 3.16-3.04 (m, 1H, 3-*O*-CO-NH-C**H**$_2$), 3.02 (s, 3 H, 6-*O*-CH_3), 2.90-2.80 (m, 1 H, H-2), 2.61-2.50 (m, 2 H, H-3′, H-10), 2.48 (t, *J* = 8.45 Hz, 2 H, -C**H**$_2$-C≡C-quinolyl), 2.35 (s, 6 H, -N(CH_3)$_2$), 2.12-2.01 (m, 1 H, H-4), 1.95-1.83 (m, 1 H, H-14eq), 1.75-1.50 (m, 8 H, 3(-CH_2-), H-4′a, H-14ax), 1.49 (s, 3 H, 12-CH_3), 1.45-1.35 (m, 3 H, 2 H-cyclopropyl, H-7a), 1.32 (s, 3 H, 6-CH_3), 1.45-1.35 (m, 10 H, 2 H-cyclopropyl, H-4′b, H-7b, 5′-CH_3, 10-CH_3), 1.13 (d, *J* = 6.8 Hz, 3 H, 2-CH_3), 1.06 (d, *J* = 7.5 Hz, 3 H, 4-CH_3), 0.93 (d, *J* = 7.0 Hz, 3 H, 8-CH_3), 0.87 (t, *J* = 7.3 Hz, 3 H, 15-CH_3). ^{13}C NMR ($CDCl_3$, 100 MHz) δ: 178.06, 174.10, 166.66, 164.59, 156.45, 154.69, 148.15, 139.98, 136.61, 129.75, 125.89, 122.63, 117.37, 108.98, 103.10, 92.81, 85.06, 83.20, 81.18, 79.28, 78.33, 78.14, 77.25, 75.47, 70.44, 69.33, 66.10, 61.40, 49.84, 43.35, 41.01, 40.35, 37.33, 35.96, 35.44, 32.73, 29.77, 29.03, 28.10, 26.02, 25.61, 22.21, 21.15, 19.38, 19.32, 18.86, 15.60, 14.89, 13.00, 10.17, 8.92, 8.32.

3-O-Descladinosyl-3-O-[N-7-(1-cyclopropyl-1,4-dihydro-4-oxo-3-quinolinecarboxylic acid-7-yl)-hept-6-ynylcarbamoyl]-6-O-methylerythromycin A 9-O- methyl oxime 11,12- cyclic carbonate ***(57c)***

Following the general procedure I-1, from compound **55** (0.300 g, 0.364 mmol) and **4** (0.129 g, 0.364 mmol), **57c** (24.8 mg, 0.246 mmol, 6.75 %) was obtained as an off-white solid (column chromatography eluents: DCM/MeOH/NH_3·H_2O = 10:0.8:0.5). HRMS (ESI) $(M+H)^+$ *m/z* 1009.5381, calcd for $C_{53}H_{77}N_4O_{15}$ 1009.5380. HPLC purity: 98.2% (t= 12.1 min). ^1H NMR ($CDCl_3$, 400 MHz) δ: 8.86 (s, 1 H, 2″-quinolyl), 8.40 (d, *J* = 8.3 Hz, 1 H, 5″-quinolyl), 8.06 (d, *J* = 1.4 Hz, 1 H, 8″-quinolyl), 7.54 (dd, *J* = 1.4 Hz, 8.3 Hz, 1 H, 6″-quinolyl), 5.16 (dd, *J* = 2.5 Hz, 10.8 Hz, 1 H, H-13), 5.07 (br, 1 H, 3-*O*-CO-N**H**-CH_2), 4.91-4.79 (m, 2 H, H-3, H-11), 4.03 (d, *J* = 7.3 Hz, 1 H, H-1′), 3.82 (s, 3 H, 9-*O*-CH_3), 3.73 (d, *J* = 3.0 Hz, 1 H, H-5), 3.72-3.64 (m, 1 H, H-8), 3.63-3.56 (m, 1 H, 1 H-cyclopropyl), 3.44-3.31 (m, 2 H, H-5′, 3-*O*-CO-NH-C**H**$_2$), 3.21 (dd, *J* = 7.2 Hz, 10.1 Hz, 1 H, H-2′), 3.15-3.05 (m, 1 H, 3-*O*-CO-NH-C**H**$_2$), 3.01 (s, 3 H, 6-*O*-CH_3), 2.87-2.76 (m, 1 H, H-2), 2.56-2.43 (m, 4 H, H-3′, H-10, -C**H**$_2$-C≡C-quinolyl), 2.33 (s, 6 H, -N(CH_3)$_2$), 2.12-2.02 (m, 1 H, H-4), 1.95-1.83 (m, 1 H, H-14eq), 1.75-1.50 (m, 8 H, 3(-CH_2-), H-4′a, H-14ax), 1.49 (s, 3 H, 12-CH_3), 1.46-1.35 (m, 3 H, 2 H-cyclopropyl, H-4′b), 1.32 (s, 3 H, 6-CH_3), 1.30-1.17 (m, 10 H, 2 H-cyclopropyl, H-7a, H-7b, 5′-CH_3, 10-CH_3), 1.12 (d, *J* = 6.8 Hz, 3 H, 2-CH_3), 1.06 (d, *J* = 7.4 Hz, 3 H, 4-CH_3), 0.93 (d, *J* = 7.0 Hz, 3 H, 8-CH_3), 0.84 (t, *J* = 7.4 Hz, 3 H, 15-CH_3). ^{13}C NMR ($CDCl_3$, 100 MHz) δ: 178.19,

174.04, 166.74, 164.55, 156.48, 154.66, 148.48, 141.07, 130.03, 129.40, 126.88, 124.86, 119.83, 109.02, 103.21, 95.50, 85.02, 83.19, 81.28, 79.99, 78.34, 78.21, 77.24, 75.52, 70.47, 69.37, 66.07, 61.42, 49.82, 43.35, 40.94, 40.35, 37.34, 35.95, 35.37, 32.73, 29.78, 28.92, 28.04, 26.08, 25.60, 22.22, 21.17, 19.54, 19.32, 18.86, 15.60, 14.87, 13.01, 10.17, 8.92, 8.36.

3-O-Descladinosyl-3-O-[N-7-(1-cyclopropyl-1,4-dihydro-4-oxo-3-quinolinecarboxamide-7-yl)-hept-6-ynylcarbamoyl]-6-O-methylerythromycin A 9-O- methyl oxime 11,12- cyclic carbonate ***(57d)***

Following the general procedure I-2, from compound **55** (0.250 g, 0.303 mmol) and **5** (0.129 g, 0.364 mmol), **57c** (0.150 g, 0.149 mmol, 49.2 %) was obtained as an off-white solid (column chromatography eluents: DCM/MeOH/$NH_3 \cdot H_2O$ = 10:0.3:0.1).

3-O-Descladinosyl-3-O-[N-7-(1-cyclopropyl-1,4-dihydro-N-hydroxy-4-oxo-3-quinolinecarboxamide-7-yl)-hept-6-ynylcarbamoyl]-6-O-methylerythromycin A 9-O- methyl oxime 11,12- cyclic carbonate ***(57e)***

Following the general procedure I-1, from compound **55** (0.311 g, 0.378 mmol) and **6** (0.210 g, 0.567 mmol), **53e** (40.4 mg, 0.0394 mmol, 10.4 %) was obtained as an off-white solid (column chromatography eluents: DCM/MeOH/$NH_3 \cdot H_2O$ = 10:0.5:0.3). m.p. 139.7-140.4 ˚C. HRMS (ESI) $(M+H)^+$ *m/z* 1124.5524, calcd for $C_{53}H_{78}N_5O_{15}$ 1024.5489. ^{1}H NMR ($CDCl_3$, 400 MHz) δ: 12.05 (s, 1 H, N**H**OH), 8.71 (s, 1 H, 2″-quinolyl), 8.27 (d, *J* = 8.3 Hz, 1 H, 5″-quinolyl), 7.91 (d, *J* = 1.4 Hz, 1 H, 8″-quinolyl), 7.38 (dd, *J* = 1.4 Hz, 8.3 Hz, 1 H, 6″-quinolyl), 5.26 (br, 1 H, 3-*O*-CO-N**H**-CH_2), 5.08 (dd, *J* = 2.5 Hz, 10.7 Hz, 1 H, H-13), 4.83-4.73 (m, 2 H, H-3, H-11), 3.97 (d, *J* = 7.2 Hz, 1 H, H-1′), 3.74 (s, 3 H, 9-*O*-CH_3), 3.64 (d, *J* = 2.9 Hz, 1 H, H-5), 3.63-3.56 (m, 1 H, H-8), 3.63-3.56 (tt, *J* = 4.0 Hz, 7.2 Hz, 1 H, 1 H-cyclopropyl), 3.37-3.42 (m, 2 H, H-5′, 3-*O*-CO-NH-C**H**$_2$), 3.16 (dd, *J* = 7.2 Hz, 10.1 Hz, 1 H, H-2′), 3.07-2.96 (m, 1H, 3-*O*-CO-NH-C**H**$_2$), 2.94 (s, 3 H, 6-*O*-CH_3), 2.78-2.66 (m, 1 H, H-2), 2.61-2.48 (m, 1 H, H-3′), 2.46-2.35 (m, 3 H, H-10, -C**H**$_2$-C≡C-quinolyl), 2.30 (s, 6 H, -N(CH_3)$_2$), 2.15-1.96 (m, 1 H, H-4), 1.88-1.75 (m, 1 H, H-14eq), 1.67-1.42 (m, 8 H, 3(-CH_2-), H-4′a, H-14ax), 1.41 (s, 3 H, 12-CH_3), 1.36-1.26 (m, 3 H, 2 H-cyclopropyl, H-4′b), 1.25 (s, 3 H, 6-CH_3), 1.21-1.07 (m, 10 H, 2 H-cyclopropyl, H-7a, H-7b, 5′-CH_3, 10-CH_3), 1.04 (d, *J* = 6.7 Hz, 3 H, 2-CH_3), 0.99 (d, *J* = 7.4 Hz, 3 H, 4-CH_3), 0.86 (d, *J* = 7.0 Hz, 3 H, 8-CH_3), 0.76 (t, *J* = 7.3 Hz, 3 H, 15-CH_3). ^{13}C NMR ($CDCl_3$, 100 MHz) δ: 175.27, 174.05, 164.54, 162.51, 156.53, 154.68, 146.72, 140.53, 128.90, 128.52, 127.00, 125.86, 119.49, 110.40, 103.27, 94.48, 85.04, 83.21, 81.69, 80.18, 78.31, 78.16, 77.25, 75.49, 70.51, 69.20, 65.85, 61.43, 49.81, 43.36, 40.95, 40.15, 37.35, 35.93, 34.87, 32.73, 29.75, 29.02, 28.08, 26.07, 25.59, 22.22, 21.17, 19.50, 19.32, 18.86, 15.61, 14.87, 13.01, 10.18, 8.97, 8.36.

3-O-Descladinosyl-3-O-[N-8-(1-cyclopropyl-1,4-dihydro-4-oxo-3-quinolinecarboxylic acid-7-yl]-oct-7-ynylcarbamoyl)-6-O-methylerythromycin A 9-O- methyl oxime 11,12- cyclic carbonate ***(58c)***

Following the general procedure I-1, from compound **56** (0.421 g, 0.502 mmol) and **4** (0.214 g, 0.602 mmol), **58c** (63.0 mg, 0.0616 mmol, 12.3%) was obtained as an off-white solid (column chromatography eluents: DCM/MeOH/$NH_3 \cdot H_2O$ = 10:0.9:0.5). m.p. 158.1-159.6 ˚C. HRMS (ESI) $(M+H)^+$ *m/z* 1023.5521, calcd for $C_{54}H_{79}N_4O_{15}$ 1023.5536. ^{1}H NMR ($CDCl_3$, 400 MHz) δ: 8.86 (s, 1 H, 2″-quinolyl), 8.40 (d, *J* = 8.4 Hz, 1 H, 5″-quinolyl), 8.06 (d, *J* = 1.4 Hz, 1 H, 8″-quinolyl), 7.54 (dd, *J* = 1.4 Hz, 8.4 Hz, 1 H, 6″-quinolyl), 5.16 (dd, *J* = 2.5 Hz, 10.8 Hz, 1 H, H-13), 4.96 (t, *J* = 5.8 Hz, 1 H, 3-*O*-CO-N**H**-CH_2), 4.90-4.82 (m, 2 H, H-3, H-11), 4.10 (d, *J* = 7.3 Hz, 1 H, H-1′), 3.82 (s, 3 H, 9-*O*-CH_3), 3.74 (d, *J* = 3.1 Hz, 1 H, H-5), 3.72-3.65 (m, 1 H, H-8), 3.59 (tt, *J* = 4.0 Hz, 7.2 Hz, 1 H, 1 H-cyclopropyl), 3.42-3.29 (m, 2 H, H-5′, 3-*O*-CO-NH-C**H**$_2$), 3.18 (dd, *J* = 7.3 Hz, 10.1 Hz, 1 H, H-2′), 3.13-3.04 (m, 1H, 3-*O*-CO-NH-C**H**$_2$), 3.02 (s, 3 H, 6-*O*-CH_3), 2.89-2.80 (m, 1 H, H-2), 2.54-2.44 (m, 3 H, H-10, -C**H**$_2$-C≡C-quinolyl), 2.42-2.34 (m, 1 H, H-3′), 2.27 (s, 6 H, -N(CH_3)$_2$), 2.12-2.04 (m, 1 H, H-4), 1.96-1.84 (m, 1 H, H-14eq), 1.73-1.50 (m, 8 H, 3(-CH_2-), H-4′a, H-14ax), 1.49 (s, 3 H, 12-CH_3), 1.47-1.36 (m, 5 H, 2 H-cyclopropyl, H-4′b, CH_2), 1.32 (s, 3 H, 6-CH_3), 1.27-1.18 (m, 10 H, 2 H-cyclopropyl, H-7a, H-7b, 5′-CH_3, 10-CH_3), 1.14 (d, *J* = 6.8 Hz, 3 H, 2-CH_3), 1.07 (d, *J* = 7.5 Hz, 3 H, 4-CH_3), 0.93 (d, *J* = 7.0 Hz, 3 H, 8-CH_3), 0.86 (t, *J* = 7.3 Hz, 3 H, 15-CH_3). ^{13}C NMR ($CDCl_3$, 175 MHz) δ: 178.19, 174.06, 166.76, 164.57, 156.42, 154.68, 148.48, 141.06, 130.11, 129.42, 126.87, 124.82, 119.80, 108.99, 103.33, 95.79, 85.03, 83.21, 81.21, 79.85, 78.34, 78.20, 75.49, 70.45, 69.49, 66.09, 61.42, 49.82, 43.35, 41.07, 40.37, 37.32, 35.96, 35.36, 32.71, 30.10, 28.64, 28.58, 28.33, 26.32, 25.60, 22.22,

21.21, 19.54, 19.34, 18.86, 15.60, 14.89, 13.02, 10.19, 8.91, 8.35.

General procedure J

To a solution of **54** (1 eq) and **37-38**, **47-50** (1.8 eq) in dry DMF, DBU (2 eq) was added. The reaction mixture was stirred at room temperature for 12 h. After completion of the reaction, CH_2Cl_2 (50 mL) and water (50 mL) were added into the reaction mixture. The organic layer was washed with water and brine, and concentrated in vacuo. The product was then dissolved in MeOH (15 mL), and the solution was for stirred at 65 ˚C for 2 h. The organic solvent was removed in vacuo. The crude mixture was purified by column chromatography on silica gel.

3-O-Descladinosyl-3-O-[N-5-(1-cyclopropyl-1,4-dihydro-4-oxo-3-quinolinecarboxamide-6-yl)pentylcarbamoyl]-6-O-methylerythromycin A 9-O- methyl oxime 11,12- cyclic carbonate ***(59b)***

Following the general procedure J, from compound **54** (0.250 g, 0.320 mmol) and **47** (0.180 g, 0.570 mmol), **59c** (19.3 mg, 0.0190 mmol, 5.95%) was obtained as an off-white solid (column chromatography eluents: DCM/MeOH/NH_3·H_2O = 10:0.1:0.1). HRMS (ESI) $(M+H)^+$ *m/z* 984.5543, Calcd for $C_{51}H_{78}N_5O_{14}$ 984.5540. ^{1}H NMR ($CDCl_3$, 400 MHz), δ: 9.73 (s, 1 H, N**H**$_2$-quinolyl), 8.87 (s, 1 H, 2″-quinolyl), 8.30 (s, 1 H, 5″-quinolyl), 8.05 (d, *J* = 8.7 Hz, 1 H, 8″-quinolyl), 7.70 (d, *J* = 8.7 Hz, 1 H, 7″-quinolyl), 5.88 (s, 1 H, N**H**$_2$-quinolyl), 5.60 (s, 1 H, 3-*O*-CON**H**-), 5.20 (dd, *J* = 2.4 Hz, 10.8 Hz, 1 H, H-13), 4.91-4.81 (m, 2 H, H-3, H-11), 4.01 (d, *J* = 7.4 Hz, 1 H, H-1′), 3.82 (s, 3 H, 9-*O*-C**H**$_3$), 3.75-3.58 (m, 2 H, H-5, H-8), 3.58-3.49 (m, 1 H, H-cyclopropyl), 3.49-3.38 (m, 1 H, H-5′), 3.38-3.25 (m, 2 H, H-2′, 3-*O*-CONH-C**H**$_2$), 3.20-3.10 (m, 1 H, 3-*O*-CONH-C**H**$_2$), 3.02 (s, 3 H, 6-*O*-C**H**$_3$), 2.91-2.81 (m, 3 H, H-2, C**H**$_2$-quinolyl), 2.61 (s, 6 H, -N(CH_3)$_2$), 2.53-2.45 (m, 2 H, H-10, H-3′), 2.12-2.02 (m, 1 H, H-4), 1.99-1.83 (m, 2 H, H-14eq, H-4′a), 1.80-1.65 (m, 2 H, CH_2), 1.65-1.55 (m, 3 H, H-14ax, CH_2), 1.49 (s, 3 H, 12-CH_3), 1.44-1.36 (m, 6 H, H-7a, 2H-cyclopropyl, CH_2, H-4′b), 1.32 (s, 3 H, 6-CH_3), 1.28-1.19 (m, 9 H, 2 H-cyclopropyl, H-7b, 10-CH_3, 5′-CH_3), 1.18-1.05 (m, 6 H, 2-CH_3, 4-CH_3), 0.93 (d, *J* = 7.0 Hz, 3 H, 8-CH_3), 0.86 (t, *J* = 7.5 Hz, 3 H, 15-CH_3). ^{13}C NMR ($CDCl_3$, 100 MHz), δ: 176.62, 174.09, 166.88, 164.46, 156.47, 154.67, 147.39, 139.92, 139.18, 133.60, 127.42, 125.85, 116.93, 111.29, 102.26, 85.05, 83.18, 81.16, 78.20, 77.80, 77.25, 75.47, 70.28, 68.64, 65.97, 61.44, 53.45, 49.88, 43.31, 40.94, 40.25, 37.30, 35.88, 35.23, 34.81, 32.74, 30.79, 30.30, 30.07, 29.70, 26.19, 25.56, 22.23, 20.98, 19.30, 18.89, 15.62, 14.82, 13.04, 10.21, 9.05, 8.20.

3-O-Descladinosyl-3-O-[N-6-(1-cyclopropyl-1,4-dihydro-4-oxo-3-quinolinecarboxamide-6-yl)hexylcarbamoyl]-6-O-methylerythromycin A 9-O- methyl oxime 11,12- cyclic carbonate ***(60b)***

Following the general procedure J, from compound **54** (0.200 g, 0.260 mmol) and **48** (0.130 g, 0.400 mmol), **60b** (17.2 mg, 0.0170 mmol, 6.56%) was obtained as an off-white solid (column chromatography eluents: DCM/MeOH/NH_3·H_2O = 10:0.1:0.1). HRMS (ESI) $(M+H)^+$ *m/z* 998.5695, Calcd for $C_{52}H_{80}N_5O_{14}$ 998.5696. ^{1}H NMR ($CDCl_3$, 400 MHz), δ: 9.73 (s, 1 H, N**H**$_2$-quinolyl), 8.89 (s, 1 H, 2″-quinolyl), 8.30 (s, 1 H, 5″-quinolyl), 8.05 (d, *J* = 8.7 Hz, 1 H, 8″-quinolyl), 7.70 (d, *J* = 8.7 Hz, 1 H, 7″-quinolyl), 5.85 (s, 1 H, N**H**$_2$-quinolyl), 5.51 (s, 1 H, 3-*O*-CON**H**-), 5.16 (dd, *J* = 2.4 Hz, 10.8 Hz, 1 H, H-13), 4.91-4.81 (m, 2 H, H-3, H-11), 4.01 (d, *J* = 7.4 Hz, 1 H, H-1′), 3.82 (s, 3 H, 9-*O*-C**H**$_3$), 3.75-3.58 (m, 3 H, H-5, H-8, H-cyclopropyl), 3.42-3.09 (m, 2 H, H-5′, H-2′, 3-*O*-CONH-C**H**$_2$), 3.10-3.05 (m, 1 H, 3-*O*-CONH-C**H**$_2$), 3.01 (s, 3 H, 6-*O*-C**H**$_3$), 2.91-2.81 (m, 3 H, H-2, CH_2-quinolyl), 2.57 (s, 6 H, -N(CH_3)$_2$), 2.52-2.40 (m, 2 H, H-10, H-3′), 2.12-2.02 (m, 2 H, H-4, CH_2), 1.99-1.83 (m, 2 H, H-14eq, CH_2), 1.65-1.55 (m, 4 H, H-4′a, H-14ax, CH_2), 1.49 (s, 3 H, 12-CH_3), 1.44-1.36 (m, 6 H, H-7a, H-cyclopropyl, 2(CH_2)), 1.32 (s, 3 H, 6-CH_3), 1.28-1.10 (m, 13 H, 2H-cyclopropyl, H-7b, H-4′a, 10-CH_3, 5′-CH_3, 2-CH_3), 1.08 (d, *J* = 7.6 Hz, 3 H, 4-CH_3), 0.93 (d, *J* = 7.0 Hz, 3 H, 8-CH_3), 0.86 (t, *J* = 7.5 Hz, 3 H, 15-CH_3). ^{13}C NMR ($CDCl_3$, 100 MHz), δ: 176.65, 174.10, 166.88, 164.48, 156.46, 154.68, 147.39, 140.15, 139.16, 133.61, 127.44, 125.89, 116.87, 111.29, 102.39, 85.05, 83.19, 81.19, 78.22, 77.85, 77.25, 75.47, 70.29, 68.71, 66.00, 61.44, 49.87, 43.35, 41.08, 40.26, 37.30, 35.91, 35.22, 34.81, 32.74, 31.09, 30.11, 29.71, 28.71, 26.58, 25.56, 22.23, 20.97, 19.31, 18.89, 15.62, 14.88, 13.04, 10.19, 9.06, 8.20.

3-O-Descladinosyl-3-O-[N-7-(1-cyclopropyl-1,4-dihydro-4-oxo-3-quinolinecarboxylic acid-6-yl)hepylcarbamoyl]-6-O-methylerythromycin A 9-O- methyl oxime 11,12- cyclic carbonate ***(61a)***

According to the general procedure E, from compound **57a** (0.0768 g, 0.0731 mmol), **61a** (24.9 mg, 0.246 mmol,

33.6%) was obtained as a white solid (column chromatography eluents: DCM/EtOH/$NH_3{\cdot}H_2O$ = 10:0.8:0.5). HRMS (ESI) $(M+H)^+$ *m/z* 1013.5671, calcd for $C_{53}H_{81}N_4O_{15}$ 1013.5693. ^1H NMR ($CDCl_3$, 400 MHz) δ: 8.86 (s, 1 H, 2″-quinolyl), 8.28 (s, 1 H, 5″-quinolyl), 8.05 (s, 1 H, 8″-quinolyl), 7.68 (s, 1 H, 7″-quinolyl), 5.49 (br, 1 H, 3-*O*-CO-N**H**-CH_2), 5.16 (d, *J* = 10.4 Hz, 1 H, H-13), 4.92-4.77 (m, 2 H, H-3, H-11), 4.15 (d, *J* = 6.6 Hz, 1 H, H-1′), 3.82 (s, 3 H, 9-*O*-CH_3), 3.76-3.60 (m, 3 H, H-5, H-8, 1 H-cyclopropyl), 3.52-3.36 (m, 1 H, H-5′), 3.37-3.24 (m, 2 H, 3-*O*-CO-NH-C**H**$_2$, H-2′), 3.12-3.04 (m, 1 H, 3-*O*-CO-NH-C**H**$_2$), 3.01 (s, 3 H, 6-*O*-CH_3), 2.94-2.73 (m, 3 H, H-2, -C**H**$_2$-quinolyl), 2.65-2.39 (m, 8 H, H-10, H-3′, -$N(CH_3)_2$), 2.13-2.00 (m, 1 H, H-4), 1.96-1.82 (m, 1 H, H-14eq), 1.75-1.63 (m, 2 H, -C**H**$_2$-CH_2-quinolyl), 1.63-1.51 (m, 4 H, CH_2, H-4′a, H-14ax), 1.49 (s, 3 H, 12-CH_3), 1.42-1.16 (m, 22 H, 2 H-cyclopropyl, H-7a, 3(-CH_2-), 6-CH_3, H-4′b, H-7b, 5′-CH_3, 10-CH_3, 2 H-cyclopropyl), 1.13 (d, *J* = 6.4 Hz, 3 H, 2-CH_3), 1.08 (br, 3 H, 4-CH_3), 0.93 (d, *J* = 7.1 Hz, 3 H, 8-CH_3), 0.85 (t, *J* = 7.1 Hz, 3 H, 15-CH_3). ^{13}C NMR ($CDCl_3$, 175 MHz) δ: 178.62, 174.17, 164.59, 156.51, 154.70, 147.67, 141.59, 139.38, 134.90, 125.88, 125.57, 117.34, 108.42, 102.40, 85.10, 83.18, 81.04, 78.24, 78.21, 77.79, 75.43, 68.75, 61.41, 49.87, 43.31, 41.08, 40.29, 37.28, 36.04, 35.91, 35.45, 35.25, 34.89, 32.72, 30.97, 30.17, 29.70, 29.65, 29.01, 28.95, 26.63, 25.58, 22.21, 21.02, 19.30, 18.90, 18.88, 15.61, 14.99, 14.87, 14.13, 13.02, 12.99, 10.22, 10.20, 9.13, 8.25, 8.13.

3-O-Descladinosyl-3-O-[N-7-(1-cyclopropyl-1,4-dihydro-4-oxo-3-quinolinecarboxamide-6-yl)hepylcarbamoyl]-6-O-methylerythromycin A 9-O- methyl oxime 11,12- cyclic carbonate ***(61b)***

Following the general procedure J, from compound **54** (0.209 g, 0.270 mmol) and **37** (0.180 g, 0.530 mmol), **61b** (54.6 mg, 0.053 mmol, 19.6%) was obtained as an off-white solid (column chromatography eluents: DCM/MeOH/$NH_3{\cdot}H_2O$ = 10:0.1:0.1). HRMS (ESI) $(M+H)^+$ *m/z* 1012.5858, Calcd for $C_{53}H_{82}N_5O_{14}$ 1012.5853. ^1H NMR ($CDCl_3$, 400 MHz), δ: 9.73 (d, *J* = 4.8 Hz, 1 H, N**H**$_2$-quinolyl), 8.87 (s, 1 H, 2″-quinolyl), 8.30 (s, 1 H, 5″-quinolyl), 8.05 (d, *J* = 8.7 Hz, 1 H, 8″-quinolyl), 7.70 (d, *J* = 8.7 Hz, 1 H, 7″-quinolyl), 5.85 (d, *J* = 4.8 Hz, 1 H, N**H**$_2$-quinolyl), 5.25-5.13 (m, 2 H, H-13, 3-*O*-CONH-), 4.91-4.81 (m, 2 H, H-3, H-11), 4.01 (d, *J* = 7.4 Hz, 1 H, H-1′), 3.82 (s, 3 H, 9-*O*-C**H**$_3$), 3.75-3.65 (m, 2 H, H-5, H-8), 3.58-3.49 (m, 1 H, H-cyclopropyl), 3.42-3.26 (m, 2 H, H-5′, 3-*O*-CONH-C**H**$_2$), 5.16 (dd, *J* = 7.6 Hz, 10.0 Hz, 1 H, H-2′), 3.02 (s, 3 H, 6-*O*-C**H**$_3$), 2.91-2.81 (m, 3 H, H-2, CH_2-quinolyl), 2.52-2.38 (m, 2 H, H-10, H-3′), 2.36 (s, 6 H, -$N(CH_3)_2$), 2.12-2.02 (m, 1 H, H-4), 1.99-1.83 (m, 1 H, H-14eq), 1.75-1.62 (m, 3 H, H-4′a, CH_2), 1.65-1.55 (m, 3 H, H-14ax, CH_2), 1.49 (s, 3 H, 12-CH_3), 1.44-1.36 (m, 9 H, H-7a, 3(CH_2), 2H-cyclopropyl), 1.32 (s, 3 H, 6-CH_3), 1.28-1.19 (m, 10 H, 2H-cyclopropyl, H-7b, H-4′b, 10-CH_3, 5′-CH_3), 1.13 (d, *J* = 6.8 Hz, 3 H, 2-CH_3), 1.08 (d, *J* = 7.6 Hz, 3 H, 4-CH_3), 0.93 (d, *J* = 7.0 Hz, 3 H, 8-CH_3), 0.86 (t, *J* = 7.5 Hz, 3 H, 15-CH_3). ^{13}C NMR ($CDCl_3$, 100 MHz), δ: 176.66, 174.09, 166.89, 164.59, 156.41, 154.70, 147.34, 140.24, 139.13, 133.59, 127.45, 125.90, 116.79, 111.30, 103.00, 85.06, 83.21, 81.17, 78.29, 78.03, 77.27, 75.44, 70.41, 69.24, 66.05, 61.42, 49.84, 43.36, 41.15, 40.32, 37.32, 35.94, 35.27, 34.78, 32.72, 31.08, 30.12, 29.05, 28.95, 26.66, 25.60, 22.22, 21.13, 19.33, 18.87, 15.61, 14.89, 13.02, 10.19, 8.97, 8.18.

3-O-Descladinosyl-3-O-[N-7-(1-cyclopropyl-1,4-dihydro-4-oxo-3-quinolinecarboxylic acid-7-yl)hepylcarbamoyl]-6-O-methylerythromycin A 9-O- methyl oxime 11,12- cyclic carbonate ***(61c)***

According to the general procedure E, from compound **57c** (0.124 g, 0.118 mmol), **61c** (58.2 mg, 0.574 mmol, 48.7%) was obtained as a white solid (column chromatography eluents: DCM/EtOH/$NH_3{\cdot}H_2O$ = 10:0.8:0.5). HRMS (ESI) $(M+H)^+$ *m/z* 1013.5681, calcd for $C_{53}H_{81}N_4O_{15}$ 1013.5693. ^1H NMR ($CDCl_3$, 400 MHz) δ: 8.84 (s, 1 H, 2″-quinolyl), 8.39 (d, *J* = 8.2 Hz, 1 H, 5″-quinolyl), 7.85 (s, 1 H, 8″-quinolyl), 7.42 (dd, *J* = 8.2 Hz, 1 H, 7″-quinolyl), 5.25 (br, 1 H, 3-*O*-CO-N**H**-CH_2), 5.16 (dd, *J* = 2.5 Hz, 10.7 Hz, 1 H, H-13), 4.92-4.75 (m, 2 H, H-3, H-11), 4.07 (br, 1 H, H-1′), 3.82 (s, 3 H, 9-*O*-CH_3), 3.75-3.59 (m, 3 H, H-5, H-8, 1 H-cyclopropyl), 3.54-3.17 (m, 3 H, H-5′, 3-*O*-CO-NH-C**H**$_2$, H-2′), 3.12-3.04 (m, 1 H, 3-*O*-CO-NH-C**H**$_2$), 3.02 (s, 3 H, 6-*O*-CH_3), 2.93-2.55 (m, 4 H, H-2, -C**H**$_2$-quinolyl, H-3′), 2.53-2.28 (m, 7 H, H-10, -$N(CH_3)_2$), 2.14-2.00 (m, 1 H, H-4), 1.97-1.81 (m, 1 H, H-14eq), 1.81-1.64 (m, 2 H, -C**H**$_2$-CH_2-quinolyl), 1.63-1.51 (m, 4 H, CH_2, H-4′a, H-14ax), 1.49 (s, 3 H, 12-CH_3), 1.45-1.17 (m, 22 H, 2 H-cyclopropyl, H-7a, 3(CH_2), 6-CH_3, H-4′b, H-7b, 5′-CH_3, 10-CH_3, 2 H-cyclopropyl), 1.13 (d, *J* = 6.6 Hz, 3 H, 2-CH_3), 1.08 (br, 3 H, 4-CH_3), 0.93 (d, *J* = 7.0 Hz, 3 H, 8-CH_3), 0.86 (t, *J* = 7.3 Hz, 3 H, 15-CH_3). ^{13}C NMR ($CDCl_3$, 175 MHz) δ: 178.47, 174.14, 167.16, 164.61, 156.51, 154.69, 150.08, 148.00, 141.32, 127.28, 126.82, 126.80, 123.99,

116.38, 108.43, 102.76, 85.13, 85.08, 83.19, 81.07, 78.28, 77.92, 75.45, 61.41, 49.84, 43.31, 41.00, 40.30, 37.29, 36.58, 35.92, 35.38, 32.71, 31.04, 30.16, 29.70, 29.65, 29.19, 29.08, 26.64, 25.59, 22.21, 21.11, 19.31, 18.90, 18.86, 15.60, 14.89, 14.86, 13.01, 10.20, 8.98, 8.34, 8.32.

3-O-Descladinosyl-3-O-[N-7-(1-cyclopropyl-1,4-dihydro-4-oxo-3-quinolinecarboxamide-7-yl)hepylcarbamoyl]-6-O-methylerythromycin A 9-O- methyl oxime 11,12- cyclic carbonate ***(61d)***

According to the general procedure E, from compound **57d** (0.150 g, 0.149 mmol), **61d** (69.6 mg, 0.0688 mmol, 46.2%) was obtained as a white solid (column chromatography eluents: DCM/EtOH/NH_3·H_2O = 10:0.3:0.1). HRMS (ESI) $(M+H)^+$ *m/z* 1012.5869, calcd for $C_{53}H_{82}N_5O_{14}$ 1012.5853. ^{1}H NMR ($CDCl_3$, 400 MHz) δ: 9.73 (d, *J* = 5.2 Hz, 1 H, N**H**$_2$), 8.88 (s, 1 H, 2″-quinolyl), 8.40 (d, *J* = 8.2 Hz, 1 H, 5″-quinolyl), 7.76 (d, *J* = 1.4 Hz, 1 H, 8″-quinolyl), 7.33 (dd, *J* = 1.4 Hz, 8.2 Hz, 1 H, 6″-quinolyl), 5.80 (d, *J* = 5.2 Hz, 1 H, N**H**$_2$), 5.17 (dd, *J* = 2.5 Hz, 10.7 Hz, 1 H, H-13), 4.97 (t, *J* = 5.8 Hz, 1 H, 3-*O*-CO-N**H**-CH_2), 4.90-4.82 (m, 2 H, H-3, H-11), 4.01 (d, *J* = 7.3 Hz, 1 H, H-1′), 3.82 (s, 3 H, 9-*O*-CH_3), 3.75 (d, *J* = 3.1 Hz, 1 H, H-5), 3.73-3.65 (m, 1 H, H-8), 3.52 (tt, *J* = 4.0 Hz, 7.2 Hz, 1 H, 1 H-cyclopropyl), 3.44-3.24 (m, 2 H, H-5′, 3-*O*-CO-NH-C**H**$_2$), 3.19 (dd, *J* = 7.3 Hz, 10.1 Hz, 1 H, H-2′), 3.11-3.04 (m, 1 H, 3-*O*-CO-NH-C**H**$_2$), 3.02 (s, 3 H, 6-*O*-CH_3), 2.90-2.76 (m, 3 H, H-2, -C**H**$_2$-quinolyl), 2.49 (d, *J* = 6.8 Hz, 1 H, H-10), 2.43-2.34 (m, 1 H, H-3′), 2.28 (s, 6 H, -N(CH_3)$_2$), 2.12-2.03 (m, 1 H, H-4), 1.96-1.84 (m, 1 H, H-14eq), 1.76-1.65 (m, 2 H, H-2, -C**H**$_2$-CH_2-quinolyl), 1.65-1.51 (m, 4 H, CH_2, H-4′a, H-14ax), 1.49 (s, 3 H, 12-CH_3), 1.44-1.33 (m, 9 H, 2 H-cyclopropyl, H-7a, 3(-CH_2-)), 1.32 (s, 3 H, 6-CH_3), 1.28-1.20 (m, 8 H, H-4′b, H-7b, 5′-CH_3, 10-CH_3), 1.19-1.15 (m, 2 H, 2 H-cyclopropyl), 1.13 (d, *J* = 6.9 Hz, 3 H, 2-CH_3), 1.07 (d, *J* = 7.4 Hz, 3 H, 4-CH_3), 0.93 (d, *J* = 7.0 Hz, 3 H, 8-CH_3), 0.86 (t, *J* = 7.4 Hz, 3 H, 15-CH_3). ^{13}C NMR ($CDCl_3$, 100 MHz) δ: 176.54, 174.05, 166.82, 164.58, 156.39, 154.67, 148.44, 147.61, 141.07, 127.16, 126.14, 125.68, 115.82, 111.46, 103.30, 85.03, 83.21, 81.26, 78.34, 78.20, 77.26, 75.50, 70.47, 69.45, 66.10, 61.41, 49.81, 43.37, 41.10, 40.36, 37.35, 36.47, 35.96, 34.67, 32.73, 31.13, 30.17, 29.22, 29.14, 28.66, 26.70, 25.61, 22.23, 21.19, 19.33, 18.86, 15.60, 14.87, 13.01, 10.18, 8.92, 8.26.

3-O-Descladinosyl-3-O-[N-8-(1-cyclopropyl-1,4-dihydro-4-oxo-3-quinolinecarboxamide-6-yl)octylcarbamoyl]-6-O-methylerythromycin A 9-O- methyl oxime 11,12- cyclic carbonate ***(62b)***

Following the general procedure J, from compound **54** and **38**, **61b** was obtained as an off-white solid (column chromatography eluents: DCM/MeOH/NH_3·H_2O = 10:0.2:0.1). HRMS (ESI) $(M+H)^+$ *m/z* 1026.6012, Calcd for $C_{54}H_{84}N_5O_{14}$ 1026.6009. ^{1}H NMR ($CDCl_3$, 400 MHz), δ: 9.74 (d, *J* = 5.2 Hz, 1 H, N**H**$_2$-quinolyl), 8.89 (s, 1 H, 2″-quinolyl), 8.29 (d, *J* = 1.4 Hz, 1 H, 5″-quinolyl), 7.94 (d, *J* = 8.7 Hz, 1 H, 8″-quinolyl), 7.59 (d, *J* = 2.5 Hz, 8.7 Hz, 1 H, 7″-quinolyl), 5.84 (s, 1 H, N**H**$_2$-quinolyl), 5.44 (s, 1 H, 3-*O*-CON**H**-), 5.17 (dd, *J* = 2.5 Hz, 10.8 Hz, 1 H, H-13), 4.92-4.79 (m, 2 H, H-3, H-11), 4.11 (d, *J* = 7.3 Hz, 1 H, H-1′), 3.83 (s, 3 H, 9-*O*-C**H**$_3$), 3.75 (d, *J* = 3.0 Hz, 1 H, H-5) 3.71-3.62 (m, 1 H, H-8), 3.59-3.49 (m, 1 H, H-cyclopropyl), 3.48-3.38 (m, 1 H, H-5′), 3.38-3.20 (m, 2 H, H-2′, 3-*O*-CONHC**H**$_2$-), 3.16-3.06 (m, 1 H, 3-*O*-CONHC**H**$_2$-), 3.00 (s, 3 H, 6-*O*-C**H**$_3$), 2.87-2.70 (m, 3 H, H-2, C**H**$_2$-quinolyl), 2.62 (s, 6 H, -N(CH_3)$_2$), 2.56-2.40 (m, 2 H, H-10, H-3′), 2.15-2.00 (m, 1 H, H-4), 1.99-1.83 (m, 2 H, H-14eq, H-4′a), 1.77-1.61 (m, 2 H, CH_2), 1.62-1.49 (m, 3 H, H-14ax, CH_2), 1.48 (s, 3 H, 12-CH_3), 1.40-1.20 (m, 22 H, H-7a, H-4′b, 2 H-cyclopropyl, 6-CH_3, H-7b, 10-CH_3, 5′-CH_3, 4(CH_2)), 1.21-1.16 (m, 2 H, 2 H-cyclopropyl), 1.14 (d, *J* = 6.8 Hz, 3 H, 2-CH_3), 1.05 (d, *J* = 7.5 Hz, 3 H, 4-CH_3), 0.93 (d, *J* = 7.0 Hz, 3 H, 8-CH_3), 0.86 (t, *J* = 7.4 Hz, 3 H, 15-CH_3). ^{13}C NMR ($CDCl_3$, 175 MHz), δ: 176.70, 174.08, 166.94, 164.45, 156.45, 154.67, 147.37, 140.39, 139.12, 133.64, 127.43, 125.96, 116.79, 111.27, 102.28, 85.04, 83.18, 81.25, 78.18, 77.84, 75.48, 70.28, 68.63, 65.97, 61.45, 49.88, 43.34, 41.17, 40.23, 37.30, 35.87, 35.25, 34.80, 32.74, 31.14, 30.42, 30.15, 29.71, 29.22, 29.13, 28.84, 26.66, 25.56, 22.23, 20.95, 19.30, 18.89, 15.62, 14.85, 13.03, 10.19, 9.07, 8.19.

3-O-Descladinosyl-3-O-[N-9-(1-cyclopropyl-1,4-dihydro-4-oxo-3-quinolinecarboxamide-6-yl)nonylcarbamoyl]-6-O-methylerythromycin A 9-O- methyl oxime 11,12- cyclic carbonate ***(63b)***

Following the general procedure J, from compound **54** (0.250 g, 0.320 mmol) and **49** (0.210 g, 0.570 mmol), **63b** (40.6 mg, 0.0390 mmol, 12.2%) was obtained as an off-white solid (column chromatography eluents: DCM/MeOH/NH_3·H_2O = 10:0.1:0.1). HRMS (ESI) $(M+H)^+$ *m/z* 1040.6190, Calcd for $C_{55}H_{86}N_5O_{14}$ 1040.6166. ^{1}H NMR ($CDCl_3$, 400 MHz), δ: 9.75 (s, 1 H, N**H**$_2$-quinolyl), 8.88 (s, 1 H, 2″-quinolyl), 8.28 (s, 1 H, 5″-quinolyl), 7.95

(d, *J* = 8.7 Hz, 1 H, 8″-quinolyl), 7.60 (d, *J* = 8.7 Hz, 1 H, 7″-quinolyl), 8.90 (s, 1 H, N**H**$_2$-quinolyl), 5.25-5.13 (dd, *J* = 2.5 Hz, 10.8 Hz, 1 H, H-13), 4.91-4.81 (m, 2 H, H-3, H-11), 4.01 (d, *J* = 7.4 Hz, 1 H, H-1′), 3.82 (s, 3 H, 9-*O*-C**H**$_3$), 3.75-3.62 (m, 2 H, H-5, H-8), 3.59-3.50 (m, 1 H, H-cyclopropyl), 3.51-3.42 (m, 2 H, H-5′, 3-*O*-CONH-C**H**$_2$), 3.40-3.32 (m, 1 H, H-2′), 3.32-3.22 (m, 1 H, 3-*O*-CONH-C**H**$_2$), 3.02 (s, 3 H, 6-*O*-C**H**$_3$), 2.92-2.76 (m, 4 H, H-2, CH$_2$-quinolyl, H-3′), 2.73 (s, 6 H, -N(CH$_3$)$_2$), 2.47 (q, *J* = 7.6 Hz, 1 H, H-10), 2.12-2.02 (m, 1 H, H-4), 2.02-1.93 (m, 1 H, H-4′a), 1.92-1.85 (m, 1 H, H-14eq), 1.71-1.59 (m, 2 H, H-7a, H-14ax), 1.49 (s, 3 H, 12-CH$_3$), 1.48-1.20 (m, 27 H, H-4′b, 2H-cyclopropyl, 6-CH$_3$, H-7b, 10-CH$_3$, 7(CH$_2$), 5′-CH$_3$), 1.20-1.10 (m, 5 H, 2H-cyclopropyl, 2-CH$_3$), 1.08 (d, *J* = 7.6 Hz, 3 H, 4-CH$_3$), 0.93 (d, *J* = 7.0 Hz, 3 H, 8-CH$_3$), 0.86 (t, *J* = 7.5 Hz, 3 H, 15-CH$_3$); ^{13}C NMR (CDCl$_3$, 100 MHz), δ: 176.66, 174.14, 166.99, 164.51, 156.52, 154.66, 147.29, 145.45, 145.40, 140.46, 139.21, 139.09, 133.62, 132.30, 132.14, 127.42, 125.89, 124.71, 124.56, 116.92, 116.78, 111.21, 101.72, 85.07, 83.17, 80.81, 78.16, 77.28, 75.39, 70.17, 68.24, 65.90, 61.41, 49.90, 45.91, 43.31, 41.12, 40.24, 39.62, 38.14, 37.25, 35.87, 35.35, 34.80, 32.73, 31.22, 30.88, 30.23, 29.51, 29.43, 29.31, 29.26, 29.14, 29.07, 27.51, 27.41, 26.77, 26.68, 25.55, 22.40, 22.21, 20.90, 19.27, 18.89, 15.60, 14.83, 13.01, 10.17, 9.07, 8.65, 8.17.

3-O-Descladinosyl-3-O-[N-10-(1-cyclopropyl-1,4-dihydro-4-oxo-3-quinolinecarboxamide-6-yl)decylcarbamoyl]-6-O-methylerythromycin A 9-O- methyl oxime 11,12- cyclic carbonate ***(64b)***

Following the general procedure K, from compound **54** (0.130 g, 0.170 mmol) and **50** (0.120 g, 0.310 mmol), **64b** (30.5 mg, 0.0290 mmol, 17.0%) was obtained as an off-white solid (column chromatography eluents: DCM/MeOH/NH$_3$·H$_2$O = 10:0.1:0.1). HRMS (ESI) (M+H)$^+$ *m/z* 1054.6315, Calcd for $C_{56}H_{88}N_5O_{14}$ 1054.6322. ^{1}H NMR (CDCl$_3$, 400 MHz), δ: 9.76 (d, *J* = 4.4 Hz, 1 H, N**H**$_2$-quinolyl), 8.88 (s, 1 H, 2″-quinolyl), 8.30 (s, 1 H, 5″-quinolyl), 7.95 (d, *J* = 8.7 Hz, 1 H, 8″-quinolyl), 7.60 (d, *J* = 8.7 Hz, 1 H, 7″-quinolyl), 5.85 (s, 1 H, N**H**$_2$-quinolyl), 5.56 (s, 1 H, 3-*O*-CONH), 5.16 (dd, *J* = 2.5 Hz, 10.8 Hz, 1 H, H-13), 4.91-4.81 (m, 2 H, H-3, H-11), 4.01 (d, *J* = 7.4 Hz, 1 H, H-1′), 3.82 (s, 3 H, 9-*O*-C**H**$_3$), 3.75-3.65 (m, 2 H, H-5, H-8), 3.58-3.48 (m, 1 H, H-cyclopropyl), 3.42-3.09 (m, 1 H, H-5′), 3.38-3.15 (m, 2 H, 3-*O*-CONH-C**H**$_2$, H-2′), 3.10-3.02 (m, 1 H, 3-*O*-CONH-C**H**$_2$), 3.02 (s, 3 H, 6-*O*-C**H**$_3$), 2.91-2.81 (m, 3 H, H-2, CH$_2$), 2.64 (s, 6 H, -N(CH$_3$)$_2$), 2.52-2.38 (m, 2 H, H-10, H-3′), 2.12-2.02 (m, 1 H, H-4), 1.99-1.83 (m, 2 H, H-14eq, H-4′a), 1.72-1.59 (m, 2 H, CH$_2$), 1.59-1.50 (m, 2 H, H-7a, H-14ax), 1.49 (s, 3 H, 12-CH$_3$), 1.40-1.10 (m, 29 H, H-4′a, 4 H-cyclopropyl, 6-CH$_3$, H-7b, 5′-CH$_3$, 7(CH$_2$), 10-CH$_3$), 1.18-1.14 (m, 6 H, 2-CH$_3$), 1.08 (d, *J* = 7.6 Hz, 3 H, 4-CH$_3$), 0.93 (d, *J* = 7.0 Hz, 3 H, 8-CH$_3$), 0.86 (t, *J* = 7.5 Hz, 3 H, 15-CH$_3$); ^{13}C NMR (CDCl$_3$, 100 MHz), δ: 176.69, 174.10, 166.96, 164.47, 156.46, 154.67, 147.32, 140.51, 139.21, 139.10, 133.61, 127.45, 125.95, 124.72, 116.89, 116.73, 111.26, 102.12, 85.05, 83.18, 81.07, 78.19, 77.72, 77.25, 70.27, 68.53, 65.97, 61.44, 49.89, 43.34, 41.16, 40.25, 39.63, 38.21, 37.30, 35.88, 35.39, 34.79, 32.74, 31.25, 30.43, 30.24, 29.70, 29.52, 29.40, 29.29, 29.23, 29.11, 27.60, 26.78, 25.56, 22.39, 22.23, 20.96, 19.29, 18.89, 15.62, 14.85, 13.03, 10.18, 9.05, 8.18.

Synthesis of compounds 70-72 and 74

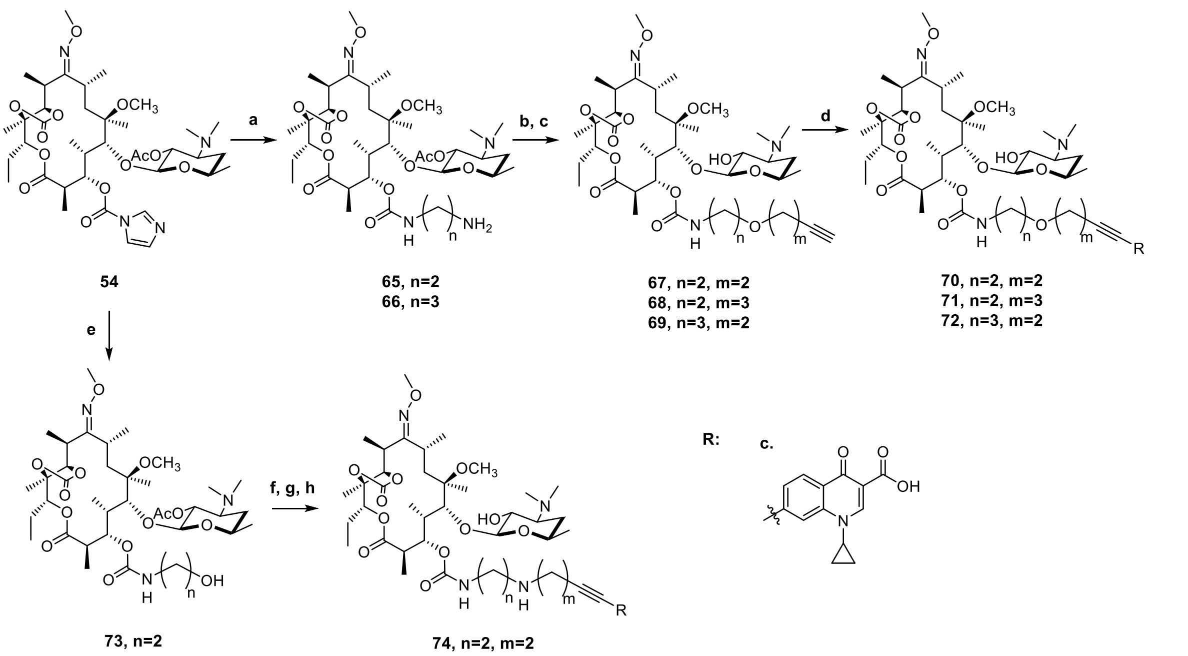

Scheme S8. Synthesis of *erythromycin A 9-O-methyl oxime 11,12-cyclic carbonate* series compounds **70-72** and **74**. Reagents and conditions: (a) ethylenediamine or 1,3-diaminopropane, DMF, rt, 3 h; (b) CH_3OH, 65 ˚C, 2 h; (c) 3-butyn-1-ol or 4-pentyn-1-ol, $NaNO_2$, HCOOH, -15˚C, 12-72 h; (d) R-I (R = **c**), CuI, $Pd(PPh_3)_2Cl_2$, Et_3N, CH_3CN, 45 ˚C, 12 h, (e) 2-aminoethanol, DBU, DMF, rt, 12 h; (f) methanesulfonyl chloride, Et_3N, DCM, rt, 10 min; (g) **34**, CH_3CN, 75 ˚C, 72 h; (h) CH_3OH, 65 ˚C, 2 h.

General procedure K

To a solution of **54** (1 eq) in dry DMF was added ethylenediamine or 1,3-diaminopropane (5 eq). The reaction mixture was stirred at room temperature for 5 h. After completion of the reaction, ethyl acetate (50 mL) and water (50 mL) were added into the reaction mixture. The organic layer was washed with water and brine and concentrated in vacuo. The crude mixture was purified by column chromatography on silica gel.

*2'-O-Acetyl-3-O-descladinosyl-3-O-(N-aminoethylcarbamoyl)-6-O-methylerythromycin A 9-O- methyl oxime 11,12-cyclic carbonate **(65)***

Following the general procedure K, from compound **54** (2.50 g, 3.20 mmol) and ethylenediamine (1.08 mL, 16.0 mmol), **65** (1.92 g, 2.48 mmol, 77.4%) was obtained as an off-white solid (column chromatography eluents: DCM/MeOH/NH_3·H_2O = 10:0.8:0.1).

*2'-O-Acetyl-3-O-descladinosyl-3-O-(N-aminopropylcarbamoyl)-6-O-methylerythromycin A 9-O- methyl oxime 11,12- cyclic carbonate **(66)***

Following the general procedure K, from compound **54** (2.48 g, 3.19 mmol) and 1,3-diaminopropane (1.24 mL, 15.9 mmol), **66** (1.23 g, 1.56 mmol, 48.9%) was obtained as an off-white solid (column chromatography eluents: DCM/MeOH/NH_3·H_2O = 10:0.8:0.1).

General procedure L

The compound **65** or **66** was then dissolved in MeOH (15 mL) at 60 ˚C for 2 h. The organic solvent was removed in vacuo. The intermediate was dissolved in 3-butyn-1-ol or 4-pentyn-1-ol (20-100 eq), and HCOOH (4.5 eq) was added dropwise. $NaNO_2$ (6 eq) was then added and the mixture was stirred at -15 ˚C for 24-48 h. After completion of the reaction, CH_2Cl_2 (50 mL) and water (50 mL) were added into the reaction mixture. The organic layer was washed with water and brine, and concentrated in vacuo. The crude mixture was purified by column chromatography on silica gel.

*3-O-Descladinosyl-3-O-(N-but-3-ynoxy-ethylcarbamoyl)-6-O-methylerythromycin A 9-O- methyl oxime 11,12- cyclic carbonate **(67)***

Following the general procedure L, from compound **65** (0.897 g, 1.16 mmol) and 3-butyn-1-ol (8.79 mL, 116 mmol), **67** (0.398 g, 0.482 mmol, 41.5%) was obtained as an off-white solid (column chromatography eluents: petroleum ether/DCM/MeOH/$NH_3 \cdot H_2O$ = 3:7:0.1:0.1).

*3-O-Descladinosyl-3-O-(N-pent-4-ynoxy-ethylcarbamoyl)-6-O-methylerythromycin A 9-O- methyl oxime 11,12- cyclic carbonate **(68)***

Following the general procedure L, from compound **65** (1.50 g, 1.94 mmol) and 4-pentyn-1-ol (5.42 mL, 58.2 mmol), **68** (0.347 g, 0.428 mmol, 22.0%) was obtained as an off-white solid (column chromatography eluents: petroleum ether/DCM/MeOH/$NH_3 \cdot H_2O$ = 3:7:0.2:0.1).

*3-O-Descladinosyl-3-O-(N-but-3-ynoxy-propylcarbamoyl)-6-O-methylerythromycin A 9-O- methyl oxime 11,12- cyclic carbonate **(69)***

Following the general procedure L, from compound **66** (1.23 g, 1.56 mmol) and 3-butyn-1-ol (11.8 mL, 156 mmol), **67** (0.469 g, 0.586 mmol, 37.6%) was obtained as an off-white solid (column chromatography eluents: petroleum ether/DCM/MeOH/$NH_3 \cdot H_2O$ = 4:6:0.1:0.1). HRMS (ESI) $(M+H)^+$ *m/z* 798.4725, calcd for $C_{40}H_{68}N_3O_{13}$ 798.4747. ^{1}H NMR ($CDCl_3$, 400 MHz) δ: 5.48 (t, *J* = 5.8 Hz, 1 H, 3-*O*-CO-N**H**-CH_2), 5.17 (dd, *J* = 2.6 Hz, 10.7 Hz, 1 H, H-13), 4.92-4.82 (m, 2 H, H-3, H-11), 4.05 (d, *J* = 7.2 Hz, 1 H, H-1′), 3.82 (s, 3H, 9-*O*-CH_3), 3.77 (d, *J* = 3.2 Hz, 1 H, H-5), 3.74-3.66 (m, 1 H, H-8), 3.61-3.52 (m, 4 H, 3-*O*-CO-NH-CH_2-C**H₂**, -O-C**H₂**-CH_2-C≡CH), 3.49-3.35 (m, 2 H, H-5′, 3-*O*-CO-NH-C**H₂**), 3.27-3.17 (m, 2 H, H-2′, 3-*O*-CO-NH-C**H₂**), 3.02 (s, 3 H, 6-*O*-CH_3), 2.90-2.81 (m, 1 H, H-2), 2.64-2.53 (m, 2 H, H-3′), 2.52-2.43 (m, 3 H, C**H₂**-C≡CH, H-10), 2.38 (s, 6 H, -N$(CH_3)_2$), 2.12-2.03 (m, 2 H, C≡CH, H-4), 1.96-1.74 (m, 3 H, H-14eq, 3-*O*-CO-NH-CH_2-CH_2-C**H₂**), 1.74-1.67 (m, 1 H, H-4′a), 1.62-1.51 (m, 1 H, H-14ax), 1.49 (s, 3 H, 12-CH_3), 1.42-1.33 (m, 1 H, H-4′b), 1.32 (s, 3 H, 6-CH_3), 1.29-1.17 (m, 8 H, H-7a, H-7b, 5′-CH_3, 10-CH_3), 1.14 (d, *J* = 6.7 Hz, 3 H, 2-CH_3), 1.06 (d, *J* = 7.5 Hz, 3 H, 4-CH_3), 0.93 (d, *J* = 7.0 Hz, 3 H, 8-CH_3), 0.86 (t, *J* = 7.3 Hz, 3 H, 15-CH_3).

*3-O-Descladinosyl-3-O-{N-[4-(1-cyclopropyl-1,4-dihydro-4-oxo-3-quinolinecarboxylic acid-7-yl)]-but-3-ynoxy-ethylcarbamoyl}-6-O-methylerythromycin A 9-O- methyl oxime 11,12- cyclic carbonate **(70c)***

Following the general procedure I-1, from compound **67** (0.398 g, 0.482 mmol) and **4** (0.205 g, 0.578 mmol), **70c** (52.5 mg, 0.0426 mmol, 7.27%) was obtained as an off-white solid (column chromatography eluents: DCM/MeOH/$NH_3 \cdot H_2O$ = 10:0.8:0.5). m.p. 161.5-163.7 °C. HRMS (ESI) $(M+H)^+$ *m/z* 1011.5182, calcd for $C_{52}H_{75}N_4O_{16}$ 1011.5173. ^{1}H NMR ($CDCl_3$, 400 MHz) δ: 8.86 (s, 1 H, 2″-quinolyl), 8.41 (d, *J* = 8.3 Hz, 1 H, 5″-quinolyl), 8.08 (s, 1 H, 8″-quinolyl), 7.56 (dd, *J* = 1.4 Hz, 8.3 Hz, 1 H, 6″-quinolyl), 5.30 (br, 1 H, 3-*O*-CO-N**H**-CH_2), 5.15 (dd, *J* = 2.5 Hz, 10.8 Hz, 1 H, H-13), 4.90-4.79 (m, 2 H, H-3, H-11), 4.01 (d, *J* = 7.2 Hz, 1 H, H-1′), 3.82 (s, 3 H, 9-*O*-CH_3), 3.77-3.66 (m, 4 H, H-5, H-8, 3-*O*-CO-NH-CH_2-C**H₂**), 3.65-3.53 (m, 4 H, C**H₂**-CH_2-O-C**H₂**-CH_2-C≡C-quinolyl, 1 H-cyclopropyl), 3.43-3.33 (m, 1 H, H-5′), 3.32-3.23 (m, 1 H, 3-*O*-CO-NH-C**H₂**), 3.18 (dd, *J* = 7.2 Hz, 10.2 Hz, 1 H, H-2′), 3.01 (s, 3 H, 6-*O*-CH_3), 2.78 (t, *J* = 6.7 Hz, 2 H, C**H₂**-C≡C-quinolyl), 2.76-2.68 (m, 1 H, H-2), 2.52-2.37 (m, 2 H, H-3′, H-10,), 2.29 (s, 6 H, -N$(CH_3)_2$), 2.10-2.00 (m, 1 H, H-4), 1.96-1.84 (m, 1 H, H-14eq), 1.69-1.62 (m, 1 H, H-4′a), 1.61-1.51 (m, 1 H, H-14ax), 1.48 (s, 3 H, 12-CH_3), 1.46-1.35 (m, 3 H, 2 H-cyclopropyl, H-4′b), 1.32 (s, 3 H, 6-CH_3), 1.30-1.16 (m, 10 H, 2 H-cyclopropyl, H-7a, H-7b, 5′-CH_3, 10-CH_3), 1.11 (d, *J* = 6.8 Hz, 3 H, 2-CH_3), 0.99 (d, *J* = 7.5 Hz, 3 H, 4-CH_3), 0.93 (d, *J* = 7.0 Hz, 3 H, 8-CH_3), 0.85 (t, *J* = 7.4 Hz, 3 H, 15-CH_3). ^{13}C NMR ($CDCl_3$, 100 MHz) δ: 178.15, 173.98, 166.65, 164.58, 156.48, 154.66, 148.53, 141.05, 129.53, 129.37, 126.95, 125.07, 120.03, 109.07, 103.25, 92.10, 85.03, 83.18, 81.11, 80.69, 78.42, 78.35, 77.24, 75.55, 70.46, 68.80, 66.05, 61.40, 49.81, 43.30, 40.89, 40.35, 37.32, 35.94, 35.36, 32.74, 28.70, 25.61, 22.20, 21.17, 20.95, 19.30, 18.86, 15.59, 14.87, 12.98, 10.17, 8.83, 8.38, 8.33.

*3-O-Descladinosyl-3-O-{N-[5-(1-cyclopropyl-1,4-dihydro-4-oxo-3-quinolinecarboxylic acid-7-yl)]pent-4-ynoxy-ethylcarbamoyl}-6-O-methylerythromycin A 9-O- methyl oxime 11,12- cyclic carbonate **(71c)***

Following the general procedure I-1, from compound **68** (0.347 g, 0.428 mmol) and **4** (0.182 g, 0.514 mmol),

70c (22.7 mg, 0.0221 mmol, 5.17%) was obtained as an off-white solid (column chromatography eluents: DCM/MeOH/$NH_3 \cdot H_2O$ = 10:0.8:0.5). m.p. 148.0-148.2 °C. HRMS (ESI) $(M+H)^+$ *m/z* 1025.5347, calcd for $C_{53}H_{77}N_4O_{16}$ 1025.5329. ^1H NMR ($CDCl_3$, 400 MHz) δ: 8.79 (s, 1 H, 2″-quinolyl), 8.34 (d, *J* = 8.3 Hz, 1 H, 5″-quinolyl), 7.99 (s, 1 H, 8″-quinolyl), 7.47 (dd, *J* = 8.3 Hz, 1 H, 6″-quinolyl), 5.29 (br, 1 H, 3-*O*-CO-N**H**-CH_2), 5.09 (dd, *J* = 2.5 Hz, 10.6 Hz, 1 H, H-13), 4.85-4.70 (m, 2 H, H-3, H-11), 3.98 (br, 1 H, H-1′), 3.74 (s, 3 H, 9-*O*-CH_3), 3.69-3.58 (m, 2 H, H-5, H-8), 3.65-3.53 (m, 6 H, 3-*O*-CO-NH-C**H₂**-C**H₂**-O-C**H₂**-CH_2-CH_2-C≡C-quinolyl, 1 H-cyclopropyl), 3.39-3.26 (m, 1 H, H-5′), 3.23-3.10 (m, 2 H, H-2′, 3-*O*-CO-NH-C**H**$_2$), 2.94 (s, 3 H, 6-*O*-CH_3), 2.83-2.70 (m, 1 H, H-2), 2.52 (t, *J* = 7.1 Hz, 2 H, C**H₂**-C≡C-quinolyl), 2.46-2.34 (m, 2 H, H-3′, H-10), 2.29 (s, 6 H, -N$(CH_3)_2$), 2.06-1.94 (m, 1 H, H-4), 1.91-1.76 (m, 3 H, H-14eq, C**H₂**-CH_2-C≡C-quinolyl), 1.67-1.55 (m, 1 H, H-4′a), 1.54-1.44 (m, 1 H, H-14ax), 1.41 (s, 3 H, 12-CH_3), 1.40-1.28 (m, 3 H, 2 H-cyclopropyl, H-4′b), 1.25 (s, 3 H, 6-CH_3), 1.21-1.11 (m, 10 H, 2 H-cyclopropyl, H-7a, H-7b, 5′-CH_3, 10-CH_3), 1.07 (d, *J* = 6.8 Hz, 3 H, 2-CH_3), 0.99 (d, *J* = 7.4 Hz, 3 H, 4-CH_3), 0.86 (d, *J* = 7.0 Hz, 3 H, 8-CH_3), 0.78 (t, *J* = 7.4 Hz, 3 H, 15-CH_3). ^{13}C NMR ($CDCl_3$, 100 MHz) δ: 178.18, 174.05, 166.72, 164.55, 156.57, 154.67, 148.51, 141.07, 129.89, 129.37, 126.93, 124.92, 119.86, 109.04, 85.03, 83.20, 80.03, 78.33, 77.25, 75.52, 70.44, 69.57, 65.92, 61.43, 49.84, 43.32, 40.98, 40.23, 37.31, 35.97, 35.36, 32.72, 28.50, 25.60, 22.22, 21.17, 19.32, 18.87, 16.43, 15.61, 14.89, 13.01, 10.19, 8.93, 8.36.

3-O-Descladinosyl-3-O-{N-[4-(1-cyclopropyl-1,4-dihydro-4-oxo-3-quinolinecarboxylic acid-7-yl)]but-3-ynoxy-propylcarbamoyl}-6-O-methylerythromycin A 9-O- methyl oxime 11,12- cyclic carbonate ***(72c)***

Following the general procedure I-1, from compound **69** (0.468 g, 0.586 mmol) and **4** (0.250 g, 0.703 mmol), **70c** (43.7 mg, 0.0426 mmol, 7.27%) was obtained as an off-white solid (column chromatography eluents: DCM/MeOH/$NH_3 \cdot H_2O$ = 10:0.9:0.5). m.p. 144.6-145.2 °C. HRMS (ESI) $(M+H)^+$ *m/z* 1025.5295, calcd for $C_{53}H_{77}N_4O_{16}$ 1025.5329. HPLC purity: 96.5 % (t= 14.3 min). ^1H NMR ($CDCl_3$, 400 MHz) δ: 8.86 (s, 1 H, 2″-quinolyl), 8.42 (d, *J* = 8.3 Hz, 1 H, 5″-quinolyl), 8.08 (s, 1 H, 8″-quinolyl), 7.56 (dd, *J* = 1.1 Hz, 8.3 Hz, 1 H, 6″-quinolyl), 5.36 (br, 1 H, 3-*O*-CO-N**H**-CH_2), 5.16 (dd, *J* = 2.3 Hz, 10.7 Hz, 1 H, H-13), 4.89-4.80 (m, 2 H, H-3, H-11), 4.03 (d, *J* = 7.2 Hz, 1 H, H-1′), 3.82 (s, 3 H, 9-*O*-CH_3), 3.75-3.65 (m, 4 H, H-5, H-8, 3-*O*-CO-NH-CH_2-C**H₂**), 3.65-3.55 (m, 3 H, -O-C**H₂**-CH_2-C≡C-quinolyl, 1 H-cyclopropyl), 3.49-3.33 (m, 2 H, H-5′, 3-*O*-CO-NH-C**H₂**), 3.28-3.16 (m, 2 H, H-2′, 3-*O*-CO-NH-C**H₂**), 3.01 (s, 3 H, 6-*O*-CH_3), 2.84-2.72 (m, 3 H, H-2, C**H₂**-C≡C-quinolyl), 2.60-2.42 (m, 2 H, H-3′, H-10), 2.35 (s, 6 H, -N$(CH_3)_2$), 2.12-2.00 (m, 1 H, H-4), 1.96-1.78 (m, 3 H, H-14eq, 3-*O*-CO-NH-CH_2-CH_2-C**H₂**), 1.71-1.63 (m, 1 H, H-4′a), 1.61-1.50 (m, 1 H, H-14ax), 1.48 (s, 3 H, 12-CH_3), 1.46-1.34 (m, 3 H, 2 H-cyclopropyl, H-4′b), 1.32 (s, 3 H, 6-CH_3), 1.30-1.16 (m, 10 H, 2 H-cyclopropyl, H-7a, H-7b, 5′-CH_3, 10-CH_3), 1.11 (d, *J* = 6.6 Hz, 3 H, 2-CH_3), 1.05 (d, *J* = 7.5 Hz, 3 H, 4-CH_3), 0.93 (d, *J* = 7.1 Hz, 3 H, 8-CH_3), 0.85 (t, *J* = 7.4 Hz, 3 H, 15-CH_3). ^{13}C NMR ($CDCl_3$, 100 MHz) δ: 178.18, 174.03, 166.67, 164.53, 156.47, 154.65, 148.52, 141.06, 129.65, 129.39, 126.97, 125.06, 119.98, 109.09, 103.24, 92.29, 85.01, 83.19, 81.51, 80.57, 78.35, 78.21, 77.23, 75.54, 70.47, 69.27, 69.08, 68.83, 65.94, 61.42, 49.80, 43.40, 40.18, 38.99, 37.36, 35.97, 35.36, 32.75, 29.97, 28.97, 25.59, 22.24, 21.14, 21.01, 19.31, 18.85, 15.59, 14.90, 13.00, 10.18, 8.92, 8.36.

2′-O-Acetyl-3-O-descladinosyl-3-O-(N-hydroxyethylcarbamoyl)-6-O-methylerythromycin A 9-O- methyl oxime 11,12- cyclic carbonate ***(73)***

Following the general procedure H, from compound **54** (1.00 g, 1.28 mmol) and 2-aminoethanol (0.23 mL, 3.85 mmol), **73** (0.472 g, 0.611 mmol, 47.7 %) was obtained as an off-white solid (column chromatography eluents: DCM/MeOH/$NH_3 \cdot H_2O$ = 10:0.3:0.1).

3-O-Descladinosyl-3-O-{N-[4-(1-cyclopropyl-1,4-dihydro-4-oxo-3-quinolinecarboxylic acid-7-yl)-1-yl]-but-3-ynyl-aminoethylcarbamoyl}-6-O-methylerythromycin A 9-O-methyl oxime 11,12- cyclic carbonate ***(74c)***

To a solution of **73** (0.472 g, 0.611 mmol) in CH_2Cl_2, methanesulfonyl chloride (0.055 mL, 0.672 mmol) and Et_3N (0.13 mL, 0.916 mmol) were added. The mixture was stirred at room temperature for 1 h. The CH_2Cl_2 layer was washed with brine, and evaporated in vacuo. The organic solvent was removed in vacuo and purified by column chromatography (100-200 mesh silicone, CH_2Cl_2/EtOH/$NH_3.H_2O$=10/0.1/0.1) to obtain an intermediate (0.350 g, 0.435 mmol, 71.2%).

To a solution of the intermediate (0.496 g, 0.616 mmol) in CH_3CN, **34** (0.0819 g, 0.246 mmol) was added. The mixture was stirred at 75˚C for 48 h, followed by the addition of CH_2Cl_2 (50 mL) and water (50 mL). The CH_2Cl_2 layer was washed with brine, and evaporated under vacuum. The residue was then dissolved in MeOH (15 mL) at 65 ˚C for 2 h. The organic solvent was removed in vacuo and purified by column chromatography (100-200 mesh silicone, CH_2Cl_2/EtOH/$NH_3.H_2O$ = 10/0.8/0.5) to yield **74c** (9.0 mg, 0.00890 mmol, 3.62 %). HRMS (ESI) $(M+H)^+$ *m/z* 1010.5351, calcd for $C_{52}H_{76}N_5O_{15}$ 1010.5332. ^1H NMR ($CDCl_3$, 400 MHz) δ: 8.84 (s, 1 H, 2″-quinolyl), 8.39 (d, *J* = 8.3 Hz, 1 H, 5″-quinolyl), 8.07 (s, 1 H, 8″-quinolyl), 7.54 (d, *J* = 8.3 Hz, 1 H, 6″-quinolyl), 5.45 (br, 1 H, 3-*O*-CO-N**H**-CH_2), 5.16 (dd, *J* = 2.5 Hz, 10.8 Hz, 1 H, H-13), 4.91-4.81 (m, 2 H, H-3, H-11), 4.03 (d, *J* = 7.2 Hz, 1 H, H-1′), 3.82 (s, 3 H, 9-*O*-CH_3), 3.75 (d, *J* = 3.0 Hz, 1 H, H-5), 3.73-3.64 (m, 1 H, H-8), 3.63-3.54 (m, 1 H, 1 H-cyclopropyl), 3.54-3.44 (m, 1 H, 3-*O*-CO-NH-C**H**$_2$), 3.42-3.31 (m, 1 H, H-5′), 3.25-3.12 (m, 2 H, 3-*O*-CO-NH-C**H**$_2$, H-2′), 3.01 (s, 3 H, 6-*O*-CH_3), 2.99-2.91 (m, 2 H, C**H**$_2$-CH_2-C≡C-quinolyl), 2.91-2.84 (m, 2 H, C**H**$_2$-NH), 2.84-2.75 (m, 1 H, H-2), 2.71 (t, *J* = 6.5 Hz, 2 H, C**H**$_2$-C≡C-quinolyl), 2.54-2.41 (m, 2 H, H-3′, H-10), 2.31 (s, 6 H, -N$(CH_3)_2$), 2.12-2.01 (m, 1 H, H-4), 1.97-1.83 (m, 1 H, H-14eq), 1.69-1.61 (m, 1 H, H-4′a), 1.61-1.51 (m, 1 H, H-14ax), 1.48 (s, 3 H, 12-CH_3), 1.46-1.34 (m, 3 H, 2 H-cyclopropyl, H-4′b), 1.32 (s, 3 H, 6-CH_3), 1.30-1.15 (m, 10 H, 2 H-cyclopropyl, H-7a, H-7b, 5′-CH_3, 10-CH_3), 1.12 (d, *J* = 6.7 Hz, 3 H, 2-CH_3), 1.04 (d, *J* = 7.4 Hz, 3 H, 4-CH_3), 0.93 (d, *J* = 7.0 Hz, 3 H, 8-CH_3), 0.84 (t, *J* = 7.3 Hz, 3 H, 15-CH_3). ^{13}C NMR ($CDCl_3$, 175 MHz) δ: 178.03, 174.02, 166.84, 164.51, 156.59, 154.67, 148.54, 140.99, 129.46, 129.32, 126.92, 125.11, 119.99, 103.26, 93.07, 85.00, 83.20, 81.24, 80.92, 78.38, 78.34, 75.55, 70.48, 61.44, 49.81, 43.38, 40.38, 37.32, 35.97, 35.30, 32.73, 25.60, 22.21, 21.20, 19.33, 18.86, 15.61, 14.95, 13.02, 10.19, 8.90, 8.38, 8.35.

Synthesis of compounds 79

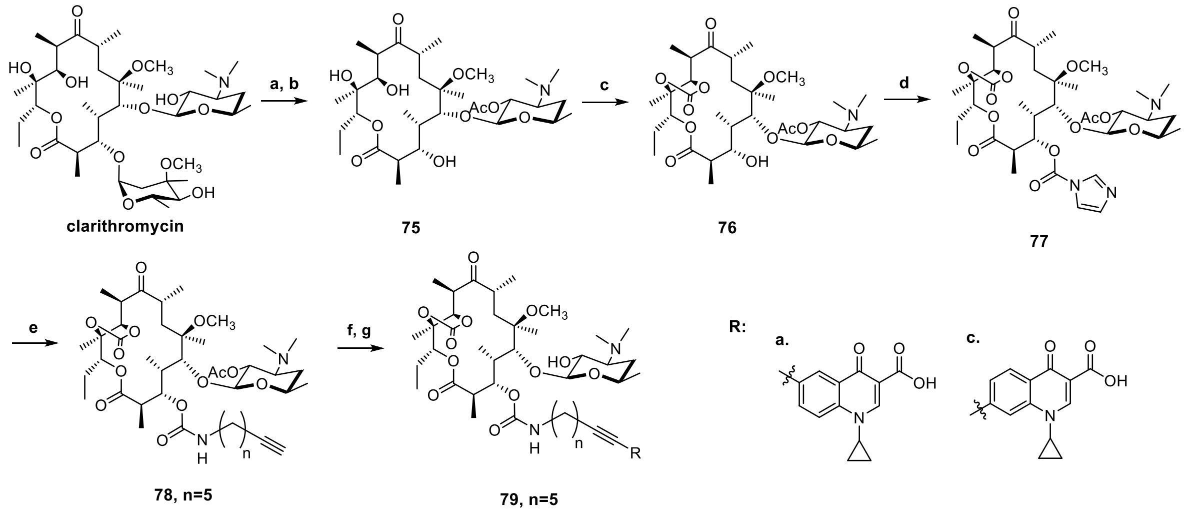

Scheme S9. Synthesis of *clarithromycin-11,12-cyclic carbonate* series compound **79**. Reagents and conditions: (a) 10% HCl, CH_3CH_2OH, 40 ˚C; (b) 1) acetic anhydride, DCM, rt, 1-1.5 h; 2) sat. $NaHCO_3$; (c) pyridine, BTC, DCM, -10 ˚C for 4 h then rt for 18 h; (d) CDI, DMAP, DCM, rt, 12 h; (e) **28**, DBU, DMF, rt, 12 h; (f) R-I (R = **a**, **c**), CuI, $Pd(PPh_3)_2Cl_2$, Et_3N, CH_3CN, 45 ˚C, 12 h; (g) CH_3OH, 65 ˚C, 2 h.

2′-O-Acetyl-3-O-descladinosyl-3-hydroxy-clarithromycin ***(75)***

10% HCl (40 mL) was added to a solution of clarithromycin (5.00 g, 6.69 mmol) in EtOH (15 mL) at 40 ˚C. The mixture was stirred at 40 ˚C for 1 h, and CH_2Cl_2 (100 mL) were added. The pH of the mixture was adjusted to 10 with ammonia water. The CH_2Cl_2 layer was washed with brine, and evaporated under vacuum to yield 3-OH-clarithromycin (3.89 g, 6.60 mmol, 98.7%).

To a solution of 3-OH-clarithromycin (3.35 g, 5.68 mmol) in dry CH_2Cl_2, acetic anhydride (1.07 mL, 11.4 mmol) was added. The mixture was stirred at room temperature for 1-2 h. The organic layer was washed with saturated

$NaHCO_3$ (5 × 50 mL), water and brine, and evaporated under reduced pressure to yield **75** (3.47 g, 5.53 mmol, 97.4%).

*2'-O-Acetyl-3-O-descladinosyl-3-hydroxy-clarithromycin-11,12- cyclic carbonate **(76)***

To a solution of **75** (2.70 g, 4.40 mmol) in CH_2Cl_2, and pyridine (2.13 mL, 26.4 mmol) was added. A solution of BTC (1.30 g, 4.40 mmol) in CH_2Cl_2 was added dropwise at -15 ˚C. After being stirred at -15 ˚C for 4 h, the reaction was warmed to room temperature and stirred for 10 h. This solution was washed with water (50 mL), saturated $NaHCO_3$ (50 mL) and brine. The organic layer was concentrated in vacuo and purified by column chromatography (100-200 mesh silicone, CH_2Cl_2/EtOH/$NH_3.H_2O$=10/0.15/0.05) to yield **76** (2.09 g, 3.18 mmol, 72.3%).

*2'-O-Acetyl-3-O-descladinosyl-3-O-(1H-imidazol-1-ylcarbonyl)-clarithromycin-11,12-cyclic carbonate **(77)***

The compound **76** (5.50 g, 8.36 mmol), CDI (4.07 g, 25.1 mmol) and DMAP (4.07 g, 25.1 mmol) were dissolved in dry CH_2Cl_2. The mixture was stirred at 25°C for 12-18 h followed by the addition of CH_2Cl_2 (50 mL) and water (50 mL). The organic layer was washed with water and brine, and concentrated in vacuo and purified by column chromatography (100-200 mesh silicone, CH_2Cl_2/EtOH/$NH_3.H_2O$=10/0.1/0.1) to yield **77** (5.65 g, 7.52 mmol, 90.0%). HRMS (ESI) $(M + H)^+$ *m/z* 752.3957, calcd for $C_{37}H_{58}N_3O_{13}$ 752.3964.^{1}H NMR ($CDCl_3$, 500 MHz) δ: 8.22 (s, 1 H, H-imidazole), 7.50 (s, 1 H, H-imidazole), 7.17 (s, 1 H, H-imidazole), 5.20 (d, *J* = 11.1 Hz, 1 H, H-3), 5.17 (dd, *J* = 10.9, 2.5 Hz, 1 H, H-13), 4.70 (s, 1 H, H-11), 4.62 (dd, *J* = 10.6, 7.5 Hz, 1 H, H-2′), 3.38 (d, *J* = 7.5 Hz, 1 H, H-1′), 4.21 (d, *J* = 3.4 Hz, 1 H, H-5), 3.10-3.09 (m, 4 H, H-2, 6-*O*-CH_3), 2.95 (q, *J* = 6.8 Hz, 1 H, H-10), 2.64-2.50 (m, 2 H, H-8, H-5′), 2.25-2.14 (m, 8 H, H-3′, H-4, $N(CH_3)_2$), 2.07 (s, 3 H, CH_3CO), 1.97-1.86 (m, 1 H, H-14eq), 1.65-1.57 (m, 1 H, H-14ax), 1.57-1.45 (m, 6 H, H-7a, H-7b, H-4′a, 12-CH_3), 1.27 (s, 3 H, 6-CH_3), 1.24-1.18 (m, 6 H, 2-CH_3, 4-CH_3), 1.17-1.08 (m, 10 H, H-4′b, 5′-CH_3, 8-CH_3, 10-CH_3), 0.89 (t, *J* = 7.4 Hz, 3 H, 15-CH_3).

*2'-O-Acetyl-3-O-descladinosyl-3-O-(N-6-heptynylcarbamoyl)-clarithromycin-11,12-cyclic carbonate **(78)***

Following the general procedure H, from compound **54** (2.00 g, 2.66 mmol) and **28** (0.471 g, 3.19 mmol), **78** (1.20 g, 1.51 mmol, 56.4%) was obtained as an off-white solid (column chromatography eluents: DCM/MeOH/$NH_3·H_2O$ = 10:0.3:0.1).

*3-O-descladinosyl-3-O-[N-7-(1-cyclopropyl-1,4-dihydro-4-oxo-3-quinolinecarboxylic acid-6-yl)-hept-6-ynylcarbamoyl]-clarithromycin-11,12-cyclic carbonate **(79a)***

Following the general procedure I-1, from compound **78** (0.270 g, 0.340 mmol) and 1-cyclopropyl-1,4-dihydro-4-oxo-6-iodo-3-quinolinecarboxylic acid (0.116 g, 0.340 mmol), **79a** (0.130 g, 0.133 mmol, 40.2%) was obtained as an off-white solid (column chromatography eluents: DCM/MeOH/$NH_3·H_2O$ = 10:0.8:0.5). HRMS (ESI) $(M + H)^+$ *m/z* 980.5068, calcd for $C_{52}H_{74}N_3O_{15}$ 980.5114. m. p. 159.2-160.4 °C. ^{1}H NMR ($CDCl_3$, 400 MHz) δ: 8.85 (s, 1 H, H-quinolyl), 8.47 (d, *J* = 1.9 Hz, 1 H, H-quinolyl), 8.02 (d, *J* = 8.7 Hz, 1 H, H-quinolyl), 7.81 (dd, *J* = 8.7, 1.9 Hz, 1 H, H-quinolyl), 5.49 (s, 1 H, CONH), 5.13 (dd, *J* = 10.8, 2.5 Hz, 1 H, H-13), 4.90 (d, *J* = 11.0 Hz, 1 H, H-3), 4.74 (s, 1 H, H-11), 4.09 (d, *J* = 7.6 Hz, 1 H, H-1′), 3.76 (d, *J* = 2.8 Hz, 1 H, H-5), 3.67-3.57 (m, 1 H, H-cyclopropyl), 3.47-3.32 (m, 2 H, -C**H**$_2$-NHCO, H-5′), 3.29-3.22 (m, 1 H, H-2′), 3.14-3.04 (m, 1H, -C**H**$_2$-NHCO), 3.00 (s, 3 H, 6-*O*-CH_3), 2.95 (q, *J* = 6.8 Hz, 1 H, H-10), 2.89-2.80 (m, 2 H, H-2, H-3′), 2.64-2.57 (m, 1 H, H-8), 2.49 (s, 6 H, $N(CH_3)_2$), 2.45 (t, *J* = 7.0 Hz, 2 H, -C**H$_2$**-C≡C-), 2.08-2.00 (m, 1 H, H-4), 1.92-1.84 (m, 1 H, H-14eq), 1.84-1.76 (m, 1 H, H-7a), 1.73-1.51 (m, 9 H, H-4′a, H-7b, 3(CH_2), H-14ax), 1.50 (s, 3 H, 12-CH_3), 1.46-1.39 (m, 2 H, H-cyclopropyl), 1.29 (s, 3 H, 6-CH_3), 1.26-1.07 (m, 18 H, 8-CH_3, 2H-cyclopropyl, H-4′b, 10-CH_3, 2-CH_3, 5′-CH_3, 4-CH_3), 0.84 (t, *J* = 7.3 Hz, 3 H, 15-CH_3). ^{13}C NMR ($CDCl_3$, 100 MHz) δ: 212.1, 178.1, 174.2, 166.7, 156.5, 154.1, 148.2, 140.0, 136.6, 129.8, 125.9, 122.7, 117.4, 109.0, 102.8, 92.9, 84.9, 81.9, 80.8, 79.3, 78.0, 78.0, 77.2, 75.3, 70.4, 69.0, 66.0, 49.9, 45.2, 43.4, 41.0, 40.1, 38.8, 37.5, 35.8, 35.5, 29.8, 29.5, 28.1, 26.0, 22.1, 21.1, 19.4, 18.4, 14.9, 13.0, 12.9, 10.1, 9.1, 8.3.

*3-O-descladinosyl-3-O-[N-7-(1-cyclopropyl-1,4-dihydro-4-oxo-3-quinolinecarboxylic acid-7-yl)-hept-6-ynylcarbamoyl]-clarithromycin-11,12-cyclic carbonate **(79c)***

Following the general procedure I-1, from compound **78** (0.275 g, 0.340 mmol) and **4** (0.135 g, 0.370 mmol), **79c** (0.160 g, 0.163 mmol, 41.8%) was obtained as an off-white solid (column chromatography eluents: DCM/MeOH/$NH_3·H_2O$ = 10:0.8:0.5). HRMS (ESI) $(M + H)^+$ *m/z* 980.5125, calcd for $C_{52}H_{74}N_3O_{15}$ 980.5114. m. p.

137.9-139.7 °C. ^{1}H NMR ($CDCl_3$, 400 MHz) δ: 8.79 (s, 1 H, H-quinolyl), 8.34 (d, *J* = 8.3 Hz, 1 H, H-quinolyl), 8.00 (s, 1 H, H-quinolyl), 7.47 (d, *J* = 8.3 Hz, 1 H, H-quinolyl), 5.45 (s, 1 H, CONH), 5.06 (dd, *J* = 10.8, 2.4 Hz, 1 H, H-13), 4.82 (d, *J* = 11.0 Hz, 1 H, H-3), 4.66 (s, 1 H, H-11), 4.01 (d, *J* = 7.0 Hz, 1 H, H-1′), 3.66 (d, *J* = 2.7 Hz, 1 H, H-5), 3.60-3.49 (m, 1 H, H-cyclopropyl), 3.40-3.26 (m, 2 H, -C**H**$_2$-NHCO, H-5′), 3.23-3.15 (m, 1 H, H-2′), 3.06-2.98 (m, 1 H, -C**H**$_2$-NHCO), 2.93 (s, 3 H, 6-*O*-CH_3), 2.88 (q, *J* = 6.8 Hz, 1 H, H-10), 2.80-2.67 (m, 2 H, H-10, H-3′), 2.60-2.49 (m, 1 H, H-8), 2.47-2.33 (m, 8 H, $N(CH_3)_2$, -**CH$_2$**-C≡C-), 2.04-1.93 (m, 1 H, H-4), 1.86-1.77 (m, 1 H, H-14eq), 1.73-1.44 (m, 10 H, H-7a, H-4′a, H-7b, -C≡CCH$_2$-C**H$_2$**-C**H$_2$**-C**H$_2$**-CH$_2$NCO, H-14ax), 1.42 (s, 3 H, 12-CH_3), 1.40-1.34 (m, 2 H, H-cyclopropyl), 1.21 (s, 3 H, 6-CH_3), 1.20-1.08 (m, 10 H, 8-CH_3, H-4′b, 2-CH_3,10-CH_3), 1.07-0.98 (m, 8 H, 2H-cyclopropyl, 5′-CH_3, 4-CH_3), 0.77 (t, *J* = 7.3 Hz, 3 H, 15-CH_3). ^{13}C NMR ($CDCl_3$, 100 MHz) δ: 212.0, 178.2, 174.2, 166.8, 156.5, 154.0, 148.5, 141.1, 130.1, 129.4, 126.9, 124.9, 119.9, 109.0, 84.9, 82.4, 80.8, 80.0, 78.1, 77.9, 77.2, 75.4, 70.4, 49.8, 45.2, 43.4, 40.9, 38.8, 37.5, 35.8, 35.4, 29.7, 28.0, 26.0, 22.0, 21.1, 19.5, 19.4, 18.4, 14.9, 13.0, 10.1, 9.1, 8.4.

Synthesis of compounds 87-90

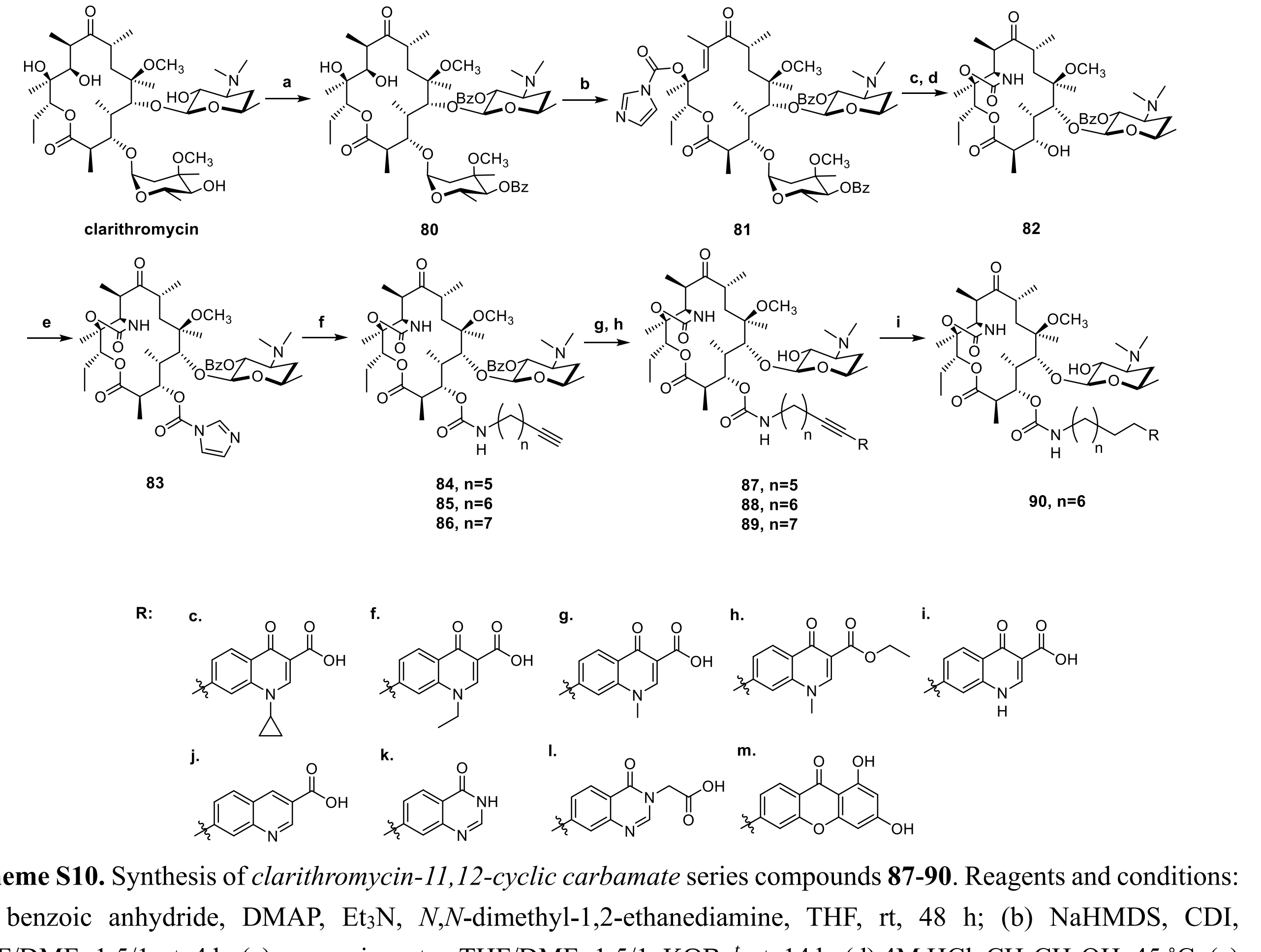

Scheme S10. Synthesis of *clarithromycin-11,12-cyclic carbamate* series compounds **87-90**. Reagents and conditions: (a) benzoic anhydride, DMAP, Et_3N, *N,N*-dimethyl-1,2-ethanediamine, THF, rt, 48 h; (b) NaHMDS, CDI, THF/DMF=1.5/1, rt, 4 h; (c) ammonia water, THF/DMF=1.5/1, KOBu^t, rt, 14 h; (d) 4M HCl, CH_3CH_2OH, 45 °C; (e) CDI, DMAP, DCM, rt, 12 h; (f) **28-30**, DBU, DMF, rt, 12 h; (g) (R = **c**, **g**, **i**, **m**), CuI, $Pd(PPh_3)_2Cl_2$, Et_3N, CH_3CN, 45 °C, 12 h; or R-Br (**j**-methyl ester) or R-I (R = **f**-ethyl ester, **h**, **k**, **l**-methyl ester), CuI, $Pd(PPh_3)_2Cl_2$, Et_3N, CH_3CN, 80 °C, 4 h; (h) CH_3OH, 65 °C, 12 h; (i) HCOOH, $HCOONH_4$, 10% Pd/C, H_2, CH_3OH, rt, 16 h.

*2′, 4″-O-dibenzoyl-clarithromycin **(80)***

To a stirred solution of clarithromycin (20.0 g, 26.7 mmol) in THF (150 mL), benzoic anhydride (18.1 g, 80.2 mmol), DMAP (3.27 g, 26.7 mmol) and Et_3N (11.1 mL, 80.2 mmol) were added. The reaction mixture was stirred at

rt for 48 h. After completion of the reaction, *N*,*N*-dimethyl-1,2-ethanediamine (5.84 mL, 53.5 mmol) was added into the reaction mixture, and the mixture was stirred at 0 ˚C for 30 min. The reaction mixture was concentrated, and the residue was diluted with CH_2Cl_2 and washed with sat. NH_4Cl, water and brine. The organic layer was concentrated in vacuo, and then the residue was redissolved in EtOH (100 mL). The precipitated solid was filtered to yield **80** (22.4 g, 23.7 mmol, 87.8%).

*2′, 4′′-O-dibenzoyl-10,11-enyl-12-O-(1H-imidazol-1-ylcarbonyl)-clarithromycin **(81)***

CDI (6.78 g, 41.8 mmol) and 2 M NaHMDS (8.37 mL, 16.7 mmol) were added to a solution of **80** (10.0 g, 10.4 mmol) in DMF/THF (42 mL/15 mL). The solution was allowed to stir for 4 h before being poured into water (50 mL) and CH_2Cl_2 (100 mL). The organic layer was washed with water and brine, and concentrated in vacuo to yield **81** (10.7 g, 10.3 mmol, 98.8%).

*2′-O-benzoyl-3-O-descladinosyl-3-hydroxy-clarithromycin-11,12- cyclic carbamate **(82)***

Compound **81** (10.7 g, 10.3 mmol) was dissolved in DMF/THF (42 mL/15 mL). Ammonia water (40 mL) was then added. The mixture was stirred at room temperature for 12 h. After then $KOBu^t$ (1.26 g, 10.3 mmol) was added to the reaction and stirred for 30 min. The reaction mixture was concentrated, and the residue was diluted with CH_2Cl_2 and washed with $NaHCO_3$ (50 mL), water and brine. The organic layer was concentrated in vacuo, and then the residue was redissolved in EtOH (40 mL). A solution of 4M HCl (40 mL) was added to the reaction. The mixture was stirred at 45 ˚C for 1 h, and then methyl tert-butyl ether (80 mL) and water (20 mL) were added. After stirring for 10 min, the pH of water layer was adjusted to 10 with ammonia water. The CH_2Cl_2 was added and CH_2Cl_2 layer was washed with brine. The organic phase was evaporated under vacuum to yield **82** (3.08 g, 4.28 mmol, 41.5%). HRMS (ESI) $(M+H)^+$ *m/z* 719.4114, calcd for $C_{38}H_{59}N_2O_{11}$ 719.4113. ^1H NMR ($CDCl_3$, 400 MHz) δ: 8.11-8.03 (m, 2 H, 2 H-Bz), 7.60-7.52 (m, 1 H, H-Bz), 7.48-7.42 (m, 2 H, 2 H-Bz), 5.77 (s, 1 H, 11-NH), 5.16 (dd, *J* = 2.3 Hz, 10.9 Hz, 1 H, H-13), 5.04 (dd, *J* = 7.6 Hz, 10.5 Hz, 1 H, H-2′), 4.76 (d, *J* = 7.6 Hz, 1 H, H-1′), 3.73 (d, *J* = 2.6 Hz, 1 H, H-5), 3.71 (s, 1 H, H-11), 3.61-3.51 (m, 1 H, H-5′), 3.46 (dd, *J* = 6.6 Hz, 10.6 Hz, 1 H, H-3), 2.95-2.89 (m, 1 H, H-3′), 2.97 (s, 3 H, 6-*O*-CH_3), 2.79 (q, *J* = 6.5 Hz, 1 H, H-10), 2.64-2.54 (m, 1 H, H-2), 2.52-2.42 (m, 1 H, H-8), 2.28 (s, 6 H, -$N(CH_3)_2$), 2.18 (d, *J* = 6.5 Hz, 1 H, 3-OH), 1.94-1.83 (m, 2 H, H-4, H-14eq), 1.94-1.81 (m, 1 H, H-4′a), 1.66-1.55 (m, 1 H, H-7a), 1.52-1.37 (m, 3 H, H-7b, H-14ax, H-4′a), 1.32 (s, 3 H, 12-CH_3), 1.31-1.24 (m, 6 H, 6-CH_3, 5′-CH_3), 1.22 (d, *J* = 6.8 Hz, 3 H, 2-CH_3), 1.09 (d, *J* = 6.6 Hz, 3 H, 10-CH_3), 1.05 (d, *J* = 7.1 Hz, 3 H, 8-CH_3), 0.81 (t, *J* = 7.4 Hz, 3 H, 15-CH_3), 0.71 (d, *J* = 7.4 Hz, 3 H, 4-CH_3). ^{13}C NMR ($CDCl_3$, 100 MHz) δ: 217.87, 175.24, 165.40, 158.54, 132.73, 130.65, 129.79, 128.26, 99.79, 83.95, 80.83, 77.96, 77.65, 75.53, 72.11, 68.99, 63.27, 58.18, 49.52, 45.34, 44.08, 40.82, 38.76, 37.18, 35.84, 32.00, 22.08, 21.15, 19.27, 18.13, 15.34, 13.82, 13.28, 10.22, 7.81.

*2′-O-benzoyl-3-O-descladinosyl-3-O-(1H-imidazol-1-ylcarbonyl) clarithromycin-11,12- cyclic carbamate **(83)***

The compound **82** (3.08 g, 4.28 mmol), CDI (2.08 g, 12.8 mmol) and DMAP (1.05 g, 8.57 mmol) were dissolved in dry CH_2Cl_2 (8 mL). The mixture was stirred at room temperature for 12 h followed by the addition of CH_2Cl_2 (50 mL) and water (50 mL). The organic layer was washed with water and brine, and concentrated in vacuo to yield **83** (3.42 g, 4.20 mmol, 98.1%).

*2′-O-benzoyl-3-O-descladinosyl-3-O-(N-6-heptynylcarbamoyl) clarithromycin-11,12- cyclic carbamate **(84)***

According to the general procedure H, from compound **83** (0.600 g, 0.748 mmol) and **28** (0.166 g, 1.12 mmol), **84** (0.308 g, 0.360 mmol, 48.1 %) was obtained as a white solid (column chromatography eluents: DCM/EtOH/$NH_3 \cdot H_2O$ = 10:0.1:0.05).

*2′-O-benzoyl-3-O-descladinosyl-3-O-(N-7-octynylcarbamoyl) clarithromycin-11,12- cyclic carbamate **(85)***

According to the general procedure H, from compound **83** (1.20 g, 1.47 mmol) and **29** (0.475 g, 2.94 mmol), **85** (0.399 g, 0.458 mmol, 31.2%) was obtained as a white solid (column chromatography eluents: DCM/EtOH/$NH_3 \cdot H_2O$ = 10:0.1:0.05). HRMS (ESI) $(M+H)^+$ *m/z* 870.5114, calcd for $C_{47}H_{72}N_3O_{12}$ 870.5111. ^1H NMR ($CDCl_3$, 400 MHz) δ: 8.08-8.00 (m, 2 H, 2 H-Bz), 7.62-7.55 (m, 1 H, 1 H-Bz), 7.50-7.43 (m, 2 H, 2 H-Bz), 5.76 (s, 1 H, 11-NH), 5.18 (dd, *J* = 2.3 Hz, 10.9 Hz, 1 H, H-13), 4.98 (dd, *J* = 7.4 Hz, 10.4 Hz, 1 H, H-2′), 4.91-4.84 (m, 2 H, H-3, 3-*O*-CO-N**H**-CH_2), 4.29 (d, *J* = 7.5 Hz, 1 H, H-1′), 3.83 (d, *J* = 3.2 Hz, 1 H, H-5), 3.70 (m, 1 H, H-11), 3.46-3.29 (m, 2 H, H-5′, 3-

O-CO-NH-C**H**$_2$), 3.19-3.09 (m, 1 H, 3-*O*-CO-NH-C**H**$_2$), 2.96 (s, 3 H, 6-*O*-CH$_3$), 2.86-2.69 (m, 3 H, H-3, H-10, H-2), 2.52-2.43 (m, 1 H, H-8), 2.28 (s, 6 H, -N(CH$_3$)$_2$), 2.23-2.16 (m, 2 H, -C**H**$_2$-C≡CH), 1.95 (t, *J* = 2.6 Hz, 1 H, -CH$_2$-C≡C**H**), 1.93-1.79 (m, 2 H, H-4, H-14eq), 1.79-1.73 (m, 1 H, H-4′a), 1.63-1.34 (m, 12 H, H-7a, H-7b, H-14eq, H-4′a, 4(-CH$_2$-)), 1.32 (s, 3 H, 12-CH$_3$), 1.29-1.23 (m, 6 H, 6-CH$_3$, 5′-CH$_3$), 1.15-1.04 (m, 9 H, 2-CH$_3$, 10-CH$_3$, 8-CH$_3$), 0.81 (t, *J* = 7.4 Hz, 3 H, 15-CH$_3$), 0.74 (d, *J* = 7.5 Hz, 3 H, 4-CH$_3$).

*2'-O-benzoyl-3-O-descladinosyl-3-O-(N-8-nonynylcarbamoyl) clarithromycin-11,12- cyclic carbamate **(86)***

According to the general procedure H, from compound **83** (0.605 g, 0.750 mmol) and **30** (0.159 g, 0.900 mmol), **86** (0.341 g, 0.350 mmol, 50.0%) was obtained as a white solid (column chromatography eluents: petroleum ether/DCM/EtOH/NH$_3$·H$_2$O = 4:6:0.1:0.05).

*3-O-descladinosyl-3-O-[N-7-(1-cyclopropyl-1,4-dihydro-4-oxo-3-quinolinecarboxylic acid-7-yl)-hept-6-ynylcarbamoyl] clarithromycin-11,12- cyclic carbamate **(87c)***

Following the general procedure I-1, from compound **84** (0.360 g, 0.470 mmol) and **4** (0.217 g, 0.570 mmol), **87c** (30.5 mg, 0.0310 mmol, 6.63%) was obtained as an off-white solid (column chromatography eluents: DCM/MeOH/NH$_3$·H$_2$O = 10:0.8:0.5). HPLC purity: 96.6% (t=10.5 min). HRMS (ESI) $(M + H)^+$ *m/z* 979.5274, calcd for $C_{52}H_{75}N_4O_{14}$ 979.5274. m. p. 137.0-139.5 ˚C. ^{1}H NMR (CDCl$_3$, 400 MHz) δ: 8.86 (s, 1 H, H-quinolyl), 8.41 (d, *J* = 8.3 Hz, 1 H, H-quinolyl), 8.07 (s, 1 H, H-quinolyl), 7.54 (d, *J* = 8.3 Hz, 1 H, H-quinolyl), 5.80 (s, 1 H, 11-CONH), 5.46 (s, 1H, *3*-CONH), 5.22 (dd, *J* = 10.9 Hz, *J* = 2.3 Hz, 1 H, H-13), 4.91 (d, *J* = 11.0 Hz, 1 H, H-3), 4.06 (d, *J* = 7.1 Hz, 1 H, H-1′), 3.76 (s, 1 H, H-11), 3.74 (d, *J* = 2.7 Hz, 1 H, H-5), 3.65-3.55 (m, 1 H, H-cyclopropyl), 3.46-3.33 (m, 2 H, -C**H**$_2$-NHCO, H-5′), 3.26-3.19 (m, 1H, H-2′), 3.11-3.02 (m, 1 H, -C**H**$_2$-NHCO), 2.97 (s, 3 H, 6-*O*-CH$_3$), 2.90 (q, *J* = 6.8 Hz, 1 H, H-10), 2.84-2.76 (m, 1 H, H-2), 2.73-2.62 (m, 1 H, H-3′), 2.50 (t, *J* = 6.7 Hz, 1 H, -C**H**$_2$-C≡CH), 2.42 (s, 7 H, N(CH$_3$)$_2$, H-8), 2.16-2.02 (m, 1 H, H-4), 1.97-1.86 (m, 1 H, H-14eq), 1.84-1.75 (m, 1 H, H-7a), 1.74-1.64 (m, 3 H, H-4′a, -C**H**$_2$-CH$_2$NCO), 1.64-1.49 (m, 6 H, H-7b, 2(CH$_2$), H-14ax), 1.49-1.37 (m, 5 H, 12-CH$_3$, 2H-cyclopropyl), 1.27 (s, 3 H, 6-CH$_3$), 1.30-1.26 (m, 8 H, 2 H-cyclopropyl, 5′-CH$_3$, 10-CH$_3$), 1.18-1.06 (m, 10 H, H-4′b, 2-CH$_3$, 4-CH$_3$, 8-CH$_3$), 0.83 (t, *J* = 7.3 Hz, 3 H, 15-CH$_3$). ^{13}C NMR (CDCl$_3$, 100 MHz) δ: 217.6, 178.2, 174.4, 166.7, 158.4, 156.5, 148.5, 141.1, 130.0, 129.4, 126.9, 124.9, 119.8, 109.0, 103.2, 95.5, 83.9, 82.5, 80.0, 78.3, 77.8, 77.2, 75.8, 70.5, 69.1, 65.7, 58.2, 49.8, 45.3, 43.4, 40.9, 39.9, 39.1, 37.3, 35.7, 35.4, 29.8, 28.0, 26.1, 22.0, 21.2, 19.5, 19.3, 18.3, 15.0, 13.9, 13.3, 10.2, 9.1, 8.4.

*3-O-descladinosyl-3-O-[N-8-(1-cyclopropyl-1,4-dihydro-4-oxo-3-quinolinecarboxylic acid-7-yl)-otc-7-ynylcarbamoyl]clarithromycin-11,12- cyclic carbamate **(88c)***

Following the general procedure I-1, from compound **85** (0.700 g, 0.870 mmol) and **4** (0.399 g, 1.04 mmol), **87c** (35.5 mg, 0.0360 mmol, 4.11%) was obtained as an off-white solid (column chromatography eluents: DCM/MeOH/NH$_3$·H$_2$O = 10:0.8:0.5). HPLC: t=10.66 min, 95.0%. HRMS (ESI) $(M + H)^+$ *m/z* 993.5415, calcd for $C_{53}H_{77}N_4O_{14}$ 993.5431. m. p. 126.3-128.4 ˚C. ^{1}H NMR (CDCl$_3$, 400 MHz) δ: 8.86 (s, 1 H, H-quinolyl), 8.41 (d, *J* = 8.3 Hz, 1 H, H-quinolyl), 8.06 (d, *J* = 1.3 Hz, 1 H, H-quinolyl), 7.55 (dd, *J* = 8.3, 1.3 Hz, 1 H, H-quinolyl), 5.80 (s, 1 H, 11-NH), 5.47 (t, *J* = 6.0 Hz, 1 H, *3*-CONH), 5.23 (dd, *J* = 10.9, 2.3 Hz, 1 H, H-13), 4.91 (d, *J* = 11.0 Hz, 1 H, H-3), 4.06 (d, *J* = 7.3 Hz, 1 H, H-1′), 3.77 (s, 1 H, H-11), 3.74 (d, *J* = 2.7 Hz, 1 H, H-5), 3.63-3.55 (m, 1 H, H-cyclopropyl), 3.44-3.30 (m, 2 H, -C**H**$_2$-NHCO, H-5′), 3.27-3.19 (m, 1 H, H-2′), 3.09-2.99 (m, 1H, -C**H**$_2$-NHCO), 2.79 (s, 3 H, 6-*O*-CH$_3$), 2.93-2.80 (m, 1 H, H-10, H-2), 2.77-2.68 (m, 1 H, H-3′), 2.49 (t, *J* = 7.1 Hz, 3 H, H-8, -C**H**$_2$-C≡C-), 2.43 (s, 6 H, N(CH$_3$)$_2$), 2.11-2.03 (m, 1 H, H-4), 1.97-1.87 (m, 1 H, H-14eq), 1.84-1.75 (m, 1 H, H-7a), 1.74-1.62 (m, 3 H, H-4′a, -C**H**$_2$-CH$_2$NHCO), 1.62-1.47 (m, 6 H, H-7b, -C≡CCH$_2$-C**H**$_2$-C**H**$_2$-CH$_2$-, H-14ax), 1.47-1.36 (m, 7 H, -C**H**$_2$-, H-14ax, 12-CH$_3$, 2H-cyclopropyl), 1.27 (s, 3 H, 6-CH$_3$), 1.25-1.19 (m, 6H, 5′-CH$_3$, 10-CH$_3$), 1.18-1.11 (m, 9H, H-4′b, 2-CH$_3$, 8-CH$_3$, 2H-cyclopropyl), 1.09 (d, *J* = 7.1 Hz, 3H, 4-CH$_3$), 0.84 (t, *J* = 7.4 Hz, 3H, 15-CH$_3$). ^{13}C NMR (CDCl$_3$, 100 MHz) δ: 217.6, 178.2, 174.4, 166.8, 158.4, 156.5, 148.5, 141.1, 130.1, 129.4, 126.9, 124.8, 119.8, 109.0, 103.2, 95.8, 83.9, 82.5, 79.9, 78.2, 77.8, 77.2, 75.8, 70.5, 69.1, 65.6, 58.2, 49.8, 45.3, 43.4, 41.0, 39.9, 39.1, 37.3, 35.8, 35.4, 30.1, 29.2, 28.7, 28.3, 26.3, 22.0, 21.1, 19.5, 19.3, 18.3, 15.0, 13.9, 13.3, 10.3, 9.1, 8.4.

3-O-descladinosyl-3-O-[N-8-(1,4-dihydro-1-ethyl-4-oxo-3-quinolinecarboxylic acid-7-yl)-otc-7-ynylcarbamoyl]

clarithromycin-11,12- cyclic carbamate ***(88f)***

Following the general procedure I-2, from compound **85** (0.200 g, 0.229 mmol) and **10** (0.102 g, 0.275 mmol), ethyl ester-**88f** was obtained. After completion of this reaction, ethyl ester-**88f** was treated with LiOH·H_2O to remove the protective group, **88f** (37.7 mg, 0.0384 mmol, 16.8%) was obtained as an off-white solid (column chromatography eluents: DCM/MeOH/NH_3·H_2O = 10:0.9:0.5). m.p. 167.2-168.3 ˚C. HRMS (ESI) $(M+H)^+$ *m/z* 981.5443, calcd for $C_{52}H_{77}N_4O_{14}$ 981.5431. ^{1}H NMR (CD_3OD, 400 MHz) δ: 8.87 (s, 1 H, 2″-quinolyl), 8.34 (d, *J* = 8.3 Hz, 1 H, 5″-quinolyl), 7.80 (s, 1 H, 8″-quinolyl), 7.43 (s, 1 H, 6″-quinolyl), 5.15 (dd, *J* = 2.5 Hz, 10.7 Hz, 1 H, H-13), 4.87 (d, *J* = 11.1 Hz, 1 H, H-3), 4.45 (br, 2 H, NC**H2**CH_3), 4.14 (d, *J* = 7.2 Hz, 1 H, H-1′), 3.84 (d, *J* = 3.1 Hz, 1 H, H-5), 3.80 (s, 1 H, H-11), 3.84-3.36 (m, 1 H, H-5′), 3.29-3.20 (m, 2 H, H-2′, 3-*O*-CO-NH-C**H**$_2$), 3.11-3.01 (m, 2 H, H-10, 3-*O*-CO-NH-C**H**$_2$), 2.97 (s, 3 H, 6-*O*-CH_3), 2.92-2.81 (m, 1 H, H-2), 2.79-2.66 (m, 1 H, H-3′), 2.55-2.47 (m, 3 H, -CH_2-C≡C-, H-8), 2.41 (s, 6 H, -N(CH_3)$_2$), 2.16-2.05 (m, 1 H, H-4), 1.90-1.73 (m, 3 H, H-14ax, H-7a, H-4′a), 1.69-1.48 (m, 9 H, 3(-CH_2-), H-7b, H-14eq, NCH_2C**H3**), 1.46 (s, 3 H, 12-CH_3), 1.32-1.26 (m, 3 H, -CH_2-, H-4′b), 1.24 (s, 3 H, 6-CH_3), 1.18 (d, *J* = 6.0 Hz, 3 H, 5′-CH_3), 1.16-1.08 (m, 12 H, 2-CH_3, 4-CH_3, 10-CH_3, 8-CH_3), 0.84 (t, *J* = 7.3 Hz, 3 H, 15-CH_3). ^{13}C NMR (CD_3OD, 100 MHz) δ: 217.95, 174.81, 159.42, 157.39, 101.70, 84.50, 79.48, 78.80, 77.88, 77.44, 75.49, 70.68, 68.67, 64.57, 58.06, 48.93, 45.34, 43.24, 40.49, 39.51, 38.68, 37.41, 35.68, 30.89, 29.44, 28.30, 28.11, 26.02, 21.80, 20.00, 18.69, 18.43, 17.17, 13.98, 13.60, 12.92, 12.22, 9.42, 8.07.

3-O-descladinosyl-3-O-[N-8-(1,4-dihydro-1-methyl-4-oxo-3-quinolinecarboxylic acid-7-yl)-otc-7-ynylcarbamoyl] clarithromycin-11,12- cyclic carbamate ***(88g) MCX-219***

Following the general procedure I-1, from compound **85** (0.361 g, 0.414 mmol) and **11** (0.177 g, 0.497 mmol), **88g** (43.2 mg, 0.0447 mmol, 10.8%) was obtained as an off-white solid (column chromatography eluents: DCM/MeOH/NH_3·H_2O=10:0.9:0.5). m.p. 179.5-180.3 ˚C. HRMS (ESI) $(M+H)^+$ *m/z* 967.5287, calcd for $C_{51}H_{75}N_4O_{14}$ 967.5274. HPLC purity: 96.0% (t= 7.41 min). ^{1}H NMR (CD_3OD, 400 MHz) δ: 8.82 (s, 1 H, 2″-quinolyl), 8.32 (d, *J* = 8.4 Hz, 1 H, 5″-quinolyl), 7.72 (s, 1 H, 8″-quinolyl), 7.42 (s, 1 H, 6″-quinolyl), 5.16 (dd, *J* = 2.4 Hz, 10.7 Hz, 1 H, H-13), 4.87 (d, *J* = 11.1 Hz, 1 H, H-3), 4.13 (d, *J* = 7.3 Hz, 1 H, H-1′), 3.99 (s, 3 H, N-CH_3), 3.84 (d, *J* = 3.1 Hz, 1 H, H-5), 3.79 (m, 1 H, H-11), 3.46-3.35 (m, 1 H, H-5′), 3.28-3.20 (m, 2 H, H-2′, 3-*O*-CO-NH-C**H**$_2$), 3.10-3.00 (m, 2 H, H-10, 3-*O*-CO-NH-C**H**$_2$), 2.97 (s, 3 H, 6-*O*-CH_3), 2.92-2.83 (m, 1 H, H-2), 3.77-3.67 (m, 1 H, H-3′), 2.57-2.47 (m, 3 H, H-8,-CH_2-C≡C-), 2.40 (s, 6 H, -N(CH_3)$_2$), 2.14-2.06 (m, 1 H, H-4), 1.91-1.71 (m, 3 H, H-14ax, H-7a, H-4′a), 1.70-1.49 (m, 8 H, 3(-CH_2-), H-7b, H-14eq), 1.46 (s, 3 H, 12-CH_3), 1.44-1.39 (m, 3 H, -CH_2-), 1.30-1.26 (m, 1 H, H-4′b), 1.25 (s, 3 H, 6-CH_3), 1.17 (d, *J* = 6.1 Hz, 3 H, 5′-CH_3), 1.15-1.07 (m, 12 H, 2-CH_3, 4-CH_3, 10-CH_3, 8-CH_3), 0.84 (t, *J* = 7.4 Hz, 3 H, 15-CH_3). ^{13}C NMR (CD_3OD, 100 MHz) δ: 217.96, 174.81, 159.41, 157.39, 101.73, 84.49, 79.48, 78.81, 77.89, 77.45, 75.49, 70.72, 68.70, 64.54, 58.06, 48.92, 45.34, 43.24, 40.93, 40.49, 39.53, 38.68, 37.41, 35.68, 30.89, 29.42, 28.28, 28.12, 26.01, 21.80, 20.01, 18.68, 18.43, 17.17, 13.99, 12.92, 12.22, 9.42, 8.07.

3-O-descladinosyl-3-O-[N-8-(ethyl 1,4-dihydro-1-methyl-4-oxo-3-quinolinecarboxylate-7-yl)-otc-7-ynylcarbamoyl] clarithromycin-11,12- cyclic carbamate ***(88h)***

Following the general procedure I-2, from compound **85** (0.199 g, 0.228 mmol) and **9** (0.0974 g, 0.342 mmol), **88h** (78.2 mg, 0.0786 mmol, 34.5%) was obtained as an off-white solid (column chromatography eluents: DCM/EtOH/NH_3·H_2O=10:0.2:0.1). m.p. 141.1-142.0 ˚C. HRMS (ESI) $(M+H)^+$ *m/z* 995.5598, calcd for $C_{53}H_{79}N_4O_{14}$ 995.5587. HPLC purity: 97.7% (t= 13.7 min). ^{1}H NMR (CD_3OD, 400 MHz) δ: 8.68 (s, 1 H, 2″-quinolyl), 8.30 (d, *J* = 8.4 Hz, 1 H, 5″-quinolyl), 7.69 (d, *J* = 1.4 Hz, 1 H, 8″-quinolyl), 7.45 (dd, *J* = 1.4 Hz, 8.4 Hz, 1 H, 6″-quinolyl), 5.17 (dd, *J* = 2.5 Hz, 10.7 Hz, 1 H, H-13), 4.87 (d, *J* = 11.0 Hz, 1 H, H-3), 4.32 (q, *J* = 7.1 Hz, 2 H, OC**H2**CH_3), 4.10 (d, *J* = 7.3 Hz, 1 H, H-1′), 3.95 (s, 3 H, N-CH_3), 3.85 (d, *J* = 3.2 Hz, 1 H, H-5), 3.80 (m, 1 H, H-11), 3.44-3.36 (m, 1 H, H-5′), 3.28-3.19 (m, 2 H, H-2′, 3-*O*-CO-NH-C**H**$_2$), 3.12-2.99 (m, 2 H, H-10, 3-*O*-CO-NH-C**H**$_2$), 2.97 (s, 3 H, 6-*O*-CH_3), 2.92-2.84 (m, 1 H, H-2), 2.62-2.45 (m, 4 H, H-3′, H-8,-CH_2-C≡C-), 2.31 (s, 6 H, -N(CH_3)$_2$), 2.14-2.06 (m, 1 H, H-4), 1.91-1.78 (m, 2 H, H-14ax, H-7a), 1.73-1.49 (m, 9 H, 3(-CH_2-), H-7b, H-14eq, H-4′a), 1.46 (s, 3 H, 12-CH_3), 1.45-1.40 (m, 3 H, -CH_2-, H-4′b), 1.37 (q, *J* = 7.1 Hz, 3 H, OCH_2C**H3**), 1.25 (s, 3 H, 6-CH_3), 1.18 (d, *J* = 6.1 Hz, 3 H, 5′-CH_3), 1.16-1.09 (m, 12 H, 2-CH_3, 4-CH_3, 10-CH_3, 8-CH_3), 0.84 (t, *J* = 7.4 Hz, 3 H, 15-CH_3). ^{13}C NMR

(CD_3OD, 100 MHz) δ: 217.98, 174.82, 164.67, 159.43, 157.39, 150.68, 140.09, 129.12, 128.06, 126.88, 126.46, 119.33, 109.92, 101.91, 94.17, 84.50, 79.52, 78.82, 77.91, 77.50, 75.48, 70.93, 68.81, 64.41, 60.20, 58.07, 48.90, 45.34, 43.27, 40.61, 40.51, 39.58, 38.69, 37.41, 35.70, 30.85, 29.41, 28.28, 28.13, 26.02, 21.80, 20.05, 18.66, 18.43, 17.15, 13.98, 13.31, 12.91, 12.22, 9.40, 8.08.

*3-O-descladinosyl-3-O-[N-8-(1,4-dihydro-4-oxo-3-quinolinecarboxylic acid-7-yl)-otc-7-ynylcarbamoyl] clarithromycin-11,12- cyclic carbamate **(88i)***

Following the general procedure I-1, from compound **85** (0.217 g, 0.249 mmol) and **12** (0.0941 g, 0.299 mmol), **88i** (31.4 mg, 0.0329 mmol, 13.23%) was obtained as an off-white solid (column chromatography eluents: DCM/MeOH/$NH_3 \cdot H_2O$ = 10:0.9:0.5). m.p. 162.9-166.0 ˚C. HRMS (ESI) $(M+H)^+$ *m/z* 953.5132, calcd for $C_{50}H_{73}N_4O_{14}$ 953.5118. ^1H NMR ($CDCl_3$, 400 MHz) δ: 8.92 (s, 1 H, 2″-quinolyl), 8.26 (d, *J* = 8.4 Hz, 1 H, 5″-quinolyl), 7.70 (s, 1 H, 8″-quinolyl), 7.42 (d, *J* = 8.4 Hz, 1 H, 6″-quinolyl), 5.88 (s, 1 H, 11-NH), 5.54 (s, 1 H, 3-*O*-CO-N**H**-CH_2), 5.20 (dd, *J* = 2.4 Hz, 10.9 Hz, 1 H, H-13), 4.89 (d, *J* = 11.0 Hz, 1 H, H-3), 4.09 (d, *J* = 7.2 Hz, 1 H, H-1′), 3.79 (d, *J* = 2.8 Hz, 1 H, H-5), 3.76 (s, 1 H, H-11), 3.48-3.37 (m, 1 H, H-5′), 3.36-3.24 (m, 2 H, H-2′, 3-*O*-CO-NH-C**H**$_2$), 3.19-3.04 (m, 2 H, NH, 3-*O*-CO-NH-C**H**$_2$), 2.97 (s, 3 H, 6-*O*-CH_3), 2.89 (q, *J* = 6.5 Hz, 1 H, H-10), 2.81-2.63 (m, 2 H, H-2, H-3′), 2.54-2.46 (m, 3 H, H-8, -C**H**$_2$-C≡C-), 2.38 (s, 6 H, -N(CH_3)$_2$), 2.09-2.00 (m, 1 H, H-4), 1.94-1.81 (m, 1 H, H-14ax), 1.80-1.67 (m, 2 H, H-14eq, H-7a), 1.67-1.42 (m, 10 H, H-7b, H-4′a , 4(-CH_2-)), 1.40 (s, 3 H, 12-CH_3), 1.31-1.18 (m, 7 H, 6-CH_3, H-4′b, 5′-CH_3), 1.18-1.02 (m, 12 H, 2-CH_3, 10-CH_3, 8-CH_3, 4-CH_3), 0.82 (t, *J* = 7.3 Hz, 3 H, 15-CH_3). ^{13}C NMR ($CDCl_3$, 100 MHz) δ: 217.61, 174.49, 158.57, 156.52, 129.31, 128.79, 125.45, 123.45, 108.69, 102.74, 84.05, 81.39, 78.18, 77.85, 77.23, 75.77, 70.68, 69.20, 65.73, 58.20, 49.81, 45.30, 43.41, 41.05, 40.13, 39.11, 37.29, 35.73, 29.93, 29.69, 28.95, 28.32, 27.92, 26.19, 22.03, 21.13, 19.30, 18.24, 15.02, 13.87, 13.27, 10.23, 9.09.

*3-O-descladinosyl-3-O-[N-8-(3-quinolinecarboxylic acid-7-yl)-otc-7-ynylcarbamoyl] clarithromycin-11,12- cyclic carbamate **(88j)***

Following the general procedure I-2, from compound **85** (0.280 g, 0.321 mmol) and methyl 7-bromoquinoline-3-carboxylate (0.0939 g, 0.353 mmol), methyl ester-**88j** was obtained. After completion of this reaction, methyl ester-**88j** was dissolved in THF/H_2O (5 mL/5 mL) and treated with LiOH·H_2O to remove the protective group. After then **88j** (28.7 mg, 0.0306 mmol, 9.54 %) was obtained as an off-white solid (column chromatography eluents: DCM/MeOH/$NH_3 \cdot H_2O$ = 10:1:0.5). m.p. 163.9-165.2 ˚C. HRMS (ESI) $(M+H)^+$ *m/z* 937.5165, calcd for $C_{50}H_{73}N_4O_{13}$ 937.5169. ^1H NMR ($CDCl_3$, 400 MHz) δ: 9.52 (d, *J* = 2.0 Hz, 1 H, 2″-quinolyl), 8.74 (d, *J* = 2.0 Hz, 1 H, 4″-quinolyl), 8.12 (s, 1 H, 8″-quinolyl), 7.79 (d, *J* = 8.4 Hz, 1 H, 5″-quinolyl), 7.45 (dd, *J* = 1.6 Hz, 8.4 Hz, 1 H, 6″-quinolyl), 6.91 (dd, *J* = 4.5 Hz, 6.9 Hz, 1 H, 3-*O*-CO-N**H**-CH_2), 5.81 (s, 1 H, 11-NH), 5.25 (dd, *J* = 2.4 Hz, 10.8 Hz, 1 H, H-13), 4.97 (d, *J* = 11.0 Hz, 1 H, H-3), 4.20 (d, *J* = 7.2 Hz, 1 H, H-1′), 3.79 (m, 1 H, H-11), 3.68 (d, *J* = 2.1 Hz, 1 H, H-5), 3.54-3.42 (m, 2 H, 3-*O*-CO-NH-C**H**$_2$, H-5′), 3.41-3.31 (m, 2 H, H-2′, 3-*O*-CO-NH-C**H**$_2$), 2.98 (s, 3 H, 6-*O*-CH_3), 2.95-2.83 (m, 3 H, H-10, H-2, H-3′), 2.77 (s, 6 H, -N(CH_3)$_2$), 2.57-2.47 (m, 2 H, H-8), 2.28-2.16 (m, 1 H, -C**H**$_2$-C≡C-), 2.15-2.00 (m, 2 H, H-4, -C**H**$_2$-C≡C-), 1.98-1.79 (m, 3 H, H-14ax, H-7a, H-4′a), 1.62-1.50 (m, 2 H, H-7b, H-14eq,), 1.45 (s, 3 H, 12-CH_3), 1.43-1.18 (m, 21 H, 6-CH_3, H-4′b, 5′-CH_3, 2-CH_3, 4-CH_3, 4(CH_2)), 1.15 (d, *J* = 6.5 Hz, 3 H, 10-CH_3), 1.09 (d, *J* = 7.1 Hz, 3 H, 8-CH_3), 0.87 (t, *J* = 7.3 Hz, 3 H, 15-CH_3). ^{13}C NMR ($CDCl_3$, 100 MHz) δ: 217.62, 174.60, 172.47, 158.52, 156.84, 152.10, 148.80, 137.36, 131.88, 130.02, 128.57, 128.10, 126.69, 126.48, 103.58, 93.31, 84.84, 84.05, 80.29, 78.33, 77.75, 77.23, 75.72, 70.76, 68.39, 64.48, 58.32, 49.67, 45.38, 43.63, 41.18, 39.18, 38.81, 37.36, 35.77, 30.25, 29.83, 29.70, 28.91, 28.47, 26.48, 22.13, 21.12, 19.46, 19.21, 18.24, 15.07, 13.92, 13.34, 10.26, 9.39.

*3-O-descladinosyl-3-O-[N-8-(quinazolin-4(3H)-one-7-yl)-otc-7-ynylcarbamoyl] clarithromycin-11,12- cyclic carbamate **(88k)***

Following the general procedure I-2, from compound **85** (0.200 g, 0.229 mmol) and **14** (0.0616 g, 0.275 mmol), **88i** (60.1 mg, 0.0660 mmol, 28.8%) was obtained as an off-white solid (column chromatography eluents: DCM/EtOH/$NH_3 \cdot H_2O$=10:0.3:0.1). m.p. 148.9-149.8 ˚C. HRMS (ESI) $(M+H)^+$ *m/z* 910.5155, calcd for $C_{48}H_{72}N_5O_{12}$

910.5172. ^{1}H NMR ($CDCl_3$, 400 MHz) δ: 8.12 (d, *J* = 8.2 Hz, 1 H, 5″-quinolyl), 8.02 (s, 1 H, 2″-quinolyl), 7.67 (d, *J* = 1.4 Hz, 1 H, 8″-quinolyl), 7.41 (dd, *J* = 1.5 Hz, 8.2 Hz, 1 H, 6″-quinolyl), 5.81 (s, 1 H, 11-NH), 5.16 (dd, *J* = 2.3 Hz, 10.9 Hz, 1 H, H-13), 5.05 (t, *J* = 5.8 Hz, 1 H, 3-*O*-CO-N**H**-CH_2), 4.85 (d, *J* = 11.0 Hz, 1 H, H-3), 3.97 (d, *J* = 7.3 Hz, 1 H, H-1′), 3.74-3.68 (m, 1 H, H-11, H-5), 3.36-3.21 (m, 2 H, 3-*O*-CO-NH-C**H**$_2$, H-5′), 3.12 (dd, *J* = 7.4 Hz, 10.2 Hz, 1 H, H-2′), 3.06-2.96 (m, 1 H, 3-*O*-CO-NH-C**H**$_2$), 2.91 (s, 3 H, 6-*O*-CH_3), 2.83 (q, *J* = 6.8 Hz, 1 H, H-10), 2.80-2.72 (m, 1 H, H-2), 2.49-2.41 (m, 4 H, H-8, H-3′, -C**H**$_2$-C≡C-), 2.20 (s, 6 H, -N(CH_3)$_2$), 2.06-1.69 (m, 1 H, H-4), 1.89-1.80 (m, 1 H, H-14ax), 1.76-1.66 (m, 1 H, H-7a), 1.62-1.38 (m, 9 H, H-4′a, 3(CH_2), H-7b, H-14eq), 1.36 (s, 3 H, 12-CH_3), 1.35-1.28 (m, 2 H, CH_2), 1.20 (s, 3 H, 6-CH_3), 1.19-1.16 (m, 1 H, H-4′b), 1.14 (d, *J* = 6.2 Hz, 3 H, 5′-CH_3),1.11-0.98 (m, 12 H, 2-CH_3, 4-CH_3, 10-CH_3, 8-CH_3), 0.77 (t, *J* = 7.3 Hz, 3 H, 15-CH_3). ^{13}C NMR ($CDCl_3$, 100 MHz) δ: 217.65, 174.38, 162.29, 158.53, 156.40, 148.85, 144.24, 130.83, 130.43, 130.27, 126.29, 121.54, 103.20, 94.65, 83.98, 81.72, 79.98, 78.25, 77.85, 77.24, 75.74, 75.53, 70.60, 69.50, 65.97, 58.18, 49.83, 45.31, 43.41, 41.15, 40.25, 39.15, 37.30, 35.75, 30.09, 28.58, 28.35, 26.37, 22.04, 21.19, 19.48, 19.31, 18.28, 15.02, 13.89, 13.30, 10.24, 9.10.

3-O-descladinosyl-3-O-[N-8-(3-carboxylmethylquinazolin-4(3H)-one-7-yl)-otc-7-ynylcarbamoyl] clarithromycin-11,12- cyclic carbamate ***(88l)***

Following the general procedure I-2, from compound **85** (0.200 g, 0.229 mmol) and **15** (0.0814 g, 0.275 mmol), methyl ester-**88l** was obtained. After reaction, methyl ester-**88l** was treated with LiOH·H_2O to remove the protective group, and **88l** (51.2 mg, 0.0529 mmol, 17.7%) was obtained as an off-white solid (column chromatography eluents: DCM/MeOH/NH_3·H_2O = 10:1:0.5). m.p. 184.9-185.0 ˚C. HRMS (ESI) $(M+H)^+$ *m/z* 968.5214, calcd for $C_{50}H_{74}N_5O_{14}$ 968.5227. ^{1}H NMR ($CDCl_3$, 400 MHz) δ: 8.08 (d, *J* = 8.2 Hz, 1 H, 5″-quinolyl), 8.00 (s, 1 H, 2″-quinolyl), 7.59 (d, *J* = 1.5 Hz, 1 H, 8″-quinolyl), 7.35 (dd, *J* = 1.5 Hz, 8.2 Hz, 1 H, 6″-quinolyl), 6.57 (t, *J* = 5.8 Hz, 1 H, 3-*O*-CO-N**H**-CH_2), 5.83 (s, 1 H, 11-NH), 5.24 (dd, *J* = 2.4 Hz, 10.8 Hz, 1 H, H-13), 4.89 (d, *J* = 11.0 Hz, 1 H, H-3), 4.62-4.48 (m, C**H**$_2$COOH), 4.11 (d, *J* = 7.2 Hz, 1 H, H-1′), 3.62 (s, 1 H, H-11), 3.62 (d, *J* = 2.24 Hz, 1 H, H-5), 3.51-3.41 (m, 1 H, H-5′), 3.41-3.32 (m, 1 H, H-3′), 3.31-3.24 (m, 1 H, H-2′), 3.23-3.14 (m, 1 H, 3-*O*-CO-NH-C**H**$_2$), 3.05-2.98 (m, 1 H, 3-*O*-CO-NH-C**H**$_2$), 2.91 (s, 3 H, 6-*O*-CH_3), 2.97 (q, *J* = 6.8 Hz, 1 H, H-10), 2.87-2.78 (m, 1 H, H-2), 2.59 (s, 6 H, -N(CH_3)$_2$), 2.54-2.40 (m, 3 H, H-8, -C**H**$_2$-C≡C-), 2.12-2.02 (m, 1 H, H-4), 2.00-1.88 (m, 1 H, H-14ax), 1.87-1.45 (m, 1 H, H-7a, H-4′a), 1.67-1.40 (m, 11 H, 3(CH_2), H-7b, H-14eq, 12-CH_3), 1.38-1.22 (m, 9 H, CH_2, 6-CH_3, H-4′b, 5′-CH_3),1.20-1.06 (m, 12 H, 2-CH_3, 4-CH_3, 10-CH_3, 8-CH_3), 0.87 (t, *J* = 7.4 Hz, 3 H, 15-CH_3). ^{13}C NMR ($CDCl_3$, 100 MHz) δ: 217.59, 174.59, 174.26, 160.74, 158.50, 156.71, 148.18, 148.04, 130.11, 129.98, 129.91, 126.30, 120.93, 103.10, 94.66, 84.33, 84.03, 79.89, 77.99, 77.68, 77.24, 75.63, 70.55, 68.18, 64.56, 58.25, 50.00, 49.72, 45.35, 43.51, 41.11, 39.10, 37.33, 35.66, 30.45, 30.07, 28.60, 28.29, 26.32, 22.11, 21.07, 19.43, 19.18, 18.27, 14.97, 13.93, 13.34, 10.25, 9.25.

3-O-descladinosyl-3-O-[N-8-(1,3-dihydroxy-9H-xanthen-9-one-6-yl)-otc-7-ynylcarbamoyl] clarithromycin-11,12-cyclic carbamate ***(88m)***

Following the general procedure I-1, from compound **85** (0.100 g, 0.115 mmol) and **18** (0.049 g, 0.138 mmol), **88i** (58.0 mg, 0.0488 mmol, 42.4%) was obtained as an off-white solid (column chromatography eluents: DCM/MeOH/NH_3·H_2O=10:0.9:0.1). HRMS (ESI) $(M+H)^+$ *m/z* 992.5115, calcd for $C_{53}H_{74}N_3O_{15}$ 992.5114. m.p. 158.6-159.8 ˚C. ^{1}H NMR ($CDCl_3$, 400 MHz) δ: 12.87 (s, 1H, H-Xanthone), 8.10 (d, *J* = 8.2 Hz, 1H, H-Xanthone), 7.36 (d, *J* = 1.4 Hz, 1H, H-Xanthone), 7.33-7.30 (m, 1H, H-Xanthone), 6.40 (s, 1H, H-Xanthone), 6.35 (s, 1H, H-Xanthone), 5.89 (s, 1H, 11,12-OCON**H**), 5.31-5.21 (m, 2 H, H-13, 3-OCON**H**), 4.95 (d, *J* = 11.0 Hz, 1H, H-3), 4.10 (d, *J* = 7.2 Hz, 1H, H-1′), 3.84 (d, *J* = 2.9 Hz, 1 H, H-5), 3.81 (s, 1H, H-11), 3.48-3.37 (m, 2 H, H-5′, -NH-C**H**$_2$-), 3.30 (dd, *J* = 10.2, 7.1 Hz, 1H, H-2′), 3.16-3.07 (m, 1H, -NH-C**H**$_2$-), 3.01 (s, 3 H, 6-O-CH_3), 2.93 (q, *J* = 6.5 Hz, 1 H, H-10), 2.84 (dd, *J* = 11.2 Hz, *J* = 6.6 Hz, 1 H, H-2), 2.77-2.68 (m, 1 H, H-3′), 2.57-2.51 (m, 1 H, H-8), 2.50-2.35 (m, 8 H, -N(CH_3)$_2$, -**CH$_2$**-Ar), 2.13-2.07 (m, 1 H, H-4), 1.97-1.87 (m, 1 H, H-14eq), 1.85-1.74 (m, 2 H, H-7a, H-4′a), 1.68-1.50 (m, 8 H, H-14ax, H-7b, -(CH_2)$_3$-), 1.49-1.40 (m, 5 H, 12-CH_3, -CH_2-), 1.36-1.31 (m, 1 H, H-4′b), 1.29 (s, 3 H, 6-CH_3), 1.25 (d, *J* = 6.0 Hz, 3 H, 5′-CH_3), 1.18 (dd, *J* = 6.7, 4.8 Hz, 6 H, 4-CH_3, 2-CH_3), 1.15-1.07 (m, 6 H, 8-

CH_3, 10-CH_3), 0.85 (t, *J* = 7.3 Hz, 3 H, 15-CH_3). ^{13}C NMR ($CDCl_3$, 100MHz) δ: 217.77, 179.96, 174.44, 165.40, 163.57, 158.72, 157.80, 156.53, 155.58, 130.83, 127.03, 125.58, 120.19, 119.56, 103.37, 102.63, 98.95, 95.30, 94.62, 84.18, 81.31, 79.88, 70.58, 69.23, 65.63, 58.18, 49.95, 45.28, 43.33, 41.05, 40.22, 39.09, 37.29, 35.67, 29.92, 29.44, 28.42, 28.20, 26.14, 21.99, 21.09, 19.41, 19.32, 18.30, 15.07, 13.93, 13.27, 10.23, 9.14.

3-O-descladinosyl-3-O-[N-9-(1,4-dihydro-1-methyl-4-oxo-3-quinolinecarboxylic acid-7-yl)-non-8-ynylcarbamoyl] clarithromycin-11,12- cyclic carbamate ***(89g)***

Following the general procedure I-1, from compound **86** (0.341 g, 0.350 mmol) and **11** (0.151 g, 0.421 mmol), **88i** (52.2 mg, 0.0532 mmol, 15.2%) was obtained as an off-white solid (column chromatography eluents: DCM/MeOH/$NH_3 \cdot H_2O$=10:0.9:0.5). HRMS (ESI) $(M+H)^+$ *m/z* 981.5438, calcd for $C_{52}H_{77}N_4O_{14}$ 981.5431. ^1H NMR (CD_3OD, 400 MHz) δ: 8.79 (s, 1 H, 2″-quinolyl), 8.29 (d, *J* = 8.4 Hz, 1 H, 5″-quinolyl), 7.71 (s, 1 H, 8″-quinolyl), 7.43 (s, 1 H, 6″-quinolyl), 5.15 (dd, *J* = 2.5 Hz, 10.7 Hz, 1 H, H-13), 4.86 (d, *J* = 11.3 Hz, 1 H, H-3), 4.13 (d, *J* = 7.2 Hz, 1 H, H-1′), 3.99 (s, 3 H, N-CH_3), 3.83 (d, *J* = 3.1 Hz, 1 H, H-5), 3.78 (m, 1 H, H-11), 3.47-3.38 (m, 1 H, H-5′), 3.29-3.20 (m, 2 H, H-2′, 3-*O*-CO-NH-C**H**$_2$), 3.10-2.99 (m, 2 H, H-10, 3-*O*-CO-NH-C**H**$_2$), 2.98 (s, 3 H, 6-*O*-CH_3), 2.90-2.80 (m, 1 H, H-8), 3.79-3.69 (m, 1 H, H-3′), 2.54-2.45 (m, 2 H, -CH_2-C≡C-), 2.42 (s, 6 H, -N$(CH_3)_2$), 2.13-2.04 (m, 1 H, H-4), 1.90-1.72 (m, 3 H, H-14ax, H-7a, H-4′a), 1.70-1.47 (m, 8 H, 3(-CH_2-), H-7b, H-14eq), 1.45 (s, 3 H, 12-CH_3), 1.42-1.27 (m, 5 H, 2(-CH_2-), H-4′b), 1.24 (s, 3 H, 6-CH_3), 1.18 (d, *J* = 6.1 Hz, 3 H, 5′-CH_3), 1.15-1.07 (m, 12 H, 2-CH_3, 4-CH_3, 10-CH_3, 8-CH_3), 0.83 (t, *J* = 7.4 Hz, 3 H, 15-CH_3). ^{13}C NMR (CD_3OD, 100 MHz) δ: 217.96, 174.79, 159.39, 157.37, 101.69, 84.48, 79.40, 78.80, 77.88, 77.42, 75.47, 70.72, 68.68, 64.54, 58.06, 48.95, 45.34, 43.22, 40.99, 40.50, 39.54, 38.69, 37.41, 35.67, 30.93, 29.52, 28.57, 28.50, 28.10, 26.38, 21.80, 20.02, 18.75, 18.44, 17.19, 14.01, 12.94, 12.24, 9.44, 8.09.

3-O-descladinosyl-3-O-[N-8-(1,4-dihydro-1-methyl-4-oxo-3-quinolinecarboxylic acid-7-yl)- octylcarbamoyl] clarithromycin-11,12- cyclic carbamate ***(90g)***

Following the general procedure E, from compound **88g** (0.100 g, 0.101 mmol), **90g** (12.9 mg, 0.0133 mmol, 13.2%) was obtained as an off-white solid (column chromatography eluents: DCM/MeOH/$NH_3 \cdot H_2O$=10:0.9:0.5). m.p. 147.5-148.0 ˚C. HRMS (ESI) $(M+H)^+$ *m/z* 971.5603, calcd for $C_{51}H_{79}N_4O_{14}$ 971.5587. ^1H NMR (CD_3OD, 400 MHz) δ: 8.83 (s, 1 H, 2″-quinolyl), 8.33 (d, *J* = 8.2 Hz, 1 H, 5″-quinolyl), 7.66 (s, 1 H, 8″-quinolyl), 7.48 (s, 1 H, 6″-quinolyl), 5.18 (dd, *J* = 2.4 Hz, 10.8 Hz, 1 H, H-13), 4.87 (d, *J* = 11.1 Hz, 1 H, H-3), 4.12 (d, *J* = 7.2 Hz, 1 H, H-1′), 3.99 (s, 3 H, N-CH_3), 3.84 (d, *J* = 3.1 Hz, 1 H, H-5), 3.80 (m, 1 H, H-11), 3.44-3.35 (m, 1 H, H-5′), 3.28-3.17 (m, 2 H, H-2′, 3-*O*-CO-NH-C**H**$_2$), 3.12-3.00 (m, 2 H, H-10, 3-*O*-CO-NH-C**H**$_2$), 2.98 (s, 3 H, 6-*O*-CH_3), 2.94-2.81 (m, 3 H, H-2, -CH_2-quinolyl), 3.74-3.62 (m, 1 H, H-3′), 2.53-2.44 (m, 1 H, H-8), 2.40 (s, 6 H, -N$(CH_3)_2$), 2.15-2.07 (m, 1 H, H-4), 1.92-1.80 (m, 2 H, H-14ax, H-7a), 1.79-1.66 (m, 3 H, H-4′a, -CH_2-), 1.66-1.50 (m, 4 H, -CH_2-, H-7b, H-14eq), 1.46 (s, 3 H, 12-CH_3), 1.43-1.22 (m, 12 H, 4(-CH_2-), H-4′b, 6-CH_3), 1.18 (d, *J* = 6.0 Hz, 3 H, 5′-CH_3), 1.15-1.07 (m, 12 H, 2-CH_3, 4-CH_3, 10-CH_3, 8-CH_3), 0.84 (t, *J* = 7.4 Hz, 3 H, 15-CH_3). ^{13}C NMR (CD_3OD, 100 MHz) δ: 217.96, 174.83, 159.43, 157.38, 101.74, 84.51, 78.80, 77.89, 77.45, 75.51, 70.69, 68.69, 64.55, 58.07, 48.91, 45.34, 43.24, 40.50, 39.48, 38.68, 37.42, 35.95, 35.70, 30.87, 29.52, 29.34, 29.04, 28.87, 28.83, 26.40, 21.81, 19.98, 18.42, 17.16, 13.96, 12.91, 12.22, 9.41, 8.05.

Synthesis of compounds 95-96 and 98

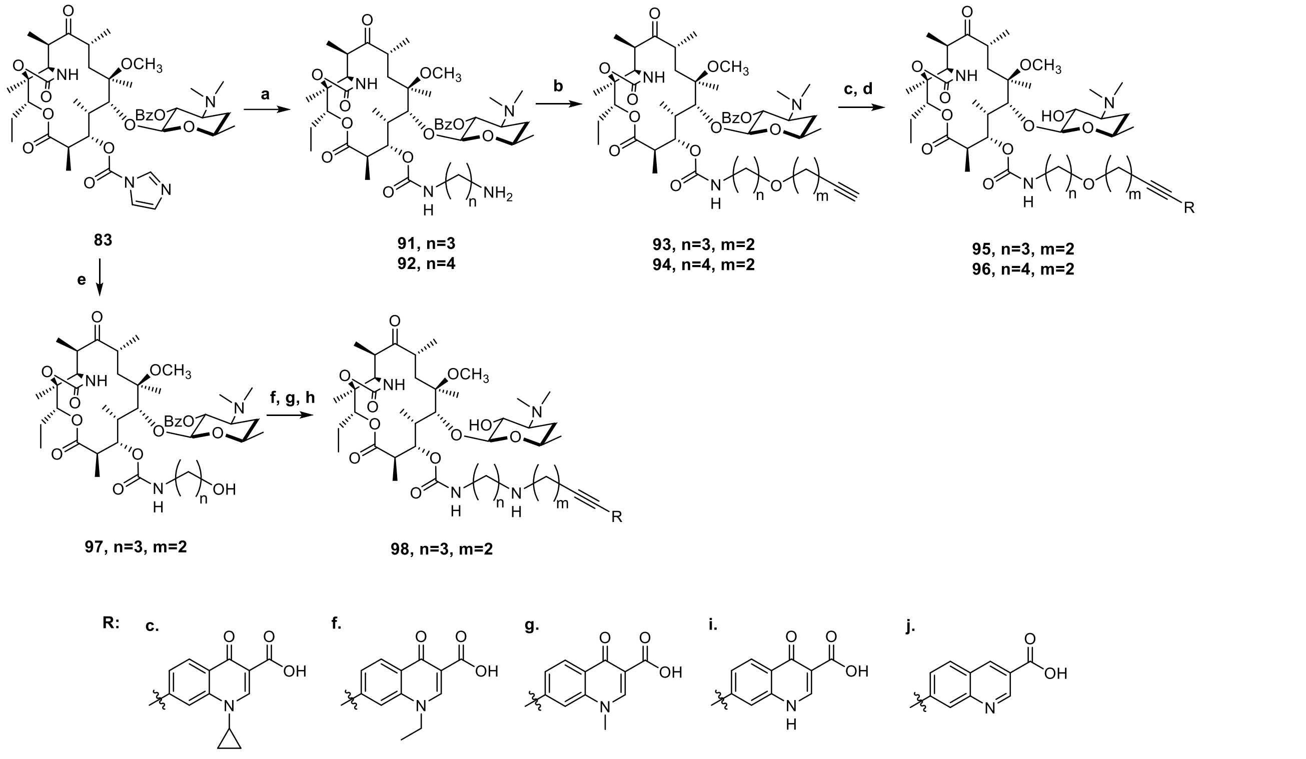

Scheme S11. Synthesis of *clarithromycin-11,12-cyclic carbamate* series compounds **95-96** and **98**. Reagents and conditions: (a) 1,3-diaminopropane or 1,4-butanediamine, DBU, DMF, rt, 3 h; (b) 3-butyn-1-ol, $NaNO_2$, HCOOH, -15˚C, 12-72 h; (c) R-I (R = **c**, **g**, **i**), CuI, $Pd(PPh_3)_2Cl_2$, Et_3N, CH_3CN, 45 ˚C, 12 h; or R-Br (R = **j**-ethyl ester) or R-I (R = **f**-ethyl ester), CuI, $Pd(PPh_3)_2Cl_2$, Et_3N, CH_3CN, 80 ˚C, 4 h; (d) CH_3OH, 65 ˚C, 12 h; (e) 1,3-diaminopropane, DBU, DMF, rt, 12 h; (f) methanesulfonyl chloride, Et_3N, DCM, rt, 10 min; (g) **34**, CH_3CN, 75 ˚C, 72 h; (h) CH_3OH, 65 ˚C, 12 h.

*2'-O-benzoyl-3-O-descladinosyl-3-O-(N-aminopropylcarbamoyl) clarithromycin-11,12- cyclic carbamate **(91)***

Following the general procedure K, from compound **83** (4.25 g, 4.98 mmol) and 1,3-diaminopropane (2.07 mL, 24.9 mmol), **91** (2.58 g, 3.60 mmol, 72.3 %) was obtained as an off-white solid (column chromatography eluents: DCM/EtOH/$NH_3{\cdot}H_2O$=10:0.9:0.1).

*2'-O-benzoyl-3-O-descladinosyl-3-O-(N-aminobutylcarbamoyl) clarithromycin-11,12- cyclic carbamate **(92)***

Following the general procedure K, from compound **83** (2.00 g, 2.63 mmol) and 1,4-butanediamine (1.33 mL, 13.2 mmol), **92** (1.18 g, 1.64 mmol, 62.5%) was obtained as an off-white solid (column chromatography eluents: DCM/EtOH/$NH_3{\cdot}H_2O$=10:0.9:0.1).

General procedure M

Compound **91**, **92** (1 eq) was dissolved in 3-butyn-1-ol (20-100 eq), and HCOOH (4.5 eq) was added dropwise. $NaNO_2$ (6 eq) was then added and the mixture was stirred at -15 ˚C for 24-48 h. After reaction, CH_2Cl_2 (50 mL) and water (50 mL) were added into the reaction mixture. The organic layer was washed with water and brine, and concentrated in vacuo. The crude mixture was purified by column chromatography on silica gel.

*2'-O-benzoyl-3-O-descladinosyl-3-O-(N-3-butynoxy-propylcarbamoyl) clarithromycin-11,12- cyclic carbamate **(93)***

Following the general procedure M, from compound **91** (2.58 g, 3.60 mmol), **93** (0.456 g, 0.522 mmol, 14.5%) was obtained as an off-white solid (column chromatography eluents: petroleum ether/DCM/EtOH/$NH_3{\cdot}H_2O$=4:6:0.1:0.1). HRMS (ESI) $(M+H)^+$ *m/z* 872.4890, calcd for $C_{46}H_{70}N_3O_{13}$ 872.4903. ^1H NMR ($CDCl_3$, 400 MHz) δ: 8.10-8.02 (m, 2 H, 2 H-Bz), 7.62-7.55 (m, 1 H, 1 H-Bz), 7.51-7.43 (m, 2 H, 2 H-Bz),

5.77 (s, 1 H, 11-NH), 5.45 (t, *J* = 5.7 Hz, 1 H, 3-*O*-CO-N**H**-CH_2), 5.18 (dd, *J* = 2.2 Hz, 10.9 Hz, 1 H, H-13), 5.00 (dd, *J* = 7.3 Hz, 10.5 Hz, 1 H, H-2′), 4.89 (d, *J* = 11.0 Hz, 1 H, H-3), 4.30 (d, *J* = 7.5 Hz, 1 H, H-1′), 3.83 (d, *J* = 3.3 Hz, 1 H, H-5), 3.70 (s, 1 H, H-11), 3.66-3.55 (m, 4 H, -C**H₂**-O-C**H₂**-), 3.51-3.39 (m, 2 H, H-5′, 3-*O*-CO-NH-C**H₂**), 3.37-3.28 (m, 1 H, 3-*O*-CO-NH-C**H₂**), 2.96 (s, 3 H, 6-*O*-CH_3), 2.89-2.70 (m, 3 H, H-3, H-10, H-2), 2.55-2.41 (m, 3 H, -C**H₂**-C≡CH, H-8), 2.27 (s, 6 H, -N(CH_3)$_2$), 2.05 (t, *J* = 2.6 Hz, 1 H, -CH_2-C≡C**H**), 1.98-1.81 (m, 4 H, H-4, H-14eq, CH_2), 1.79-1.70 (m, 1 H, H-4′a), 1.60-1.36 (m, 4 H, H-7a, H-7b, H-14eq, H-4′a), 1.31 (s, 3 H, 12-CH_3), 1.28-1.23 (m, 6 H, 6-CH_3, 5′-CH_3), 1.16-1.03 (m, 9 H, 2-CH_3, 10-CH_3, 8-CH_3), 0.81 (t, *J* = 7.4 Hz, 3 H, 15-CH_3), 0.74 (d, *J* = 7.5 Hz, 3 H, 4-CH_3).

2′-O-benzoyl-3-O-descladinosyl-3-O-(N-3-butynoxy-butylcarbamoyl) clarithromycin-11,12- cyclic carbamate ***(94)***

Following the general procedure M, from compound **92** (1.18 g, 1.64 mmol), **94** (0.320 g, 0.361 mmol, 22.0%) was obtained as an off-white solid (column chromatography eluents: petroleum ether/DCM/EtOH/$NH_3\cdot H_2O$=4:6:0.1:0.1). HRMS (ESI) $(M+H)^+$ *m/z* 886.5061, calcd for $C_{47}H_{72}N_3O_{13}$ 886.5060. ^1H NMR ($CDCl_3$, 400 MHz) δ: 8.10-7.98 (m, 2 H, 2 H-Bz), 7.62-7.55 (m, 1 H, 1 H-Bz), 7.51-7.42 (m, 2 H, 2 H-Bz), 5.76 (s, 1 H, 11-NH), 5.18 (dd, *J* = 2.4 Hz, 10.9 Hz, 1 H, H-13), 5.15-5.08 (m, 1 H, 3-*O*-CO-N**H**-CH_2), 4.98 (dd, *J* = 7.5 Hz, 10.4 Hz, 1 H, H-2′), 4.88 (d, *J* = 10.8 Hz, 1 H, H-3), 4.29 (d, *J* = 7.1 Hz, 1 H, H-1′), 3.83 (d, *J* = 3.2 Hz, 1 H, H-5), 3.70 (m, 1 H, H-11), 3.60-3.48 (m, 4 H, -C**H₂**-O-C**H₂**-),), 3.47-3.36 (m, 2 H, H-5′, 3-*O*-CO-NH-C**H₂**), 3.22-3.12 (m, 1 H, 3-*O*-CO-NH-C**H₂**), 2.96 (s, 3 H, 6-*O*-CH_3), 2.85-2.69 (m, 3 H, H-3, H-10, H-2), 2.52-2.43 (m, 1 H, -C**H₂**-C≡CH), 2.38-2.31 (m, 1 H, H-8), 2.27 (s, 6 H, -N(CH_3)$_2$), 2.00 (t, *J* = 2.7 Hz, 1 H, -CH_2-C≡C**H**), 1.97-1.82 (m, 2 H, H-4, H-14ax), 1.79-1.63 (m, 3 H, H-4′a, -CH_2-), 1.58-1.37 (m, 6 H, H-7a, H-7b, H-14eq, H-4′a, -CH_2-), 1.31 (s, 3 H, 12-CH_3), 1.29-1.23 (m, 6 H, 6-CH_3, 5′-CH_3), 1.15-1.04 (m, 9 H, 2-CH_3, 10-CH_3, 8-CH_3), 0.81 (t, *J* = 7.4 Hz, 3 H, 15-CH_3), 0.74 (d, *J* = 7.6 Hz, 3 H, 4-CH_3).

3-O-descladinosyl-3-O-{N-[4-(1-cyclopropyl-1,4-dihydro-4-oxo-3-quinolinecarboxylic acid-7-yl)]-but-3-ynoxy-propylcarbamoyl} clarithromycin-11,12- cyclic carbamate ***(95c)***

Following the general procedure I-1, from compound **93** (0.170 g, 0.195 mmol) and **4** (0.0831 g, 0.234 mmol), **95c** (25.6 mg, 0.0257 mmol, 13.2%) was obtained as an off-white solid (column chromatography eluents: DCM/MeOH/$NH_3\cdot H_2O$ = 10:0.9:0.5). m.p. 163.6-163.9 ˚C. HRMS (ESI) $(M+H)^+$ *m/z* 995.5229, calcd for $C_{52}H_{75}N_4O_{15}$ 995.5223. ^1H NMR (CD_3OD, 400 MHz) δ: 8.86 (s, 1 H, 2″-quinolyl), 8.32 (d, *J* = 8.2 Hz, 1 H, 5″-quinolyl), 7.18 (s, 1 H, 8″-quinolyl), 7.50 (s, 1 H, 6″-quinolyl), 5.16 (dd, *J* = 2.5 Hz, 10.7 Hz, 1 H, H-13), 4.85 (d, *J* = 11.2 Hz, 1 H, H-3), 4.12 (d, *J* = 7.2 Hz, 1 H, H-1′), 3.84 (d, *J* = 3.1 Hz, 1 H, H-5), 3.78 (s, 1 H, H-11), 3.76-3.65 (m, 3 H, 1 H-cyclopropyl, -CH_2-O-C**H₂**-), 3.63-3.55 (m, 2 H, -C**H₂**-O-CH_2-), 3.46-3.38 (m, 1 H, H-5′), 3.37-3.32 (m, 1 H, 3-*O*-CO-NH-C**H₂**), 3.26 (dd, *J* = 7.3 Hz, 10.3 Hz, 1 H, H-2′), 3.21-3.13 (m, 1 H, 3-*O*-CO-NH-C**H₂**), 3.07 (q, *J* = 6.6 Hz, 1 H, H-10), 2.97 (s, 3 H, 6-*O*-CH_3), 2.86-2.70 (m, 4 H, H-2, H-3′, -CH_2-C≡C-), 2.55-2.47 (m, 1 H, H-8), 2.45 (s, 6 H, -N(CH_3)$_2$), 2.14-2.03 (m, 1 H, H-4), 1.90-1.72 (m, 5 H, H-14ax, H-7a, H-4′a, -CH_2-), 1.66-1.51 (m, 2 H, H-7b, H-14eq), 1.46 (s, 3 H, 12-CH_3), 1.42-1.34 (m, 2 H, 2 H-cyclopropyl), 1.31-1.15 (m, 9 H, H-4′b, 6-CH_3, 5′-CH_3, 2 H-cyclopropyl), 1.15-1.04 (m, 12 H, 2-CH_3, 4-CH_3, 10-CH_3, 8-CH_3), 0.83 (t, *J* = 7.3 Hz, 3 H, 15-CH_3). ^{13}C NMR (CD_3OD, 100 MHz) δ: 17.93, 174.78, 159.41, 157.30, 101.65, 84.48, 78.77, 77.88, 77.49, 75.51, 70.53, 68.67, 68.53, 68.18, 58.05, 48.94, 45.33, 43.22, 39.44, 38.68, 38.05, 37.41, 35.68, 30.75, 29.58, 21.80, 20.26, 19.96, 18.43, 17.16, 14.00, 12.91, 12.22, 9.41, 8.06, 7.19.

3-O-descladinosyl-3-O-{N-[4-(1,4-dihydro-1-ethyl-4-oxo-3-quinolinecarboxylic acid-7-yl)]-but-3-ynoxy-propylcarbamoyl} clarithromycin-11,12- cyclic carbamate ***(95f)***

Following the general procedure I-2, from compound **93** (0.250 g, 0.286 mmol) and **10** (0.128 g, 0.334 mmol), ethyl ester-**95f** was obtained. After reaction, ethyl ester-**95f** was treated with $LiOH\cdot H_2O$ to remove the protective group, **95f** (46.4 mg, 0.0472 mmol, 16.5%) was obtained as an off-white solid (column chromatography eluents: DCM/MeOH/$NH_3\cdot H_2O$ = 10:0.9:0.5). m.p. 170.9-171.1 ˚C. HRMS (ESI) $(M+H)^+$ *m/z* 983.5234, calcd for $C_{51}H_{75}N_4O_{15}$ 983.5223. ^1H NMR (CD_3OD, 400 MHz) δ: 8.87 (s, 1 H, 2″-quinolyl), 8.35 (d, *J* = 8.3 Hz, 1 H, 5″-quinolyl), 7.83 (s, 1 H, 8″-quinolyl), 7.44 (s, 1 H, 6″-quinolyl), 5.15 (dd, *J* = 2.5 Hz, 10.7 Hz, 1 H, H-13), 4.85 (d, *J*

= 11.4 Hz, 1 H, H-3), 4.45 (br, 2 H, NC**H**$_2$CH$_3$), 4.12 (d, *J* = 7.2 Hz, 1 H, H-1′), 3.83 (d, *J* = 3.1 Hz, 1 H, H-5), 3.75 (m, 1 H, H-11), 3.67 (t, *J* = 6.5 Hz, 2 H, -CH$_2$-O-C**H**$_2$-), 3.64-3.54 (m, 2 H, -C**H**$_2$-O-CH$_2$-), 3.46-3.38 (m, 1 H, H-5′), 3.38-3.33 (m, 2 H, 3-*O*-CO-NH-C**H**$_2$), 3.26 (dd, *J* = 7.3 Hz, 10.2 Hz, 1 H, H-2′), 3.22-3.10 (m, 1 H, 3-*O*-CO-NH-C**H**$_2$), 3.07 (q, *J* = 6.5 Hz, 1 H, H-10), 2.97 (s, 3 H, 6-*O*-CH$_3$), 2.87-2.71 (m, 4 H, H-2, H-3′, -CH$_2$-C≡C-), 2.52-2.45 (m, 1 H, H-8), 2.42 (s, 6 H, -N(CH$_3$)$_2$), 2.14-2.03 (m, 1 H, H-4), 1.91-1.71 (m, 5 H, H-14ax, H-7a, H-4′a, -CH$_2$-), 1.68-1.55 (m, 2 H, H-7b, H-14eq), 1.49 (t, *J* = 7.2 Hz, 3 H, NCH$_2$C**H**$_3$), 1.45 (s, 3 H, 12-CH$_3$), 1.32-1.26 (m, 1 H, H-4′b), 1.24 (s, 3 H, 6-CH$_3$), 1.16 (d, *J* = 6.2 Hz, 3 H, 5′-CH$_3$), 1.14-1.04 (m, 12 H, 2-CH$_3$, 4-CH$_3$, 10-CH$_3$, 8-CH$_3$), 0.83 (t, *J* = 7.3 Hz, 3 H, 15-CH$_3$). ^{13}C NMR (CD$_3$OD, 100 MHz) δ: 217.95, 174.77, 159.41, 157.30, 101.68, 84.48, 79.96, 78.77, 77.88, 77.50, 75.49, 70.64, 68.69, 68.54, 68.17, 64.60, 58.05, 48.94, 45.33, 43.22, 39.52, 38.68, 38.02, 37.41, 35.67, 30.84, 29.58, 21.79, 20.25, 19.99, 18.43, 17.18, 14.02, 13.63, 12.92, 12.23, 9.42, 8.07.

*3-O-descladinosyl-3-O-{N-[4-(1,4-dihydro-1-methyl-4-oxo-3-quinolinecarboxylic acid-7-yl)]-but-3-ynoxy-propylcarbamoyl} clarithromycin-11,12- cyclic carbamate **(95g)MCX-190***

Following the general procedure I-1, from compound **93** (0.200 g, 0.229 mmol) and **11** (0.0905 g, 0.275 mmol), **95g** (16.1 mg, 0.0166 mmol, 7.25%) was obtained as an off-white solid (column chromatography eluents: DCM/MeOH/NH$_3$·H$_2$O = 10:0.9:0.5). m.p. 157.9-158.8 ˚C. HRMS (ESI) (M+H)$^+$ *m/z* 969.5081, calcd for C$_{50}$H$_{73}$N$_4$O$_{15}$ 969.5067. HPLC purity: 98.0% (t= 13.3 min). ^{1}H NMR (CDCl$_3$, 400 MHz) δ: 8.76 (s, 1 H, 2″-quinolyl), 8.44 (d, *J* = 8.2 Hz, 1 H, 5″-quinolyl), 7.63 (s, 1 H, 8″-quinolyl), 7.56 (d, *J* = 8.2 Hz, 1 H, 6″-quinolyl), 5.80 (s, 1 H, 11-NH), 5.34 (s, 1 H, 3-*O*-CO-N**H**-CH$_2$), 5.22 (dd, *J* = 2.3 Hz, 10.9 Hz, 1 H, H-13), 4.89 (d, *J* = 11.1 Hz, 1 H, H-3), 4.10-3.96 (m, 4 H, H-1′, N-CH$_3$), 3.80-3.73 (m, 2 H, H-5, H-11), 3.71-3.56 (m, 4 H, -CH$_2$-O-CH$_2$-), 3.48-3.33 (m, 2 H, H-5′, 3-*O*-CO-NH-C**H**$_2$), 3.28-3.14 (m, 2 H, H-2′, 3-*O*-CO-NH-C**H**$_2$), 2.97 (s, 3 H, 6-*O*-CH$_3$), 2.89 (q, *J* = 6.4 Hz, 1 H, H-10), 2.81-2.71 (m, 3 H, H-2, -C**H**$_2$-C≡C-), 2.56-2.42 (m, 2 H, H-8, H-3′), 2.31 (s, 6 H, -N(CH$_3$)$_2$), 2.08-1.99 (m, 1 H, H-4), 1.95-1.88 (m, 1 H, H-14ax), 1.88-1.72 (m, 3 H, H-7a, -CH$_2$-), 1.67-1.50 (m, 3 H, H-7b, H-14eq, H-4′a), 1.42 (s, 3 H, 12-CH$_3$), 1.32-1.18 (m, 7 H, 6-CH$_3$, H-4′b, 5′-CH$_3$), 1.16-1.05 (m, 12 H, 2-CH$_3$, 10-CH$_3$, 4-CH$_3$, 8-CH$_3$), 0.83 (t, *J* = 7.4 Hz, 3 H, 15-CH$_3$). ^{13}C NMR (CDCl$_3$, 100 MHz) δ: 217.62, 178.07, 174.37, 166.78, 158.42, 156.44, 149.54, 140.16, 129.89, 129.35, 127.06, 125.28, 119.32, 109.12, 92.59, 83.89, 80.34, 77.87, 75.80, 70.51, 69.39, 69.09, 68.78, 58.14, 49.81, 45.30, 43.38, 40.26, 39.13, 37.29, 35.78, 29.90, 29.69, 22.03, 21.17, 20.98, 19.29, 18.28, 15.02, 13.86, 13.27, 10.24, 9.02.

*3-O-descladinosyl-3-O-{N-[4-(1,4-dihydro-4-oxo-3-quinolinecarboxylic acid-7-yl)]-but-3-ynoxy-propylcarbamoyl} clarithromycin-11,12- cyclic carbamate **(95i)***

Following the general procedure I-1, from compound **93** (0.200 g, 0.229 mmol) and **12** (0.0866 g, 0.275 mmol), **95i** (26.3 mg, 0.0275 mmol, 12.0%) was obtained as an off-white solid (column chromatography eluents: DCM/MeOH/NH$_3$·H$_2$O = 10:0.9:0.5). m.p. 163.7-164.9 ˚C. HRMS (ESI) (M+H)$^+$ *m/z* 955.4890, calcd for C$_{49}$H$_{71}$N$_4$O$_{15}$ 955.4910. ^{1}H NMR (CDCl$_3$, 400 MHz) δ: 8.84 (s, 1 H, 2″-quinolyl), 8.28 (d, *J* = 8.4 Hz, 1 H, 5″-quinolyl), 7.70 (s, 1 H, 8″-quinolyl), 7.44 (d, *J* = 8.4 Hz, 1 H, 6″-quinolyl), 5.85 (s, 1 H, 11-NH), 5.82 (s, 1 H, 3-*O*-CO-N**H**-CH$_2$), 5.20 (dd, *J* = 2.3 Hz, 10.8 Hz, 1 H, H-13), 4.89 (d, *J* = 11.0 Hz, 1 H, H-3), 4.11 (d, *J* = 7.1 Hz, 1 H, H-1′), 3.83 (d, *J* = 2.6 Hz, 1 H, H-5), 3.75 (s, 1 H, H-11), 3.71-3.59 (m, 4 H, -CH$_2$-O-CH$_2$-), 3.51-3.38 (m, 2 H, H-5′, 3-*O*-CO-NH-C**H**$_2$), 3.37-3.25 (m, 2 H, H-2′, 3-*O*-CO-NH-C**H**$_2$), 2.96 (s, 3 H, 6-*O*-CH$_3$), 2.87 (q, *J* = 6.5 Hz, 1 H, H-10), 2.80-2.66 (m, 4 H, H-2, -C**H**$_2$-C≡C-, H-3′), 2.54-2.46 (m, 2 H, H-8), 2.42 (s, 6 H, -N(CH$_3$)$_2$), 2.08-1.98 (m, 1 H, H-4), 1.94-1.80 (m, 3 H, H-14ax, -CH$_2$-), 1.79-1.67 (m, 2 H, H-7a, H-4′a), 1.57-1.46 (m, 2 H, H-7b, H-14eq), 1.39 (s, 3 H, 12-CH$_3$), 1.33-1.18 (m, 7 H, 6-CH$_3$, H-4′b, 5′-CH$_3$), 1.15-1.05 (m, 9 H, 2-CH$_3$, 10-CH$_3$, 8-CH$_3$), 0.99 (t, *J* = 7.3 Hz, 3 H, 4-CH$_3$), 0.82 (t, *J* = 7.3 Hz, 3 H, 15-CH$_3$). ^{13}C NMR (CDCl$_3$, 100 MHz) δ: 217.59, 177.50, 174.43, 168.97, 158.54, 156.56, 145.85, 128.90, 125.60, 123.90, 123.04, 108.71, 102.51, 92.14, 84.04, 81.04, 80.24, 78.15, 77.83, 77.24, 75.78, 70.71, 69.40, 69.17, 68.70, 65.65, 58.19, 49.77, 45.27, 43.34, 40.06, 39.57, 39.07, 37.28, 35.71, 29.51, 28.92, 22.02, 21.09, 19.28, 18.21, 15.04, 13.84, 13.26, 10.22, 9.08.

*3-O-descladinosyl-3-O-{N-[4-(3-quinolinecarboxylic acid-7-yl)-1-yl]-but-3-ynoxy-propylcarbamoyl} clarithromycin-11,12- cyclic carbamate **(95j)***

Following the general procedure I-2, from compound **93** (0.300 g, 0.344 mmol) and methyl 7-bromoquinoline-3-carboxylate (0.110 g, 0.413 mmol), methyl ester-**95j** was obtained. After completion of this reaction, the protective group of methyl ester was removed in a solution of LiOH·H_2O in THF/H_2O (5mL/5mL), and **95j** (49.3 mg, 0.0524 mmol, 15.3%) was obtained as an off-white solid (column chromatography eluents: DCM/MeOH/NH_3·H_2O = 10:1:0.5). m.p. 163.6-164.7 ˚C. HRMS (ESI) $(M+H)^+$ *m/z* 939.4967, calcd for $C_{49}H_{71}N_4O_{14}$ 939.4961. ^1H NMR ($CDCl_3$, 400 MHz) δ: 9.53 (d, *J* = 2.0 Hz, 1 H, 2″-quinolyl), 8.75 (d, *J* = 2.0 Hz, 1 H, 4″-quinolyl), 8.06 (s, 1 H, 8″-quinolyl), 7.76 (d, *J* = 8.4 Hz, 1 H, 5″-quinolyl), 7.35 (dd, *J* = 1.6 Hz, 8.4 Hz, 1 H, 6″-quinolyl), 6.90 (d, *J* = 5.7 Hz, 1 H, 3-*O*-CO-N**H**-CH_2), 5.80 (s, 1 H, 11-NH), 5.24 (dd, *J* = 2.4 Hz, 10.8 Hz, 1 H, H-13), 4.96 (d, *J* = 11.0 Hz, 1 H, H-3), 4.18 (d, *J* = 7.0 Hz, 1 H, H-1′), 3.79 (m, 1 H, H-11), 3.70 (d, *J* = 2.1 Hz, 1 H, H-5), 3.54-3.44 (m, 3 H, -C**H₂**-O-, H-5′), 3.43-3.32 (m, 3 H, 3-*O*-CO-NH-C**H**$_2$, -O-C**H**$_2$-, H-2′), 3.32-3.23 (m, 1 H, -O-C**H**$_2$-), 3.48-3.33 (m, 1 H, 3-*O*-CO-NH-C**H₂**), 3.15-3.05 (m, 1 H, 3-*O*-CO-NH-C**H₂**), 2.98 (s, 3 H, 6-*O*-CH_3), 2.94-2.83 (m, 2 H, H-10, H-2), 2.78 (s, 6 H, -N$(CH_3)_2$), 2.75-2.68 (m, 1 H, H-3′), 2.56-2.40 (m, 2 H, H-8, -C**H₂**-C≡C-), 2.29-2.18 (m, 1 H, -C**H**$_2$-C≡C-), 2.14-2.05 (m, 1 H, H-4), 1.97-1.78 (m, 4 H, H-14ax, -C**H**$_2$-, H-7a, H-4′a), 1.77-1.66 (m, 1 H, -C**H**$_2$-), 1.62-1.50 (m, 2 H, H-7b, H-14eq,), 1.44 (s, 3 H, 12-CH_3), 1.42-1.24 (m, 7 H, 6-CH_3, H-4′b, 5′-CH_3), 1.24-1.18 (m, 6 H, 2-CH_3, 4-CH_3), 1.15 (d, *J* = 6.5 Hz, 3 H, 10-CH_3), 1.09 (d, *J* = 7.1 Hz, 3 H, 8-CH_3), 0.86 (t, *J* = 7.4 Hz, 3 H, 15-CH_3). ^{13}C NMR ($CDCl_3$, 100 MHz) δ: 217.58, 174.52, 172.30, 158.48, 156.88, 152.13, 148.73, 137.37, 132.06, 129.82, 128.63, 128.15, 126.63, 126.04, 103.53, 89.09, 84.61, 84.01, 81.16, 78.42, 77.76, 77.22, 75.72, 70.64, 68.86, 68.37, 64.65, 58.29, 49.66, 45.36, 43.57, 39.16, 38.86, 38.34, 37.35, 35.78, 30.26, 29.74, 22.11, 21.10, 20.61, 19.22, 18.23, 15.05, 13.90, 13.33, 10.25, 9.34.

3-O-descladinosyl-3-O-{N-[4-(1,4-dihydro-1-methyl-4-oxo-3-quinolinecarboxylic acid-7-yl)-1-yl]-but-3-ynoxy-butylcarbamoy} clarithromycin-11,12- cyclic carbamate ***(96g)***

Following the general procedure I-1, from compound **94** (0.421 g, 0.502 mmol) and **11** (0.214 g, 0.602 mmol), **96g** (51.2 mg, 0.0521 mmol, 10.4%) was obtained as an off-white solid (column chromatography eluents: DCM/MeOH/NH_3·H_2O = 10:0.9:0.5). m.p. 172.1-173.5 ˚C. HRMS (ESI) $(M+H)^+$ *m/z* 983.5243, calcd for $C_{51}H_{75}N_4O_{15}$ 983.5223. ^1H NMR (CD_3OD, 400 MHz) δ: 8.82 (s, 1 H, 2″-quinolyl), 8.32 (d, *J* = 8.2 Hz, 1 H, 5″-quinolyl), 7.72 (s, 1 H, 8″-quinolyl), 7.40 (s, 1 H, 6″-quinolyl), 5.15 (dd, *J* = 2.4 Hz, 10.7 Hz, 1 H, H-13), 4.85 (d, *J* = 11.4 Hz, 1 H, H-3), 4.13 (d, *J* = 7.3 Hz, 1 H, H-1′), 3.99 (s, 3 H, N-CH_3), 3.83 (d, *J* = 3.1 Hz, 1 H, H-5), 3.78 (m, 1 H, H-11), 3.66 (t, *J* = 6.5 Hz, 2 H, -CH_2-O-C**H₂**-), 3.58-3.51 (m, 2 H, -C**H₂**-O-CH_2-), 3.46-3.37 (m, 1 H, H-5′), 3.29-3.22 (m, 2 H, H-2′, 3-*O*-CO-NH-C**H₂**), 3.10-3.01 (m, 2 H, H-10, 3-*O*-CO-NH-C**H₂**), 2.97 (s, 3 H, 6-*O*-CH_3), 2.89-2.78 (m, 2 H, H-2, H-3′), 2.74 (t, *J* = 6.4 Hz, 2 H, -CH_2-C≡C-), 2.51-2.45 (m, 1 H, H-8), 2.43 (s, 6 H, -N$(CH_3)_2$), 2.13-2.05 (m, 1 H, H-4), 1.90-1.74 (m, 3 H, H-14ax, H-7a, H-4′a), 1.70-1.52 (m, 6 H, 2(-CH_2-), H-7b, H-14eq), 1.45 (s, 3 H, 12-CH_3), 1.32-1.26 (m, 1 H, H-4′b), 1.24 (s, 3 H, 6-CH_3), 1.17 (d, *J* = 6.1 Hz, 3 H, 5′-CH_3), 1.15-1.05 (m, 12 H, 2-CH_3, 4-CH_3, 10-CH_3, 8-CH_3), 0.82 (t, *J* = 7.4 Hz, 3 H, 15-CH_3). ^{13}C NMR (CD_3OD, 100 MHz) δ: 217.96, 174.78, 159.40, 157.34, 101.69, 84.49, 79.92, 78.83, 77.88, 77.46, 75.48, 70.66, 70.20, 68.69, 68.44, 64.58, 58.06, 48.93, 45.34, 43.23, 40.95, 40.37, 39.54, 38.68, 37.41, 35.66, 30.87, 26.68, 26.37, 21.79, 20.25, 20.00, 18.43, 17.19, 14.00, 12.93, 12.23, 9.43, 8.09.

2′-O-benzoyl-3-O-descladinosyl-3-O-(N-hydroxypropylcarbamoyl) clarithromycin-11,12- cyclic carbamate ***(97)***

Following the general procedure H, from compound **83** (0.500 g, 0.615 mmol) and 1,3-diaminopropane (0.0940 mL, 1.23 mmol), **97** (0.282 g, 0.371 mmol, 60.3%) was obtained as an off-white solid (column chromatography eluents: DCM/EtOH/NH_3·H_2O = 10:0.7:0.1). HRMS (ESI) $(M+H)^+$ *m/z* 820.4589, calcd for $C_{42}H_{66}N_3O_{13}$ 820.4590. ^1H NMR ($CDCl_3$, 400 MHz) δ: 8.09-8.02 (m, 2 H, 2 H-Bz), 7.61-7.55 (m, 1 H, 1 H-Bz), 7.49-7.43 (m, 2 H, 2 H-Bz), 5.80 (s, 1 H, 11-NH), 5.35 (t, *J* = 6.0 Hz, 1 H, 3-*O*-CO-N**H**-CH_2), 5.18 (dd, *J* = 2.3 Hz, 11.0 Hz, 1 H, H-13), 4.99 (dd, *J* = 7.5 Hz, 10.5 Hz, 1 H, H-2′), 4.88 (d, *J* = 11.0 Hz, 1 H, H-3), 4.30 (d, *J* = 7.5 Hz, 1 H, H-1′), 3.80 (d, *J* = 3.1 Hz, 1 H, H-5), 3.78-3.72 (m, 2 H, -C**H₂**-OH), 3.70 (s, 1 H, H-11), 3.60-3.50 (m, 1 H, 3-*O*-CO-NH-C**H₂**), 3.47-3.38 (m, 1 H, H-5′), 3.33-3.24 (m, 1 H, 3-*O*-CO-NH-C**H₂**), 2.96 (s, 3 H, 6-*O*-CH_3), 2.90-2.70 (m, 3 H, H-3′, H-10, H-2), 2.52-2.43 (m, 1 H, H-8), 2.28 (s, 6 H, -N$(CH_3)_2$), 1.99-1.69 (m, 5 H, H-4, H-14eq, H-4′a,-CH_2-), 1.58-1.36 (m, 4 H, H-7a,

H-7b, H-14eq, H-4′a), 1.31 (s, 3 H, 12-CH_3), 1.29-1.23 (m, 6 H, 6-CH_3, 5′-CH_3), 1.15-1.03 (m, 9 H, 2-CH_3, 10-CH_3, 8-CH_3), 0.81 (t, J = 7.4 Hz, 3 H, 15-CH_3), 0.73 (d, J = 7.5 Hz, 3 H, 4-CH_3).

3-O-descladinosyl-3-O-[N-4-(1-cyclopropyl-1,4-dihydro-4-oxo-3-quinolinecarboxylic acid-7-yl)-but-3-ynamino-propylcarbamoyl] clarithromycin-11,12- cyclic carbamate ***(98c)***

To a solution of **97** (0.490 g, 0.645 mmol) in CH_2Cl_2, methanesulfonyl chloride (0.058 mL, 0.710 mmol) and Et_3N (0.13 mL, 0.968 mmol) were added. The mixture was stirred at room temperature for 1 h. Then the CH_2Cl_2 layer was washed with brine, and evaporated under vacuum. The organic solvent was removed in vacuo and purified by column chromatography (100-200 mesh silicone, CH_2Cl_2/EtOH/$NH_3.H_2O$=10/0.1/0.1) to an intermediate (0.277 g, 0.308 mmol, 47.8%).

To a solution of the intermediate (0.277 g, 0.308 mmol) in CH_3CN, **34** (0.137 g, 0.463 mmol) was added. The mixture was stirred at 75˚C for 48 h followed by the addition of CH_2Cl_2 (50 mL) and water (50 mL). The CH_2Cl_2 layer was washed with brine, and evaporated under vacuum. The residue was then dissolved in MeOH (15 mL) at 65 ˚C for 12 h. The organic solvent was removed in vacuo and purified by column chromatography (100-200 mesh silicone, CH_2Cl_2/MeOH/$NH_3.H_2O$ = 10/0.9/0.5) to yield **98c** (9.6 mg, 0.00966 mmol, 3.14%). M.p. 148.6-149.5 ˚C. HRMS (ESI) $(M+H)^+$ *m/z* 994.5401, calcd for $C_{52}H_{76}N_5O_{14}$ 994.5383. ^1H NMR ($CDCl_3$, 400 MHz) δ: 8.85 (s, 1 H, 2″-quinolyl), 8.41 (d, J = 8.3 Hz, 1 H, 5″-quinolyl), 8.07 (s, 1 H, 8″-quinolyl), 7.55 (d, J = 8.3 Hz, 1 H, 6″-quinolyl), 5.80 (s, 1 H, 11-NH), 5.52 (s, 1 H, 3-*O*-CO-N**H**-CH_2), 5.22 (dd, J = 2.3 Hz, 10.9 Hz, 1 H, H-13), 4.90 (d, J = 11.1 Hz, 1 H, H-3), 4.04 (d, J = 7.0 Hz, 1 H, H-1′), 3.83-3.74 (m, 1 H, H-5, H-11), 3.63-3.54 (m, 1 H, 1 H-cyclopropyl), 3.50-3.31 (m, 2 H, H-5′, 3-*O*-CO-NH-C**H**$_2$), 3.23-3.12 (m, 2 H, H-2′, 3-*O*-CO-NH-C**H**$_2$), 2.97 (s, 3 H, 6-*O*-CH_3), 2.96-2.84 (m, 4 H, H-10, H-2, -CH_2-NH-), 2.85-2.76 (m, 2 H, -NH-CH_2-), 2.75-2.63 (m, 2 H, -C**H**$_2$-C≡C-), 2.56-2.39 (m, 2 H, H-8, H-3′), 2.28 (s, 6 H, -$N(CH_3)_2$), 2.09-2.02 (m, 1 H, H-4), 1.97-1.85 (m, 1 H, H-14ax), 1.82-1.70 (m, 3 H, H-7a, -CH_2-), 1.67-1.49 (m, 3 H, H-7b, H-14eq, H-4′a), 1.47-1.38 (s, 5 H, 12-CH_3, 2 H-cyclopropyl), 1.31-1.17 (m, 9 H, 6-CH_3, H-4′b, 5′-CH_3, 2 H-cyclopropyl), 1.20-1.06 (m, 12 H, 2-CH_3, 10-CH_3, 4-CH_3, 8-CH_3), 0.87 (t, J = 7.4 Hz, 3 H, 15-CH_3). ^{13}C NMR ($CDCl_3$, 175 MHz) δ: 217.68, 174.40, 158.45, 156.57, 148.54, 141.00, 129.49, 129.36, 126.94, 125.07, 120.00, 103.13, 83.90, 81.60, 80.84, 77.86, 75.79, 70.53, 69.45, 58.13, 49.82, 45.31, 43.39, 40.35, 39.13, 37.29, 35.79, 35.31, 29.70, 29.37, 22.70, 22.03, 21.19, 19.31, 18.29, 15.06, 14.13, 13.89, 13.29, 11.10, 10.26, 9.28, 9.06, 8.36.

Synthesis of compounds 107-111

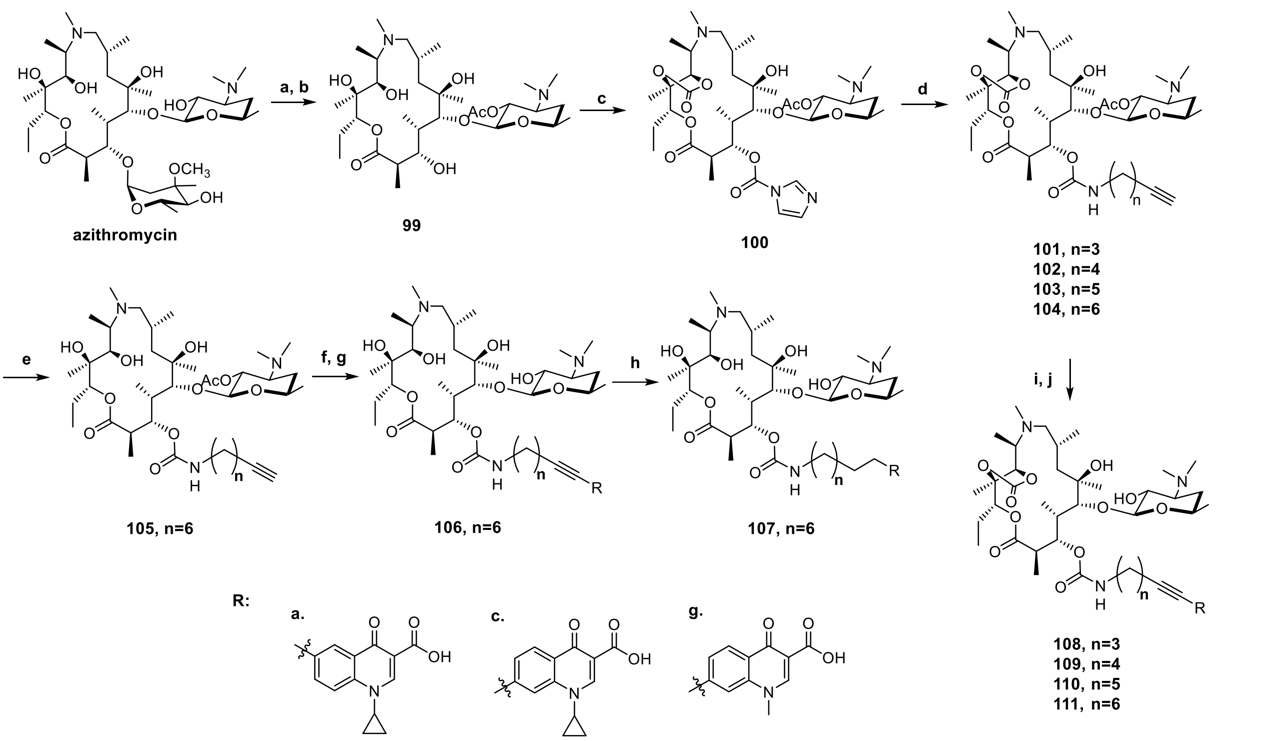

Scheme S12. Synthesis of *azithromycin* series compounds **107-111**. Reagents and conditions: (a) 10% HCl, CH_3CH_2OH, 40 ˚C; (b) 1) acetic anhydride, DCM, rt, 1-1.5 h; 2) sat. $NaHCO_3$; (c) CDI, DMAP, DCM, rt, 12 h; (d) **26-29**, DBU, DMF, rt, 12 h; (e) $LiOH{\cdot}H_2O$, THF/ H_2O, rt, 2 h; (f) R-I (R = **c**), CuI, $Pd(PPh_3)_2Cl_2$, Et_3N, CH_3CN, 45 ˚C, 12 h; (g) CH_3OH, 65 ˚C, 2 h; (h) HCOOH, $HCOONH_4$, 10% Pd/C, H_2, CH_3OH, rt, 16 h; (i) R-I (R = **a**, **c**), CuI, $Pd(PPh_3)_2Cl_2$, Et_3N, CH_3CN, 45 ˚C, 2 h; (j) CH_3OH, 65 ˚C, 2 h.

2'-O-Acetyl-3-O-descladinosyl-3-hydroxy-azithromycin ***(99)***

1M HCl (30 mL) was added to a solution of azithromycin (20.0 g, 26.7 mmol) in EtOH (35 mL) at 40 ˚C. The mixture was stirred at 40 ˚C for 1 h. CH_2Cl_2 (100 mL) were added and the pH was adjusted to 10 with ammonia water. The CH_2Cl_2 layer was washed with brine, and evaporated under vacuum to yield 3-OH-azithromycin.

To a solution of 3-OH-azithromycin (16.1 g, 27.2 mmol) in dry CH_2Cl_2 (150 mL), acetic anhydride (5.15 mL, 54.5 mmol) was added. The mixture was stirred at room temperature for 1 h. The organic layer was washed with saturated $NaHCO_3$ (5×50 mL), water and brine, and evaporated under reduced pressure to yield **99** (16.4 g, 25.9 mmol, 95.1%).

2'-O-Acetyl-3-O-descladinosyl-3-O-(1H-imidazol-1-ylcarbonyl) azithromycin 11,12- cyclic carbonate ***(100)***

The compound **99** (14.9 g, 23.5 mmol), CDI (11.4 g, 70.5 mmol) and DMAP (5.74 g, 47.0 mmol) were dissolved in dry CH_2Cl_2. The mixture was stirred at 25˚C for 24 h followed by the addition of CH_2Cl_2 (50 mL) and water (50 mL). The organic layer was washed with water and brine, and concentrated in vacuo to crude product. The crude product was redissolved in ethyl acetate precipitated as a white solid **100** (14.2 g, 18.9 mmol, 80.6%) that was filtered off. HRMS (ESI) $(M+H)^+$ *m/z* 753.4280, calcd for $C_{37}H_{61}N_4O_{12}$ 753.4278. ^1H NMR ($CDCl_3$, 400 MHz) δ: 8.22 (s, 1 H, H-imidazole), 7.51 (t, *J* = 1.5 Hz, 1 H, H-imidazole), 7.14 (dd, *J* = 1.7, 0.9 Hz, 1 H, H-imidazole), 6.62 (s, 1 H, 6-OH), 5.67 (dd, *J* = 9.2, 3.3 Hz, 1 H, H-3), 5.07 (dd, *J* = 10.8, 2.4 Hz, 1 H, H-13), 4.69 (dd, *J* = 10.6, 7.5 Hz, 1 H, H-2′), 4.59 (s, 1 H, H-11), 4.02 (d, *J* = 7.5 Hz, 1 H, H-1′), 3.60 (d, *J* = 2.7 Hz, 1 H, H-5), 3.21-3.12 (m, 1 H, H-2), 2.86 (q, *J* = 6.6 Hz, 1 H, H-10), 2.69-2.60 (m, 1 H, H-5′), 2.40-2.34 (m, 1 H, H-9a), 2.34-2.29 (m, 1 H, H-3′), 2.23-2.30 (m, 4 H, -NCH_3, H-4), 2.20 (s, 6 H, -$N(CH_3)_2$), 2.09 (s, 3 H, 2′-O-CO-CH_3), 2.07-2.03 (m, 1 H, H-9b), 1.94-1.82 (m, 2 H, H-7a, H-14eq), 1.79-1.67 (m, 1 H, H-8), 1.64-1.56 (m, 1 H, H-14ax), 1.55-1.48 (m, 2 H, H-7b, H-4′a), 1.43 (s, 3 H, 12-CH_3), 1.31-1.26 (m, 1 H, H-4′b), 1.25 (d, *J* = 7.1 Hz, 3 H, 5′-CH_3), 1.22 (s, 3 H, 6-CH_3) , 1.11 (d, *J* = 6.1 Hz,

3 H, 10-CH_3), 1.09-1.04 (m, 6 H, 2-CH_3, 4-CH_3), 0.94-0.88 (m, 6 H, 8-CH_3, 15-CH_3).

*2'-O-Acetyl-3-O-descladinosyl-3-O-(N-4-pentynylcarbamoyl) azithromycin 11,12- cyclic carbonate **(101)***

Following the general procedure H, from compound **100** (0.800 g, 1.06 mmol) and **26** (0.150 g, 2.54 mmol), **101** (0.164 g, 0.214 mmol, 20.2%) was obtained as an off-white solid (column chromatography eluents: DCM/EtOH/NH_3·H_2O = 10:0.4:0.05).

*2'-O-Acetyl-3-O-descladinosyl-3-O-(N-5-hexynylcarbamoyl) azithromycin 11,12- cyclic carbonate **(102)***

Following the general procedure H, from compound **100** (1.00 g, 1.33 mmol) and **27** (0.270 g, 2.00 mmol), **102** (0.250 g, 0.320 mmol, 24.1%) was obtained as an off-white solid (column chromatography eluents: DCM/EtOH/NH_3·H_2O = 10:0.3:0.05).

*2'-O-Acetyl-3-O-descladinosyl-3-O-(N-6-heptynylcarbamoyl) azithromycin 11,12- cyclic carbonate **(103)***

Following the general procedure H, from compound **100** (1.50 g, 1.99 mmol) and **28** (0.440 g, 2.98 mmol), **103** (0.326 g, 0.410 mmol, 20.6%) was obtained as an off-white solid (column chromatography eluents: DCM/EtOH/NH_3·H_2O = 10:0.3:0.05).

*2'-O-Acetyl-3-O-descladinosyl-3-O-(N-7-octynylcarbamoyl) azithromycin 11,12- cyclic carbonate **(104)***

Following the general procedure H, from compound **100** (1.05 g, 1.06 mmol) and **29** (0.480 g, 6.96 mmol), **104** (0.243 g, 0.300 mmol, 15.1%) was obtained as an off-white solid (column chromatography eluents: DCM/EtOH/NH_3·H_2O = 10:0.4:0.05).

General procedure N

Compound **104** (1eq) was dissolved in THF/H_2O (10 mL/10 mL) at room temperature. LiOH·H_2O (4 eq) was added, and the reaction mixture was stirred at room temperature for 2 h. The reaction mixture was concentrated and the crude residue was purified by column chromatography to yield **105**.

*2'-O-Acetyl-3-O-descladinosyl-3-O-(N-7-octynylcarbamoyl) azithromycin **(105)***

Following the general procedure N, from compound **104** (1.84 g, 2.27 mmol), **105** (0.243 g, 0.300 mmol, 15.1%) was obtained as an off-white solid (column chromatography eluents: DCM/EtOH/NH_3·H_2O = 10:0.1:0.05).

*3-O-descladinosyl-3-O-[N-8-(1,4-dihydro-1-methyl-4-oxo-3-quinolinecarboxylic acid-7-yl)-otc-7-ynylcarbamoyl] azithromycin **(106g)***

Following the general procedure I-1, from compound **105** (0.257 g, 0.33 mmol) and **11** (0.129 g, 0.390 mmol), **106g** (0.150 g, 0.160 mmol, 48.5%) was obtained as an off-white solid (column chromatography eluents: DCM/MeOH/NH_3·H_2O = 10:1.2:0.5). m.p. 147.5-149.2 ˚C. HRMS (ESI) $(M + H)^+$ *m/z* 943.5645, calcd for $C_{50}H_{79}N_4O_{13}$ 943.5638. HPLC purity: 96.3% (t= 12.6 min). ^1H NMR ($CDCl_3$, 400 MHz) δ: 8.76 (s, 1 H, H-quinolyl), 8.44 (d, *J* = 10.0 Hz, 1 H, H-quinolyl), 7.68 (s, 1 H, H-quinolyl), 7.55 (d, *J* = 10.0 Hz, 1 H, H-quinolyl), 5.08 (d, *J* = 10.8 Hz, 1 H, H-3), 5.03 (s, 1 H, -CONH-), 4.66 (dd, *J* = 10.4, 2.0 Hz, 1 H, H-13), 4.10-4.06 (m, 3 H, N-CH_3) 3.63-3.52 (m, 3 H, H-11, H-1′, H-5), 3.45-3.41 (m, 2 H, H-5′, -CONH-C**H**$_2$-), 3.28-3.23 (m, 1 H, H-2′), 3.08-3.04 (m, 1 H, -CONH-C**H**$_2$-), 2.84-2.64 (m, 3 H, H-3′, H-2, H-10), 2.50-2.47 (m, 3 H, -C**H$_2$**C≡C-Ar, H-9a), 2.37 (s, 3 H, -N-CH_3), 2.29 (s, 6 H, -N(CH_3)$_2$), 2.05-2.00 (m, 2 H, H-4, H-9b), 1.92-1.87 (m, 3 H, H-8, H-14eq, H-7a), 1.66-1.48 (m, 8 H, 3(CH_2), H-14ax, H-4′a), 1.44-1.39 (m, 2 H, -C**H$_2$**CH_2CH_2C≡C-Ar), 1.30-1.26 (m, 2 H, H-7b, H-4′b), 1.22 (s, 3 H, 12-CH_3), 1.18 (d, *J* = 6.0 Hz, 3 H, 5′-CH_3), 1.15-1.11 (m, 6 H, 2-CH_3, 6-CH_3), 1.06-1.05 (m, 6 H, 4-CH_3, 10-CH_3), 0.90-0.84 (m, 6 H, 8- CH_3, 15-CH_3). ^{13}C NMR ($CDCl_3$, 175 MHz) δ: 178.1, 176.4, 156.4, 149.6, 129.3, 126.9, 119.4, 103.6, 95.8, 87.9, 79.9, 79.5, 77.8, 76.0, 74.2, 73.0, 71.1, 70.9, 69.3, 65.9, 62.4, 43.4, 43.3, 42.2, 42.0, 41.2, 40.7, 40.4, 36.8, 35.7, 30.1, 30.0, 28.9, 28.2, 28.1, 26.3, 26.2, 21.4, 21.2, 20.8, 19.4, 16.0, 15.8, 15.7, 9.0, 7.6.

*3-O-descladinosyl-3-O-[N-8-(1,4-dihydro-1-methyl-4-oxo-3-quinolinecarboxylic acid-7-yl)-otcylcarbamoyl] azithromycin **(107g)***

Following the general procedure E, from compound **106g** (0.150 g, 0.16 mmol), **107g** (68.0 mg, 0.0700 mmol, 44.8%) was obtained as an off-white solid (column chromatography eluents: DCM/MeOH/NH_3·H_2O = 10:1.2:0.5). m.p. 142.8-144.1 ˚C. HRMS (ESI) $(M + H)^+$ *m/z* 947.5951, calcd for $C_{50}H_{83}N_4O_{13}$ 947.5951. ^1H NMR (CD_3OD, 400 MHz) δ: 8.75 (s, 1 H, H-quinolyl), 8.27 (d, *J* = 10.0 Hz, 1 H, H-quinolyl), 7.53 (s, 1 H, H-quinolyl), 7.31 (s, 1 H, H-

quinolyl), 5.04 (d, *J* = 10.8 Hz, 1 H, H-3), 4.93 (dd, *J* = 10.8, 2.0 Hz, 1 H, H-13), 4.24 (d, *J* = 7.2 Hz, 1 H, H-1′), 4.00 (s, 3 H, N-CH_3), 3.65 (d, *J* = 2.0 Hz, 1 H, H-11), 3.59 (m, 1 H, H-5), 3.49-3.41 (m, 1 H, H-5′), 3.28-3.25 (m, 2 H, -CONH-C**H**$_2$-, H-2′), 3.00-2.92 (m, 1 H, -CONH-C**H**$_2$-), 2.87-2.79 (m, 4 H, CH_2, H-3′, H-2), 2.52-2.50 (m, 1 H, H-9a), 2.41 (s, 6 H, -N(CH_3)$_2$), 2.37 (s, 4 H, H-10, -N-CH_3), 2.31-2.29 (m, 1 H, H-4), 2.25-2.15 (m, 1 H, H-9b), 1.90-1.85 (m, 2 H, H-14eq, H-7a), 1.75-1.67 (m, 3 H, H-8, 2(CH_2)), 1.52-1.48 (m, 4 H, CH_2, H-14ax, H-4′a), 1.35-1.26 (m, 10 H, 4(CH_2), H-7b, H-4′b), 1.20 (s, 3 H, 12-CH_3), 1.16-1.11 (m, 9 H, 5′-CH_3, 2-CH_3, 6-CH_3), 1.06-1.04 (m, 6 H, 4-CH_3, 10-CH_3), 0.92 (d, *J* = 6.8 Hz, 3 H, 8-CH_3), 0.86 (t, *J* = 7.2 Hz, 3 H, 15-CH_3). ^{13}C NMR (CD_3OD-d_4, 100 MHz) δ: 177.6, 175.7, 157.4, 140.4, 126.4, 115.7, 101.2, 83.3, 78.7, 77.4, 75.8, 74.1, 73.4, 71.0, 69.7, 68.4, 64.5, 62.4, 43.1, 40.9, 40.5, 39.6, 35.9, 35.8, 31.0, 30.9, 29.6, 29.1, 28.9, 28.8, 26.5, 26.1, 25.1, 20.6, 20.3, 20.0, 16.2, 14.7, 9.8, 8.2, 5.9.

3-O-descladinosyl-3-O-[N-5-(1-cyclopropyl-1,4-dihydro-4-oxo-3-quinolinecarboxylic acid-7-yl)-pent-4-ynylcarbamoyl] azithromycin 11,12- cyclic carbonate ***(108c)***

Following the general procedure I-1, from compound **101** (0.159 g, 0.210 mmol) and **4** (0.0890 g, 0.250 mmol), **108c** (27.7 mg, 0.0291 mmol, 13.8%) was obtained as an off-white solid (column chromatography eluents: DCM/MeOH/$NH_3 \cdot H_2O$ = 10:1:0.5). m.p. 149-151 °C. HRMS (ESI) $(M+H)^+$ *m/z* 953.5092, calcd for $C_{50}H_{73}N_4O_{14}$ 953.5118. ^{1}H NMR ($CDCl_3$, 400 MHz) δ: 8.87 (s, 1 H, H-quinolyl), 8.39 (d, *J* = 8.3 Hz, 1 H, H-quinolyl), 8.22 (s, 1 H, H-quinolyl), 7.82 (d, *J* = 8.0 Hz, 1 H, H-quinolyl), 6.18-5.94 (m, 2 H, -CONH-, 6-OH), 5.14 (d, *J* =10.3 Hz, 1 H, H-3), 5.05 (dd, *J* = 10.8, 2.4 Hz, 1 H, H-13), 4.58 (s, 1 H, H-11), 4.20-4.13 (m, 1 H, H-1′), 3.72-3.63 (m, 1 H, H-5′), 3.63-3.54 (m, 1 H, -CONH-C**H**$_2$-), 3.50 (d, *J* =1.8 Hz, 1 H, H-5), 3.48-3.40 (m, 1 H, 1 H-cyclopropyl), 3.34-3.17 (m, 2 H, H-2′, -CONH-C**H**$_2$-), 2.87-2.71 (m, 3 H, H-10, H-3′, H-2), 2.61-2.53 (m, 2 H, -C**H$_2$**C≡C-Ar), 2.48 (s, 6 H, -N(CH_3)$_2$), 2.39-2.33 (m, 1 H, H-9a), 2.26 (s, 3 H, -N-CH3), 2.18-2.10 (m, 1 H, H-4), 2.06-1.98 (m, 1 H, H-9b), 1.95-1.78 (m, 5 H, H-8, -CO-NH-CH_2C**H$_2$**-, H-14eq, H-7a), 1.64-1.53 (m, 3 H, H-14ax, 2 H-cyclopropyl), 1.52-1.47 (m, 1 H, H-4′a), 1.40 (s, 3 H, 12-CH_3), 1.35-1.23 (m, 4 H, H-7b, 2 H-cyclopropyl, H-4′b), 1.23-1.14 (m, 9 H, 6-CH_3, 5′-CH_3, 2-CH_3), 1.09-1.01 (m, 6 H, 10-CH_3, 4-CH_3), 0.94-0.85 (m, 6 H, 8-CH_3, 15-CH_3). ^{13}C NMR (CDCl3, 100 MHz) δ: 178.2, 174.4, 166.8, 156.6, 153.4, 148.5, 141.2, 129.8, 129.1, 126.8, 124.9, 120.5, 108.9, 103.4, 94.5, 88.6, 86.4, 84.8, 80.6, 78.8, 77.2, 75.8, 72.9, 70.7, 68.8, 68.0, 65.3, 61.9, 43.3, 42.0, 40.0, 39.6, 35.9, 35.6, 34.9, 29.5, 28.6, 26.0, 25.7, 21.7, 21.4, 21.1, 17.1, 15.7, 13.4, 10.1, 9.5, 8.4, 4.9.

3-O-descladinosyl-3-O-[N-6-(1-cyclopropyl-1,4-dihydro-4-oxo-3-quinolinecarboxylic acid-6-yl)-hex-5-ynylcarbamoyl] azithromycin 11,12- cyclic carbonate ***(109a)***

Following the general procedure I-1, from compound **102** (0.250 g, 0.320 mmol) and 1-cyclopropyl-1,4-dihydro-4-oxo-6-iodo-3-quinolinecarboxylic acid (0.130 g, 0.380 mmol), **109a** (55.3 mg, 0.0572 mmol, 17.9%) was obtained as an off-white solid (column chromatography eluents: DCM/MeOH/$NH_3 \cdot H_2O$ = 10:1:0.5). m.p. 131-133 °C. HRMS (ESI) $(M+H)^+$ *m/z* 967.5288, calcd for $C_{51}H_{75}N_4O_{14}$ 967.5274. ^{1}H NMR ($CDCl_3$, 400 MHz) δ: 8.85 (s, 1 H, H-quinolyl), 8.48 (d, *J* = 2.1 Hz, 1 H, H-quinolyl), 8.02 (d, *J* = 8.8 Hz, 1 H, H-quinolyl), 7.82 (dd, *J* = 8.9, 2.1 Hz, 1 H, H-quinolyl), 4.91 (dd, *J* = 9.0, 3.4 Hz, 1 H, H-13), 4.78 (t, *J* = 6.0 Hz, 1 H, -CONH-), 4.53 (d, *J* = 7.2 Hz, 1 H, H-1′), 4.49 (s, 1 H, H-11), 4.45 (d, *J* = 4.4 Hz, 1 H, H-3), 3.67-3.51 (m, 3 H, H-5, 1 H-cyclopropyl, H-5′), 3.33 (dd, *J* = 10.1, 7.3 Hz, H-2′), 3.30-3.21 (m, 1 H, -CONH-C**H**$_2$-), 3.18-3.09 (m, 1 H, -CONH-C**H**$_2$-), 2.87-2.79 (m, 1 H, H-3′), 2.75-2.62 (m, 2 H, H-10, H-2), 2.50 (t, *J* = 4.3 Hz, 2 H, -C**H$_2$**C≡C-Ar), 2.40 (s, 6 H, -N(CH_3)$_2$), 2.36-2.31 (m, 1 H, H-9a), 2.18 (s, 3 H, -N-CH_3), 1.99-1.79 (m, 5 H, H-4, H-9b, H-14eq, H-8, H-7a), 1.79-1.73 (m, 1 H, H-4′a), 1.73-1.66 (m, 7 H, -CO-NH-CH_2C**H$_2$CH$_2$**-, 12-CH_3), 1.66-1.61 (m, 1 H, H-14ax), 1.44 (s, 3 H, 6-CH_3), 1.43-1.39 (m, 2 H, 2 H-cyclopropyl), 1.36-1.32 (m, 1 H, H-7b), 1.29 (d, *J* = 6.7 Hz, 3 H, 2-CH_3), 1.26 (d, *J* = 6.1 Hz, 3 H, 5′-CH_3), 1.23-1.13 (m, 3 H, 2 H-cyclopropyl, H-4′b), 1.06-1.00 (m, 6 H, 10-CH_3, 4-CH_3), 0.94-0.86 (m, 6 H, 8-CH_3, 15-CH_3). ^{13}C NMR ($CDCl_3$, 100 MHz) δ: 178.1, 176.0, 169.5, 166.7, 156.0, 153.6, 148.1, 140.0, 136.7, 129.8, 125.9, 122.7, 117.3, 108.9, 106.2, 93.0, 87.0, 86.3, 86.0, 85.5, 79.2, 77.8, 77.2, 76.1, 70.3, 69.6, 66.0, 60.7, 44.9, 40.3, 40.1, 39.2, 37.2, 35.4, 29.2, 28.9, 26.0, 25.8, 21.8, 21.7, 21.1, 20.7, 19.2, 15.9, 14.8, 10.1, 8.4, 8.3, 6.7.

3-O-descladinosyl-3-O-[N-7-(1-cyclopropyl-1,4-dihydro-4-oxo-3-quinolinecarboxylic acid-6-yl)-hept-6-

ynylcarbamoyl] azithromycin 11,12- cyclic carbonate ***(110a)***

Following the general procedure I-1, from compound **103** (0.225 g, 0.280 mmol) and 1-cyclopropyl-1,4-dihydro-4-oxo-6-iodo-3-quinolinecarboxylic acid (0.110 g, 0.310 mmol), **110a** (39.1 mg, 0.0398 mmol, 14.2 %) was obtained as an off-white solid (column chromatography eluents: DCM/MeOH/$NH_3 \cdot H_2O$ = 10:1:0.5). m.p. 142-144 ˚C. HRMS (ESI) $(M+H)^+$ *m/z* 981.5452, calcd for $C_{52}H_{77}N_4O_{14}$ 981.5431. ^1H NMR ($CDCl_3$, 400 MHz) δ: 8.83 (s, 1 H, H-quinolyl), 8.44 (d, *J* = 2.0 Hz, 1 H, H-quinolyl), 8.07 (d, *J* = 8.9 Hz, 1 H, H-quinolyl), 7.90-7.84 (m, 1 H, H-quinolyl), 6.10 (s, 1 H, 6-OH), 6.07-6.00 (m, 1 H, -CONH-), 5.13 (dd, *J* = 10.5, 1.7 Hz, 1 H, H-3), 5.05 (dd, *J* = 10.8, 2.4 Hz, 1 H, H-13), 4.59 (s, 1 H, H-11), 4.17 (d, *J* = 7.3 Hz, 1 H, H-1′), 3.70-3.63 (m, 1 H, 1 H-cyclopropyl), 3.51-3.42 (m, 2 H, H-5, H-5′), 3.39-3.27 (m, 2 H, -CONH-C**H**$_2$-, H-2′), 3.13-3.03 (m, 1 H, -CONH-C**H**$_2$-), 3.00-2.90 (m, 1 H, H-3′), 2.90-2.79 (m, 2 H, H-2, H-10), 2.54 (s, 6 H, -N(CH_3)$_2$), 2.46 (td, *J* = 6.7, 1.7 Hz, 2 H, - C**H₂**C≡C-Ar), 2.39-2.32 (m, 1 H, H-9a), 2.25 (s, 3 H, -N-CH_3), 2.18-2.10 (m, 1 H, H-4), 2.02 (t, *J* = 11.8 Hz, 1 H, H-9b), 1.92-1.75 (m, 3 H, H-8, H-14eq, H-7a), 1.70-1.51 (m, 8 H, 3(CH_2), H-14ax, H-4′a), 1.47-1.42 (m, 2 H, 2 H-cyclopropyl), 1.42 (s, 3 H, 12-CH_3), 1.36-1.26 (m, 2 H, H-7b, H-4′b), 1.25-1.17 (m, 8 H, 5′-CH_3, 2-CH_3, 2 H-cyclopropyl), 1.15 (s, 3 H, 6-CH_3), 1.10-1.04 (m, 6 H, 4-CH_3, 10-CH_3), 0.93-0.84 (m, 6 H, 8-CH_3, 15-CH_3). ^{13}C NMR ($CDCl_3$, 100 MHz) δ: 178.0, 174.5, 170.1, 166.7, 156.5, 153.4, 148.1, 140.0, 136.9, 129.4, 125.8, 122.6, 117.6, 108.8, 103.3, 92.8, 88.6, 86.4, 84.9, 79.4, 78.8, 77.3, 75.7, 72.9, 70.7, 68.6, 68.1, 65.2, 61.9, 43.4, 42.0, 40.8, 39.5, 35.9, 35.5, 34.9, 29.9, 29.7, 28.0, 26.0, 26.0, 25.7, 21.7, 21.3, 19.4, 15.7, 13.4, 10.1, 9.4, 8.4, 8.2, 5.0.

3-O-descladinosyl-3-O-[N-7-(1-cyclopropyl-1,4-dihydro-4-oxo-3-quinolinecarboxylic acid-7-yl)-hept-6-ynylcarbamoyl] azithromycin 11,12- cyclic carbonate ***(110c)***

Following the general procedure I-1, from compound **103** (0.326 g, 0.410 mmol) and **4** (0.140 g, 0.410 mmol), **110c** (18.7 mg, 0.0190 mmol, 4.63%) was obtained as an off-white solid (column chromatography eluents: DCM/MeOH/$NH_3 \cdot H_2O$ = 10:1:0.5). m.p. 135-137 ˚C. HRMS (ESI) $(M+H)^+$ *m/z* 981.5454, calcd for $C_{52}H_{77}N_4O_{14}$ 981.5431. ^1H NMR ($CDCl_3$, 400 MHz) δ: 8.86 (s, 1 H, H-quinolyl), 8.40 (d, *J* = 8.3 Hz, 1 H, H-quinolyl), 8.11 (s, 1 H, H-quinolyl), 7.55 (d, *J* = 8.4 Hz, 1 H, H-quinolyl), 6.08 (s, 1 H, 6-OH), 6.01-5.90 (m, 1 H, -CONH-), 5.10 (d, *J* = 10.0 Hz, 1 H, H-3), 5.04 (dd, *J* = 10.8, 2.4 Hz, 1 H, H-13), 4.57 (s, 1 H, H-11), 4.17 (d, *J* = 6.8 Hz, 1 H, H-1′), 3.69-3.61 (m, 1 H, H-5′), 3.52-3.48 (m, 1 H, H-5), 3.47-3.36 (m, 2 H, 1 H-cyclopropyl, -CONH-C**H**$_2$-), 3.35-2.28 (m, 1 H, H-2′), 3.09-2.89 (m, 2 H, -CONH-C**H**$_2$-, H-3′), 2.81 (q, *J* = 6.8 Hz, 1 H, H-10), 2.73-7.64 (m, 1 H, H-2), 2.55 (s, 6 H, -N(CH_3)$_2$), 2.50 (t, *J* = 6.8 Hz, 2 H, - C**H₂**C≡C-Ar), 2.39-2.32 (m, 1 H, H-9a), 2.25 (s, 3 H, -N-CH_3), 2.17-2.10 (m, 1 H, H-4), 2.06-1.98 (m, 1 H, H-9b), 1.93-1.75 (m, 3 H, H-8, H-14eq, H-7a), 1.72-1.64 (m, 2 H, -C**H₂**CH_2C≡C-Ar), 1.63-1.51 (m, 6 H, 2(CH_2), H-14ax, H-4′a), 1.48-1.44 (m, 2 H, 2 H-cyclopropyl), 1.40 (s, 3 H, 12-CH_3), 1.35-1.28 (m, 2 H, H-7b, H-4′b), 1.26-1.17 (m, 8 H, 2 H-cyclopropyl, 5′-CH_3, 6-CH_3), 1.14 (d, *J* = 6.8 Hz, 2-CH_3), 1.06 (d, *J* = 6.6 Hz, 10-CH_3), 1.01 (d, *J* = 7.3 Hz, 4-CH_3), 0.91 (d, *J* = 6.8 Hz, 8-CH_3), 0.87 (t, *J* = 7.6 Hz, 15-CH_3). ^{13}C NMR ($CDCl_3$, 100 MHz) δ: 178.2, 174.3, 166.8, 156.5, 153.4, 148.5, 141.1, 130.1, 129.4, 126.8, 124.8, 120.1, 108.9, 103.4, 95.6, 88.8, 86.4, 84.8, 80.1, 78.7, 77.2, 75.7, 72.8, 70.7, 68.7, 68.0, 65.3, 61.9, 43.3, 42.0, 40.7, 39.6, 35.8, 35.5, 34.9, 29.7, 27.8, 26.0, 25.9, 25.7, 21.7, 21.3, 21.1, 19.5, 15.6, 13.4, 10.1, 9.4, 8.4, 8.3, 5.0.

3-O-descladinosyl-3-O-[N-8-(1-cyclopropyl-1,4-dihydro-4-oxo-3-quinolinecarboxylic acid-6-yl)-otc-7-ynylcarbamoyl] azithromycin 11,12- cyclic carbonate ***(111a)***

Following the general procedure I-1, from compound **104** (0.500 g, 0.620 mmol) and 1-cyclopropyl-1,4-dihydro-4-oxo-6-iodo-3-quinolinecarboxylic acid (0.240 g, 0.680 mmol), **111a** (31.3 mg, 0.0314 mmol, 50.6%) was obtained as an off-white solid (column chromatography eluents: DCM/MeOH/$NH_3 \cdot H_2O$ = 10:1:0.5). m.p. 137-140 ˚C. HRMS (ESI) $(M+H)^+$ *m/z* 995.5586, calcd for $C_{53}H_{79}N_4O_{14}$ 995.5587. ^1H NMR ($CDCl_3$, 400 MHz) δ: 8.84 (s, 1 H, H-quinolyl), 8.45 (d, *J* = 2.0 Hz, 1 H, H-quinolyl), 8.07 (d, *J* = 8.8 Hz, 1 H, H-quinolyl), 7.86 (dd, *J* = 8.7, 2.0 Hz, 1 H, H-quinolyl), 6.07 (s, 1 H, 6-OH), 5.96-5.90 (m, 1 H, -CONH-), 5.14 (dd, *J* = 10.5, 1.8 Hz, 1 H, H-3), 5.05 (dd, *J* = 10.8, 2.5 Hz, 1 H, H-13), 4.59 (s, 1 H, H-11), 4.17 (d, *J* = 7.3 Hz, 1 H, H-1′), 3.71-3.62 (m, 1 H, 1 H-cyclopropyl), 3.51-3.49 (m, 1 H, H-5), 3.48-3.41 (m, 1 H, H-5′), 3.37-3.27 (m, 2 H, -CONH-C**H**$_2$-, H-2′), 3.14-3.04 (m, 1 H, -CONH-C**H**$_2$-), 2.99-2.91 (m, 1 H, H-3′), 2.89-2.80 (m, 2 H, H-2, H-10), 2.54 (s, 6 H, -N(CH_3)$_2$), 2.45 (t, *J* = 6.8 Hz,

2 H, -C**H**$_2$C≡C-Ar), 2.36 (dd, *J* = 12.3, 2.9 Hz, H-9a), 2.25 (s, 3 H, -N-CH$_3$), 2.19-2.11 (m, 1 H, H-4), 2.02 (t, *J* = 12.0 Hz, 1 H, H-9b), 1.92-1.75 (m, 3 H, H-8, H-14eq, H-7a), 1.67-1.48 (m, 8 H, -CONH-CH$_2$C**H**$_2$C**H**$_2$CH$_2$C**H**$_2$CH$_2$C≡C-Ar, H-14ax, H-4′a), 1.46-1.39 (m, 7 H, -C**H**$_2$CH$_2$CH$_2$C≡C-Ar, 2 H-cyclopropyl, 12-CH$_3$), 1.36-1.26 (m, 2 H, H-7b, H-4′b), 1.24-1.16 (m, 11 H, 5′-CH$_3$, 2-CH$_3$, 2 H-cyclopropyl, 6-CH$_3$), 1.10-1.04 (m, 6 H, 4-CH$_3$, 10-CH$_3$), 0.93-0.86 (m, 6 H, 8-CH$_3$, 15-CH$_3$). ^{13}C NMR (CDCl$_3$, 100 MHz) δ: 178.1, 174.5, 170.1, 166.7, 156.5, 153.4, 148.1, 140.0, 136.8, 129.5, 125.8, 122.7, 117.6, 108.8, 103.3, 93.1, 88.6, 86.4, 84.9, 79.2, 78.7, 77.3, 75.7, 72.9, 70.7, 68.7, 68.1, 65.3, 61.9, 43.4, 42.0, 41.0, 39.6, 35.9, 35.5, 34.9, 30.1, 29.7, 28.5, 28.3, 26.2, 26.0, 25.7, 21.7, 21.3, 21.1, 19.4, 15.7, 13.4, 10.1, 9.4, 8.3, 8.3, 5.0.

3-O-descladinosyl-3-O-[N-8-(1-cyclopropyl-1,4-dihydro-4-oxo-3-quinolinecarboxylic acid-7-yl)-otc-7-ynylcarbamoyl] azithromycin 11,12- cyclic carbonate ***(111c)***

Following the general procedure I-1, from compound **104** and **4**, **111c** was obtained as an off-white solid (column chromatography eluents: DCM/MeOH/NH$_3$·H$_2$O = 10:1:0.5). HRMS (ESI) (M+H)$^+$ *m/z* 995.5601, calcd for $C_{53}H_{79}N_4O_{14}$ 995.5587. ^{1}H NMR (CDCl$_3$, 400 MHz) δ: 8.87 (s, 1 H, 2″-quinolyl), 8.41 (d, *J* = 8.3 Hz, 1 H, 5″-quinolyl), 8.08 (s, 1 H, 8″-quinolyl), 7.55 (d, *J* = 8.1 Hz, 1 H, 6″-quinolyl), 6.08 (s, 1 H, 6-OH), 5.96 (s, 1 H, -CONH-), 5.13 (d, *J* = 10.2 Hz, 1 H, H-3), 5.06 (dd, *J* = 2.5 Hz, 10.7 Hz, 1 H, H-13), 4.57 (s, 1 H, H-11), 4.18 (d, *J* = 7.3 Hz, 1 H, H-1′), 3.66-3.57 (m, 1 H, 1 H-cyclopropyl), 3.54 (s, 1 H, H-5), 3.50-3.41 (m, 1 H, H-5′), 3.40-3.26 (m, 2 H, -CONH-C**H**$_2$-, H-2′), 3.10-2.90 (m, 2 H, -CONH-C**H**$_2$-, H-3′), 2.89-2.76 (m, 2 H, H-2, H-10), 2.57 (s, 6 H, -N(CH$_3$)$_2$), 2.49 (t, *J* = 7.0 Hz, 2 H, -C**H**$_2$C≡C-Ar), 2.36 (dd, *J* = 2.9 Hz, 12.3 Hz, H-9a), 2.25 (s, 3 H, -N-CH$_3$), 2.19-2.10 (m, 1 H, H-4), 2.02 (t, *J* = 11.6 Hz, 1 H, H-9b), 1.94-1.76 (m, 3 H, H-8, H-14eq, H-7a), 1.67-1.48 (m, 8 H, 3(CH$_2$), H-14ax, H-4′a), 1.72-1.36 (m, 7 H, -C**H**$_2$CH$_2$CH$_2$C≡C-Ar, 2 H-cyclopropyl, 12-CH$_3$), 1.37-1.27 (m, 2 H, H-7b, H-4′b), 1.27-1.14 (m, 11 H, 5′-CH$_3$, 2-CH$_3$, 2 H-cyclopropyl, 6-CH$_3$), 1.11-0.98 (m, 6 H, 4-CH$_3$, 10-CH$_3$), 0.96-0.83 (m, 6 H, 8-CH$_3$, 15-CH$_3$). ^{13}C NMR (CDCl$_3$, 100 MHz) δ: 178.24, 174.30, 166.80, 156.42, 153.39, 153.39, 148.50, 141.11, 129.42, 126.88, 124.85, 119.88, 109.01, 95.85, 88.54, 86.26, 84.89, 79.91, 78.66, 77.34, 77.22, 76.70, 75.83, 72.87, 61.84, 43.42, 35.41, 34.89, 30.13, 28.55, 28.29, 26.23, 26.02, 25.72, 21.74, 21.34, 21.00, 19.53, 15.56, 13.57, 10.11, 9.49, 8.36.

synthesis of compounds 116-117

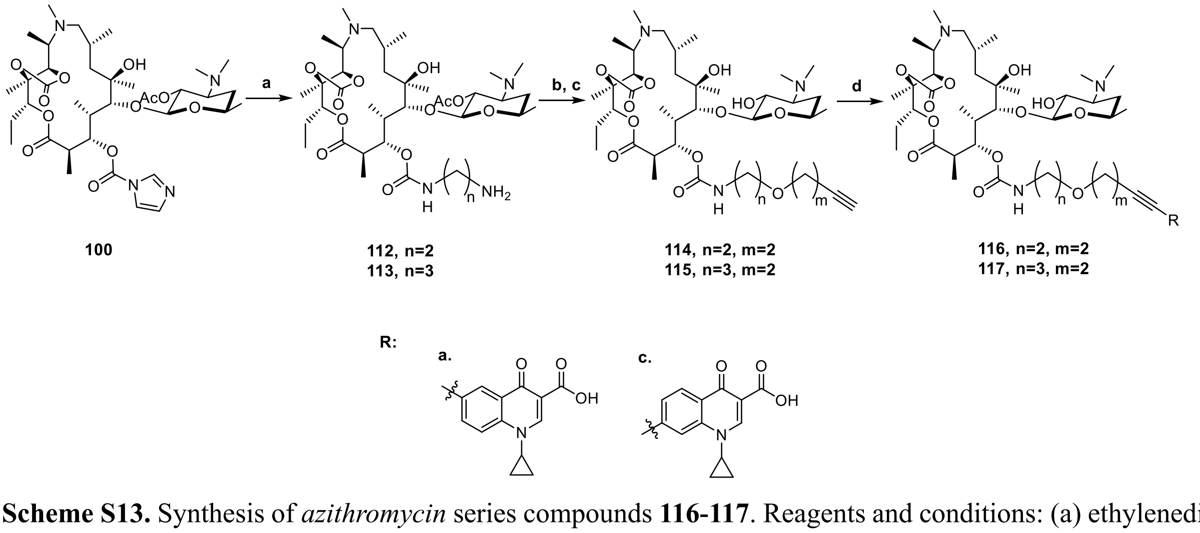

Scheme S13. Synthesis of *azithromycin* series compounds **116-117**. Reagents and conditions: (a) ethylenediamine or 1,3-diaminopropane, DMF, rt, 3 h; (b) CH$_3$OH, 65 ˚C, 2 h; (c) 3-butyn-1-ol, NaNO$_2$, HCOOH, -15˚C, 12-72 h; (d) R-I (R = **a**, **c**), CuI, Pd(PPh$_3$)$_2$Cl$_2$, Et$_3$N, CH$_3$CN, 45 ˚C, 12 h.

2′-O-Acetyl-3-O-descladinosyl-3-O-(N-ethylamine-1-ylcarbamoyl) azithromycin 11,12- cyclic carbonate ***(112)***

Following the general procedure K, from compound **100** (4.00 g, 5.31 mmol) and ethylenediamine (1.77 mL,

26.6 mmol), **112** (2.06 g, 2.76 mmol, 52.0%) was obtained as an off-white solid (column chromatography eluents: DCM/MeOH/$NH_3 \cdot H_2O$ = 10:1:0.1). HRMS (ESI) $(M+H)^+$ *m/z* 745.4588, calcd for $C_{36}H_{65}N_4O_{12}$ 745.4593. ^{1}H NMR ($CDCl_3$, 400 MHz) δ: 6.03 (s, 1 H, 6-OH), 5.54-5.47 (m, 1 H, -CONH-), 5.16 (dd, *J* = 10.1, 2.2 Hz, 1 H, H-3), 5.06 (dd, *J* = 10.8, 2.5 Hz, 1 H, H-13), 4.76 (dd, *J* = 10.6, 7.5 Hz, 1 H, H-2′), 4.61 (s, 1 H, H-11), 4.25 (d, *J* = 7.6 Hz, 1 H, H-1′), 3.57 (d, *J* = 2.3 Hz, 1 H, H-5), 3.49-3.34 (m, 2 H, H-5′, -CONH-C**H**$_2$-), 3.22-3.13 (m, 1 H, -CONH-C**H**$_2$-), 2.95-2.79 (m, 4 H, H-3′, -CONH-CH_2C**H$_2$**-, H-10), 2.76-2.68 (m, 2 H, H-2), 2.34 (dd, *J* = 12.5, 3.1 Hz, H-9a), 2.28 (s, 6 H, -N(CH_3)$_2$), 2.25 (s, 3 H, -N-CH_3), 2.17-2.10 (m, 1 H, H-4), 2.08 (s, 3 H, 2′-O-CO-CH_3), 2.02 (t, *J* = 11.9 Hz, 1 H, H-9b), 1.92-1.80 (m, 2 H, H-8, H-14eq), 1.74-1.68 (m, 1 H, H-7a), 1.63-1.55 (m, 1 H, H-14ax), 1.54-1.48 (m, 1 H, H-4′a), 1.41 (s, 3 H, 12-CH_3), 1.35-1.29 (m, 1 H, H-7b), 1.28-1.22 (m, 1 H, H-4′b), 1.22-1.17 (m, 9 H, 5′-CH_3, 6-CH_3, 2-CH_3), 1.06 (d, *J* = 6.6 Hz, 3 H, 10-CH_3), 0.95-0.87 (m, 9 H, 4-CH_3, 8-CH_3, 15-CH_3).

*2′-O-Acetyl-3-O-descladinosyl-3-O-(N-aminopropylcarbamoyl) azithromycin 11,12- cyclic carbonate **(113)***

Following the general procedure K, from compound **100** (2.00 g, 2.66 mmol) and 1,3-diaminopropane (1.11 mL, 13.3 mmol), **113** (0.830 g, 1.09 mmol, 41.0%) was obtained as an off-white solid (column chromatography eluents: DCM/MeOH/$NH_3 \cdot H_2O$ = 10:1:0.1).

*3-O-descladinosyl-3-O-(N-but-3-ynoxy-ethylcarbamoyl) azithromycin 11,12- cyclic carbonate **(114)***

Following the general procedure L, from compound **112** (1.70 g, 2.42 mmol) and 3-butyn-1-ol (18.3 mL, 241 mmol), **114** (0.348 g, 0.460 mmol, 19.0 %) was obtained as an off-white solid (column chromatography eluents: DCM/MeOH/$NH_3 \cdot H_2O$ = 10:0.3:0.05). HRMS (ESI) $(M+H)^+$ *m/z* 756.4625, calcd for $C_{38}H_{66}N_3O_{12}$ 756.4641. ^{1}H NMR ($CDCl_3$, 400 MHz) δ: 6.02 (s, 1 H, 6-OH), 5.43-5.37 (m, 1 H, -CONH-), 5.16 (dd, *J* = 10.3, 2.2 Hz, 1 H, H-3), 5.07 (dd, *J* = 10.7, 2.5 Hz, 1 H, H-13), 4.62 (s, 1 H, H-11), 4.14 (d, *J* = 7.3 Hz, 1 H, H-1′), 3.62-3.50 (m, 6 H, H-5, H-5′, -C**H$_2$**-O-C**H$_2$**-), 3.46-3.36 (m, 1 H, -CONH-C**H**$_2$-), 3.29-3.19 (m, 2 H, -CONH-C**H**$_2$-, H-2′), 2.93-2.80 (m, 2 H, H-3′, H-10), 2.46 (dt, *J* = 6.7, 2.6 Hz, 3 H, -C**H$_2$**C≡CH, H-2), 2.39-2.33 (m, 1 H, H-9a), 2.30 (s, 6 H, -N(CH_3)$_2$), 2.26 (s, 3 H, -N-CH_3), 2.20-2.12 (m, 1 H, H-4), 2.08-1.98 (m, 2 H, H-9b, -CH2C≡C**H**), 1.95-1.80 (m, 2 H, H-8, H-14eq), 1.68-1.55 (m, 3 H, H-7a, H-14ax, H-4′a), 1.42 (s, 3 H, 12-CH_3), 1.35-1.28 (m, 1 H, H-7b), 1.26-1.23 (m, 4 H, H-4′b, 6-CH_3), 1.23-1.17 (m, 6 H, 5′-CH_3, 2-CH_3), 1.09-1.04 (m, 6 H, 10-CH_3, 4-CH_3), 0.94-0.87 (m, 6 H, 8-CH_3, 15-CH_3).

*3-O-descladinosyl-3-O-(N-but-3-ynoxy-propylcarbamoyl) azithromycin 11,12- cyclic carbonate **(115)***

Following the general procedure L, from compound **113** (0.830 g, 1.16 mmol) and 3-butyn-1-ol (4.40 mL, 58.0 mmol), **115** (0.247 g, 0.321 mmol, 27.7%) was obtained as an off-white solid (column chromatography eluents: DCM/MeOH/$NH_3 \cdot H_2O$ = 10:0.3:0.05).

*3-O-descladinosyl-3-O-[N-4-(1-cyclopropyl-1,4-dihydro-4-oxo-3-quinolinecarboxylic acid-6-yl)-but-3-ynoxy-ethylcarbamoyl] azithromycin 11,12- cyclic carbonate **(116a)***

Following the general procedure I-1, from compound **114** (0.348 g, 0.460 mmol) and 1-cyclopropyl-1,4-dihydro-4-oxo-6-iodo-3-quinolinecarboxylic acid (0.196 g, 0.418 mmol), **116a** (70.1 mg, 0.0713 mmol, 15.5%) was obtained as an off-white solid (column chromatography eluents: DCM/MeOH/$NH_3 \cdot H_2O$ = 10:1:0.5). m.p. 137-139 ˚C. HRMS (ESI) $(M+H)^+$ *m/z* 983.5249, calcd for $C_{51}H_{75}N_4O_{15}$ 983.5223. ^{1}H NMR ($CDCl_3$, 400 MHz) δ: 8.84 (s, 1 H, H-quinolyl), 8.48 (d, *J* = 2.0 Hz, 1 H, H-quinolyl), 8.07 (d, *J* = 8.8 Hz, 1 H, H-quinolyl), 7.90 (d, *J* = 8.6 Hz, 1 H, H-quinolyl), 6.05 (s, 1 H, 6-OH), 5.91 (t, *J* = 5.9 Hz, 1 H, -CONH-), 5.15 (dd, *J* = 10.5, 1.9 Hz, 1 H, H-3), 5.05 (dd, *J* = 10.7, 2.5 Hz, 1 H, H-13), 4.58 (s, 1 H, H-11), 4.15 (d, *J* = 7.3 Hz, 1 H, H-1′), 3.74-3.60 (m, 5 H, 1 H-cyclopropyl, -C**H$_2$**-O-C**H$_2$**-), 3.56-3.45 (m, 3 H, H-5′, -CONH-C**H**$_2$-, H-5), 3.39-3.31 (m, 1 H, -CONH-C**H**$_2$-), 3.31-3.24 (m, 1 H, H-2′), 2.89-2.78 (m, 3 H, H-2, H-10, H-3′), 2.72 (dt, *J* = 6.7, 2.2 Hz, -C**H$_2$**C≡C-Ar), 2.47 (s, 6 H, -N(CH_3)$_2$), 2.38-2.33 (m, 1 H, H-9a), 2.24 (s, 3 H, -N-CH_3), 2.16-2.09 (m, 1 H, H-4), 2.02 (t, *J* = 11.9 Hz, H-9b), 1.91-1.80 (m, 2 H, H-8, H-14eq), 1.77-1.71 (m, 1 H, H-4′a), 1.64-1.52 (m, 2 H, H-14ax, H-7a), 1.46-1.39 (m, 5 H, 2 H-cyclopropyl, 12-CH_3), 1.33-1.24 (m, 2 H, H-7b, H-4′b), 1.24-1.17 (m, 8 H, 2 H-cyclopropyl, 10-CH_3, 5′-CH_3), 1.15 (s, 3 H, 6-CH_3), 1.09-1.00 (m, 6 H, 2-CH_3, 4-CH_3), 0.93-0.86 (m, 6 H, 8-CH_3, 15-CH_3). ^{13}C NMR ($CDCl_3$, 100 MHz) δ: 178.0, 174.5, 166.7, 156.5, 153.4, 148.1, 140.2, 136.8, 129.7, 125.9, 122.2, 117.6, 109.0, 103.3, 89.6, 88.1, 86.4, 84.9, 80.0, 79.0, 77.2, 78.8, 72.9, 70.7, 69.8, 68.8, 68.8, 68.1, 65.4, 61.8, 43.4, 42.1, 40.8, 39.8, 36.0, 35.5, 34.9, 29.6, 26.0, 25.7, 21.7,

21.3, 21.1, 20.8, 15.6, 13.5, 10.1, 9.4, 8.4, 8.2, 5.0.

3-O-descladinosyl-3-O-[N-4-(1-cyclopropyl-1,4-dihydro-4-oxo-3-quinolinecarboxylic acid-7-yl)-but-3-ynoxy-ethylcarbamoyl] azithromycin 11,12- cyclic carbonate ***(116c)***

Following the general procedure I-1, from compound **114** and **4**, **116a** was obtained as an off-white solid (column chromatography eluents: DCM/MeOH/$NH_3 \cdot H_2O$ = 10:1:0.5). HRMS (ESI) $(M+H)^+$ *m/z* 983.5231, calcd for $C_{51}H_{75}N_4O_{15}$ 983.5223. ^1H NMR ($CDCl_3$, 400 MHz) δ: 8.86 (s, 1 H, H-quinolyl), 8.40 (d, *J* = 8.4 Hz, 1 H, H-quinolyl), 8.17 (s, 1 H, H-quinolyl), 7.59 (d, *J* =1.3 Hz, 8.3 Hz, 1 H, H-quinolyl), 6.22 (s, 1 H, 6-OH), 6.07 (s, 1 H, -CONH-), 5.03 (dd, *J* = 2.4 Hz,10.8 Hz, 1 H, H-3), 5.09 (dd, *J* = 1.9 Hz, 10.4 Hz, 1 H, H-13), 4.54 (s, 1 H, H-11), 4.16 (d, *J* = 7.3 Hz, 1 H, H-1′), 3.80-3.52 (m, 6 H, 1 H-cyclopropyl, -C**H2**-O-C**H2**-, H-5′), 3.51-3.38 (m, 2 H, -CONH-C**H**2-, H-5), 3.34-3.18 (m, 2 H, -CONH-C**H**2-, H-2′), 3.02-2.89 (m, 1 H, H-3′), 2.85-2.71 (m, 3 H, H-10, -C**H2**C≡C-Ar), 2.47 (s, 7 H, H-2, -N(CH_3)2), 2.43-2.30 (m, 1 H, H-9a), 2.24 (s, 3 H, -N-CH_3), 2.15-1.97 (m, 2 H, H-4, H-9b), 1.95-1.72 (m, 2 H, H-8, H-14eq, H-4′a), 1.69-1.50 (m, 2 H, H-14ax, H-7a), 1.51-1.36 (m, 5 H, 2 H-cyclopropyl, 12-CH_3), 1.36-1.27 (m, 2 H, H-7b, H-4′b), 1.24-1.16 (m, 8 H, 2 H-cyclopropyl, 10-CH_3, 6-CH_3), 1.12 (d, *J* = 6.8 Hz, 3 H, 5′-CH_3), 1.06 (d, *J* = 6.5 Hz, 3 H, 2-CH_3), 0.99-0.83 (m, 9 H, 8-CH_3, 4-CH_3,15-CH_3). ^{13}C NMR ($CDCl_3$, 100 MHz) δ: 178.14, 174.37, 166.75, 156.61, 153.43, 148.53, 141.13, 129.68, 129.36, 126.79, 124.96, 120.36, 108.90, 103.26, 92.42, 88.47, 86.37, 84.85, 80.62, 78.83, 75.78, 72.86, 70.60, 69.74, 68.57, 68.53, 67.99, 65.27, 61.84, 43.19, 42.01, 40.68, 39.47, 35.83, 35.52, 34.91, 29.71, 25.99, 25.68, 21.66, 21.31, 21.06, 20.94, 15.59, 13.37, 10.11, 9.35, 8.47, 8.25, 4.89.

3-O-descladinosyl-3-O-[N-4-(1-cyclopropyl-1,4-dihydro-4-oxo-3-quinolinecarboxylic acid-6-yl)-but-3-ynoxy-propylcarbamoyl] azithromycin 11,12- cyclic carbonate ***(117a)***

Following the general procedure I-1, from compound **115** (0.247 g, 0.321 mmol) and 1-cyclopropyl-1,4-dihydro-4-oxo-6-iodo-3-quinolinecarboxylic acid (0.237 g, 0.506 mmol), **116a** (54.9 mg, 0.0550 mmol, 17.1 %) was obtained as an off-white solid (column chromatography eluents: DCM/MeOH/$NH_3 \cdot H_2O$ = 10:1:0.5). m.p. 136-138 °C. HRMS (ESI) $(M+H)^+$ *m/z* 997.5390, calcd for $C_{52}H_{77}N_4O_{15}$ 997.5380. ^1H NMR ($CDCl_3$, 500 MHz) δ: 8.84 (s, 1 H, H-quinolyl), 8.47 (d, *J* = 2.1 Hz, 1 H, H-quinolyl), 8.08 (d, *J* = 8.9 Hz, 1 H, H-quinolyl), 7.89 (dd, *J* = 8.8, 2.1 Hz, 1 H, H-quinolyl), 6.07 (s, 1 H, 6-OH), 5.62-5.56 (m, 1 H, -CONH-), 5.14 (dd, *J* = 10.4, 2.0 Hz, 1 H, H-3), 5.04 (dd, *J* = 10.8, 2.5 Hz, 1 H, H-13), 4.59 (s, 1 H, H-11), 4.12 (d, *J* = 7.3 Hz, 1 H, H-1′), 3.71-3.64 (m, 3 H, 1 H-cyclopropyl, -C≡C-CH_2C**H2**-), 3.63-3.57 (m, 2 H, -O-C**H2**$CH_2$$CH_2$-CONH-), 3.52 (d, *J* = 2.1 Hz, 1 H, H-5), 3.46-3.39 (m, 2 H, -CONH-C**H**2-, H-5′), 3.30-3.22 (m, 2 H, -CONH-C**H**2-, H-2′), 2.88-2.80 (m, 2 H, H-2, H-10), 2.72 (t, *J* = 6.7 Hz, -C**H2**C≡C-Ar), 2.66-2.58 (m, 1 H, H-3′), 2.38 (s, 6 H, -N(CH_3)2), 2.36-2.29 (m, 1 H, H-9a), 2.25 (s, 3 H, -N-CH_3), 2.16-2.10 (m, 1 H, H-4), 2.02 (t, *J* = 11.9 Hz, H-9b), 1.93-1.79 (m, 4 H, H-8, -CONH-CH_2C**H2**-, H-14eq), 1.72-1.66 (m, 1 H, H-4′a), 1.63-1.53 (m, 2 H, H-7a, H-14ax), 1.47-1.43 (m, 2 H, 2 H-cyclopropyl), 1.42 (s, 3 H, 12-CH_3), 1.33-1.24 (m, 2 H, H-7b, H-4′b), 1.23-1.13 (m, 11 H, 2 H-cyclopropyl, 6-CH_3, 10-CH_3, 5′-CH_3), 1.06 (t, *J* = 7.2 Hz, 6 H, 2-CH_3, 4-CH_3), 0.92-0.86 (m, 6 H, 8-CH_3, 15-CH_3). ^{13}C NMR ($CDCl_3$, 125 MHz) δ: 178.0, 174.5, 170.3, 166.7, 156.4, 153.4, 148.2, 140.2, 136.9, 129.6, 125.8, 122.3, 117.6, 108.9, 103.5, 89.8, 88.0, 86.3, 84.9, 79.9, 78.8, 77.3, 75.7, 72.9, 70.7, 69.1, 68.9, 68.9, 68.0, 65.7, 61.8, 43.3, 42.1, 40.1, 38.8, 36.0, 35.5, 34.9, 34.9, 30.0, 29.1, 26.0, 25.8, 21.7, 21.3, 21.1, 20.9, 15.6, 13.5, 10.1, 9.4, 8.7, 8.3, 8.2, 5.0.

Synthesis of compounds 126-131

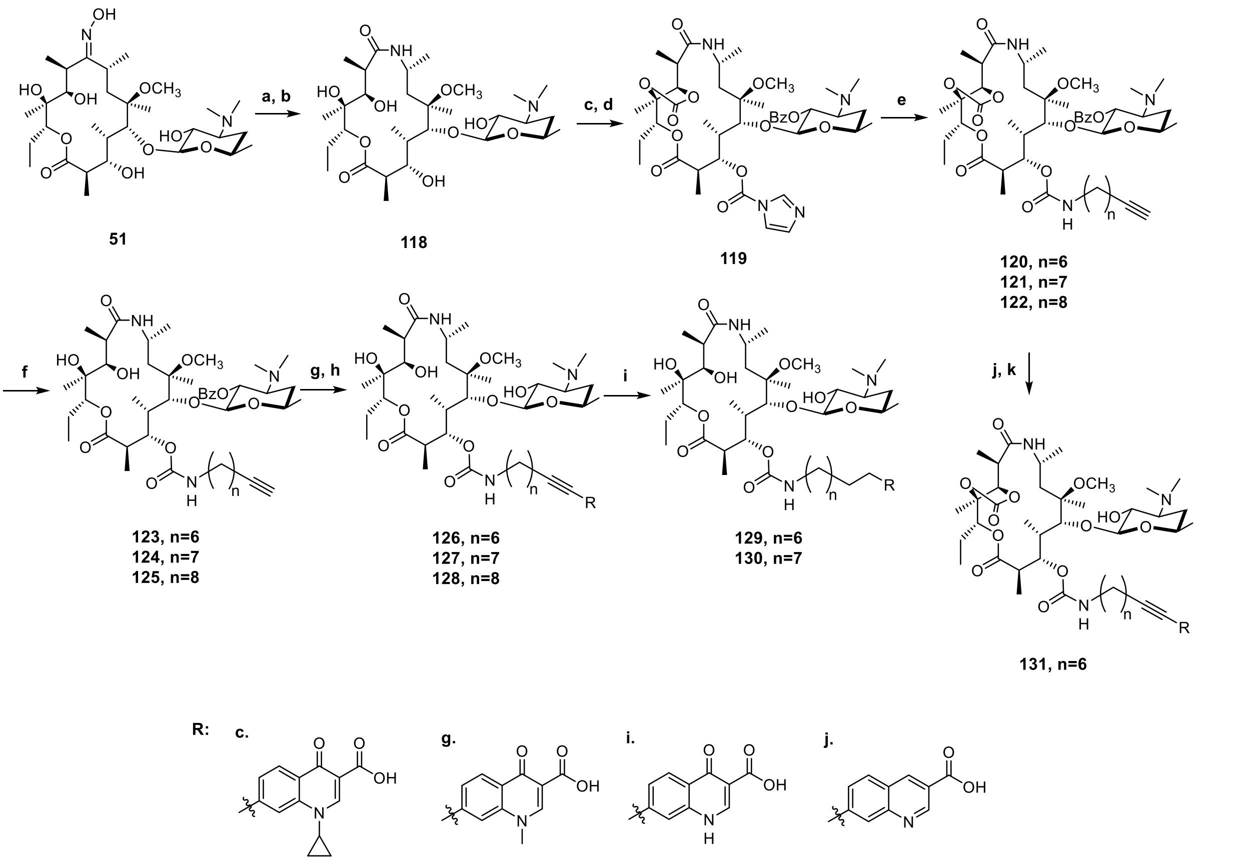

Scheme S14. Synthesis of *8a-azahomoerythromycin* series compounds **126-131**. Reagents and conditions: (a) LiOH·H_2O, CH_3CH_2OH, rt, 72 h; (b) tosyl chloride, $NaHCO_3$, acetone/ H_2O, rt, 2 h; (c) benzoic anhydride, CH_2Cl_2, rt, 12 h; (d) CDI, DMAP, DCM, rt, 12 h; (e) **29-31**, DBU, DMF, rt, 12 h; (f) LiOH·H_2O, THF/ H_2O, rt, 2 h; (g) R-I (R = **c**, **g**, **i**), CuI, $Pd(PPh_3)_2Cl_2$, Et_3N, CH_3CN, 45 ˚C, 12 h; or R-Br (R = **j**-ethyl ester), CuI, $Pd(PPh_3)_2Cl_2$, Et_3N, CH_3CN, 80 ˚C, 4 h; (h) CH_3OH, 65 ˚C, 12 h; (i) HCOOH, $HCOONH_4$, 10% Pd/C, H_2, CH_3OH, rt, 16 h; (j) R-I (R = **i**), CuI, $Pd(PPh_3)_2Cl_2$, Et_3N, CH_3CN, 45 ˚C, 12 h; (k) CH_3OH, 65 ˚C, 12 h.

3-O-descladinosyl-3-hydroxy-8a-azahomoerythromycin ***(118)***

Compound **51** (12.0 g, 19.8 mmol) and LiOH·H_2O (2.78 g, 66.0 mmol) were dissolved in EtOH (120 mL), and the reaction was stirred at room temperature for 72 h. After evaporation of EtOH, the residue was redissolved in CH_2Cl_2 (100 mL). The CH_2Cl_2 layer was washed with brine, evaporated in vacuum, and purified by column chromatography (100-200 mesh silicone, CH_2Cl_2/EtOH/NH_3.H_2O=10/0.2/0.1) to yield an intermediate (3.20 g, 5.29 mmol, 32.1%).

The intermediate (3.85 g, 6.37 mmol) obtained in the previous step was dissolved in acetone (8 mL), and a solution of $NaHCO_3$ (1.94 g, 10.2 mmol) in H_2O (16 mL) was added. The mixture was stirred at 0 ˚C followed by the addition of tosyl chloride (1.94 g, 10.2 mmol) in acetone (8 mL). The mixture was stirred at 25 ˚C for 1 h, then the mixture was evaporated in vacuum. The pH of mixture was adjusted to 10 with 2 N NaOH. The CH_2Cl_2 was added to the reaction, The CH_2Cl_2 layer was washed with brine, and evaporated under vacuum to yield **118** (3.00 g, 4.96 mmol, 78.0 %).

2'-O-benzoyl-3-O-descladinosyl-3-O-(1H-imidazol-1-ylcarbonyl)-8a-azahomoerythromycin 11,12- cyclic carbonate ***(119)***

To a stirred solution of **118** (3.00 g, 4.96 mmol) in CH_2Cl_2 (30 mL), benzoic anhydride (1.54 g, 7.44 mmol) was added, and the reaction mixture was stirred at rt for 12 h. After completion of the reaction, the reaction mixture was washed with water and brine. The organic layer was concentrated in vacuo and purified by column chromatography

(100-200 mesh silicone, $CH_2Cl_2/EtOH/NH_3.H_2O$=10/0.1/0.05) to yield intermediate (2.80 g, 3.95 mmol, 79.6%). HRMS (ESI) $(M + H)^+$ *m/z* 709.4284, calcd for $C_{37}H_{60}N_2O_{11}$ 709.4270. ^1H NMR ($CDCl_3$, 400 MHz) δ: 8.07 (d, *J* = 7.2 Hz, 2 H, 2 H-Bz), 7.57 (t, *J* = 7.2 Hz, 1 H, 1 H-Bz), 7.45 (t, *J* = 7.6 Hz, 2 H, 2 H-Bz), 6.59 (d, *J* = 8.8 Hz, 1 H, 8a-CO-NH-), 5.12 (dd, *J* = 10.4, 7.6 Hz, 1 H, H-2′), 4.99 (dd, *J* = 10.8, 2 Hz, 1 H, H-13), 4.75 (s, 1 H, 11-OH), 4.60 (d, *J* = 7.6 Hz, 1 H, H-1′), 4.08-4.04 (m, 1 H, H-8), 3.84 (q, *J* = 9.6, 6.8 Hz, 1 H, H-3), 3.67 (d, *J* = 4.0 Hz, 1 H, H-5), 3.62-3.57 (m, 1 H, H-5′), 3.38 (s, 1 H, H-11), 3.14 (s, 1 H, 12-OH), 3.12 (s, 3 H, 6-*O*-CH_3), 2.98-2.92 (m, 1 H, H-3′), 2.51-2.26 (m, 1 H, H-2), 2.28 (s, 6 H, -N(CH_3)$_2$), 2.21(q, *J* = 15.6, 10.4 Hz, 1 H, H-7a), 2.01 (q, *J* = 13.6, 7.2 Hz, 1 H, H-10), 1.91 (t, *J* = 7.6 Hz, 1 H, H-4), 1.85-1.79 (m, 2 H, H-14ax, H-4′a), 1.70 (d, *J* = 6.8 Hz, 1 H, 3-OH), 1.62 (q, *J* = 15.2, 4.8 Hz, 1 H, H-7b), 1.45 (q, *J* = 12.4 Hz, 1 H, H-4′b), 1.36-1.31 (m, 1H, H-14eq), 1.29 (d, *J* = 6.0 Hz, 3 H, 5′-CH_3), 1.26 (s, 3 H, 6-CH_3), 1.19-1.15 (m, 9 H, 10-CH_3, 8-CH_3, 2-CH_3), 0.83 (d, *J* = 7.2 Hz, 3 H, 4-CH_3), 0.77 (t, *J* = 7.2 Hz, 3 H, 15-CH_3), 0.45 (s, 3 H, 12-CH_3).

The intermediate (2.80 g, 3.95 mmol), CDI (1.92 g, 11.8 mmol) and DMAP (0.965 g, 7.90 mmol) were dissolved in dry CH_2Cl_2 (4 mL). The mixture was stirred at 25 ˚C for 12 h followed by the addition of CH_2Cl_2 (50 mL) and water (50 mL). The organic layer was washed with water and brine, and concentrated in vacuo to yield **119** (3.00 g, 3.62 mmol, 91.6%).

2′-O-benzoyl-3-O-descladinosyl-3-O-(N-7-octynylcarbamoyl)-8a-azahomoerythromycin 11,12- cyclic carbonate ***(120)***

Following the general procedure H, from compound **119** (0.525 g, 0.630 mmol) and **29** (0.124 g, 0.750 mmol), **120** (0.284 g, 0.320 mmol, 50.8%) was obtained as an off-white solid (column chromatography eluents: petroleum ether/DCM/EtOH/$NH_3·H_2O$ = 3:7:0.1:0.05). HRMS (ESI) $(M + H)^+$ *m/z* 886.5058, calcd for $C_{47}H_{71}N_3O_{13}$ 886.5060. ^1H NMR ($CDCl_3$, 400 MHz) δ: 8.03 (d, *J* = 7.6 Hz, 2 H, 2H-Bz), 7.57 (t, *J* = 7.6 Hz, 1 H, 1 H-Bz), 7.45 (t, *J* = 7.6 Hz, 2 H, 2 H-Bz), 5.89 (d, *J* = 8.0 Hz, 1 H, 8a-CO-NH-), 5.11 (d, 1 H, *J* = 10.0 Hz, H-3), 4.97-5.04 (m, 2 H, H-2′, H-13), 4.80 (t, 1 H, *J* = 9.6 Hz, 3-*O*-CO-NH-), 4.46 (d, *J* = 8.8 Hz, 1 H, H-11), 4.34 (d, *J* = 9.6 Hz, 1 H, H-1′), 3.99-3.96 (m, 1 H, H-8), 3.71 (d, *J* = 5.2 Hz, 1 H, H-5), 3.50-3.46 (m, 1 H, H-5′), 3.32-3.10 (m, 5 H, 6-*O*-CH_3, -CONH-C**H₂**-), 2.90-2.85 (m, 1 H, H-3′), 2.69-2.65 (m, 1 H, H-2), 2.29 (s, 6 H, -N(CH_3)$_2$), 2.25-2.17 (m, 3 H, H-10, -C**H₂**-C≡CH), 1.94 (t, *J* = 2.8 Hz, 1H, -C≡CH), 1.87-1.78 (m, 1 H, H-4), 1.93-1.88 (m, 2 H, H-14eq, H-7a), 1.75-1.68 (m, 1 H, H-4′a), 1.67-1.38 (m, 11 H, H-4′b, H-14ax, H-7b, 4(CH_2)), 1.29-1.27 (m, 9 H, 5′-CH_3, 6-CH_3, 12-CH_3), 1.21 (d, *J* = 6.8 Hz, 3 H, 8-CH_3), 1.13-1.12 (m, 6 H, 10-CH_3, 2-CH_3), 0.87 (t, *J* = 7.2 Hz, 3 H, 15-CH_3), 0.73 (d, *J* = 6.8 Hz, 3 H, 4-CH_3).

2′-O-benzoyl-3-O-descladinosyl-3-O-(N-8-nonynylcarbamoyl)-8a-azahomoerythromycin 11,12- cyclic carbonate ***(121)***

Following the general procedure H, from compound **119** (2.00 g, 2.41 mmol) and **30** (0.511 g, 2.88 mmol), **121** (1.24 g, 1.38 mmol, 57.3%) was obtained as an off-white solid (column chromatography eluents: petroleum ether/DCM/EtOH/$NH_3·H_2O$ = 3:7:0.1:0.05). HRMS (ESI) $(M + H)^+$ *m/z* 900.5225, calcd for $C_{48}H_{73}N_3O_{13}$ 900.5216. ^1H NMR ($CDCl_3$, 400 MHz) δ: 8.03 (d, *J* = 7.6 Hz, 2 H, 2 H-Bz), 7.57 (t, *J* = 7.6 Hz, 1 H, 1 H-Bz), 7.45 (t, *J* = 7.6 Hz, 2 H, 2 H-Bz), 5.95 (d, *J* = 8.0 Hz, 1 H, 8a-CO-NH-), 5.11 (d, *J* = 9.6 Hz, 1 H, H-3), 5.04-4.97 (m, 2 H, H-2′, H-13), 4.82 (t, *J* = 9.6 Hz, 1 H, 3-*O*-CO-NH-), 4.46 (d, *J* = 8.8 Hz, 1 H, H-11), 4.34 (d, *J* = 7.2 Hz, 1 H, H-1′), 4.02-3.95 (m, 1 H, H-8), 3.71 (d, *J* = 5.2 Hz, 1 H, H-5), 3.50-3.46 (m, 1 H, H-5′), 3.32-3.10 (m, 5 H, 6-*O*-CH_3, -CONH-C**H₂**-), 2.89-2.83 (m, 1 H, H-3′), 2.71-2.63 (m, 1 H, H-2), 2.28 (s, 6 H, -N(CH_3)$_2$), 2.23-2.15 (m, 3 H, H-10, -C**H₂**-C≡CH), 1.94 (t, *J* = 2.4 Hz, 1H, -C≡CH), 1.80-1.72 (m, 4 H, H-4, H-14eq, H-7a, H-4′a), 1.67-1.38 (m, 13 H, H-4′b, H-14ax, H-7b, 5(CH_2)), 1.29-1.25 (m, 9 H, 5′-CH_3, 6-CH_3, 12-CH_3), 1.21 (d, *J* = 6.8 Hz, 3 H, 8-CH_3), 1.13-1.11 (m, 6 H, 10-CH_3, 2-CH_3), 0.87 (t, *J* = 7.2 Hz, 3 H, 15-CH_3), 0.74 (d, *J* = 7.6 Hz, 3 H, 4-CH_3).

2′-O-benzoyl-3-O-descladinosyl-3-O-(N-9-decynylcarbamoyl)-8a-azahomoerythromycin 11,12- cyclic carbonate ***(122)***

Following the general procedure H, from compound **119** (1.00 g, 1.21 mmol) and **31** (0.277 g, 1.45 mmol), **122** (0.501 g, 0.550 mmol, 45.5 %) was obtained as an off-white solid (column chromatography eluents: petroleum

ether/DCM/EtOH/$NH_3 \cdot H_2O$ = 3:7:0.1:0.05). HRMS (ESI) $(M + H)^+$ *m/z* 914.5397, calcd for $C_{49}H_{75}N_3O_{13}$ 914.5373. ^1H NMR ($CDCl_3$, 400 MHz) δ: 8.03 (d, *J* = 7.2 Hz, 2 H, 2 H-Bz), 7.57 (t, *J* = 7.6 Hz, 1 H, 1 H-Bz), 7.45 (t, *J* = 7.6 Hz, 2 H, 2 H-Bz), 6.00 (d, *J* = 8.0 Hz, 1 H, 8a-CO-NH-), 5.11 (d, *J* = 10.0 Hz, 1 H, H-3), 5.04-4.97 (m, 2 H, H-2′, H-13), 4.84 (t, *J* = 9.6 Hz, 1 H, 3-*O*-CO-NH-), 4.46 (d, *J* = 8.4 Hz, 1 H, H-11), 4.42 (d, *J* = 7.2 Hz, 1 H, H-1′), 4.05-3.95 (m, 1 H, H-8), 3.70 (d, *J* = 5.6 Hz, 1 H, H-5), 3.55-3.46 (m, 1 H, H-5′), 3.27-3.16 (m, 5 H, 6-*O*-CH_3, -CONH-C**H₂**-), 2.89-2.80 (m, 1 H, H-3′), 2.70-2.61 (m, 1 H, H-2), 2.28 (s, 6 H, -N(CH_3)$_2$), 2.20-2.16 (m, 3 H, H-10, -C**H₂**-C≡CH), 1.94 (t, *J* = 2.8 Hz, 1 H, -C≡CH), 1.92-1.72 (m, 4 H, H-4, H-14eq, H-7a, H-4′a), 1.55-1.38 (m, 15 H, 6(CH_2), H-4′b, H-14ax, H-7b), 1.28-1.26 (m, 9 H, 5′-CH_3, 6-CH_3, 12-CH_3), 1.20 (d, *J* = 6.8 Hz, 3 H, 8-CH_3), 1.13-1.11 (m, 6 H, 10-CH_3, 2-CH_3), 0.87 (t, *J* = 7.9 Hz, 3 H, 15-CH_3), 0.74 (d, *J* = 7.2 Hz, 3 H, 4-CH_3).

2′-O-benzoyl-3-O-descladinosyl-3-O-(N-7-octynylcarbamoyl)-8a-azahomoerythromycin ***(123)***

Following the general procedure N, from compound **120** (0.284 g, 0.320 mmol), **123** (0.140 g, 0.170 mmol, 53.1%) was obtained as an off-white solid (column chromatography eluents: petroleum ether/DCM/EtOH/$NH_3 \cdot H_2O$ = 3:7:0.1:0.05). HRMS (ESI) $(M + H)^+$ *m/z* 860.5281, calcd for $C_{46}H_{73}N_3O_{12}$ 860.5267. ^1H NMR ($CDCl_3$, 400 MHz) δ: 8.04 (d, *J* = 7.6 Hz, 2 H, 2 H-Bz), 7.57 (t, *J* = 7.6 Hz, 1 H, 1 H-Bz), 7.45 (t, *J* = 7.6 Hz, 2 H, 2 H-Bz), 6.16 (d, *J* = 8.0 Hz, 1 H, 8a-CO-NH-), 5.15 (d, *J* = 10.4 Hz, 1 H, H-3), 5.08-4.94 (m, 2 H, H-2′, H-13), 4.84 (t, *J* = 6.0 Hz, 1 H, 3-*O*-CO-NH-), 4.37 (d, *J* = 7.6 Hz, 1 H, H-1′), 4.31 (s, 1 H, 11-OH), 3.84-3.78 (m, 1 H, H-8), 3.67 (d, *J* = 5.2 Hz, 1 H, H-5), 3.51-3.46 (m, 1 H, H-5′), 3.37-3.27 (m, 2 H, H-11, -CONH-C**H₂**-), 3.20 (s, 3 H, 6-*O*-CH_3), 3.22-3.17 (m, 1 H, -CONH-C**H₂**-), 2.92-2.85 (m, 1 H, H-3′), 2.79 (s, 1 H, 12-OH), 2.72-2.66 (m, 1 H, H-2), 2.29 (s, 6 H, -N(CH_3)$_2$), 2.22-2.15 (m, 3 H, H-10, -C**H₂**-C≡CH), 1.98 (t, *J* = 2.8 Hz, 1H, -C≡CH), 1.89-1.76 (m, 4 H, H-4, H-14eq, H-7a, H-4′a), 1.58-1.36 (m, 11 H, H-4′b, H-14ax, H-7b, 4(CH_2)), 1.28-1.22 (m, 9 H, 6-CH_3, 12-CH_3, 5′-CH_3), 1.17 (d, *J* = 6.8 Hz, 3 H, 8-CH_3), 1.13 (d, *J* = 7.2 Hz, 3 H, 2-CH_3), 0.81-0.76 (m, 9 H, 10-CH_3, 15-CH_3, 4-CH_3)

2′-O-benzoyl-3-O-descladinosyl-3-O-(N-8-nonynylcarbamoy)-8a-azahomoerythromycin ***(124)***

Following the general procedure N, from compound **121** (1.24 g, 1.38 mmol), **124** (0.600 g, 0.700 mmol, 50.7%) was obtained as an off-white solid (column chromatography eluents: petroleum ether/DCM/EtOH/$NH_3 \cdot H_2O$ = 3:7:0.1:0.05).

2′-O-benzoyl-3-O-descladinosyl-3-O-(N-9-decynylcarbamoy)-8a-azahomoerythromycin ***(125)***

Following the general procedure N, from compound **122** (0.501 g, 0.550 mmol), **125** (0.300 g, 0.350 mmol, 63.6%) was obtained as an off-white solid (column chromatography eluents: petroleum ether/DCM/EtOH/$NH_3 \cdot H_2O$ = 3:7:0.1:0.05). HRMS (ESI) $(M + H)^+$ *m/z* 888.5597, calcd for $C_{48}H_{77}N_3O_{12}$ 888.5580. ^1H NMR ($CDCl_3$, 400 MHz) δ: 8.04 (d, *J* = 7.6 Hz, 2 H, 2 H-Bz), 7.56 (t, *J* = 7.6 Hz, 1 H, 1 H-Bz), 7.45 (t, *J* = 7.6 Hz, 2 H, 2 H-Bz), 6.16 (d, *J* = 8.0 Hz, 1 H, 8a-CO-NH-), 5.15 (d, *J* = 10.8 Hz, 1 H, H-3), 5.04-4.94 (m, 2 H, H-2′, H-13), 4.83 (t, *J* = 5.6 Hz, 1 H, 3-*O*-CO-NH-), 4.37 (d, *J* = 6.8 Hz, 1 H, H-1′), 4.31 (s, 1 H, 11-OH), 3.84-3.75 (m, 1 H, H-8), 3.68 (d, *J* = 5.2 Hz, 1 H, H-5), 3.51-3.46 (m, 1 H, H-5′), 3.31-3.25 (m, 2 H, H-11, -CONH-C**H₂**-), 3.20 (s, 3 H, 6-*O*-CH_3), 3.18-3.10 (m, 1 H, -CONH-C**H₂**-), 2.90-2.82 (m, 1 H, H-3′), 2.79 (s, 1 H, 12-OH), 2.72-2.66 (m, 1 H, H-2), 2.28 (s, 6 H, -N(CH_3)$_2$), 2.19-2.15 (m, 3 H, H-10, -C**H₂**-C≡CH), 1.94 (t, *J* = 2.4 Hz, 1 H, -C≡CH), 1.88-1.76 (m, 4 H, H-4, H-14eq, H-7a, H-4′a), 1.58-1.36 (m, 15 H, H-4′b, H-14ax, H-7b, 6(CH_2)), 1.28-1.24 (m, 9 H, 6-CH_3, 12-CH_3, 5′-CH_3), 1.17-1.12 (m, 6 H, 2-CH_3, 8-CH_3), 0.83-0.75 (m, 9 H, 10-CH_3, 15-CH_3, 4-CH_3)

3-O-descladinosyl-3-O-[N-8-(1-cyclopropyl-1,4-dihydro-4-oxo-3-quinolinecarboxylic acid-7-yl)-oct-7-ynylcarbamoyl]-8a-azahomoerythromycin ***(126c)***

Following the general procedure I-1, from compound **123** (0.300 g, 0.350 mmol) and **4** (0.149 g, 0.420 mmol), **126c** (48.0 mg, 0.0500 mmol, 13.9%) was obtained as an off-white solid (column chromatography eluents: DCM/MeOH/$NH_3 \cdot H_2O$ = 10:1.2:0.5). m.p. 147.4-148.9 ˚C. HRMS (ESI) $(M + H)^+$ *m/z* 983.5595, calcd for $C_{52}H_{78}N_4O_{14}$ 983.5587. ^1H NMR ($CDCl_3$, 400 MHz) δ: 8.85 (s, 1 H, 2-quinolyl), 8.39 (d, *J* = 8.4 Hz, 1 H, 5-quinolyl), 8.06 (s, 1 H, 8-quinolyl), 7.54 (d, *J* = 8.4 Hz, 1 H, 6-quinolyl), 5.98 (d, *J* = 7.6 Hz, 1 H, 8a-CO-NH-), 5.16 (d, *J* = 10.4 Hz, 1 H, H-3), 5.01 (dd, *J* = 10.4, 2.0 Hz, 1 H, H-13), 4.06 (d, *J* = 6.4 Hz, 1 H, H-1′), 3.94 (s, 1 H, 1H-cyclopropyl), 3.81 (s, 1 H, 11-OH), 3.73-3.67 (m, 1 H, H-8), 3.62-3.58 (m, 1 H, H-5′), 3.40-3.32 (m, 3 H, H-11, H-5, -CONH-

C**H**$_2$-), 3.23-3.21 (m, 4 H, -CONH-C**H**$_2$-, 6-*O*-CH_3), 3.09-3.03 (m, 1 H, H-3′), 2.82-2.78 (m, 2 H, H-2), 2.51-2.41 (m, 3 H, H-10, -C**H**$_2$-C≡C-Ar), 2.32 (s, 6 H, -N(CH_3)$_2$), 2.13-2.11 (m, 1 H, H-4), 1.95-1.90 (m, 2 H, H-14eq, H-7a), 1.68-1.41 (m, 12 H, H-4′a, H-4′b, H-14ax, H-7b, 4(CH_2)), 1.30-1.25 (m, 8 H, 2H-cyclopropyl, 6-CH_3, 5′-CH_3), 1.22-1.17 (m, 11 H, 2H-cyclopropyl, 10-CH_3, 8-CH_3, 2-CH_3), 1.12 (s, 3 H, 12-CH_3), 1.07 (d, *J* = 7.2 Hz, 3 H, 4-CH_3), 0.87 (t, *J* = 7.2 Hz, 3 H, 15-CH_3). ^{13}C NMR ($CDCl_3$, 100 MHz) δ: 178.2, 175.8, 175.2, 166.8, 156.6, 148.5, 141.0, 130.1, 129.4, 126.8, 124.8, 119.8, 108.9, 103.3, 95.9, 82.5, 79.8, 78.4, 78.0, 75.2, 70.9, 69.3, 65.8, 50.9, 43.3, 43.3, 43.2, 42.0, 41.1, 40.4, 36.1, 30.0, 28.5, 28.3, 28.3, 26.3, 22.9, 21.4, 21.2, 20.6, 19.5, 16.5, 15.2, 10.9, 10.5, 9.3.

*3-O-descladinosyl-3-O-[N-8-(1,4-dihydro-1-methyl-4-oxo-3-quinolinecarboxylic acid-7-yl)-oct-7-ynylcarbamoyl]-8a-azahomoerythromycin **(126g)***

Following the general procedure I-1, from compound **123** (0.300 g, 0.350 mmol) and **11** (0.138 g, 0.420 mmol), **126g** (52.0 mg, 0.0500 mmol, 14.3%) was obtained as an off-white solid (column chromatography eluents: DCM/MeOH/$NH_3{\cdot}H_2O$ = 10:1.2:0.5). m.p. 169.9-172.3 °C. HRMS (ESI) (M + H)$^+$ *m/z* 957.5436, calcd for $C_{51}H_{78}N_4O_{14}$ 957.5431. ^{1}H NMR ($CDCl_3$, 400 MHz) δ: 8.75 (s, 1 H, 2-quinolyl), 8.43 (d, *J* = 8.4 Hz, 1 H, 5-quinolyl), 7.60 (s, 1 H, 8-quinolyl), 7.55 (d, *J* = 8.4 Hz, 1 H, 6-quinolyl), 5.92 (d, *J* = 7.6 Hz, 1 H, 3-*O*-CONH-), 5.15 (d, *J* = 10.8 Hz, 1 H, H-3), 5.01 (dd, *J* = 10.6, 2.0 Hz, 1 H, H-13), 4.05 (d, *J* = 6.4 Hz, 1 H, H-1′), 4.00 (s, 3 H, N-CH_3), 3.87 (s, 1 H, 11-OH), 3.72-3.67 (m, 1 H, H-8), 3.40-3.32 (m, 3 H, H-11, H-5, -CONH-C**H**$_2$-), 3.22-3.20 (m, 4 H, -CONH-C**H**$_2$-, 6-*O*-CH_3), 3.09-3.04 (m, 1 H, H-3′), 2.82-2.77 (m, 1 H, H-2), 2.50-2.40 (m, 3 H, H-10, -C**H**$_2$-C≡C-Ar), 2.30 (s, 6 H, -N(CH_3)$_2$), 2.12-2.10 (m, 1 H, H-4), 1.96-1.90 (m, 2 H, H-14eq, H-7a), 1.65-1.38 (m, 12 H, H-4′a, H-4′b, H-14ax, H-7b, 4(CH_2)), 1.30-1.24 (m, 6 H, 6-CH_3, 5′-CH_3), 1.21-1.16 (m, 9 H, 10-CH_3, 8-CH_3, 2-CH_3), 1.11-1.06 (m, 6 H, 4-CH_3, 12-CH_3), 0.86 (t, *J* = 7.2 Hz, 3 H, 15-CH_3). ^{13}C NMR ($CDCl_3$, 100 MHz) δ: 178.1, 175.8, 175.2, 166.8, 156.6, 149.5, 140.2, 130.4, 129.4, 127.0, 125.1, 119.1, 109.1, 96.0, 79.7, 78.5, 78.1, 75.3, 70.9, 70.7, 70.7, 69.2, 65.7, 50.9, 43.2, 41.9, 41.0, 40.3, 36.1, 35.4, 30.1, 29.7, 29.1, 28.6, 28.3, 26.3, 22.9, 21.4, 21.2, 20.6, 19.5, 16.5, 15.6, 10.9, 10.5, 9.4, 8.3.

*3-O-descladinosyl-3-O-[N-8-(1,4-dihydro-4-oxo-3-quinolinecarboxylic acid-7-yl)-oct-7-ynylcarbamoyl]-8a-azahomoerythromycin **(126i)***

Following the general procedure I-1, from compound **123** (0.200 g, 0.240 mmol) and **12** (0.0890 g, 0.290 mmol), **126i** (50.0 mg, 0.0500 mmol, 20.8%) was obtained as an off-white solid (column chromatography eluents: DCM/MeOH/$NH_3{\cdot}H_2O$ = 10:1.2:0.5). m.p. 174.2-176.0 °C. HRMS (ESI) (M + H)$^+$ *m/z* 943.5277, calcd for $C_{49}H_{74}N_4O_{14}$ 943.5274. ^{1}H NMR ($CDCl_3$, 400 MHz) δ: 8.92 (s, 1 H, 2-quinolyl), 8.23 (d, *J* = 8.4 Hz, 1 H, 5-quinolyl), 7.75 (s, 1 H, 8-quinolyl), 7.75 (d, *J* = 8.4 Hz, 1 H, 6-quinolyl), 6.28 (d, *J* = 6.0 Hz, 1 H, 8a-CO-NH-), 5.80 (s, 1 H, 3-*O*-CO-NH-), 5.14 (d, *J* = 10.4 Hz, 1 H, H-3), 4.96 (dd, *J* = 10.4, 2.0 Hz, 1 H, H-13), 4.14 (d, *J* = 6.4 Hz, 1 H, H-1′), 3.98-3.94 (m, 1 H, 1-NH), 3.84 (s, 1 H, 11-OH), 3.71-3.64 (m, 1 H, H-8), 3.49-3.25 (m, 4 H, H-5, H-11, -CONH-C**H**$_2$-), 3.21 (s, 3 H, 6-*O*-CH_3), 3.18-3.11 (m, 1 H, H-5′), 2.74 (m, 2 H, H-2, H-3′), 2.49-2.44 (m, 3 H, H-10, -C**H**$_2$-C≡C-Ar), 2.39 (s, 6 H, -N(CH_3)$_2$), 2.14-2.10 (m, 1 H, H-4), 1.93-1.88 (m, 2 H, H-14eq, H-7a), 1.75-1.68 (m, 1 H, H-4′a), 1.67-1.38 (m, 11 H, H-4′b, H-14ax, H-7b, 4(CH_2)), 1.29 (d, *J* = 7.2 Hz, 3 H, 5′-CH_3), 1.26 (s, 3 H, 6-CH_3), 1.22-1.15 (m, 9 H, 10-CH_3, 8-CH_3, 2-CH_3), 1.08 (s, 3 H, 12-CH_3).1.01 (d, *J* = 5.6 Hz, 3 H, 4-CH_3), 0.85 (t, *J* = 7.2 Hz, 3 H, 15-CH_3). ^{13}C NMR ($CDCl_3$, 100 MHz) δ: 177.1, 176.4, 175.6, 169.6, 156.8, 146.4, 141.1, 129.2, 128.7, 125.3, 123.5, 108.6, 102.5, 94.9, 81.2, 79.9, 78.3, 75.4, 71.2, 70.6, 68.9, 65.7, 51.1, 43.3, 43.1, 41.7, 41.1, 40.2, 36.1, 29.9, 29.6, 29.3, 28.3, 27.9, 16.2, 22.8, 21.4, 21.1, 20.5, 19.3, 16.6, 10.9, 10.7, 9.3.

*3-O-descladinosyl-3-O-[N-8-(3-quinolinecarboxylic acid-7-yl)-oct-7-ynylcarbamoyl]-8a-azahomoerythromycin **(126j)***

Following the general procedure I-2, from compound **123** (0.354 g, 0.410 mmol) and methyl 7-bromoquinoline-3-carboxylate (0.131 g, 0.490 mmol), methyl ester-**126j** was obtained. After completion of this reaction, methyl ester-**126j** was treated with $LiOH{\cdot}H_2O$ to remove the protective group, and **126j** (50.0 mg，0.0500 mmol，13.2 %) was obtained as an off-white solid (column chromatography eluents: DCM/MeOH/$NH_3{\cdot}H_2O$ = 10:1:0.5). m.p. 165.2-166.1 °C. HRMS (ESI) (M + H)$^+$ *m/z* 927.5329, calcd for $C_{51}H_{78}N_4O_{14}$ 927.5325. ^{1}H NMR ($CDCl_3$, 400 MHz) δ: 9.50 (d, 1 H, *J* = 1.2 Hz, 2-quinolyl), 8.73 (s, 1 H, 4-quinolyl), 8.10 (s, 1 H, 8-quinolyl), 7.42 (d, *J* = 4.4 Hz, 1 H, 5-

quinolyl), 7.77 (d, J = 4.4 Hz, 1 H, 6-quinolyl), 6.98 (s, 1 H, 3-*O*-CO-NH-), 5.90 (d, J = 7.2 Hz, 1 H, 8a-CO-NH-), 5.20 (d, J = 6.8 Hz, 1 H, H-3), 5.02 (dd, J = 10.7, 2.0 Hz, 1 H, H-13), 4.23 (d, J =7.2 Hz, 1 H, H-1′), 3.77 (s, 1 H, 11-OH), 3.74-3.68 (m, 1 H, H-8), 3.51-3.47 (m, 1 H, H-5′, -CONH-C**H**$_2$-), 3.41-3.34 (m, 3 H, H-5, H-11, -CONH-C**H**$_2$-), 3.21 (s, 3 H, 6-*O*-CH_3), 2.90-2.86 (m, 2 H, H-2, H-3′), 2.78 (s, 6 H, -N(CH_3)$_2$), 2.44 (q, 1 H, J = 10.2 Hz, 4.0 Hz, H-10), 2.27-2.05 (m, 3 H, H-4, H-7a, -C**H**$_2$-C≡C-Ar), 1.95-1.86 (m, 3 H, H-4′a, H-14eq, -C**H**$_2$-C≡C-Ar), 1.57-1.24 (m, 17 H, H-4′b, H-14ax, H-7b, 4(CH_2), 5′-CH_3, 6-CH_3), 1.21-1.16 (m, 9 H, 10-CH_3, 8-CH_3, 2-CH_3), 1.16 (d, J = 7.2 Hz, 3 H, 4-CH_3), 1.12 (s, 3 H, 12-CH_3), 0.89 (t, J = 7.2 Hz, 3 H, 15-CH_3). ^{13}C NMR ($CDCl_3$, 100 MHz) δ: 176.2, 175.2, 172.2, 157.0, 152.1, 148.7, 137.3, 131.8, 129.9, 128.6, 128.2, 126.6, 126.5, 103.4, 93.2, 84.5, 80.3, 78.2, 75.3, 70.9, 70.8, 68.3, 64.4, 50.9, 43.3, 43.2, 42.2, 41.2, 38.9, 35.9, 30.4, 29.9, 28.9, 28.5, 26.5, 22.9, 21.4, 21.1, 19.5, 16.5, 15.7, 10.9, 10.6, 9.5.

3-O-descladinosyl-3-O-(N-9-(1,4-dihydro-4-oxo-3-quinolinecarboxylic acid-7-yl)-non-8-ynylcarbamoy)-8a-azahomoerythromycin ***(127i)***

Following the general procedure I-1, from compound **124** (0.300 g, 0.340 mmol) and **12** (0.129 g, 0.400 mmol), **127i** (45.0 mg, 0.0500 mmol, 13.8%) was obtained as an off-white solid (column chromatography eluents: DCM/MeOH/NH_3·H_2O = 10:1.2:0.5). m.p. 171.7-173.2 ˚C. HRMS (ESI) $(M + H)^+$ *m/z* 957.5437, calcd for $C_{50}H_{76}N_4O_{14}$ 957.5431. ^{1}H NMR ($CDCl_3$, 400 MHz) δ: 8.90 (s, 1 H, 2-quinolyl), 8.21 (d, J = 8.4 Hz, 1 H, 5-quinolyl), 7.69 (s, 1 H, 8-quinolyl), 7.39 (d, J = 8.4 Hz, 1 H, 6-quinolyl), 6.27 (d, J = 6.0 Hz, 1 H, 8a-CO-NH-), 5.75 (s, 1 H, 3-*O*-CO-NH-), 5.12 (d, J = 10.4 Hz, 1 H, H-3), 4.92 (dd, J = 10.4, 2.0 Hz, 1 H, H-13), 4.15 (d, J = 6.4 Hz, 1 H, H-1′), 4.04-4.00 (m, 1 H, 1-NH), 3.84 (s, 1 H, 11-OH), 3.70-3.65 (m, 1 H, H-8), 3.44-3.26 (m, 4 H, H-5, H-11, -CONH-C**H**$_2$-), 3.21 (s, 3 H, 6-*O*-CH_3), 3.13-3.08 (m, 1 H, H-5′), 2.76-2.63 (m, 2 H, H-2, H-3′), 2.48-2.40 (m, 9 H, H-10, -C**H**$_2$-C≡C-Ar, -N(CH_3)$_2$), 2.11-2.09 (m, 1 H, H-4), 1.96-1.87 (m, 2 H, H-14eq, H-7a), 1.73-1.71 (m, 1 H, H-4′a), 1.60-1.39 (m, 13 H, H-4′b, H-14ax, H-7b, 5(CH_2)), 1.30 (d, J = 6.8 Hz, 3 H, 5′-CH_3), 1.26 (s, 3 H, 6-CH_3), 1.23-1.20 (m, 6 H, 10-CH_3, 8-CH_3), 1.12 (d, J = 6.8 Hz, 3 H, 2-CH_3), 1.08 (s, 3 H, 12-CH_3), 0.99 (d, J = 6.8 Hz, 3 H, 4-CH_3), 0.84 (t, J = 7.2 Hz, 3 H, 15-CH_3). ^{13}C NMR ($CDCl_3$, 100 MHz) δ: 177.2, 176.3, 175.7, 169.5, 156.7, 146.3, 140.8, 129.4, 128.7, 125.4, 123.5, 108.6, 102.6, 95.2, 81.2, 79.9, 78.4, 77.6, 75.4, 71.1, 70.7, 69.0, 65.6, 51.1, 43.4, 43.2, 43.1, 41.7, 41.1, 40.2, 36.0, 29.8, 29.1, 28.6, 28.5, 27.9, 26.7, 22.8, 21.3, 21.1, 20.6, 19.4, 16.5, 15.7, 10.9, 9.3.

3-O-descladinosyl-3-O-(N-10-(1,4-dihydro-4-oxo-3-quinolinecarboxylic acid-7-yl)-dec-9-ynylcarbamoy)-8a-azahomoerythromycin ***(128i)***

Following the general procedure I-1, from compound **125** (0.295 g, 0.330 mmol) and **12** (0.126 g, 0.390 mmol), **128i** (20.0 mg, 0.0200 mmol, 6.20 %) was obtained as an off-white solid (column chromatography eluents: DCM/MeOH/NH_3·H_2O = 10:1.2:0.5). m.p. 172.6-174.1 ˚C. HRMS (ESI) $(M + H)^+$ *m/z* 971.5599, calcd for $C_{51}H_{78}N_4O_{14}$ 971.5587. ^{1}H NMR ($CDCl_3$, 400 MHz) δ: 8.92 (s, 1 H, 2-quinolyl), 8.27 (d, J = 7.6 Hz, 1 H, 5-quinolyl), 7.76 (s, 1 H, 8-quinolyl), 7.42 (d, J = 8.4 Hz, 1 H, 6-quinolyl), 6.23 (d, J = 6.0 Hz, 1 H, 8a-CO-NH-), 5.67 (s, 1 H, 3-*O*-CO-NH-), 5.13 (d, J = 10.4 Hz, 1 H, H-3), 4.93 (dd, J = 10.4 Hz, 1 H, H-13), 4.11 (d, J = 6.4 Hz, 1 H, H-1′), 4.04-4.00 (m, 1 H, 1-NH), 3.83 (s, 1 H, 11-OH), 3.70-3.68 (m, 1 H, H-8), 3.44-3.25 (m, 4 H, H-5, H-11, -CONH-C**H**$_2$-), 3.20 (s, 3 H, 6-*O*-CH_3), 3.08-3.06 (m, 1 H, H-5′), 2.71 (m, 2 H, H-2, H-3′), 2.49-2.40 (m, 9 H, H-10, -C**H**$_2$-C≡C-Ar, -N(CH_3)$_2$), 2.11-2.09 (m, 1 H, H-4), 1.93-1.89 (m, 2 H, H-14eq, H-7a), 1.72-1.70 (m, 1 H, H-4′a), 1.59-1.34 (m, 15 H, H-4′b, H-14ax, H-7b, 6(CH_2)), 1.29 (d, J = 6.8 Hz, 3 H, 5′-CH_3), 1.26 (s, 3 H, 6-CH_3), 1.22-1.21 (m, 6 H, 10-CH_3, 8-CH_3), 1.15 (d, J = 6.4 Hz, 3 H, 2-CH_3), 1.08 (s, 3 H, 12-CH_3), 0.99 (d, J = 6.4 Hz, 3 H, 4-CH_3), 0.86 (t, J = 7.2 Hz, 3 H, 15-CH_3). ^{13}C NMR ($CDCl_3$, 100 MHz) δ: 176.3, 175.6, 169.6, 156.7, 129.4, 128.7, 125.3, 123.5, 108.6, 102.8, 95.2, 81.4, 79.8, 78.4, 77.6, 75.4, 71.2, 70.6, 69.0, 65.7, 51.1, 43.4, 43.1, 43.1, 41.8, 41.1, 40.2, 36.1, 30.0, 29.7, 29.2, 28.9, 28.5, 28.1, 26.5, 22.8, 21.4, 21.1, 20.6, 19.5, 15.7, 10.9, 10.7, 9.3.

3-O-descladinosyl-3-O-(N-8-(1,4-dihydro-1-methyl-4-oxo-3-quinolinecarboxylic acid-7-yl)-octylcarbamoyl)-8a-azahomoerythromycin ***(129g)***

Following the general procedure E, from compound **126g** (0.100 g, 0.100 mmol), **129g** (43.5 mg, 0.0500 mmol, 20.0%) was obtained as an off-white solid (column chromatography eluents: DCM/MeOH/NH_3·H_2O = 10:1.2:0.5).

m.p. 139.3-140.0 °C. HRMS (ESI) $(M + H)^+$ *m/z* 961.5757, calcd for $C_{50}H_{81}N_4O_{14}$ 961.5744. ^1H NMR ($CDCl_3$, 400 MHz) δ: 8.73 (s, 1 H, 2-quinolyl), 8.44 (d, *J* = 8.4 Hz, 1 H, 5-quinolyl), 7.43 (d, *J* = 8.4 Hz, 1 H, 6-quinolyl), 7.35 (s, 1 H, 8-quinolyl), 5.89 (d, *J* = 7.6 Hz, 1 H, 3-*O*-CO-NH-), 5.16 (d, *J* = 10.8 Hz, 1 H, H-3), 5.01 (d, *J* = 9.6 Hz, 1 H, H-13), 4.02 (s, 3 H, N-CH_3), 3.92 (s, 1 H, H-1′), 3.81 (s, 1 H, 11-OH), 3.72-3.67 (m, 1 H, H-8), 3.41-3.28 (m, 3 H, H-5, -CONH-C**H₂**-), 3.21 (s, 4 H, H-11, 6-*O*-CH_3), 3.06-3.01 (m, 1 H, H-5′), 2.84-2.80 (m, 3 H, H-2, H-3′, -C**H**$_2$-Ar), 2.44-2.40 (m, 2 H, H-10, -C**H**$_2$-Ar), 2.29 (s, 6 H, -N(CH_3)$_2$), 2.14-2.11 (m, 1 H, H-4), 1.97-1.92 (m, 2 H, H-14eq, H-7a), 1.72-1.60 (m, 3 H, H-4′a, H-14ax, H-7b), 1.56-1.46 (m, 4 H, 2(CH_2)), 1.34-1.26 (m, 11 H, H-4′b, 5′-CH_3, 6-CH_3, -CH_2CH_2C**H**$_2$C**H**$_2$$CH_2CH_2$-Ar), 1.21-1.18 (m, 9 H, 10-CH_3, 8-CH_3, 2-CH_3), 1.17 (s, 3 H, 12-CH_3), 1.08 (d, *J* = 7.2 Hz, 3 H, 4-CH_3), 0.87 (t, *J* = 7.2 Hz, 3 H, 15-CH_3). ^{13}C NMR ($CDCl_3$, 100MHz) δ: 178.4, 175.1, 167.1, 150.4, 149.0, 140.5, 127.3, 127.1, 124.4, 115.5, 108.7, 78.5, 75.3, 70.7, 69.3, 51.0, 43.4, 43.3, 43.2, 42.0, 41.1, 40.4, 36.6, 31.2, 30.2, 29.7, 29.3, 29.2, 29.1, 22.9, 21.5, 20.6, 16.5, 15.6, 10.9, 10.5, 9.4.

3-O-descladinosyl-3-O-[N-8-(1,4-dihydro-4-oxo-3-quinolinecarboxylic acid-7-yl)-octylcarbamoyl]-8a-azahomoerythromycin ***(129i)***

Following the general procedure E, from compound **126i** (0.180 g, 0.190 mmol), **129i** (32.0 mg, 0.0300 mmol, 17.8%) was obtained as an off-white solid (column chromatography eluents: DCM/MeOH/$NH_3 \cdot H_2O$ = 10:1.2:0.5). m.p. 165.7-168.1 °C. HRMS (ESI) $(M + H)^+$ *m/z* 947.5591, calcd for $C_{49}H_{79}N_4O_{14}$ 947.5587. ^1H NMR ($CDCl_3$, 400 MHz) δ: 8.90 (s, 1 H, 2-quinolyl), 8.27 (d, *J* = 7.2 Hz, 1 H, 5-quinolyl), 7.58 (s, 1 H, 8-quinolyl), 7.31 (d, *J* = 7.6 Hz, 1 H, 6-quinolyl), 6.27 (d, *J* = 5.6 Hz, 1 H, 8a-CO-NH-), 5.62 (s, 1 H, 3-*O*-CO-NH-), 5.15 (d, *J* = 10.4 Hz, 1 H, H-3), 4.96 (dd, *J* = 10.5 Hz, 1 H, H-13), 4.12 (d, *J* = 5.6 Hz, 1 H, H-1′), 4.04-4.01 (m, 1 H, 1-NH), 3.84 (s, 1 H, 11-OH), 3.71-3.69 (m, 1 H, H-8), 3.40-3.26 (m, 4 H, H-5, H-11, -CONH-C**H₂**-), 3.20 (s, 3 H, 6-*O*-CH_3), 3.09-3.02 (m, 1 H, H-5′), 2.79-2.63 (m, 4 H, H-2, H-3′, -C**H₂**-Ar), 2.50-2.48 (m, 1 H, H-10), 2.38 (s, 6 H, -N(CH_3)$_2$), 2.13-2.11 (m, 1 H, H-4), 1.97-1.86 (m, 2 H, H-14eq, H-7a), 1.72-1.64 (m, 3 H, H-4′a, H-14ax, H-7b), 1.51-1.45 (m, 5 H, H-4′b, 2(CH_2)), 1.29-1.27 (m, 14 H, -CONH-CH_2CH_2(C**H₂**)$_4$$CH_2CH_2$-, 5′-$CH_3$, 6-$CH_3$), 1.21-1.15 (m, 9 H, 10-CH_3, 8-CH_3, 2-CH_3), 1.09 (s, 3 H, 12-CH_3), 1.04 (d, *J* = 7.4 Hz, 3 H, 4-CH_3), 0.85 (t, *J* = 6.4 Hz, 3 H, 15-CH_3). ^{13}C NMR ($CDCl_3$, 100MHz) δ: 178.2, 176.3, 175.6, 169.5, 156.7, 149.5, 145.2, 140.7, 127.3, 125.5, 122.9, 118.8, 108.0, 102.9, 81.6, 78.4, 77.6, 75.3, 71.1, 70.7, 69.1, 65.7, 51.1, 43.4, 43.2, 43.1, 41.8, 41.0, 40.3, 36.2, 35.8, 31.9, 30.4, 29.9, 29.7, 28.9, 28.7, 26.5, 22.8, 21.4, 21.2, 20.6, 16.5, 15.6, 14.1, 10.9, 10.7, 9.4

3-O-descladinosyl-3-O-[N-9-(1,4-dihydro-4-oxo-3-quinolinecarboxylic acid-7-yl)-nonylcarbamoy)]8a-azahomoerythromycin ***(130i)***

Following the general procedure E, from compound **127i** (0.200 g, 0.210 mmol), **130i** (20.0 mg, 0.0200 mmol, 9.9 %) was obtained as an off-white solid (column chromatography eluents: DCM/MeOH/$NH_3 \cdot H_2O$ = 10:1.2:0.5). m.p. 160.6-163.0 °C. HRMS (ESI) $(M + H)^+$ *m/z* 961.5764, calcd for $C_{50}H_{81}N_4O_{14}$ 961.5744. ^1H NMR ($CDCl_3$, 400 MHz) δ: 8.90 (s, 1 H, 2-quinolyl), 8.25 (d, *J* = 7.6 Hz, 1 H, 5-quinolyl), 7.57 (s, 1 H, 8-quinolyl), 7.31 (d, *J* = 6.8 Hz, 1 H, 6-quinolyl), 6.27 (d, *J* = 6.0 Hz, 1 H, 8a-CO-NH-), 5.62 (s, 1 H, 3-*O*-CO-NH-), 5.14 (d, *J* = 10.4 Hz, 1 H, H-3), 4.95 (dd, *J* = 10.4 Hz, 1 H, H-13), 4.13 (d, *J* = 5.2 Hz, 1 H, H-1′), 4.06-4.01 (m, 1 H, 1-NH), 3.84 (s, 1 H, 11-OH), 3.71-3.69 (m, 1 H, H-8), 3.43-3.26 (m, 4 H, H-5, H-11, -CONH-C**H₂**-), 3.20 (s, 3 H, 6-*O*-CH_3), 3.09-3.02 (m, 1 H, H-5′), 2.79-2.71 (m, 4 H, H-2, H-3′,-C**H₂**-Ar), 2.50-2.48 (m, 1 H, H-10), 2.39 (s, 6 H, -N(CH_3)$_2$), 2.13-2.11 (m, 1 H, H-4), 1.93-1.91 (m, 2 H, H-14eq, H-7a), 1.72-1.64 (m, 3 H, H-4′a, H-14ax, H-7b), 1.51-1.45 (m, 5 H, H-4′b, -CONH-CH_2C**H₂**(CH_2)$_5$C**H₂**CH_2-), 1.30-1.27 (m, 16 H, 5′-CH, 6-CH_3, -CONH-CH_2CH_2(C**H₂**)$_5$$CH_2CH_2$-), 1.23-1.15 (m, 9 H, 10-CH_3, 8-CH_3, 2-CH_3), 1.08 (s, 3 H, 12-CH_3), 1.03 (d, *J* = 7.0 Hz, 3 H, 4-CH_3), 0.85 (t, *J* = 6.4 Hz, 3 H, 15-CH_3). ^{13}C NMR ($CDCl_3$, 100MHz) δ: 178.2, 176.4, 175.6, 169.5, 156.7, 149.6, 140.6, 127.3, 125.5, 122.9, 118.8, 108.0, 102.8, 81.5, 78.4, 75.4, 71.2, 70.7, 69.0, 65.6, 51.1, 43.4, 43.2, 43.1, 41.8, 41.1, 40.2, 36.1, 35.9, 30.5, 29.9, 29.7, 29.4, 29.2, 29.1, 28.9, 28.9, 26.6, 22.8, 21.4, 21.2, 20.6, 16.6, 15.7, 10.9, 10.7, 9.4

3-O-descladinosyl-3-O-[N-8-(1,4-dihydro-4-oxo-3-quinolinecarboxylic acid-7-yl)-oct-7-ynylcarbamoyl]-8a-azahomoerythromycin-11,12-cyclic carbonate ***(131i)***

Following the general procedure I-1, from compound **120** (0.330 g, 0.37 mmol) and **12** (0.141 g, 4.47 mmol),

131i (102.8 mg, 0.110 mmol, 29.7%) was obtained as an off-white solid (column chromatography eluents: DCM/MeOH/$NH_3{\cdot}H_2O$ = 10:1.2:0.5). m.p. 181.6-183.1 ˚C. HRMS (ESI) $(M + H)^+$ *m/z* 969.5070, calcd for $C_{50}H_{72}N_4O_{15}$ 969.5067. ^{1}H NMR (CD_3OD-d_4, 400 MHz) δ: 8.88 (s, 1 H, 2-quinolyl), 8.25 (d, *J* = 8.0 Hz, 1 H, 5-quinolyl), 7.74 (s, 1 H, 8-quinolyl), 7.47 (d, *J* = 8.4 Hz, 1 H, 6-quinolyl), 5.09 (d, *J* = 10.0 Hz, 1 H, H-3), 5.03 (dd, *J* = 8.4, 2.8 Hz, 1 H, H-13), 4.50 (d, *J* = 4.4 Hz, 1 H, H-11), 4.19 (d, *J* = 6.8 Hz, 1 H, H-1′), 4.13-4.10 (m, 1 H, 1-NH), 3.92 (d, *J* = 2.8 Hz, 1 H, H-5), 3.69-3.61 (m, 4 H, H-5′, H-8, -CONH-C**H**$_2$-), 3.16 (s, 3 H, 6-*O*-CH_3), 2.75-2.65 (m, 11 H, -N$(CH_3)_2$, -C**H**$_2$-C≡C-Ar, H-10, H-2, H-3′), 2.03-2.02 (m, 1 H, H-4), 1.89-1.82 (m, 3 H, H-14eq, H-7a, H-4′a), 1.67-1.60 (m, 2 H, H-14ax, H-7b), 1.52 (d, *J* = 2.8 Hz, 1 H, H-4′b), 1.46-1.42 (m, 5 H , -C**H**$_2$-CH_2-C≡C-Ar, 12-CH_3), 1.25-1.21 (m, 11 H, -CONH-CH_2CH_2C**H**$_2$-, 8-CH_3, 6-CH_3, 5′-CH_3), 1.12-1.08 (m, 6 H, 2-CH_3, 10-CH_3), 1.03 (d, *J* = 7.2 Hz, 3 H, 4-CH_3), 0.90 (t, *J* = 7.2 Hz, 3 H, 15-CH_3). ^{13}C NMR (CD_3OD-d_4, 100 MHz) δ: 174.8, 170.9, 170.6, 169.0, 157.5, 153.7, 148.4, 144.0, 128.4, 128.1, 124.4, 122.9, 108.4, 101.4, 93.8, 86.4, 86.3, 82.2, 79.5, 78.9, 77.8, 77.2, 75.9, 69.4, 65.5, 50.0, 43.3, 42.3, 41.1, 40.5, 40.3, 38.9, 37.1, 30.1, 29.5, 28.3, 28.2, 26.0, 22.0, 21.9, 21.9, 19.9, 19.8, 19.7, 18.7, 14.5, 14.4, 11.4, 9.5, 8.6.

Synthesis of compounds 137 and 141-143

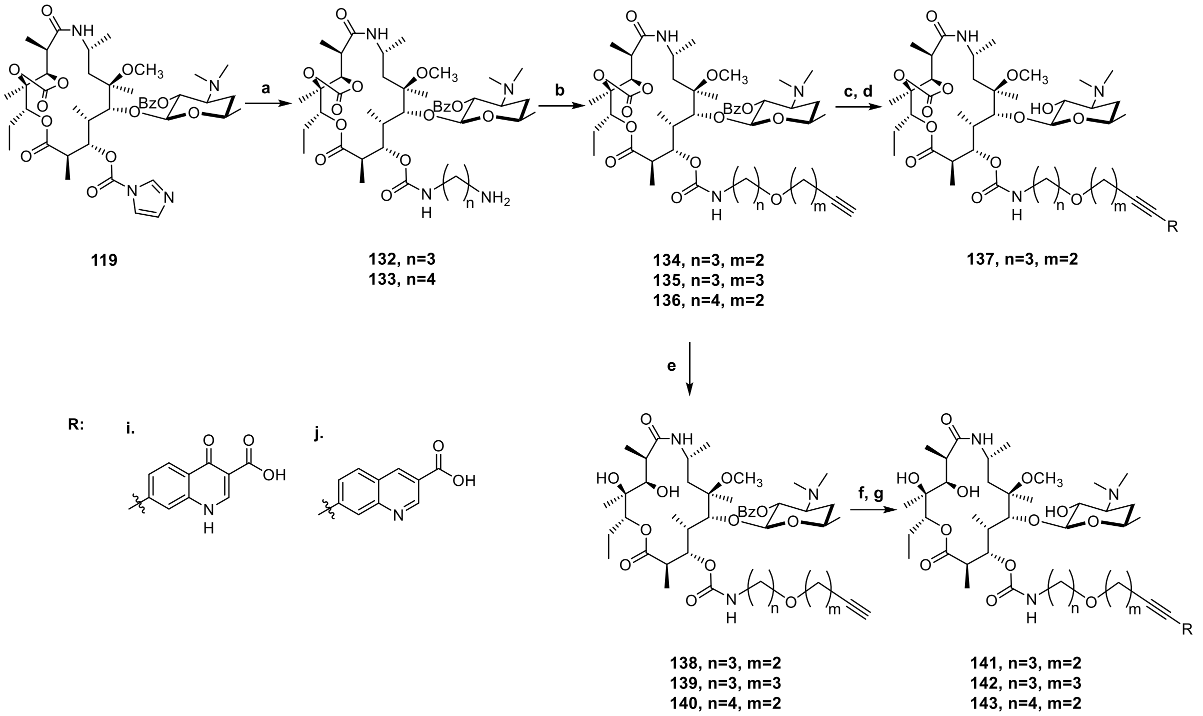

Scheme S15. Synthesis of *8a-azahomoerythromycin* series compounds **137** and **141-143**. Reagents and conditions: (a) 1,3-diaminopropane or 1,4-butanediamine, DBU, DMF, rt, 3 h; (b) 3-butyn-1-ol or 4-pentyn-1-ol, $NaNO_2$, HCOOH, -15˚C, 12-72 h; (c) R-I (R = **i**), CuI, $Pd(PPh_3)_2Cl_2$, Et_3N, CH_3CN, 45 ˚C, 12 h; (d) CH_3OH, 65 ˚C, 12 h; (e) $LiOH{\cdot}H_2O$, THF/ H_2O, rt, 2 h; (f) R-I (R = **i**), CuI, $Pd(PPh_3)_2Cl_2$, Et_3N, CH_3CN, 45 ˚C, 12 h; or R-Br (R = **j**-methyl ester), CuI, $Pd(PPh_3)_2Cl_2$, Et_3N, CH_3CN, 80 ˚C, 4 h; (g) CH_3OH, 65 ˚C, 12 h.

*2′-O-benzoyl-3-O-descladinosyl-3-O-(N-aminopropylcarbamoyl)-8a-azahomoerythromycin 11,12- cyclic carbonate **(132)***

Following the general procedure K, from compound **119** (2.90 g, 3.49 mmol) and 1,3-diaminopropane (1.46 mL, 17.4 mmol), **132** (1.53 g, 1.83 mmol, 52.4%) was obtained as an off-white solid (column chromatography eluents: DCM/MeOH/$NH_3{\cdot}H_2O$ = 10:0.7:0.05).

2′-O-benzoyl-3-O-descladinosyl-3-O-(N-aminobutylcarbamoyl)-8a-azahomoerythromycin 11,12- cyclic carbonate

(133)

Following the general procedure K, from compound **119** (2.00 g, 2.41 mmol) and 1,4-butanediamine (1.21 mL, 12.0 mmol), **133** (1.25 g, 1.47 mmol, 61.0%) was obtained as an off-white solid (column chromatography eluents: DCM/MeOH/$NH_3{\cdot}H_2O$ = 10:0.7:0.05).

*2′-O-benzoyl-3-O-descladinosyl-3-O-(N-but-3-ynoxy-propylcarbamoyl)-8a-azahomoerythromycin 11,12- cyclic carbonate **(134)***

Following the general procedure L, from compound **132** (1.53 g, 1.83 mmol) and 3-butyn-1-ol (13.0 mL, 183 mmol), **134** (0.657 g, 0.740 mmol, 40.4%) was obtained as an off-white solid (column chromatography eluents: petroleum ether/DCM/EtOH/$NH_3{\cdot}H_2O$ = 3:7:0.1:0.05). HRMS (ESI) $(M + H)^+$ *m/z* 888.4883, calcd for $C_{46}H_{69}N_3O_{14}$ 888.4852. ^{1}H NMR ($CDCl_3$, 400 MHz) δ: 8.03 (d, *J* = 7.6 Hz, 2 H, 2 H-Bz), 7.57 (t, *J* = 7.6 Hz, 1 H, 1 H-Bz), 7.45 (t, *J* = 7.6 Hz, 2 H, 2 H-Bz), 5.92 (d, *J* = 8.0 Hz, 1 H, 8a-CO-NH-), 5.38 (t, 1 H, *J* = 5.6 Hz, 3-O-CO-NH-), 5.12 (d, *J* = 10.0 Hz, 1 H, H-3), 5.05-4.97 (m, 2 H, H-2′, H-13), 4.47 (d, *J* = 4.4 Hz, 1 H, H-11), 4.35 (d, *J* = 7.6 Hz, 1 H, H-1′), 4.01-3.97 (m, 1 H, H-8), 3.71 (d, 1 H, *J* = 5.2 Hz, H-5), 3.62-3.57 (m, 3 H, -C**H**$_2$-O-C**H$_2$**-), 3.51-3.31 (m, 3 H, -CONH-C**H**$_2$-, -C**H**$_2$-O-), 3.21 (s, 3 H, 6-O-CH_3), 2.89-2.84 (m, 1 H, H-3′), 2.69-2.65 (m, 1 H, H-2), 2.52-2.48 (m, 2 H, -C**H$_2$**-C≡CH), 2.28 (s, 6 H, -N(CH_3)$_2$), 2.23-2.21 (m, 1 H, H-7a), 2.03 (t, *J* = 2.4 Hz, 1 H, -CH_2-C≡C**H**), 1.88-1.75 (m, 5 H, -C**H$_2$**-CH_2-O-, H-4, H-14eq, H-4′a), 1.55-1.41 (m, 3 H, H-4′b, H-14ax, H-7b), 1.29-1.26 (m, 9 H, 5′-CH_3, 6-CH_3, 2-CH_3), 1.21 (d, 3 H, *J* = 6.8 Hz, 8-CH_3), 1.14-1.12 (m, 6 H, 10-CH_3, 12-CH_3), 0.87 (t, *J* = 7.2 Hz, 3 H, 15-CH_3), 0.74 (d, *J* = 5.6 Hz, 3 H, 4-CH_3).

*2′-O-benzoyl-3-O-descladinosyl-3-O-(N-pent-4-ynoxy-propylcarbamoyl)-8a-azahomoerythromycin 11,12- cyclic carbonate **(135)***

Following the general procedure L, from compound **132** (2.00 g, 2.40 mmol) and 4-pentyn-1-ol (18 mL, 240 mmol), **135** (0.561 g, 0.640 mmol, 26.7 %) was obtained as an off-white solid (column chromatography eluents: petroleum ether/DCM/EtOH/$NH_3{\cdot}H_2O$ = 3:7:0.1:0.05).

*2′-O-benzoyl-3-O-descladinosyl-3-O-(N-but-3-ynoxy-butylcarbamoyl)-8a-azahomoerythromycin 11,12- cyclic carbonate **(136)***

Following the general procedure L, from compound **133** (1.25 g, 1.47 mmol) and 3-butyn-1-ol (11 mL, 147 mmol), **136** (0.400 g, 0.440 mmol, 29.9%) was obtained as an off-white solid (column chromatography eluents: petroleum ether/DCM/EtOH/$NH_3{\cdot}H_2O$ = 3:7:0.1:0.05). HRMS (ESI) $(M + H)^+$ *m/z* 902.5009, calcd for $C_{47}H_{72}N_3O_{14}$ 902.5012. ^{1}H NMR ($CDCl_3$, 400 MHz) δ: 8.04 (d, *J* = 7.6 Hz, 2 H, 2 H-Bz), 7.58 (t, *J* = 7.6 Hz, 1 H, 1 H-Bz), 7.45 (t, *J* = 7.6 Hz, 2 H, 2 H-Bz), 5.52 (d, *J* = 8.0 Hz, 1 H, 8a-CO-NH-), 5.12 (d, *J* = 10.0 Hz, 1 H, H-3), 5.04-4.97 (m, 2 H, H-2′, H-13), 4.47 (d, *J* = 4.4 Hz, 1 H, H-11), 4.35 (d, *J* = 7.6 Hz, 1 H, H-1′), 4.02-3.95 (m, 1 H, H-8), 3.71 (d, 1 H, *J* = 5.2 Hz, H-5), 3.58-3.50 (m, 4 H, -C**H**$_2$-O-C**H$_2$**-), 3.36-3.31 (m, 1 H, -CONH-C**H**$_2$-), 3.22 (s, 4 H, -CONH-C**H**$_2$-, 6-O-CH_3), 2.91-2.86 (m, 1 H, H-3′), 2.71-2.64 (m, 1 H, H-2), 2.49-2.45 (m, 2 H, -C**H$_2$**-C≡CH), 2.29 (s, 6 H, -N(CH_3)$_2$), 2.24-2.22 (m, 1 H, H-7a), 1.99 (t, *J* = 2.8 Hz, 1 H, -CH_2-C≡C**H**), 1.88-1.73 (m, 5 H, -C**H$_2$**-CH_2-O-, H-4, H-14eq, H-4′a), 1.65 (m, 2 H, -CONH-CH_2-C**H$_2$**-), 1.57-1.42 (m, 3 H, H-4′b, H-14ax, H-7b), 1.29-1.26 (m, 9 H, 5′-CH_3, 6-CH_3, 2-CH_3), 1.21 (d, 3 H, *J* = 6.4 Hz, 8-CH_3), 1.13-1.11 (m, 6 H, 10-CH_3, 12-CH_3), 0.87 (t, *J* = 7.2 Hz, 3 H, 15-CH_3), 0.74 (d, *J* = 7.2 Hz, 3 H, 4-CH_3).

*3-O-descladinosyl-3-O-[N-4-(1,4-dihydro-4-oxo-3-quinolinecarboxylic acid-7-yl)-but-3-ynoxy-propylcarbamoyl]-8a-azahomoerythromycin 11,12- cyclic carbonate **(137i)***

According to the general procedure I-1, from compound **134** (0.201 g, 0.230 mmol) and **12** (0.0860 g, 0.270 mmol), **137i** (25.0 mg, 0.0300 mmol, 11.2%) was obtained as an off-white solid (column chromatography eluents: DCM/MeOH/$NH_3{\cdot}H_2O$ = 10:1:0.5). m.p. 178.8-180.2 ˚C. HRMS (ESI) $(M + H)^+$ *m/z* 971.4869, calcd for $C_{49}H_{70}N_4O_{16}$ 971.4860. ^{1}H NMR (CD_3OD-*d$_4$*, 400 MHz) δ: 8.84 (s, 1 H, 2-quinolyl), 8.31 (d, *J* = 8.4 Hz, 1 H, 5-quinolyl), 7.74 (s, 1 H, 8-quinolyl), 7.45 (d, *J* = 8.4 Hz, 1 H, 6-quinolyl), 6.29 (d, *J* = 6.0 Hz, 1 H, 8a-CO-NH-), 5.64 (s, 1 H, 3-O-CO-NH-), 5.17 (d, *J* = 9.6 Hz, 1 H, H-3), 5.06 (dd, *J* = 8.4, 3.2 Hz, 1 H, H-13), 4.54 (d, *J* = 4.4 Hz, 1 H, H-11), 4.19 (d, *J* = 6.8 Hz, 1 H, H-1′), 3.93-3.89 (m, 2 H, H-5, 1-NH), 3.67-3.61 (m, 4 H, H-8, -C**H**$_2$-O-C**H$_2$**-), 3.45-

3.26 (m, 2 H, H-5′, -C**H**$_2$-O-), 3.45-3.26 (m, 2 H, -CONH-C**H$_2$**-), 3.18 (s, 3 H, 6-O-CH$_3$), 2.75-2.69 (m, 4 H, H-2, H-3′, -C**H$_2$**C≡C-Ar), 2.46 (q, J = 11.2, 4.0 Hz, 1 H, H-10), 2.36 (s, 6 H, -N(CH$_3$)$_2$), 1.98-1.96 (m, 1 H, H-4), 1.88-1.81 (m, 4 H, H-14eq, H-7a, -NH-CH$_2$-C**H$_2$**-CH$_2$-O-), 1.74-1.71 (m, 1 H, H-4′a), 1.63-1.62 (m, 1 H, H-14ax), 1.47-1.43 (m, 4 H, H-4′b, 12-CH$_3$), 1.31-1.23 (m, 13 H, 5′-CH$_3$, 8-CH$_3$, 6-CH$_3$, 2-CH$_3$, H-7b), 1.12 (d, J = 6.8 Hz, 3 H, 10-CH$_3$), 0.95-0.88 (m, 6 H, 4-CH$_3$, 15-CH$_3$). ^{13}C NMR (CDCl$_3$, 100MHz) δ: 177.6, 174.6, 170.9, 168.9, 168.1, 157.5, 153.6, 146.2, 129.5, 128.6, 125.1, 122.4, 101.3, 94.7, 86.4, 82.2, 79.2, 79.0, 77.8, 77.2, 76.0, 69.0, 68.2, 65.7, 49.9, 43.3, 42.2, 41.2, 40.5, 40.3, 38.6, 37.2, 29.8, 29.5, 28.2, 28.1, 25.9, 21.9, 21.8, 19.7, 19.6, 18.6, 14.6, 14.2, 11.6, 9.4, 8.6

*2′-O-benzoyl-3-O-descladinosyl-3-O-(N-but-3-ynoxy-propylcarbamoyl)-8a-azahomoerythromycin **(138)***

Following the general procedure N, from compound **134** (0.657g, 0.740 mmol), **138** (0.338 g, 0.390 mmol, 40.5%) was obtained as an off-white solid (column chromatography eluents: petroleum ether/DCM/EtOH/NH$_3$·H$_2$O = 3:7:0.1:0.05).

*2′-O-benzoyl-3-O-descladinosyl-3-O-(N-pent-4-ynoxy-propylcarbamoyl)-8a-azahomoerythromycin **(139)***

Following the general procedure N, from compound **135** (0.561 g, 0.640 mmol), **139** (0.180 g, 0.210 mmol, 32.8%) was obtained as an off-white solid (column chromatography eluents: petroleum ether/DCM/EtOH/NH$_3$·H$_2$O = 3:7:0.1:0.05).

*2′-O-benzoyl-3-O-descladinosyl-3-O-(N-but-3-ynoxy-butylcarbamoyl)-8a-azahomoerythromycin **(140)***

Following the general procedure N, from compound **136** (0.400 g, 0.440 mmol), **140** (0.210 g, 0.240 mmol, 54.5%) was obtained as an off-white solid (column chromatography eluents: petroleum ether/DCM/EtOH/NH$_3$·H$_2$O = 3:7:0.1:0.05).

*3-O-descladinosyl-3-O-[N-4-(1,4-dihydro-4-oxo-3-quinolinecarboxylic acid-7-yl)-but-3-ynoxy-propylcarbamoyl]-8a-azahomoerythromycin **(141i)***

According to the general procedure I-1, from compound **138** (0.338 g, 0.390 mmol) and **12** (0.148 g, 0.460 mmol), **141i** (35.0 mg, 0.0400 mmol, 9.5%) was obtained as an off-white solid (column chromatography eluents: DCM/MeOH/NH$_3$·H$_2$O = 10:1:0.5). m.p. 175.1-176.2 ˚C. HRMS (ESI) (M + H)$^+$ *m/z* 945.5089, calcd for $C_{48}H_{72}N_4O_{15}$ 945.5067. ^{1}H NMR (CDCl$_3$, 400 MHz) δ: 8.90 (s, 1 H, 2-quinolyl), 8.25 (d, J = 8.4 Hz, 1 H, 5-quinolyl), 7.77 (s, 1 H, 8-quinolyl), 7.42 (d, J = 8.4 Hz, 1 H, 6-quinolyl), 6.31 (d, J = 6.0 Hz, 1 H, 8a-CO-NH-), 5.94 (s, 1 H, 3-O-CO-NH-), 5.12 (d, J = 10.4 Hz, 1 H, H-3), 4.96 (dd, J = 10.8 Hz, 1 H, H-13), 4.12 (d, J = 6.8 Hz, 1 H, H-1′), 3.95 (s, 1 H, 1-NH), 3.85 (s, 1 H, 11-OH), 3.67-3.61 (m, 5 H, H-8, H-5, -C**H$_2$**-O-C**H$_2$**-), 3.45-3.26 (m, 5 H, H-5′, H-11, -CONH-C**H$_2$**-, -C**H**$_2$-O-), 3.19 (s, 3 H, 6-O-CH$_3$), 2.73-2.65 (m, 4 H, H-2, H-3′, -C**H$_2$**C≡C-Ar), 2.46 (q, J = 11.2, 4.0 Hz, 1 H, H-10), 2.38 (s, 6 H, -N(CH$_3$)$_2$), 2.12-2.07 (m, 1 H, H-4), 1.90-1.80 (m, 4 H, H-14eq, H-7a, -NH-CH$_2$-C**H$_2$**-CH$_2$-O-), 1.74-1.68 (m, 1 H, H-4′a), 1.53-1.42 (m, 2 H, H-14ax, H-7b) 1.28-1.19 (m, 13 H, H-4′b, 5′-CH$_3$, 6-CH$_3$, 10-CH$_3$, 8-CH$_3$, 2-CH$_3$), 1.08 (s, 3 H, 12-CH$_3$), 0.97 (d, J = 5.6 Hz, 3 H, 4-CH$_3$), 0.83 (t, J = 7.2 Hz, 3 H, 15-CH$_3$). ^{13}C NMR (CDCl$_3$, 100MHz) δ: 176.2, 175.6, 169.1, 156.8, 146.1, 140.7, 146.1, 140.7, 128.8, 125.5, 123.9, 108.7, 102.6, 92.1, 81.1, 80.3, 78.4, 77.7, 77.6, 75.3, 71.1, 70.7, 69.2, 69.0, 68.7, 65.6, 51.1, 43.3, 43.1, 41.7, 40.1, 39.4, 36.2, 29.6, 29.1, 22.8, 21.4, 21.1, 20.5, 16.5, 15.6, 10.9, 10.6, 9.3.

*3-O-descladinosyl-3-O-[N-4-(3-quinolinecarboxylic acid-7-yl)-but-3-ynoxy-propylcarbamoyl]-8a-azahomoerythromycin **(141j)***

Following the general procedure I-2, from compound **138** (0.246 g, 0.290 mmol) and methyl 7-bromoquinoline-3-carboxylate (0.0910 g, 0.330 mmol), methyl ester-**141j** was obtained. After completion of this reaction, methyl ester-**141j** was treated with LiOH·H$_2$O to remove the protective group, and **141j** (48.0 mg, 0.0500 mmol, 17.8%) was obtained as an off-white solid (column chromatography eluents: DCM/MeOH/NH$_3$·H$_2$O = 10:1:0.5). m.p. 174.8-176.2 ˚C. HRMS (ESI) (M + H)$^+$ *m/z* 929.5130, calcd for $C_{51}H_{78}N_4O_{14}$ 929.5118. ^{1}H NMR (CDCl$_3$, 400 MHz) δ: 9.50 (d, 1 H, J = 1.2 Hz, 2-quinolyl), 8.73 (s, 1 H, 4-quinolyl), 8.07 (s, 1 H, 8-quinolyl), 7.76 (d, J = 8.8 Hz, 1 H, 5-quinolyl), 7.37 (d, J = 8.8 Hz, 1 H, 6-quinolyl), 6.85 (s, 1 H, 3-O-CO-NH-), 5.91 (d, J = 7.2 Hz, 1 H, 8a-CO-NH), 5.19 (d, J = 10.4 Hz, 1 H, H-3), 5.00 (dd, J = 10.8 Hz, 1 H, H-13), 4.20 (d, J = 6.0 Hz, 1 H, H-1′), 3.79 (s, 1 H, 11-

OH), 3.73-3.67 (m, 1 H, H-8), 3.50-3.36 (m, 9 H, H-5, H-5′, H-11, -CONH-C**H$_2$**-, -C**H$_2$**-O-C**H$_2$**-), 3.21 (s, 3 H, 6-O-CH_3), 3.14-3.09 (m, 1 H, H-3′), 2.82-2.78 (m, 1 H, H-2), 2.70 (s, 6 H, -N(CH_3)$_2$), 2.54-2.43 (m, 3 H, H-10, -C**H$_2$**C≡C-Ar), 2.13-2.11 (m, 1 H, H-4), 1.97-1.71 (m, 5 H, H-14eq, H-7a, -NH-CH_2-C**H$_2$**-CH_2-O-, H-4′a), 1.55-1.42 (m, 2 H, H-14ax, H-7b), 1.38-1.26 (m, 10 H, H-4′b, 5′-CH_3, 6-CH_3, 2-CH_3), 1.26-1.19 (m, 6 H, 10-CH_3, 8-CH_3), 1.13-1.12 (m, 6 H, 12-CH_3, 4-CH_3), 0.87 (t, J = 7.2 Hz, 3 H, 15-CH_3). ^{13}C NMR ($CDCl_3$, 100MHz) δ: 176.1, 175.2, 156.9, 152.2, 148.5, 137.3, 130.9, 129.7, 128.7, 126.7, 125.9, 102.9, 81.2, 78.2, 75.3, 70.9, 70.6, 68.9, 68.5, 68.3, 64.8, 50.9, 43.3, 43.1, 42.1, 39.1, 38.4, 35.9, 30.3, 29.9, 29.7, 22.9, 21.4, 21.1, 20.8, 20.5, 16.5, 15.7, 10.9, 10.6, 9.4

3-O-descladinosyl-3-O-[N-5-(1,4-dihydro-4-oxo-3-quinolinecarboxylic acid-7-yl)-pent-4-ynoxy-propylcarbamoyl]-8a-azahomoerythromycin ***(142i)***

According to the general procedure I-1, from compound **139** (0.180 g, 0.210 mmol) and **12** (0.0780 g, 0.250 mmol), **142i** (28.0 mg, 0.0300 mmol, 13.9%) was obtained as an off-white solid (column chromatography eluents: DCM/MeOH/$NH_3 \cdot H_2O$ = 10:1:0.5). m.p. 171.6-173.6 ˚C. HRMS (ESI) $(M + H)^+$ *m/z* 959.5234, calcd for $C_{49}H_{74}N_4O_{15}$ 959.5223. ^{1}H NMR ($CDCl_3$, 400 MHz) δ: 8.83 (s, 1 H, 2-quinolyl), 8.26 (d, J = 8.4 Hz, 1 H, 5-quinolyl), 7.67 (s, 1 H, 8-quinolyl), 7.42 (d, J = 8.4 Hz, 1 H, 6-quinolyl), 6.49 (d, J = 6.8 Hz, 1 H, 8a-CO-NH-), 6.07 (s, 1 H, 3-O-CO-NH-), 5.09 (d, J = 10.8 Hz, 1 H, H-3), 4.96 (dd, 1 H, J = 10.4 Hz, H-13), 4.14 (d, J = 7.6 Hz, 1 H, H-1′), 3.95 (s, 1 H, 1-NH), 3.91 (s, 1 H, 11-OH), 3.62-3.56 (m, 5 H, H-8, H-5, -C**H$_2$**-O-C**H$_2$**-), 3.48-3.27 (m, 5 H, H-5′, H-11, -CONH-C**H$_2$**-, -C**H$_2$**-O-), 3.23 (s, 3 H, 6-O-CH_3), 2.79-2.75 (m, 1 H, H-3′), 2.65-2.58 (m, 3 H, H-2, -C**H$_2$**C≡C-Ar), 2.46 (q, J = 10.8, 4.0 Hz, 1 H, H-10), 2.36 (s, 6 H, -N(CH_3)$_2$), 2.11-2.09 (m, 1 H, H-4), 1.93-1.81 (m, 5 H, H-14eq, H-7a, -NH-CH_2-C**H$_2$**-CH_2-O-, -O-CH_2-C**H$_2$**-), 1.71-1.68 (m, 1 H, H-4′a), 1.46-1.42 (m, 2 H, H-14ax, H-7b), 1.35 (d, J = 6.8 Hz, 3 H, 5′-CH_3), 1.29 (s, 3 H, 6-CH_3), 1.25-1.17 (m, 14 H, -O-CH_2-C**H$_2$**-, H-4′b, 4-CH_3, 10-CH_3, 8-CH_3, 2-CH_3), 0.94 (s, 3 H, 12-CH_3), 0.85 (t, J = 7.2 Hz, 3 H, 15-CH_3). ^{13}C NMR ($CDCl_3$, 100MHz) δ: 177.9, 176.6, 176.1, 168.7, 156.2, 145.8, 140.2, 129.3, 128.8, 125.7, 123.9, 122.4, 108.8, 102.7, 94.4, 81.6, 79.9, 78.8, 78.1, 75.5, 70.9, 70.7, 70.5, 69.8, 65.7, 50.9, 44.5, 43.6, 43.5, 40.5, 40.3, 35.9, 29.7, 29.2, 29.1, 28.8, 22.3, 21.3, 21.2, 20.5, 16.9, 16.4, 15.8, 10.9, 10.8, 9.9.

3-O-descladinosyl-3-O-[N-4-(1,4-dihydro-4-oxo-3-quinolinecarboxylic acid-7-yl)-but-3-ynoxy-butylcarbamoyl]-8a-azahomoerythromycin ***(143i)***

According to the general procedure I-1, from compound **140** (0.210 g, 0.240 mmol) and **12** (0.0910 g, 0280 mmol), **143i** (18.0 mg, 0.0200 mmol, 7.8%) was obtained as an off-white solid (column chromatography eluents: DCM/MeOH/$NH_3 \cdot H_2O$ = 10:1:0.5). m.p. 167.7-169.2 ˚C. HRMS (ESI) $(M + H)^+$ *m/z* 959.5232, calcd for $C_{49}H_{74}N_4O_{15}$ 959.5223. ^{1}H NMR ($CDCl_3$, 400 MHz) δ: 8.87 (s, 1 H, 2-quinolyl), 8.29 (d, J = 8.4 Hz, 1 H, 5-quinolyl), 7.74 (s, 1 H, 8-quinolyl), 7.42 (d, J = 8.4 Hz, 1 H, 6-quinolyl), 6.29 (d, J = 6.0 Hz, 1 H, 8a-CO-NH-), 6.02 (s, 1 H, 3-O-CO-NH-), 5.11 (d, J = 10.4 Hz, 1 H, H-3), 4.95 (dd, J = 10.4 Hz,1 H, H-13), 4.13 (d, J = 6.8 Hz, 1 H, H-1′), 3.96 (s, 1 H, 1-NH), 3.87 (s, 1 H, 11-OH), 3.67-3.55 (m, 5 H, H-8, H-5, -C**H$_2$**-O-C**H$_2$**-), 3.46-3.21 (m, 5 H, H-5′, H-11, -CONH-C**H$_2$**-, -C**H$_2$**-O-), 3.16 (s, 3 H, 6-O-CH_3), 2.76-2.71 (m, 4 H, H-2, H-3′, -C**H$_2$**C≡C-Ar), 2.46 (q, J = 14.0, 6.8 Hz, 1 H, H-10), 2.36 (s, 6 H, -N(CH_3)$_2$), 2.11-2.05 (m, 1 H, H-4, H-7a), 1.92-1.86 (m, 1 H, H-14eq), 1.73-1.71 (m, 5 H, H-4′a, -NH-CH_2-C**H$_2$**-C**H$_2$**-CH_2-O-), 1.52-1.46 (m, 2 H, H-14ax, H-7b), 1.34 (d, J = 12.8 Hz, 3 H, 5′-CH_3), 1.31 (s, 3 H, 6-CH_3), 1.24-1.20 (m, 7 H, H-4′b, 8-CH_3, 2-CH_3), 1.12-1.06 (m, 6 H, 4-CH_3, 10-CH_3), 1.03 (s, 3 H, 12-CH_3), 0.85 (t, J = 7.2 Hz, 3 H, 15-CH_3). ^{13}C NMR ($CDCl_3$, 175MHz) δ: 177.7, 176.1, 175.9, 168.9, 156.6, 145.9, 140.5, 128.9, 128.7, 125.7, 123.9, 123.1, 108.7, 102.6, 91.9, 81.6, 80.3, 78.5, 77.8, 77.4, 75.4, 71.0, 70.7, 69.0, 68.5, 65.7, 51.1, 43.8, 43.3, 43.3, 41.3, 41.1, 40.2, 35.9, 31.9, 29.7, 29.7, 29.0, 27.4, 27.3, 722.7, 22.6, 21.3, 21.1, 20.9, 20.6, 16.5, 15.6, 14.1, 10.9, 10.7, 9.7.

Synthesis of compounds 149

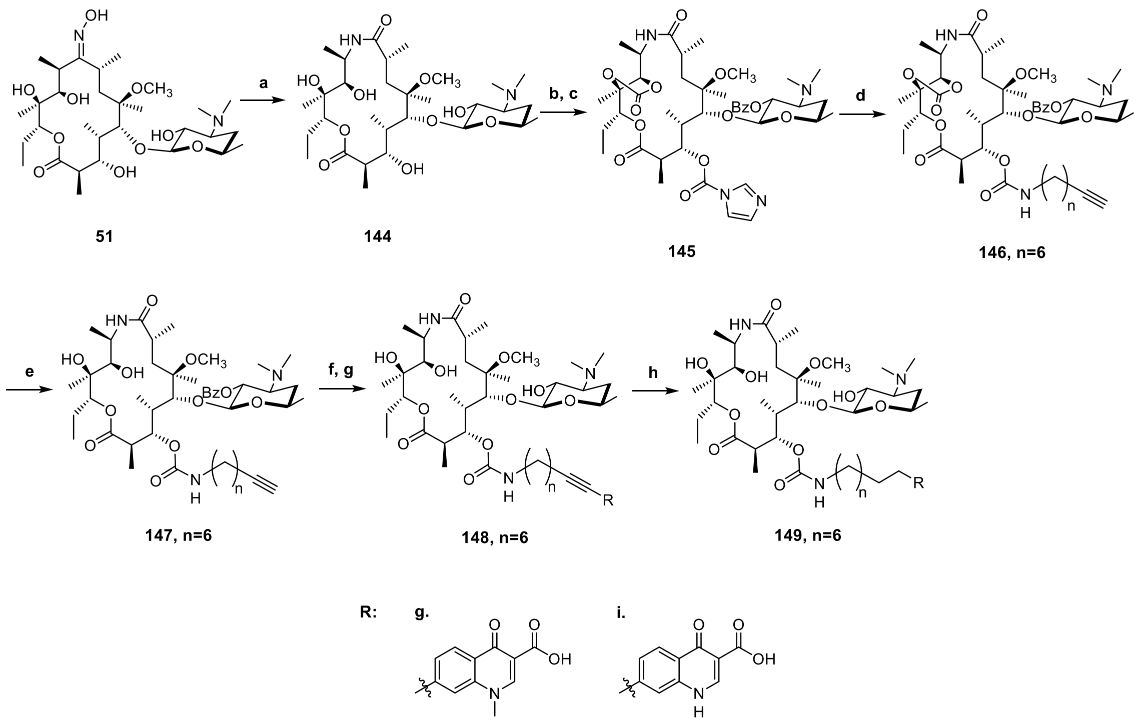

Scheme S16. Synthesis of *9a-azahomoerythromycin* series compounds **149**. Reagents and conditions: (a) tosyl chloride, $NaHCO_3$, acetone/ H_2O, rt, 2 h; (b) benzoic anhydride, DCM, rt, 12 h; (c) CDI, DMAP, DCM, rt, 12 h; (d) **29**, DBU, DMF, rt, 12 h; (e) $LiOH{\cdot}H_2O$, THF/ H_2O, rt, 2 h; (f) R-I (R = **g**, **i**), CuI, $Pd(PPh_3)_2Cl_2$, Et_3N, CH_3CN, 45 ˚C, 12 h; (g) CH_3OH, 65 ˚C, 12 h; (h) HCOOH, $HCOONH_4$, 10% Pd/C, H_2, CH_3OH, rt, 16 h.

3-O-descladinosyl-3-hydroxy-9a-azahomoerythromycin ***(144)***

Compound **51** (5.00 g, 8.27 mmol) was dissolved in acetone (8 mL), and a solution of $NaHCO_3$ (1.04 g, 12.4 mmol) in H_2O (16 mL) was added. The mixture was stirred at 0 ˚C followed by the addition of tosyl chloride (2.52 g, 13.2 mmol) in acetone (8 mL). The mixture was stirred at 25 ˚C for 1 h, and then evaporated under vacuum. The pH of mixture was adjusted to 10 by 2 N NaOH. The CH_2Cl_2 was added to the reaction, The CH_2Cl_2 layer was washed with brine, and evaporated under vacuum to yield **144** (4.50 g, 7.44 mmol, 90.0 %).

2'-O-benzoyl-3-O-descladinosyl-3-O-(1H-imidazol-1-ylcarbonyl)-9a-azahomoerythromycin 11,12- cyclic carbonate ***(145)***

To a stirred solution of **144** (4.50 g, 7.44 mmol) in CH_2Cl_2 (40 mL), benzoic anhydride (2.30 g, 11.2 mmol) was added, and the reaction mixture was stirred at rt for 12 h. After completion of the reaction, the mixture was washed with water and brine. The organic layer was concentrated in vacuo and purified by column chromatography (100-200 mesh silicone, $CH_2Cl_2/EtOH/NH_3.H_2O$ = 10/0.1/0.05) to yield of an intermediate (4.200 g, 5.92 mmol, 79.6%). ^1H NMR ($CDCl_3$, 400 MHz) δ: 8.02 (d, J = 7.2 Hz, 2 H, 2 H-Bz), 7.54 (t, J = 7.2 Hz, 1 H, 1 H-Bz), 7.42 (t, J = 7.6 Hz, 2 H, 2 H-Bz), 7.05 (d, J = 7.6 Hz, 1 H, 9a-CO-NH-), 5.05 (dd, J = 10.4, 7.6 Hz, 1 H, H-2′), 4.82 (dd, J = 7.6 Hz, 1 H, H-13), 4.56 (d, J = 9.6 Hz, 1 H, H-1′), 4.05-4.01 (m, 1 H, H-8, H-11), 3.90 (s, 1 H, 11-OH), 3.69 (q, J = 10.0, 7.2 Hz, 1 H, H-3), 3.57 (d, J = 4.0 Hz, 1 H, H-5), 3.60-3.55 (m, 1 H, H-5′), 3.25 (s, 3 H, 6-*O*-CH_3), 2.95-2.88 (m, 1 H, H-3′), 2.56-2.22 (m, 1 H, H-2), 2.37 (s, 1 H, 12-OH), 2.33-2.28 (s, 7 H, -$N(CH_3)_2$, H-7a), 2.08 (d, J = 7.2 Hz, 1 H, 3-OH), 1.90-1.76 (m, 4 H, H-10, H-4, H-14ax, H-4′a), 1.55-1.39 (m, 3 H, H-4′b, H-7b, H-14eq), 1.29 (s, 3 H, 6-CH_3), 1.28-1.26 (m, 6 H, 5′-CH_3, 8-CH_3), 1.15 (d, 3 H, J = 7.2 Hz, 2-CH_3), 1.07 (d, 3 H, J = 0.8 Hz, 10-CH_3), 1.02 (s, 3 H, 12-CH_3), 0.87 (t, J = 7.2 Hz, 3 H, 15-CH_3), 0.54 (d, J = 7.2 Hz, 3 H, 4-CH_3).

The intermediate (2.80 g, 3.95 mmol), CDI (1.92 g, 11.8 mmol) and DMAP (0.965 g, 7.90 mmol) were dissolved

in dry CH_2Cl_2. The mixture was stirred at 25°C for 12-18 h followed by the addition of CH_2Cl_2 (50 mL) and water (50 mL). The organic layer was washed with water and brine, and concentrated in vacuo to yield **145** (3.00 g, 3.62 mmol, 91.6%).

*2'-O-benzoyl-3-O-descladinosyl-3-O-(N-7-octynylcarbamoyl)-9a-azahomoerythromycin 11,12- cyclic carbonate **(146)***

Following the general procedure H, from compound **145** (1.50 g, 1.87 mmol) and **42** (0.366 g, 2.24 mmol), **146** (0.800 g, 0.900 mmol, 48.1%) was obtained as an off-white solid (column chromatography eluents: petroleum ether/DCM/EtOH/$NH_3{\cdot}H_2O$ = 3:7:0.1:0.05).

*2'-O-benzoyl-3-O-descladinosyl-3-O-(N-7-octynylcarbamoyl)-9a-azahomoerythromycin **(147)***

Following the general procedure N, from compound **146** (0.800 g, 0.900 mmol), **147** (0.350 g, 0.410 mmol, 45.6%) was obtained as an off-white solid (column chromatography eluents: petroleum ether/DCM/EtOH/$NH_3{\cdot}H_2O$ = 3:7:0.1:0.05).

*3-O-descladinosyl-3-O-[N-8-(1,4-dihydro-4-oxo-1-methyl-3-quinolinecarboxylic acid-7-yl)-otc-7-ynylcarbamoyl]-9a-azahomoerythromycin **(148g)***

According to the general procedure I-1, from compound **147** (0.153 g, 0.180 mmol) and **11** (0.0710 g, 0.22 mmol), **148g** (80.0 mg, 0.0800 mmol, 44.4 %) was obtained as an off-white solid (column chromatography eluents: DCM/MeOH/$NH_3{\cdot}H_2O$ = 10:1.2:0.5).

*3-O-descladinosyl-3-O-[N-8-(1,4-dihydro-4-oxo-3-quinolinecarboxylic acid-7-yl)-otc-7-ynylcarbamoyl]-9a-azahomoerythromycin **(148i)***

According to the general procedure I-1, from compound **147** (0.350 g, 0.410 mmol) and **13** (0.154 g, 0.490 mmol), **148i** (35.0 mg, 0.0470 mmol, 11.5%) was obtained as an off-white solid (column chromatography eluents: DCM/MeOH/$NH_3{\cdot}H_2O$ = 10:1.2:0.5). m.p. 182.3-183.5 ˚C. HRMS (ESI) $(M + H)^+$ *m/z* 943.5292, calcd for $C_{49}H_{74}N_4O_{14}$ 943.5274. ^1H NMR ($CDCl_3$, 400 MHz) δ: 8.91 (s, 1 H, 2-quinolyl), 8.21 (d, *J* = 8.0 Hz, 1 H, 5-quinolyl), 7.70 (s, 1 H, 8-quinolyl), 7.38 (d, *J* = 8.4 Hz, 1 H, 6-quinolyl), 7.06 (d, *J* = 6.0 Hz, 1 H, 9a-CO-NH-), 5.88 (s, 1 H, 3-*O*-CO-NH-), 5.16 (d, *J* = 10.4 Hz, 1 H, H-3), 4.62 (dd, *J* = 10.8 Hz, 1 H, H-13), 4.14-4.10 (m, 2 H, H-1′, H-8), 3.90 (s, 1 H, 11-OH), 3.45-3.12 (m, 8 H, H-5, H-11, -CONH-C**H₂**-, 6-*O*-CH_3, H-5′), 2.80-2.72 (m, 2 H, H-2, H-3′), 2.45-2.39 (m, 8 H, -C**H₂**-C≡C-Ar, -N(CH_3)₂), 2.06-2.04 (m, 1 H, H-4), 1.91-1.88 (m, 1 H, H-14eq), 1.70-1.10 (m, 12 H, H-4′a, H-7a, H-14ax, H-7b, -CONH-CH_2C**H₂**C**H₂**C**H₂**C**H₂**-), 1.32-1.28 (m, 4 H, H-4′b, 6-CH_3), 1.28-1.16 (m, 9 H, 8-CH_3, 5′-CH_3, 2-CH_3), 1.10-1.08 (m, 6 H, 10-CH_3, 12-CH_3), 0.96 (d, *J* = 6.8 Hz, 3 H, 4-CH_3), 0.88 (t, *J* = 6.8 Hz, 3 H, 15-CH_3). ^{13}C NMR ($CDCl_3$, 100MHz) δ: 178.1, 177.4, 177.2, 169.5, 156.7, 140.8, 129.3, 128.8, 125.4, 123.5, 123.2, 108.6, 102.7, 94.9, 80.6, 79.9, 79.5, 79.4, 74.0, 70.7, 69.0, 65.7, 50.8, 45.9, 42.9, 41.1, 40.9, 40.2, 35.9, 33.5, 29.8, 29.2, 28.3, 27.9, 26.2, 19.3, 19.2, 18.1, 16.1, 15.8, 15.0, 11.2, 9.1

*3-O-descladinosyl-3-O-[N-8-(1,4-dihydro-1-methyl-4-oxo-3-quinolinecarboxylic acid-7-yl)-octylcarbamoyl]-9a-azahomoerythromycin **(149g)***

According to the general procedure D, from compound **148g** (80.0 mg, 0.0800 mmol), **149g** (36.7 mg, 0.0400 mmol, 50.0%) was obtained as an off-white solid (column chromatography eluents: DCM/MeOH/$NH_3{\cdot}H_2O$ = 10:1.2:0.5). m.p. 137.6-138.8°C. HRMS (ESI) $(M + H)^+$ *m/z* 961.5742, calcd for $C_{50}H_{81}N_4O_{14}$ 961.5744. ^1H NMR ($CDCl_3$, 400 MHz) δ: 8.73 (s, 1 H, 2-quinolyl), 8.42 (d, *J* = 8.0 Hz, 1 H, 5-quinolyl), 7.42 (d, *J* = 8.4 Hz, 1 H, 6-quinolyl), 7.36 (s, 1 H, 8-quinolyl), 5.17 (d, *J* = 6.0 Hz, 1 H, 3-*O*-CO-NH-), 4.62 (d, *J* = 10.4 Hz, 1 H, H-3), 4.12-4.02 (m, 4 H, H-13, N-CH_3), 3.85 (s, 1 H, H-1′), 3.33-3.21 (m, 5 H, -CONH-C**H₂**-, 6-*O*-CH_3), 3.12 (s, 1 H, 11-OH), 3.05-3.03 (m, 1 H, H-5′), 2.84-2.82 (m, 2 H, H-2, H-3′), 2.37-2.34 (m, 2 H, -C**H₂**-Ar), 2.28 (s, 6 H, -N(CH_3)₂), 2.07-2.05 (m, 1 H, H-4), 1.93-1.89 (m, 1 H, H-14eq), 1.70-1.42 (m, 6 H, H-4′a, H-14ax, 2(CH_2)), 1.42-1.32 (m, 7 H, H-7a, H-7b, H-4′b, 2(CH_2), 1.28 (s, 3 H, 6-CH_3), 1.21-1.18 (m, 9 H, 8-CH_3, 5′-CH_3, 2-CH_3), 1.11-1.08 (m, 6 H, 10-CH_3, 12-CH_3), 1.00 (d, *J* = 6.8 Hz, 3 H, 4-CH_3), 0.90 (t, *J* = 7.2 Hz, 3 H, 15-CH_3). ^{13}C NMR ($CDCl_3$, 100MHz) δ: 178.4, 178.1, 177.0, 167.2, 156.5, 149.0, 140.5, 127.2, 127.0, 115.6, 108.6, 103.6, 81.6, 79.6, 74.1, 70.7, 69.4, 65.8, 50.7, 45.8, 42.9, 42.0, 41.1, 40.4, 36.6, 35.9, 33.4, 30.1, 29.7, 29.3, 29.2, 26.8, 21.2, 19.1, 17.9, 16.1, 15.8, 15.1, 11.2, 9.1.

Synthesis of compounds 152

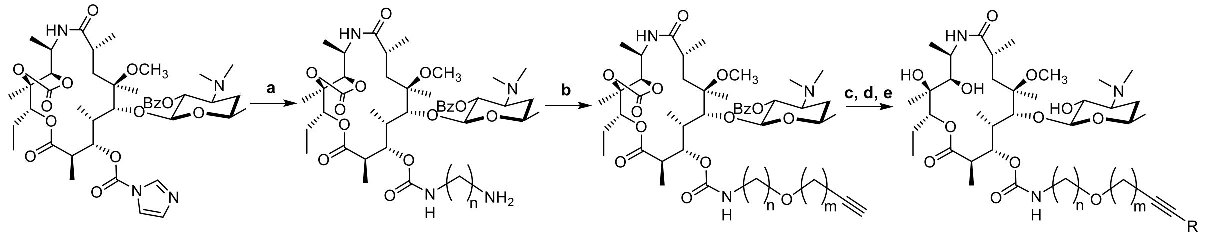

145 150, n=3 151, n=3, m=2 152, n=3, m=2

R: i.

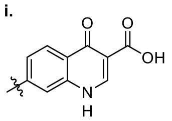

Scheme S17. Synthesis of *9a-azahomoerythromycin* series compounds **152**. Reagents and conditions: (a) 1,3-diaminopropane, DBU, DMF, rt, 3 h; (b) 3-butyn-1-ol, $NaNO_2$, HCOOH, -15˚C, 12-72 h; (c) $LiOH·H_2O$, THF/ H_2O, rt, 2 h; (d) R-I (R = **i**), CuI, $Pd(PPh_3)_2Cl_2$, Et_3N, CH_3CN, 45 ˚C, 12 h; (e) CH_3OH, 65 ˚C, 12 h.

*2'-O-benzoyl-3-O-descladinosyl-3-O-(N-aminopropylcarbamoyl)-9a-azahomoerythromycin 11,12- cyclic carbonate **(150)***

According to the general procedure K, from compound **145** (3.00 g, 3.62 mmol) and 1,3-diaminopropane (1.51 mL, 0.0180 mmol), **150** (1.60 g, 1.92 mmol, 53.0%) was obtained as an off-white solid (column chromatography eluents: DCM/MeOH/$NH_3·H_2O$ = 10:1.2:0.5).

*2'-O-benzoyl-3-O-descladinosyl-3-O-(N-but-3-ynoxy-propylcarbamoyl)-9a-azahomoerythromycin 11,12- cyclic carbonate **(151)***

According to the general procedure M, from compound **150** (1.60 g, 1.92 mmol) and 1,3-diaminopropane (14 mL, 0.192 mol), **151** (0.508 g, 0.570 mmol, 29.7%) was obtained as an off-white solid (column chromatography eluents: DCM/MeOH/$NH_3·H_2O$ = 10:1.2:0.5). HRMS (ESI) $(M + H)^+$ *m/z* 888.4878, calcd for $C_{46}H_{69}N_3O_{14}$ 888.4852. ^1H NMR ($CDCl_3$, 400 MHz) δ: 8.02 (d, *J* = 7.2 Hz, 2 H, 2 H-Bz), 7.55 (t, *J* = 6.0 Hz, 1 H, 1 H-Bz), 7.45 (t, *J* = 7.6 Hz, 2 H, 2 H-Bz), 5.55 (t, 1 H, *J* = 5.6 Hz, 3-*O*-CO-NH-), 5.03-4.95 (m, 2 H, H-2′, H-3), 4.89 (dd, *J* = 10.8 Hz, 1 H, H-13), 4.38 (d, *J* = 7.2 Hz, 1 H, H-11), 4.32 (d, *J* = 8.2 Hz, 1 H, H-1′), 4.25-4.19 (m, 1 H, H-8), 4.96 (d, 1 H, *J* = 2.8 Hz, H-5), 3.64-3.59 (m, 3 H, -C**H**$_2$-O-C**H**$_2$-), 3.32-3.31 (m, 3 H, -CONH-C**H**$_2$-, -C**H**$_2$-O-), 3.32 (s, 3 H, 6-*O*-CH_3), 2.89-2.75 (m, 2 H, H-3′, H-2), 2.55-2.50 (m, 2 H, -C**H**$_2$-C≡CH), 2.29 (s, 6 H, -N(CH_3)$_2$), 2.06 (t, *J* = 2.8 Hz, 1 H, -C≡CH), 2.00-1.95 (m, 1 H, H-7a), 1.83-1.71 (m, 5 H, -C**H**$_2$-CH$_2$-O, H-4, H-14eq, H-4′a), 1.60-1.41 (m, 3 H, H-4′b, H-14ax, H-7b), 1.29 (s, 3 H, 6-CH_3), 1.28-1.26 (m, 6 H, 5′-CH_3, 2-CH_3), 1.16-1.13 (m, 6 H, 8-CH_3, 12-CH_3), 1.10 (d, *J* = 7.2 Hz, 3 H, 10-CH_3), 0.86 (t, *J* = 7.2 Hz, 3 H, 15-CH_3), 0.70 (d, *J* = 7.2 Hz, 3 H, 4-CH_3).

*3-O-descladinosyl-3-O-[N-4-(1,4-dihydro-4-oxo-3-quinolinecarboxylic acid-7-yl)-but-3-ynoxy-propylcarbamoyl]-9a-azahomoerythromycin **(152i)***

According to the general procedure N, from compound **151** (0.508 g, 0.570 mmol), 11,12-dihydroxy-**151** (0.180 g, 0.210 mmol, 36.8%) was obtained as an off-white solid (column chromatography eluents: petroleum ether/DCM/EtOH/$NH_3·H_2O$ = 3:7:0.1:0.1).

Following the general procedure I-1, from compound 11,12-dihydroxy-**151** (0.180 g, 0.210 mmol) and **12** (0.0780 g, 0.250 mmol), **152i** (20.0 mg, 0.0210 mmol, 10.1%) was obtained as an off-white solid (column chromatography eluents: DCM/MeOH/$NH_3·H_2O$ = 10:1:0.5). m.p. 167.8-169.5 ˚C. HRMS (ESI) $(M + H)^+$ *m/z* 945.5061, calcd for $C_{48}H_{72}N_4O_{15}$ 945.5067. ^1H NMR ($CDCl_3$, 400 MHz) δ: 8.88 (s, 1 H, 2-quinolyl), 8.28 (d, *J* = 7.2 Hz, 1 H, 5-quinolyl), 7.75 (s, 1 H, 8-quinolyl), 7.43 (d, *J* = 7.2 Hz, 1 H, 6-quinolyl), 7.00 (d, *J* = 7.6 Hz, 1 H, 8a-CO-NH-), 6.04 (s, 1 H, 3-*O*-CO-NH-), 5.16 (d, *J* = 10.8 Hz, 1 H, H-3), 4.63 (dd, *J* = 10.8 Hz, 1 H, H-13), 4.16-4.11 (m, 1 H, H-1′, H-8), 3.95

(s, 1 H, 11-OH), 3.65-3.33 (m, 6 H, H-5, -C**H**$_2$-O-C**H**$_2$-, H-5′), 3.19 (m, 5 H, -CONH-C**H**$_2$-, 6-*O*-CH_3), 3.11 (s, 1 H, H-11), 2.74-2.73 (m, 4 H, H-2, H-3′, -C**H**$_2$-C≡C-Ar), 2.38 (s, 6 H, -N$(CH_3)_2$), 2.05-2.03 (m, 1 H, H-4), 1.93-1.88 (m, 3 H, H-14eq, -NH-CH_2-C**H**$_2$-CH_2-O-), 1.74-1.67 (m, 1 H, H-7a, H-4′a), 1.57-1.52 (m, 2 H, H-14a), 1.38-1.34 (m, 1 H, H-7b), 1.27-1.18 (m, 10 H, H-4′b, 5′-CH_3, 6-CH_3, 2-CH_3), 1.13 (d, J = 6.8 Hz, 3 H, 8-CH_3), 1.09-1.07 (m, 6 H, 10-CH_3, 12-CH_3), 0.97-0.83 (m, 6 H, 4-CH_3, 15-CH_3). ^{13}C NMR ($CDCl_3$, 176 MHz) δ: 178.2, 178.1, 177.2, 168.4, 156.8, 145.5, 139.9, 128.9, 128.7, 125.8, 124.2, 122.7, 108.6, 102.5, 92.3, 80.3, 80.0, 79.6, 79.4, 74.0, 73.9, 70.8, 69.4, 69.0, 68.6, 65.6, 50.8, 45.9, 42.8, 40.8, 40.2, 39.8, 36.1, 33.5, 29.7, 29.4, 29.1, 21.1, 20.8, 19.2, 18.2, 16.0, 15.8, 14.9, 10.2, 9.1

Spectra of compounds

57a

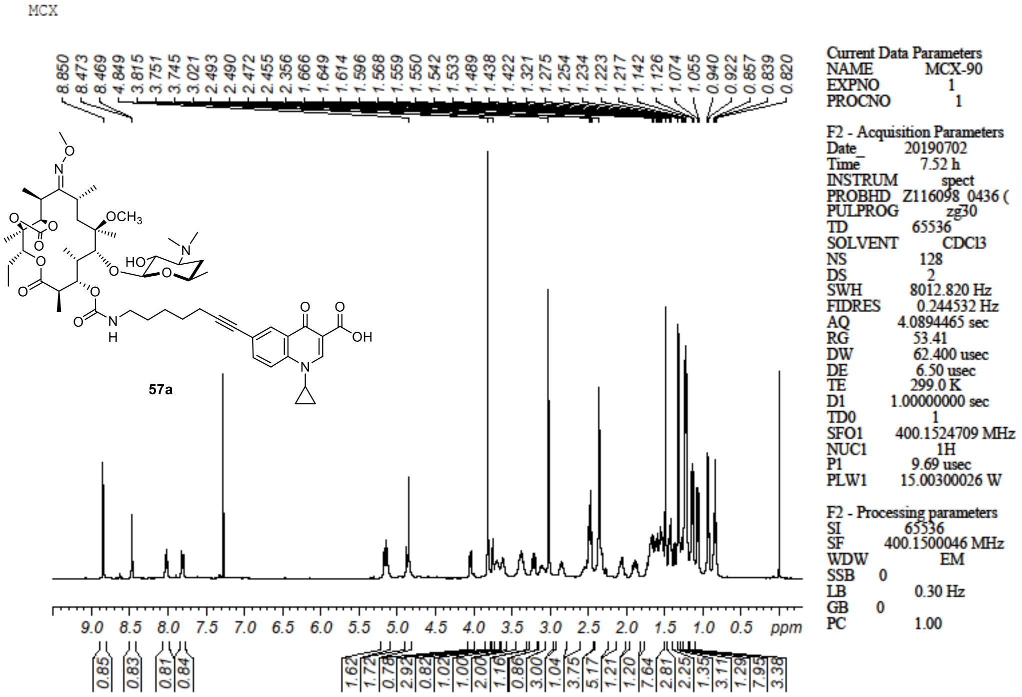

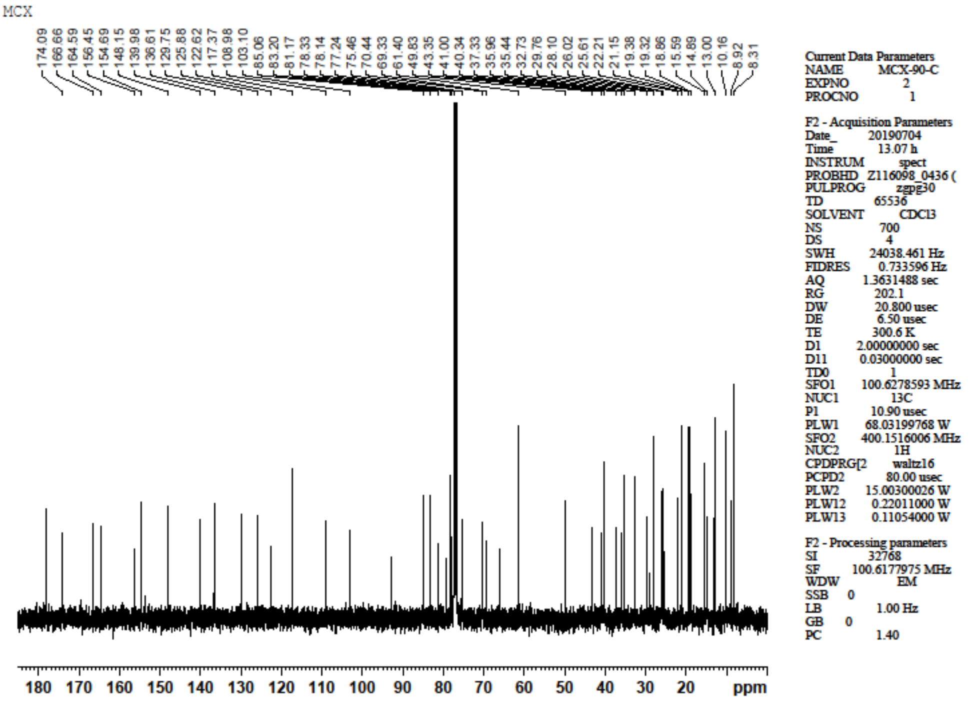

57c

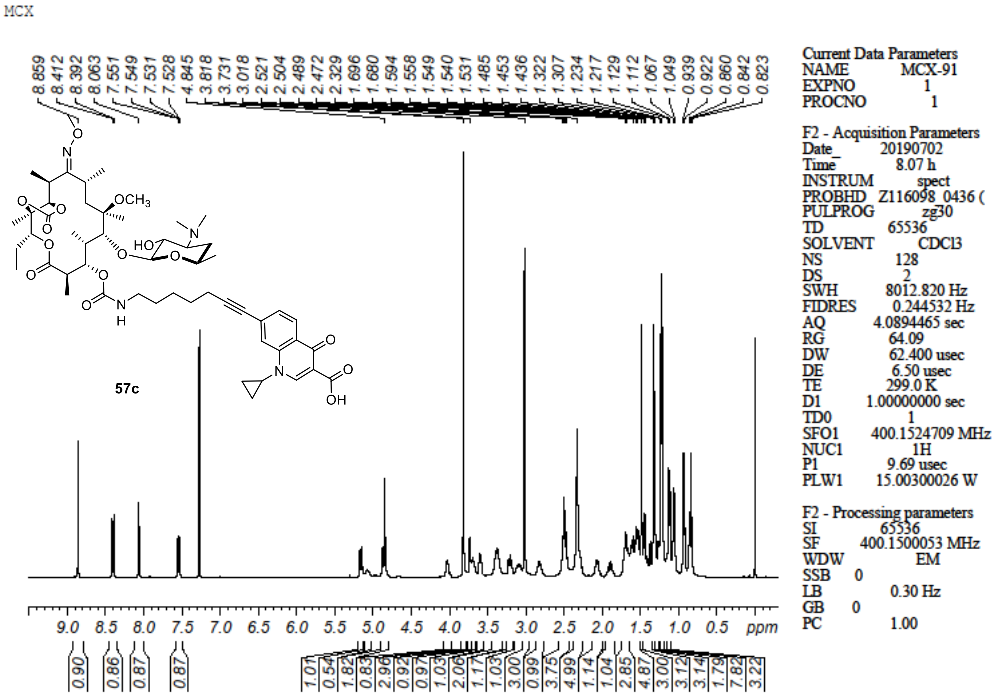
MCX
57c
Current Data Parameters
NAME MCX-91
EXPNO 1
PROCNO 1
F2 - Acquisition Parameters
Date_ 20190702
Time 8.07 h
INSTRUM spect
PROBHD Z116098_0436 (
PULPROG zg30
TD 65536
SOLVENT CDCl3
NS 128
DS 2
SWH 8012.820 Hz
FIDRES 0.244532 Hz
AQ 4.0894465 sec
RG 64.09
DW 62.400 usec
DE 6.50 usec
TE 299.0 K
D1 1.00000000 sec
TD0 1
SFO1 400.1524709 MHz
NUC1 1H
P1 9.69 usec
PLW1 15.00300026 W
F2 - Processing parameters
SI 65536
SF 400.1500053 MHz
WDW EM
SSB 0
LB 0.30 Hz
GB 0
PC 1.00
ppm

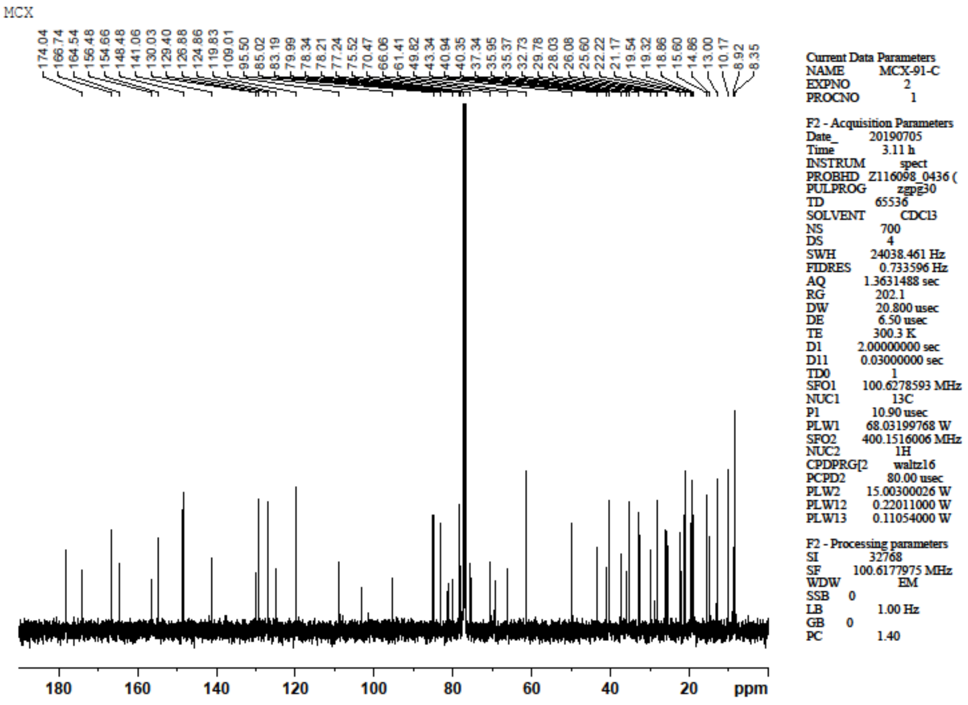
MCX
Current Data Parameters
NAME MCX-91-C
EXPNO 2
PROCNO 1
F2 - Acquisition Parameters
Date_ 20190705
Time 3.11 h
INSTRUM spect
PROBHD Z116098_0436 (
PULPROG zgpg30
TD 65536
SOLVENT CDCl3
NS 700
DS 4
SWH 24038.461 Hz
FIDRES 0.733596 Hz
AQ 1.3631488 sec
RG 202.1
DW 20.800 usec
DE 6.50 usec
TE 300.3 K
D1 2.00000000 sec
D11 0.03000000 sec
TD0 1
SFO1 100.6278593 MHz
NUC1 13C
P1 10.90 usec
PLW1 68.03199768 W
SFO2 400.1516006 MHz
NUC2 1H
CPDPRG[2 waltz16
PCPD2 80.00 usec
PLW2 15.00300026 W
PLW12 0.22011000 W
PLW13 0.11054000 W
F2 - Processing parameters
SI 32768
SF 100.6177975 MHz
WDW EM
SSB 0
LB 1.00 Hz
GB 0
PC 1.40
ppm

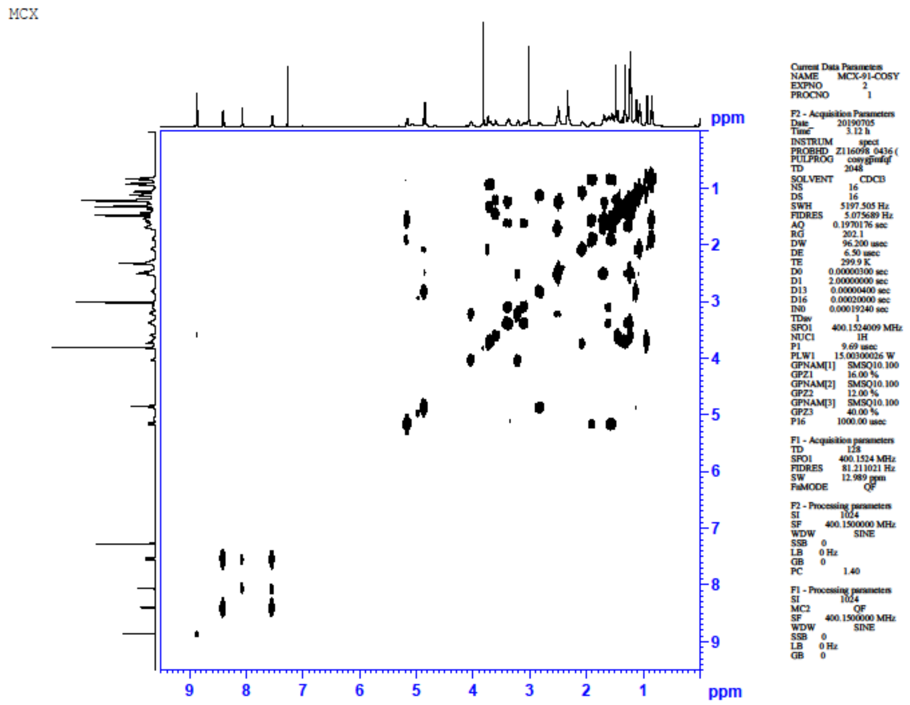

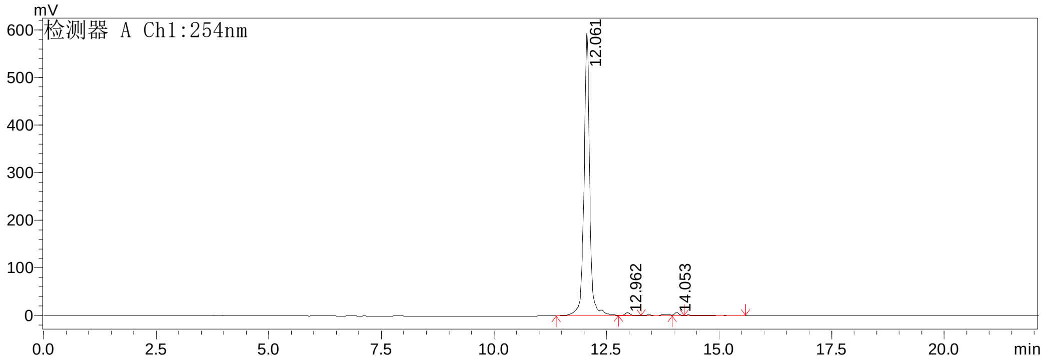

Retention	Peak start	Peak End	peak area	peak height	Area %
12.061	11.383	15.583	5337521	593554	98.2592
12.962	12.767	13.275	46513	6036	0.8563
14.053	13.958	14.225	48049	6903	0.8845

57e

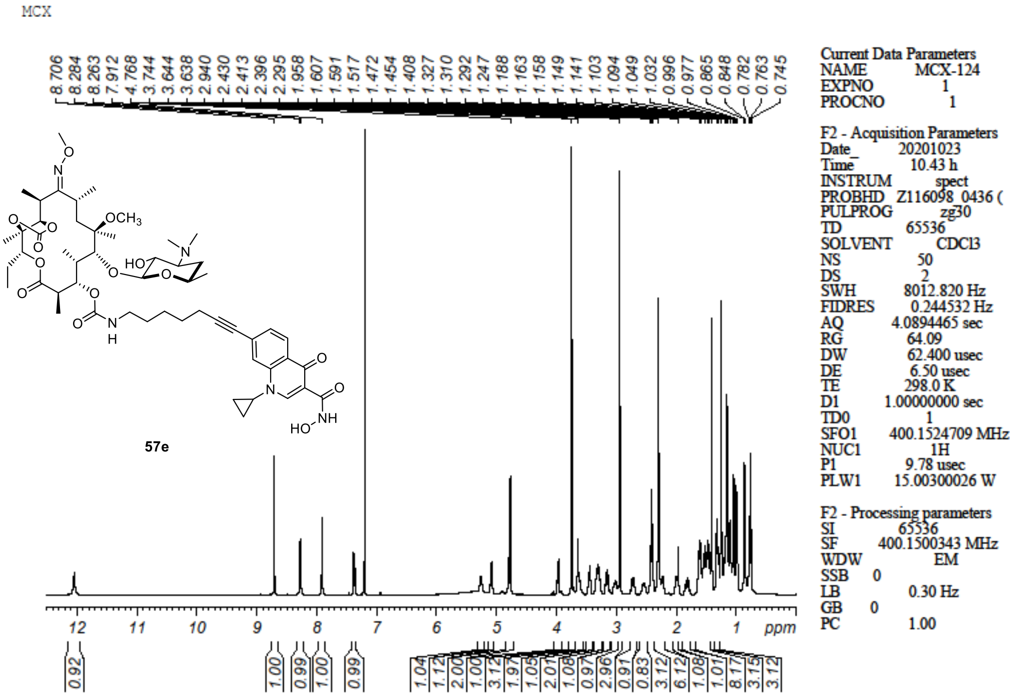
MCX
57e
Current Data Parameters
NAME MCX-124
EXPNO 1
PROCNO 1
F2 - Acquisition Parameters
Date_ 20201023
Time 10.43 h
INSTRUM spect
PROBHD Z116098_0436 (
PULPROG zg30
TD 65536
SOLVENT CDCl3
NS 50
DS 2
SWH 8012.820 Hz
FIDRES 0.244532 Hz
AQ 4.0894465 sec
RG 64.09
DW 62.400 usec
DE 6.50 usec
TE 298.0 K
D1 1.00000000 sec
TD0 1
SFO1 400.1524709 MHz
NUC1 1H
P1 9.78 usec
PLW1 15.00300026 W
F2 - Processing parameters
SI 65536
SF 400.1500343 MHz
WDW EM
SSB 0
LB 0.30 Hz
GB 0
PC 1.00
ppm

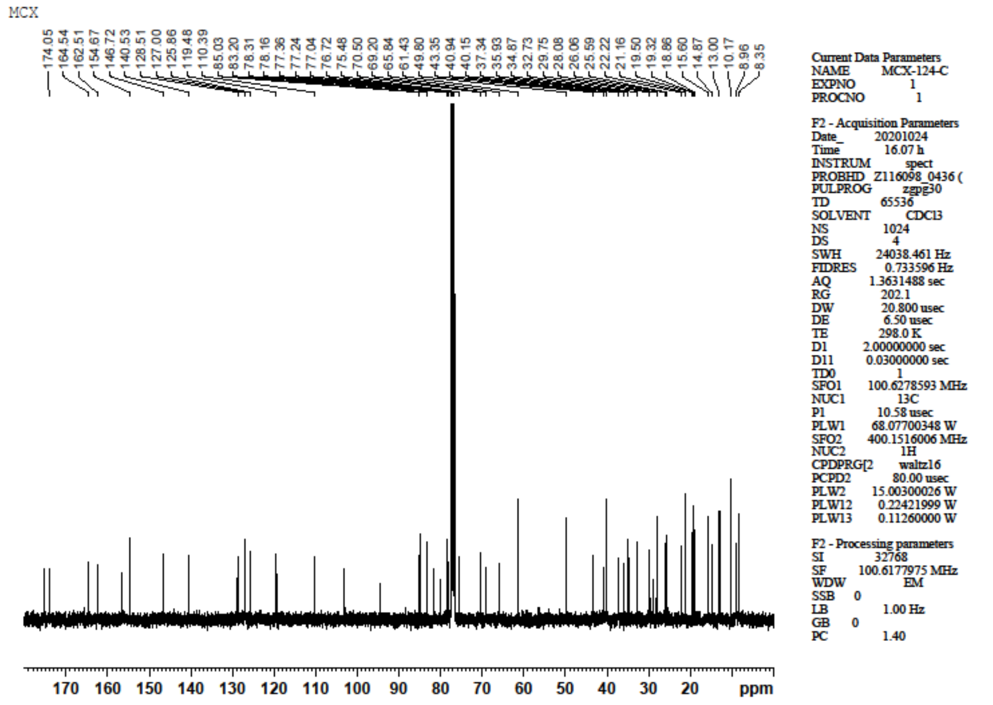
MCX
Current Data Parameters
NAME MCX-124-C
EXPNO 1
PROCNO 1
F2 - Acquisition Parameters
Date_ 20201024
Time 16.07 h
INSTRUM spect
PROBHD Z116098_0436 (
PULPROG zgpg30
TD 65536
SOLVENT CDCl3
NS 1024
DS 4
SWH 24038.461 Hz
FIDRES 0.733596 Hz
AQ 1.3631488 sec
RG 202.1
DW 20.800 usec
DE 6.50 usec
TE 298.0 K
D1 2.00000000 sec
D11 0.03000000 sec
TD0 1
SFO1 100.6278593 MHz
NUC1 13C
P1 10.58 usec
PLW1 68.07700348 W
SFO2 400.1516006 MHz
NUC2 1H
CPDPRG[2 waltz16
PCPD2 80.00 usec
PLW2 15.00300026 W
PLW12 0.22421999 W
PLW13 0.11260000 W
F2 - Processing parameters
SI 32768
SF 100.6177975 MHz
WDW EM
SSB 0
LB 1.00 Hz
GB 0
PC 1.40
ppm

58c

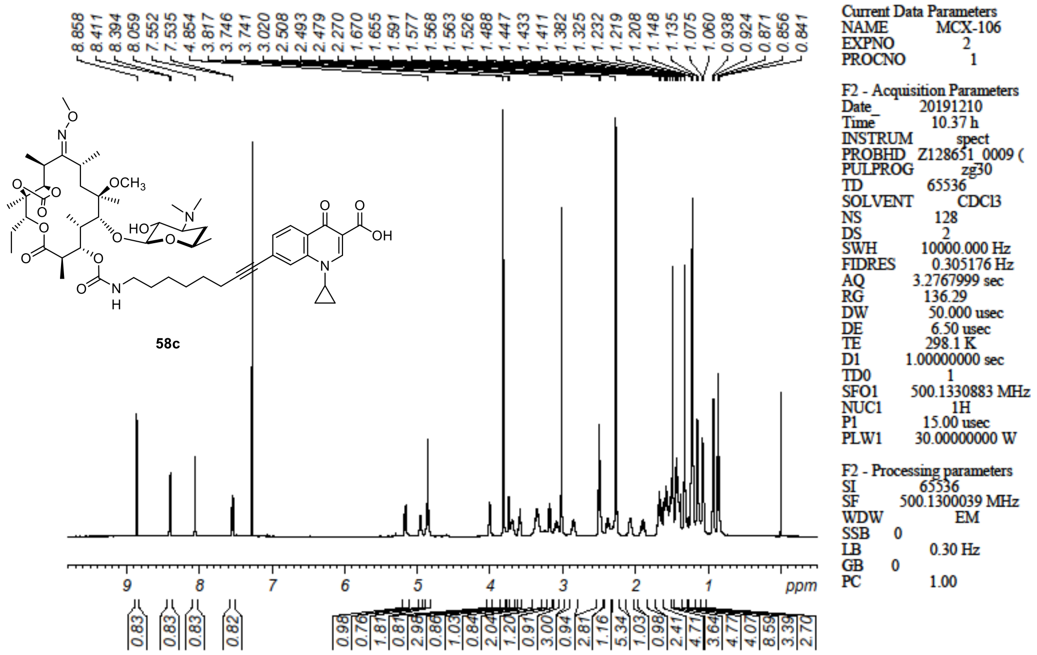
8.858
8.411
8.394
8.059
7.552
7.535
4.854
3.817
3.746
3.741
3.020
2.508
2.493
2.479
2.270
1.670
1.655
1.591
1.577
1.568
1.563
1.526
1.488
1.447
1.433
1.411
1.382
1.325
1.232
1.219
1.208
1.148
1.135
1.075
1.060
0.938
0.924
0.871
0.856
0.841
OCH3
HO
OH
58c
9
8
7
6
5
4
3
2
1
ppm
0.83
0.83
0.83
0.83
0.82
Current Data Parameters
NAME MCX-106
EXPNO 2
PROCNO 1
F2 - Acquisition Parameters
Date_ 20191210
Time 10.37 h
INSTRUM spect
PROBHD Z128651_0009 (
PULPROG zg30
TD 65536
SOLVENT CDCl3
NS 128
DS 2
SWH 10000.000 Hz
FIDRES 0.305176 Hz
AQ 3.2767999 sec
RG 136.29
DW 50.000 usec
DE 6.50 usec
TE 298.1 K
D1 1.00000000 sec
TD0 1
SFO1 500.1330883 MHz
NUC1 1H
P1 15.00 usec
PLW1 30.00000000 W
F2 - Processing parameters
SI 65536
SF 500.1300039 MHz
WDW EM
SSB 0
LB 0.30 Hz
GB 0
PC 1.00

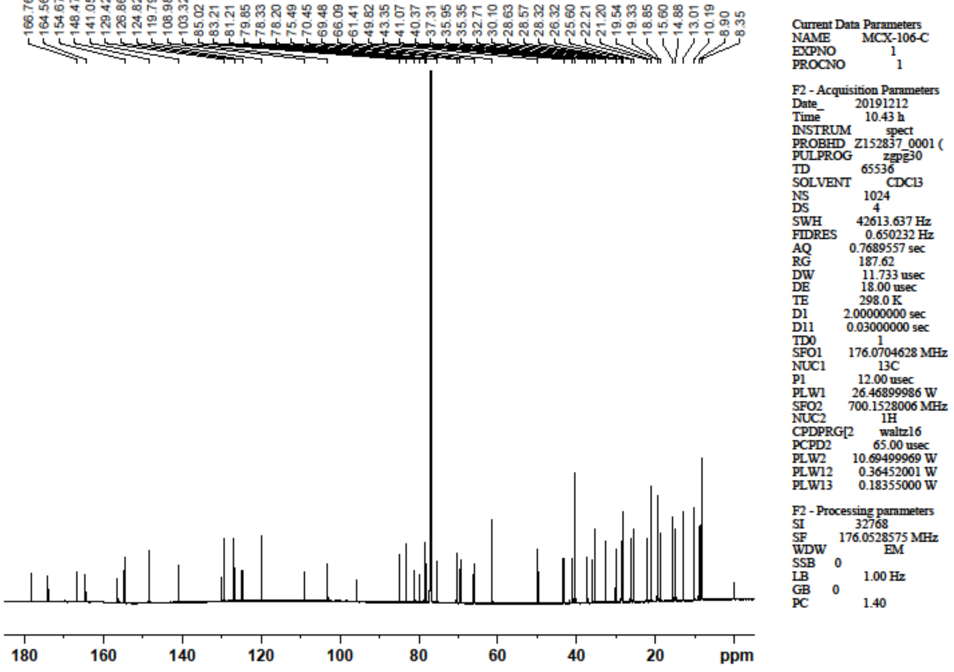
166.76
164.56
154.67
148.47
141.05
129.42
126.86
124.82
119.79
108.98
103.32
85.02
83.21
81.21
79.85
78.33
78.20
75.49
70.45
69.48
66.09
61.41
49.82
43.35
41.07
40.37
37.31
35.95
35.35
32.71
30.10
28.63
28.57
28.32
26.32
25.60
22.21
21.20
19.54
19.33
18.85
15.60
14.88
13.01
10.19
8.90
8.35
180
160
140
120
100
80
60
40
20
ppm
Current Data Parameters
NAME MCX-106-C
EXPNO 1
PROCNO 1
F2 - Acquisition Parameters
Date_ 20191212
Time 10.43 h
INSTRUM spect
PROBHD Z152837_0001 (
PULPROG zgpg30
TD 65536
SOLVENT CDCl3
NS 1024
DS 4
SWH 42613.637 Hz
FIDRES 0.650232 Hz
AQ 0.7689557 sec
RG 187.62
DW 11.733 usec
DE 18.00 usec
TE 298.0 K
D1 2.00000000 sec
D11 0.03000000 sec
TD0 1
SFO1 176.0704628 MHz
NUC1 13C
P1 12.00 usec
PLW1 26.46899986 W
SFO2 700.1528006 MHz
NUC2 1H
CPDPRG[2 waltz16
PCPD2 65.00 usec
PLW2 10.69499969 W
PLW12 0.36452001 W
PLW13 0.18355000 W
F2 - Processing parameters
SI 32768
SF 176.0528575 MHz
WDW EM
SSB 0
LB 1.00 Hz
GB 0
PC 1.40

61a

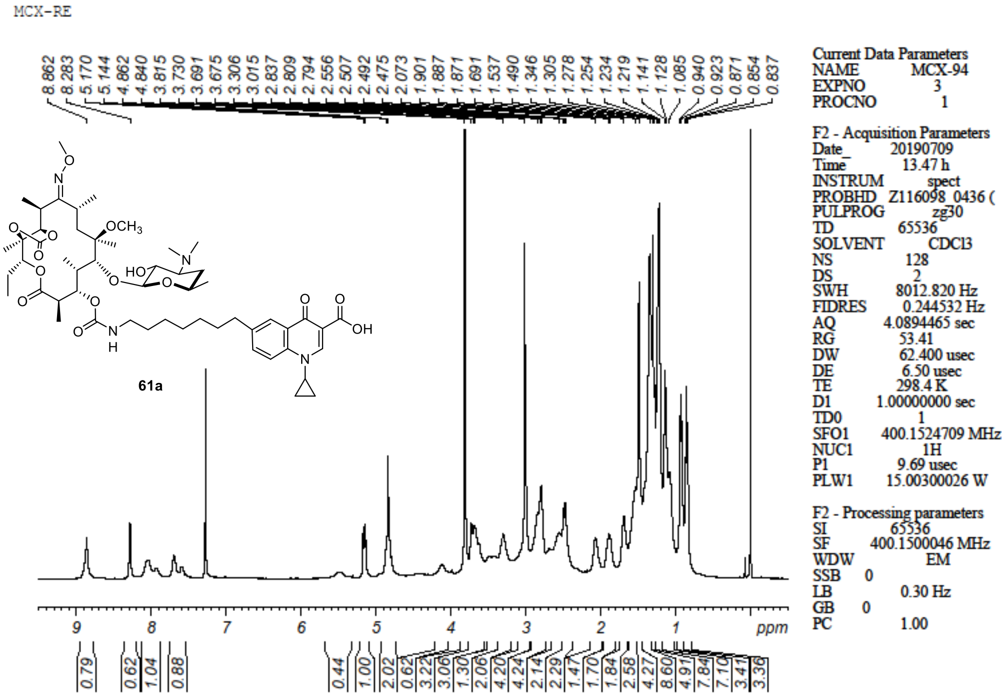
MCX-RE
8.862 8.283 5.170 5.144 4.862 4.840 3.815 3.730 3.691 3.675 3.306 3.015 2.837 2.809 2.794 2.556 2.507 2.492 2.475 2.073 1.901 1.887 1.871 1.691 1.537 1.490 1.346 1.305 1.278 1.254 1.234 1.219 1.141 1.128 1.085 0.940 0.923 0.871 0.854 0.837
61a
9 8 7 6 5 4 3 2 1 ppm
0.79 0.62 1.04 0.88 0.44 1.00 2.02 0.62 3.22 3.06 1.30 2.06 4.20 4.24 2.14 2.29 1.47 1.70 1.84 2.58 4.27 8.60 4.91 7.84 7.10 3.41 3.36
Current Data Parameters
NAME MCX-94
EXPNO 3
PROCNO 1
F2 - Acquisition Parameters
Date_ 20190709
Time 13.47 h
INSTRUM spect
PROBHD Z116098_0436 (
PULPROG zg30
TD 65536
SOLVENT CDCl3
NS 128
DS 2
SWH 8012.820 Hz
FIDRES 0.244532 Hz
AQ 4.0894465 sec
RG 53.41
DW 62.400 usec
DE 6.50 usec
TE 298.4 K
D1 1.00000000 sec
TD0 1
SFO1 400.1524709 MHz
NUC1 1H
P1 9.69 usec
PLW1 15.00300026 W
F2 - Processing parameters
SI 65536
SF 400.1500046 MHz
WDW EM
SSB 0
LB 0.30 Hz
GB 0
PC 1.00

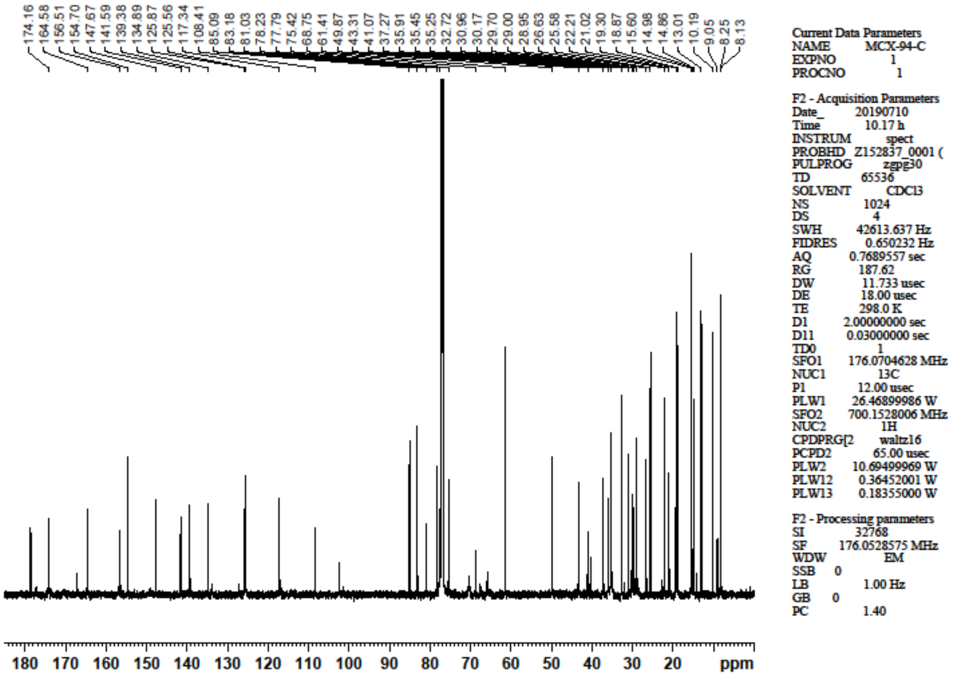
174.16 164.58 156.51 154.70 147.67 141.59 139.38 134.89 125.87 125.56 117.34 108.41 85.09 83.18 81.03 78.23 77.79 75.42 68.75 61.41 49.87 43.31 41.07 37.27 35.91 35.45 35.25 32.72 30.96 30.17 29.70 29.00 28.95 26.63 25.58 22.21 21.02 19.30 18.87 15.60 14.98 14.86 13.01 10.19 9.05 8.25 8.13
180 170 160 150 140 130 120 110 100 90 80 70 60 50 40 30 20 ppm
Current Data Parameters
NAME MCX-94-C
EXPNO 1
PROCNO 1
F2 - Acquisition Parameters
Date_ 20190710
Time 10.17 h
INSTRUM spect
PROBHD Z152837_0001 (
PULPROG zgpg30
TD 65536
SOLVENT CDCl3
NS 1024
DS 4
SWH 42613.637 Hz
FIDRES 0.650232 Hz
AQ 0.7689557 sec
RG 187.62
DW 11.733 usec
DE 18.00 usec
TE 298.0 K
D1 2.00000000 sec
D11 0.03000000 sec
TD0 1
SFO1 176.0704628 MHz
NUC1 13C
P1 12.00 usec
PLW1 26.46899986 W
SFO2 700.1528006 MHz
NUC2 1H
CPDPRG[2 waltz16
PCPD2 65.00 usec
PLW2 10.69499969 W
PLW12 0.36452001 W
PLW13 0.18355000 W
F2 - Processing parameters
SI 32768
SF 176.0528575 MHz
WDW EM
SSB 0
LB 1.00 Hz
GB 0
PC 1.40

61c

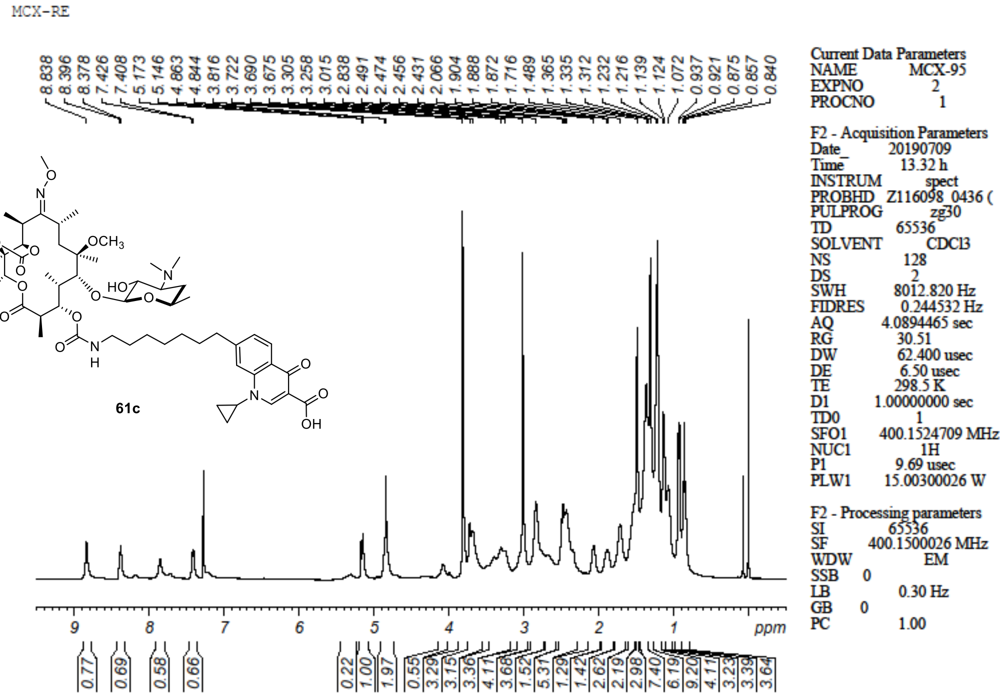
MCX-RE
8.838 8.396 8.378 7.426 7.408 5.173 5.146 4.863 4.844 3.816 3.722 3.690 3.675 3.305 3.258 3.015 2.838 2.491 2.474 2.456 2.431 2.066 1.904 1.888 1.872 1.716 1.489 1.365 1.335 1.312 1.232 1.216 1.139 1.124 1.072 0.937 0.921 0.875 0.857 0.840
OCH3
HO
61c
OH
ppm
0.77 0.69 0.58 0.66 0.22 1.00 1.97 0.55 3.29 3.15 3.36 4.11 3.66 1.52 5.31 1.29 1.42 2.62 2.19 2.98 7.40 6.19 9.20 4.11 3.23 3.39 3.64
Current Data Parameters
NAME MCX-95
EXPNO 2
PROCNO 1
F2 - Acquisition Parameters
Date_ 20190709
Time 13.32 h
INSTRUM spect
PROBHD Z116098_0436 (
PULPROG zg30
TD 65536
SOLVENT CDCl3
NS 128
DS 2
SWH 8012.820 Hz
FIDRES 0.244532 Hz
AQ 4.0894465 sec
RG 30.51
DW 62.400 usec
DE 6.50 usec
TE 298.5 K
D1 1.00000000 sec
TD0 1
SFO1 400.1524709 MHz
NUC1 1H
P1 9.69 usec
PLW1 15.00300026 W
F2 - Processing parameters
SI 65536
SF 400.1500026 MHz
WDW EM
SSB 0
LB 0.30 Hz
GB 0
PC 1.00

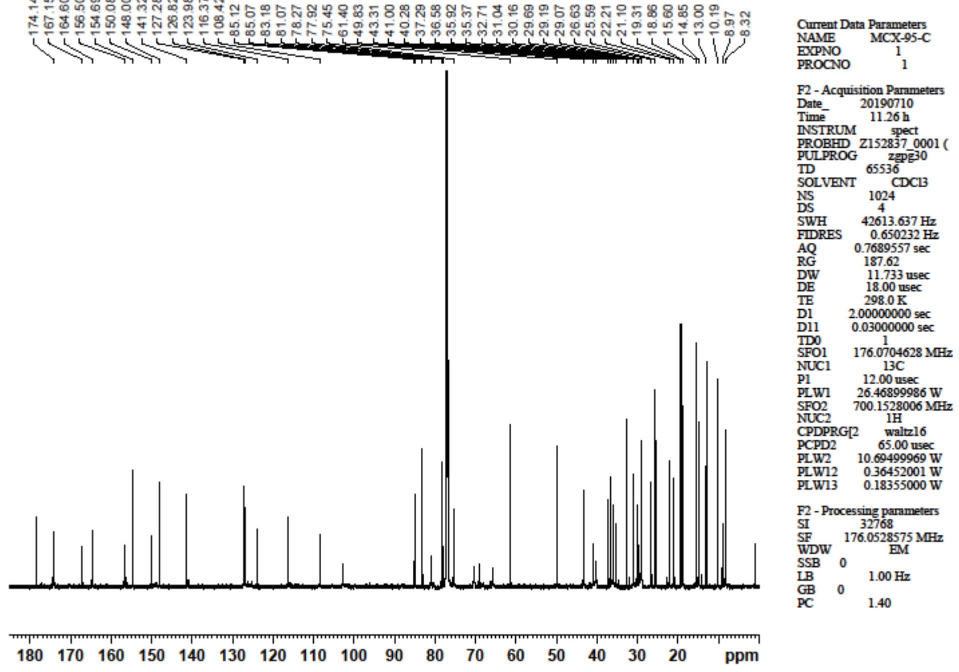
174.14 167.15 164.60 156.50 154.69 150.08 148.00 141.32 127.28 126.82 123.98 116.37 108.42 85.12 85.07 83.18 81.07 78.27 77.92 75.45 61.40 49.83 43.31 41.00 40.28 37.29 36.58 35.92 35.37 32.71 31.04 30.16 29.69 29.19 29.07 26.63 25.59 22.21 21.10 19.31 18.86 15.60 14.85 13.00 10.19 8.97 8.32
180 170 160 150 140 130 120 110 100 90 80 70 60 50 40 30 20 ppm
Current Data Parameters
NAME MCX-95-C
EXPNO 1
PROCNO 1
F2 - Acquisition Parameters
Date_ 20190710
Time 11.26 h
INSTRUM spect
PROBHD Z152837_0001 (
PULPROG zgpg30
TD 65536
SOLVENT CDCl3
NS 1024
DS 4
SWH 42613.637 Hz
FIDRES 0.650232 Hz
AQ 0.7689557 sec
RG 187.62
DW 11.733 usec
DE 18.00 usec
TE 298.0 K
D1 2.00000000 sec
D11 0.03000000 sec
TD0 1
SFO1 176.0704628 MHz
NUC1 13C
P1 12.00 usec
PLW1 26.46899986 W
SFO2 700.1528006 MHz
NUC2 1H
CPDPRG[2 waltz16
PCPD2 65.00 usec
PLW2 10.69499969 W
PLW12 0.36452001 W
PLW13 0.18355000 W
F2 - Processing parameters
SI 32768
SF 176.0528575 MHz
WDW EM
SSB 0
LB 1.00 Hz
GB 0
PC 1.40

61d

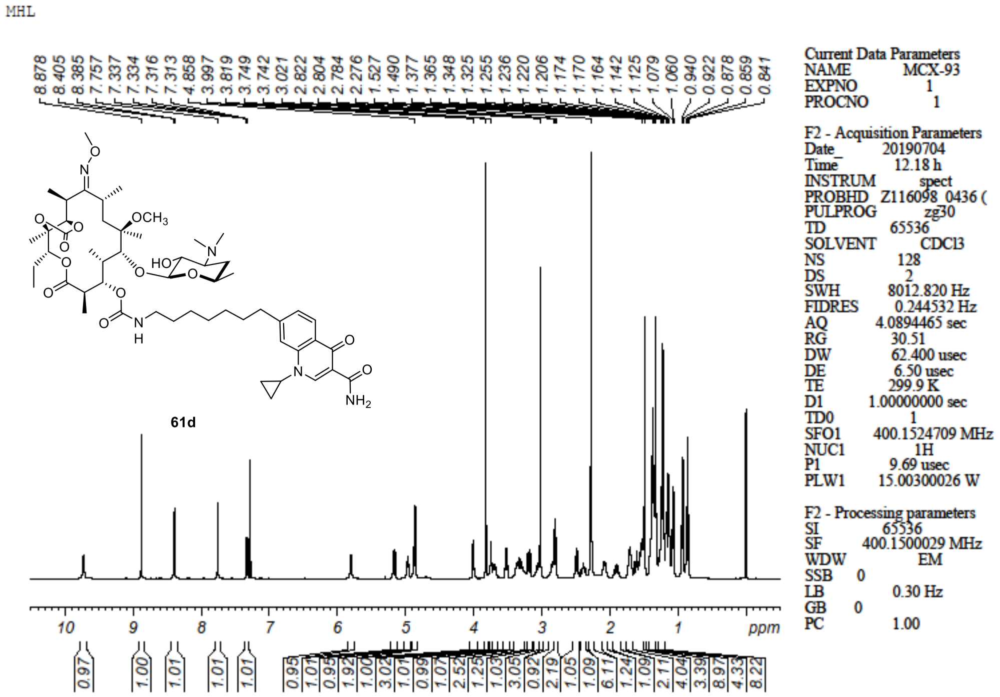
MHL
61d
Current Data Parameters
NAME MCX-93
EXPNO 1
PROCNO 1
F2 - Acquisition Parameters
Date_ 20190704
Time 12.18 h
INSTRUM spect
PROBHD Z116098_0436 (
PULPROG zg30
TD 65536
SOLVENT CDCl3
NS 128
DS 2
SWH 8012.820 Hz
FIDRES 0.244532 Hz
AQ 4.0894465 sec
RG 30.51
DW 62.400 usec
DE 6.50 usec
TE 299.9 K
D1 1.00000000 sec
TD0 1
SFO1 400.1524709 MHz
NUC1 1H
P1 9.69 usec
PLW1 15.00300026 W
F2 - Processing parameters
SI 65536
SF 400.1500029 MHz
WDW EM
SSB 0
LB 0.30 Hz
GB 0
PC 1.00
ppm

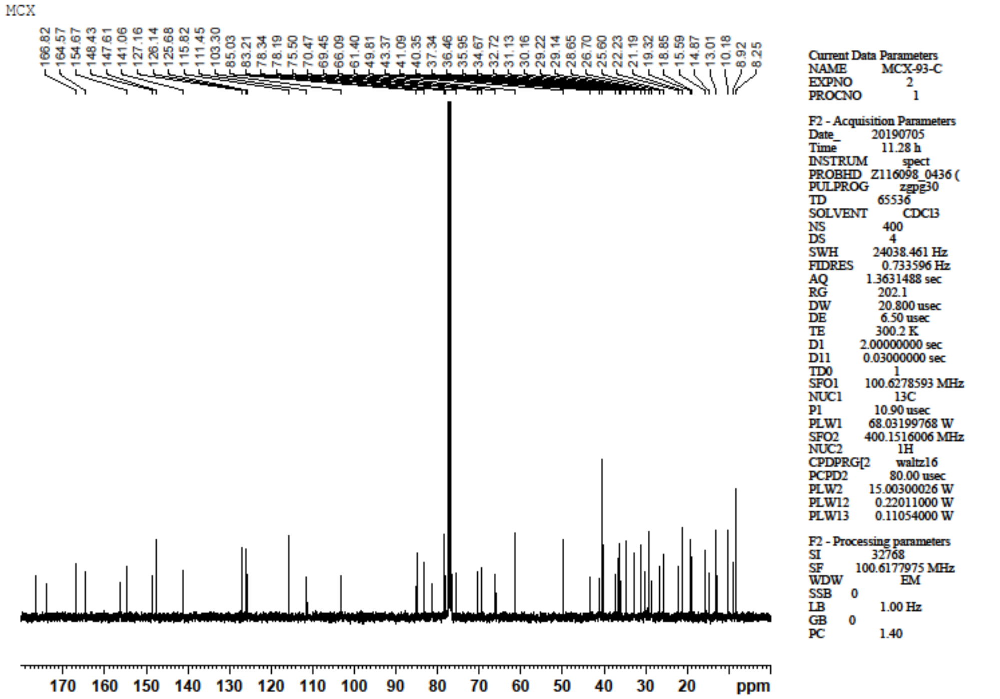
MCX
Current Data Parameters
NAME MCX-93-C
EXPNO 2
PROCNO 1
F2 - Acquisition Parameters
Date_ 20190705
Time 11.28 h
INSTRUM spect
PROBHD Z116098_0436 (
PULPROG zgpg30
TD 65536
SOLVENT CDCl3
NS 400
DS 4
SWH 24038.461 Hz
FIDRES 0.733596 Hz
AQ 1.3631488 sec
RG 202.1
DW 20.800 usec
DE 6.50 usec
TE 300.2 K
D1 2.00000000 sec
D11 0.03000000 sec
TD0 1
SFO1 100.6278593 MHz
NUC1 13C
P1 10.90 usec
PLW1 68.03199768 W
SFO2 400.1516006 MHz
NUC2 1H
CPDPRG[2 waltz16
PCPD2 80.00 usec
PLW2 15.00300026 W
PLW12 0.22011000 W
PLW13 0.11054000 W
F2 - Processing parameters
SI 32768
SF 100.6177975 MHz
WDW EM
SSB 0
LB 1.00 Hz
GB 0
PC 1.40
ppm

70c

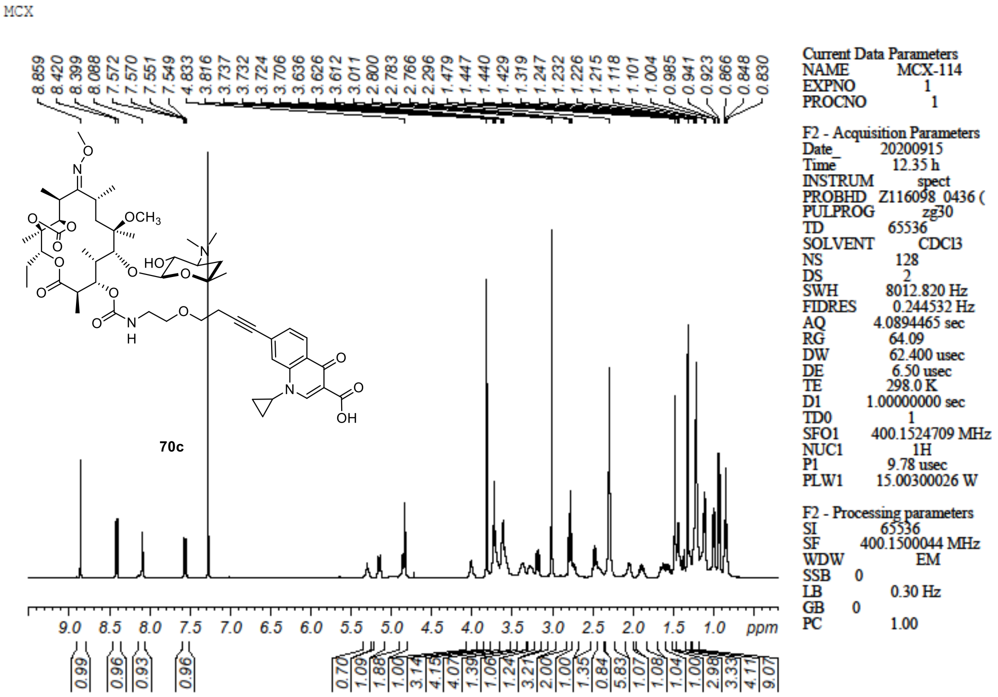
MCX
8.859 8.420 8.399 8.088 7.572 7.570 7.551 7.549 4.833 3.816 3.737 3.732 3.724 3.706 3.636 3.626 3.612 3.011 2.800 2.783 2.766 2.296 1.479 1.447 1.440 1.429 1.319 1.247 1.232 1.226 1.215 1.118 1.101 1.004 0.985 0.941 0.923 0.866 0.848 0.830
70c
9.0 8.5 8.0 7.5 7.0 6.5 6.0 5.5 5.0 4.5 4.0 3.5 3.0 2.5 2.0 1.5 1.0 ppm
0.99 0.96 0.93 0.96
0.70 1.09 1.88 1.00 3.14 4.15 4.07 1.39 1.06 1.24 3.21 2.00 1.00 1.35 0.84 5.83 1.07 1.08 1.04 1.00 2.98 3.33 4.11 9.07
Current Data Parameters
NAME MCX-114
EXPNO 1
PROCNO 1
F2 - Acquisition Parameters
Date_ 20200915
Time 12.35 h
INSTRUM spect
PROBHD Z116098_0436 (
PULPROG zg30
TD 65536
SOLVENT CDCl3
NS 128
DS 2
SWH 8012.820 Hz
FIDRES 0.244532 Hz
AQ 4.0894465 sec
RG 64.09
DW 62.400 usec
DE 6.50 usec
TE 298.0 K
D1 1.00000000 sec
TD0 1
SFO1 400.1524709 MHz
NUC1 1H
P1 9.78 usec
PLW1 15.00300026 W
F2 - Processing parameters
SI 65536
SF 400.1500044 MHz
WDW EM
SSB 0
LB 0.30 Hz
GB 0
PC 1.00

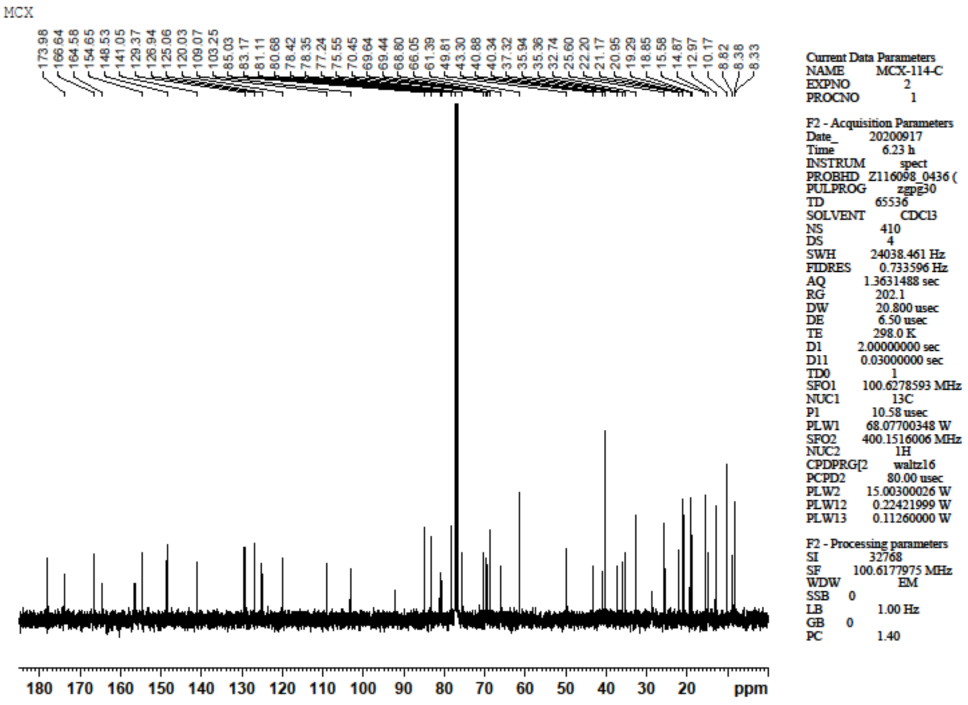
MCX
173.98 166.64 164.58 154.65 148.53 141.05 129.37 126.94 125.06 120.03 109.07 103.25 85.03 83.17 81.11 80.68 78.42 78.35 77.24 75.55 70.45 69.64 69.44 68.80 66.05 61.39 49.81 43.30 40.88 40.34 37.32 35.94 35.36 32.74 25.60 22.20 21.17 20.95 19.29 18.85 15.58 14.87 12.97 10.17 8.82 8.38 8.33
180 170 160 150 140 130 120 110 100 90 80 70 60 50 40 30 20 ppm
Current Data Parameters
NAME MCX-114-C
EXPNO 2
PROCNO 1
F2 - Acquisition Parameters
Date_ 20200917
Time 6.23 h
INSTRUM spect
PROBHD Z116098_0436 (
PULPROG zgpg30
TD 65536
SOLVENT CDCl3
NS 410
DS 4
SWH 24038.461 Hz
FIDRES 0.733596 Hz
AQ 1.3631488 sec
RG 202.1
DW 20.800 usec
DE 6.50 usec
TE 298.0 K
D1 2.00000000 sec
D11 0.03000000 sec
TD0 1
SFO1 100.6278593 MHz
NUC1 13C
P1 10.58 usec
PLW1 68.07700348 W
SFO2 400.1516006 MHz
NUC2 1H
CPDPRG[2 waltz16
PCPD2 80.00 usec
PLW2 15.00300026 W
PLW12 0.22421999 W
PLW13 0.11260000 W
F2 - Processing parameters
SI 32768
SF 100.6177975 MHz
WDW EM
SSB 0
LB 1.00 Hz
GB 0
PC 1.40

71c

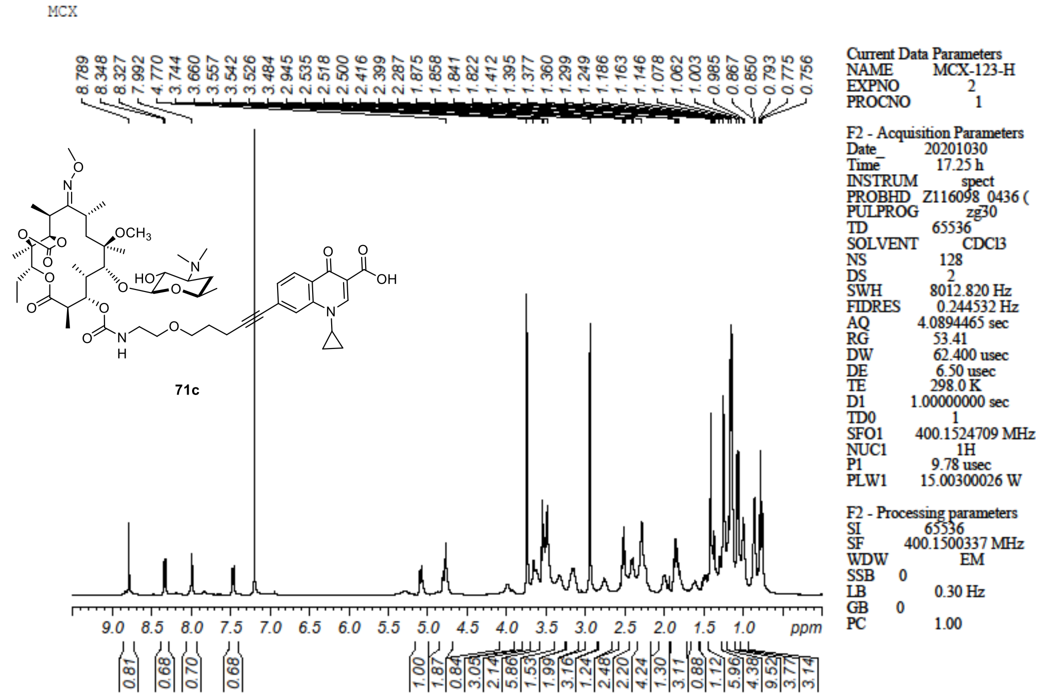
MCX
8.789 8.348 8.327 7.992 4.770 3.744 3.660 3.557 3.542 3.526 3.484 2.945 2.535 2.518 2.500 2.416 2.399 2.287 1.875 1.858 1.841 1.822 1.412 1.395 1.377 1.360 1.299 1.249 1.186 1.163 1.146 1.078 1.062 1.003 0.985 0.867 0.850 0.793 0.775 0.756
71c
Current Data Parameters
NAME MCX-123-H
EXPNO 2
PROCNO 1
F2 - Acquisition Parameters
Date_ 20201030
Time 17.25 h
INSTRUM spect
PROBHD Z116098_0436 (
PULPROG zg30
TD 65536
SOLVENT CDCl3
NS 128
DS 2
SWH 8012.820 Hz
FIDRES 0.244532 Hz
AQ 4.0894465 sec
RG 53.41
DW 62.400 usec
DE 6.50 usec
TE 298.0 K
D1 1.00000000 sec
TD0 1
SFO1 400.1524709 MHz
NUC1 1H
P1 9.78 usec
PLW1 15.00300026 W
F2 - Processing parameters
SI 65536
SF 400.1500337 MHz
WDW EM
SSB 0
LB 0.30 Hz
GB 0
PC 1.00
9.0 8.5 8.0 7.5 7.0 6.5 6.0 5.5 5.0 4.5 4.0 3.5 3.0 2.5 2.0 1.5 1.0 ppm
0.81 0.68 0.70 0.68 1.00 1.87 0.84 3.05 2.14 5.86 1.53 1.99 3.16 1.24 2.48 2.20 4.24 1.30 3.11 0.88 1.12 5.96 4.38 9.52 3.77 3.14

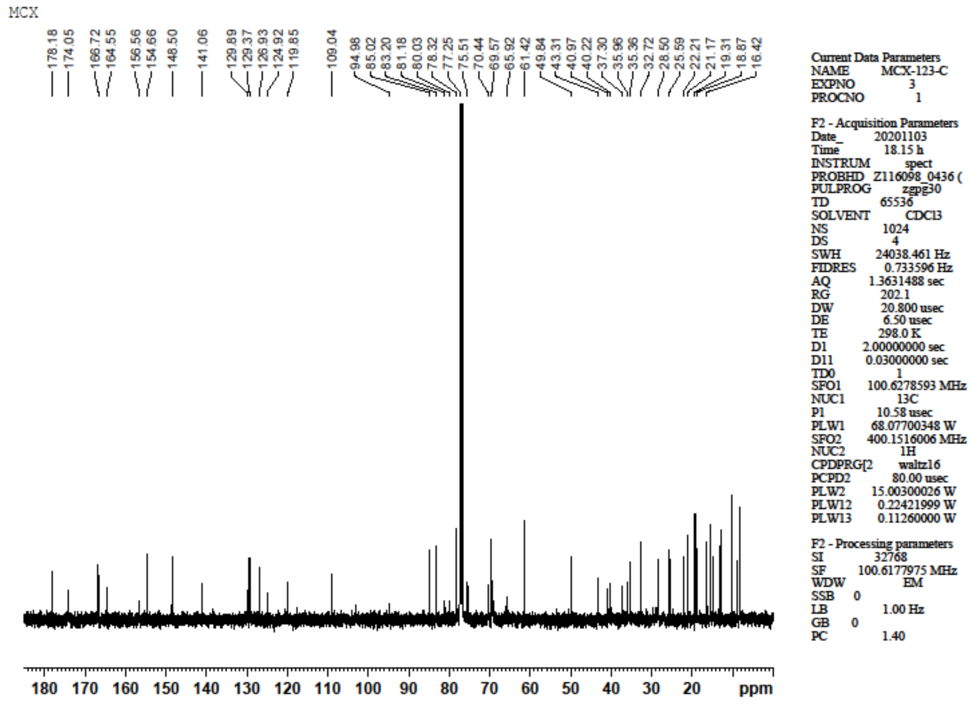
MCX
178.18 174.05 166.72 164.55 156.56 154.66 148.50 141.06 129.89 129.37 126.93 124.92 119.85 109.04 94.98 85.02 83.20 81.18 80.03 78.32 77.25 75.51 70.44 69.57 65.92 61.42 49.84 43.31 40.97 40.22 37.30 35.96 35.36 32.72 28.50 25.59 22.21 21.17 19.31 18.87 16.42
Current Data Parameters
NAME MCX-123-C
EXPNO 3
PROCNO 1
F2 - Acquisition Parameters
Date_ 20201103
Time 18.15 h
INSTRUM spect
PROBHD Z116098_0436 (
PULPROG zgpg30
TD 65536
SOLVENT CDCl3
NS 1024
DS 4
SWH 24038.461 Hz
FIDRES 0.733596 Hz
AQ 1.3631488 sec
RG 202.1
DW 20.800 usec
DE 6.50 usec
TE 298.0 K
D1 2.00000000 sec
D11 0.03000000 sec
TD0 1
SFO1 100.6278593 MHz
NUC1 13C
P1 10.58 usec
PLW1 68.07700348 W
SFO2 400.1516006 MHz
NUC2 1H
CPDPRG[2 waltz16
PCPD2 80.00 usec
PLW2 15.00300026 W
PLW12 0.22421999 W
PLW13 0.11260000 W
F2 - Processing parameters
SI 32768
SF 100.6177975 MHz
WDW EM
SSB 0
LB 1.00 Hz
GB 0
PC 1.40
180 170 160 150 140 130 120 110 100 90 80 70 60 50 40 30 20 ppm

72c

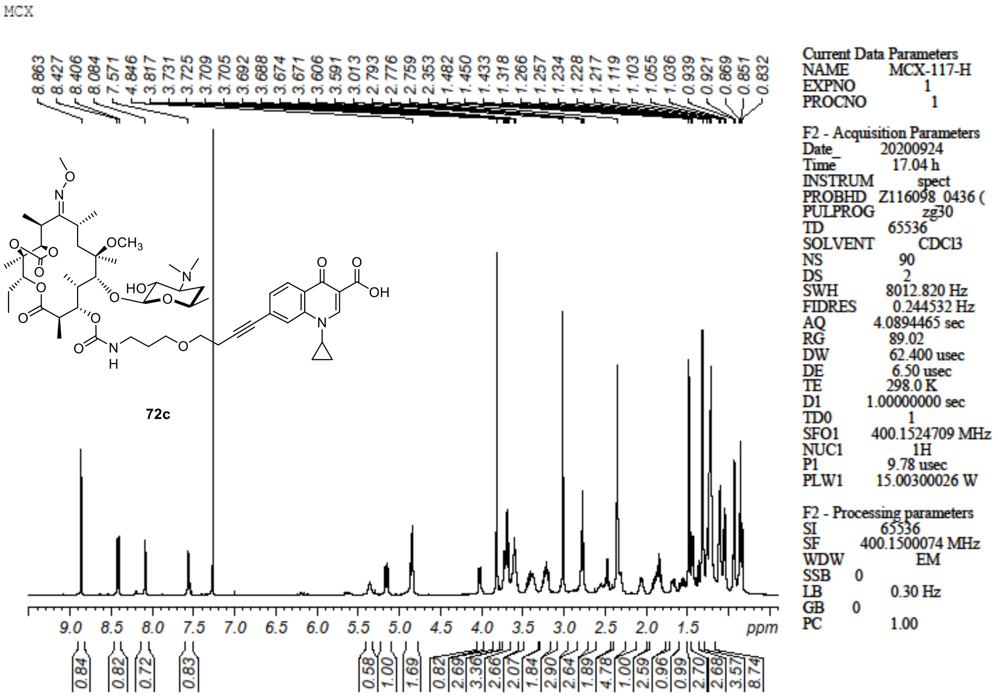
MCX
8.863
8.427
8.406
8.084
7.571
4.846
3.817
3.731
3.725
3.709
3.705
3.692
3.688
3.674
3.671
3.606
3.591
3.013
2.793
2.776
2.759
2.353
1.482
1.450
1.433
1.318
1.266
1.257
1.234
1.228
1.217
1.119
1.103
1.055
1.036
0.939
0.921
0.869
0.851
0.832
72c
Current Data Parameters
NAME MCX-117-H
EXPNO 1
PROCNO 1
F2 - Acquisition Parameters
Date_ 20200924
Time 17.04 h
INSTRUM spect
PROBHD Z116098_0436 (
PULPROG zg30
TD 65536
SOLVENT CDCl3
NS 90
DS 2
SWH 8012.820 Hz
FIDRES 0.244532 Hz
AQ 4.0894465 sec
RG 89.02
DW 62.400 usec
DE 6.50 usec
TE 298.0 K
D1 1.00000000 sec
TD0 1
SFO1 400.1524709 MHz
NUC1 1H
P1 9.78 usec
PLW1 15.00300026 W
F2 - Processing parameters
SI 65536
SF 400.1500074 MHz
WDW EM
SSB 0
LB 0.30 Hz
GB 0
PC 1.00
9.0 8.5 8.0 7.5 7.0 6.5 6.0 5.5 5.0 4.5 4.0 3.5 3.0 2.5 2.0 1.5 ppm

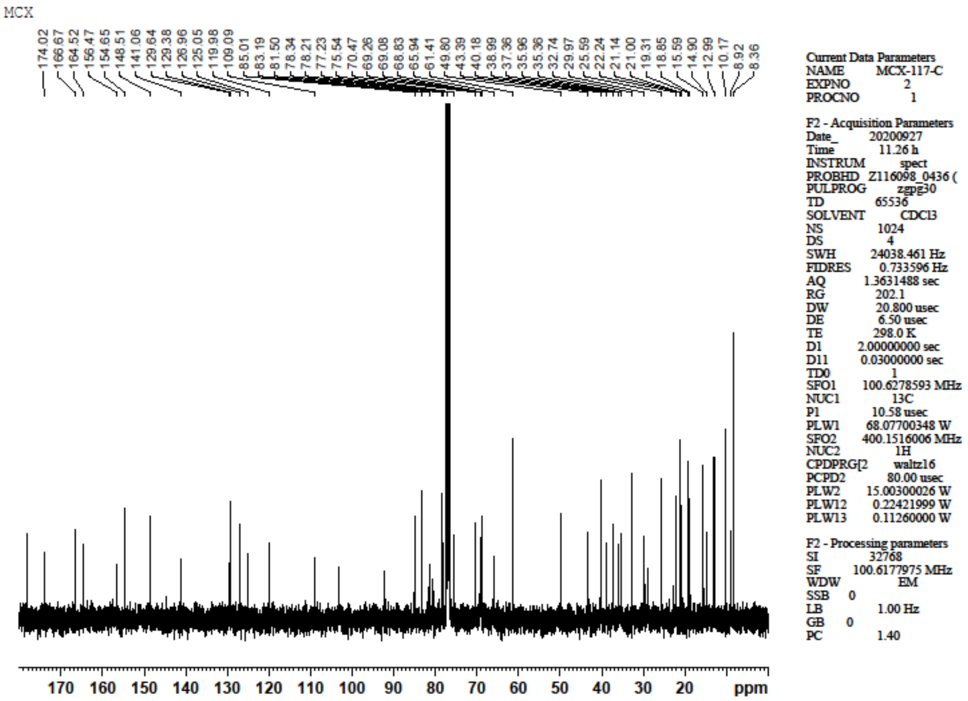
MCX
174.02
166.67
164.52
156.47
154.65
148.51
141.06
129.64
129.38
126.96
125.05
119.98
109.09
85.01
83.19
81.50
78.34
78.21
77.23
75.54
70.47
69.26
69.08
68.83
65.94
61.41
49.80
43.39
40.18
38.99
37.36
35.96
35.36
32.74
29.97
25.59
22.24
21.14
21.00
19.31
18.85
15.59
14.90
12.99
10.17
8.92
8.36
Current Data Parameters
NAME MCX-117-C
EXPNO 2
PROCNO 1
F2 - Acquisition Parameters
Date_ 20200927
Time 11.26 h
INSTRUM spect
PROBHD Z116098_0436 (
PULPROG zgpg30
TD 65536
SOLVENT CDCl3
NS 1024
DS 4
SWH 24038.461 Hz
FIDRES 0.733596 Hz
AQ 1.3631488 sec
RG 202.1
DW 20.800 usec
DE 6.50 usec
TE 298.0 K
D1 2.00000000 sec
D11 0.03000000 sec
TD0 1
SFO1 100.6278593 MHz
NUC1 13C
P1 10.58 usec
PLW1 68.07700348 W
SFO2 400.1516006 MHz
NUC2 1H
CPDPRG[2 waltz16
PCPD2 80.00 usec
PLW2 15.00300026 W
PLW12 0.22421999 W
PLW13 0.11260000 W
F2 - Processing parameters
SI 32768
SF 100.6177975 MHz
WDW EM
SSB 0
LB 1.00 Hz
GB 0
PC 1.40
170 160 150 140 130 120 110 100 90 80 70 60 50 40 30 20 ppm

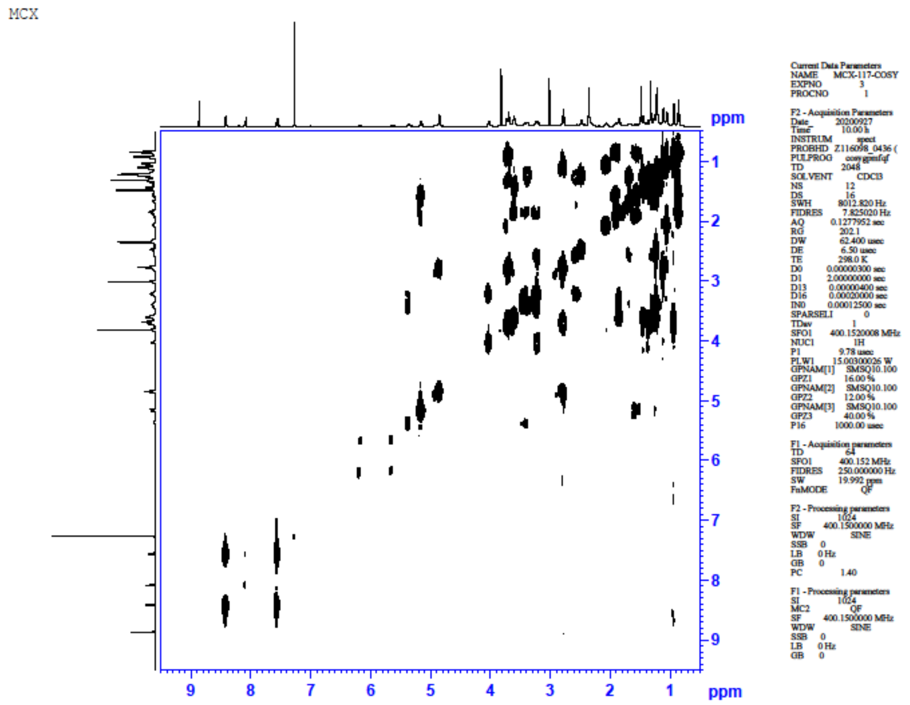

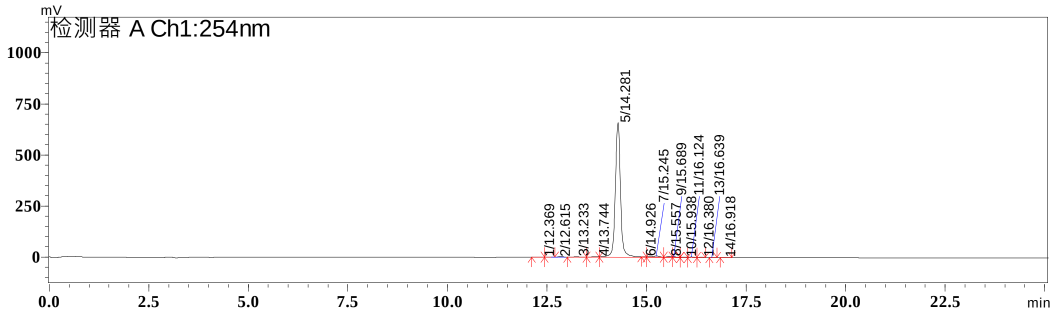

Retention	Peak start	Peak End	peak area	peak height	Area %
12.369	12.117	12.442	4665	459	0.0733
12.615	12.442	12.700	4064	311	0.0639
13.233	13.017	13.492	29047	2778	0.4565
13.744	13.492	13.817	40476	3414	0.6361
14.281	13.817	15.850	6138529	659053	96.4651
14.926	14.867	15.000	1816	345	0.0285
15.245	15.000	15.433	62522	5553	0.9825
15.557	15.433	15.650	28881	4144	0.4539
15.689	15.650	15.842	12813	2145	0.2013
15.938	15.850	16.033	7706	1408	0.1211
16.124	16.033	16.267	15973	2587	0.2510
16.380	16.267	16.483	10658	1741	0.1675
16.639	16.575	16.767	4024	760	0.0632
16.918	16.850	17.142	2295	217	0.0361

74c

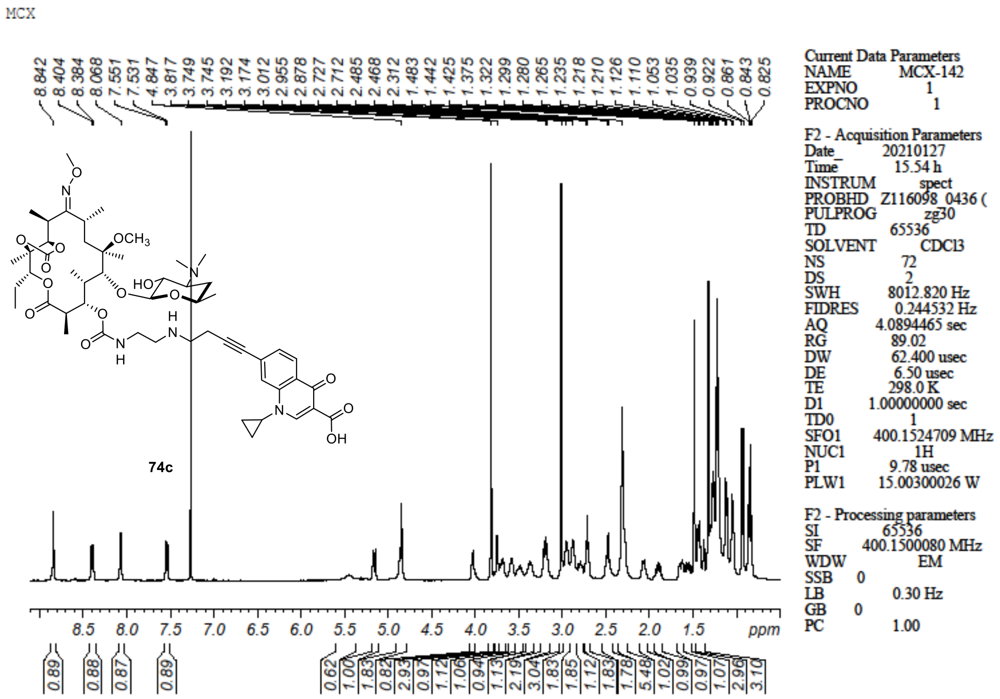
MCX
8.842
8.404
8.384
8.068
7.551
7.531
4.847
3.817
3.749
3.745
3.192
3.174
3.012
2.955
2.878
2.727
2.712
2.485
2.468
2.312
1.483
1.442
1.425
1.375
1.322
1.299
1.280
1.265
1.235
1.218
1.210
1.126
1.110
1.053
1.035
0.939
0.922
0.861
0.843
0.825
OCH3
HO
OH
74c
8.5 8.0 7.5 7.0 6.5 6.0 5.5 5.0 4.5 4.0 3.5 3.0 2.5 2.0 1.5 ppm
0.89 0.88 0.87 0.89 0.62 1.00 1.83 0.82 2.93 0.97 1.12 1.06 0.94 1.13 2.19 3.04 1.83 1.85 1.12 1.83 1.78 5.48 1.02 0.99 0.97 1.07 2.96 3.10
Current Data Parameters
NAME MCX-142
EXPNO 1
PROCNO 1
F2 - Acquisition Parameters
Date_ 20210127
Time 15.54 h
INSTRUM spect
PROBHD Z116098_0436 (
PULPROG zg30
TD 65536
SOLVENT CDCl3
NS 72
DS 2
SWH 8012.820 Hz
FIDRES 0.244532 Hz
AQ 4.0894465 sec
RG 89.02
DW 62.400 usec
DE 6.50 usec
TE 298.0 K
D1 1.00000000 sec
TD0 1
SFO1 400.1524709 MHz
NUC1 1H
P1 9.78 usec
PLW1 15.00300026 W
F2 - Processing parameters
SI 65536
SF 400.1500080 MHz
WDW EM
SSB 0
LB 0.30 Hz
GB 0
PC 1.00

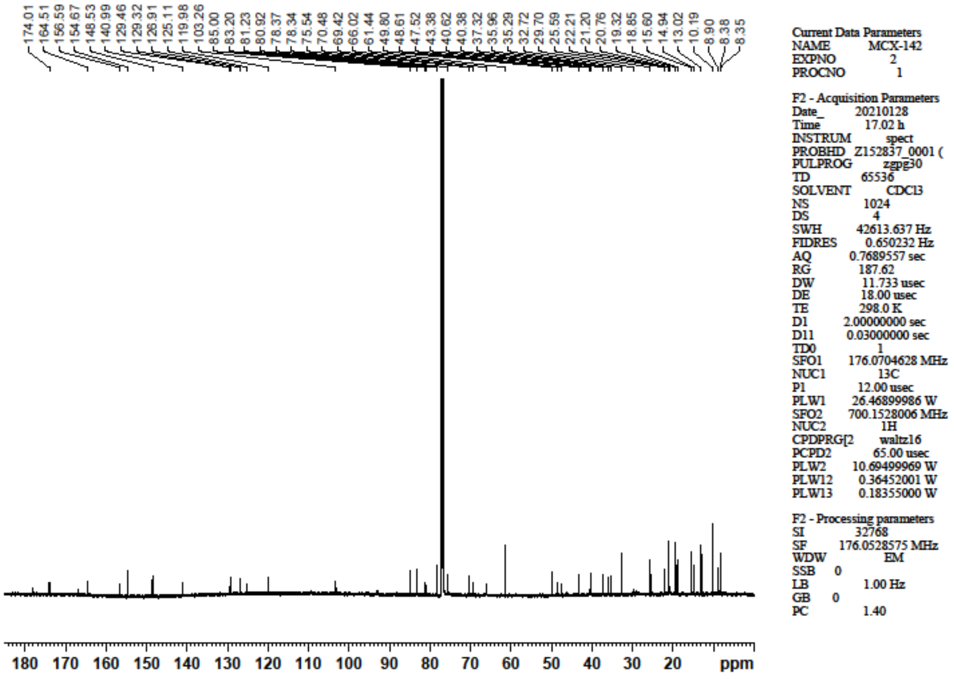
174.01
164.51
156.59
154.67
148.53
140.99
129.46
129.32
126.91
125.11
119.98
103.26
85.00
83.20
81.23
80.92
78.37
78.34
75.54
70.48
69.42
66.02
61.44
49.80
48.61
47.52
43.38
40.62
40.38
37.32
35.96
35.29
32.72
29.70
25.59
22.21
21.20
20.76
19.32
18.85
15.60
14.94
13.02
10.19
8.90
8.38
8.35
180 170 160 150 140 130 120 110 100 90 80 70 60 50 40 30 20 ppm
Current Data Parameters
NAME MCX-142
EXPNO 2
PROCNO 1
F2 - Acquisition Parameters
Date_ 20210128
Time 17.02 h
INSTRUM spect
PROBHD Z152837_0001 (
PULPROG zgpg30
TD 65536
SOLVENT CDCl3
NS 1024
DS 4
SWH 42613.637 Hz
FIDRES 0.650232 Hz
AQ 0.7689557 sec
RG 187.62
DW 11.733 usec
DE 18.00 usec
TE 298.0 K
D1 2.00000000 sec
D11 0.03000000 sec
TD0 1
SFO1 176.0704628 MHz
NUC1 13C
P1 12.00 usec
PLW1 26.46899986 W
SFO2 700.1528006 MHz
NUC2 1H
CPDPRG[2 waltz16
PCPD2 65.00 usec
PLW2 10.69499969 W
PLW12 0.36452001 W
PLW13 0.18355000 W
F2 - Processing parameters
SI 32768
SF 176.0528575 MHz
WDW EM
SSB 0
LB 1.00 Hz
GB 0
PC 1.40

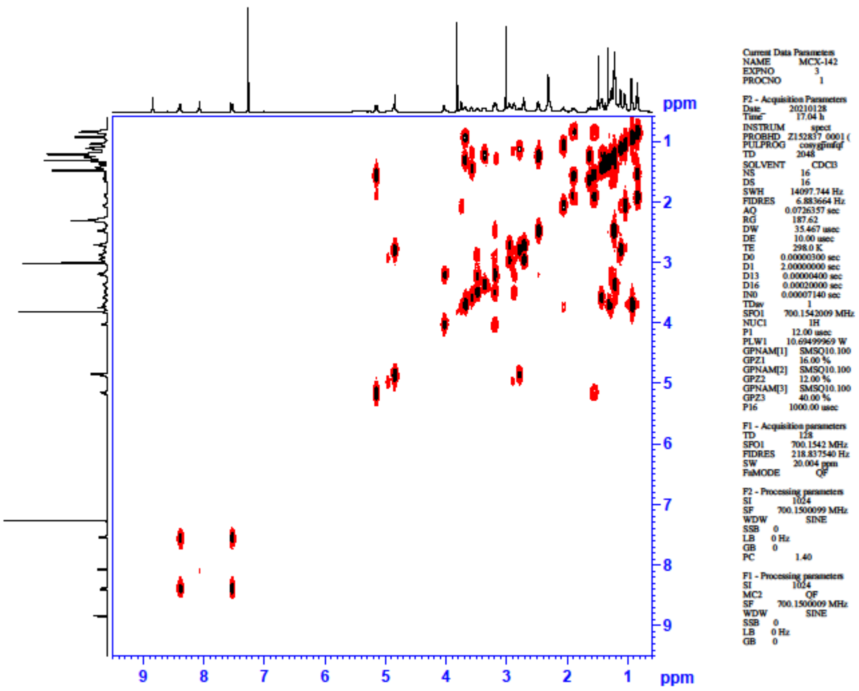
ppm
1
2
3
4
5
6
7
8
9
9
8
7
6
5
4
3
2
1
ppm
Current Data Parameters
NAME MCX-142
EXPNO 3
PROCNO 1
F2 - Acquisition Parameters
Date_ 20210128
Time 17.04 h
INSTRUM spect
PROBHD Z152837_0001 (
PULPROG cosygpmfqf
TD 2048
SOLVENT CDCl3
NS 16
DS 16
SWH 14097.744 Hz
FIDRES 6.883664 Hz
AQ 0.0726357 sec
RG 187.62
DW 35.467 usec
DE 10.00 usec
TE 298.0 K
D0 0.00000300 sec
D1 2.00000000 sec
D13 0.00000400 sec
D16 0.00020000 sec
IN0 0.00007140 sec
TDav 1
SFO1 700.1542009 MHz
NUC1 1H
P1 12.00 usec
PLW1 10.69499969 W
GPNAM[1] SMSQ10.100
GPZ1 16.00 %
GPNAM[2] SMSQ10.100
GPZ2 12.00 %
GPNAM[3] SMSQ10.100
GPZ3 40.00 %
P16 1000.00 usec
F1 - Acquisition parameters
TD 128
SFO1 700.1542 MHz
FIDRES 218.837540 Hz
SW 20.004 ppm
FnMODE QF
F2 - Processing parameters
SI 1024
SF 700.1500099 MHz
WDW SINE
SSB 0
LB 0 Hz
GB 0
PC 1.40
F1 - Processing parameters
SI 1024
MC2 QF
SF 700.1500009 MHz
WDW SINE
SSB 0
LB 0 Hz
GB 0

79c

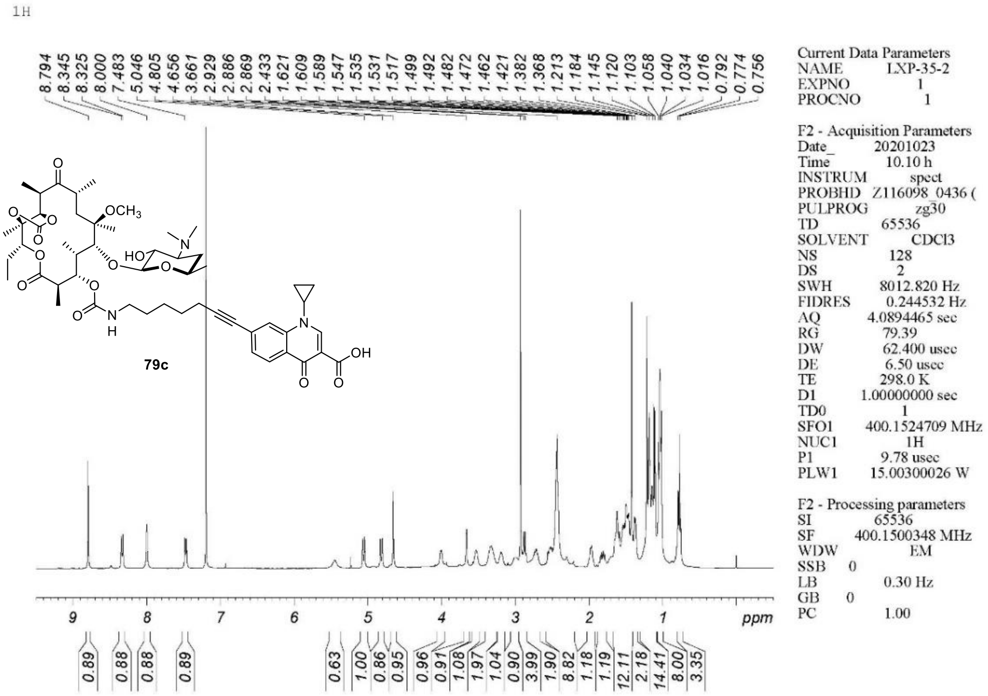
1H
8.794
8.345
8.325
8.000
7.483
5.046
4.805
4.656
3.661
2.929
2.886
2.869
2.433
1.621
1.609
1.589
1.547
1.535
1.531
1.517
1.499
1.492
1.482
1.472
1.462
1.421
1.382
1.368
1.213
1.184
1.145
1.120
1.103
1.058
1.040
1.034
1.016
0.792
0.774
0.756
79c
ppm
Current Data Parameters
NAME LXP-35-2
EXPNO 1
PROCNO 1
F2 - Acquisition Parameters
Date_ 20201023
Time 10.10 h
INSTRUM spect
PROBHD Z116098_0436 (
PULPROG zg30
TD 65536
SOLVENT CDCl3
NS 128
DS 2
SWH 8012.820 Hz
FIDRES 0.244532 Hz
AQ 4.0894465 sec
RG 79.39
DW 62.400 usec
DE 6.50 usec
TE 298.0 K
D1 1.00000000 sec
TD0 1
SFO1 400.1524709 MHz
NUC1 1H
P1 9.78 usec
PLW1 15.00300026 W
F2 - Processing parameters
SI 65536
SF 400.1500348 MHz
WDW EM
SSB 0
LB 0.30 Hz
GB 0
PC 1.00

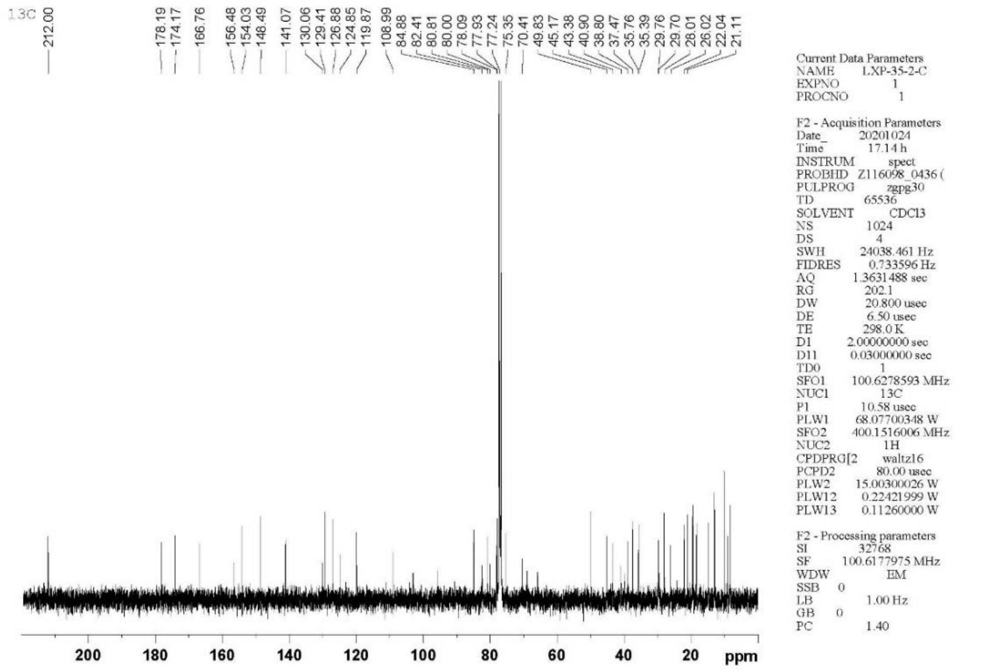
13C
212.00
178.19
174.17
166.76
156.48
154.03
148.49
141.07
130.06
129.41
126.88
124.85
119.87
108.99
84.88
82.41
80.81
80.00
78.09
77.93
77.24
75.35
70.41
49.83
45.17
43.38
40.90
38.80
37.47
35.76
35.39
29.76
29.70
28.01
26.02
22.04
21.11
ppm
Current Data Parameters
NAME LXP-35-2-C
EXPNO 1
PROCNO 1
F2 - Acquisition Parameters
Date_ 20201024
Time 17.14 h
INSTRUM spect
PROBHD Z116098_0436 (
PULPROG zgpg30
TD 65536
SOLVENT CDCl3
NS 1024
DS 4
SWH 24038.461 Hz
FIDRES 0.733596 Hz
AQ 1.3631488 sec
RG 202.1
DW 20.800 usec
DE 6.50 usec
TE 298.0 K
D1 2.00000000 sec
D11 0.03000000 sec
TD0 1
SFO1 100.6278593 MHz
NUC1 13C
P1 10.58 usec
PLW1 68.07700348 W
SFO2 400.1516006 MHz
NUC2 1H
CPDPRG[2 waltz16
PCPD2 80.00 usec
PLW2 15.00300026 W
PLW12 0.22421999 W
PLW13 0.11260000 W
F2 - Processing parameters
SI 32768
SF 100.6177975 MHz
WDW EM
SSB 0
LB 1.00 Hz
GB 0
PC 1.40

87c

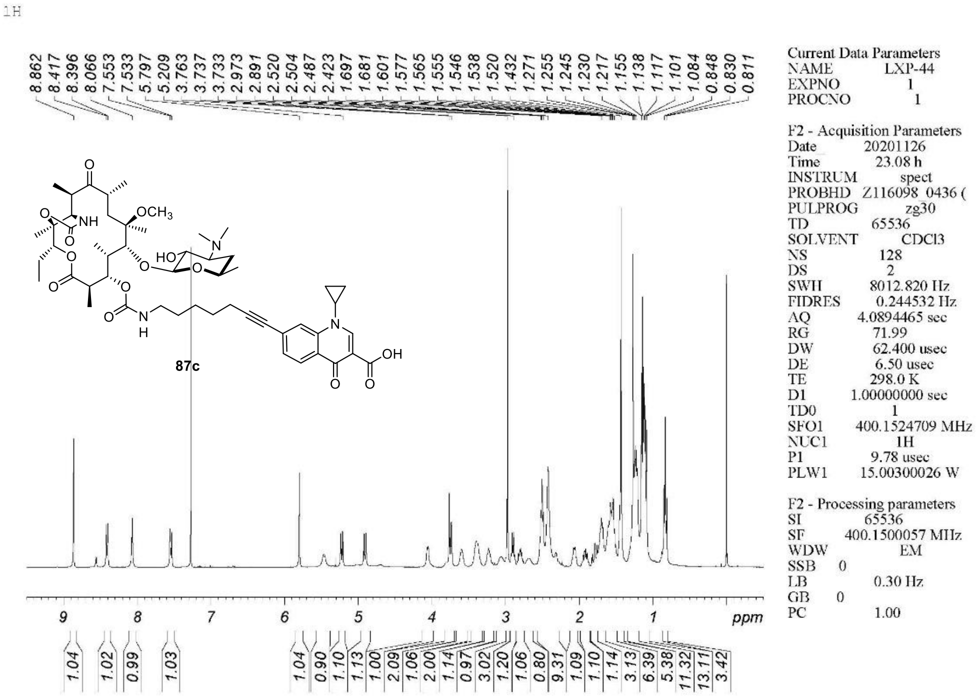
1H
8.862 8.417 8.396 8.066 7.553 7.533 5.797 5.209 3.763 3.737 3.733 2.973 2.891 2.520 2.504 2.487 2.423 1.697 1.681 1.601 1.577 1.565 1.555 1.546 1.538 1.520 1.432 1.271 1.255 1.245 1.230 1.217 1.155 1.138 1.117 1.101 1.084 0.848 0.830 0.811
87c
OCH3
NH
HO
N
O
OH
H
9 8 7 6 5 4 3 2 1 ppm
1.04 1.02 0.99 1.03 1.04 0.90 1.10 1.13 1.00 2.09 1.06 2.00 1.14 0.97 3.02 1.20 1.06 0.80 9.31 1.09 1.10 1.14 3.13 6.39 5.38 11.32 13.11 3.42
Current Data Parameters
NAME LXP-44
EXPNO 1
PROCNO 1
F2 - Acquisition Parameters
Date 20201126
Time 23.08 h
INSTRUM spect
PROBHD Z116098_0436 (
PULPROG zg30
TD 65536
SOLVENT CDCl3
NS 128
DS 2
SWH 8012.820 Hz
FIDRES 0.244532 Hz
AQ 4.0894465 sec
RG 71.99
DW 62.400 usec
DE 6.50 usec
TE 298.0 K
D1 1.00000000 sec
TD0 1
SFO1 400.1524709 MHz
NUC1 1H
P1 9.78 usec
PLW1 15.00300026 W
F2 - Processing parameters
SI 65536
SF 400.1500057 MHz
WDW EM
SSB 0
LB 0.30 Hz
GB 0
PC 1.00

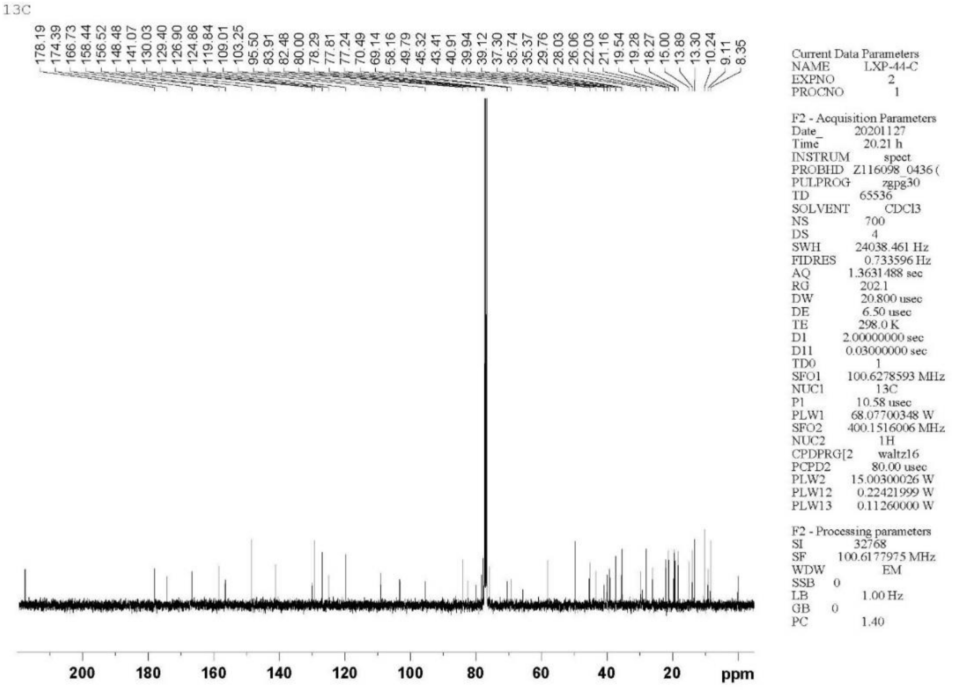
13C
178.19 174.39 166.73 158.44 156.52 148.48 141.07 130.03 129.40 126.90 124.86 119.84 109.01 103.25 95.50 83.91 82.48 80.00 78.29 77.81 77.24 70.49 69.14 58.16 49.79 45.32 43.41 40.91 39.94 39.12 37.30 35.74 35.37 29.76 28.03 26.06 22.03 21.16 19.54 19.28 18.27 15.00 13.89 13.30 10.24 9.11 8.35
200 180 160 140 120 100 80 60 40 20 ppm
Current Data Parameters
NAME LXP-44-C
EXPNO 2
PROCNO 1
F2 - Acquisition Parameters
Date_ 20201127
Time 20.21 h
INSTRUM spect
PROBHD Z116098_0436 (
PULPROG zgpg30
TD 65536
SOLVENT CDCl3
NS 700
DS 4
SWH 24038.461 Hz
FIDRES 0.733596 Hz
AQ 1.3631488 sec
RG 202.1
DW 20.800 usec
DE 6.50 usec
TE 298.0 K
D1 2.00000000 sec
D11 0.03000000 sec
TD0 1
SFO1 100.6278593 MHz
NUC1 13C
P1 10.58 usec
PLW1 68.07700348 W
SFO2 400.1516006 MHz
NUC2 1H
CPDPRG[2 waltz16
PCPD2 80.00 usec
PLW2 15.00300026 W
PLW12 0.22421999 W
PLW13 0.11260000 W
F2 - Processing parameters
SI 32768
SF 100.6177975 MHz
WDW EM
SSB 0
LB 1.00 Hz
GB 0
PC 1.40

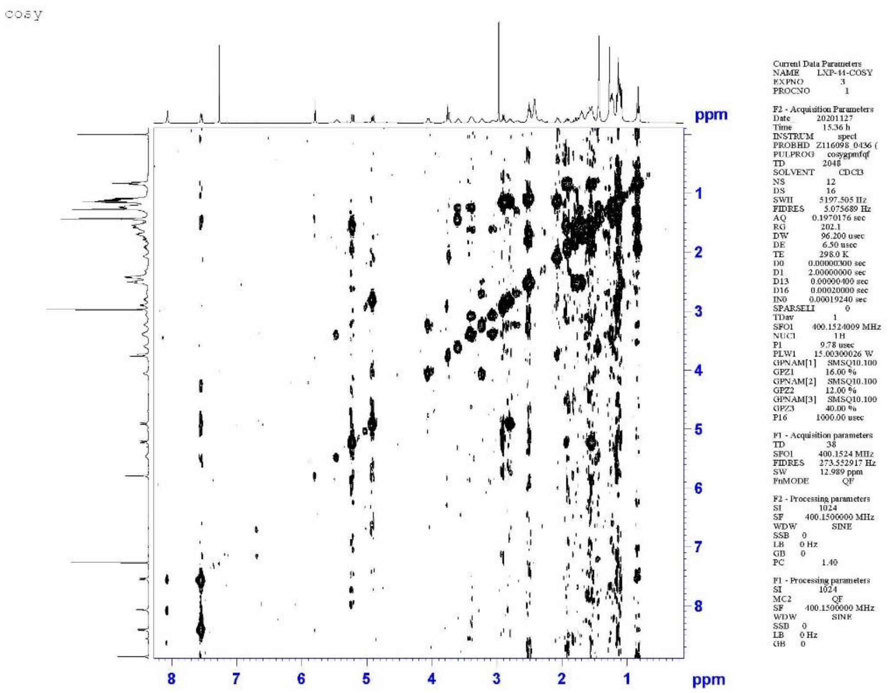
cosy
ppm
1
2
3
4
5
6
7
8
8 7 6 5 4 3 2 1 ppm
Current Data Parameters
NAME LXP-44-COSY
EXPNO 3
PROCNO 1
F2 - Acquisition Parameters
Date_ 20201127
Time 15.36 h
INSTRUM spect
PROBHD Z116098_0436 (
PULPROG cosygpmfqf
TD 2048
SOLVENT CDCl3
NS 12
DS 16
SWH 5197.505 Hz
FIDRES 5.075689 Hz
AQ 0.1970176 sec
RG 202.1
DW 96.200 usec
DE 6.50 usec
TE 298.0 K
D0 0.00000300 sec
D1 2.00000000 sec
D13 0.00000400 sec
D16 0.00020000 sec
IN0 0.00019240 sec
SPARSELI 0
TDav 1
SFO1 400.1524009 MHz
NUC1 1H
P1 9.78 usec
PLW1 15.00300026 W
GPNAM[1] SMSQ10.100
GPZ1 16.00 %
GPNAM[2] SMSQ10.100
GPZ2 12.00 %
GPNAM[3] SMSQ10.100
GPZ3 40.00 %
P16 1000.00 usec
F1 - Acquisition parameters
TD 38
SFO1 400.1524 MHz
FIDRES 273.552917 Hz
SW 12.989 ppm
FnMODE QF
F2 - Processing parameters
SI 1024
SF 400.1500000 MHz
WDW SINE
SSB 0
LB 0 Hz
GB 0
PC 1.40
F1 - Processing parameters
SI 1024
MC2 QF
SF 400.1500000 MHz
WDW SINE
SSB 0
LB 0 Hz
GB 0

88c

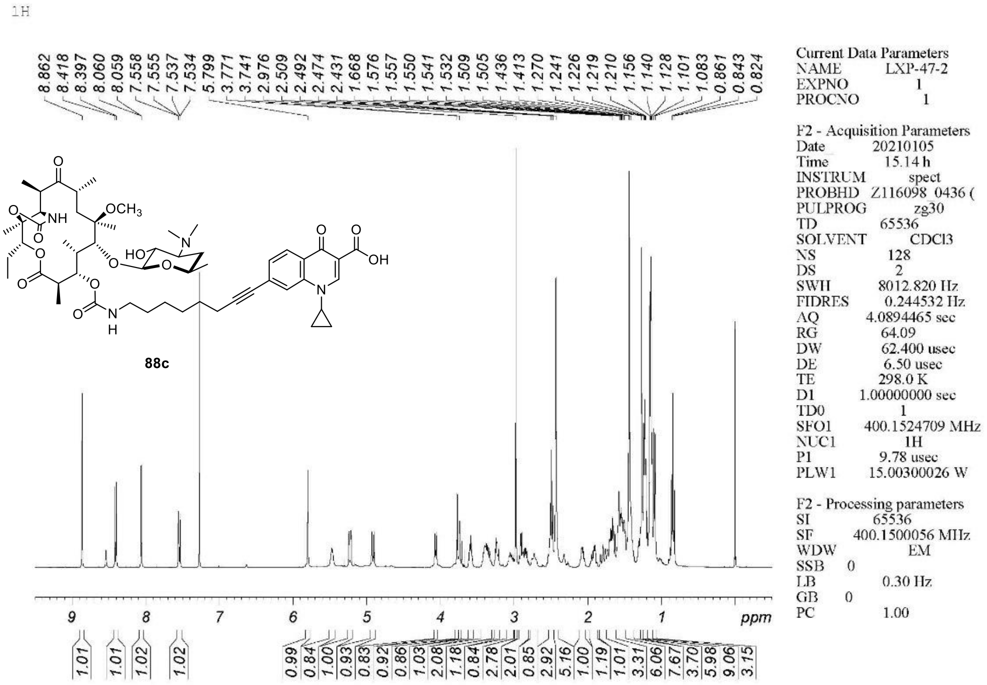
1H
8.862 8.418 8.397 8.060 8.059 7.558 7.555 7.537 7.534 5.799 3.771 3.741 2.976 2.509 2.492 2.474 2.431 1.668 1.576 1.557 1.550 1.541 1.532 1.509 1.505 1.436 1.413 1.270 1.241 1.226 1.219 1.210 1.156 1.140 1.128 1.101 1.083 0.861 0.843 0.824
88c
Current Data Parameters
NAME LXP-47-2
EXPNO 1
PROCNO 1
F2 - Acquisition Parameters
Date_ 20210105
Time 15.14 h
INSTRUM spect
PROBHD Z116098_0436 (
PULPROG zg30
TD 65536
SOLVENT CDCl3
NS 128
DS 2
SWH 8012.820 Hz
FIDRES 0.244532 Hz
AQ 4.0894465 sec
RG 64.09
DW 62.400 usec
DE 6.50 usec
TE 298.0 K
D1 1.00000000 sec
TD0 1
SFO1 400.1524709 MHz
NUC1 1H
P1 9.78 usec
PLW1 15.00300026 W
F2 - Processing parameters
SI 65536
SF 400.1500056 MHz
WDW EM
SSB 0
LB 0.30 Hz
GB 0
PC 1.00
9 8 7 6 5 4 3 2 1 ppm
1.01 1.01 1.02 1.02 0.99 0.84 1.00 0.93 0.83 0.92 0.86 1.03 2.08 1.18 0.84 2.78 2.01 0.85 2.92 5.16 1.00 1.19 1.01 3.31 6.06 7.67 3.70 5.98 9.06 3.15

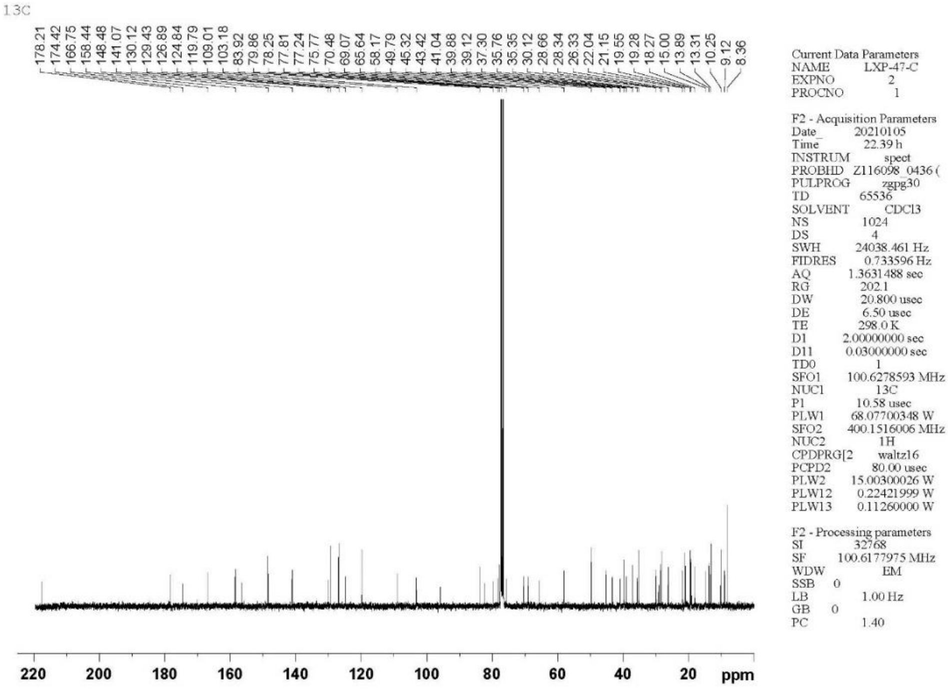
13C
178.21 174.42 166.75 158.44 148.48 141.07 130.12 129.43 126.89 124.84 119.79 109.01 103.18 83.92 79.86 78.25 77.81 77.24 75.77 70.48 69.07 65.64 58.17 49.79 45.32 43.42 41.04 39.88 39.12 37.30 35.76 35.35 30.12 28.66 28.34 26.33 22.04 21.15 19.55 19.28 18.27 15.00 13.89 13.31 10.25 9.12 8.36
Current Data Parameters
NAME LXP-47-C
EXPNO 2
PROCNO 1
F2 - Acquisition Parameters
Date_ 20210105
Time 22.39 h
INSTRUM spect
PROBHD Z116098_0436 (
PULPROG zgpg30
TD 65536
SOLVENT CDCl3
NS 1024
DS 4
SWH 24038.461 Hz
FIDRES 0.733596 Hz
AQ 1.3631488 sec
RG 202.1
DW 20.800 usec
DE 6.50 usec
TE 298.0 K
D1 2.00000000 sec
D11 0.03000000 sec
TD0 1
SFO1 100.6278593 MHz
NUC1 13C
P1 10.58 usec
PLW1 68.07700348 W
SFO2 400.1516006 MHz
NUC2 1H
CPDPRG[2 waltz16
PCPD2 80.00 usec
PLW2 15.00300026 W
PLW12 0.22421999 W
PLW13 0.11260000 W
F2 - Processing parameters
SI 32768
SF 100.6177975 MHz
WDW EM
SSB 0
LB 1.00 Hz
GB 0
PC 1.40
220 200 180 160 140 120 100 80 60 40 20 ppm

88f

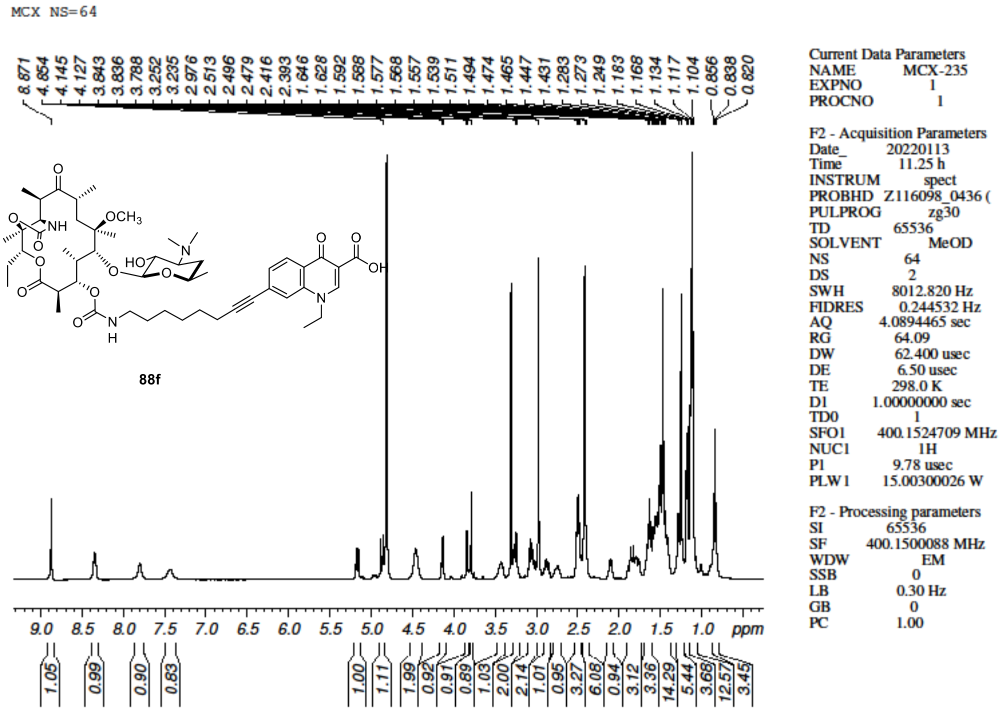
MCX NS=64
8.871
4.854
4.145
4.127
3.843
3.836
3.788
3.252
3.235
2.976
2.513
2.496
2.479
2.416
2.393
1.646
1.628
1.592
1.588
1.577
1.568
1.557
1.539
1.511
1.494
1.474
1.465
1.447
1.431
1.283
1.273
1.249
1.183
1.168
1.134
1.117
1.104
0.856
0.838
0.820
88f
Current Data Parameters
NAME MCX-235
EXPNO 1
PROCNO 1
F2 - Acquisition Parameters
Date_ 20220113
Time 11.25 h
INSTRUM spect
PROBHD Z116098_0436 (
PULPROG zg30
TD 65536
SOLVENT MeOD
NS 64
DS 2
SWH 8012.820 Hz
FIDRES 0.244532 Hz
AQ 4.0894465 sec
RG 64.09
DW 62.400 usec
DE 6.50 usec
TE 298.0 K
D1 1.00000000 sec
TD0 1
SFO1 400.1524709 MHz
NUC1 1H
P1 9.78 usec
PLW1 15.00300026 W
F2 - Processing parameters
SI 65536
SF 400.1500088 MHz
WDW EM
SSB 0
LB 0.30 Hz
GB 0
PC 1.00
9.0
8.5
8.0
7.5
7.0
6.5
6.0
5.5
5.0
4.5
4.0
3.5
3.0
2.5
2.0
1.5
1.0
ppm
1.05
0.99
0.90
0.83
1.00
1.11
1.99
0.92
0.91
0.89
1.03
2.00
2.14
1.01
0.95
3.27
6.08
0.94
3.12
3.36
14.29
5.44
3.68
12.57
3.45

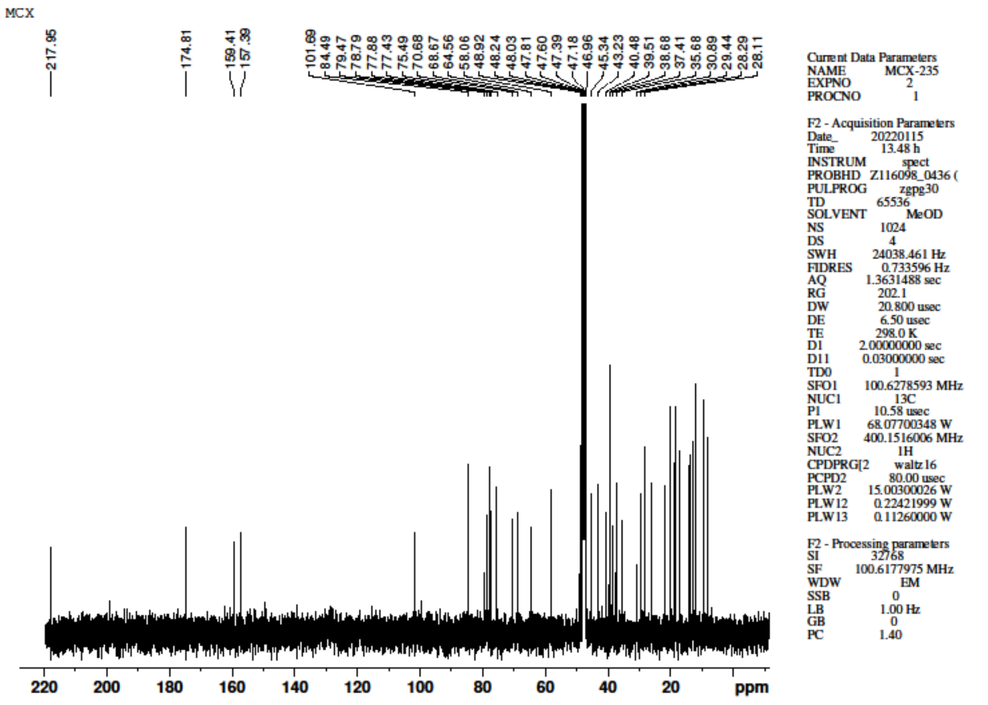
MCX
217.95
174.81
159.41
157.39
101.69
84.49
79.47
78.79
77.88
77.43
75.49
70.68
68.67
64.56
58.06
48.92
48.24
48.03
47.81
47.60
47.39
47.18
46.96
45.34
43.23
40.48
39.51
38.68
37.41
35.68
30.89
29.44
28.29
28.11
Current Data Parameters
NAME MCX-235
EXPNO 2
PROCNO 1
F2 - Acquisition Parameters
Date_ 20220115
Time 13.48 h
INSTRUM spect
PROBHD Z116098_0436 (
PULPROG zgpg30
TD 65536
SOLVENT MeOD
NS 1024
DS 4
SWH 24038.461 Hz
FIDRES 0.733596 Hz
AQ 1.3631488 sec
RG 202.1
DW 20.800 usec
DE 6.50 usec
TE 298.0 K
D1 2.00000000 sec
D11 0.03000000 sec
TD0 1
SFO1 100.6278593 MHz
NUC1 13C
P1 10.58 usec
PLW1 68.07700348 W
SFO2 400.1516006 MHz
NUC2 1H
CPDPRG[2 waltz16
PCPD2 80.00 usec
PLW2 15.00300026 W
PLW12 0.22421999 W
PLW13 0.11260000 W
F2 - Processing parameters
SI 32768
SF 100.6177975 MHz
WDW EM
SSB 0
LB 1.00 Hz
GB 0
PC 1.40
220
200
180
160
140
120
100
80
60
40
20
ppm

88g

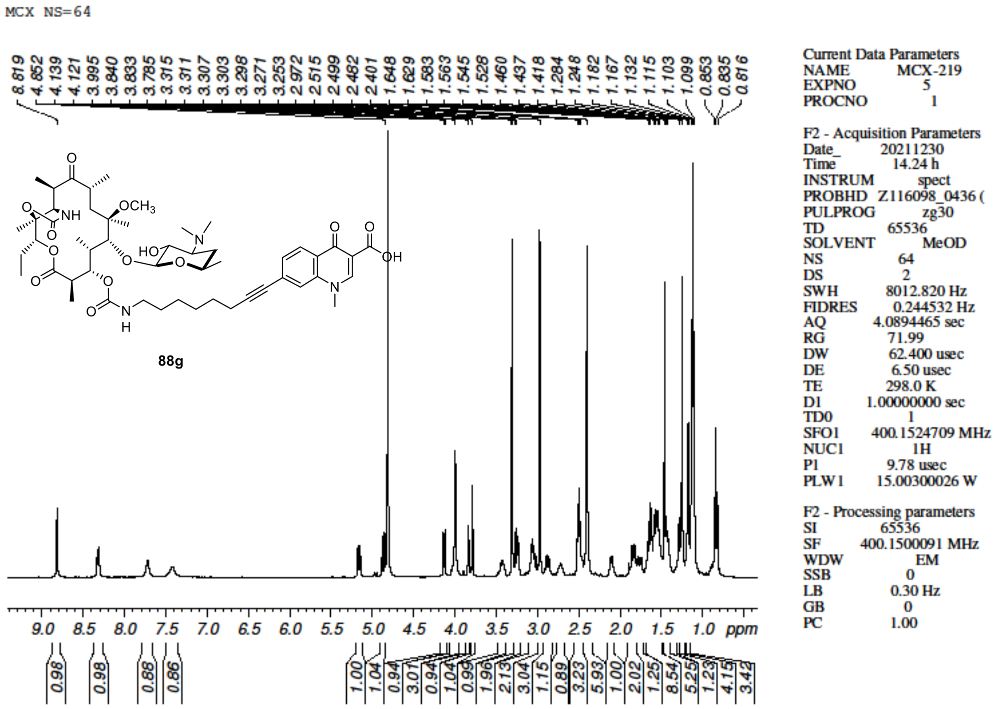
MCX NS=64
8.819 4.852 4.139 4.121 3.995 3.840 3.833 3.785 3.315 3.311 3.307 3.303 3.298 3.271 3.253 2.972 2.515 2.499 2.482 2.401 1.648 1.629 1.583 1.563 1.545 1.528 1.460 1.437 1.418 1.284 1.248 1.182 1.167 1.132 1.115 1.103 1.099 0.853 0.835 0.816
OCH3
NH
HO
N
O
OH
88g
9.0 8.5 8.0 7.5 7.0 6.5 6.0 5.5 5.0 4.5 4.0 3.5 3.0 2.5 2.0 1.5 1.0 ppm
0.98 0.98 0.88 0.86 1.00 1.04 0.94 3.01 0.94 1.04 0.99 0.96 1.96 2.13 3.04 1.15 0.89 3.23 5.93 1.00 2.02 1.25 8.54 5.25 1.23 4.15 3.42
Current Data Parameters
NAME MCX-219
EXPNO 5
PROCNO 1
F2 - Acquisition Parameters
Date_ 20211230
Time 14.24 h
INSTRUM spect
PROBHD Z116098_0436 (
PULPROG zg30
TD 65536
SOLVENT MeOD
NS 64
DS 2
SWH 8012.820 Hz
FIDRES 0.244532 Hz
AQ 4.0894465 sec
RG 71.99
DW 62.400 usec
DE 6.50 usec
TE 298.0 K
D1 1.00000000 sec
TD0 1
SFO1 400.1524709 MHz
NUC1 1H
P1 9.78 usec
PLW1 15.00300026 W
F2 - Processing parameters
SI 65536
SF 400.1500091 MHz
WDW EM
SSB 0
LB 0.30 Hz
GB 0
PC 1.00

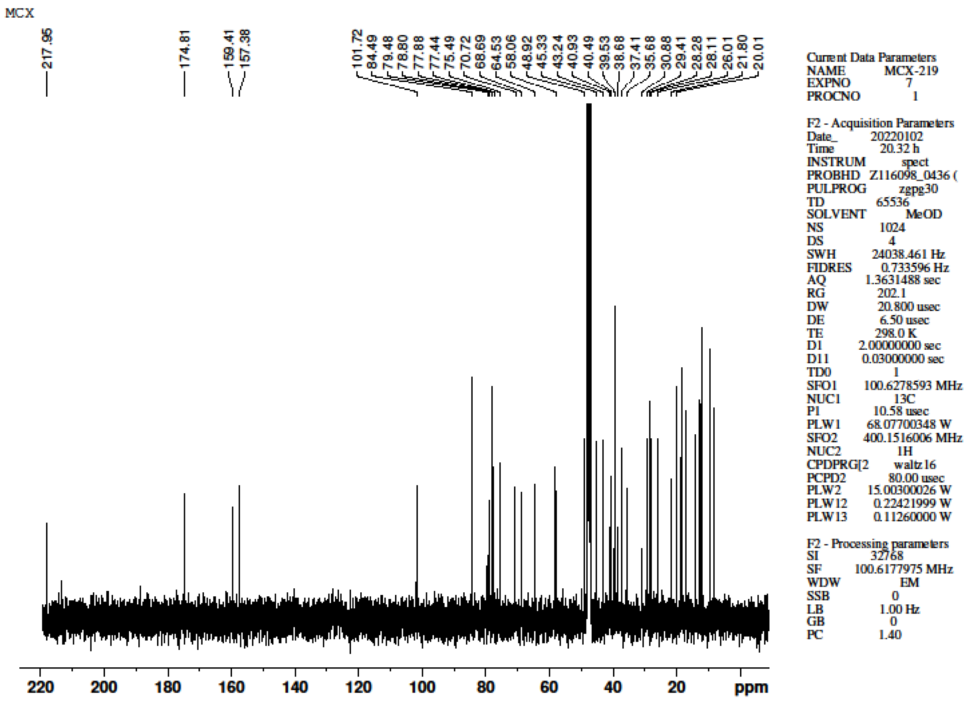
MCX
217.95 174.81 159.41 157.38 101.72 84.49 79.48 78.80 77.88 77.44 75.49 70.72 68.69 64.53 58.06 48.92 45.33 43.24 40.93 40.49 39.53 38.68 37.41 35.68 30.88 29.41 28.28 28.11 26.01 21.80 20.01
220 200 180 160 140 120 100 80 60 40 20 ppm
Current Data Parameters
NAME MCX-219
EXPNO 7
PROCNO 1
F2 - Acquisition Parameters
Date_ 20220102
Time 20.32 h
INSTRUM spect
PROBHD Z116098_0436 (
PULPROG zgpg30
TD 65536
SOLVENT MeOD
NS 1024
DS 4
SWH 24038.461 Hz
FIDRES 0.733596 Hz
AQ 1.3631488 sec
RG 202.1
DW 20.800 usec
DE 6.50 usec
TE 298.0 K
D1 2.00000000 sec
D11 0.03000000 sec
TD0 1
SFO1 100.6278593 MHz
NUC1 13C
P1 10.58 usec
PLW1 68.07700348 W
SFO2 400.1516006 MHz
NUC2 1H
CPDPRG[2 waltz16
PCPD2 80.00 usec
PLW2 15.00300026 W
PLW12 0.22421999 W
PLW13 0.11260000 W
F2 - Processing parameters
SI 32768
SF 100.6177975 MHz
WDW EM
SSB 0
LB 1.00 Hz
GB 0
PC 1.40

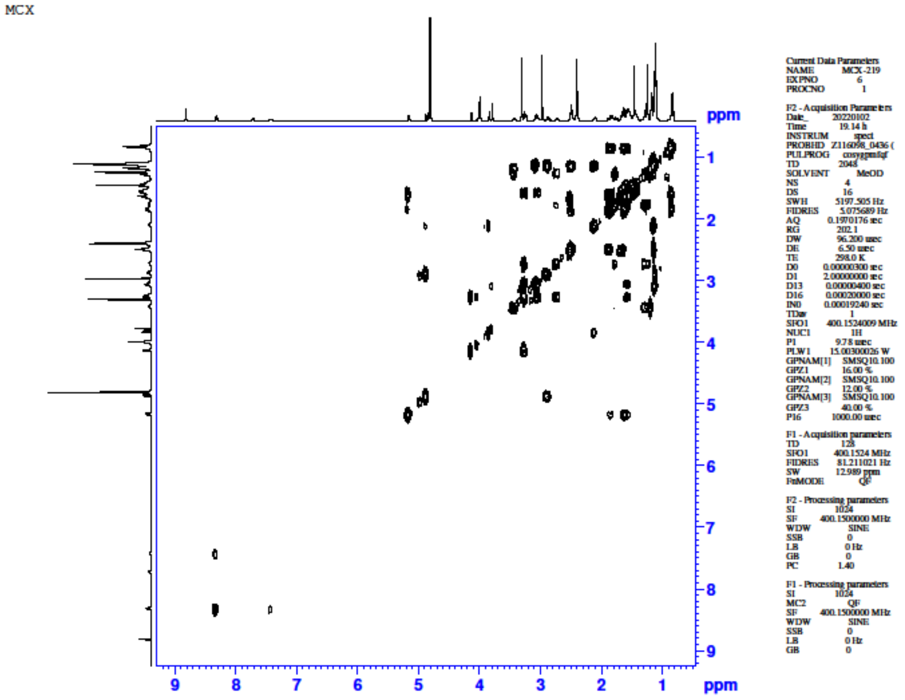

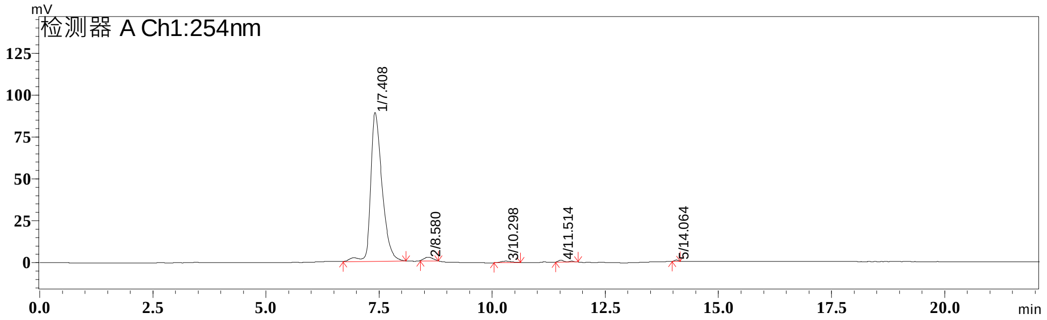

Retention	Peak start	Peak End	peak area	peak height	Area %
7.408	6.708	8.092	1568877	88890	96.0230
8.580	8.417	8.808	33286	2283	2.0373
10.298	10.042	10.625	14889	886	0.9113
11.514	11.400	11.900	12436	1169	0.7612
14.064	13.975	14.158	4367	834	0.2673

88h

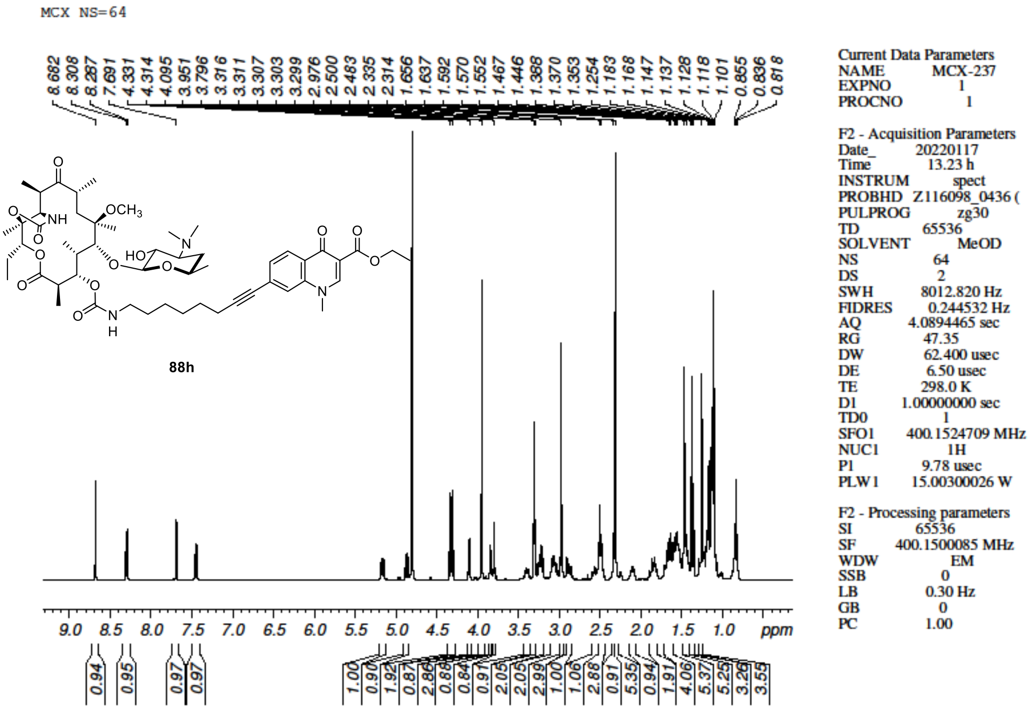
MCX NS=64
8.682 8.308 8.287 7.691 4.331 4.314 4.095 3.951 3.796 3.316 3.311 3.307 3.303 3.299 2.976 2.500 2.483 2.335 2.314 1.656 1.637 1.592 1.570 1.552 1.467 1.446 1.388 1.370 1.353 1.254 1.183 1.168 1.147 1.137 1.128 1.118 1.101 0.855 0.836 0.818
OCH3
NH
HO
N
88h
9.0 8.5 8.0 7.5 7.0 6.5 6.0 5.5 5.0 4.5 4.0 3.5 3.0 2.5 2.0 1.5 1.0 ppm
0.94 0.95 0.97 0.97 1.00 0.90 1.92 0.87 2.86 0.88 0.84 0.91 2.05 2.05 2.99 1.00 1.06 2.88 0.91 5.35 0.94 1.91 4.06 5.37 5.25 3.26 3.55
Current Data Parameters
NAME MCX-237
EXPNO 1
PROCNO 1
F2 - Acquisition Parameters
Date_ 20220117
Time 13.23 h
INSTRUM spect
PROBHD Z116098_0436 (
PULPROG zg30
TD 65536
SOLVENT MeOD
NS 64
DS 2
SWH 8012.820 Hz
FIDRES 0.244532 Hz
AQ 4.0894465 sec
RG 47.35
DW 62.400 usec
DE 6.50 usec
TE 298.0 K
D1 1.00000000 sec
TD0 1
SFO1 400.1524709 MHz
NUC1 1H
P1 9.78 usec
PLW1 15.00300026 W
F2 - Processing parameters
SI 65536
SF 400.1500085 MHz
WDW EM
SSB 0
LB 0.30 Hz
GB 0
PC 1.00

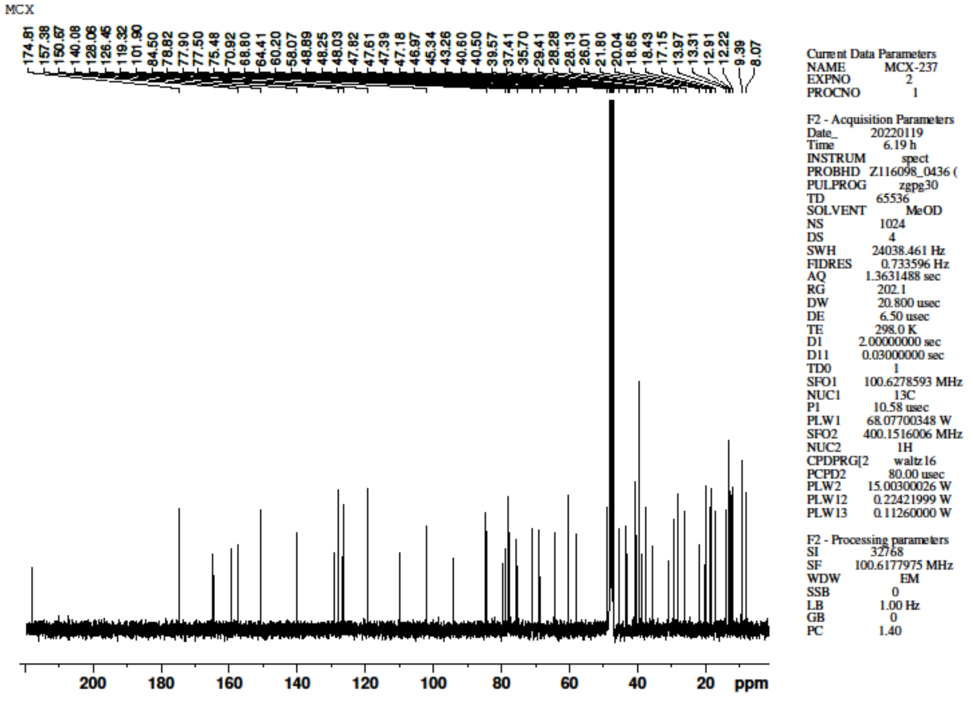
MCX
174.81 157.38 150.67 140.08 128.06 126.45 119.32 101.90 84.50 78.82 77.90 77.50 75.48 70.92 68.80 64.41 60.20 58.07 48.89 48.25 48.03 47.82 47.61 47.39 47.18 46.97 45.34 43.26 40.60 40.50 39.57 37.41 35.70 29.41 28.28 28.13 26.01 21.80 20.04 18.65 18.43 17.15 13.97 13.31 12.91 12.22 9.39 8.07
200 180 160 140 120 100 80 60 40 20 ppm
Current Data Parameters
NAME MCX-237
EXPNO 2
PROCNO 1
F2 - Acquisition Parameters
Date_ 20220119
Time 6.19 h
INSTRUM spect
PROBHD Z116098_0436 (
PULPROG zgpg30
TD 65536
SOLVENT MeOD
NS 1024
DS 4
SWH 24038.461 Hz
FIDRES 0.733596 Hz
AQ 1.3631488 sec
RG 202.1
DW 20.800 usec
DE 6.50 usec
TE 298.0 K
D1 2.00000000 sec
D11 0.03000000 sec
TD0 1
SFO1 100.6278593 MHz
NUC1 13C
P1 10.58 usec
PLW1 68.07700348 W
SFO2 400.1516006 MHz
NUC2 1H
CPDPRG[2 waltz16
PCPD2 80.00 usec
PLW2 15.00300026 W
PLW12 0.22421999 W
PLW13 0.11260000 W
F2 - Processing parameters
SI 32768
SF 100.6177975 MHz
WDW EM
SSB 0
LB 1.00 Hz
GB 0
PC 1.40

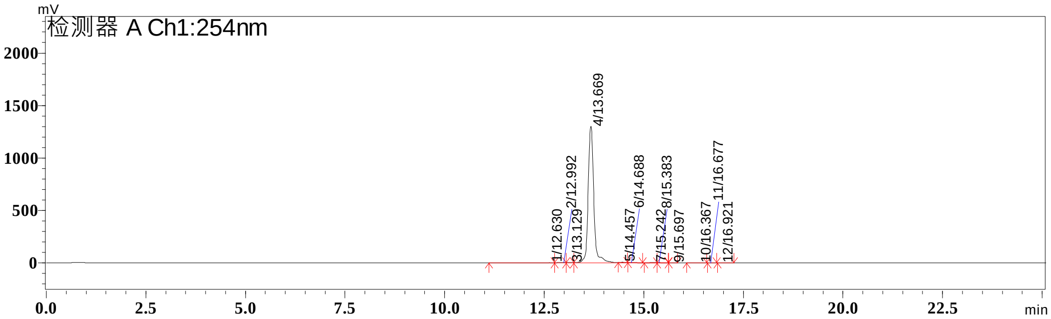

Retention	Peak start	Peak End	peak area	peak height	Area %
12.630	11.117	12.758	80123	1885	0.6073
12.992	12.758	13.050	59851	4451	0.4537
13.129	13.050	13.242	51038	4625	0.3869
13.669	13.242	15.625	12884831	1301656	97.6652
14.457	14.358	14.592	20311	2857	0.1540
14.688	14.600	14.975	16843	1712	0.1277
15.242	15.017	15.333	40402	4470	0.3062
15.383	15.333	15.617	16077	2415	0.1219
15.697	15.625	15.842	2561	468	0.0194
16.367	16.075	16.583	18492	2185	0.1402
16.677	16.600	16.833	1146	200	0.0087
16.921	16.850	17.258	1187	113	0.0090

88i

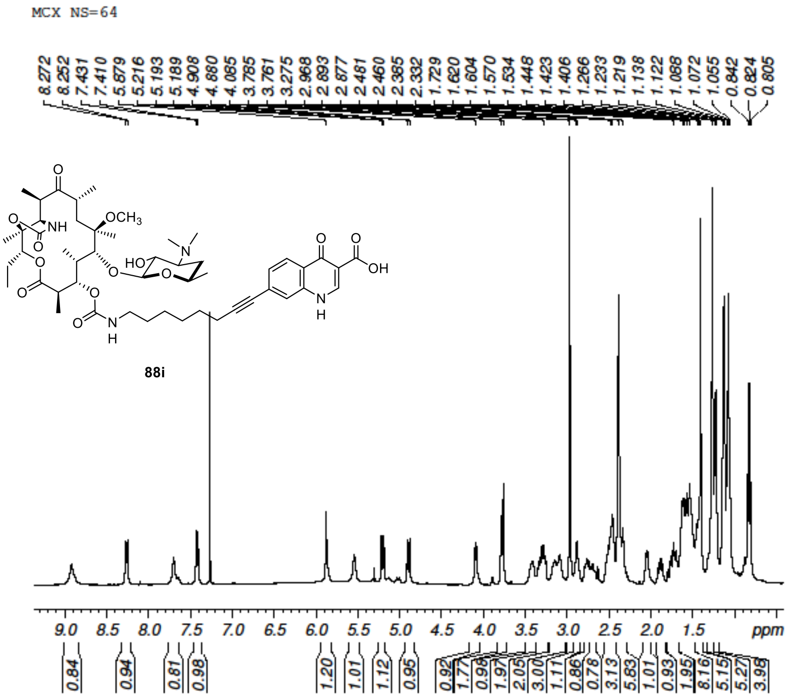

Current Data Parameters
NAME MCX-204
EXPNO 1
PROCNO 1

F2 - Acquisition Parameters
Date_ 20210730
Time 10.03 h
INSTRUM spect
PROBHD Z116098_0436 (
PULPROG zg30
TD 65536
SOLVENT CDCl3
NS 64
DS 2
SWH 8012.820 Hz
FIDRES 0.244532 Hz
AQ 4.0894465 sec
RG 89.02
DW 62.400 usec
DE 6.50 usec
TE 300.2 K
D1 1.00000000 sec
TD0 1
SFO1 400.1524709 MHz
NUC1 1H
P1 9.78 usec
PLW1 15.00300026 W

F2 - Processing parameters
SI 65536
SF 400.1500073 MHz
WDW EM
SSB 0
LB 0.30 Hz
GB 0
PC 1.00

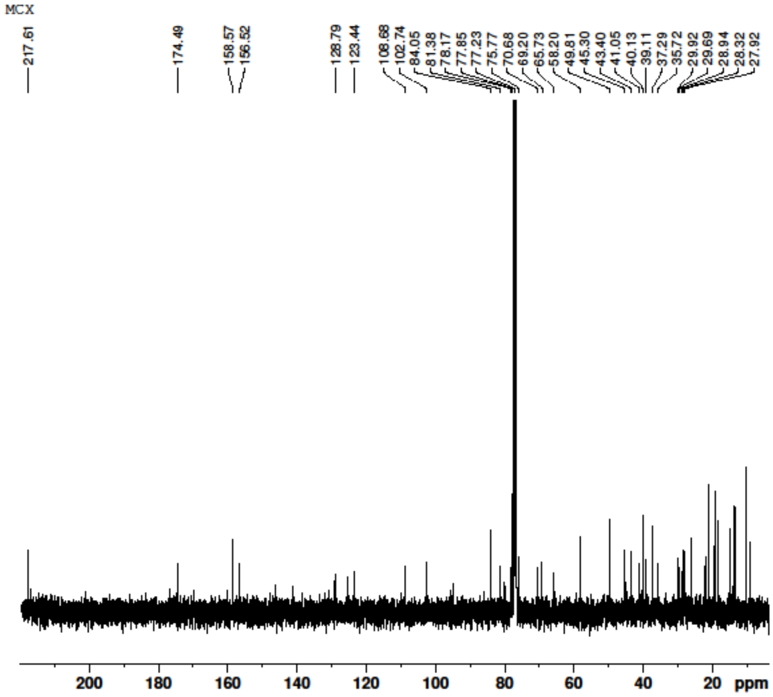

Current Data Parameters
NAME MCX-204
EXPNO 2
PROCNO 1

F2 - Acquisition Parameters
Date_ 20210801
Time 2.17 h
INSTRUM spect
PROBHD Z116098_0436 (
PULPROG zgpg30
TD 65536
SOLVENT CDCl3
NS 1024
DS 4
SWH 24038.461 Hz
FIDRES 0.733596 Hz
AQ 1.3631488 sec
RG 202.1
DW 20.800 usec
DE 6.50 usec
TE 302.2 K
D1 2.00000000 sec
D11 0.03000000 sec
TD0 1
SFO1 100.6278593 MHz
NUC1 13C
P1 10.58 usec
PLW1 68.07700348 W
SFO2 400.1516006 MHz
NUC2 1H
CPDPRG[2 waltz16
PCPD2 80.00 usec
PLW2 15.00300026 W
PLW12 0.22421999 W
PLW13 0.11260000 W

F2 - Processing parameters
SI 32768
SF 100.6177975 MHz
WDW EM
SSB 0
LB 1.00 Hz
GB 0
PC 1.40

88j

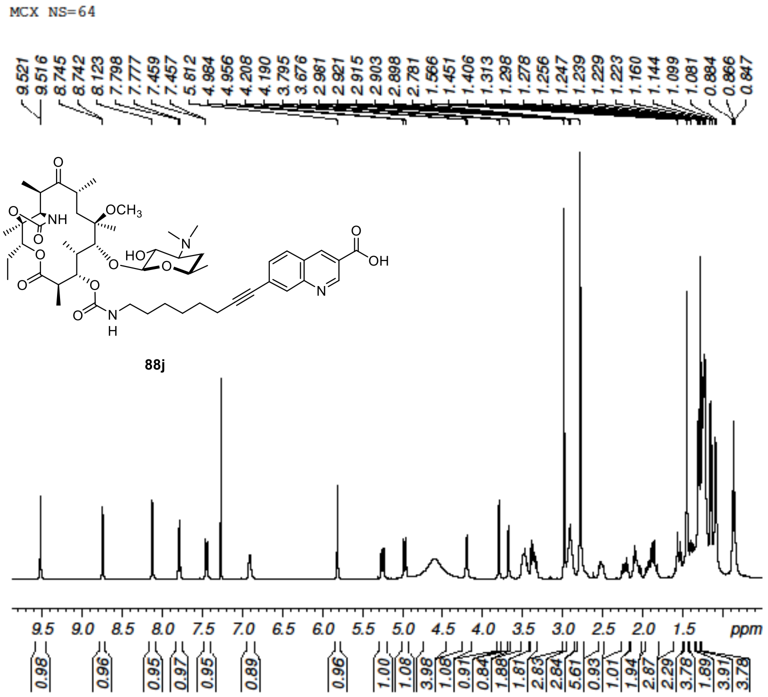

Current Data Parameters
NAME MCX-210
EXPNO 1
PROCNO 1

F2 - Acquisition Parameters
Date_ 20210813
Time 20.42 h
INSTRUM spect
PROBHD Z116098_0436 (
PULPROG zg30
TD 65536
SOLVENT CDCl3
NS 64
DS 2
SWH 8012.820 Hz
FIDRES 0.244532 Hz
AQ 4.0894465 sec
RG 79.39
DW 62.400 usec
DE 6.50 usec
TE 301.3 K
D1 1.00000000 sec
TD0 1
SFO1 400.1524709 MHz
NUC1 1H
P1 9.78 usec
PLW1 15.00300026 W

F2 - Processing parameters
SI 65536
SF 400.1500068 MHz
WDW EM
SSB 0
LB 0.30 Hz
GB 0
PC 1.00

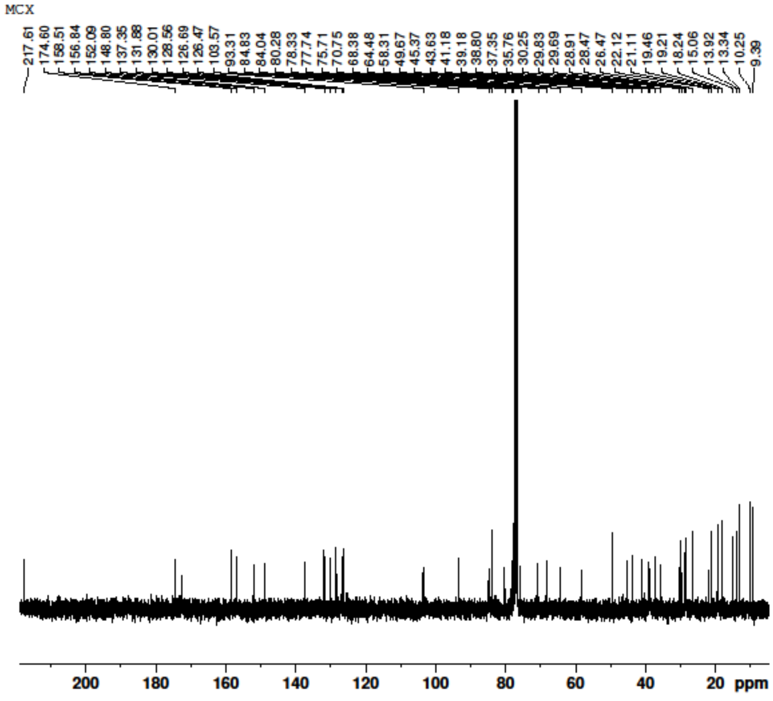

Current Data Parameters
NAME MCX-210
EXPNO 2
PROCNO 1

F2 - Acquisition Parameters
Date_ 20210814
Time 13.08 h
INSTRUM spect
PROBHD Z116098_0436 (
PULPROG zgpg30
TD 65536
SOLVENT CDCl3
NS 1024
DS 4
SWH 24038.461 Hz
FIDRES 0.733596 Hz
AQ 1.3631488 sec
RG 202.1
DW 20.800 usec
DE 6.50 usec
TE 301.8 K
D1 2.00000000 sec
D11 0.03000000 sec
TD0 1
SFO1 100.6278593 MHz
NUC1 13C
P1 10.58 usec
PLW1 68.07700348 W
SFO2 400.1516006 MHz
NUC2 1H
CPDPRG[2 waltz16
PCPD2 80.00 usec
PLW2 15.00300026 W
PLW12 0.22421999 W
PLW13 0.11260000 W

F2 - Processing parameters
SI 32768
SF 100.6177975 MHz
WDW EM
SSB 0
LB 1.00 Hz
GB 0
PC 1.40

88k

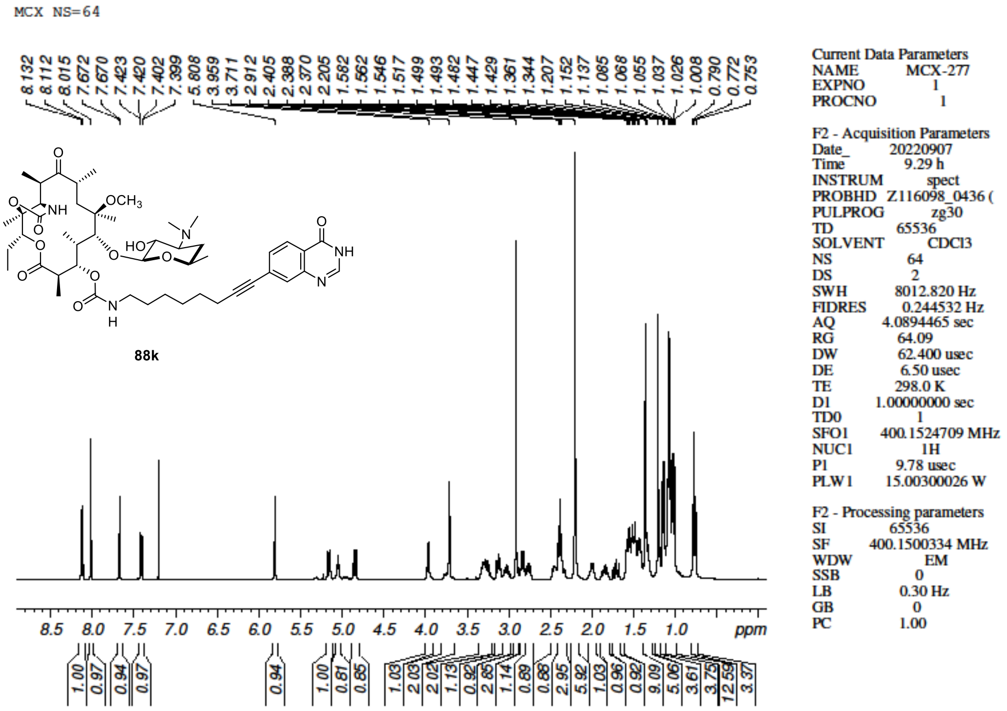
MCX NS=64
8.132 8.112 8.015 7.672 7.670 7.423 7.420 7.402 7.399 5.808 3.959 3.711 2.912 2.405 2.388 2.370 2.205 1.582 1.562 1.546 1.517 1.499 1.493 1.482 1.447 1.429 1.361 1.344 1.207 1.152 1.137 1.085 1.068 1.055 1.037 1.026 1.008 0.790 0.772 0.753
88k
OCH3
NH
HO
N
8.5 8.0 7.5 7.0 6.5 6.0 5.5 5.0 4.5 4.0 3.5 3.0 2.5 2.0 1.5 1.0 ppm
1.00 0.97 0.94 0.97 0.94 1.00 0.81 0.85 1.03 2.03 2.02 1.13 0.92 2.85 1.14 0.89 0.88 2.95 5.92 1.03 0.96 0.92 9.09 5.06 3.61 3.75 12.59 3.37
Current Data Parameters
NAME MCX-277
EXPNO 1
PROCNO 1
F2 - Acquisition Parameters
Date_ 20220907
Time 9.29 h
INSTRUM spect
PROBHD Z116098_0436 (
PULPROG zg30
TD 65536
SOLVENT CDCl3
NS 64
DS 2
SWH 8012.820 Hz
FIDRES 0.244532 Hz
AQ 4.0894465 sec
RG 64.09
DW 62.400 usec
DE 6.50 usec
TE 298.0 K
D1 1.00000000 sec
TD0 1
SFO1 400.1524709 MHz
NUC1 1H
P1 9.78 usec
PLW1 15.00300026 W
F2 - Processing parameters
SI 65536
SF 400.1500334 MHz
WDW EM
SSB 0
LB 0.30 Hz
GB 0
PC 1.00

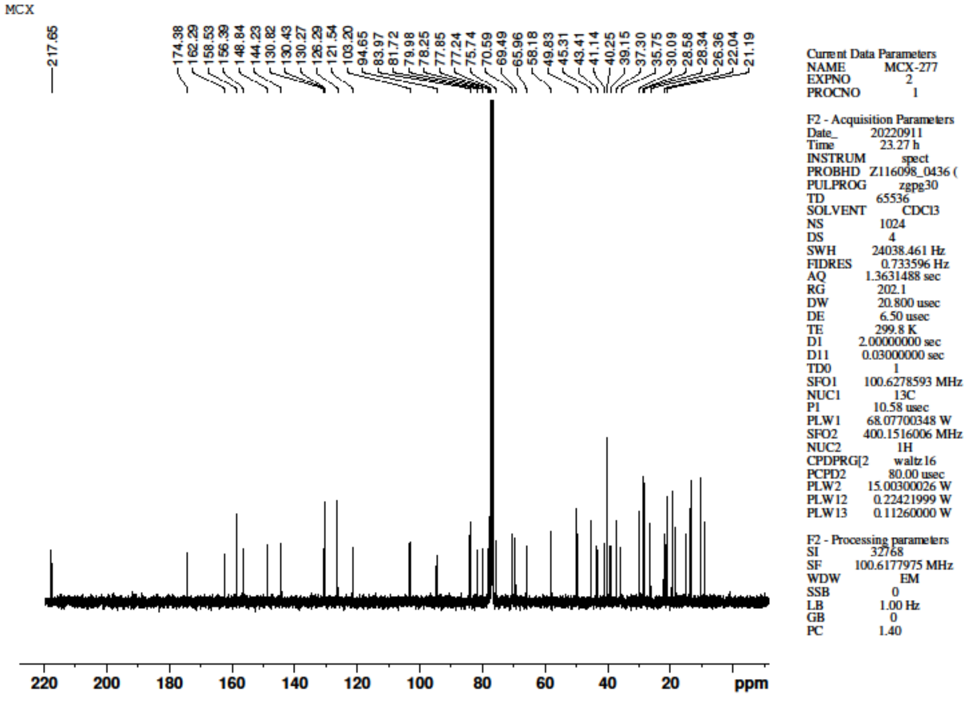
MCX
217.65
174.38 162.29 158.53 156.39 148.84 144.23 130.82 130.43 130.27 126.29 121.54 103.20 94.65 83.97 81.72 79.98 78.25 77.85 77.24 75.74 70.59 69.49 65.96 58.18 49.83 45.31 43.41 41.14 40.25 39.15 37.30 35.75 30.09 28.58 28.34 26.36 22.04 21.19
220 200 180 160 140 120 100 80 60 40 20 ppm
Current Data Parameters
NAME MCX-277
EXPNO 2
PROCNO 1
F2 - Acquisition Parameters
Date_ 20220911
Time 23.27 h
INSTRUM spect
PROBHD Z116098_0436 (
PULPROG zgpg30
TD 65536
SOLVENT CDCl3
NS 1024
DS 4
SWH 24038.461 Hz
FIDRES 0.733596 Hz
AQ 1.3631488 sec
RG 202.1
DW 20.800 usec
DE 6.50 usec
TE 299.8 K
D1 2.00000000 sec
D11 0.03000000 sec
TD0 1
SFO1 100.6278593 MHz
NUC1 13C
P1 10.58 usec
PLW1 68.07700348 W
SFO2 400.1516006 MHz
NUC2 1H
CPDPRG[2 waltz16
PCPD2 80.00 usec
PLW2 15.00300026 W
PLW12 0.22421999 W
PLW13 0.11260000 W
F2 - Processing parameters
SI 32768
SF 100.6177975 MHz
WDW EM
SSB 0
LB 1.00 Hz
GB 0
PC 1.40

88l

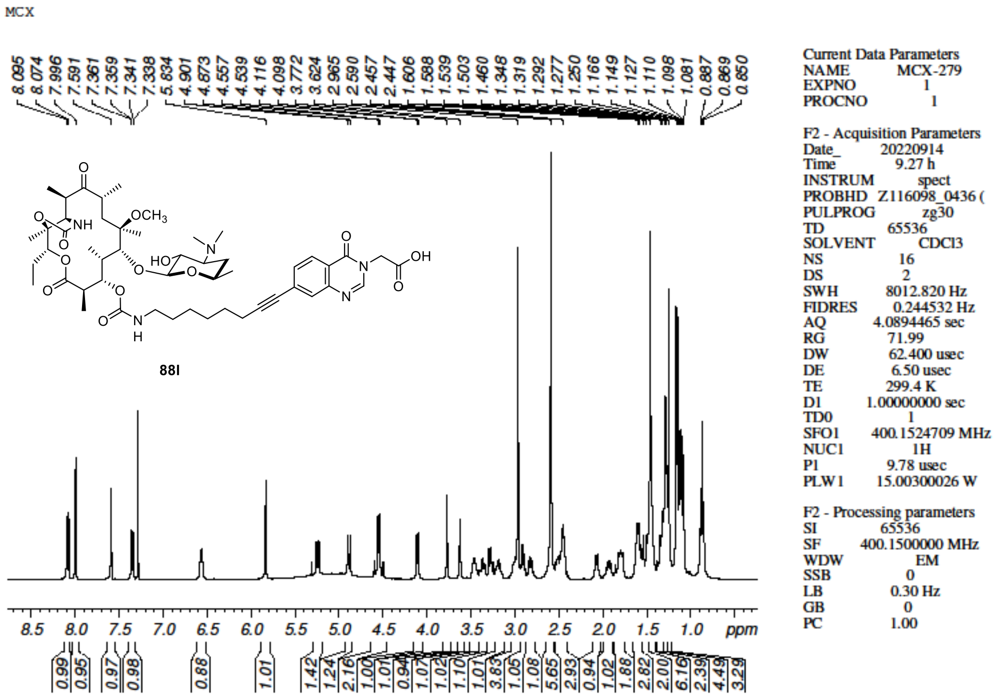
MCX
8.095 8.074 7.996 7.591 7.361 7.359 7.341 7.338 5.834 4.901 4.673 4.557 4.539 4.116 4.098 3.772 3.624 2.965 2.590 2.457 2.447 1.606 1.588 1.539 1.503 1.460 1.348 1.319 1.292 1.277 1.250 1.166 1.149 1.127 1.110 1.098 1.081 0.887 0.869 0.850
88l
8.5 8.0 7.5 7.0 6.5 6.0 5.5 5.0 4.5 4.0 3.5 3.0 2.5 2.0 1.5 1.0 ppm
0.99 0.95 0.97 0.98 0.88 1.01 1.42 1.24 2.16 1.00 1.01 0.94 1.07 1.02 1.10 1.01 3.83 1.05 1.08 5.65 2.93 0.94 1.02 1.88 2.82 2.00 6.16 2.39 4.49 3.29
Current Data Parameters
NAME MCX-279
EXPNO 1
PROCNO 1
F2 - Acquisition Parameters
Date_ 20220914
Time 9.27 h
INSTRUM spect
PROBHD Z116098_0436 (
PULPROG zg30
TD 65536
SOLVENT CDCl3
NS 16
DS 2
SWH 8012.820 Hz
FIDRES 0.244532 Hz
AQ 4.0894465 sec
RG 71.99
DW 62.400 usec
DE 6.50 usec
TE 299.4 K
D1 1.00000000 sec
TD0 1
SFO1 400.1524709 MHz
NUC1 1H
P1 9.78 usec
PLW1 15.00300026 W
F2 - Processing parameters
SI 65536
SF 400.1500000 MHz
WDW EM
SSB 0
LB 0.30 Hz
GB 0
PC 1.00

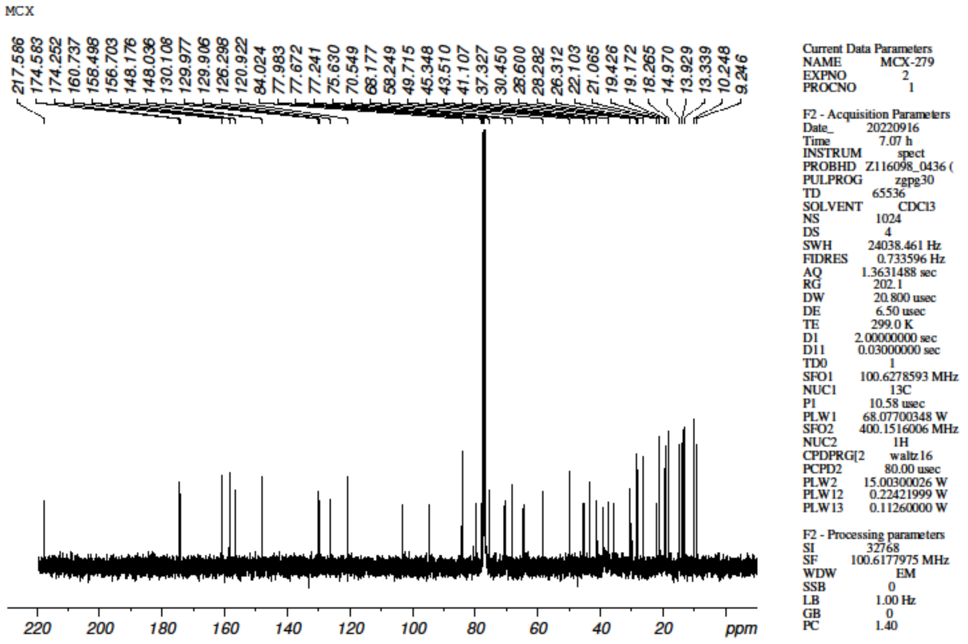
MCX
217.586 174.583 174.252 160.737 158.498 156.703 148.176 148.036 130.108 129.977 129.906 126.298 120.922 84.024 77.983 77.672 77.241 75.630 70.549 68.177 58.249 49.715 45.348 43.510 41.107 37.327 30.450 28.600 28.282 26.312 22.103 21.065 19.426 19.172 18.265 14.970 13.929 13.339 10.248 9.246
220 200 180 160 140 120 100 80 60 40 20 ppm
Current Data Parameters
NAME MCX-279
EXPNO 2
PROCNO 1
F2 - Acquisition Parameters
Date_ 20220916
Time 7.07 h
INSTRUM spect
PROBHD Z116098_0436 (
PULPROG zgpg30
TD 65536
SOLVENT CDCl3
NS 1024
DS 4
SWH 24038.461 Hz
FIDRES 0.733596 Hz
AQ 1.3631488 sec
RG 202.1
DW 20.800 usec
DE 6.50 usec
TE 299.0 K
D1 2.00000000 sec
D11 0.03000000 sec
TD0 1
SFO1 100.6278593 MHz
NUC1 13C
P1 10.58 usec
PLW1 68.07700348 W
SFO2 400.1516006 MHz
NUC2 1H
CPDPRG[2 waltz16
PCPD2 80.00 usec
PLW2 15.00300026 W
PLW12 0.22421999 W
PLW13 0.11260000 W
F2 - Processing parameters
SI 32768
SF 100.6177975 MHz
WDW EM
SSB 0
LB 1.00 Hz
GB 0
PC 1.40

88m

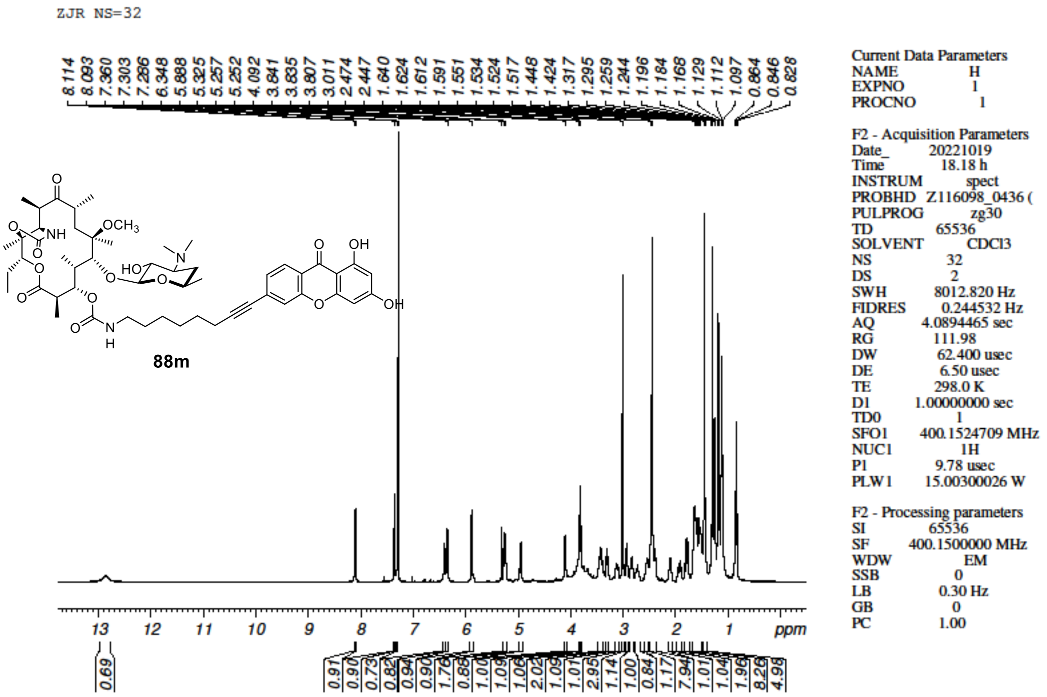
ZJR NS=32
8.114 8.093 7.360 7.303 7.286 6.348 5.888 5.325 5.257 5.252 4.092 3.841 3.835 3.807 3.011 2.474 2.447 1.640 1.624 1.612 1.591 1.551 1.534 1.524 1.517 1.448 1.424 1.317 1.295 1.259 1.244 1.196 1.184 1.168 1.129 1.112 1.097 0.864 0.846 0.828
OCH3
NH
HO
OH
88m
13 12 11 10 9 8 7 6 5 4 3 2 1 ppm
0.69 0.91 0.90 0.73 0.82 0.94 0.90 1.76 0.88 1.00 1.09 1.06 2.02 1.09 1.01 2.95 1.14 1.00 0.84 1.17 7.94 1.01 1.04 1.96 8.26 4.98
Current Data Parameters
NAME H
EXPNO 1
PROCNO 1
F2 - Acquisition Parameters
Date_ 20221019
Time 18.18 h
INSTRUM spect
PROBHD Z116098_0436 (
PULPROG zg30
TD 65536
SOLVENT CDCl3
NS 32
DS 2
SWH 8012.820 Hz
FIDRES 0.244532 Hz
AQ 4.0894465 sec
RG 111.98
DW 62.400 usec
DE 6.50 usec
TE 298.0 K
D1 1.00000000 sec
TD0 1
SFO1 400.1524709 MHz
NUC1 1H
P1 9.78 usec
PLW1 15.00300026 W
F2 - Processing parameters
SI 65536
SF 400.1500000 MHz
WDW EM
SSB 0
LB 0.30 Hz
GB 0
PC 1.00

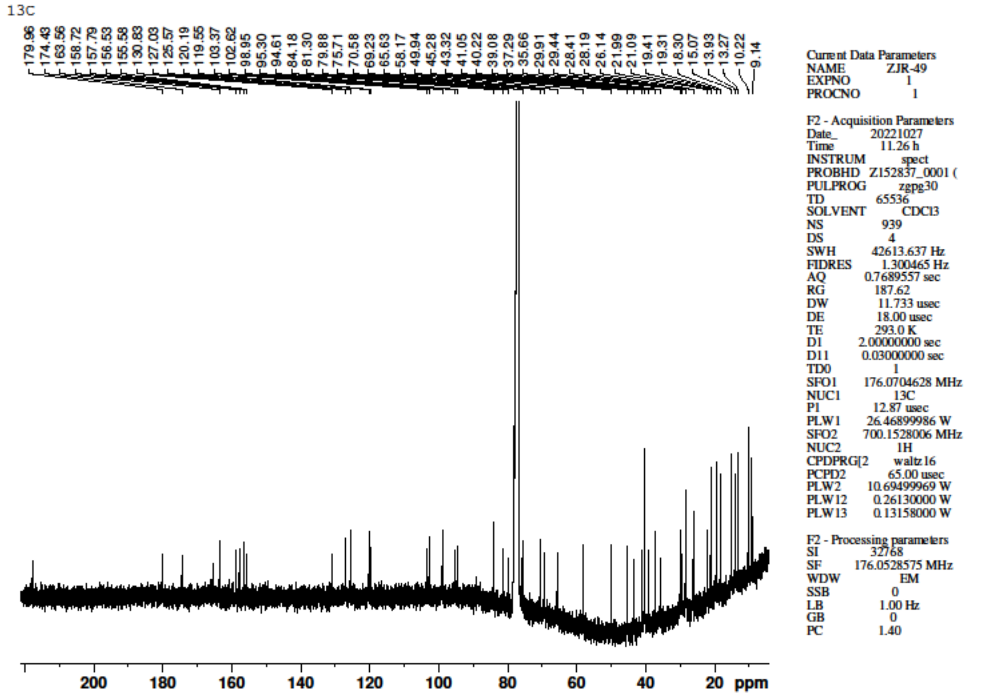
13C
179.96 174.43 163.56 158.72 157.79 156.53 155.58 130.83 127.03 125.57 120.19 119.55 103.37 102.62 98.95 95.30 94.61 84.18 81.30 79.88 75.71 70.58 69.23 65.63 58.17 49.94 45.28 43.32 41.05 40.22 39.08 37.29 35.66 29.91 29.44 28.41 28.19 26.14 21.99 21.09 19.41 19.31 18.30 15.07 13.93 13.27 10.22 9.14
200 180 160 140 120 100 80 60 40 20 ppm
Current Data Parameters
NAME ZJR-49
EXPNO 1
PROCNO 1
F2 - Acquisition Parameters
Date_ 20221027
Time 11.26 h
INSTRUM spect
PROBHD Z152837_0001 (
PULPROG zgpg30
TD 65536
SOLVENT CDCl3
NS 939
DS 4
SWH 42613.637 Hz
FIDRES 1.300465 Hz
AQ 0.7689557 sec
RG 187.62
DW 11.733 usec
DE 18.00 usec
TE 293.0 K
D1 2.00000000 sec
D11 0.03000000 sec
TD0 1
SFO1 176.0704628 MHz
NUC1 13C
P1 12.87 usec
PLW1 26.46899986 W
SFO2 700.1528006 MHz
NUC2 1H
CPDPRG[2 waltz16
PCPD2 65.00 usec
PLW2 10.69499969 W
PLW12 0.26130000 W
PLW13 0.13158000 W
F2 - Processing parameters
SI 32768
SF 176.0528575 MHz
WDW EM
SSB 0
LB 1.00 Hz
GB 0
PC 1.40

90g

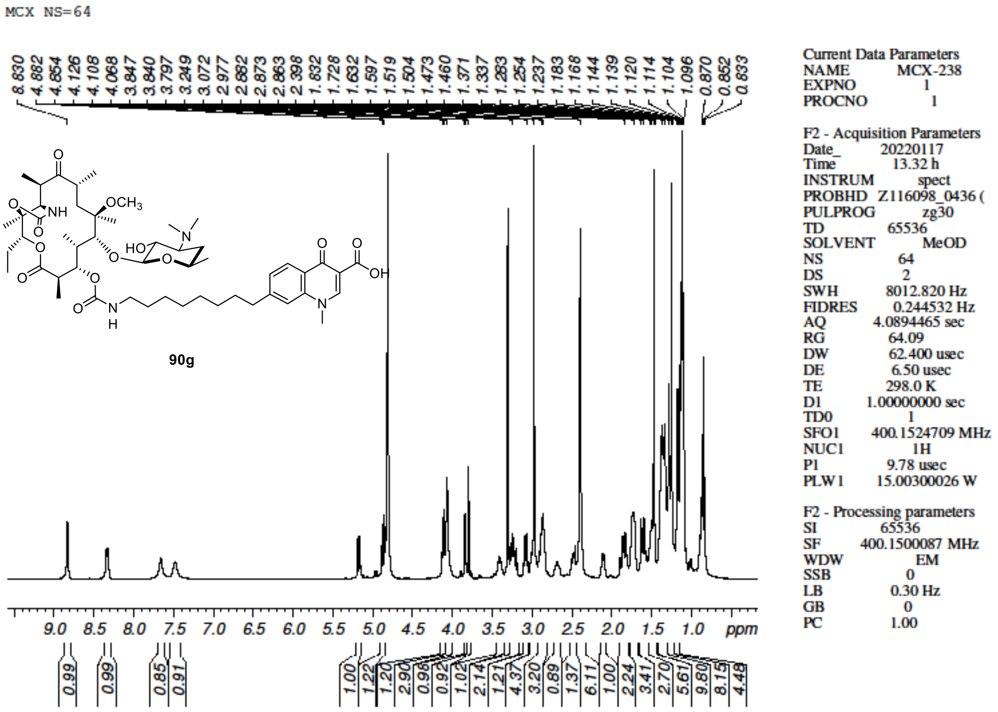
MCX NS=64
8.830 4.882 4.854 4.126 4.108 4.068 3.847 3.840 3.797 3.249 3.072 2.977 2.882 2.873 2.863 2.398 1.832 1.728 1.632 1.597 1.519 1.504 1.473 1.460 1.371 1.337 1.283 1.254 1.237 1.183 1.168 1.144 1.139 1.120 1.114 1.104 1.096 0.870 0.852 0.833
OCH3
HO
OH
90g
9.0 8.5 8.0 7.5 7.0 6.5 6.0 5.5 5.0 4.5 4.0 3.5 3.0 2.5 2.0 1.5 1.0 ppm
0.99 0.99 0.85 0.91 1.00 1.22 1.20 2.90 0.96 0.92 1.02 2.14 1.21 4.37 3.20 0.89 1.37 6.11 1.00 2.24 3.41 2.70 5.61 9.80 8.15 4.48
Current Data Parameters
NAME MCX-238
EXPNO 1
PROCNO 1
F2 - Acquisition Parameters
Date_ 20220117
Time 13.32 h
INSTRUM spect
PROBHD Z116098_0436 (
PULPROG zg30
TD 65536
SOLVENT MeOD
NS 64
DS 2
SWH 8012.820 Hz
FIDRES 0.244532 Hz
AQ 4.0894465 sec
RG 64.09
DW 62.400 usec
DE 6.50 usec
TE 298.0 K
D1 1.00000000 sec
TD0 1
SFO1 400.1524709 MHz
NUC1 1H
P1 9.78 usec
PLW1 15.00300026 W
F2 - Processing parameters
SI 65536
SF 400.1500087 MHz
WDW EM
SSB 0
LB 0.30 Hz
GB 0
PC 1.00

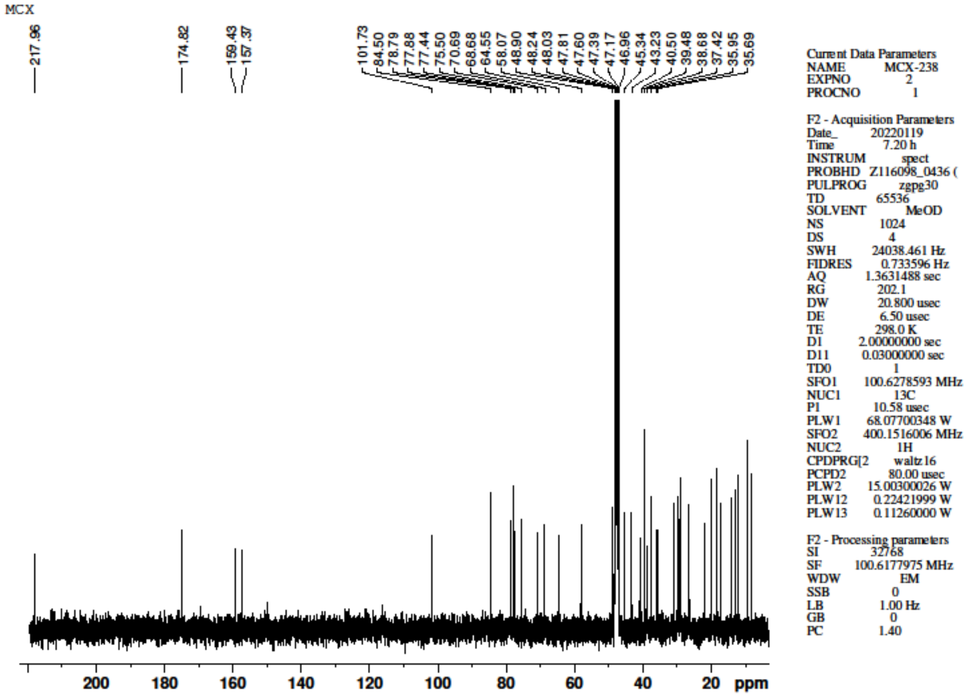
MCX
217.96 174.82 159.43 157.37 101.73 84.50 78.79 77.88 77.44 75.50 70.69 68.68 64.55 58.07 48.90 48.24 48.03 47.81 47.60 47.39 47.17 46.96 45.34 43.23 40.50 39.48 38.68 37.42 35.95 35.69
200 180 160 140 120 100 80 60 40 20 ppm
Current Data Parameters
NAME MCX-238
EXPNO 2
PROCNO 1
F2 - Acquisition Parameters
Date_ 20220119
Time 7.20 h
INSTRUM spect
PROBHD Z116098_0436 (
PULPROG zgpg30
TD 65536
SOLVENT MeOD
NS 1024
DS 4
SWH 24038.461 Hz
FIDRES 0.733596 Hz
AQ 1.3631488 sec
RG 202.1
DW 20.800 usec
DE 6.50 usec
TE 298.0 K
D1 2.00000000 sec
D11 0.03000000 sec
TD0 1
SFO1 100.6278593 MHz
NUC1 13C
P1 10.58 usec
PLW1 68.07700348 W
SFO2 400.1516006 MHz
NUC2 1H
CPDPRG[2 waltz16
PCPD2 80.00 usec
PLW2 15.00300026 W
PLW12 0.22421999 W
PLW13 0.11260000 W
F2 - Processing parameters
SI 32768
SF 100.6177975 MHz
WDW EM
SSB 0
LB 1.00 Hz
GB 0
PC 1.40

95c

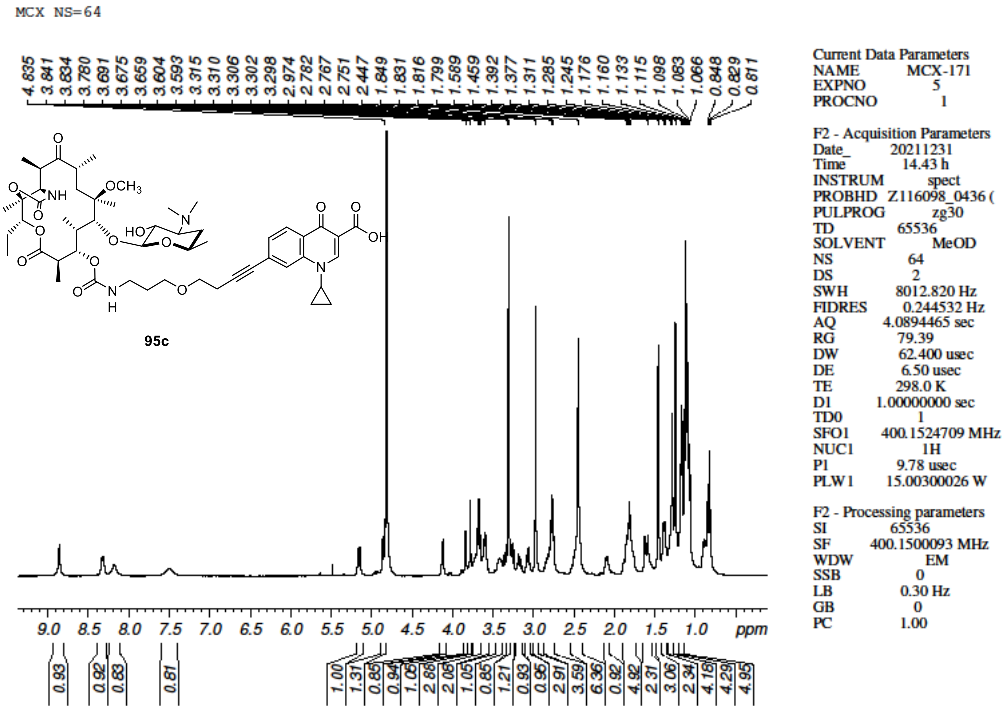

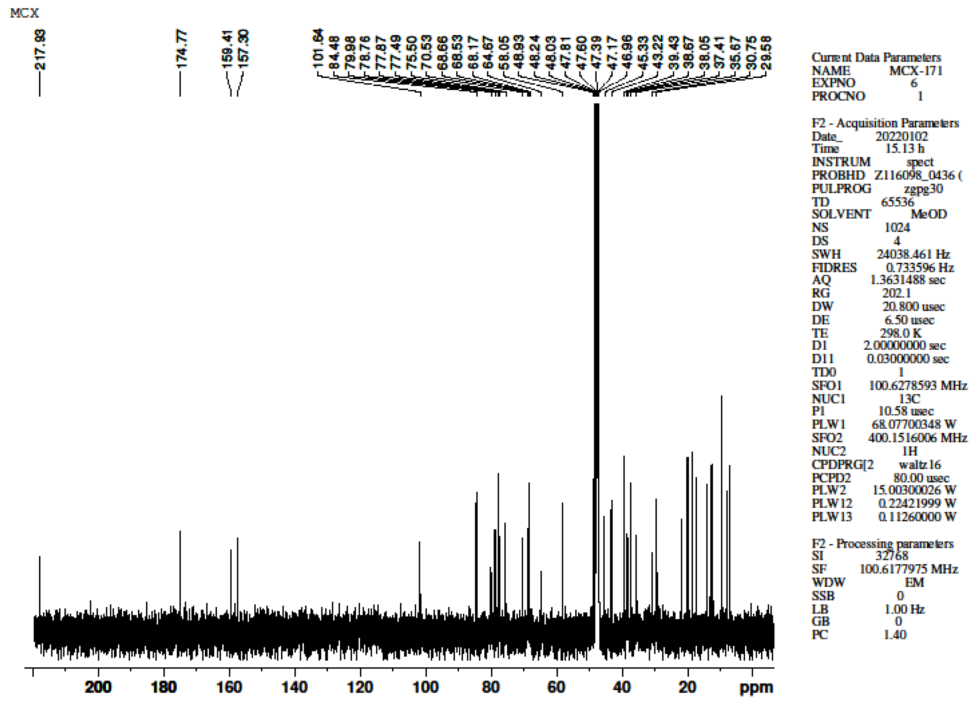

95f

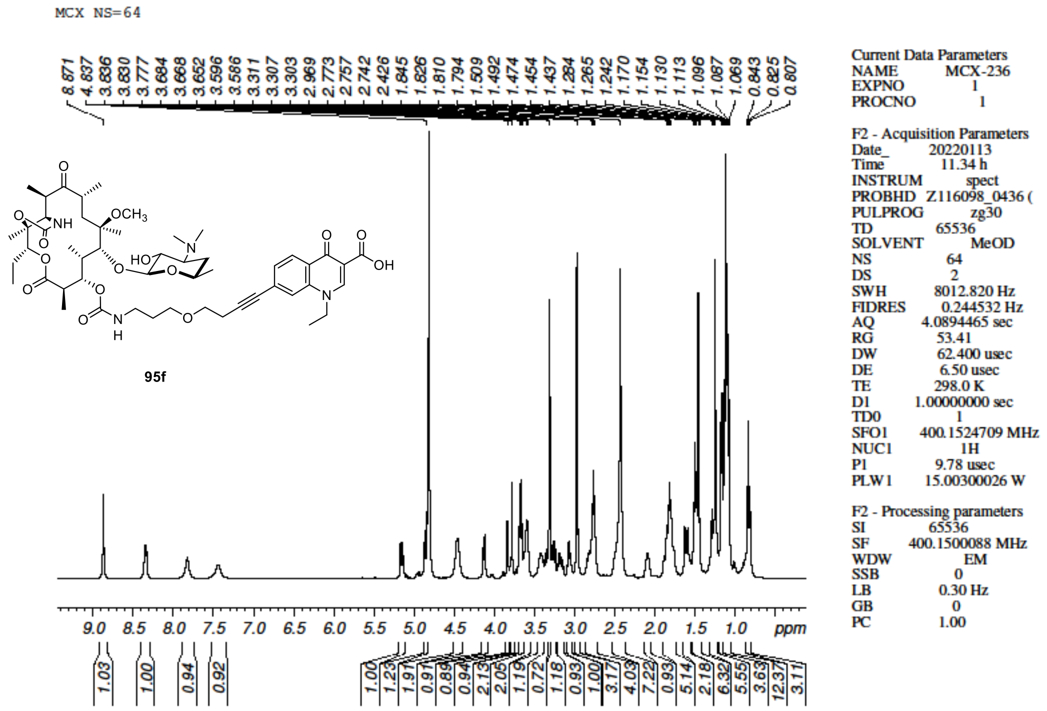
MCX NS=64
8.871 4.837 3.836 3.830 3.777 3.684 3.668 3.652 3.596 3.586 3.311 3.307 3.303 2.969 2.773 2.757 2.742 2.426 1.845 1.826 1.810 1.794 1.509 1.492 1.474 1.454 1.437 1.284 1.265 1.242 1.170 1.154 1.130 1.113 1.096 1.087 1.069 0.843 0.825 0.807
95f
9.0 8.5 8.0 7.5 7.0 6.5 6.0 5.5 5.0 4.5 4.0 3.5 3.0 2.5 2.0 1.5 1.0 ppm
1.03 1.00 0.94 0.92 1.00 1.23 1.91 0.91 0.89 0.94 2.13 2.05 1.19 0.72 1.18 0.93 1.00 3.17 4.03 7.22 0.93 5.14 2.18 6.32 5.55 3.63 12.37 3.11
Current Data Parameters
NAME MCX-236
EXPNO 1
PROCNO 1
F2 - Acquisition Parameters
Date_ 20220113
Time 11.34 h
INSTRUM spect
PROBHD Z116098_0436 (
PULPROG zg30
TD 65536
SOLVENT MeOD
NS 64
DS 2
SWH 8012.820 Hz
FIDRES 0.244532 Hz
AQ 4.0894465 sec
RG 53.41
DW 62.400 usec
DE 6.50 usec
TE 298.0 K
D1 1.00000000 sec
TD0 1
SFO1 400.1524709 MHz
NUC1 1H
P1 9.78 usec
PLW1 15.00300026 W
F2 - Processing parameters
SI 65536
SF 400.1500088 MHz
WDW EM
SSB 0
LB 0.30 Hz
GB 0
PC 1.00

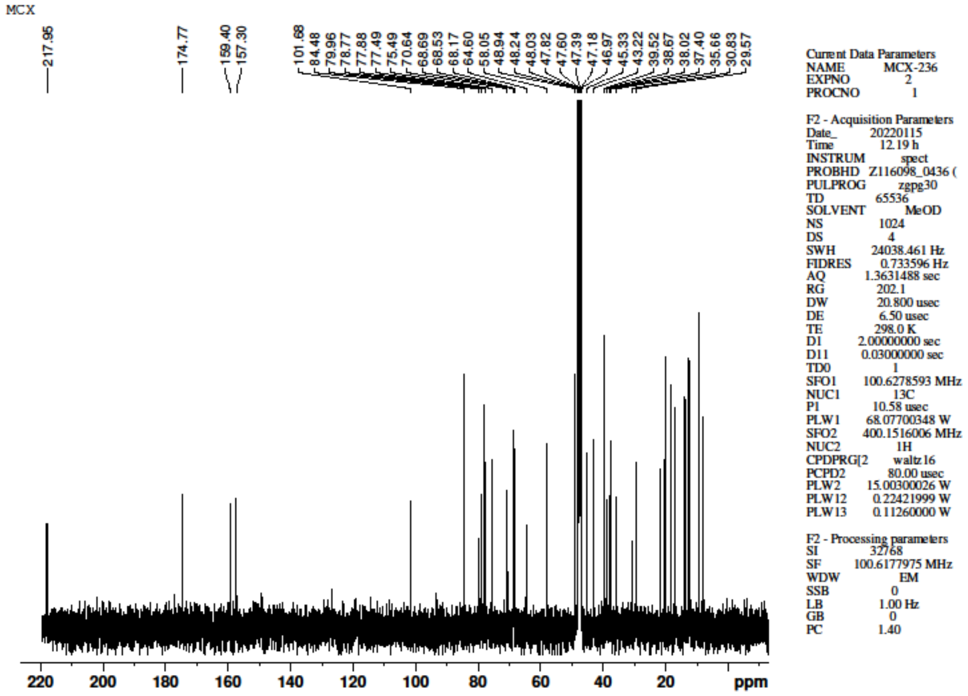
MCX
217.95 174.77 159.40 157.30 101.68 84.48 79.96 78.77 77.88 77.49 75.49 70.64 68.69 68.53 68.17 64.60 58.05 48.94 48.24 48.03 47.82 47.60 47.39 47.18 46.97 45.33 43.22 39.52 38.67 38.02 37.40 35.66 30.83 29.57
220 200 180 160 140 120 100 80 60 40 20 ppm
Current Data Parameters
NAME MCX-236
EXPNO 2
PROCNO 1
F2 - Acquisition Parameters
Date_ 20220115
Time 12.19 h
INSTRUM spect
PROBHD Z116098_0436 (
PULPROG zgpg30
TD 65536
SOLVENT MeOD
NS 1024
DS 4
SWH 24038.461 Hz
FIDRES 0.733596 Hz
AQ 1.3631488 sec
RG 202.1
DW 20.800 usec
DE 6.50 usec
TE 298.0 K
D1 2.00000000 sec
D11 0.03000000 sec
TD0 1
SFO1 100.6278593 MHz
NUC1 13C
P1 10.58 usec
PLW1 68.07700348 W
SFO2 400.1516006 MHz
NUC2 1H
CPDPRG[2 waltz16
PCPD2 80.00 usec
PLW2 15.00300026 W
PLW12 0.22421999 W
PLW13 0.11260000 W
F2 - Processing parameters
SI 32768
SF 100.6177975 MHz
WDW EM
SSB 0
LB 1.00 Hz
GB 0
PC 1.40

95g (MCX-190)

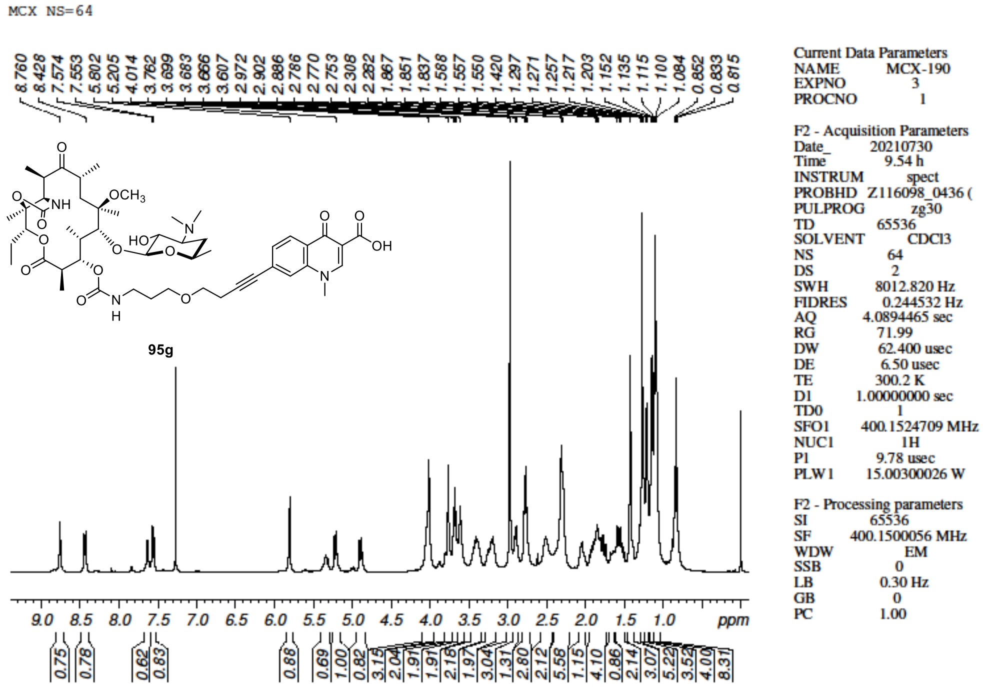
MCX NS=64
95g
Current Data Parameters
NAME MCX-190
EXPNO 3
PROCNO 1
F2 - Acquisition Parameters
Date_ 20210730
Time 9.54 h
INSTRUM spect
PROBHD Z116098_0436 (
PULPROG zg30
TD 65536
SOLVENT CDCl3
NS 64
DS 2
SWH 8012.820 Hz
FIDRES 0.244532 Hz
AQ 4.0894465 sec
RG 71.99
DW 62.400 usec
DE 6.50 usec
TE 300.2 K
D1 1.00000000 sec
TD0 1
SFO1 400.1524709 MHz
NUC1 1H
P1 9.78 usec
PLW1 15.00300026 W
F2 - Processing parameters
SI 65536
SF 400.1500056 MHz
WDW EM
SSB 0
LB 0.30 Hz
GB 0
PC 1.00
ppm

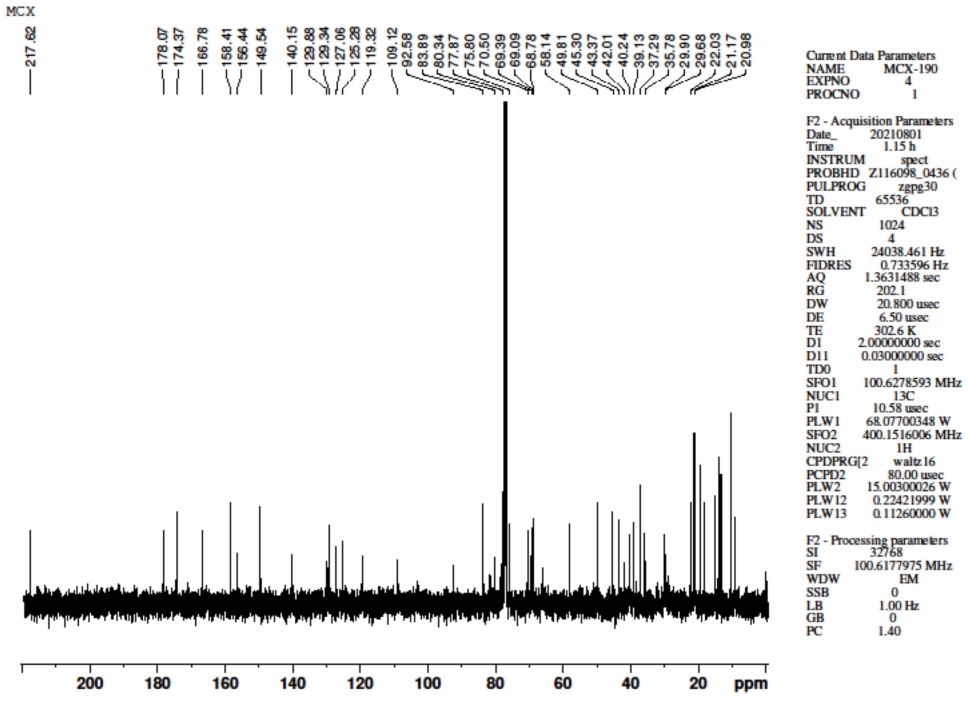
MCX
Current Data Parameters
NAME MCX-190
EXPNO 4
PROCNO 1
F2 - Acquisition Parameters
Date_ 20210801
Time 1.15 h
INSTRUM spect
PROBHD Z116098_0436 (
PULPROG zgpg30
TD 65536
SOLVENT CDCl3
NS 1024
DS 4
SWH 24038.461 Hz
FIDRES 0.733596 Hz
AQ 1.3631488 sec
RG 202.1
DW 20.800 usec
DE 6.50 usec
TE 302.6 K
D1 2.00000000 sec
D11 0.03000000 sec
TD0 1
SFO1 100.6278593 MHz
NUC1 13C
P1 10.58 usec
PLW1 68.07700348 W
SFO2 400.1516006 MHz
NUC2 1H
CPDPRG[2 waltz16
PCPD2 80.00 usec
PLW2 15.00300026 W
PLW12 0.22421999 W
PLW13 0.11260000 W
F2 - Processing parameters
SI 32768
SF 100.6177975 MHz
WDW EM
SSB 0
LB 1.00 Hz
GB 0
PC 1.40
ppm

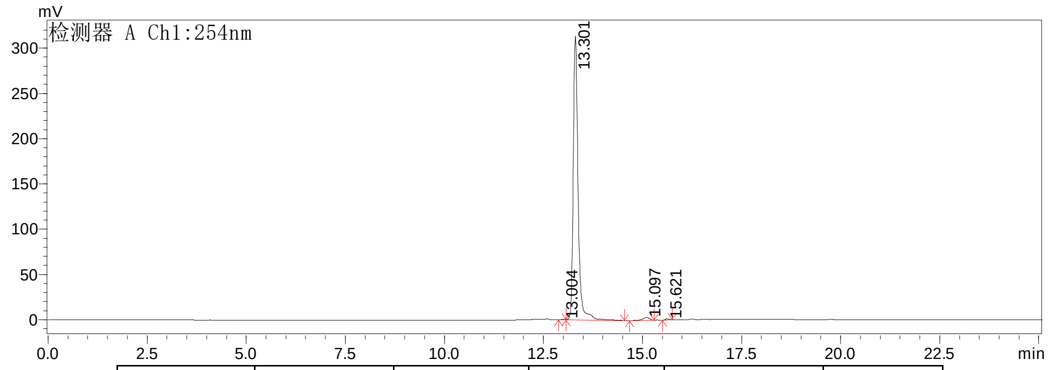

Retention	Peak start	Peak End	peak area	peak height	Area %
13.004	12.883	13.075	4953	638	0.1826
13.301	13.075	14.542	2658872	313577	98.0267
15.097	14.675	15.292	38510	3118	1.4198
15.621	15.500	15.750	10061	1301	0.3709

95i

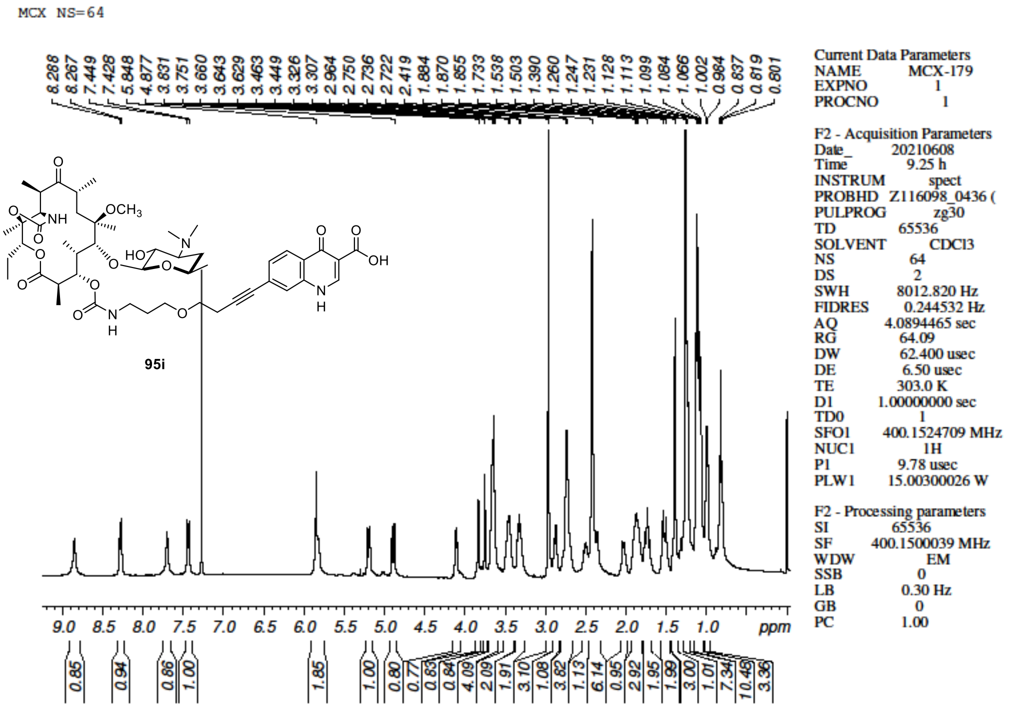
MCX NS=64
8.288 8.267 7.449 7.428 5.848 4.877 3.831 3.751 3.660 3.643 3.629 3.463 3.449 3.326 3.307 2.964 2.750 2.736 2.722 2.419 1.884 1.870 1.855 1.733 1.538 1.503 1.390 1.260 1.247 1.231 1.128 1.113 1.099 1.084 1.066 1.002 0.984 0.837 0.819 0.801
95i
9.0 8.5 8.0 7.5 7.0 6.5 6.0 5.5 5.0 4.5 4.0 3.5 3.0 2.5 2.0 1.5 1.0 ppm
0.85 0.94 0.86 1.00 1.85 1.00 0.80 0.77 0.83 0.84 4.09 2.09 1.91 3.10 1.08 3.82 1.13 6.14 0.95 2.92 1.95 1.99 3.00 1.01 7.34 10.48 3.36
Current Data Parameters
NAME MCX-179
EXPNO 1
PROCNO 1
F2 - Acquisition Parameters
Date_ 20210608
Time 9.25 h
INSTRUM spect
PROBHD Z116098_0436 (
PULPROG zg30
TD 65536
SOLVENT CDCl3
NS 64
DS 2
SWH 8012.820 Hz
FIDRES 0.244532 Hz
AQ 4.0894465 sec
RG 64.09
DW 62.400 usec
DE 6.50 usec
TE 303.0 K
D1 1.00000000 sec
TD0 1
SFO1 400.1524709 MHz
NUC1 1H
P1 9.78 usec
PLW1 15.00300026 W
F2 - Processing parameters
SI 65536
SF 400.1500039 MHz
WDW EM
SSB 0
LB 0.30 Hz
GB 0
PC 1.00

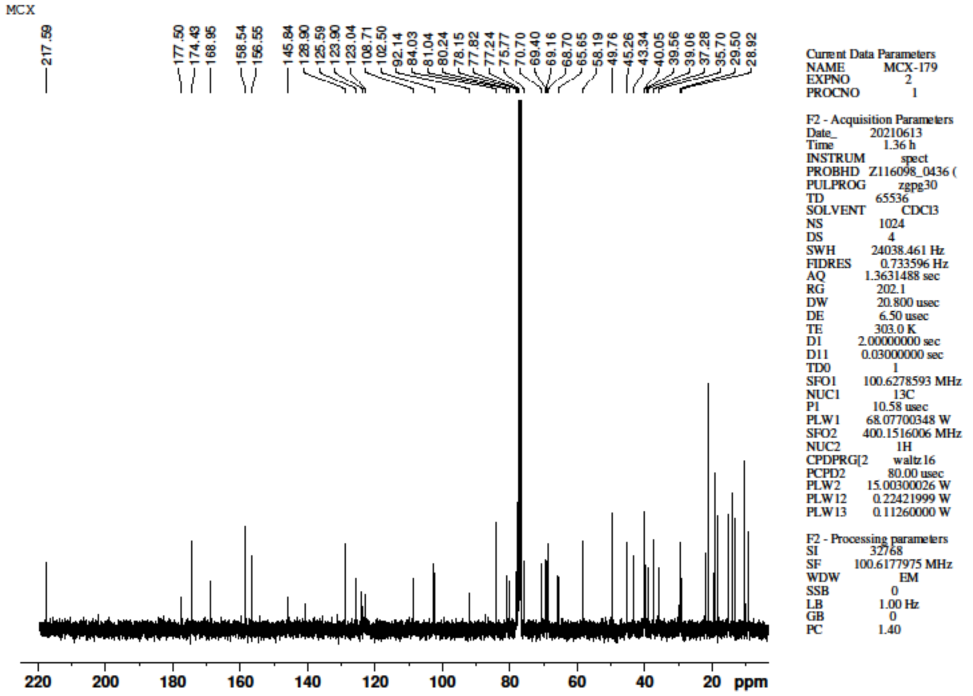
MCX
217.59
177.50 174.43 168.95 158.54 156.55 145.84 128.90 125.59 123.90 123.04 108.71 102.50 92.14 84.03 81.04 80.24 78.15 77.82 77.24 75.77 70.70 69.40 68.16 68.70 65.65 58.19 49.76 45.26 43.34 40.05 39.56 39.06 37.28 35.70 29.50 28.92
220 200 180 160 140 120 100 80 60 40 20 ppm
Current Data Parameters
NAME MCX-179
EXPNO 2
PROCNO 1
F2 - Acquisition Parameters
Date_ 20210613
Time 1.36 h
INSTRUM spect
PROBHD Z116098_0436 (
PULPROG zgpg30
TD 65536
SOLVENT CDCl3
NS 1024
DS 4
SWH 24038.461 Hz
FIDRES 0.733596 Hz
AQ 1.3631488 sec
RG 202.1
DW 20.800 usec
DE 6.50 usec
TE 303.0 K
D1 2.00000000 sec
D11 0.03000000 sec
TD0 1
SFO1 100.6278593 MHz
NUC1 13C
P1 10.58 usec
PLW1 68.07700348 W
SFO2 400.1516006 MHz
NUC2 1H
CPDPRG[2 waltz16
PCPD2 80.00 usec
PLW2 15.00300026 W
PLW12 0.22421999 W
PLW13 0.11260000 W
F2 - Processing parameters
SI 32768
SF 100.6177975 MHz
WDW EM
SSB 0
LB 1.00 Hz
GB 0
PC 1.40

95j

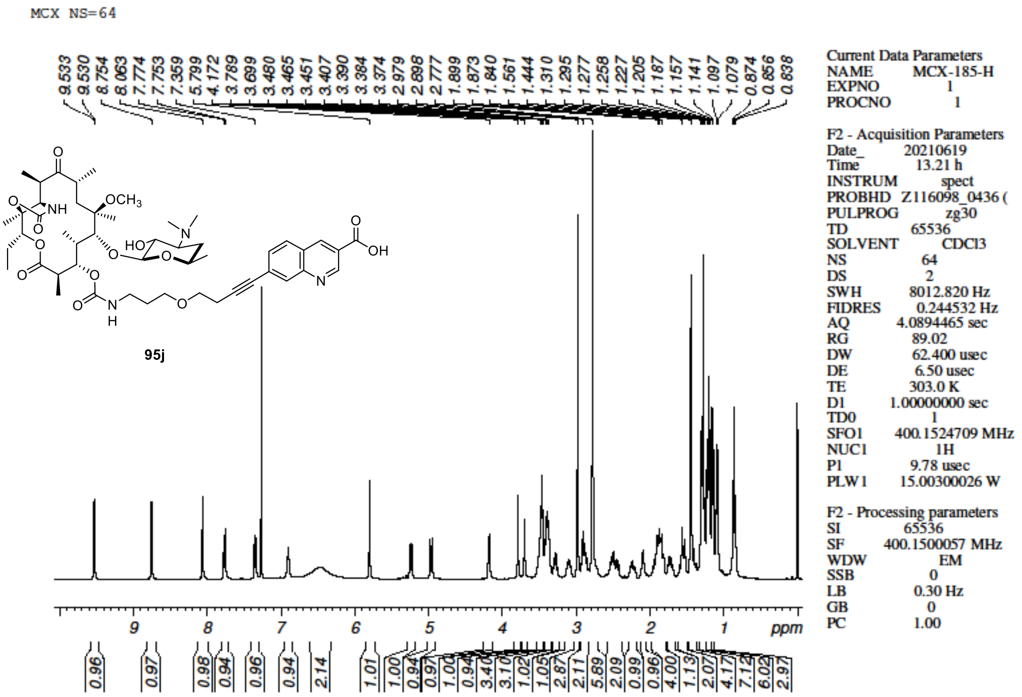
MCX NS=64
95j
Current Data Parameters
NAME MCX-185-H
EXPNO 1
PROCNO 1
F2 - Acquisition Parameters
Date_ 20210619
Time 13.21 h
INSTRUM spect
PROBHD Z116098_0436 (
PULPROG zg30
TD 65536
SOLVENT CDCl3
NS 64
DS 2
SWH 8012.820 Hz
FIDRES 0.244532 Hz
AQ 4.0894465 sec
RG 89.02
DW 62.400 usec
DE 6.50 usec
TE 303.0 K
D1 1.00000000 sec
TD0 1
SFO1 400.1524709 MHz
NUC1 1H
P1 9.78 usec
PLW1 15.00300026 W
F2 - Processing parameters
SI 65536
SF 400.1500057 MHz
WDW EM
SSB 0
LB 0.30 Hz
GB 0
PC 1.00
ppm

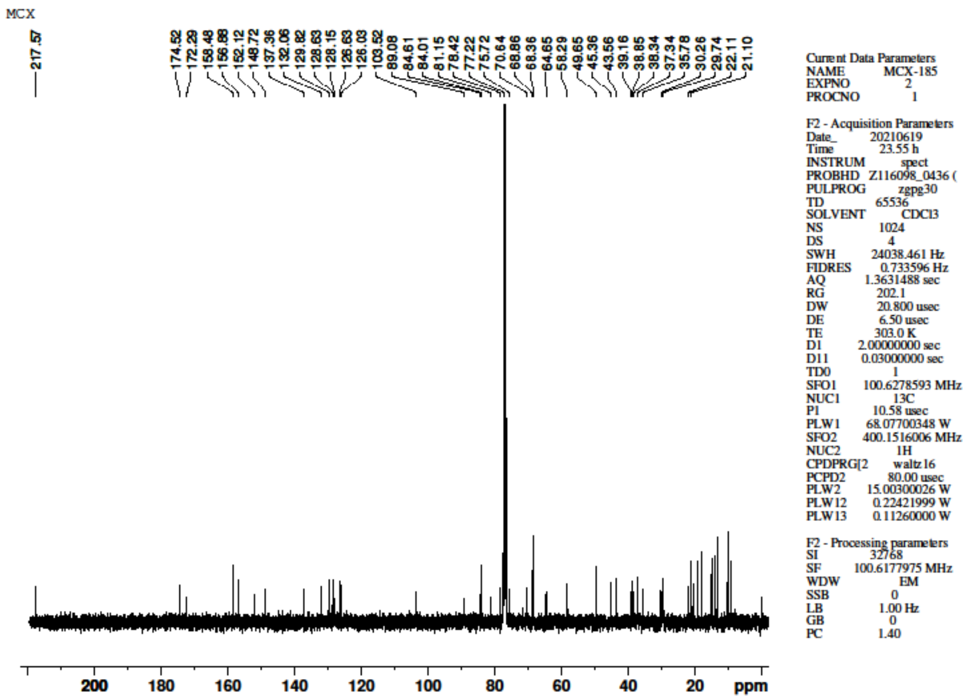
MCX
Current Data Parameters
NAME MCX-185
EXPNO 2
PROCNO 1
F2 - Acquisition Parameters
Date_ 20210619
Time 23.55 h
INSTRUM spect
PROBHD Z116098_0436 (
PULPROG zgpg30
TD 65536
SOLVENT CDCl3
NS 1024
DS 4
SWH 24038.461 Hz
FIDRES 0.733596 Hz
AQ 1.3631488 sec
RG 202.1
DW 20.800 usec
DE 6.50 usec
TE 303.0 K
D1 2.00000000 sec
D11 0.03000000 sec
TD0 1
SFO1 100.6278593 MHz
NUC1 13C
P1 10.58 usec
PLW1 68.07700348 W
SFO2 400.1516006 MHz
NUC2 1H
CPDPRG[2 waltz16
PCPD2 80.00 usec
PLW2 15.00300026 W
PLW12 0.22421999 W
PLW13 0.11260000 W
F2 - Processing parameters
SI 32768
SF 100.6177975 MHz
WDW EM
SSB 0
LB 1.00 Hz
GB 0
PC 1.40
ppm

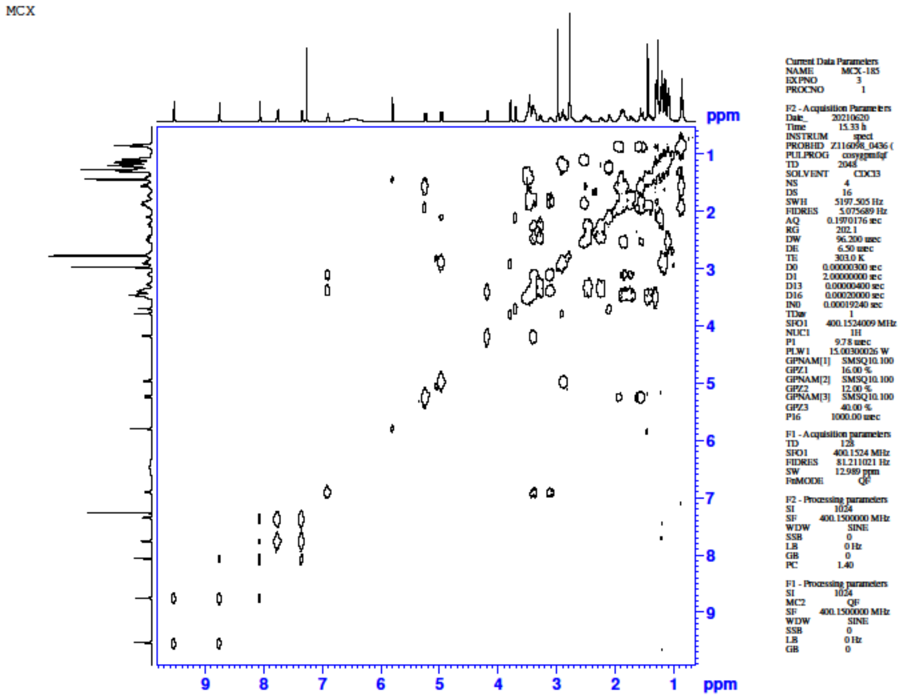
MCX
ppm
1
2
3
4
5
6
7
8
9
9
8
7
6
5
4
3
2
1
ppm
Current Data Parameters
NAME MCX-185
EXPNO 3
PROCNO 1
F2 - Acquisition Parameters
Date_ 20210620
Time 15.33 h
INSTRUM spect
PROBHD Z116098_0436 (
PULPROG cosygpmfqf
TD 2048
SOLVENT CDCl3
NS 4
DS 16
SWH 5197.505 Hz
FIDRES 5.075689 Hz
AQ 0.1970176 sec
RG 202.1
DW 96.200 usec
DE 6.50 usec
TE 303.0 K
D0 0.00000300 sec
D1 2.00000000 sec
D13 0.00000400 sec
D16 0.00020000 sec
IN0 0.00019240 sec
TDav 1
SFO1 400.1524009 MHz
NUC1 1H
P1 9.78 usec
PLW1 15.00300026 W
GPNAM[1] SMSQ10.100
GPZ1 16.00 %
GPNAM[2] SMSQ10.100
GPZ2 12.00 %
GPNAM[3] SMSQ10.100
GPZ3 40.00 %
P16 1000.00 usec
F1 - Acquisition parameters
TD 128
SFO1 400.1524 MHz
FIDRES 81.211021 Hz
SW 12.989 ppm
FnMODE QF
F2 - Processing parameters
SI 1024
SF 400.1500000 MHz
WDW SINE
SSB 0
LB 0 Hz
GB 0
PC 1.40
F1 - Processing parameters
SI 1024
MC2 QF
SF 400.1500000 MHz
WDW SINE
SSB 0
LB 0 Hz
GB 0

96g

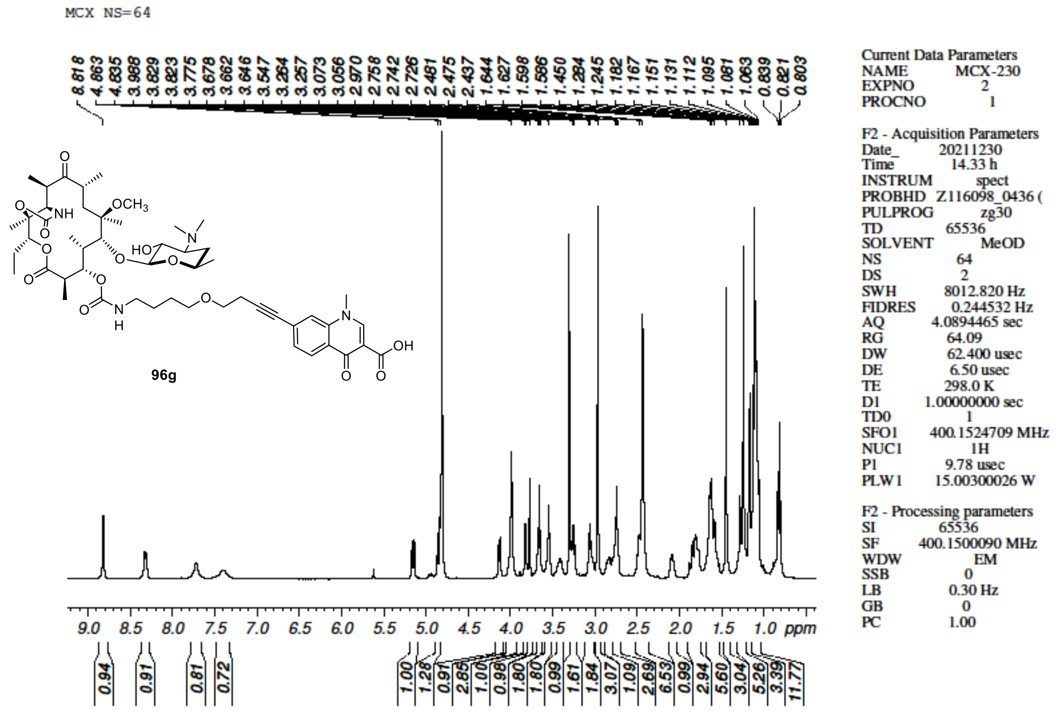
MCX NS=64
8.818
4.863
4.835
3.988
3.829
3.823
3.775
3.678
3.662
3.646
3.547
3.264
3.257
3.073
3.056
2.970
2.758
2.742
2.726
2.481
2.475
2.437
1.644
1.627
1.598
1.586
1.450
1.284
1.245
1.182
1.167
1.151
1.131
1.112
1.095
1.081
1.063
0.839
0.821
0.803
OCH3
NH
HO
OH
96g
9.0 8.5 8.0 7.5 7.0 6.5 6.0 5.5 5.0 4.5 4.0 3.5 3.0 2.5 2.0 1.5 1.0 ppm
0.94
0.91
0.81
0.72
1.00
1.28
0.91
2.85
1.00
0.98
1.80
1.80
0.99
1.61
1.84
3.07
1.09
2.69
6.53
0.99
2.94
5.60
3.04
5.26
3.39
11.77
Current Data Parameters
NAME MCX-230
EXPNO 2
PROCNO 1
F2 - Acquisition Parameters
Date_ 20211230
Time 14.33 h
INSTRUM spect
PROBHD Z116098_0436 (
PULPROG zg30
TD 65536
SOLVENT MeOD
NS 64
DS 2
SWH 8012.820 Hz
FIDRES 0.244532 Hz
AQ 4.0894465 sec
RG 64.09
DW 62.400 usec
DE 6.50 usec
TE 298.0 K
D1 1.00000000 sec
TD0 1
SFO1 400.1524709 MHz
NUC1 1H
P1 9.78 usec
PLW1 15.00300026 W
F2 - Processing parameters
SI 65536
SF 400.1500090 MHz
WDW EM
SSB 0
LB 0.30 Hz
GB 0
PC 1.00

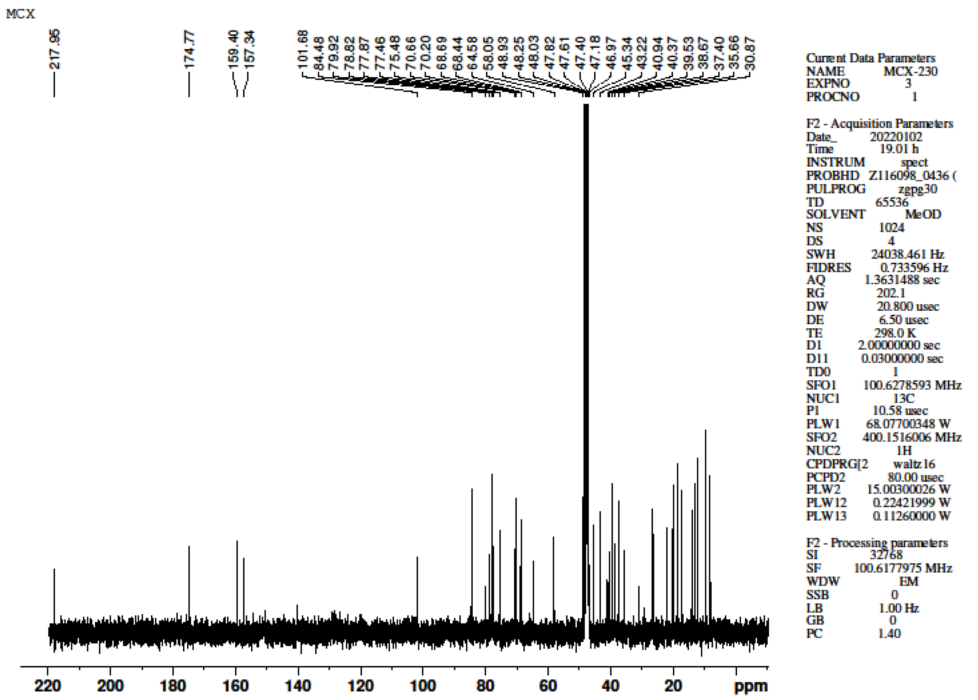
MCX
217.95
174.77
159.40
157.34
101.68
84.48
79.92
78.82
77.87
77.46
75.48
70.66
70.20
68.69
68.44
64.58
58.05
48.93
48.25
48.03
47.82
47.61
47.40
47.18
46.97
45.34
43.22
40.94
40.37
39.53
38.67
37.40
35.66
30.87
220 200 180 160 140 120 100 80 60 40 20 ppm
Current Data Parameters
NAME MCX-230
EXPNO 3
PROCNO 1
F2 - Acquisition Parameters
Date_ 20220102
Time 19.01 h
INSTRUM spect
PROBHD Z116098_0436 (
PULPROG zgpg30
TD 65536
SOLVENT MeOD
NS 1024
DS 4
SWH 24038.461 Hz
FIDRES 0.733596 Hz
AQ 1.3631488 sec
RG 202.1
DW 20.800 usec
DE 6.50 usec
TE 298.0 K
D1 2.00000000 sec
D11 0.03000000 sec
TD0 1
SFO1 100.6278593 MHz
NUC1 13C
P1 10.58 usec
PLW1 68.07700348 W
SFO2 400.1516006 MHz
NUC2 1H
CPDPRG[2 waltz16
PCPD2 80.00 usec
PLW2 15.00300026 W
PLW12 0.22421999 W
PLW13 0.11260000 W
F2 - Processing parameters
SI 32768
SF 100.6177975 MHz
WDW EM
SSB 0
LB 1.00 Hz
GB 0
PC 1.40

98c

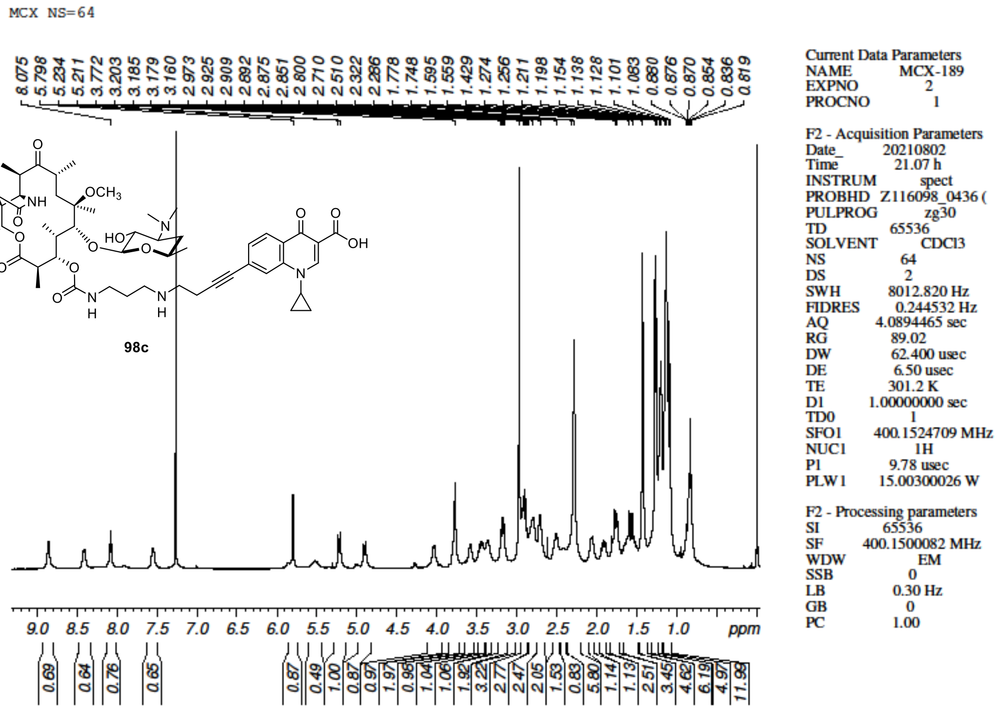
MCX NS=64
8.075 5.798 5.234 5.211 3.772 3.203 3.185 3.179 3.160 2.973 2.925 2.909 2.892 2.875 2.851 2.800 2.710 2.510 2.322 2.286 1.778 1.748 1.595 1.559 1.429 1.274 1.256 1.211 1.198 1.154 1.138 1.128 1.101 1.083 0.880 0.876 0.870 0.854 0.836 0.819
OCH3
NH
HO
OH
98c
9.0 8.5 8.0 7.5 7.0 6.5 6.0 5.5 5.0 4.5 4.0 3.5 3.0 2.5 2.0 1.5 1.0 ppm
0.69 0.64 0.76 0.65 0.87 0.49 1.00 0.87 0.97 1.97 0.95 1.04 1.06 1.92 3.22 2.77 2.47 2.05 1.53 0.83 5.80 1.14 1.13 2.51 3.45 4.62 6.19 4.97 11.98
Current Data Parameters
NAME MCX-189
EXPNO 2
PROCNO 1
F2 - Acquisition Parameters
Date_ 20210802
Time 21.07 h
INSTRUM spect
PROBHD Z116098_0436 (
PULPROG zg30
TD 65536
SOLVENT CDCl3
NS 64
DS 2
SWH 8012.820 Hz
FIDRES 0.244532 Hz
AQ 4.0894465 sec
RG 89.02
DW 62.400 usec
DE 6.50 usec
TE 301.2 K
D1 1.00000000 sec
TD0 1
SFO1 400.1524709 MHz
NUC1 1H
P1 9.78 usec
PLW1 15.00300026 W
F2 - Processing parameters
SI 65536
SF 400.1500082 MHz
WDW EM
SSB 0
LB 0.30 Hz
GB 0
PC 1.00

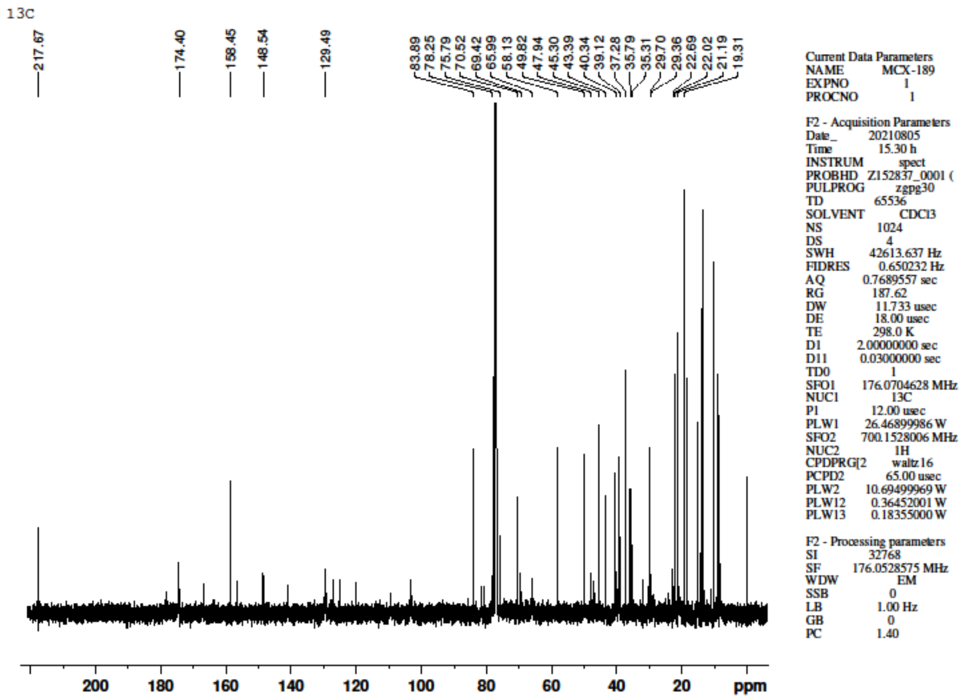
13C
217.67 174.40 158.45 148.54 129.49 83.89 78.25 75.79 70.52 69.42 65.99 58.13 49.82 47.94 45.30 43.39 40.34 39.12 37.28 35.79 35.31 29.70 29.36 22.69 22.02 21.19 19.31
200 180 160 140 120 100 80 60 40 20 ppm
Current Data Parameters
NAME MCX-189
EXPNO 1
PROCNO 1
F2 - Acquisition Parameters
Date_ 20210805
Time 15.30 h
INSTRUM spect
PROBHD Z152837_0001 (
PULPROG zgpg30
TD 65536
SOLVENT CDCl3
NS 1024
DS 4
SWH 42613.637 Hz
FIDRES 0.650232 Hz
AQ 0.7689557 sec
RG 187.62
DW 11.733 usec
DE 18.00 usec
TE 298.0 K
D1 2.00000000 sec
D11 0.03000000 sec
TD0 1
SFO1 176.0704628 MHz
NUC1 13C
P1 12.00 usec
PLW1 26.46899986 W
SFO2 700.1528006 MHz
NUC2 1H
CPDPRG[2 waltz16
PCPD2 65.00 usec
PLW2 10.69499969 W
PLW12 0.36452001 W
PLW13 0.18355000 W
F2 - Processing parameters
SI 32768
SF 176.0528575 MHz
WDW EM
SSB 0
LB 1.00 Hz
GB 0
PC 1.40

106g

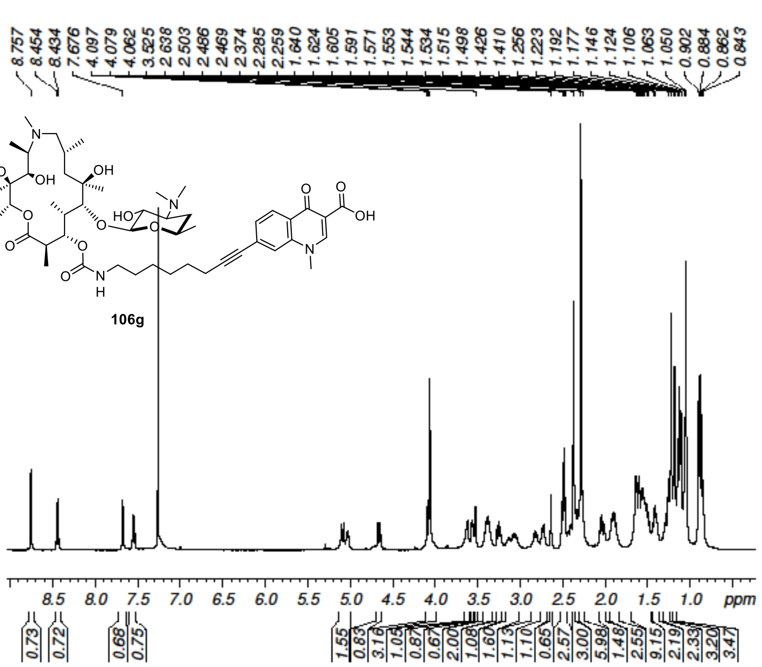

Current Data Parameters
NAME LXT-69
EXPNO 2
PROCNO 1

F2 - Acquisition Parameters
Date_ 20211223
Time 10.06 h
INSTRUM spect
PROBHD Z116098_0436 (
PULPROG zg30
TD 65536
SOLVENT CDCl3
NS 64
DS 2
SWH 8012.820 Hz
FIDRES 0.244532 Hz
AQ 4.0894465 sec
RG 98.25
DW 62.400 usec
DE 6.50 usec
TE 298.0 K
D1 1.00000000 sec
TD0 1
SFO1 400.1524709 MHz
NUC1 1H
P1 9.78 usec
PLW1 15.00300026 W

F2 - Processing parameters
SI 65536
SF 400.1500092 MHz
WDW EM
SSB 0
LB 0.30 Hz
GB 0
PC 1.00

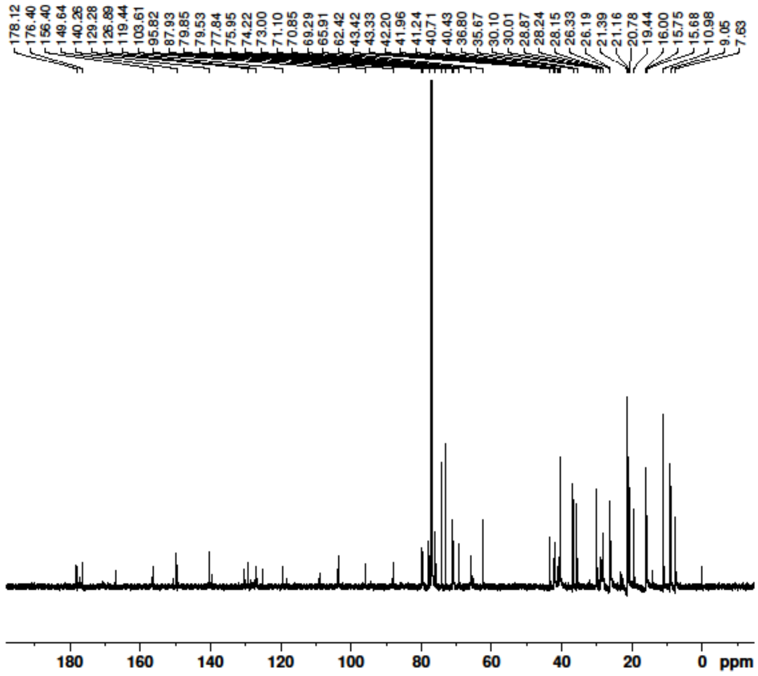

Current Data Parameters
NAME LXT-69-1
EXPNO 1
PROCNO 1

F2 - Acquisition Parameters
Date_ 20220330
Time 8.28 h
INSTRUM spect
PROBHD Z152837_0001 (
PULPROG zgpg30
TD 65536
SOLVENT CDCl3
NS 943
DS 4
SWH 42613.637 Hz
FIDRES 0.650232 Hz
AQ 0.7689557 sec
RG 187.62
DW 11.733 usec
DE 18.00 usec
TE 298.0 K
D1 2.00000000 sec
D11 0.03000000 sec
TD0 1
SFO1 176.0704628 MHz
NUC1 13C
P1 12.00 usec
PLW1 26.46899986 W
SFO2 700.1528006 MHz
NUC2 1H
CPDPRG[2 waltz16
PCPD2 65.00 usec
PLW2 10.69499969 W
PLW12 0.36452001 W
PLW13 0.18355000 W

F2 - Processing parameters
SI 32768
SF 176.0528575 MHz
WDW EM
SSB 0
LB 1.00 Hz
GB 0
PC 1.40

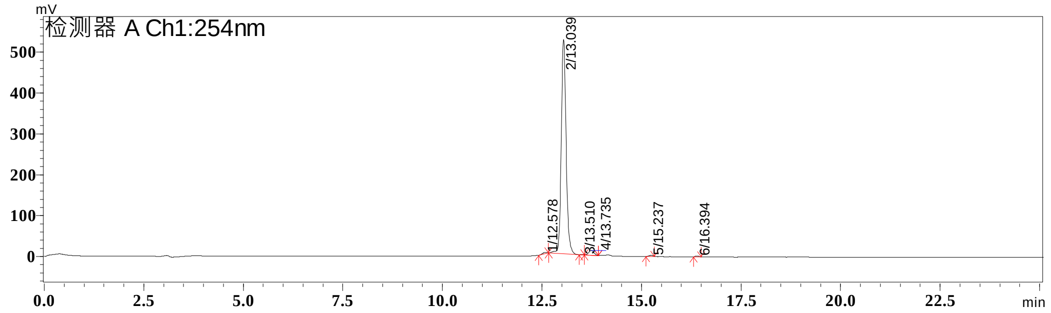

Retention	Peak start	Peak End	peak area	peak height	Area %
12.578	12.417	12.658	22053	2925	0.4846
13.039	12.667	13.917	4384982	523935	96.3500
13.510	13.433	13.567	4005	651	0.0880
13.735	13.567	13.917	107158	11479	2.3545
15.237	15.117	15.325	18376	2881	0.4038
16.394	16.308	16.500	14522	2326	0.3191

107g

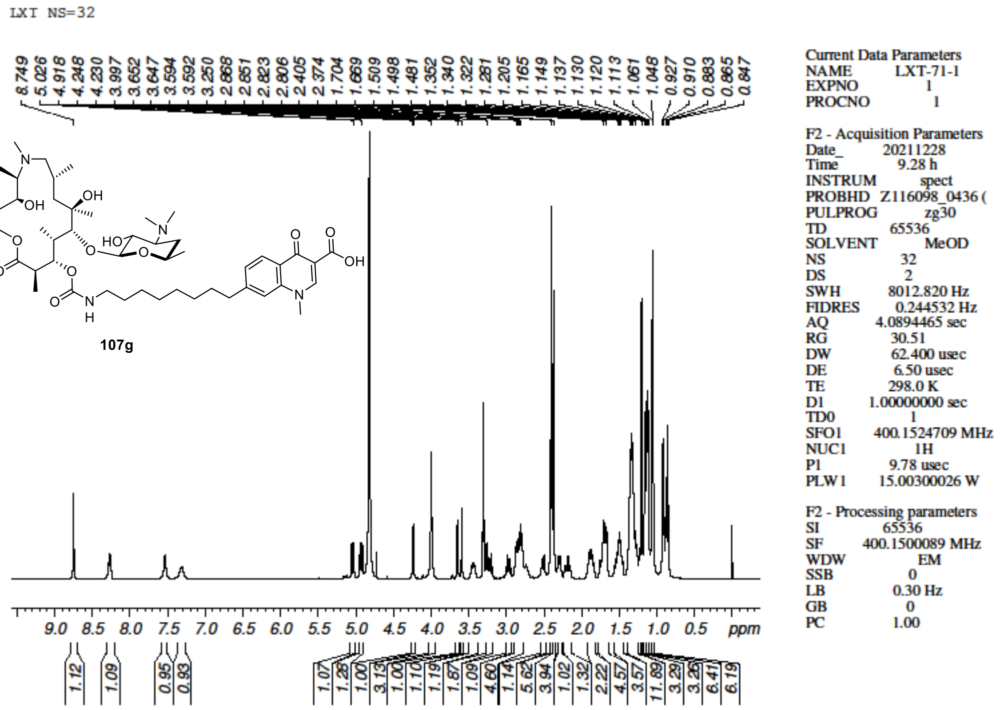
LXT NS=32
8.749 5.026 4.918 4.248 4.230 3.997 3.652 3.647 3.594 3.592 3.250 2.868 2.851 2.823 2.806 2.405 2.374 1.704 1.669 1.509 1.498 1.481 1.352 1.340 1.322 1.281 1.205 1.165 1.149 1.137 1.130 1.120 1.113 1.061 1.048 0.927 0.910 0.883 0.865 0.847
107g
9.0 8.5 8.0 7.5 7.0 6.5 6.0 5.5 5.0 4.5 4.0 3.5 3.0 2.5 2.0 1.5 1.0 0.5 ppm
1.12 1.09 0.95 0.93 1.07 1.28 1.00 3.13 1.00 1.10 1.19 1.87 1.09 4.60 1.14 5.62 3.94 1.02 1.32 2.22 4.57 3.57 11.89 3.29 3.28 6.41 6.19
Current Data Parameters
NAME LXT-71-1
EXPNO 1
PROCNO 1
F2 - Acquisition Parameters
Date_ 20211228
Time 9.28 h
INSTRUM spect
PROBHD Z116098_0436 (
PULPROG zg30
TD 65536
SOLVENT MeOD
NS 32
DS 2
SWH 8012.820 Hz
FIDRES 0.244532 Hz
AQ 4.0894465 sec
RG 30.51
DW 62.400 usec
DE 6.50 usec
TE 298.0 K
D1 1.00000000 sec
TD0 1
SFO1 400.1524709 MHz
NUC1 1H
P1 9.78 usec
PLW1 15.00300026 W
F2 - Processing parameters
SI 65536
SF 400.1500089 MHz
WDW EM
SSB 0
LB 0.30 Hz
GB 0
PC 1.00

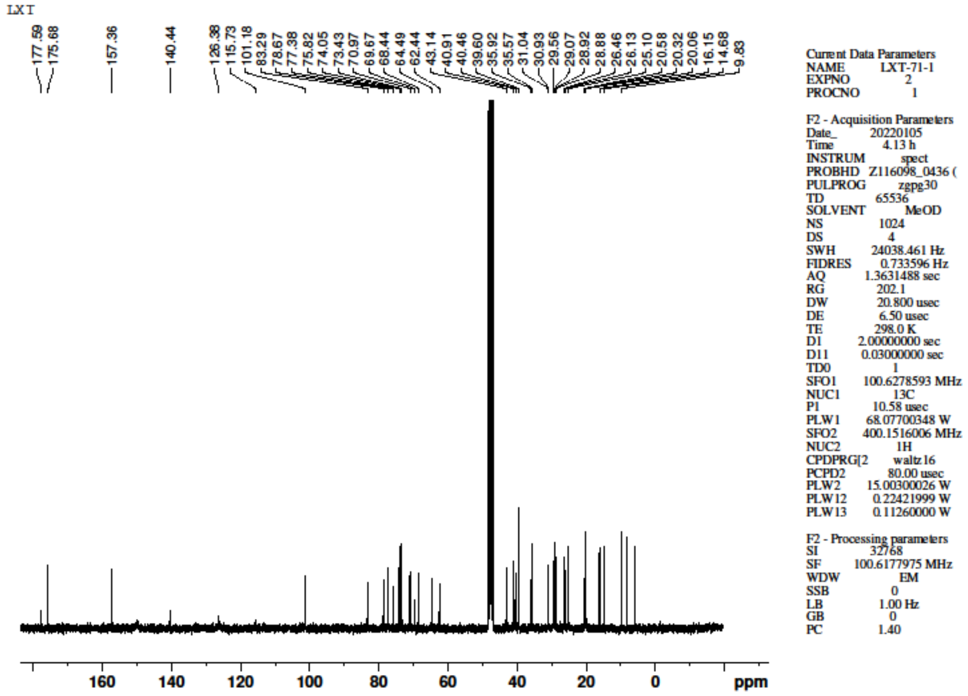
LXT
177.59 175.68 157.36 140.44 126.38 115.73 101.18 83.29 78.67 77.38 75.82 74.05 73.43 70.97 69.67 68.44 64.49 62.44 43.14 40.91 40.46 39.60 35.92 35.57 31.04 30.93 29.56 29.07 28.92 28.88 26.46 26.13 25.10 20.58 20.32 20.06 16.15 14.68 9.83
160 140 120 100 80 60 40 20 0 ppm
Current Data Parameters
NAME LXT-71-1
EXPNO 2
PROCNO 1
F2 - Acquisition Parameters
Date_ 20220105
Time 4.13 h
INSTRUM spect
PROBHD Z116098_0436 (
PULPROG zgpg30
TD 65536
SOLVENT MeOD
NS 1024
DS 4
SWH 24038.461 Hz
FIDRES 0.733596 Hz
AQ 1.3631488 sec
RG 202.1
DW 20.800 usec
DE 6.50 usec
TE 298.0 K
D1 2.00000000 sec
D11 0.03000000 sec
TD0 1
SFO1 100.6278593 MHz
NUC1 13C
P1 10.58 usec
PLW1 68.07700348 W
SFO2 400.1516006 MHz
NUC2 1H
CPDPRG[2 waltz16
PCPD2 80.00 usec
PLW2 15.00300026 W
PLW12 0.22421999 W
PLW13 0.11260000 W
F2 - Processing parameters
SI 32768
SF 100.6177975 MHz
WDW EM
SSB 0
LB 1.00 Hz
GB 0
PC 1.40

108c

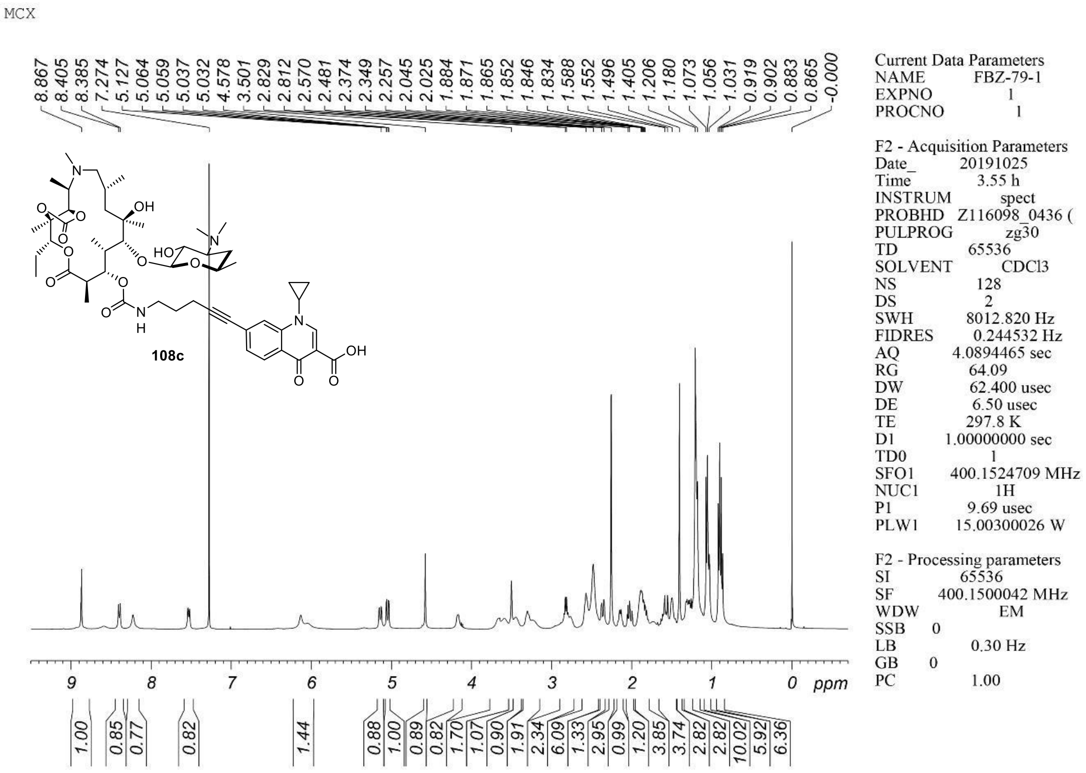
MCX
8.867
8.405
8.385
7.274
5.127
5.064
5.059
5.037
5.032
4.578
3.501
2.829
2.812
2.570
2.481
2.374
2.349
2.257
2.045
2.025
1.884
1.871
1.865
1.852
1.846
1.834
1.588
1.552
1.496
1.405
1.206
1.180
1.073
1.056
1.031
0.919
0.902
0.883
0.865
-0.000
108c
OH
HO
N
O
H
OH
9
8
7
6
5
4
3
2
1
0
ppm
1.00
0.85
0.77
0.82
1.44
0.88
1.00
0.89
0.82
1.70
1.07
0.90
1.91
2.34
6.09
1.33
2.95
0.99
1.20
3.85
3.74
2.82
2.82
10.02
5.92
6.36
Current Data Parameters
NAME FBZ-79-1
EXPNO 1
PROCNO 1
F2 - Acquisition Parameters
Date_ 20191025
Time 3.55 h
INSTRUM spect
PROBHD Z116098_0436 (
PULPROG zg30
TD 65536
SOLVENT CDCl3
NS 128
DS 2
SWH 8012.820 Hz
FIDRES 0.244532 Hz
AQ 4.0894465 sec
RG 64.09
DW 62.400 usec
DE 6.50 usec
TE 297.8 K
D1 1.00000000 sec
TD0 1
SFO1 400.1524709 MHz
NUC1 1H
P1 9.69 usec
PLW1 15.00300026 W
F2 - Processing parameters
SI 65536
SF 400.1500042 MHz
WDW EM
SSB 0
LB 0.30 Hz
GB 0
PC 1.00

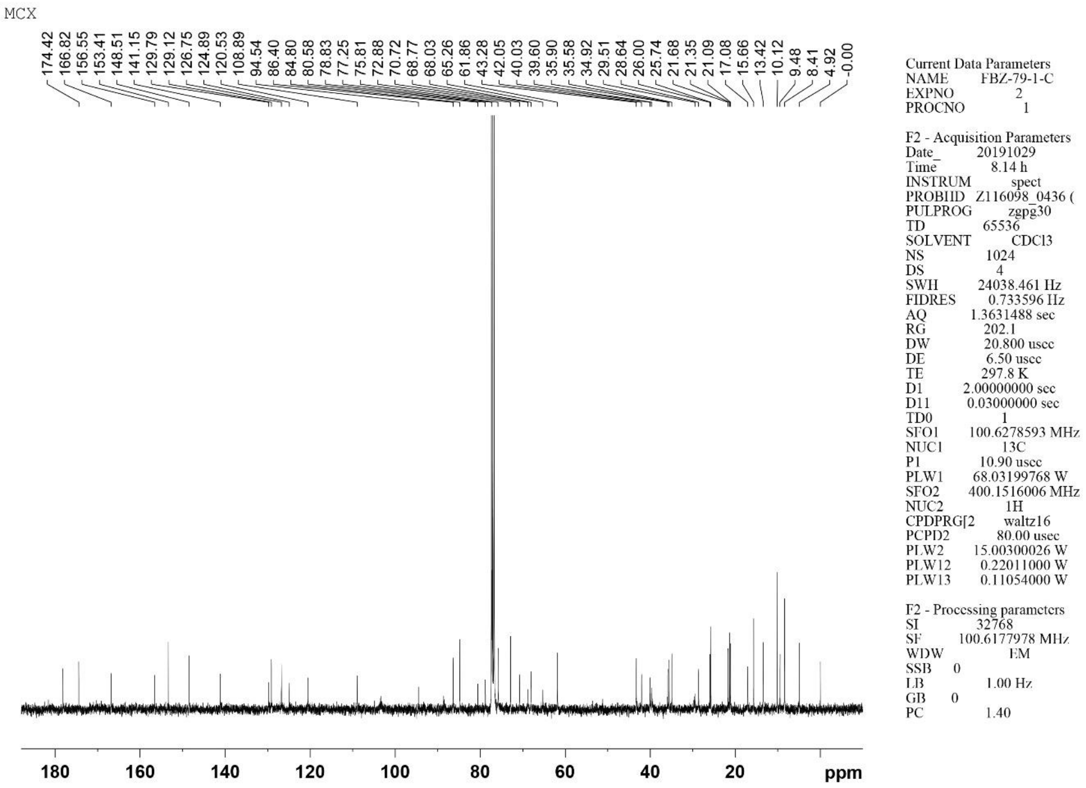
MCX
174.42
166.82
156.55
153.41
148.51
141.15
129.79
129.12
126.75
124.89
120.53
108.89
94.54
86.40
84.80
80.58
78.83
77.25
75.81
72.88
70.72
68.77
68.03
65.26
61.86
43.28
42.05
40.03
39.60
35.90
35.58
34.92
29.51
28.64
26.00
25.74
21.68
21.35
21.09
17.08
15.66
13.42
10.12
9.48
8.41
4.92
-0.00
180
160
140
120
100
80
60
40
20
ppm
Current Data Parameters
NAME FBZ-79-1-C
EXPNO 2
PROCNO 1
F2 - Acquisition Parameters
Date_ 20191029
Time 8.14 h
INSTRUM spect
PROBHD Z116098_0436 (
PULPROG zgpg30
TD 65536
SOLVENT CDCl3
NS 1024
DS 4
SWH 24038.461 Hz
FIDRES 0.733596 Hz
AQ 1.3631488 sec
RG 202.1
DW 20.800 usec
DE 6.50 usec
TE 297.8 K
D1 2.00000000 sec
D11 0.03000000 sec
TD0 1
SFO1 100.6278593 MHz
NUC1 13C
P1 10.90 usec
PLW1 68.03199768 W
SFO2 400.1516006 MHz
NUC2 1H
CPDPRG[2 waltz16
PCPD2 80.00 usec
PLW2 15.00300026 W
PLW12 0.22011000 W
PLW13 0.11054000 W
F2 - Processing parameters
SI 32768
SF 100.6177978 MHz
WDW EM
SSB 0
LB 1.00 Hz
GB 0
PC 1.40

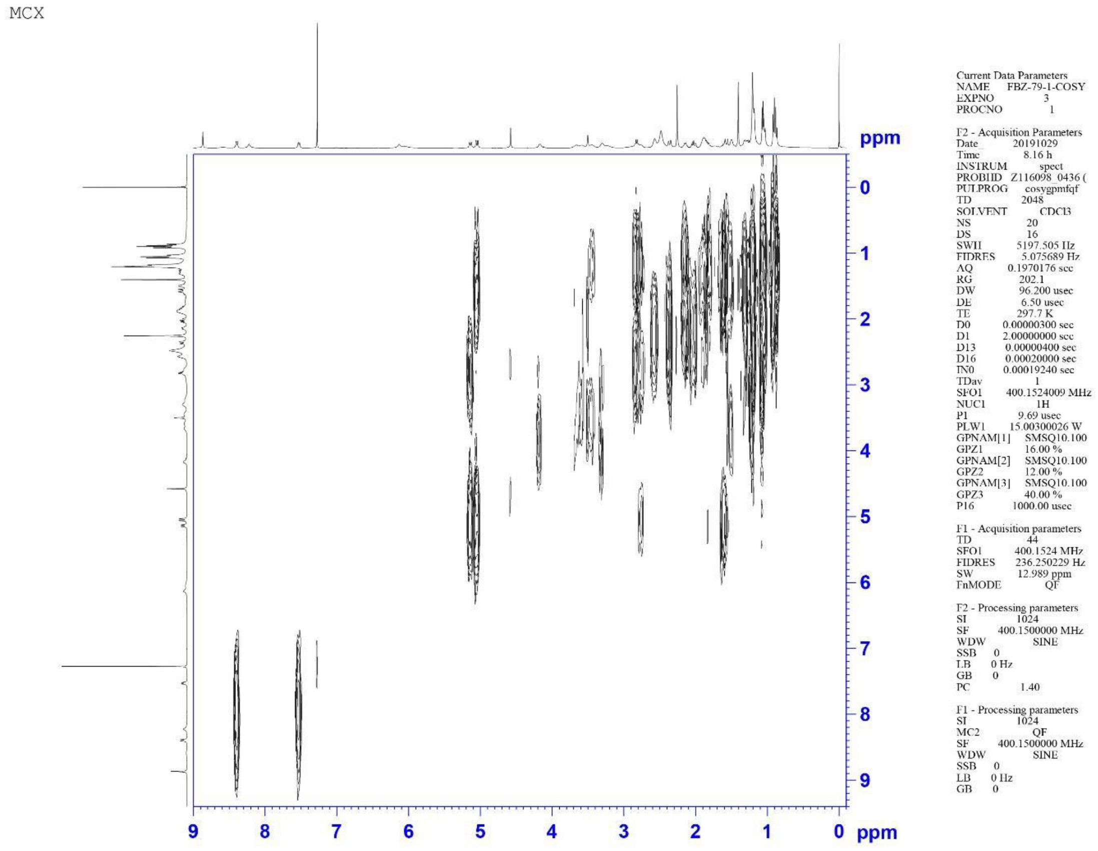
MCX
ppm
0
1
2
3
4
5
6
7
8
9
9 8 7 6 5 4 3 2 1 0 ppm
Current Data Parameters
NAME FBZ-79-1-COSY
EXPNO 3
PROCNO 1
F2 - Acquisition Parameters
Date_ 20191029
Time 8.16 h
INSTRUM spect
PROBHD Z116098_0436 (
PULPROG cosygpmfqf
TD 2048
SOLVENT CDCl3
NS 20
DS 16
SWH 5197.505 Hz
FIDRES 5.075689 Hz
AQ 0.1970176 sec
RG 202.1
DW 96.200 usec
DE 6.50 usec
TE 297.7 K
D0 0.00000300 sec
D1 2.00000000 sec
D13 0.00000400 sec
D16 0.00020000 sec
IN0 0.00019240 sec
TDav 1
SFO1 400.1524009 MHz
NUC1 1H
P1 9.69 usec
PLW1 15.00300026 W
GPNAM[1] SMSQ10.100
GPZ1 16.00 %
GPNAM[2] SMSQ10.100
GPZ2 12.00 %
GPNAM[3] SMSQ10.100
GPZ3 40.00 %
P16 1000.00 usec
F1 - Acquisition parameters
TD 44
SFO1 400.1524 MHz
FIDRES 236.250229 Hz
SW 12.989 ppm
FnMODE QF
F2 - Processing parameters
SI 1024
SF 400.1500000 MHz
WDW SINE
SSB 0
LB 0 Hz
GB 0
PC 1.40
F1 - Processing parameters
SI 1024
MC2 QF
SF 400.1500000 MHz
WDW SINE
SSB 0
LB 0 Hz
GB 0

109a

FBZ

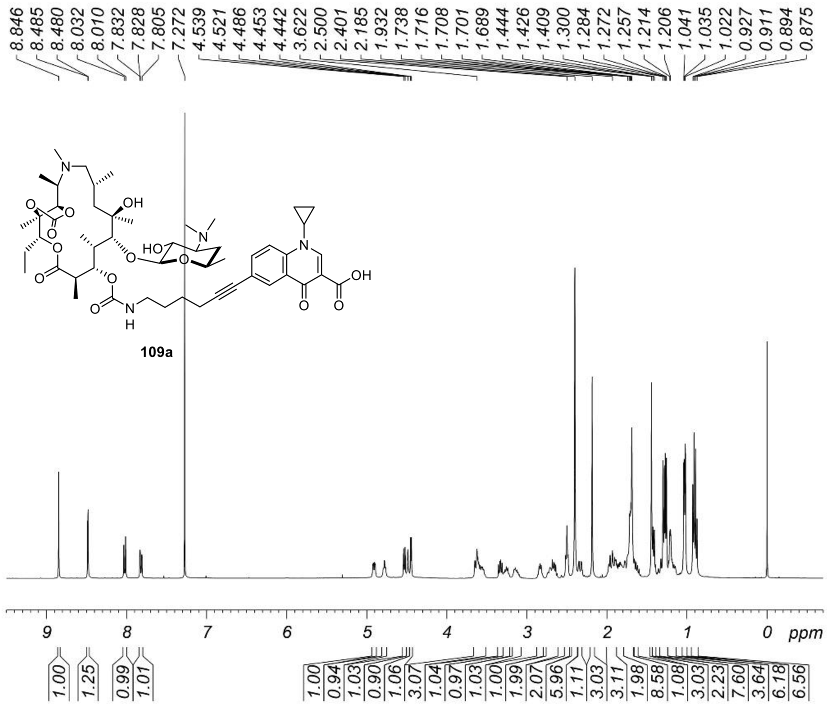

Current Data Parameters
NAME FBZ-86-1
EXPNO 1
PROCNO 1

F2 - Acquisition Parameters
Date_ 20191209
Time 11.27 h
INSTRUM spect
PROBHD Z116098_0436 (
PULPROG zg30
TD 65536
SOLVENT CDCl3
NS 60
DS 2
SWH 8012.820 Hz
FIDRES 0.244532 Hz
AQ 4.0894465 sec
RG 64.09
DW 62.400 usec
DE 6.50 usec
TE 297.8 K
D1 1.00000000 sec
TD0 1
SFO1 400.1524709 MHz
NUC1 1H
P1 9.69 usec
PLW1 15.00300026 W

F2 - Processing parameters
SI 65536
SF 400.1500049 MHz
WDW EM
SSB 0
LB 0.30 Hz
GB 0
PC 1.00

FBZ

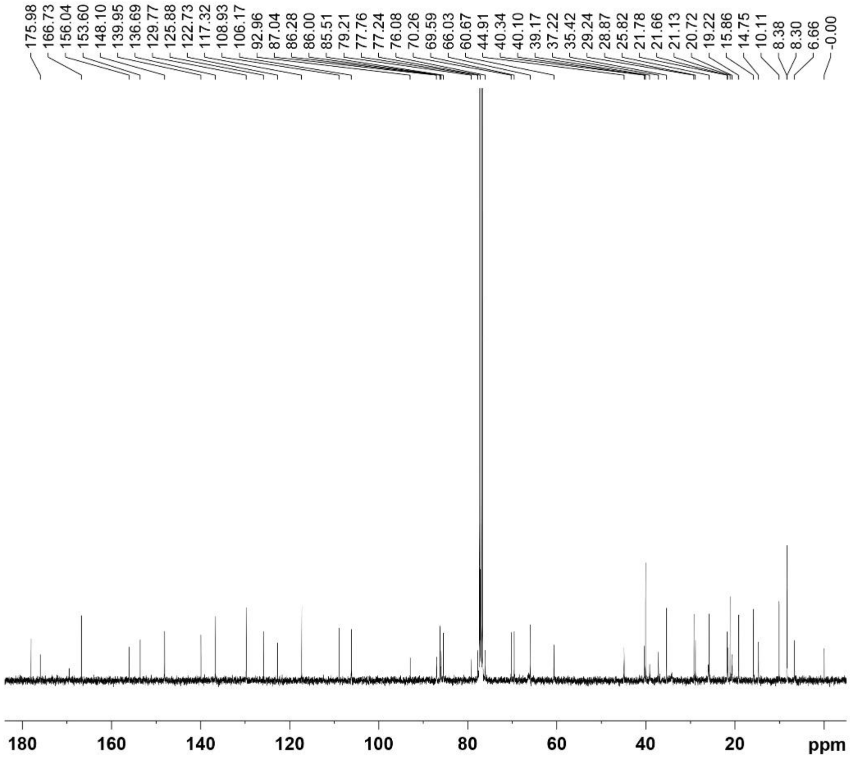

Current Data Parameters
NAME FBZ-86-1-C
EXPNO 2
PROCNO 1

F2 - Acquisition Parameters
Date_ 20191212
Time 1.23 h
INSTRUM spect
PROBHD Z116098_0436 (
PULPROG zgpg30
TD 65536
SOLVENT CDCl3
NS 1024
DS 4
SWH 24038.461 Hz
FIDRES 0.733596 Hz
AQ 1.3631488 sec
RG 202.1
DW 20.800 usec
DE 6.50 usec
TE 297.8 K
D1 2.00000000 sec
D11 0.03000000 sec
TD0 1
SFO1 100.6278593 MHz
NUC1 13C
P1 10.90 usec
PLW1 68.03199768 W
SFO2 400.1516006 MHz
NUC2 1H
CPDPRG[2 waltz16
PCPD2 80.00 usec
PLW2 15.00300026 W
PLW12 0.22011000 W
PLW13 0.11054000 W

F2 - Processing parameters
SI 32768
SF 100.6177979 MHz
WDW EM
SSB 0
LB 1.00 Hz
GB 0
PC 1.40

110a

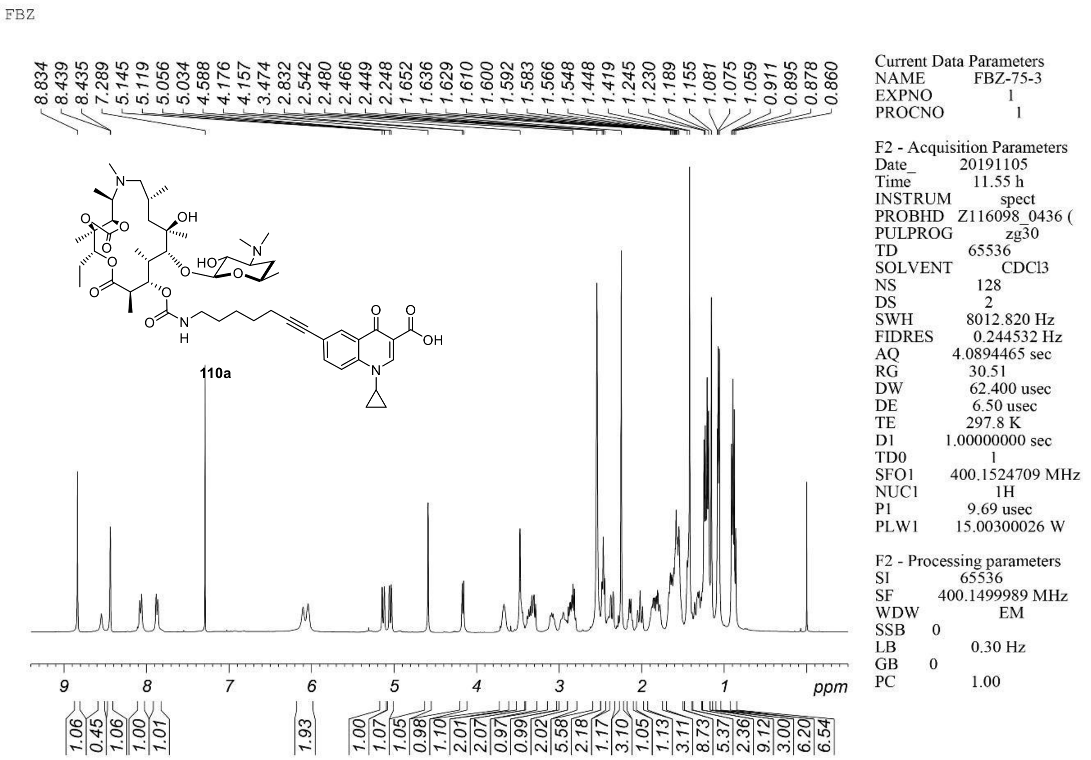
FBZ
8.834
8.439
8.435
7.289
5.145
5.119
5.056
5.034
4.588
4.176
4.157
3.474
2.832
2.542
2.480
2.466
2.449
2.248
1.652
1.636
1.629
1.610
1.600
1.592
1.583
1.566
1.548
1.448
1.419
1.245
1.230
1.189
1.155
1.081
1.075
1.059
0.911
0.895
0.878
0.860
110a
OH
HO
OH
O
N
H
9
8
7
6
5
4
3
2
1
ppm
1.06
0.45
1.06
1.00
1.01
1.93
1.00
1.07
1.05
0.98
1.10
2.01
2.07
0.97
0.99
2.02
5.58
2.18
1.17
3.10
1.05
1.13
3.11
8.73
5.37
2.36
9.12
3.00
6.20
6.54
Current Data Parameters
NAME FBZ-75-3
EXPNO 1
PROCNO 1
F2 - Acquisition Parameters
Date_ 20191105
Time 11.55 h
INSTRUM spect
PROBHD Z116098_0436 (
PULPROG zg30
TD 65536
SOLVENT CDCl3
NS 128
DS 2
SWH 8012.820 Hz
FIDRES 0.244532 Hz
AQ 4.0894465 sec
RG 30.51
DW 62.400 usec
DE 6.50 usec
TE 297.8 K
D1 1.00000000 sec
TD0 1
SFO1 400.1524709 MHz
NUC1 1H
P1 9.69 usec
PLW1 15.00300026 W
F2 - Processing parameters
SI 65536
SF 400.1499989 MHz
WDW EM
SSB 0
LB 0.30 Hz
GB 0
PC 1.00

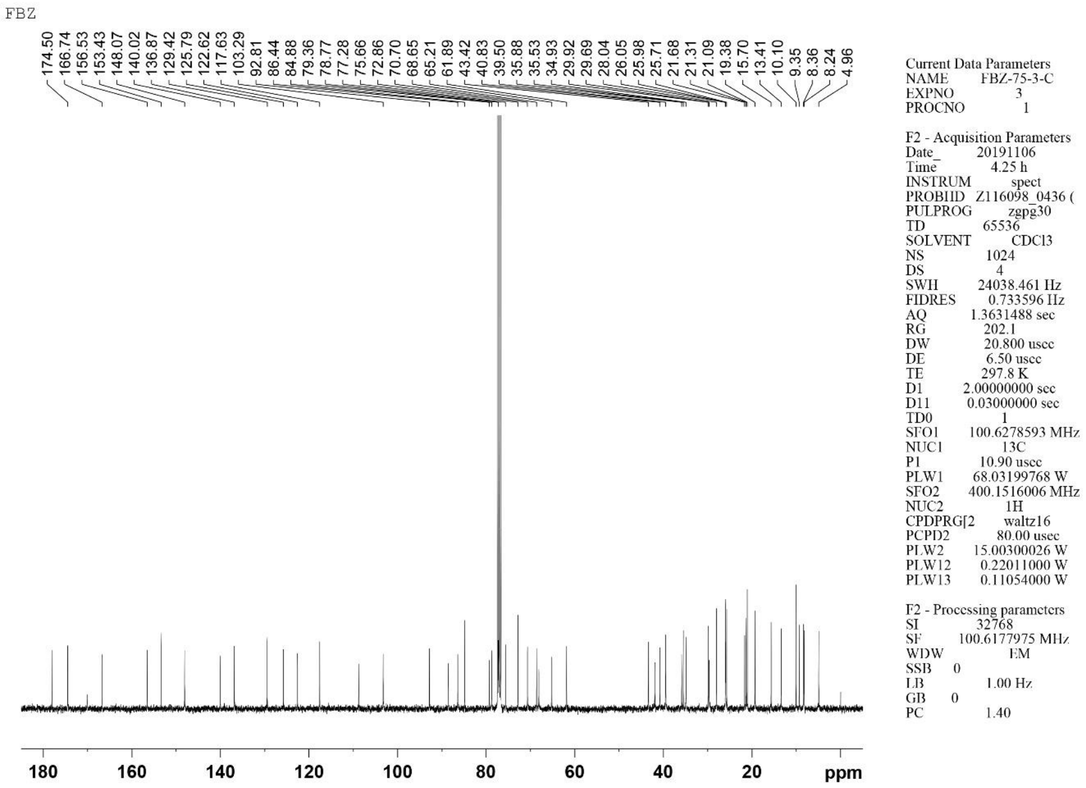
FBZ
174.50
166.74
156.53
153.43
148.07
140.02
136.87
129.42
125.79
122.62
117.63
103.29
92.81
86.44
84.88
79.36
78.77
77.28
75.66
72.86
70.70
68.65
65.21
61.89
43.42
40.83
39.50
35.88
35.53
34.93
29.92
29.69
28.04
26.05
25.98
25.71
21.68
21.31
21.09
19.38
15.70
13.41
10.10
9.35
8.36
8.24
4.96
180
160
140
120
100
80
60
40
20
ppm
Current Data Parameters
NAME FBZ-75-3-C
EXPNO 3
PROCNO 1
F2 - Acquisition Parameters
Date_ 20191106
Time 4.25 h
INSTRUM spect
PROBHD Z116098_0436 (
PULPROG zgpg30
TD 65536
SOLVENT CDCl3
NS 1024
DS 4
SWH 24038.461 Hz
FIDRES 0.733596 Hz
AQ 1.3631488 sec
RG 202.1
DW 20.800 usec
DE 6.50 usec
TE 297.8 K
D1 2.00000000 sec
D11 0.03000000 sec
TD0 1
SFO1 100.6278593 MHz
NUC1 13C
P1 10.90 usec
PLW1 68.03199768 W
SFO2 400.1516006 MHz
NUC2 1H
CPDPRG[2 waltz16
PCPD2 80.00 usec
PLW2 15.00300026 W
PLW12 0.22011000 W
PLW13 0.11054000 W
F2 - Processing parameters
SI 32768
SF 100.6177975 MHz
WDW EM
SSB 0
LB 1.00 Hz
GB 0
PC 1.40

FBZ

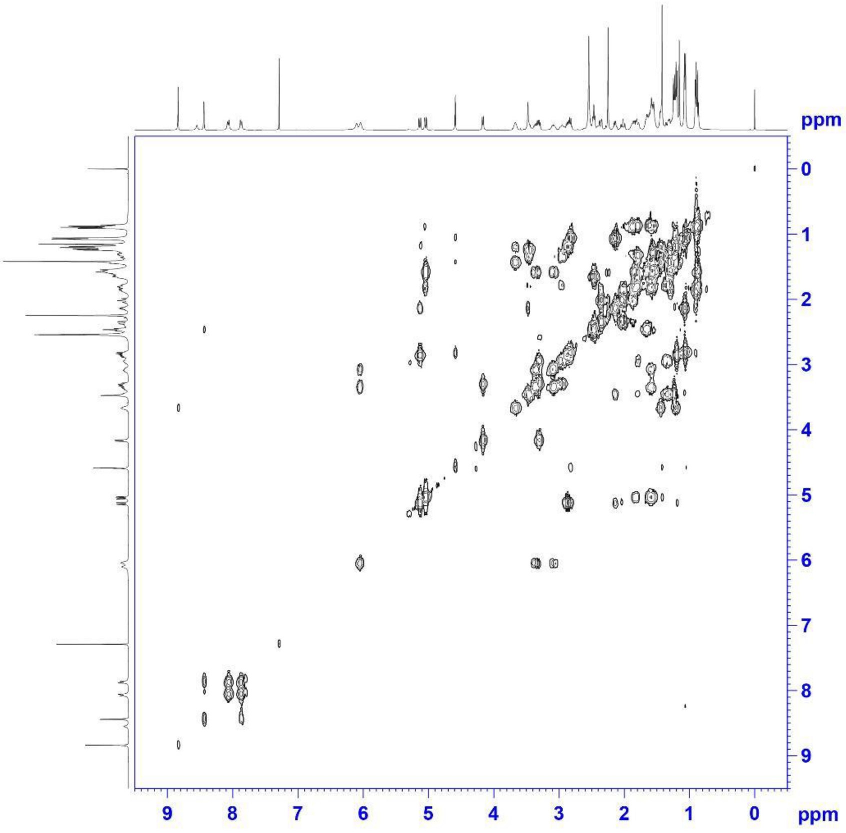

Current Data Parameters
NAME FBZ-75-3-COSY
EXPNO 2
PROCNO 1

F2 - Acquisition Parameters
Date_ 20191106
Time 2.29 h
INSTRUM spect
PROBHD Z116098_0436 (
PULPROG cosygpmfqf
TD 2048
SOLVENT CDCl3
NS 12
DS 16
SWH 5197.505 Hz
FIDRES 5.075689 Hz
AQ 0.1970176 sec
RG 202.1
DW 96.200 usec
DE 6.50 usec
TE 297.8 K
D0 0.00000300 sec
D1 2.00000000 sec
D13 0.00000400 sec
D16 0.00020000 sec
IN0 0.00019240 sec
TDav 1
SFO1 400.1524009 MHz
NUC1 1H
P1 9.69 usec
PLW1 15.00300026 W
GPNAM[1] SMSQ10.100
GPZ1 16.00 %
GPNAM[2] SMSQ10.100
GPZ2 12.00 %
GPNAM[3] SMSQ10.100
GPZ3 40.00 %
P16 1000.00 usec

F1 - Acquisition parameters
TD 128
SFO1 400.1524 MHz
FIDRES 81.211021 Hz
SW 12.989 ppm
FnMODE QF

F2 - Processing parameters
SI 1024
SF 400.1500000 MHz
WDW SINE
SSB 0
LB 0 Hz
GB 0
PC 1.40

F1 - Processing parameters
SI 1024
MC2 QF
SF 400.1500000 MHz
WDW SINE
SSB 0
LB 0 Hz
GB 0

110c

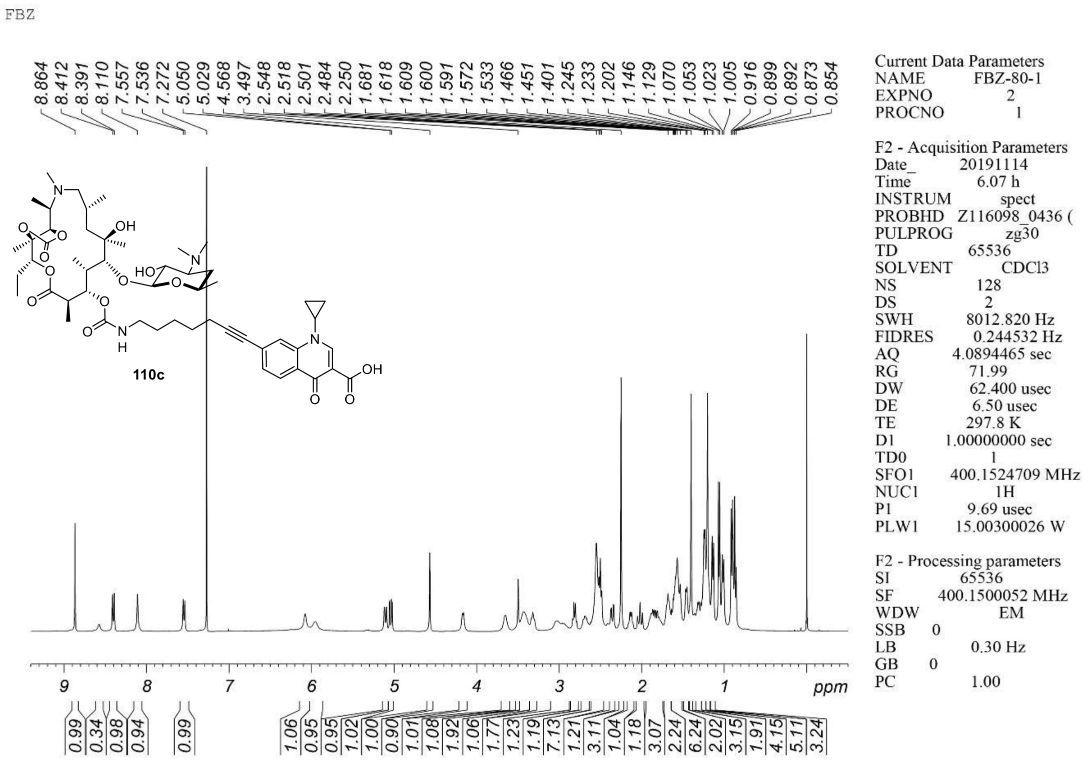
FBZ
8.864 8.412 8.391 8.110 7.557 7.536 7.272 5.050 5.029 4.568 3.497 2.548 2.518 2.501 2.484 2.250 1.681 1.618 1.609 1.600 1.591 1.572 1.533 1.466 1.451 1.401 1.245 1.233 1.202 1.146 1.129 1.070 1.053 1.023 1.005 0.916 0.899 0.892 0.873 0.854
110c
Current Data Parameters
NAME FBZ-80-1
EXPNO 2
PROCNO 1
F2 - Acquisition Parameters
Date_ 20191114
Time 6.07 h
INSTRUM spect
PROBHD Z116098_0436 (
PULPROG zg30
TD 65536
SOLVENT CDCl3
NS 128
DS 2
SWH 8012.820 Hz
FIDRES 0.244532 Hz
AQ 4.0894465 sec
RG 71.99
DW 62.400 usec
DE 6.50 usec
TE 297.8 K
D1 1.00000000 sec
TD0 1
SFO1 400.1524709 MHz
NUC1 1H
P1 9.69 usec
PLW1 15.00300026 W
F2 - Processing parameters
SI 65536
SF 400.1500052 MHz
WDW EM
SSB 0
LB 0.30 Hz
GB 0
PC 1.00
9 8 7 6 5 4 3 2 1 ppm
0.99 0.34 0.98 0.94 0.99 1.06 0.95 0.95 1.02 1.00 0.90 1.01 1.08 1.92 1.06 1.77 1.23 1.19 7.13 1.21 3.11 1.04 1.18 3.07 2.24 6.24 2.02 3.15 1.91 4.15 5.11 3.24

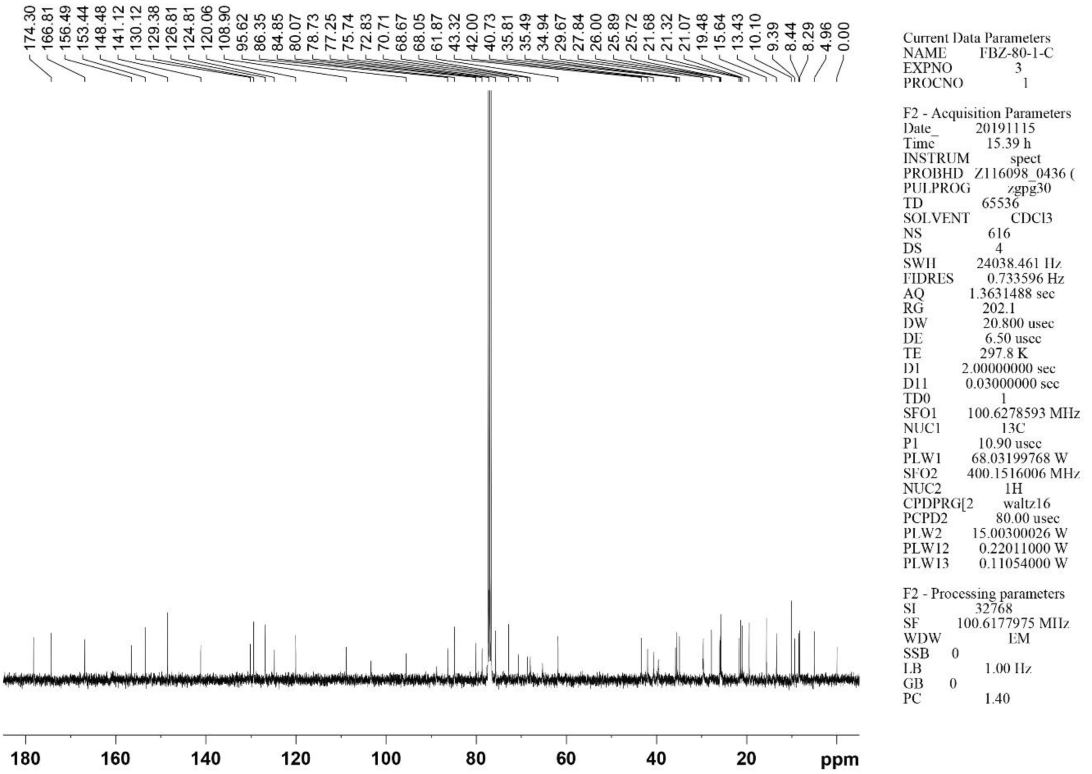
174.30 166.81 156.49 153.44 148.48 141.12 130.12 129.38 126.81 124.81 120.06 108.90 95.62 86.35 84.85 80.07 78.73 77.25 75.74 72.83 70.71 68.67 68.05 61.87 43.32 42.00 40.73 35.81 35.49 34.94 29.67 27.84 26.00 25.89 25.72 21.68 21.32 21.07 19.48 15.64 13.43 10.10 9.39 8.44 8.29 4.96 0.00
Current Data Parameters
NAME FBZ-80-1-C
EXPNO 3
PROCNO 1
F2 - Acquisition Parameters
Date_ 20191115
Time 15.39 h
INSTRUM spect
PROBHD Z116098_0436 (
PULPROG zgpg30
TD 65536
SOLVENT CDCl3
NS 616
DS 4
SWH 24038.461 Hz
FIDRES 0.733596 Hz
AQ 1.3631488 sec
RG 202.1
DW 20.800 usec
DE 6.50 usec
TE 297.8 K
D1 2.00000000 sec
D11 0.03000000 sec
TD0 1
SFO1 100.6278593 MHz
NUC1 13C
P1 10.90 usec
PLW1 68.03199768 W
SFO2 400.1516006 MHz
NUC2 1H
CPDPRG[2 waltz16
PCPD2 80.00 usec
PLW2 15.00300026 W
PLW12 0.22011000 W
PLW13 0.11054000 W
F2 - Processing parameters
SI 32768
SF 100.6177975 MHz
WDW EM
SSB 0
LB 1.00 Hz
GB 0
PC 1.40
180 160 140 120 100 80 60 40 20 ppm

111a

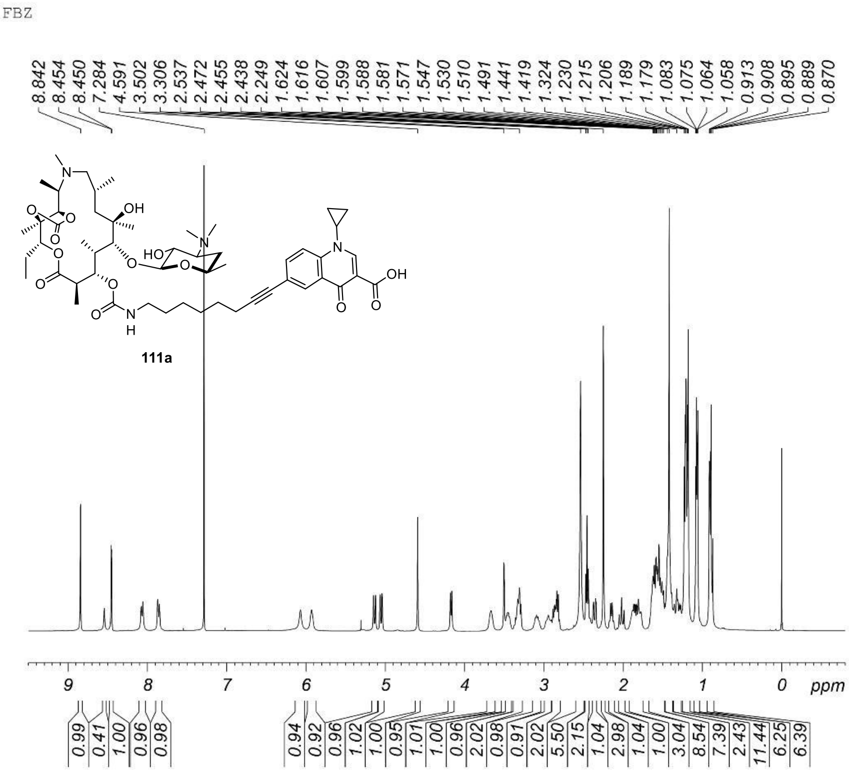

Current Data Parameters
NAME FBZ-83-2
EXPNO 1
PROCNO 1

F2 - Acquisition Parameters
Date_ 20191211
Time 23.18 h
INSTRUM spect
PROBHD Z116098_0436 (
PULPROG zg30
TD 65536
SOLVENT CDCl3
NS 128
DS 2
SWH 8012.820 Hz
FIDRES 0.244532 Hz
AQ 4.0894465 sec
RG 30.51
DW 62.400 usec
DE 6.50 usec
TE 297.8 K
D1 1.00000000 sec
TD0 1
SFO1 400.1524709 MHz
NUC1 1H
P1 9.69 usec
PLW1 15.00300026 W

F2 - Processing parameters
SI 65536
SF 400.1500009 MHz
WDW EM
SSB 0
LB 0.30 Hz
GB 0
PC 1.00

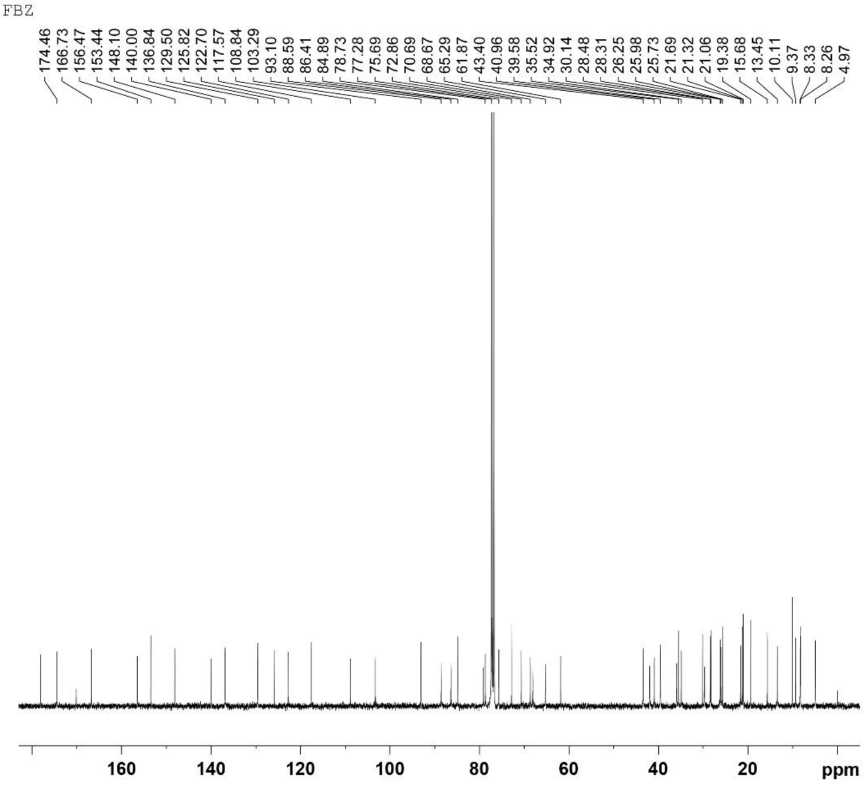

Current Data Parameters
NAME FBZ-83-2-C
EXPNO 2
PROCNO 1

F2 - Acquisition Parameters
Date_ 20191213
Time 22.33 h
INSTRUM spect
PROBHD Z116098_0436 (
PULPROG zgpg30
TD 65536
SOLVENT CDCl3
NS 1024
DS 4
SWH 24038.461 Hz
FIDRES 0.733596 Hz
AQ 1.3631488 sec
RG 202.1
DW 20.800 usec
DE 6.50 usec
TE 297.8 K
D1 2.00000000 sec
D11 0.03000000 sec
TD0 1
SFO1 100.6278593 MHz
NUC1 13C
P1 10.90 usec
PLW1 68.03199768 W
SFO2 400.1516006 MHz
NUC2 1H
CPDPRG[2 waltz16
PCPD2 80.00 usec
PLW2 15.00300026 W
PLW12 0.22011000 W
PLW13 0.11054000 W

F2 - Processing parameters
SI 32768
SF 100.6177975 MHz
WDW EM
SSB 0
LB 1.00 Hz
GB 0
PC 1.40

111c

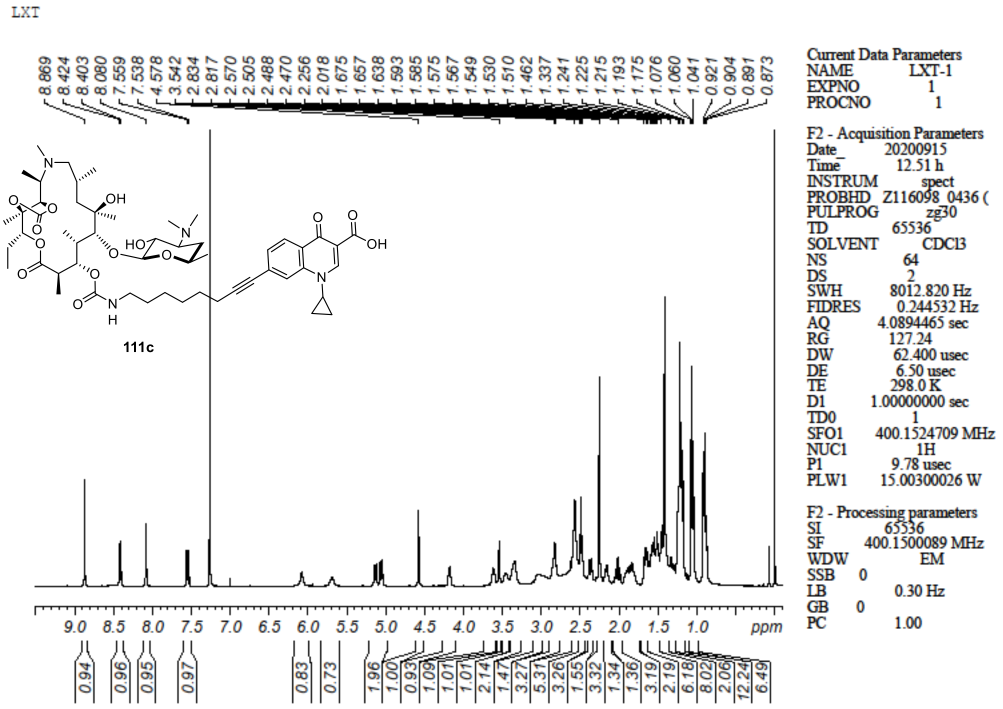
LXT
8.869 8.424 8.403 8.080 7.559 7.538 4.578 3.542 2.834 2.817 2.570 2.505 2.488 2.470 2.256 2.018 1.675 1.657 1.638 1.593 1.585 1.575 1.567 1.549 1.530 1.510 1.462 1.337 1.241 1.225 1.215 1.193 1.175 1.076 1.060 1.041 0.921 0.904 0.891 0.873
111c
9.0 8.5 8.0 7.5 7.0 6.5 6.0 5.5 5.0 4.5 4.0 3.5 3.0 2.5 2.0 1.5 1.0 ppm
0.94 0.96 0.95 0.97 0.83 0.73 1.96 1.00 0.93 1.09 1.01 1.01 2.14 1.47 3.27 5.31 3.26 1.55 3.32 1.34 1.36 3.19 2.19 6.18 8.02 2.06 12.24 6.49
Current Data Parameters
NAME LXT-1
EXPNO 1
PROCNO 1
F2 - Acquisition Parameters
Date_ 20200915
Time 12.51 h
INSTRUM spect
PROBHD Z116098_0436 (
PULPROG zg30
TD 65536
SOLVENT CDCl3
NS 64
DS 2
SWH 8012.820 Hz
FIDRES 0.244532 Hz
AQ 4.0894465 sec
RG 127.24
DW 62.400 usec
DE 6.50 usec
TE 298.0 K
D1 1.00000000 sec
TD0 1
SFO1 400.1524709 MHz
NUC1 1H
P1 9.78 usec
PLW1 15.00300026 W
F2 - Processing parameters
SI 65536
SF 400.1500089 MHz
WDW EM
SSB 0
LB 0.30 Hz
GB 0
PC 1.00

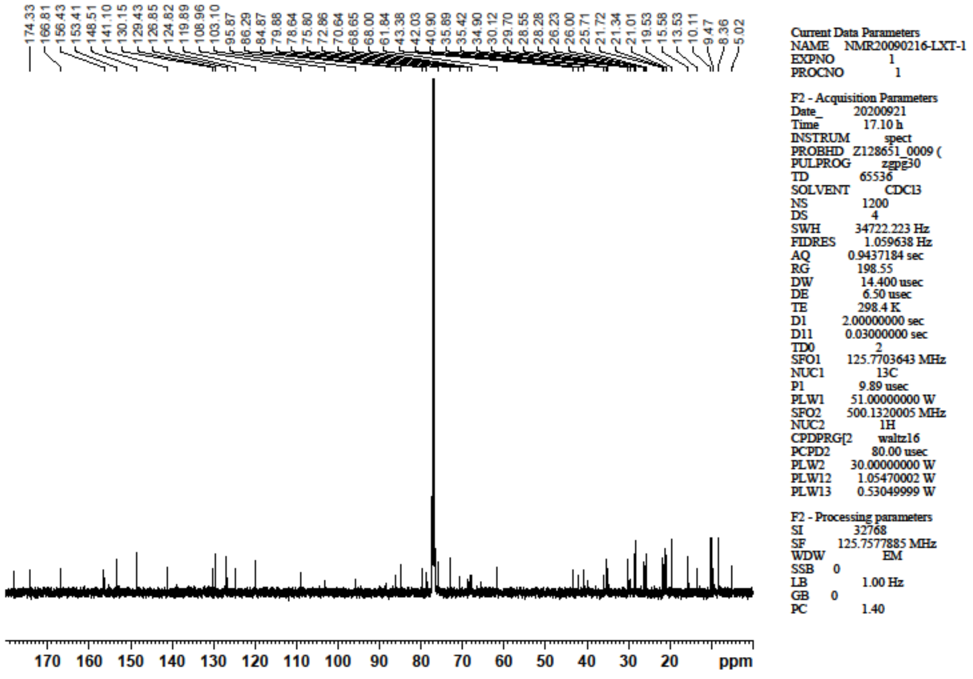
174.33 166.81 156.43 153.41 148.51 141.10 130.15 129.43 126.85 124.82 119.89 108.96 103.10 95.87 86.29 84.87 79.88 78.64 75.80 72.86 70.64 68.65 68.00 61.84 43.38 42.03 40.90 35.89 35.42 34.90 30.12 29.70 28.55 28.28 26.23 26.00 25.71 21.72 21.34 21.01 19.53 15.58 13.53 10.11 9.47 8.36 5.02
170 160 150 140 130 120 110 100 90 80 70 60 50 40 30 20 ppm
Current Data Parameters
NAME NMR20090216-LXT-1
EXPNO 1
PROCNO 1
F2 - Acquisition Parameters
Date_ 20200921
Time 17.10 h
INSTRUM spect
PROBHD Z128651_0009 (
PULPROG zgpg30
TD 65536
SOLVENT CDCl3
NS 1200
DS 4
SWH 34722.223 Hz
FIDRES 1.059638 Hz
AQ 0.9437184 sec
RG 198.55
DW 14.400 usec
DE 6.50 usec
TE 298.4 K
D1 2.00000000 sec
D11 0.03000000 sec
TD0 2
SFO1 125.7703643 MHz
NUC1 13C
P1 9.89 usec
PLW1 51.00000000 W
SFO2 500.1320005 MHz
NUC2 1H
CPDPRG[2 waltz16
PCPD2 80.00 usec
PLW2 30.00000000 W
PLW12 1.05470002 W
PLW13 0.53049999 W
F2 - Processing parameters
SI 32768
SF 125.7577885 MHz
WDW EM
SSB 0
LB 1.00 Hz
GB 0
PC 1.40

116a

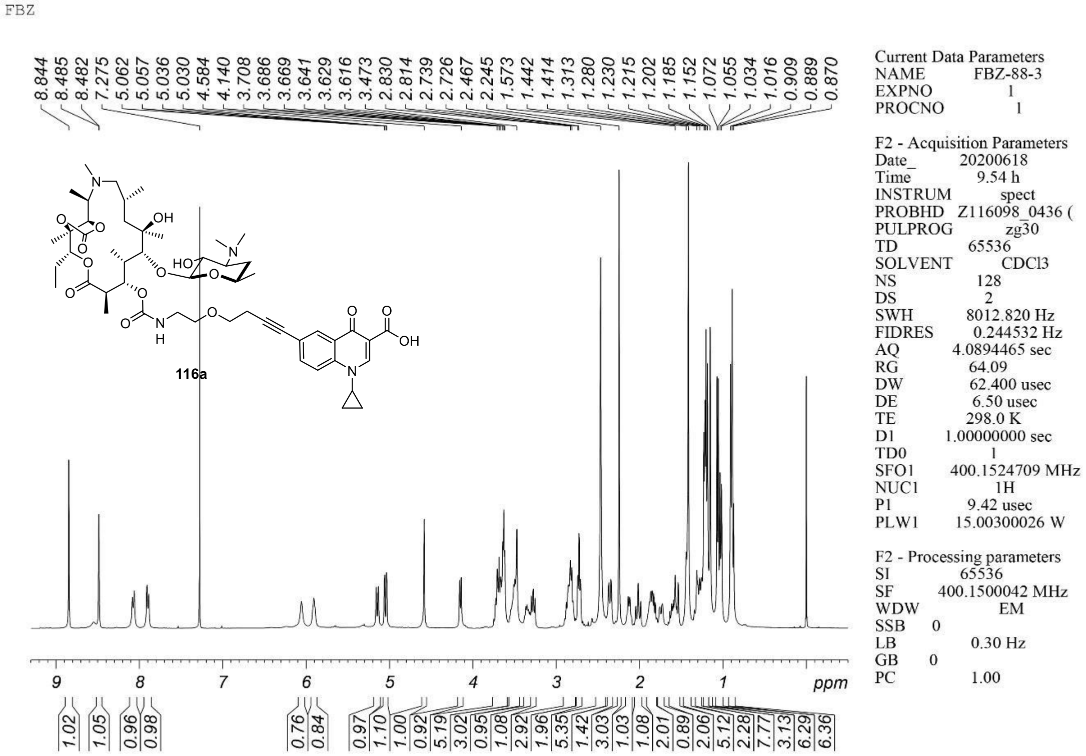
FBZ
8.844
8.485
8.482
7.275
5.062
5.057
5.036
5.030
4.584
4.140
3.708
3.686
3.669
3.641
3.629
3.616
3.473
2.830
2.814
2.739
2.726
2.467
2.245
1.573
1.442
1.414
1.313
1.280
1.230
1.215
1.202
1.185
1.152
1.072
1.055
1.034
1.016
0.909
0.889
0.870
116a
OH
HO
O
N
H
OH
9
8
7
6
5
4
3
2
1
ppm
1.02
1.05
0.96
0.98
0.76
0.84
0.97
1.10
1.00
0.92
5.19
3.02
0.95
1.08
2.92
1.96
5.35
1.42
3.03
1.03
1.08
2.01
0.89
2.06
5.12
2.28
7.77
3.13
6.29
6.36
Current Data Parameters
NAME FBZ-88-3
EXPNO 1
PROCNO 1
F2 - Acquisition Parameters
Date_ 20200618
Time 9.54 h
INSTRUM spect
PROBHD Z116098_0436 (
PULPROG zg30
TD 65536
SOLVENT CDCl3
NS 128
DS 2
SWH 8012.820 Hz
FIDRES 0.244532 Hz
AQ 4.0894465 sec
RG 64.09
DW 62.400 usec
DE 6.50 usec
TE 298.0 K
D1 1.00000000 sec
TD0 1
SFO1 400.1524709 MHz
NUC1 1H
P1 9.42 usec
PLW1 15.00300026 W
F2 - Processing parameters
SI 65536
SF 400.1500042 MHz
WDW EM
SSB 0
LB 0.30 Hz
GB 0
PC 1.00

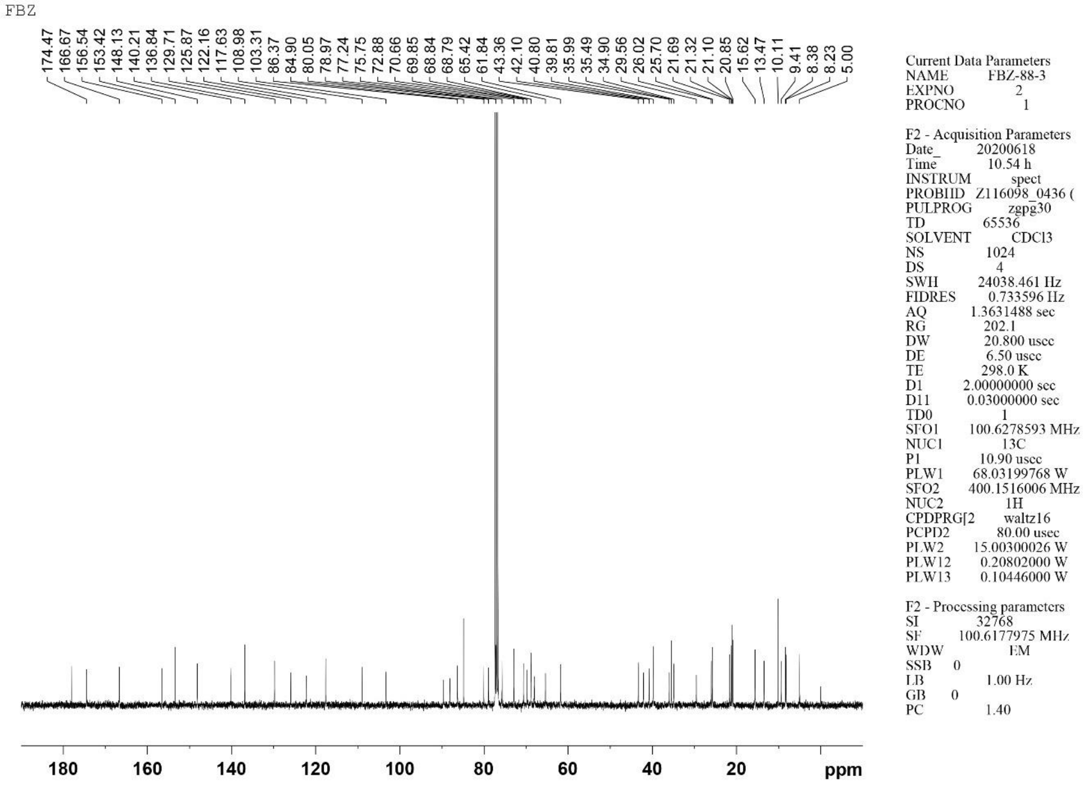
FBZ
174.47
166.67
156.54
153.42
148.13
140.21
136.84
129.71
125.87
122.16
117.63
108.98
103.31
86.37
84.90
80.05
78.97
77.24
75.75
72.88
70.66
69.85
68.84
68.79
65.42
61.84
43.36
42.10
40.80
39.81
35.99
35.49
34.90
29.56
26.02
25.70
21.69
21.32
21.10
20.85
15.62
13.47
10.11
9.41
8.38
8.23
5.00
180
160
140
120
100
80
60
40
20
ppm
Current Data Parameters
NAME FBZ-88-3
EXPNO 2
PROCNO 1
F2 - Acquisition Parameters
Date_ 20200618
Time 10.54 h
INSTRUM spect
PROBHD Z116098_0436 (
PULPROG zgpg30
TD 65536
SOLVENT CDCl3
NS 1024
DS 4
SWH 24038.461 Hz
FIDRES 0.733596 Hz
AQ 1.3631488 sec
RG 202.1
DW 20.800 usec
DE 6.50 usec
TE 298.0 K
D1 2.00000000 sec
D11 0.03000000 sec
TD0 1
SFO1 100.6278593 MHz
NUC1 13C
P1 10.90 usec
PLW1 68.03199768 W
SFO2 400.1516006 MHz
NUC2 1H
CPDPRG[2 waltz16
PCPD2 80.00 usec
PLW2 15.00300026 W
PLW12 0.20802000 W
PLW13 0.10446000 W
F2 - Processing parameters
SI 32768
SF 100.6177975 MHz
WDW EM
SSB 0
LB 1.00 Hz
GB 0
PC 1.40

116c

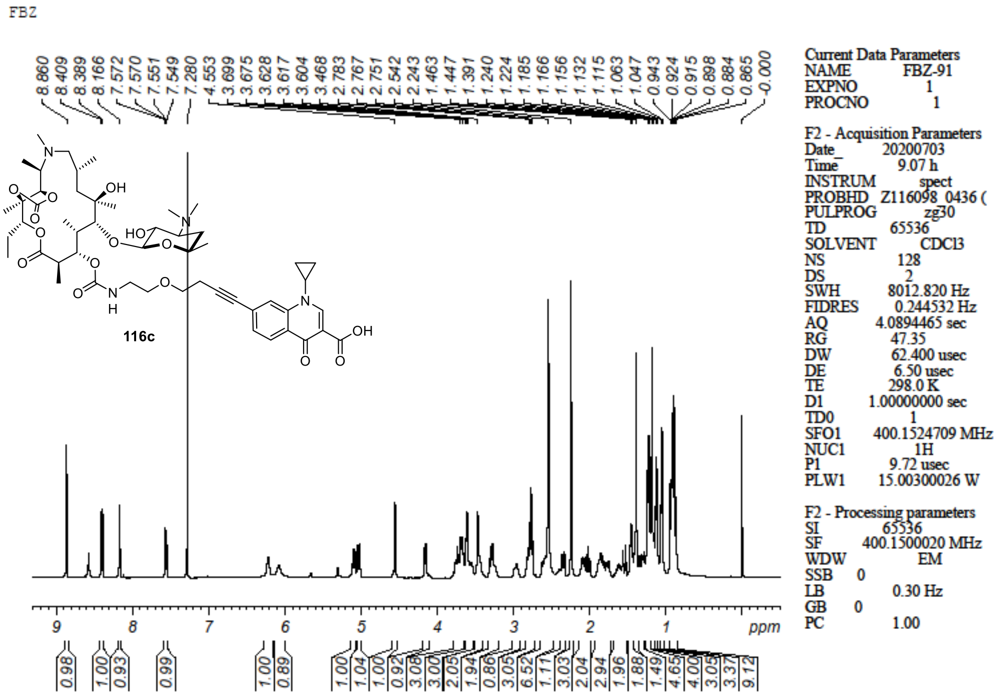
FBZ
116c
Current Data Parameters
NAME FBZ-91
EXPNO 1
PROCNO 1
F2 - Acquisition Parameters
Date_ 20200703
Time 9.07 h
INSTRUM spect
PROBHD Z116098_0436 (
PULPROG zg30
TD 65536
SOLVENT CDCl3
NS 128
DS 2
SWH 8012.820 Hz
FIDRES 0.244532 Hz
AQ 4.0894465 sec
RG 47.35
DW 62.400 usec
DE 6.50 usec
TE 298.0 K
D1 1.00000000 sec
TD0 1
SFO1 400.1524709 MHz
NUC1 1H
P1 9.72 usec
PLW1 15.00300026 W
F2 - Processing parameters
SI 65536
SF 400.1500020 MHz
WDW EM
SSB 0
LB 0.30 Hz
GB 0
PC 1.00
ppm

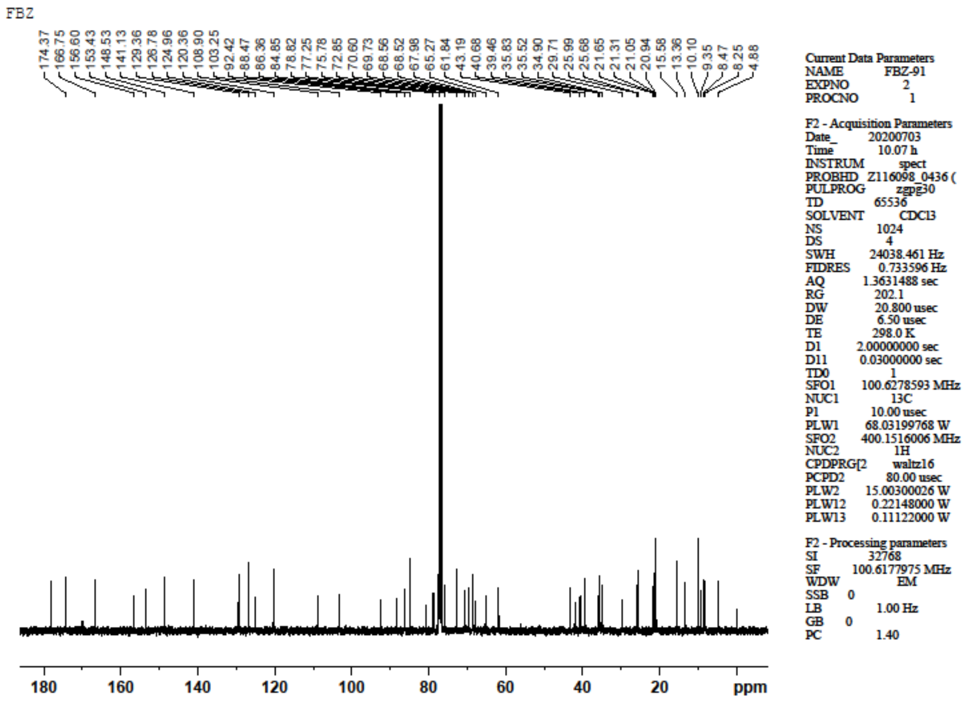
FBZ
Current Data Parameters
NAME FBZ-91
EXPNO 2
PROCNO 1
F2 - Acquisition Parameters
Date_ 20200703
Time 10.07 h
INSTRUM spect
PROBHD Z116098_0436 (
PULPROG zgpg30
TD 65536
SOLVENT CDCl3
NS 1024
DS 4
SWH 24038.461 Hz
FIDRES 0.733596 Hz
AQ 1.3631488 sec
RG 202.1
DW 20.800 usec
DE 6.50 usec
TE 298.0 K
D1 2.00000000 sec
D11 0.03000000 sec
TD0 1
SFO1 100.6278593 MHz
NUC1 13C
P1 10.00 usec
PLW1 68.03199768 W
SFO2 400.1516006 MHz
NUC2 1H
CPDPRG[2 waltz16
PCPD2 80.00 usec
PLW2 15.00300026 W
PLW12 0.22148000 W
PLW13 0.11122000 W
F2 - Processing parameters
SI 32768
SF 100.6177975 MHz
WDW EM
SSB 0
LB 1.00 Hz
GB 0
PC 1.40
ppm

117a

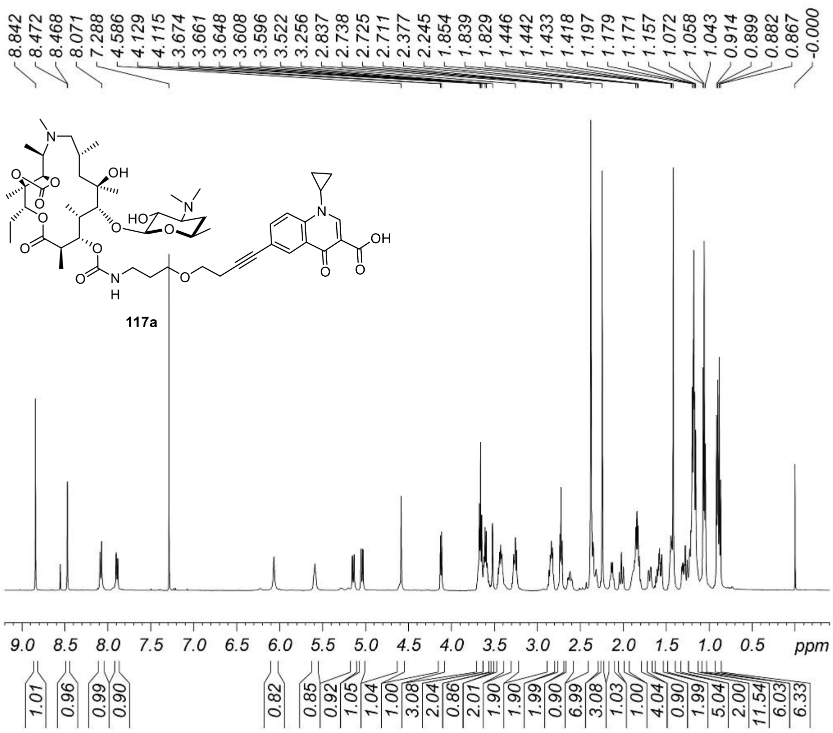

Current Data Parameters
NAME NMR20010045-FBZ-90
EXPNO 1
PROCNO 1

F2 - Acquisition Parameters
Date_ 20200106
Time 18.20 h
INSTRUM spect
PROBHD Z128651_0009 (
PULPROG zg30
TD 65536
SOLVENT CDCl3
NS 128
DS 2
SWH 10000.000 Hz
FIDRES 0.305176 Hz
AQ 3.2767999 sec
RG 100.47
DW 50.000 usec
DE 6.50 usec
TE 298.1 K
D1 1.00000000 sec
TD0 1
SFO1 500.1330883 MHz
NUC1 1H
P1 15.00 usec
PLW1 30.00000000 W

F2 - Processing parameters
SI 65536
SF 500.1299980 MHz
WDW EM
SSB 0
LB 0.30 Hz
GB 0
PC 1.00

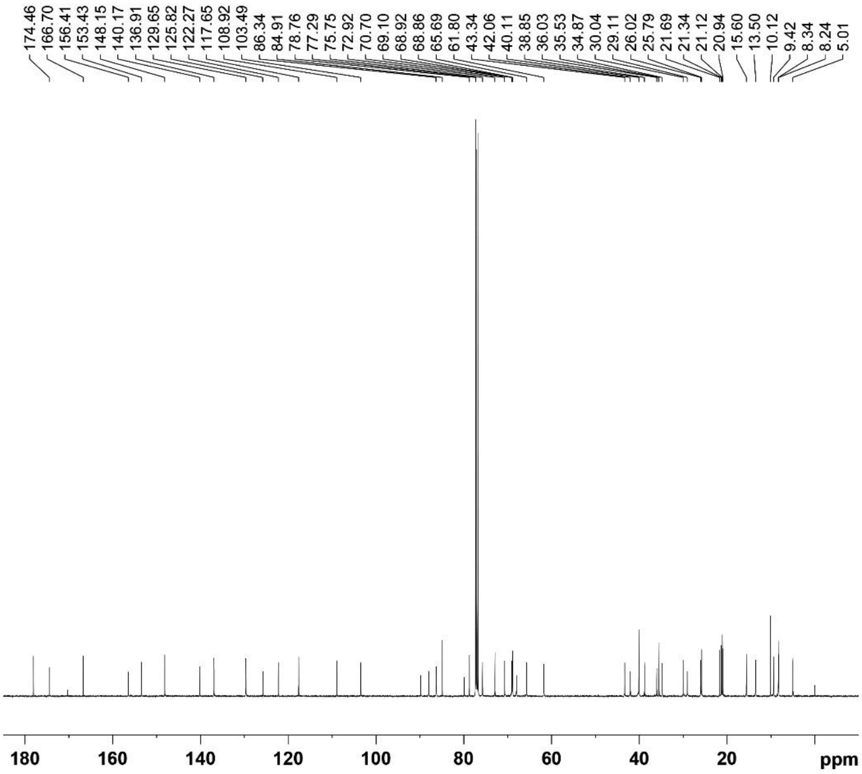

Current Data Parameters
NAME NMR20010045-FBZ-90
EXPNO 2
PROCNO 1

F2 - Acquisition Parameters
Date_ 20200107
Time 21.59 h
INSTRUM spect
PROBHD Z128651_0009 (
PULPROG zgpg30
TD 65536
SOLVENT CDCl3
NS 3072
DS 4
SWH 34722.223 Hz
FIDRES 1.059638 Hz
AQ 0.9437184 sec
RG 198.55
DW 14.400 usec
DE 6.50 usec
TE 298.1 K
D1 2.00000000 sec
D11 0.03000000 sec
TD0 1
SFO1 125.7703643 MHz
NUC1 13C
P1 9.89 usec
PLW1 51.00000000 W
SFO2 500.1320005 MHz
NUC2 1H
CPDPRG[2 waltz16
PCPD2 80.00 usec
PLW2 30.00000000 W
PLW12 1.05470002 W
PLW13 0.53049999 W

F2 - Processing parameters
SI 32768
SF 125.7577875 MHz
WDW EM
SSB 0
LB 1.00 Hz
GB 0
PC 1.40

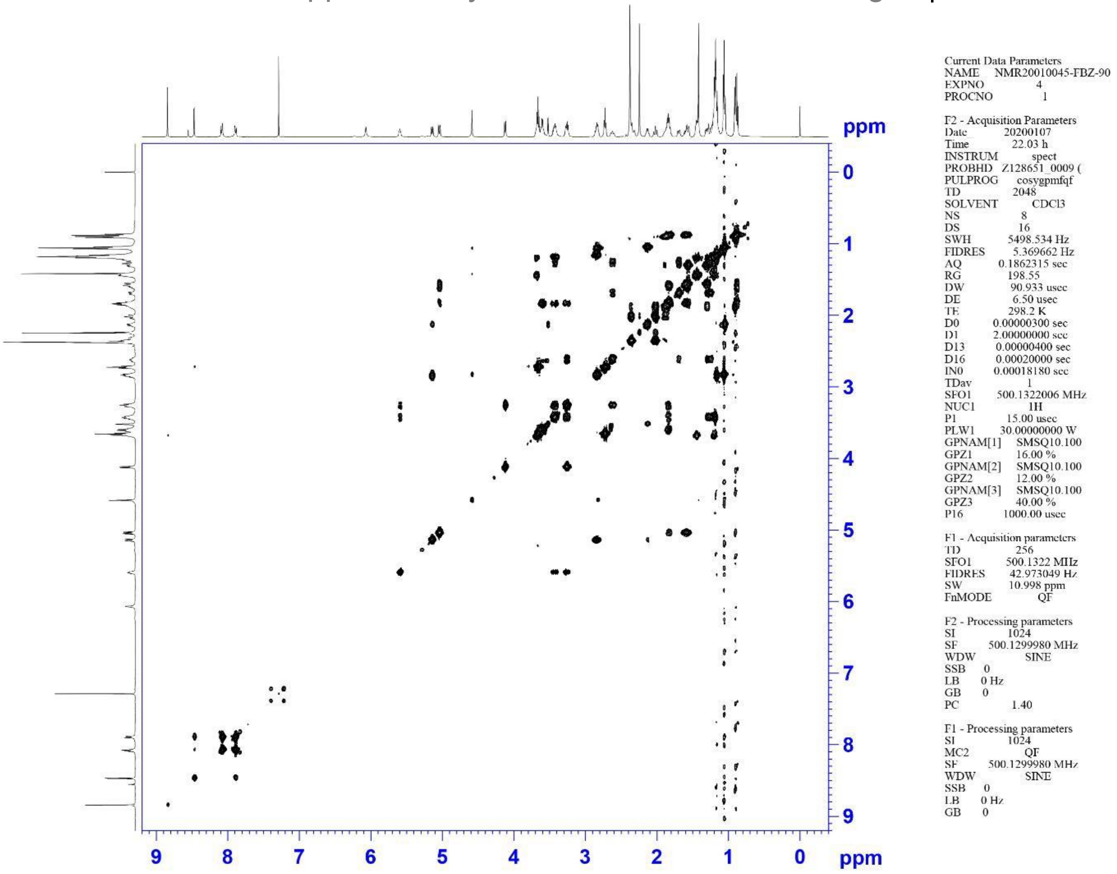
ppm
9 8 7 6 5 4 3 2 1 0
Current Data Parameters
NAME NMR20010045-FBZ-90
EXPNO 4
PROCNO 1
F2 - Acquisition Parameters
Date 20200107
Time 22.03 h
INSTRUM spect
PROBHD Z128651_0009 (
PULPROG cosygpmfqf
TD 2048
SOLVENT CDCl3
NS 8
DS 16
SWH 5498.534 Hz
FIDRES 5.369662 Hz
AQ 0.1862315 sec
RG 198.55
DW 90.933 usec
DE 6.50 usec
TE 298.2 K
D0 0.00000300 sec
D1 2.00000000 sec
D13 0.00000400 sec
D16 0.00020000 sec
IN0 0.00018180 sec
TDav 1
SFO1 500.1322006 MHz
NUC1 1H
P1 15.00 usec
PLW1 30.00000000 W
GPNAM[1] SMSQ10.100
GPZ1 16.00 %
GPNAM[2] SMSQ10.100
GPZ2 12.00 %
GPNAM[3] SMSQ10.100
GPZ3 40.00 %
P16 1000.00 usec
F1 - Acquisition parameters
TD 256
SFO1 500.1322 MHz
FIDRES 42.973049 Hz
SW 10.998 ppm
FnMODE QF
F2 - Processing parameters
SI 1024
SF 500.1299980 MHz
WDW SINE
SSB 0
LB 0 Hz
GB 0
PC 1.40
F1 - Processing parameters
SI 1024
MC2 QF
SF 500.1299980 MHz
WDW SINE
SSB 0
LB 0 Hz
GB 0

126c

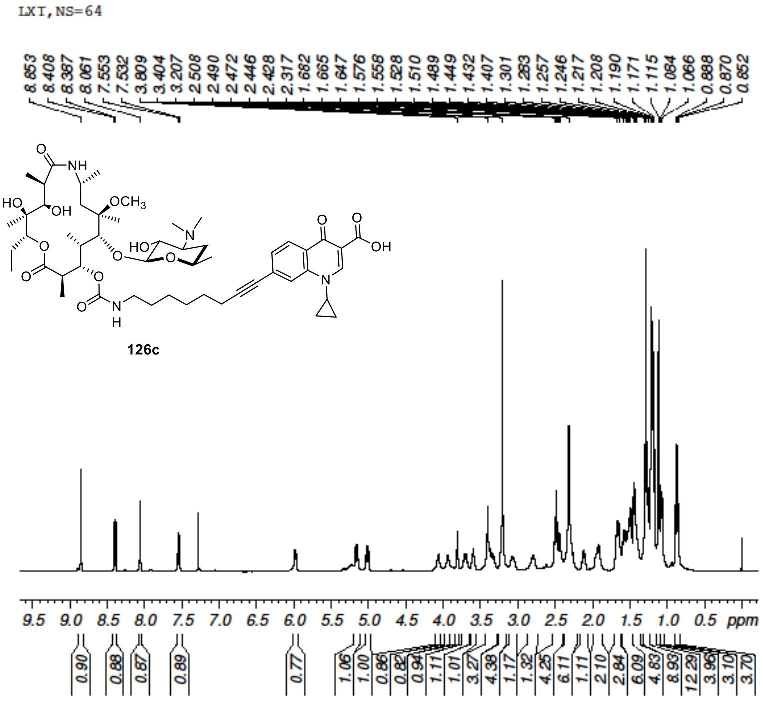

Current Data Parameters
NAME LXT-43-1
EXPNO 1
PROCNO 1

F2 - Acquisition Parameters
Date_ 20210802
Time 21.25 h
INSTRUM spect
PROBHD Z116098_0436 (
PULPROG zg30
TD 65536
SOLVENT CDCl3
NS 64
DS 2
SWH 8012.820 Hz
FIDRES 0.244532 Hz
AQ 4.0894465 sec
RG 30.51
DW 62.400 usec
DE 6.50 usec
TE 301.1 K
D1 1.00000000 sec
TD0 1
SFO1 400.1524709 MHz
NUC1 1H
P1 9.78 usec
PLW1 15.00300026 W

F2 - Processing parameters
SI 65536
SF 400.1500008 MHz
WDW EM
SSB 0
LB 0.30 Hz
GB 0
PC 1.00

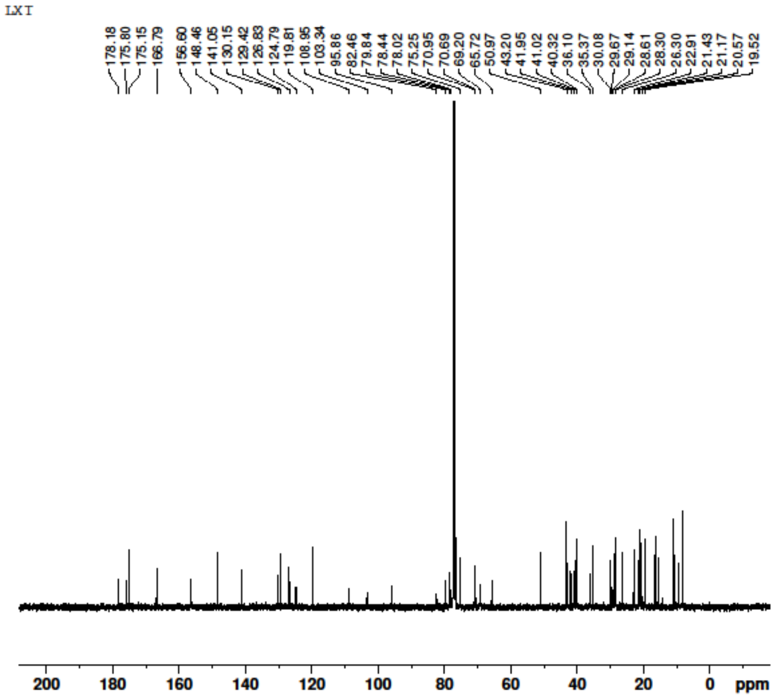

Current Data Parameters
NAME LXT-43
EXPNO 2
PROCNO 1

F2 - Acquisition Parameters
Date_ 20210807
Time 19.19 h
INSTRUM spect
PROBHD Z116098_0436 (
PULPROG zgpg30
TD 65536
SOLVENT CDCl3
NS 1024
DS 4
SWH 24038.461 Hz
FIDRES 0.733596 Hz
AQ 1.3631488 sec
RG 202.1
DW 20.800 usec
DE 6.50 usec
TE 302.7 K
D1 2.00000000 sec
D11 0.03000000 sec
TD0 1
SFO1 100.6278593 MHz
NUC1 13C
P1 10.58 usec
PLW1 68.07700348 W
SFO2 400.1516006 MHz
NUC2 1H
CPDPRG[2 waltz16
PCPD2 80.00 usec
PLW2 15.00300026 W
PLW12 0.22421999 W
PLW13 0.11260000 W

F2 - Processing parameters
SI 32768
SF 100.6177975 MHz
WDW EM
SSB 0
LB 1.00 Hz
GB 0
PC 1.40

126g

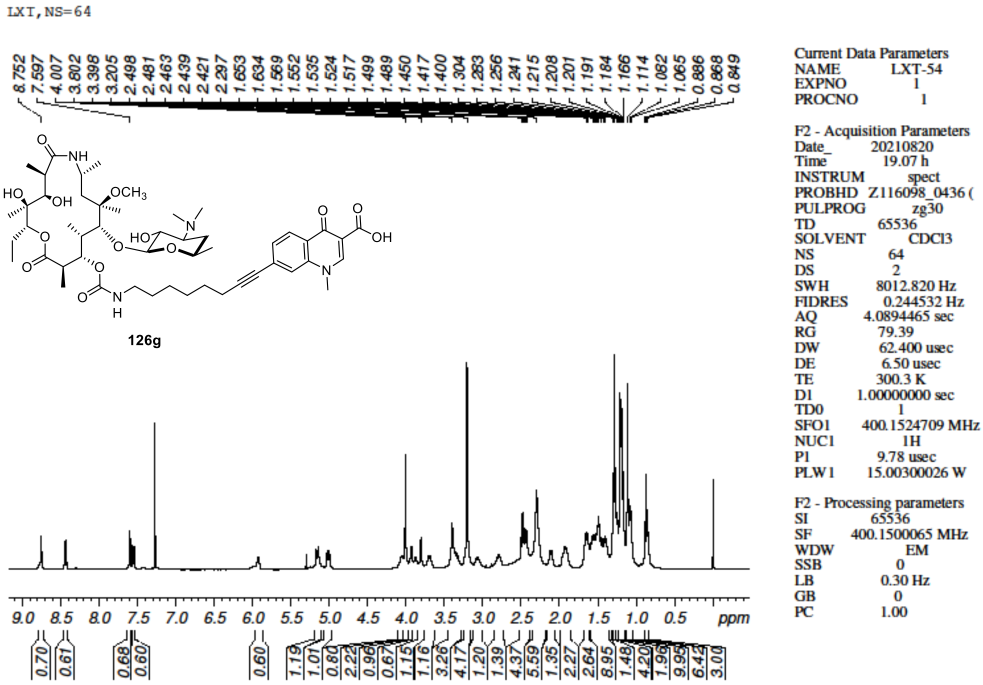
LXT,NS=64
8.752
7.597
4.007
3.802
3.398
3.205
2.498
2.481
2.463
2.439
2.421
2.297
1.653
1.634
1.569
1.552
1.535
1.524
1.517
1.499
1.489
1.450
1.417
1.400
1.304
1.283
1.256
1.241
1.215
1.208
1.201
1.191
1.184
1.166
1.114
1.082
1.065
0.886
0.868
0.849
126g
9.0 8.5 8.0 7.5 7.0 6.5 6.0 5.5 5.0 4.5 4.0 3.5 3.0 2.5 2.0 1.5 1.0 0.5 ppm
0.70
0.61
0.68
0.60
0.60
1.19
1.01
0.80
2.22
0.96
0.67
1.15
1.16
3.26
4.17
1.20
1.39
4.37
5.59
1.35
2.27
2.64
8.95
1.48
4.20
1.96
9.95
6.42
3.00
Current Data Parameters
NAME LXT-54
EXPNO 1
PROCNO 1
F2 - Acquisition Parameters
Date_ 20210820
Time 19.07 h
INSTRUM spect
PROBHD Z116098_0436 (
PULPROG zg30
TD 65536
SOLVENT CDCl3
NS 64
DS 2
SWH 8012.820 Hz
FIDRES 0.244532 Hz
AQ 4.0894465 sec
RG 79.39
DW 62.400 usec
DE 6.50 usec
TE 300.3 K
D1 1.00000000 sec
TD0 1
SFO1 400.1524709 MHz
NUC1 1H
P1 9.78 usec
PLW1 15.00300026 W
F2 - Processing parameters
SI 65536
SF 400.1500065 MHz
WDW EM
SSB 0
LB 0.30 Hz
GB 0
PC 1.00

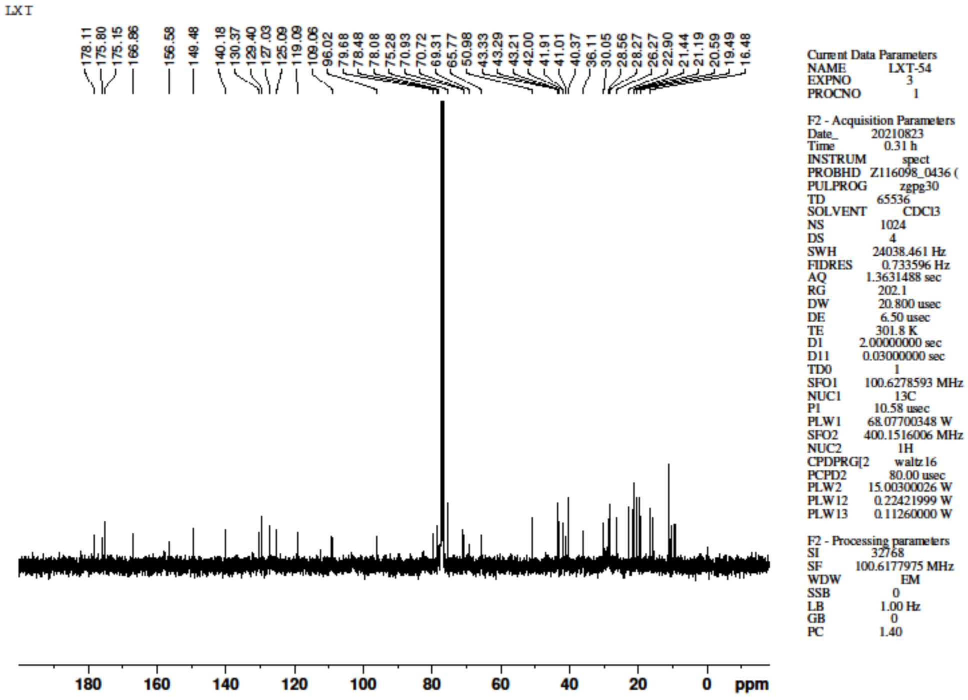
LXT
178.11
175.80
175.15
166.86
156.58
149.48
140.18
130.37
129.40
127.03
125.09
119.09
109.06
96.02
79.68
78.48
78.08
75.28
70.93
70.72
69.31
65.77
50.98
43.33
43.29
43.21
42.00
41.91
41.01
40.37
36.11
30.05
28.56
28.27
26.27
22.90
21.44
21.19
20.59
19.49
16.48
180 160 140 120 100 80 60 40 20 0 ppm
Current Data Parameters
NAME LXT-54
EXPNO 3
PROCNO 1
F2 - Acquisition Parameters
Date_ 20210823
Time 0.31 h
INSTRUM spect
PROBHD Z116098_0436 (
PULPROG zgpg30
TD 65536
SOLVENT CDCl3
NS 1024
DS 4
SWH 24038.461 Hz
FIDRES 0.733596 Hz
AQ 1.3631488 sec
RG 202.1
DW 20.800 usec
DE 6.50 usec
TE 301.8 K
D1 2.00000000 sec
D11 0.03000000 sec
TD0 1
SFO1 100.6278593 MHz
NUC1 13C
P1 10.58 usec
PLW1 68.07700348 W
SFO2 400.1516006 MHz
NUC2 1H
CPDPRG[2 waltz16
PCPD2 80.00 usec
PLW2 15.00300026 W
PLW12 0.22421999 W
PLW13 0.11260000 W
F2 - Processing parameters
SI 32768
SF 100.6177975 MHz
WDW EM
SSB 0
LB 1.00 Hz
GB 0
PC 1.40

126i

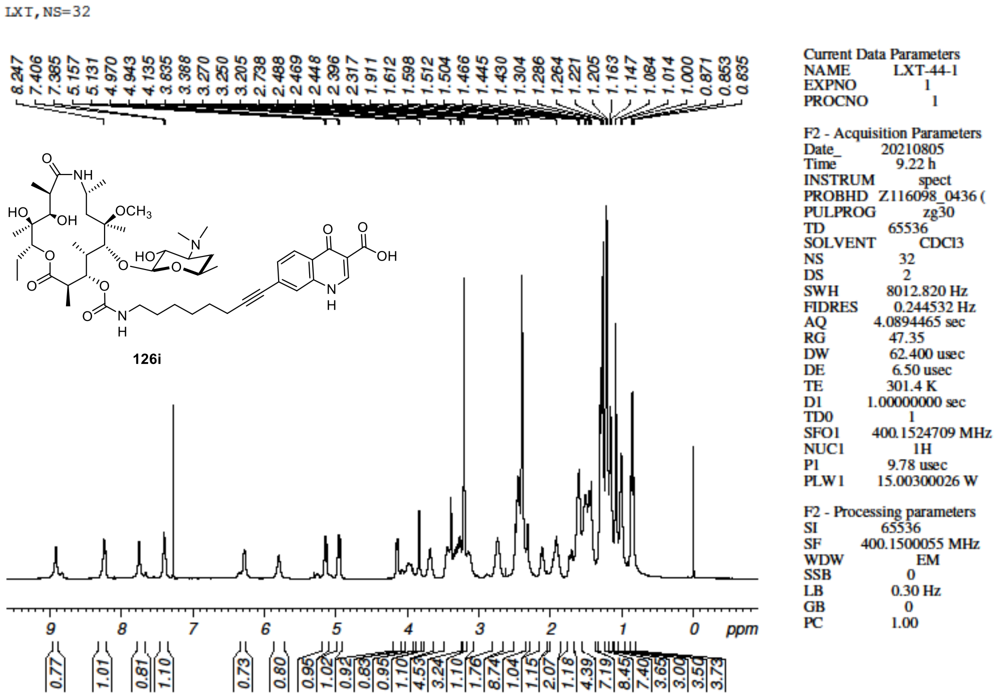

LXT,NS=32
8.247 7.406 7.385 5.157 5.131 4.970 4.943 4.135 3.835 3.388 3.270 3.250 3.205 2.738 2.488 2.469 2.448 2.396 2.317 1.911 1.612 1.598 1.512 1.504 1.466 1.445 1.430 1.304 1.286 1.264 1.221 1.205 1.163 1.147 1.084 1.014 1.000 0.871 0.853 0.835
126i
9 8 7 6 5 4 3 2 1 0 ppm
0.77 1.01 0.81 1.10 0.73 0.80 0.95 1.02 0.92 0.83 0.95 1.10 4.53 3.24 1.10 1.76 8.74 1.04 1.15 2.07 1.18 4.39 7.19 8.45 7.40 3.65 3.00 3.50 3.73
Current Data Parameters
NAME LXT-44-1
EXPNO 1
PROCNO 1
F2 - Acquisition Parameters
Date_ 20210805
Time 9.22 h
INSTRUM spect
PROBHD Z116098_0436 (
PULPROG zg30
TD 65536
SOLVENT CDCl3
NS 32
DS 2
SWH 8012.820 Hz
FIDRES 0.244532 Hz
AQ 4.0894465 sec
RG 47.35
DW 62.400 usec
DE 6.50 usec
TE 301.4 K
D1 1.00000000 sec
TD0 1
SFO1 400.1524709 MHz
NUC1 1H
P1 9.78 usec
PLW1 15.00300026 W
F2 - Processing parameters
SI 65536
SF 400.1500055 MHz
WDW EM
SSB 0
LB 0.30 Hz
GB 0
PC 1.00

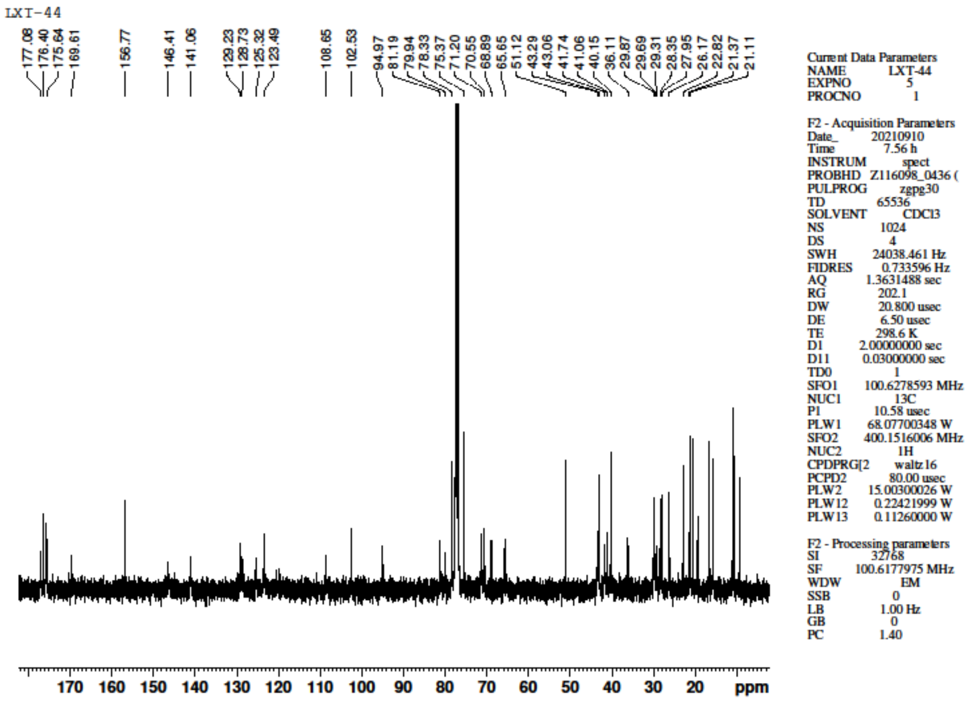

LXT-44
177.08 176.40 175.64 169.61 156.77 146.41 141.06 129.23 128.73 125.32 123.49 108.65 102.53 94.97 81.19 79.94 78.33 75.37 71.20 70.55 68.89 65.65 51.12 43.29 43.06 41.74 41.06 40.15 36.11 29.87 29.69 29.31 28.35 27.95 26.17 22.82 21.37 21.11
170 160 150 140 130 120 110 100 90 80 70 60 50 40 30 20 ppm
Current Data Parameters
NAME LXT-44
EXPNO 5
PROCNO 1
F2 - Acquisition Parameters
Date_ 20210910
Time 7.56 h
INSTRUM spect
PROBHD Z116098_0436 (
PULPROG zgpg30
TD 65536
SOLVENT CDCl3
NS 1024
DS 4
SWH 24038.461 Hz
FIDRES 0.733596 Hz
AQ 1.3631488 sec
RG 202.1
DW 20.800 usec
DE 6.50 usec
TE 298.6 K
D1 2.00000000 sec
D11 0.03000000 sec
TD0 1
SFO1 100.6278593 MHz
NUC1 13C
P1 10.58 usec
PLW1 68.07700348 W
SFO2 400.1516006 MHz
NUC2 1H
CPDPRG[2 waltz16
PCPD2 80.00 usec
PLW2 15.00300026 W
PLW12 0.22421999 W
PLW13 0.11260000 W
F2 - Processing parameters
SI 32768
SF 100.6177975 MHz
WDW EM
SSB 0
LB 1.00 Hz
GB 0
PC 1.40

126j

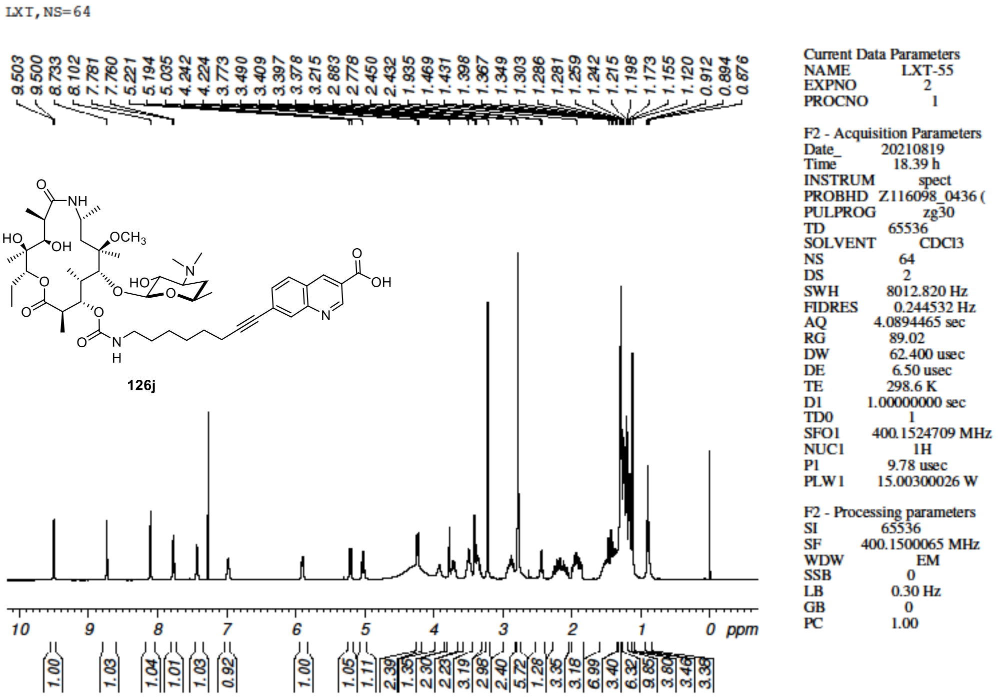
LXT, NS=64
9.503 9.500 8.733 8.102 7.781 7.760 5.221 5.194 5.035 4.242 4.224 3.773 3.490 3.409 3.397 3.378 3.215 2.883 2.778 2.450 2.432 1.935 1.469 1.431 1.398 1.367 1.349 1.303 1.286 1.281 1.259 1.242 1.215 1.198 1.173 1.155 1.120 0.912 0.894 0.876
126j
10 9 8 7 6 5 4 3 2 1 0 ppm
1.00 1.03 1.04 1.01 1.03 0.92 1.00 1.05 1.11 2.39 1.35 2.30 2.23 3.19 2.98 2.40 5.72 1.28 3.35 3.18 6.99 3.40 6.32 9.85 3.80 3.46 3.39
Current Data Parameters
NAME LXT-55
EXPNO 2
PROCNO 1
F2 - Acquisition Parameters
Date_ 20210819
Time 18.39 h
INSTRUM spect
PROBHD Z116098_0436 (
PULPROG zg30
TD 65536
SOLVENT CDCl3
NS 64
DS 2
SWH 8012.820 Hz
FIDRES 0.244532 Hz
AQ 4.0894465 sec
RG 89.02
DW 62.400 usec
DE 6.50 usec
TE 298.6 K
D1 1.00000000 sec
TD0 1
SFO1 400.1524709 MHz
NUC1 1H
P1 9.78 usec
PLW1 15.00300026 W
F2 - Processing parameters
SI 65536
SF 400.1500065 MHz
WDW EM
SSB 0
LB 0.30 Hz
GB 0
PC 1.00

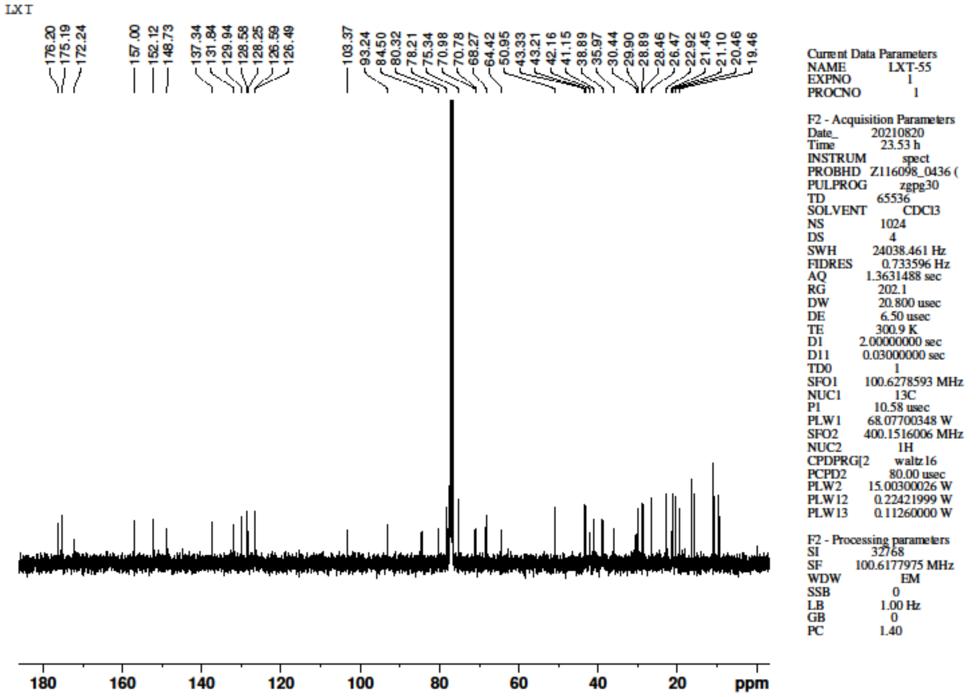
LXT
176.20 175.19 172.24 157.00 152.12 148.73 137.34 131.84 129.94 128.58 128.25 126.59 126.49 103.37 93.24 84.50 80.32 78.21 75.34 70.98 70.78 68.27 64.42 50.95 43.33 43.21 42.16 41.15 38.89 35.97 30.44 29.90 28.89 28.46 26.47 22.92 21.45 21.10 20.46 19.46
180 160 140 120 100 80 60 40 20 ppm
Current Data Parameters
NAME LXT-55
EXPNO 1
PROCNO 1
F2 - Acquisition Parameters
Date_ 20210820
Time 23.53 h
INSTRUM spect
PROBHD Z116098_0436 (
PULPROG zgpg30
TD 65536
SOLVENT CDCl3
NS 1024
DS 4
SWH 24038.461 Hz
FIDRES 0.733596 Hz
AQ 1.3631488 sec
RG 202.1
DW 20.800 usec
DE 6.50 usec
TE 300.9 K
D1 2.00000000 sec
D11 0.03000000 sec
TD0 1
SFO1 100.6278593 MHz
NUC1 13C
P1 10.58 usec
PLW1 68.07700348 W
SFO2 400.1516006 MHz
NUC2 1H
CPDPRG[2 waltz16
PCPD2 80.00 usec
PLW2 15.00300026 W
PLW12 0.22421999 W
PLW13 0.11260000 W
F2 - Processing parameters
SI 32768
SF 100.6177975 MHz
WDW EM
SSB 0
LB 1.00 Hz
GB 0
PC 1.40

127i

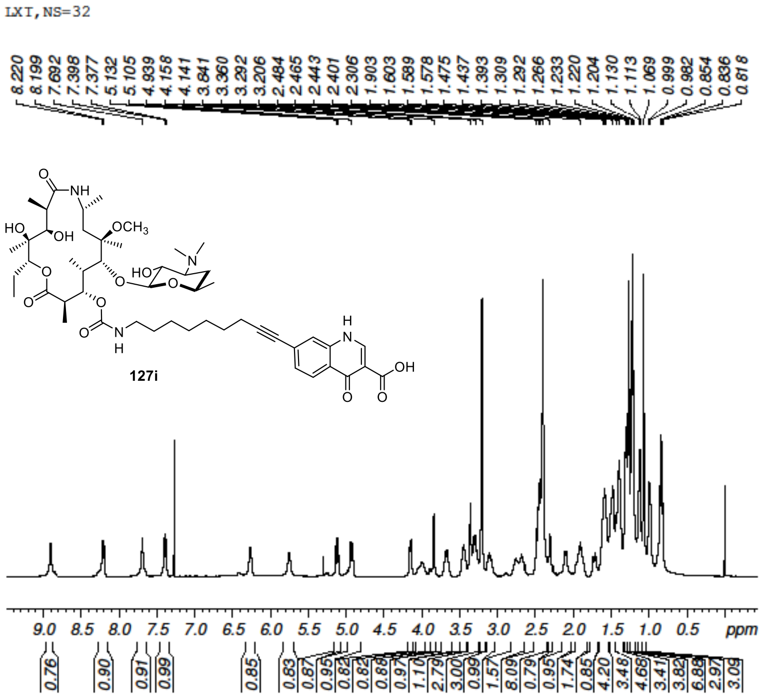

Current Data Parameters
NAME LXT-48-1
EXPNO 1
PROCNO 1

F2 - Acquisition Parameters
Date_ 20210816
Time 19.21 h
INSTRUM spect
PROBHD Z116098_0436 (
PULPROG zg30
TD 65536
SOLVENT CDCl3
NS 32
DS 2
SWH 8012.820 Hz
FIDRES 0.244532 Hz
AQ 4.0894465 sec
RG 53.41
DW 62.400 usec
DE 6.50 usec
TE 299.6 K
D1 1.00000000 sec
TD0 1
SFO1 400.1524709 MHz
NUC1 1H
P1 9.78 usec
PLW1 15.00300026 W

F2 - Processing parameters
SI 65536
SF 400.1500053 MHz
WDW EM
SSB 0
LB 0.30 Hz
GB 0
PC 1.00

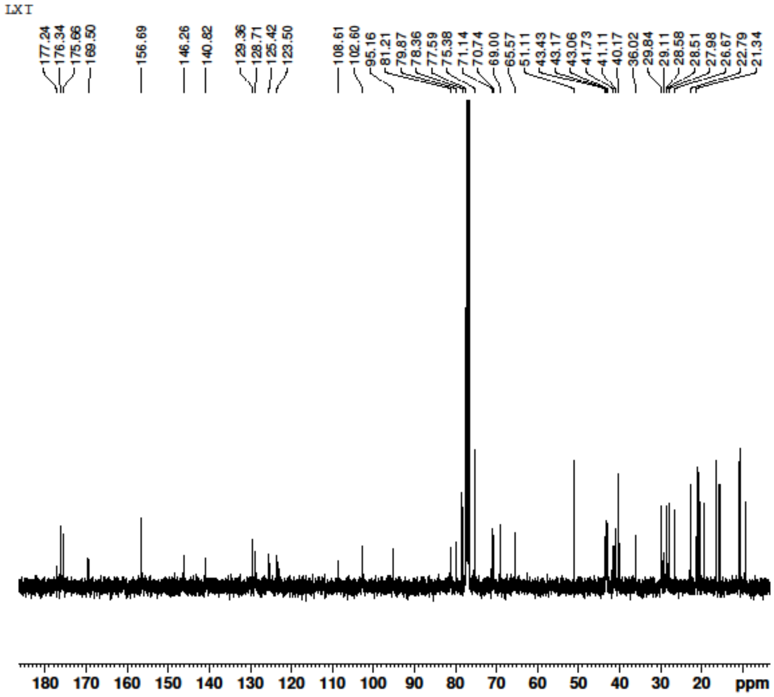

Current Data Parameters
NAME LXT-48
EXPNO 2
PROCNO 1

F2 - Acquisition Parameters
Date_ 20210817
Time 3.22 h
INSTRUM spect
PROBHD Z116098_0436 (
PULPROG zgpg30
TD 65536
SOLVENT CDCl3
NS 1024
DS 4
SWH 24038.461 Hz
FIDRES 0.733596 Hz
AQ 1.3631488 sec
RG 202.1
DW 20.800 usec
DE 6.50 usec
TE 299.2 K
D1 2.00000000 sec
D11 0.03000000 sec
TD0 1
SFO1 100.6278593 MHz
NUC1 13C
P1 10.58 usec
PLW1 68.07700348 W
SFO2 400.1516006 MHz
NUC2 1H
CPDPRG[2 waltz16
PCPD2 80.00 usec
PLW2 15.00300026 W
PLW12 0.22421999 W
PLW13 0.11260000 W

F2 - Processing parameters
SI 32768
SF 100.6177975 MHz
WDW EM
SSB 0
LB 1.00 Hz
GB 0
PC 1.40

128i

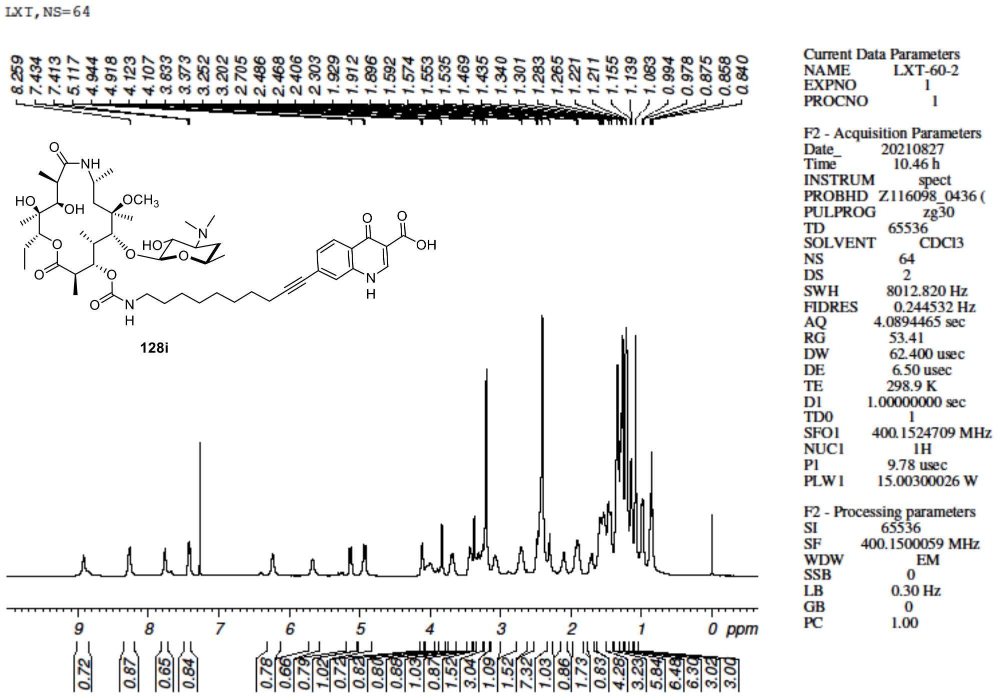
LXT,NS=64
8.259 7.434 7.413 5.117 4.944 4.918 4.123 4.107 3.833 3.373 3.252 3.202 2.705 2.486 2.468 2.406 2.303 1.929 1.912 1.896 1.592 1.574 1.553 1.535 1.469 1.435 1.340 1.301 1.283 1.265 1.221 1.211 1.155 1.139 1.083 0.994 0.978 0.875 0.858 0.840
O
NH
HO
OH
OCH3
N
HO
O
O
O
OH
O
N
H
O
N
H
128i
9 8 7 6 5 4 3 2 1 0 ppm
0.72 0.87 0.65 0.84 0.78 0.66 0.79 1.02 0.74 0.82 0.80 0.88 1.03 0.87 1.52 3.04 1.09 1.52 7.32 1.03 0.86 1.73 0.83 4.28 3.23 5.84 6.49 6.30 3.02 3.00
Current Data Parameters
NAME LXT-60-2
EXPNO 1
PROCNO 1
F2 - Acquisition Parameters
Date_ 20210827
Time 10.46 h
INSTRUM spect
PROBHD Z116098_0436 (
PULPROG zg30
TD 65536
SOLVENT CDCl3
NS 64
DS 2
SWH 8012.820 Hz
FIDRES 0.244532 Hz
AQ 4.0894465 sec
RG 53.41
DW 62.400 usec
DE 6.50 usec
TE 298.9 K
D1 1.00000000 sec
TD0 1
SFO1 400.1524709 MHz
NUC1 1H
P1 9.78 usec
PLW1 15.00300026 W
F2 - Processing parameters
SI 65536
SF 400.1500059 MHz
WDW EM
SSB 0
LB 0.30 Hz
GB 0
PC 1.00

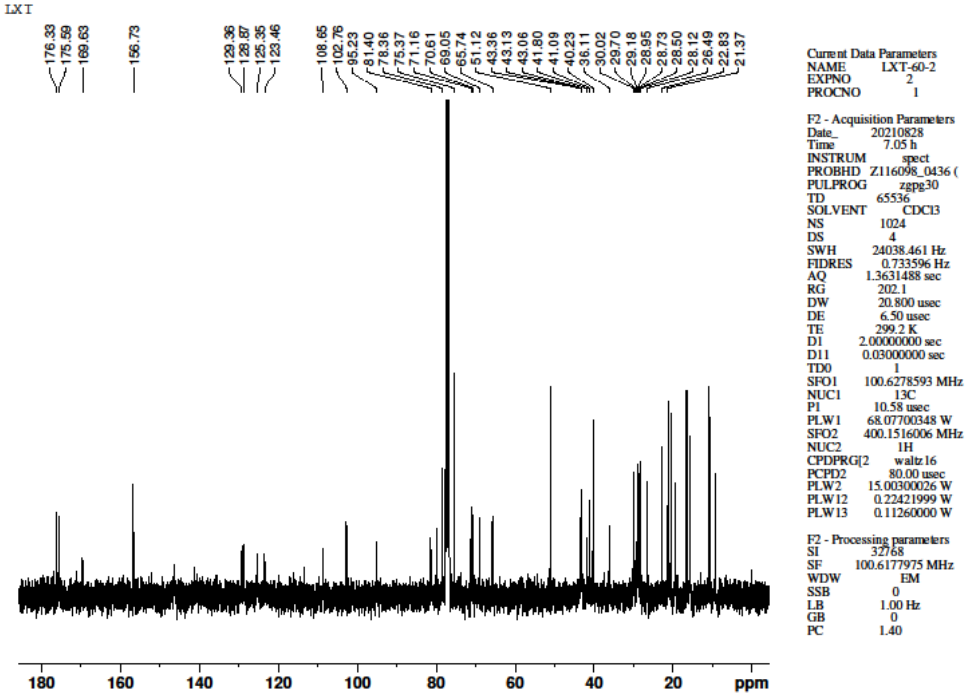
LXT
176.33 175.59 169.63 156.73 129.36 128.87 125.35 123.46 108.65 102.76 95.23 81.40 78.36 75.37 71.16 70.61 69.05 65.74 51.12 43.36 43.13 43.06 41.80 41.09 40.23 36.11 30.02 29.70 29.18 28.95 28.73 28.50 28.12 26.49 22.83 21.37
180 160 140 120 100 80 60 40 20 ppm
Current Data Parameters
NAME LXT-60-2
EXPNO 2
PROCNO 1
F2 - Acquisition Parameters
Date_ 20210828
Time 7.05 h
INSTRUM spect
PROBHD Z116098_0436 (
PULPROG zgpg30
TD 65536
SOLVENT CDCl3
NS 1024
DS 4
SWH 24038.461 Hz
FIDRES 0.733596 Hz
AQ 1.3631488 sec
RG 202.1
DW 20.800 usec
DE 6.50 usec
TE 299.2 K
D1 2.00000000 sec
D11 0.03000000 sec
TD0 1
SFO1 100.6278593 MHz
NUC1 13C
P1 10.58 usec
PLW1 68.07700348 W
SFO2 400.1516006 MHz
NUC2 1H
CPDPRG[2 waltz16
PCPD2 80.00 usec
PLW2 15.00300026 W
PLW12 0.22421999 W
PLW13 0.11260000 W
F2 - Processing parameters
SI 32768
SF 100.6177975 MHz
WDW EM
SSB 0
LB 1.00 Hz
GB 0
PC 1.40

129g

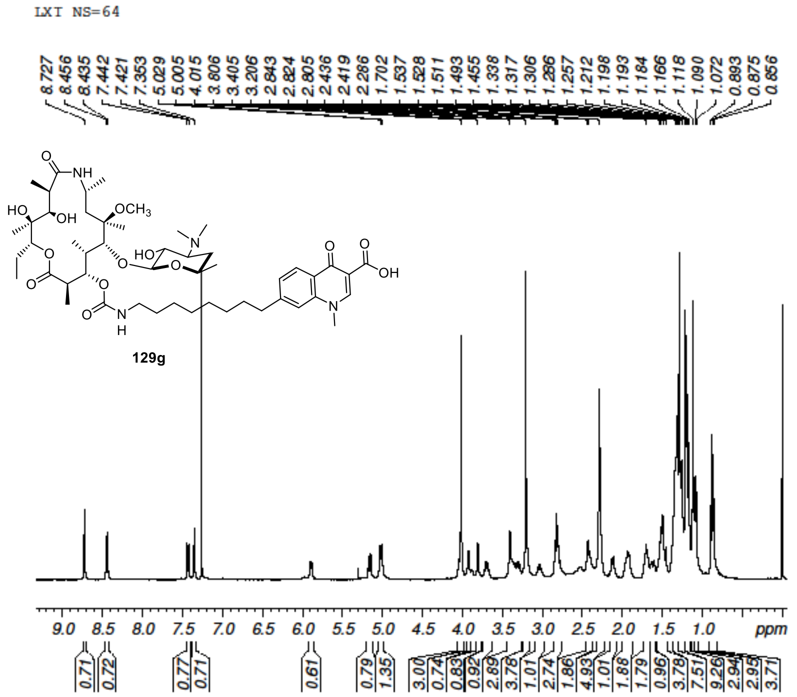

Current Data Parameters
NAME LXT-70
EXPNO 1
PROCNO 1

F2 - Acquisition Parameters
Date_ 20211220
Time 17.21 h
INSTRUM spect
PROBHD Z116098_0436 (
PULPROG zg30
TD 65536
SOLVENT CDCl3
NS 64
DS 2
SWH 8012.820 Hz
FIDRES 0.244532 Hz
AQ 4.0894465 sec
RG 89.02
DW 62.400 usec
DE 6.50 usec
TE 298.0 K
D1 1.00000000 sec
TD0 1
SFO1 400.1524709 MHz
NUC1 1H
P1 9.78 usec
PLW1 15.00300026 W

F2 - Processing parameters
SI 65536
SF 400.1500087 MHz
WDW EM
SSB 0
LB 0.30 Hz
GB 0
PC 1.00

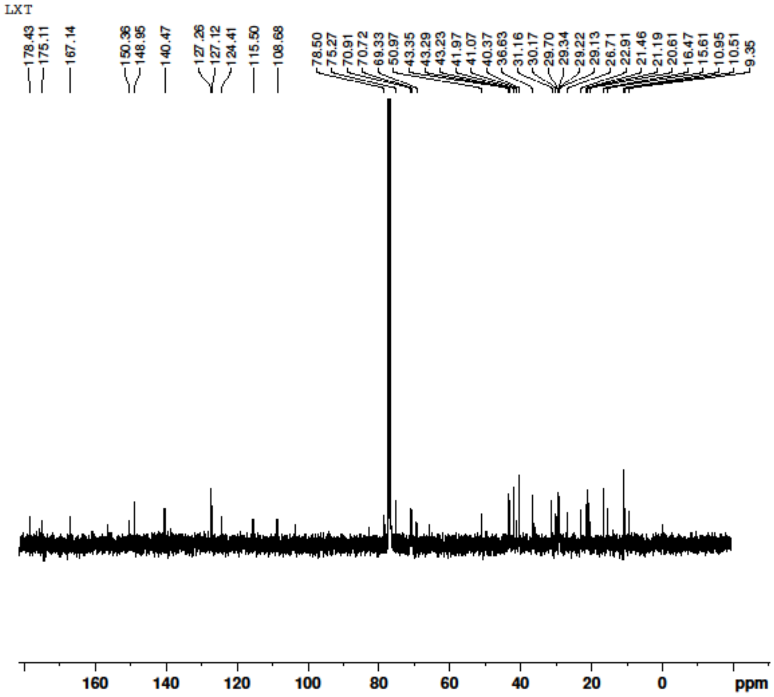

Current Data Parameters
NAME LXT-70
EXPNO 2
PROCNO 1

F2 - Acquisition Parameters
Date_ 20211222
Time 6.57 h
INSTRUM spect
PROBHD Z116098_0436 (
PULPROG zgpg30
TD 65536
SOLVENT CDCl3
NS 1024
DS 4
SWH 24038.461 Hz
FIDRES 0.733596 Hz
AQ 1.3631488 sec
RG 202.1
DW 20.800 usec
DE 6.50 usec
TE 298.0 K
D1 2.00000000 sec
D11 0.03000000 sec
TD0 1
SFO1 100.6278593 MHz
NUC1 13C
P1 10.58 usec
PLW1 68.07700348 W
SFO2 400.1516006 MHz
NUC2 1H
CPDPRG[2 waltz16
PCPD2 80.00 usec
PLW2 15.00300026 W
PLW12 0.22421999 W
PLW13 0.11260000 W

F2 - Processing parameters
SI 32768
SF 100.6177975 MHz
WDW EM
SSB 0
LB 1.00 Hz
GB 0
PC 1.40

129i

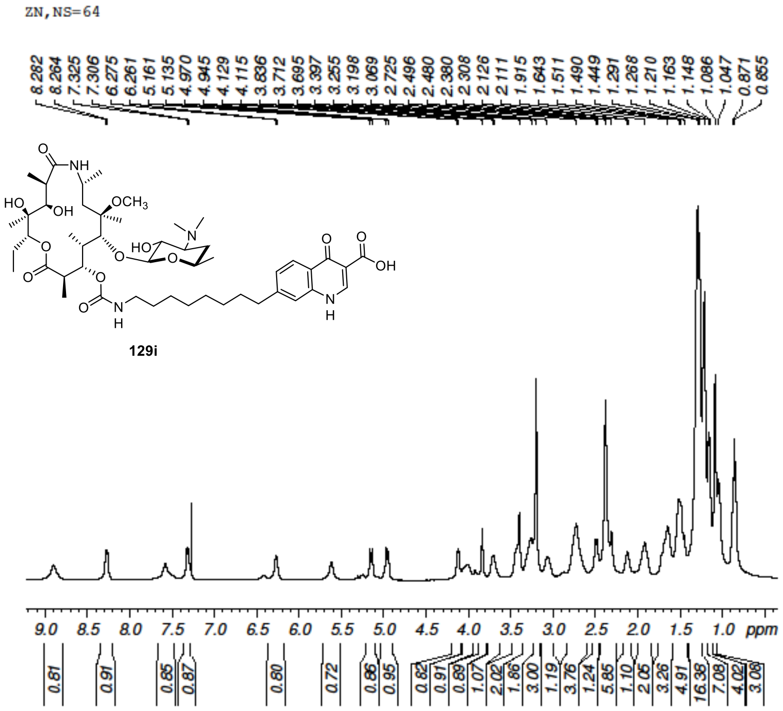

Current Data Parameters
NAME ZN-2
EXPNO 1
PROCNO 1

F2 - Acquisition Parameters
Date_ 20211007
Time 12.49 h
INSTRUM spect
PROBHD Z116098_0436 (
PULPROG zg30
TD 65536
SOLVENT CDCl3
NS 64
DS 2
SWH 8012.820 Hz
FIDRES 0.244532 Hz
AQ 4.0894465 sec
RG 30.51
DW 62.400 usec
DE 6.50 usec
TE 298.0 K
D1 1.00000000 sec
TD0 1
SFO1 400.1524709 MHz
NUC1 1H
P1 9.78 usec
PLW1 15.00300026 W

F2 - Processing parameters
SI 65536
SF 400.1500052 MHz
WDW EM
SSB 0
LB 0.30 Hz
GB 0
PC 1.00

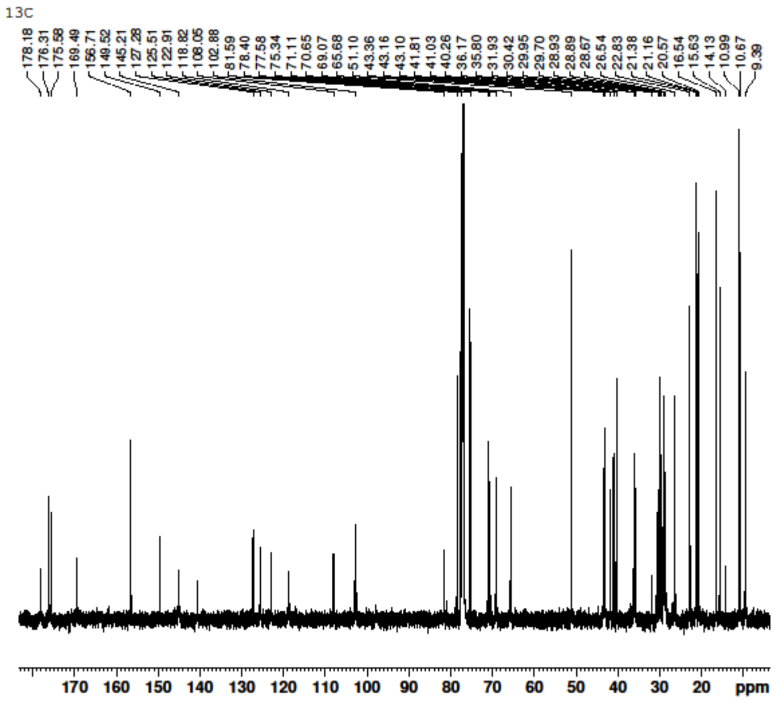

Current Data Parameters
NAME ZN-2-C
EXPNO 1
PROCNO 1

F2 - Acquisition Parameters
Date_ 20211011
Time 17.56 h
INSTRUM spect
PROBHD Z152837_0001 (
PULPROG zgpg30
TD 65536
SOLVENT CDCl3
NS 466
DS 4
SWH 42613.637 Hz
FIDRES 0.650232 Hz
AQ 0.7689557 sec
RG 187.62
DW 11.733 usec
DE 18.00 usec
TE 298.0 K
D1 2.00000000 sec
D11 0.03000000 sec
TD0 1
SFO1 176.0704628 MHz
NUC1 13C
P1 12.00 usec
PLW1 26.46899986 W
SFO2 700.1528006 MHz
NUC2 1H
CPDPRG[2 waltz16
PCPD2 65.00 usec
PLW2 10.69499969 W
PLW12 0.36452001 W
PLW13 0.18355000 W

F2 - Processing parameters
SI 32768
SF 176.0528575 MHz
WDW EM
SSB 0
LB 1.00 Hz
GB 0
PC 1.40

130i

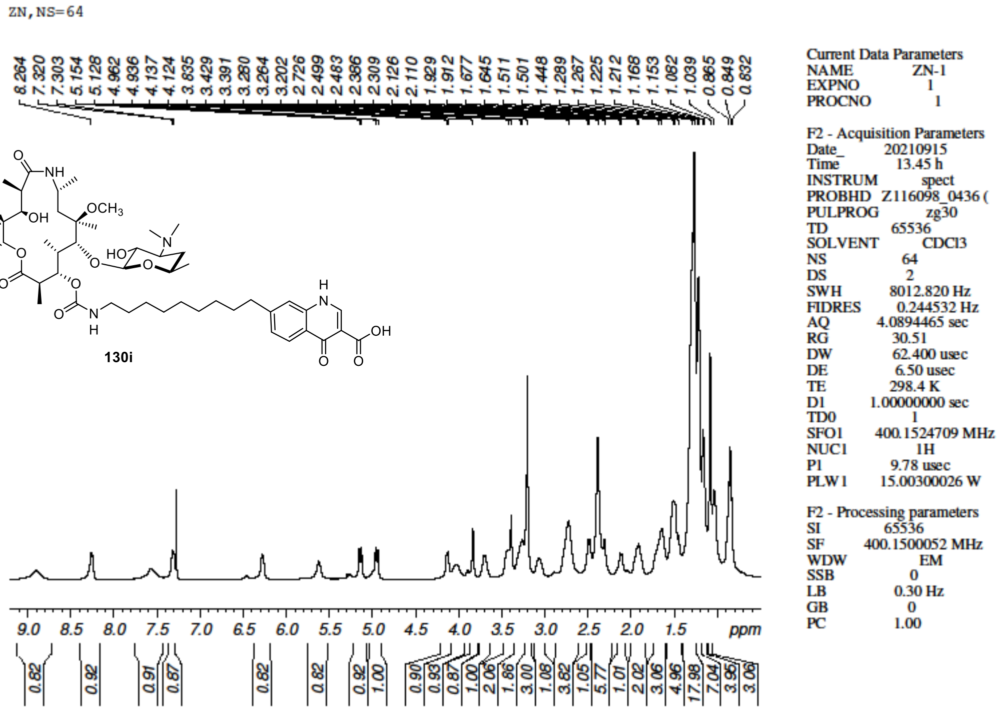
ZN,NS=64
8.264 7.320 7.303 5.154 5.128 4.962 4.936 4.137 4.124 3.835 3.429 3.391 3.280 3.264 3.202 2.726 2.499 2.483 2.386 2.309 2.126 2.110 1.929 1.912 1.677 1.645 1.511 1.501 1.448 1.289 1.267 1.225 1.212 1.168 1.153 1.082 1.039 0.865 0.849 0.832
OCH3
HO
OH
NH
130i
OH
9.0 8.5 8.0 7.5 7.0 6.5 6.0 5.5 5.0 4.5 4.0 3.5 3.0 2.5 2.0 1.5 ppm
0.82 0.92 0.91 0.87 0.82 0.82 0.92 1.00 0.90 0.93 0.87 1.00 2.06 1.86 3.00 1.08 3.82 1.05 5.77 1.01 2.02 3.06 4.96 17.98 7.04 3.95 3.06
Current Data Parameters
NAME ZN-1
EXPNO 1
PROCNO 1
F2 - Acquisition Parameters
Date_ 20210915
Time 13.45 h
INSTRUM spect
PROBHD Z116098_0436 (
PULPROG zg30
TD 65536
SOLVENT CDCl3
NS 64
DS 2
SWH 8012.820 Hz
FIDRES 0.244532 Hz
AQ 4.0894465 sec
RG 30.51
DW 62.400 usec
DE 6.50 usec
TE 298.4 K
D1 1.00000000 sec
TD0 1
SFO1 400.1524709 MHz
NUC1 1H
P1 9.78 usec
PLW1 15.00300026 W
F2 - Processing parameters
SI 65536
SF 400.1500052 MHz
WDW EM
SSB 0
LB 0.30 Hz
GB 0
PC 1.00

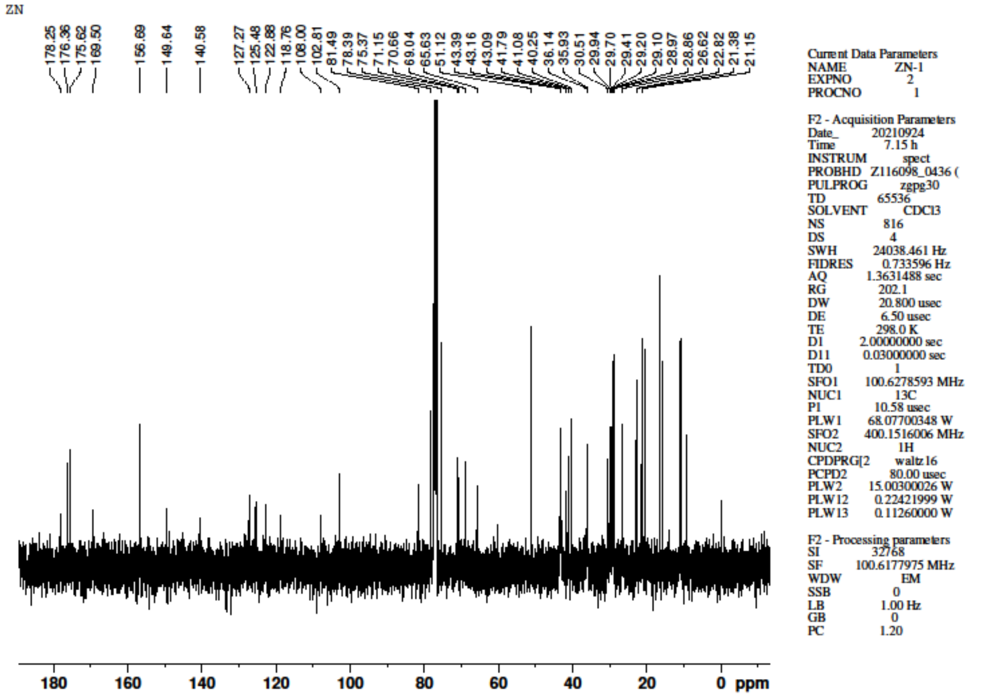
ZN
178.25 176.36 175.62 169.50 156.69 149.64 140.58 127.27 125.48 122.88 118.76 108.00 102.81 81.49 78.39 75.37 71.15 70.66 69.04 65.63 51.12 43.39 43.16 43.09 41.79 41.08 40.25 36.14 35.93 30.51 29.94 29.70 29.41 29.20 29.10 28.97 28.86 26.62 22.82 21.38 21.15
180 160 140 120 100 80 60 40 20 0 ppm
Current Data Parameters
NAME ZN-1
EXPNO 2
PROCNO 1
F2 - Acquisition Parameters
Date_ 20210924
Time 7.15 h
INSTRUM spect
PROBHD Z116098_0436 (
PULPROG zgpg30
TD 65536
SOLVENT CDCl3
NS 816
DS 4
SWH 24038.461 Hz
FIDRES 0.733596 Hz
AQ 1.3631488 sec
RG 202.1
DW 20.800 usec
DE 6.50 usec
TE 298.0 K
D1 2.00000000 sec
D11 0.03000000 sec
TD0 1
SFO1 100.6278593 MHz
NUC1 13C
P1 10.58 usec
PLW1 68.07700348 W
SFO2 400.1516006 MHz
NUC2 1H
CPDPRG[2 waltz16
PCPD2 80.00 usec
PLW2 15.00300026 W
PLW12 0.22421999 W
PLW13 0.11260000 W
F2 - Processing parameters
SI 32768
SF 100.6177975 MHz
WDW EM
SSB 0
LB 1.00 Hz
GB 0
PC 1.20

131i

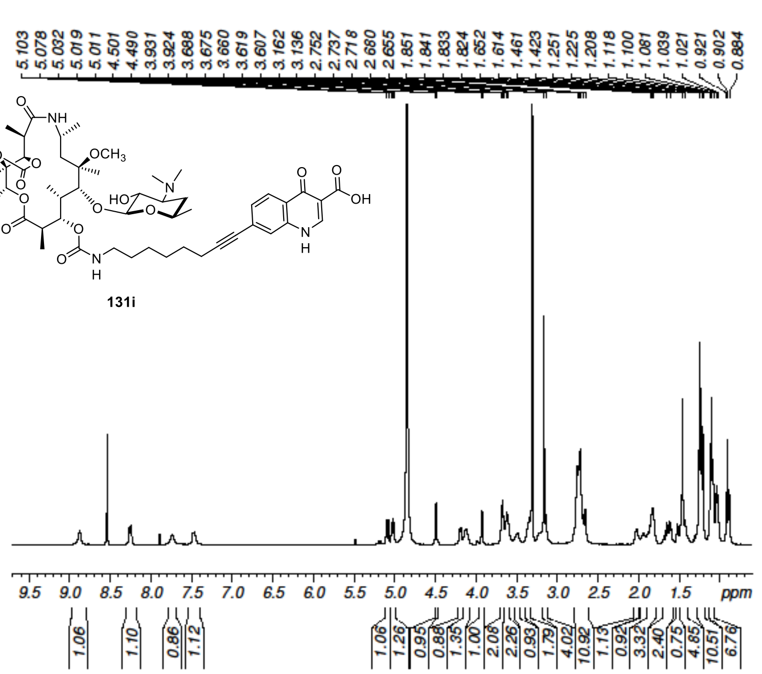

Current Data Parameters
NAME LXT-62-3
EXPNO 1
PROCNO 1

F2 - Acquisition Parameters
Date_ 20210903
Time 16.32 h
INSTRUM spect
PROBHD Z116098_0436 (
PULPROG zg30
TD 65536
SOLVENT MeOD
NS 64
DS 2
SWH 8012.820 Hz
FIDRES 0.244532 Hz
AQ 4.0894465 sec
RG 98.25
DW 62.400 usec
DE 6.50 usec
TE 300.2 K
D1 1.00000000 sec
TD0 1
SFO1 400.1524709 MHz
NUC1 1H
P1 9.78 usec
PLW1 15.00300026 W

F2 - Processing parameters
SI 65536
SF 400.1500090 MHz
WDW EM
SSB 0
LB 0.30 Hz
GB 0
PC 1.00

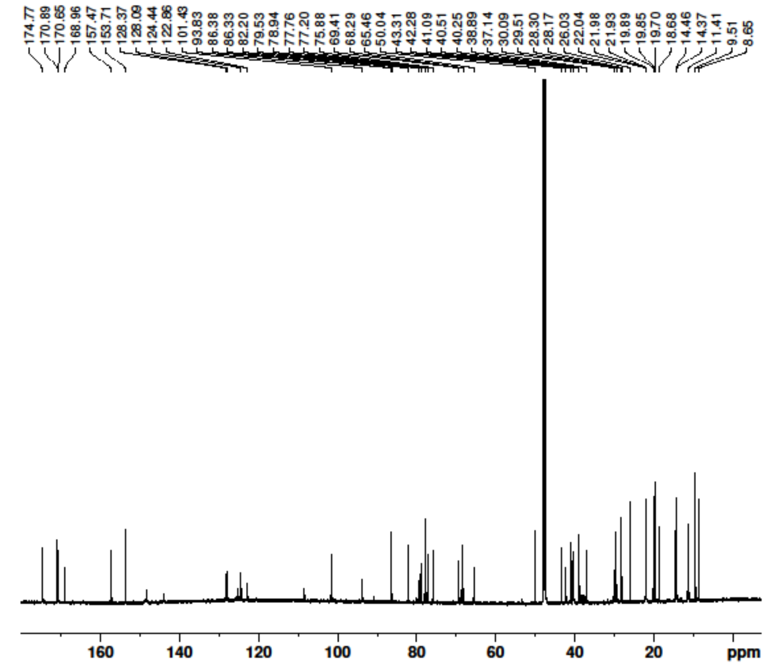

Current Data Parameters
NAME LXT-62-3-C-MeOD
EXPNO 1
PROCNO 1

F2 - Acquisition Parameters
Date_ 20211013
Time 9.40 h
INSTRUM spect
PROBHD Z152837_0001 (
PULPROG zgpg30
TD 65536
SOLVENT MeOD
NS 342
DS 4
SWH 42613.637 Hz
FIDRES 0.650232 Hz
AQ 0.7689557 sec
RG 187.62
DW 11.733 usec
DE 18.00 usec
TE 298.0 K
D1 2.00000000 sec
D11 0.03000000 sec
TD0 1
SFO1 176.0704628 MHz
NUC1 13C
P1 12.00 usec
PLW1 26.46899986 W
SFO2 700.1528006 MHz
NUC2 1H
CPDPRG[2 waltz16
PCPD2 65.00 usec
PLW2 10.69499969 W
PLW12 0.36452001 W
PLW13 0.18355000 W

F2 - Processing parameters
SI 32768
SF 176.0528575 MHz
WDW EM
SSB 0
LB 1.00 Hz
GB 0
PC 1.10

137i

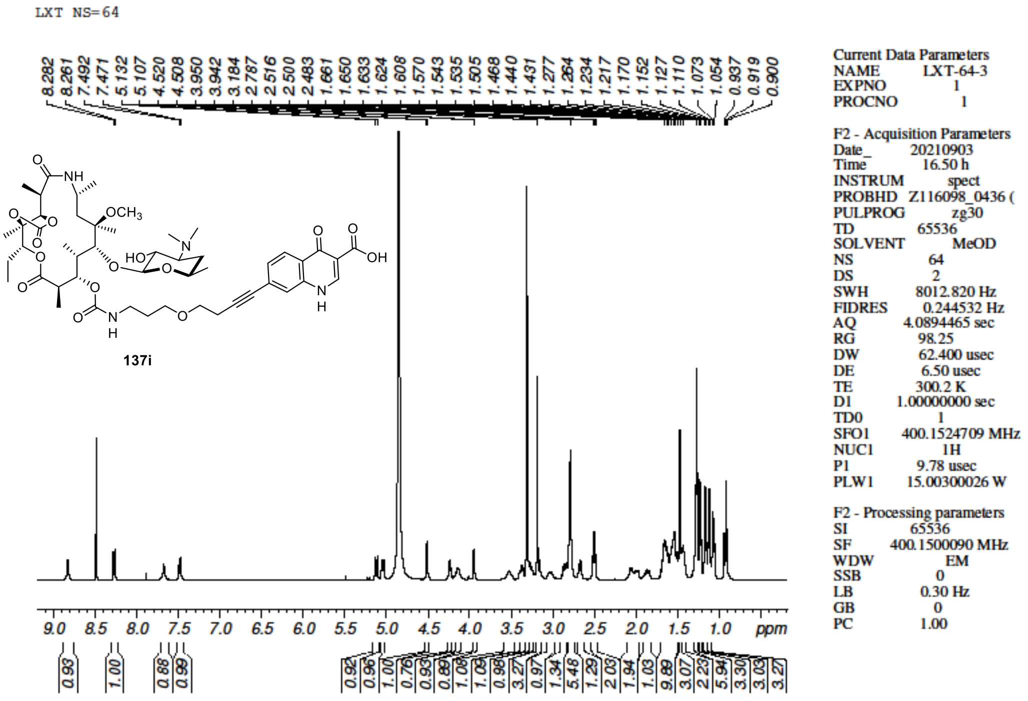
LXT NS=64
8.282 8.261 7.492 7.471 5.132 5.107 4.520 4.508 3.950 3.942 3.184 2.787 2.516 2.500 2.483 1.661 1.650 1.633 1.624 1.608 1.570 1.543 1.535 1.505 1.468 1.440 1.431 1.277 1.264 1.234 1.217 1.170 1.152 1.127 1.110 1.073 1.054 0.937 0.919 0.900
137i
9.0 8.5 8.0 7.5 7.0 6.5 6.0 5.5 5.0 4.5 4.0 3.5 3.0 2.5 2.0 1.5 1.0 ppm
0.93 1.00 0.88 0.99 0.92 0.96 1.00 0.76 0.93 0.89 1.08 1.03 0.98 3.27 0.97 1.34 5.48 1.29 2.03 1.94 1.03 9.89 3.07 2.23 5.94 3.30 3.03 3.27
Current Data Parameters
NAME LXT-64-3
EXPNO 1
PROCNO 1
F2 - Acquisition Parameters
Date_ 20210903
Time 16.50 h
INSTRUM spect
PROBHD Z116098_0436 (
PULPROG zg30
TD 65536
SOLVENT MeOD
NS 64
DS 2
SWH 8012.820 Hz
FIDRES 0.244532 Hz
AQ 4.0894465 sec
RG 98.25
DW 62.400 usec
DE 6.50 usec
TE 300.2 K
D1 1.00000000 sec
TD0 1
SFO1 400.1524709 MHz
NUC1 1H
P1 9.78 usec
PLW1 15.00300026 W
F2 - Processing parameters
SI 65536
SF 400.1500090 MHz
WDW EM
SSB 0
LB 0.30 Hz
GB 0
PC 1.00

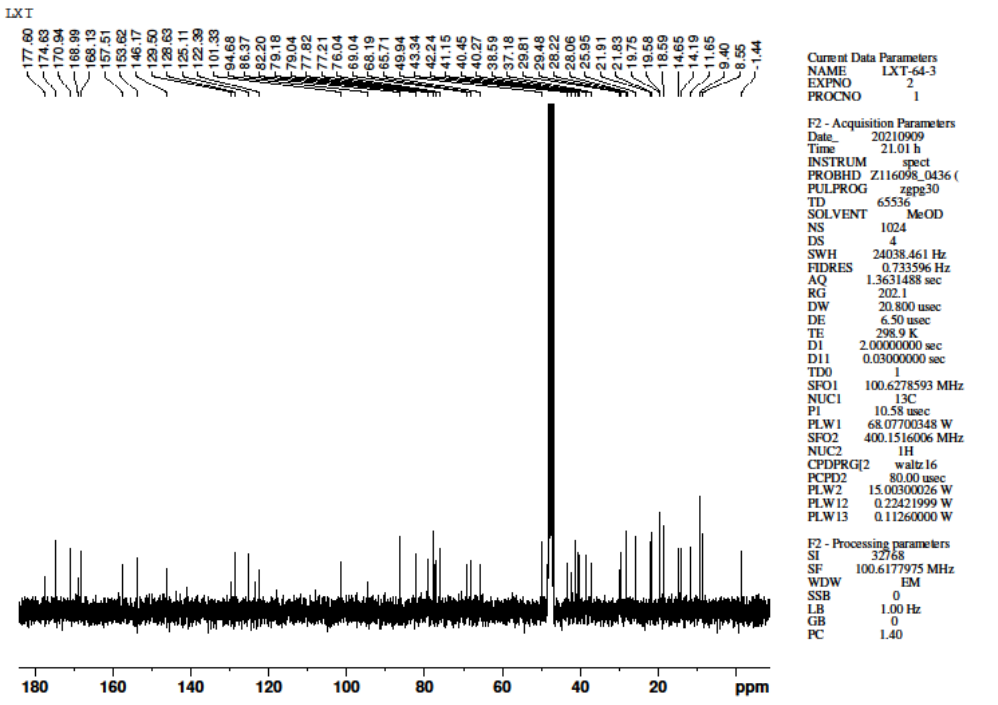
LXT
177.60 174.63 170.94 168.99 168.13 157.51 153.62 146.17 129.50 128.63 125.11 122.39 101.33 94.68 86.37 82.20 79.18 79.04 77.82 77.21 76.04 69.04 68.19 65.71 49.94 43.34 42.24 41.15 40.45 40.27 38.59 37.18 29.81 29.48 28.22 28.06 25.95 21.91 21.83 19.75 19.58 18.59 14.65 14.19 11.65 9.40 8.55 -1.44
180 160 140 120 100 80 60 40 20 ppm
Current Data Parameters
NAME LXT-64-3
EXPNO 2
PROCNO 1
F2 - Acquisition Parameters
Date_ 20210909
Time 21.01 h
INSTRUM spect
PROBHD Z116098_0436 (
PULPROG zgpg30
TD 65536
SOLVENT MeOD
NS 1024
DS 4
SWH 24038.461 Hz
FIDRES 0.733596 Hz
AQ 1.3631488 sec
RG 202.1
DW 20.800 usec
DE 6.50 usec
TE 298.9 K
D1 2.00000000 sec
D11 0.03000000 sec
TD0 1
SFO1 100.6278593 MHz
NUC1 13C
P1 10.58 usec
PLW1 68.07700348 W
SFO2 400.1516006 MHz
NUC2 1H
CPDPRG[2 waltz16
PCPD2 80.00 usec
PLW2 15.00300026 W
PLW12 0.22421999 W
PLW13 0.11260000 W
F2 - Processing parameters
SI 32768
SF 100.6177975 MHz
WDW EM
SSB 0
LB 1.00 Hz
GB 0
PC 1.40

141i

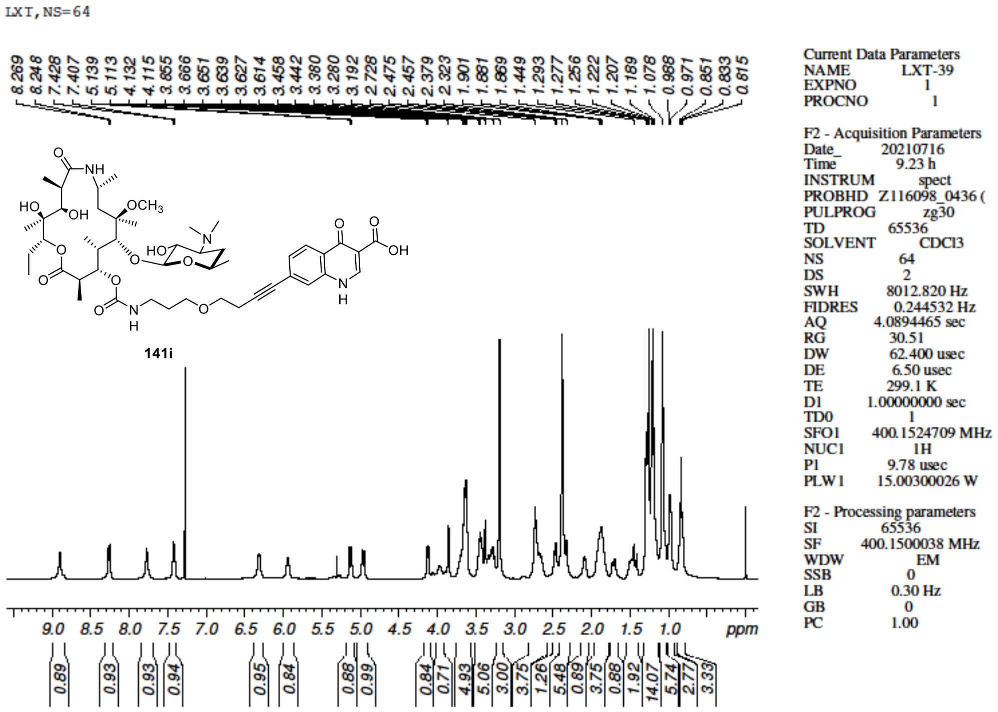
LXT,NS=64
8.269
8.248
7.428
7.407
5.139
5.113
4.132
4.115
3.855
3.666
3.651
3.639
3.627
3.614
3.458
3.442
3.380
3.280
3.192
2.728
2.475
2.457
2.379
2.323
1.901
1.881
1.869
1.449
1.293
1.277
1.256
1.222
1.207
1.189
1.078
0.988
0.971
0.851
0.833
0.815
HO
OH
NH
O
OCH3
N
HO
O
O
O
OH
N
H
O
N
H
141i
9.0 8.5 8.0 7.5 7.0 6.5 6.0 5.5 5.0 4.5 4.0 3.5 3.0 2.5 2.0 1.5 1.0 ppm
0.89
0.93
0.93
0.94
0.95
0.84
0.88
0.99
0.84
0.71
4.93
5.06
3.00
3.75
1.26
5.48
0.89
3.75
0.88
1.92
14.07
5.74
2.77
3.33
Current Data Parameters
NAME LXT-39
EXPNO 1
PROCNO 1
F2 - Acquisition Parameters
Date_ 20210716
Time 9.23 h
INSTRUM spect
PROBHD Z116098_0436 (
PULPROG zg30
TD 65536
SOLVENT CDCl3
NS 64
DS 2
SWH 8012.820 Hz
FIDRES 0.244532 Hz
AQ 4.0894465 sec
RG 30.51
DW 62.400 usec
DE 6.50 usec
TE 299.1 K
D1 1.00000000 sec
TD0 1
SFO1 400.1524709 MHz
NUC1 1H
P1 9.78 usec
PLW1 15.00300026 W
F2 - Processing parameters
SI 65536
SF 400.1500038 MHz
WDW EM
SSB 0
LB 0.30 Hz
GB 0
PC 1.00

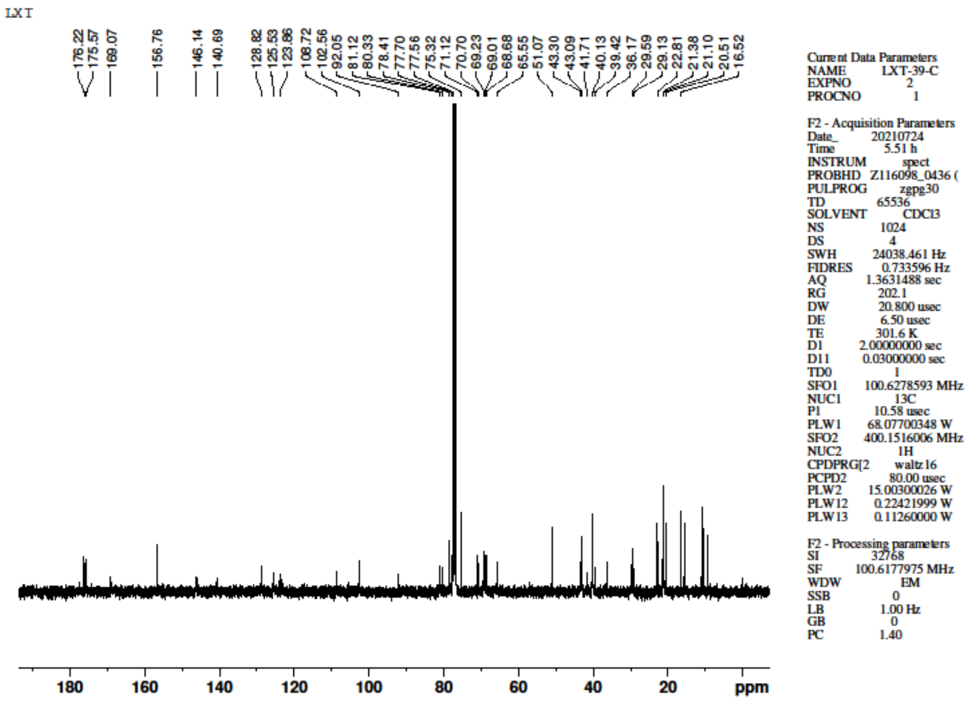
LXT
176.22
175.57
169.07
156.76
146.14
140.69
128.82
125.53
123.86
108.72
102.56
92.05
81.12
80.33
78.41
77.70
77.56
75.32
71.12
70.70
69.23
69.01
68.68
65.55
51.07
43.30
43.09
41.71
40.13
39.42
36.17
29.59
29.13
22.81
21.38
21.10
20.51
16.52
180 160 140 120 100 80 60 40 20 ppm
Current Data Parameters
NAME LXT-39-C
EXPNO 2
PROCNO 1
F2 - Acquisition Parameters
Date_ 20210724
Time 5.51 h
INSTRUM spect
PROBHD Z116098_0436 (
PULPROG zgpg30
TD 65536
SOLVENT CDCl3
NS 1024
DS 4
SWH 24038.461 Hz
FIDRES 0.733596 Hz
AQ 1.3631488 sec
RG 202.1
DW 20.800 usec
DE 6.50 usec
TE 301.6 K
D1 2.00000000 sec
D11 0.03000000 sec
TD0 1
SFO1 100.6278593 MHz
NUC1 13C
P1 10.58 usec
PLW1 68.07700348 W
SFO2 400.1516006 MHz
NUC2 1H
CPDPRG[2 waltz16
PCPD2 80.00 usec
PLW2 15.00300026 W
PLW12 0.22421999 W
PLW13 0.11260000 W
F2 - Processing parameters
SI 32768
SF 100.6177975 MHz
WDW EM
SSB 0
LB 1.00 Hz
GB 0
PC 1.40

141j

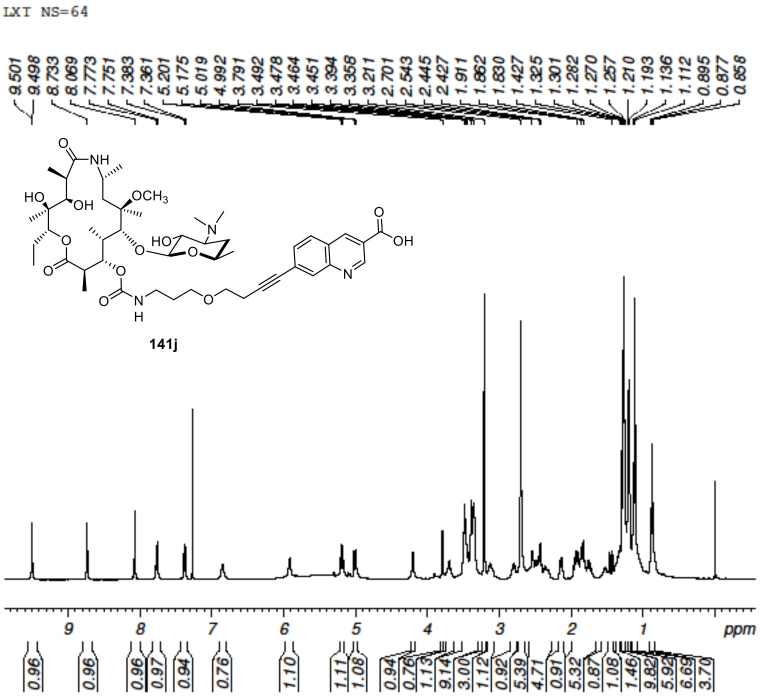

Current Data Parameters
NAME LXT-65
EXPNO 1
PROCNO 1

F2 - Acquisition Parameters
Date_ 20210903
Time 16.59 h
INSTRUM spect
PROBHD Z116098_0436 (
PULPROG zg30
TD 65536
SOLVENT CDCl3
NS 64
DS 2
SWH 8012.820 Hz
FIDRES 0.244532 Hz
AQ 4.0894465 sec
RG 79.39
DW 62.400 usec
DE 6.50 usec
TE 300.2 K
D1 1.00000000 sec
TD0 1
SFO1 400.1524709 MHz
NUC1 1H
P1 9.78 usec
PLW1 15.00300026 W

F2 - Processing parameters
SI 65536
SF 400.1500066 MHz
WDW EM
SSB 0
LB 0.30 Hz
GB 0
PC 1.00

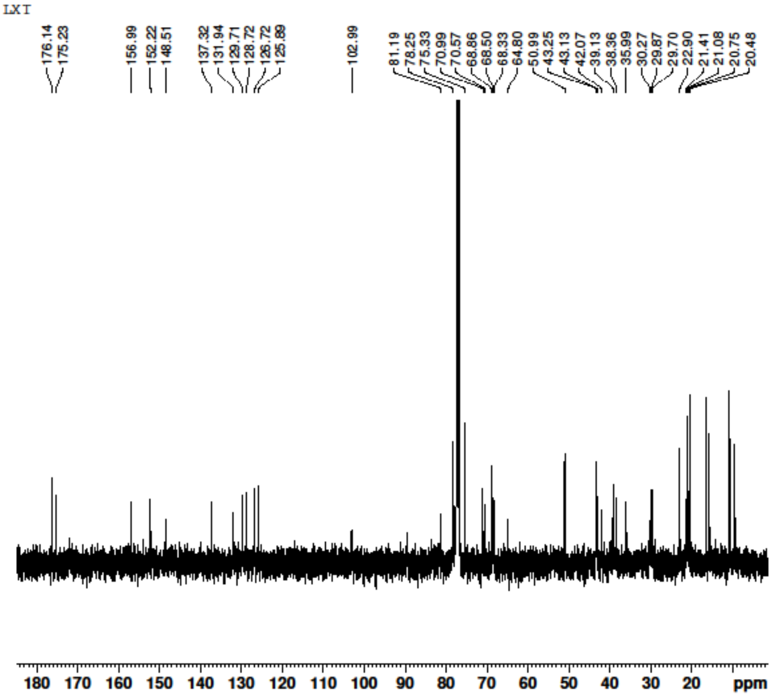

Current Data Parameters
NAME LXT-65
EXPNO 2
PROCNO 1

F2 - Acquisition Parameters
Date_ 20210909
Time 8.28 h
INSTRUM spect
PROBHD Z116098_0436 (
PULPROG zgpg30
TD 65536
SOLVENT CDCl3
NS 1024
DS 4
SWH 24038.461 Hz
FIDRES 0.733596 Hz
AQ 1.3631488 sec
RG 202.1
DW 20.800 usec
DE 6.50 usec
TE 298.9 K
D1 2.00000000 sec
D11 0.03000000 sec
TD0 1
SFO1 100.6278593 MHz
NUC1 13C
P1 10.58 usec
PLW1 68.07700348 W
SFO2 400.1516006 MHz
NUC2 1H
CPDPRG[2 waltz16
PCPD2 80.00 usec
PLW2 15.00300026 W
PLW12 0.22421999 W
PLW13 0.11260000 W

F2 - Processing parameters
SI 32768
SF 100.6177975 MHz
WDW EM
SSB 0
LB 1.00 Hz
GB 0
PC 1.40

142i

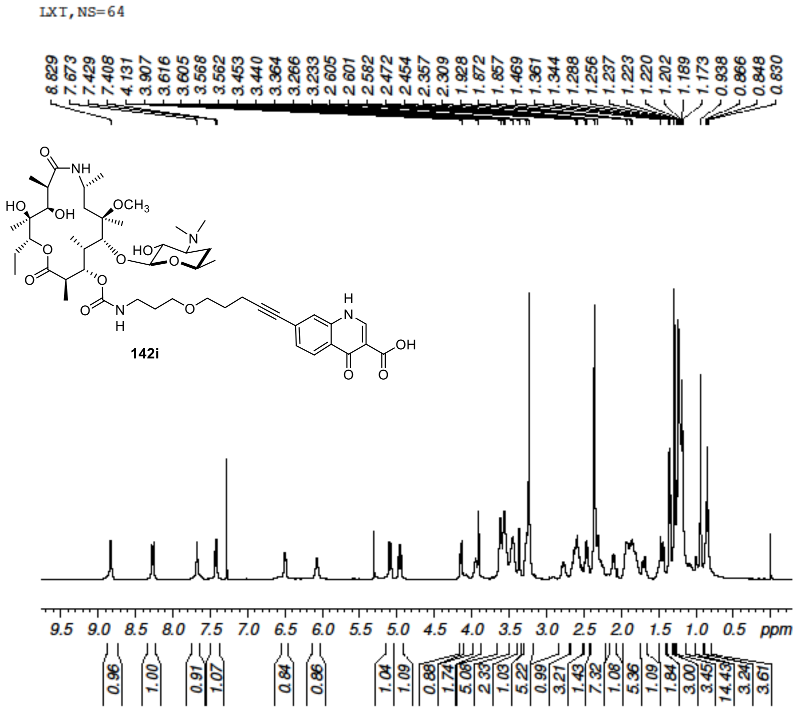

Current Data Parameters
NAME LXT-67
EXPNO 1
PROCNO 1

F2 - Acquisition Parameters
Date_ 20210908
Time 12.41 h
INSTRUM spect
PROBHD Z116098_0436 (
PULPROG zg30
TD 65536
SOLVENT CDCl3
NS 64
DS 2
SWH 8012.820 Hz
FIDRES 0.244532 Hz
AQ 4.0894465 sec
RG 53.41
DW 62.400 usec
DE 6.50 usec
TE 299.0 K
D1 1.00000000 sec
TD0 1
SFO1 400.1524709 MHz
NUC1 1H
P1 9.78 usec
PLW1 15.00300026 W

F2 - Processing parameters
SI 65536
SF 400.1500042 MHz
WDW EM
SSB 0
LB 0.30 Hz
GB 0
PC 1.00

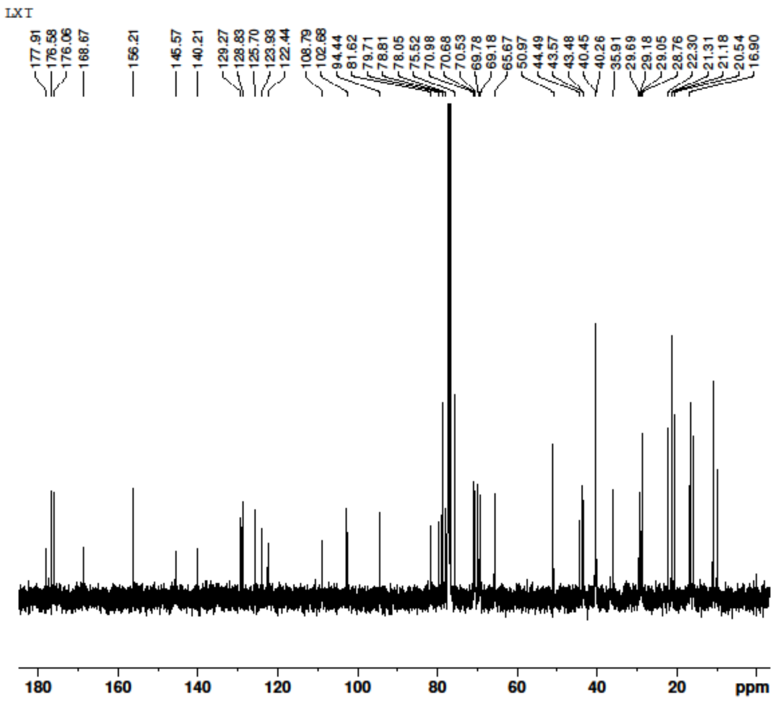

Current Data Parameters
NAME LXT-67
EXPNO 2
PROCNO 1

F2 - Acquisition Parameters
Date_ 20210909
Time 9.32 h
INSTRUM spect
PROBHD Z116098_0436 (
PULPROG zgpg30
TD 65536
SOLVENT CDCl3
NS 1024
DS 4
SWH 24038.461 Hz
FIDRES 0.733596 Hz
AQ 1.3631488 sec
RG 202.1
DW 20.800 usec
DE 6.50 usec
TE 298.9 K
D1 2.00000000 sec
D11 0.03000000 sec
TD0 1
SFO1 100.6278593 MHz
NUC1 13C
P1 10.58 usec
PLW1 68.07700348 W
SFO2 400.1516006 MHz
NUC2 1H
CPDPRG[2 waltz16
PCPD2 80.00 usec
PLW2 15.00300026 W
PLW12 0.22421999 W
PLW13 0.11260000 W

F2 - Processing parameters
SI 32768
SF 100.6177975 MHz
WDW EM
SSB 0
LB 1.00 Hz
GB 0
PC 1.40

143i

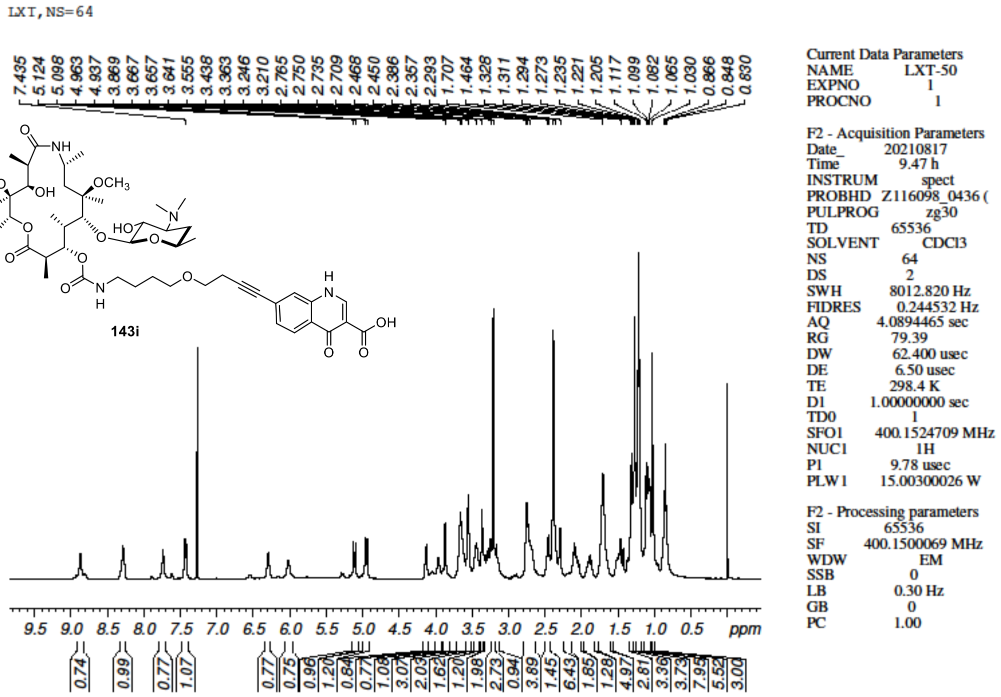
LXT,NS=64
143i
Current Data Parameters
NAME LXT-50
EXPNO 1
PROCNO 1
F2 - Acquisition Parameters
Date_ 20210817
Time 9.47 h
INSTRUM spect
PROBHD Z116098_0436 (
PULPROG zg30
TD 65536
SOLVENT CDCl3
NS 64
DS 2
SWH 8012.820 Hz
FIDRES 0.244532 Hz
AQ 4.0894465 sec
RG 79.39
DW 62.400 usec
DE 6.50 usec
TE 298.4 K
D1 1.00000000 sec
TD0 1
SFO1 400.1524709 MHz
NUC1 1H
P1 9.78 usec
PLW1 15.00300026 W
F2 - Processing parameters
SI 65536
SF 400.1500069 MHz
WDW EM
SSB 0
LB 0.30 Hz
GB 0
PC 1.00
ppm

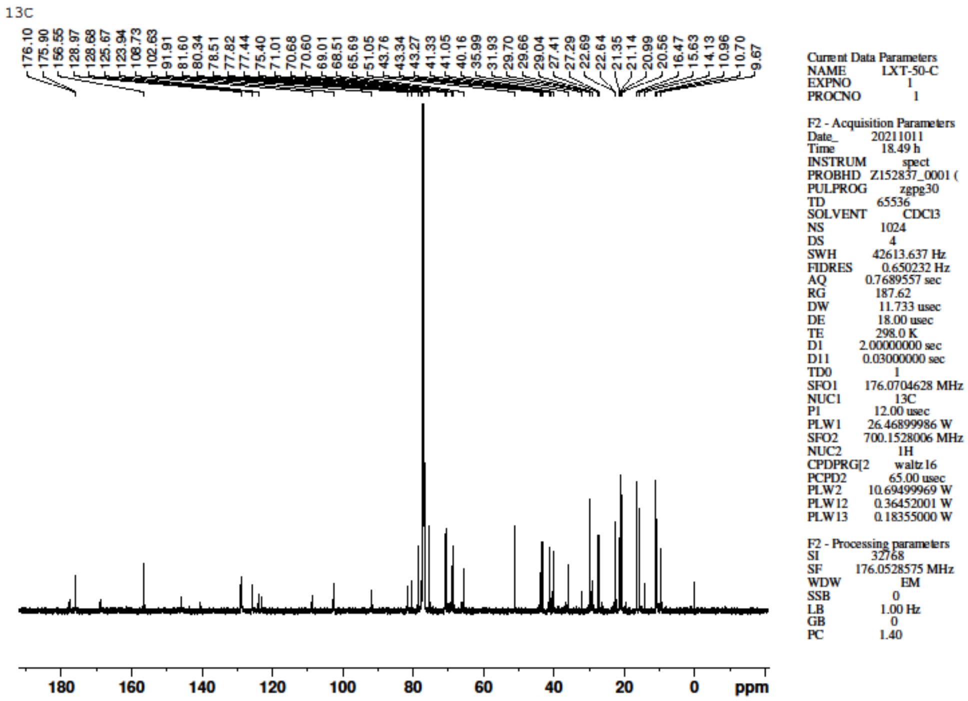
13C
Current Data Parameters
NAME LXT-50-C
EXPNO 1
PROCNO 1
F2 - Acquisition Parameters
Date_ 20211011
Time 18.49 h
INSTRUM spect
PROBHD Z152837_0001 (
PULPROG zgpg30
TD 65536
SOLVENT CDCl3
NS 1024
DS 4
SWH 42613.637 Hz
FIDRES 0.650232 Hz
AQ 0.7689557 sec
RG 187.62
DW 11.733 usec
DE 18.00 usec
TE 298.0 K
D1 2.00000000 sec
D11 0.03000000 sec
TD0 1
SFO1 176.0704628 MHz
NUC1 13C
P1 12.00 usec
PLW1 26.46899986 W
SFO2 700.1528006 MHz
NUC2 1H
CPDPRG[2 waltz16
PCPD2 65.00 usec
PLW2 10.69499969 W
PLW12 0.36452001 W
PLW13 0.18355000 W
F2 - Processing parameters
SI 32768
SF 176.0528575 MHz
WDW EM
SSB 0
LB 1.00 Hz
GB 0
PC 1.40
ppm

148i

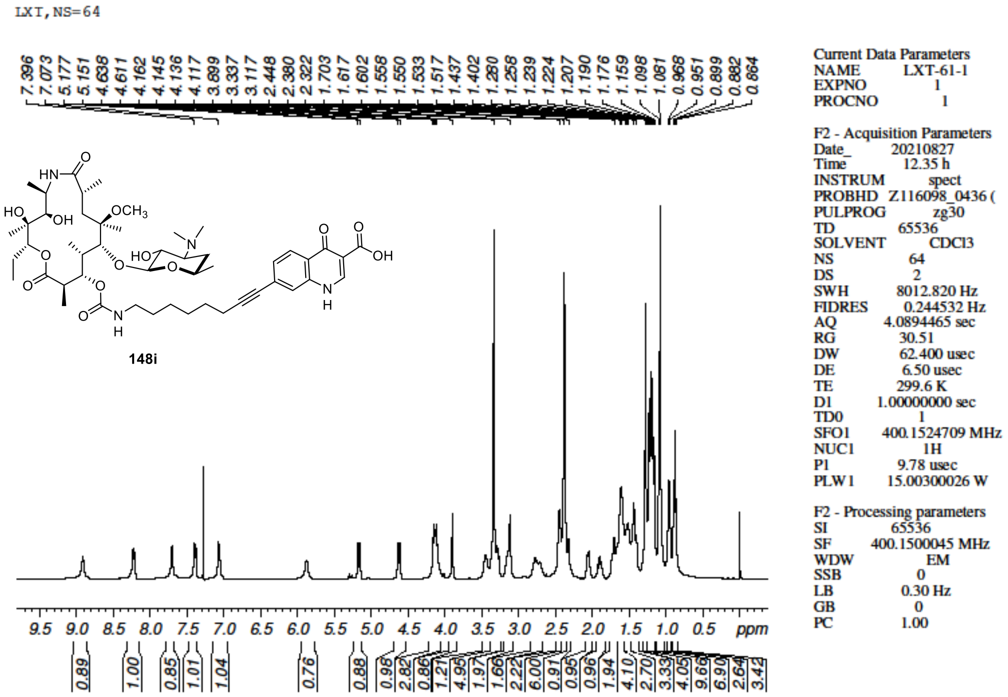
LXT,NS=64
148i
Current Data Parameters
NAME LXT-61-1
EXPNO 1
PROCNO 1
F2 - Acquisition Parameters
Date_ 20210827
Time 12.35 h
INSTRUM spect
PROBHD Z116098_0436 (
PULPROG zg30
TD 65536
SOLVENT CDCl3
NS 64
DS 2
SWH 8012.820 Hz
FIDRES 0.244532 Hz
AQ 4.0894465 sec
RG 30.51
DW 62.400 usec
DE 6.50 usec
TE 299.6 K
D1 1.00000000 sec
TD0 1
SFO1 400.1524709 MHz
NUC1 1H
P1 9.78 usec
PLW1 15.00300026 W
F2 - Processing parameters
SI 65536
SF 400.1500045 MHz
WDW EM
SSB 0
LB 0.30 Hz
GB 0
PC 1.00
ppm

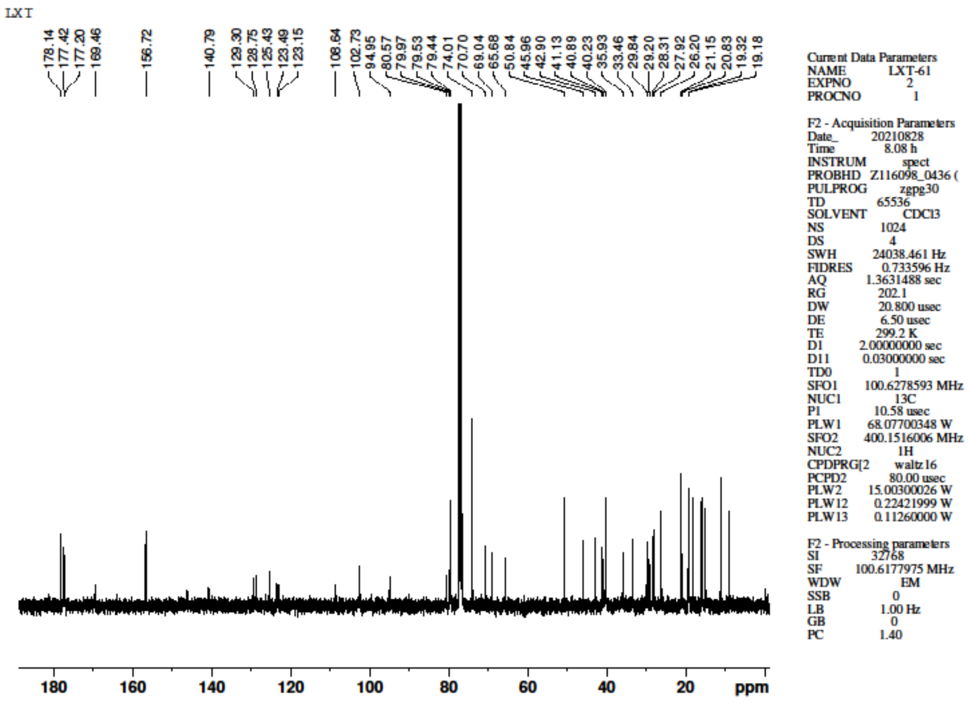
LXT
Current Data Parameters
NAME LXT-61
EXPNO 2
PROCNO 1
F2 - Acquisition Parameters
Date_ 20210828
Time 8.08 h
INSTRUM spect
PROBHD Z116098_0436 (
PULPROG zgpg30
TD 65536
SOLVENT CDCl3
NS 1024
DS 4
SWH 24038.461 Hz
FIDRES 0.733596 Hz
AQ 1.3631488 sec
RG 202.1
DW 20.800 usec
DE 6.50 usec
TE 299.2 K
D1 2.00000000 sec
D11 0.03000000 sec
TD0 1
SFO1 100.6278593 MHz
NUC1 13C
P1 10.58 usec
PLW1 68.07700348 W
SFO2 400.1516006 MHz
NUC2 1H
CPDPRG[2 waltz16
PCPD2 80.00 usec
PLW2 15.00300026 W
PLW12 0.22421999 W
PLW13 0.11260000 W
F2 - Processing parameters
SI 32768
SF 100.6177975 MHz
WDW EM
SSB 0
LB 1.00 Hz
GB 0
PC 1.40
ppm

149g

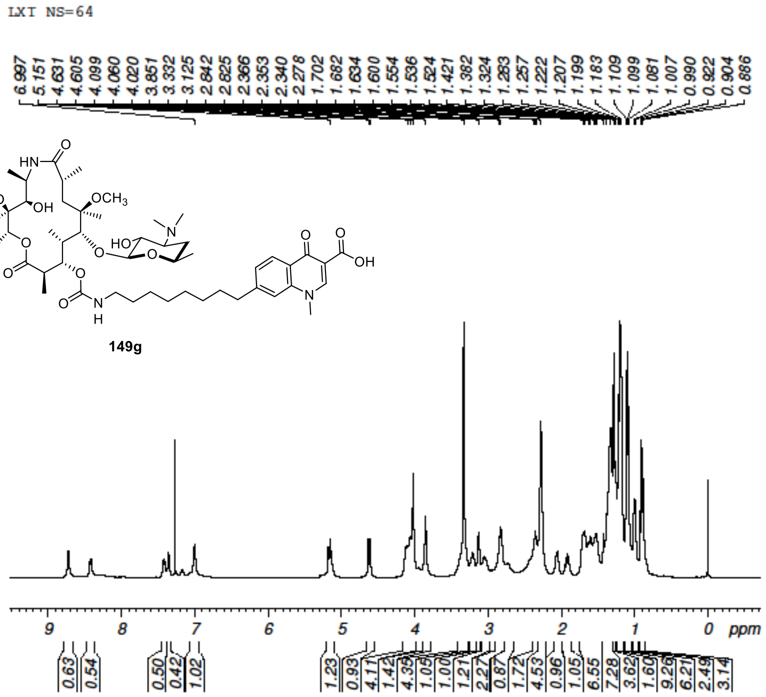

Current Data Parameters
NAME LXT-72
EXPNO 1
PROCNO 1

F2 - Acquisition Parameters
Date_ 20211228
Time 9.38 h
INSTRUM spect
PROBHD Z116098_0436 (
PULPROG zg30
TD 65536
SOLVENT CDCl3
NS 64
DS 2
SWH 8012.820 Hz
FIDRES 0.244532 Hz
AQ 4.0894465 sec
RG 30.51
DW 62.400 usec
DE 6.50 usec
TE 298.0 K
D1 1.00000000 sec
TD0 1
SFO1 400.1524709 MHz
NUC1 1H
P1 9.78 usec
PLW1 15.00300026 W

F2 - Processing parameters
SI 65536
SF 400.1500039 MHz
WDW EM
SSB 0
LB 0.30 Hz
GB 0
PC 1.00

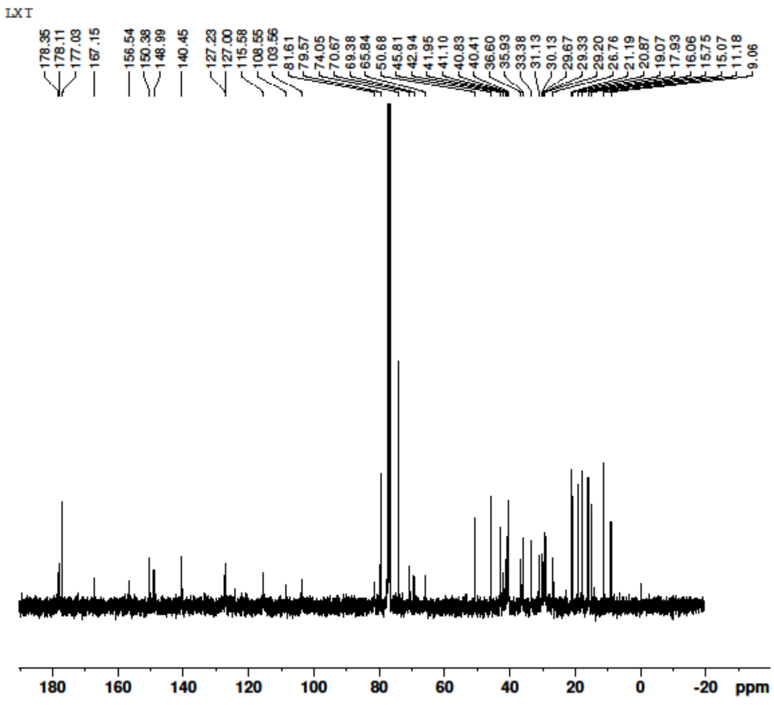

Current Data Parameters
NAME LXT-72
EXPNO 2
PROCNO 1

F2 - Acquisition Parameters
Date_ 20220105
Time 5.16 h
INSTRUM spect
PROBHD Z116098_0436 (
PULPROG zgpg30
TD 65536
SOLVENT CDCl3
NS 1024
DS 4
SWH 24038.461 Hz
FIDRES 0.733596 Hz
AQ 1.3631488 sec
RG 202.1
DW 20.800 usec
DE 6.50 usec
TE 298.0 K
D1 2.00000000 sec
D11 0.03000000 sec
TD0 1
SFO1 100.6278593 MHz
NUC1 13C
P1 10.58 usec
PLW1 68.07700348 W
SFO2 400.1516006 MHz
NUC2 1H
CPDPRG[2 waltz16
PCPD2 80.00 usec
PLW2 15.00300026 W
PLW12 0.22421999 W
PLW13 0.11260000 W

F2 - Processing parameters
SI 32768
SF 100.6177975 MHz
WDW EM
SSB 0
LB 1.00 Hz
GB 0
PC 1.40

152i

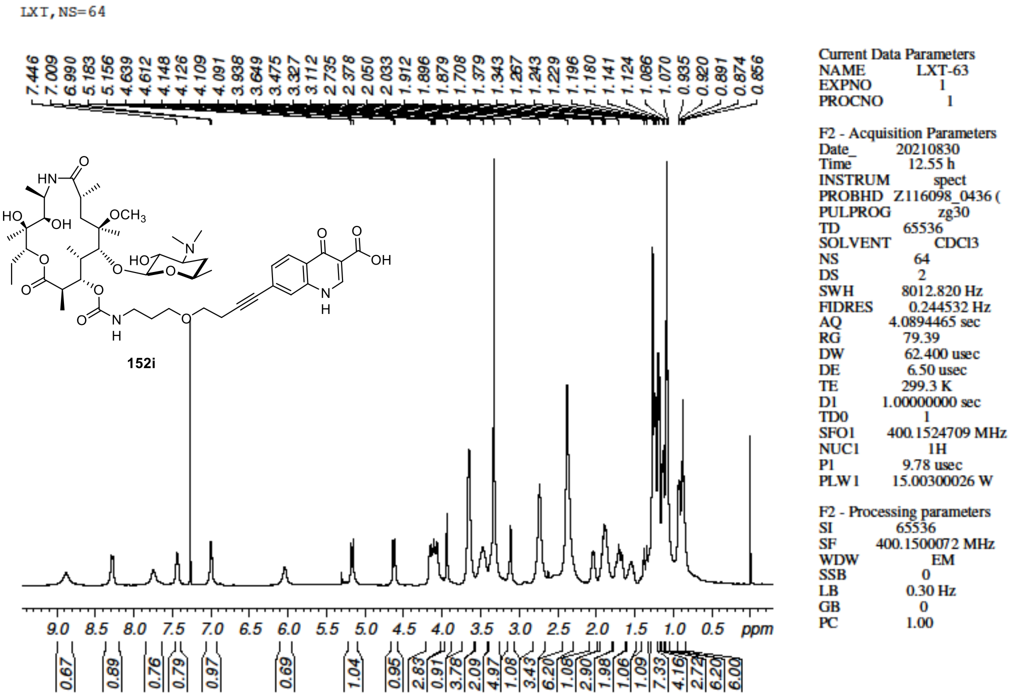
LXT,NS=64
152i
Current Data Parameters
NAME LXT-63
EXPNO 1
PROCNO 1
F2 - Acquisition Parameters
Date_ 20210830
Time 12.55 h
INSTRUM spect
PROBHD Z116098_0436 (
PULPROG zg30
TD 65536
SOLVENT CDCl3
NS 64
DS 2
SWH 8012.820 Hz
FIDRES 0.244532 Hz
AQ 4.0894465 sec
RG 79.39
DW 62.400 usec
DE 6.50 usec
TE 299.3 K
D1 1.00000000 sec
TD0 1
SFO1 400.1524709 MHz
NUC1 1H
P1 9.78 usec
PLW1 15.00300026 W
F2 - Processing parameters
SI 65536
SF 400.1500072 MHz
WDW EM
SSB 0
LB 0.30 Hz
GB 0
PC 1.00
ppm

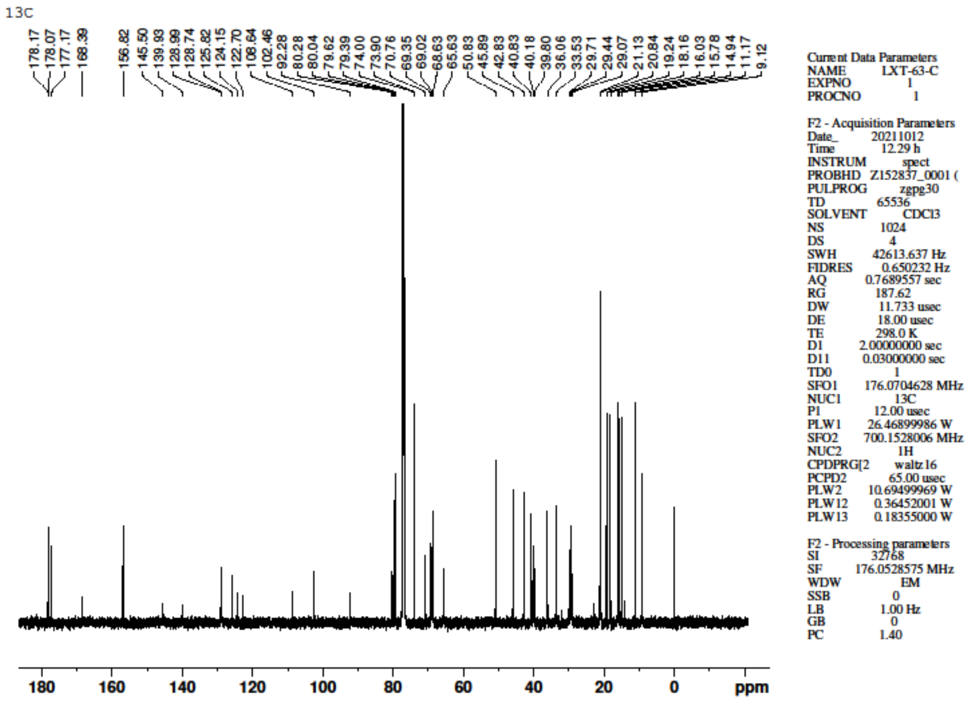
13C
Current Data Parameters
NAME LXT-63-C
EXPNO 1
PROCNO 1
F2 - Acquisition Parameters
Date_ 20211012
Time 12.29 h
INSTRUM spect
PROBHD Z152837_0001 (
PULPROG zgpg30
TD 65536
SOLVENT CDCl3
NS 1024
DS 4
SWH 42613.637 Hz
FIDRES 0.650232 Hz
AQ 0.7689557 sec
RG 187.62
DW 11.733 usec
DE 18.00 usec
TE 298.0 K
D1 2.00000000 sec
D11 0.03000000 sec
TD0 1
SFO1 176.0704628 MHz
NUC1 13C
P1 12.00 usec
PLW1 26.46899986 W
SFO2 700.1528006 MHz
NUC2 1H
CPDPRG[2 waltz16
PCPD2 65.00 usec
PLW2 10.69499969 W
PLW12 0.36452001 W
PLW13 0.18355000 W
F2 - Processing parameters
SI 32768
SF 176.0528575 MHz
WDW EM
SSB 0
LB 1.00 Hz
GB 0
PC 1.40
ppm
